# Breast Cancer-Derived Microparticles Reduce Cancer Cell Adhesion, an Effect Augmented by Chemotherapy

**DOI:** 10.3390/cells9102269

**Published:** 2020-10-10

**Authors:** Dvir Shechter, Michal Harel, Abhishek Mukherjee, Leonel M. Sagredo, David Loven, Elad Prinz, Shimrit Avraham, Veronique Orian-Rousseau, Tamar Geiger, Yuval Shaked, Haguy Wolfenson

**Affiliations:** 1 Department of Cell Biology and Cancer Science, Rappaport Faculty of Medicine, Technion–Israel Institute of Technology, Haifa 3525433, Israel; dshech@campus.technion.ac.il (D.S.); eladprinz@gmail.com (E.P.); shimritavra@gmail.com (S.A.); 2 Department of Human Molecular Genetics and Biochemistry, Sackler Faculty of Medicine, Tel Aviv University, Tel Aviv 6997801, Israel; harel.mic@gmail.com (M.H.); geiger@tauex.tau.ac.il (T.G.); 3 Department of Genetics and Developmental Biology, Rappaport Faculty of Medicine, Technion–Israel Institute of Technology, Haifa 31096, Israel; abhishekm@campus.technion.ac.il; 4 Karlsruhe Institute of Technology, Institute of Biological and Chemical Systems–Functional Molecular Systems (IBCS-FMS), 76021 Karlsruhe, Germany; leonel.munoz@kit.edu (L.M.S.); veronique.orian-rousseau@kit.edu (V.O.-R.); 5 Ha’Emek Medical Center, Department of Oncology, Afula 1834111, Israel; loven_da@clalit.org.il

**Keywords:** chemotherapy, breast cancer, adhesion, metastasis, CD44, extracellular vesicles

## Abstract

Tumor cell heterogeneity is primarily dictated by mutational changes, sometimes leading to clones that undergo a metastatic switch. However, little is known about tumor heterogeneity following chemotherapy perturbation. Here we studied the possible involvement of tumor-derived extracellular vesicles, often referred to as tumor-derived microparticles (TMPs), as mediators of the metastatic switch in the tumor microenvironment by hindering cell adhesion properties. Specifically, we show that highly metastatic or chemotherapy-treated breast cancer cells shed an increased number of TMPs compared to their respective controls. We found that these TMPs substantially reduce cell adhesion and disrupt actin filament structure, therefore increasing their biomechanical force pace, further implicating tumor cell dissemination as part of the metastatic cascade. Our results demonstrate that these pro-metastatic effects are mediated in part by CD44 which is highly expressed in TMPs obtained from highly metastatic cells or cells exposed to chemotherapy when compared to cells with low metastatic potential. Consequently, when we inhibited CD44 expression on TMPs by a pharmacological or a genetic approach, increased tumor cell adhesion and re-organized actin filament structure were observed. We also demonstrated that breast cancer patients treated with paclitaxel chemotherapy exhibited increased CD44-expressing TMPs. Overall, our study provides further insights into the role of TMPs in promoting metastasis, an effect which is augmented when tumor cells are exposed to chemotherapy.

## 1. Introduction

Although significant progress has been made in the last decades towards the development of novel anti-cancer therapies for the treatment of advanced metastatic disease, most cancer types are still incurable, with metastasis being the main cause of death. In breast cancer, the most frequently diagnosed cancer in women, approximately half a million cancer deaths due to metastasis are reported per year [1]. Although a minority of breast cancer patients are diagnosed with stage IV advanced metastatic incurable disease, approximately 30% of all breast cancer will develop metastasis within months and years after diagnosis [2]. Metastasis is a multi-step process that includes cancer cell dissemination from the primary tumor, intravasation to the blood or lymphatic system, survival in the circulation, extravasation to a target organ, and seeding and proliferation at a distant site [3,4]. These effects require a tight regulation of the cellular machinery that supports tumor cell detachment from the primary tumor and their binding to the metastatic site.

Microparticles have recently emerged as having a potentially significant role in tumor progression and metastasis. Microparticles belong to heterogeneous double layered membrane-coated particles exerted from cells, called extracellular vesicles (EVs) [5]. EVs encompass lipids, proteins, mRNA, non-coding RNA, and DNA. EVs have been mostly studied in the context of intercellular communication, whereby they transfer cargo between cells, including signaling proteins and RNA, as well as stimulate cells by membrane binding [5,6]. There are three main family members of EVs: apoptotic bodies (1000–4000 nm in diameter), microparticles (100–1000 nm in diameter), and exosomes (20–100 nm in diameter) [5,7]. EVs have been shown to affect cancer progression in various ways. For example, at remote secondary sites, the EVs are taken up by organ-derived cells, thereby preparing the microenvironment for future tumor cell seeding. These effects are associated with integrins expressed by EVs, which direct them to specific organs [8]. In melanoma, EVs derived from tumors contribute to the mobilization of myeloid cells to the pre-metastatic site and support metastasis [9]. Such pre-metastatic niche formation has been recently reported to be enhanced in response to chemotherapy [10]. At the primary tumor site, EVs promote tumor progression by transferring oncogenic proteins such as EGFRvIII between glioblastoma cells [11]. Thus, EVs play a significant role in tumor growth and metastasis.

When focusing on microparticles, it has been shown that tumor-derived microparticles (TMPs) obtained from multidrug-resistant breast cancer cells support immune evasion by polarizing macrophages into an inactive state. This process contributes to the colonization of breast cancer cells at distant sites [12]. In another study, TMPs were shown to selectively transfer p-glycoprotein to breast cancer cells, supporting their multidrug resistance capacity and therefore contributing to tumor growth [13]. We have recently demonstrated that breast cancer cells exposed to chemotherapy shed an increased number of TMPs expressing osteopontin. These TMPs support the mobilization and tumor homing of pro-angiogenic bone marrow-derived cells (BMDCs), ultimately enhancing tumor growth and metastasis [14]. However, little is known about the effect of TMPs on the biomechanical pro-metastatic forces of cells within the primary tumor site, which support their dissemination, and about the contribution of chemotherapy to this process.

Metastasis is regulated by an abundant number of proteins, which participate in each step of the metastatic process. Cell adhesion is one of the key factors contributing to the early stage of metastasis, when tumor cells disseminate from the primary tumor site. To do this, tumor cells reduce their adhesion ability and increase their biomechanical forces in order to allow their dissemination from the surrounding microenvironment. CD44 is a transmembrane glycoprotein which is expressed on the cell surface of many cancer cells [15]. CD44 confers several variant isoforms, some of which are found to be upregulated in tumor progression [16]. It has been demonstrated that the high expression of CD44 on cancer cells is associated with increased metastasis [17]. The interaction of CD44 with ECM ligands such as hyaluronic acid, osteopontin, collagens, and matrix metalloproteinases contributes to cell migration and invasion and supports metastasis [18]. Furthermore, the transfer of CD44/CD44v6 expressed by EVs supports tumor initiation and progression [19]. Similarly, CD44-expressing EVs also contribute to metastasis by remodeling the ECM within the primary tumor, supporting tumor cell invasion [20]. Thus, CD44 is an important regulator for the metastatic process.

Here we show that TMPs from highly metastatic tumor cells or cells exposed to chemotherapy enhance the metastatic characteristics of low-metastatic recipient cells. These effects include decreased focal adhesions, actin cytoskeleton rearrangement, and an increased number of catch/release cycles in the process of biomechanical force production, all driven by TMPs expressing CD44. Thus, this study describes a biomechanical mechanism that drives the metastatic switch in chemotherapy-treated tumors.

## 2. Materials and Methods

### 2.1. Cell Culture

MDA-MB-231 human breast carcinoma cells, 4T1 murine breast carcinoma cells, and HEK293T human embryonic kidney cells were purchased from the American Type Culture Collection (ATCC). The LM2-4 cell line, a lung metastatic variant of the MDA-MB-231 cell line, was kindly provided by Prof. Robert Kerbel (Sunnybrook Health Sciences Centre, Toronto, Ontario, Canada). 67NR, a low metastatic variant of the 4T1 cell line, was kindly provided by Prof. Jonathan Sleeman (Medical Faculty Mannheim, Centre for Biomedicine and Medical Technology Mannheim (CBTM), University of Heidelberg, Mannheim 68167, Germany). In some cases, cells were fluorescently tagged with green fluorescent protein (GFP). All cells were used within 6 months of resuscitation. Human cell lines were cultured in RPMI 1640 (Sigma-Aldrich, Rehovot, Israel) and murine cell lines in Dulbecco’s modified Eagle’s medium (Sigma-Aldrich). In all cases, medium was supplemented with 5% fetal bovine serum (FBS), 1% l-glutamine, 1% sodium pyruvate, and 1% streptomycin, penicillin, and neomycin in solution (10 mg/mL, Biological Industries, Beit, Ha’Emek, Israel). Cells were tested routinely to be mycoplasma-free. Human cell lines were tested to be authentic or they were used within 6 months from resuscitation.

### 2.2. Extraction and Quantification of TMP

The extraction and quantification of TMPs were carried out as previously demonstrated and validated [13,14,21,22]. Briefly, cells were grown to 80% confluency, at which point medium was replaced with serum free (SF) medium or SF medium supplemented with 200 nM paclitaxel (PTX), as previously described [14]. After 48 h, conditioned medium (CM) was collected and centrifuged at 4000× *g* for 20 min at room temperature (RT) to remove floating cells and apoptotic bodies. Supernatants were collected and centrifuged at 20,000 *g* for 1 h at 4 °C. The TMP-containing pellet was resuspended in phosphate buffered saline (PBS) and stored at −80 °C until further use. It should be noted that the dosage of 200 nM PTX for a duration of 48 h in serum-free conditions did not result in cell death, excluding the possibility that samples were contaminated with apoptotic bodies, as previously described [14].

TMP quantification was performed using flow cytometry by calculating the ratio between 7.35-µm counting beads (Calbiochem, Burlington, MA) and the number of events collected in the TMP gate (approximately 0.6–0.9 µm), as previously described [14,23]. Additional information can be found in the Supplemental Online Materials.

Quantification and measurement of TMPs by Nanosight NS300 (NanoSight LTD., Malvern, UK) was performed as previously described [24]. Additional information can be found in the Supplemental Online Materials.

### 2.3. Modified Boyden Chamber Assay

The invasion properties of MDA-MB-231 or 67NR cells pre-exposed to different TMP conditions were evaluated in Matrigel-coated Boyden chambers as previously described [25]. Additional information can be found in the Supplemental Online Materials.

### 2.4. Cell Spreading Assay

TMPs were pre-exposed to 1 μg/mL IgG or anti-CD44 (BioXcell, West Lebanon, NH) for 1 h, followed by extensive washing, in which a volume of 100 times PBS was used. The TMPs were then added to MDA-MB-231 GFP+ cultures and incubated for 24 h. Subsequently, the cells were trypsinized and re-plated on fibronectin-coated glass plates. Time-lapse movies were generated from images acquired every 5 min for a total of 4 h using an ImageXpress Micro Confocal system (Molecular Devices, San Jose, CA). The percentage of cells spreading over fibronectin was analyzed using FIJI, as previously described [26].

### 2.5. Cell Viability AlamarBlue^TM^ Assay

The metabolic indicator dye AlamarBlue^TM^ (Serotec Ltd., Oxford, UK) was used to determine cell viability, as previously described [27]. Additional information can be found in the Supplemental Online Materials.

### 2.6. Pillar Fabrication

Pillar fabrication was performed as previously described [26,28]. Briefly, PDMS (Sylgard 184, Dow Corning; 10:1 base to curing agent ratio) was poured over silicon molds with wells that were 1.3 µm deep, 0.5 µm wide, and spaced 1 µm apart (center-to-center distance). The molds were then flipped over onto glass-bottom dishes (no. 0 glass coverslip, Cellvis), which were then placed at 60 °C for 12 h to cure the PDMS. The molds were peeled off from the plates while immersed in pure ethanol, which was then replaced by PBS. The pillars were coated with fibronectin (10 µg/mL, 1 hr, 37 °C). To measure cellular forces on the fibronectin-coated pillars, serum-starved MDA-MB-231 cells were cultured for 24 h in the presence of 100,000 TMPs obtained from untreated or PTX-treated MDA-MB-231 cells, or from untreated or PTX-treated LM2-4 cells. The cells were then spread on the pillar arrays and imaged at 37 °C using a Leica DMIRE2 microscope (Leica Microsystems, Wetzlar, Germany), 100× 1.4 NA oil objective, and a CCD camera (QImaging Retiga EXi). Analyses of pillar movements was performed with ImageJ (V.1.51, National Institutes of Health, Bethesda, MD, USA) using the Nano Tracking plugin as previously described [26]. Pillar displacement curves were generated using Matlab (V.9.4, MathWorks, Natick, MA, USA). Analyses of pillar releases by the cells was performed on the pillar displacement curves using the ‘findpeaks’ function in Matlab. All peaks identified were categorized into bins ranging from 1 to 12 nm, and the number of releases per second was calculated for each of these bins. For each condition >30 pillars from >3 cells were analyzed.

### 2.7. CD44 Short Hairpin RNA

Several different short hairpin RNA (shRNA) sequences specific to human CD44 or a scrambled control sequence were cloned into the GIPZ Lentiviral Human shRNA plasmid (Horizon Discovery, Lafayette, CO, USA), as previously described [29]. Subconfluent LM2-4 cells were transfected with shRNA plasmids using lipofectamine reagent (Thermo Fisher Scientific, MA, USA) according to the manufacturer’s instructions. Forty-eight hours post-transfection, cells were incubated in growth medium containing puromycin (1 µL/mL) for the selection of stable transfectants. After 3 weeks of selection, the protein level of CD44 was evaluated by flow cytometry using appropriate controls. Two independent CD44-depleted clones generated from the pooled cells were selected for this study.

### 2.8. Western Blot

Paxillin and phospho-paxillin analysis was performed as follows. MDA-MB-231 cells were pre-exposed to 100,000 TMPs for 24 h, and then seeded on a fibronectin (20 µg/mL) coated plate overnight. Subsequently, the cells were lysed in Hepes 50 mM PH-7.5, EDTA 4 mM, Triton 1%, 0.5 mg/mL Na_3_VO_4_, and 4.5 mg/mL Na_2_P_2_O_7_ and lysates were subjected to SDS-PAGE. Proteins were electro-transferred to nitrocellulose membranes, which were then probed with polyclonal rabbit anti-paxillin (1:1000; catalog number 2542, Cell Signaling Technologies, Danvers, MA, USA), anti-phospho-paxillin (1:1000; catalog number 2541, Cell Signaling Technologies), or anti-GAPDH (1:5000, catalog number sc-25778, Santa Cruz Biotechnology Inc., Dallas, TX, USA). GAPDH was used for loading controls. All experiments were performed in triplicate.

### 2.9. Tumor-Derived Microparticle Proteomics

TMP proteomics were assessed and analyzed in accordance with the previously described method [30]. Briefly, TMPs were isolated by multiple centrifugations of conditioned media from MDA-MB-231 and LM2-4 cells cultured in serum-free media in the presence or absence of PTX, as described above. Pellets were immediately resuspended in 200 µL of lysis buffer (6 M urea, 2 M thiourea in 0.1 M Tris pH 7.6). Protein concentration was calculated based on the Bradford assay, and 10 μg of each sample was used for the analysis. The proteins were reduced using 1 mM DTT and alkylated using 5 mM iodoacetamide, followed by overnight in-solution trypsin/LysC digestion. The peptides were acidified with 0.1% TFA and purified on C18 stageTips [31]. A total of three biological replicates were prepared for each system, some of which were analyzed with two technical replicates, and were later combined into a single data point.

Resulting peptides were analyzed by LC-MS/MS on the EASY-nLC1000 UHPLC system (Thermo Fisher Scientific) coupled to the Q-Exactive Plus mass spectrometer (Thermo Fisher Scientific). The peptides were separated using a gradient of 140 min (technical replicate I) or 240 min (technical replicate II) of water-acetonitrile on a 50 cm EASY-spray column. Data were acquired using a top-10 method in a data-dependent mode. Resolution values were 70,000 and 17,500 in the MS and MS/MS scans, respectively.

MS raw files were processed using MaxQuant version 1.5.6.9 [32] and the Andromeda search engine [33]. MS/MS spectra were searched against the human uniprot database on a forward and decoy database with 1% false discovery rate for both the protein and the peptide levels. The ‘match between runs’ option was enabled to transfer identifications between runs. The label-free algorithm was used for protein quantification. Technical replicates were averaged into a single biological replicate during the MaxQuant data processing. The proteinGroups output was further analyzed using Perseus software [34]. Label-free quantification (LFQ) intensity values [35] were log2 transformed. Each comparison (MDA-MB-231 vs. MDA-MB-231 PTX or MDA-MB-231 vs. LM2-4) was processed separately following data filtration that kept only those proteins that were quantified in at least two of the three replicates in at least one of the sample groups. Student’s *t*-test was performed with permutation-based false discovery rate (FDR) correction with a cutoff of 0.1 and S0 correction of 0.2 [36]. Enrichment analysis was done using the Fisher exact test with an FDR cutoff of 0.02. Principal component analysis (PCA) was performed following data imputation, by replacing the missing values with values that formed a normal distribution with a downshift of 1.6 standard deviations and a width of 0.4 of the original data distribution.

### 2.10. Ex Vivo Pulmonary Metastasis Assay

The ex vivo pulmonary metastasis assay (PuMA) was performed as previously described [37,38]. Additional information can be found in the Supplemental Online Materials.

### 2.11. Blood Samples from Cancer Patients

The study was approved by the hospital’s Ethics Committee (Ha’Emek medical center), and written informed consent was obtained from all involved patients, in compliance with the Helsinki declaration for human studies. Patients with localized breast carcinoma were treated by neoadjuvant chemotherapy (before tumor removal), consisting of adriamycin and cyclophosphamide followed by PTX chemotherapy, at the Ha’Emek Medical Center, Afula, Israel. Blood was collected in 5-mL sodium citrate (3.2%) tubes before the first cycle of PTX chemotherapy and 24 h post-treatment (*n* = 15). Plasma was obtained by centrifugation at 1000 *g* for 10 min at RT. Subsequently, platelet-poor plasma (PPP) was extracted by plasma centrifugation at 3000 *g* for 5 min at RT, as previously described [39]. This procedure eliminated most of the platelets (<10,000 per μL). To ensure purified PPP, another centrifugation (3000 *g* for 5 min) was performed on separated PPP at RT. Subsequently PPP was stored at −80 °C. Thawed PPP was centrifuged at 20,000 *g* for 1 h, and the pellet was resuspended in PBS. Anti-MUC-1 antibody and anti-CD44 antibody were added according to the manufacturer’s instructions and were subsequently analyzed by flow cytometry as previously described [14]. A fluorescence-minus-one control (FMO) was used to determine nonspecific binding of antibodies, as previously described [40].

### 2.12. Immunostaining and Imaging

Serum-starved cells were cultured for 24 h in the presence of 100,000 TMPs obtained from untreated or PTX-treated cells, as indicated in the text. In some experiments, TMPs were cultured with cells for 24 h in serum-free medium in the presence of anti-CD44 antibodies (1 μg/mL). Cells were rigorously washed in PBS (100-fold volume), and then seeded on fibronectin-coated plates (20 µg/mL fibronectin, Biological Industries). After 4 h, cells were fixed using 4% paraformaldehyde (PFA) and immunostained with a primary anti-vinculin antibody (1:100, Sigma-Aldrich) and Cy2-conjugated secondary antibody. Actin was stained with Alexa 488 conjugated phalloidin (1:100, Invitrogen, Carlsbad, CA, USA). Images were acquired with a LSM 700 Zeiss confocal microscope (Zeiss Ltd. Oberkochen, Germany).

### 2.13. Statistical Analysis

Data are presented as mean ± standard error (SE). All in vitro studies were performed at least in three biological replicates. The in vivo studies were performed twice. Statistically significant differences were assessed by one-way ANOVA, followed by the Tukey post-hoc test (when comparing between more than two groups) using GraphPad Prism (V.6, La Jolla, CA, USA). When applicable, an estimate of variance was performed and statistical significance comparing only two sets of data was determined by the two-tailed Student’s *t*-test. Significance was set at values of *p* < 0.05, and designated as follows: * *p* < 0.05; ** *p* < 0.01; *** *p* < 0.001.

## 3. Results

### 3.1. TMPs from Highly Metastatic or Chemotherapy-Treated Tumor Cells Induce Tumor Cell Invasion

To study the effect of TMPs on the metastatic potential and the adhesion properties of tumor cells, we compared TMPs from MDA-MB-231 cells with their highly metastatic variant, LM2-4 cells [41], or in the presence or absence of PTX chemotherapy. TMPs were extracted from the medium and quantified by flow cytometry and NanoSight. Although the number of TMPs extracted from highly metastatic cells or from cells exposed to chemotherapy was higher than control cells, as previously reported [10,14], their size was not significantly different as evaluated by NanoSight (Figure S1). Based on their size, we ruled out the possibility that some of the TMPs detected are apoptotic bodies, as also previously reported [14].

**Figure 1:**
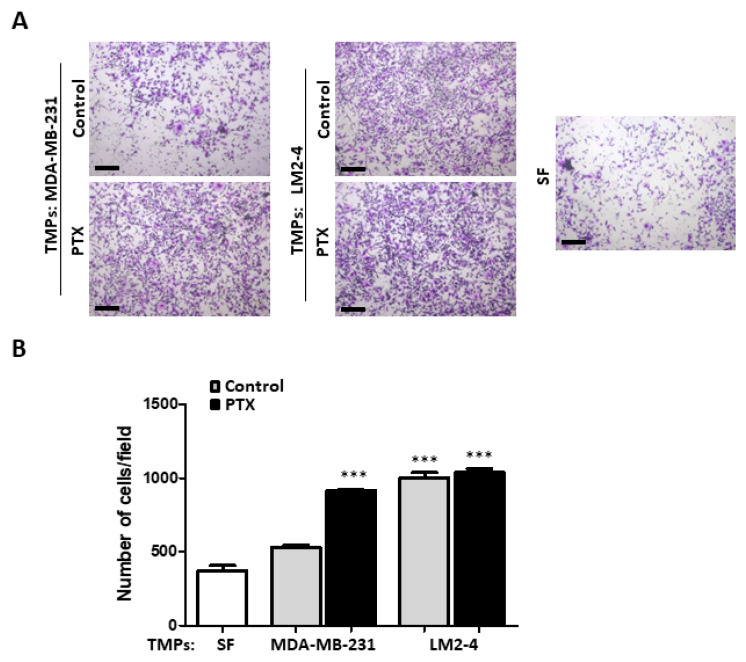
**Tumor-derived microparticles (TMPs) from highly metastatic cells or cells exposed to chemotherapy increase cell invasion**. (**A**,**B**) MDA-MB-231 cells cultured with serum-free medium or TMPs from MDA-MB-231 or LM2-4 cells exposed to paclitaxel (PTX) or vehicle control were assessed for invasion properties using the Boyden chamber assay. Representative images of invading cells are shown in (**A**). Scale bar, 200 µm. Quantifications of invading cells are shown in (**B**) (*n* = 5 repeats and 7 images/repeat). *, differences compared to MDA-MB-231 control. ***, *p* < 0.001, as assessed by one-way ANOVA followed by Tukey post-hoc test.

We next postulated that TMPs from highly metastatic cells or from cells exposed to chemotherapy could augment the metastatic potential of low-metastatic cells. To this end, TMPs were extracted from MDA-MB-231 and LM2-4 cells which had been treated with PTX or the vehicle control. Subsequently, these TMPs were cultured with low-metastatic MDA-MB-231 cells for 24 h and further analyzed for invasion properties using the Boyden chamber assay. TMPs from PTX-treated MDA-MB-231 cells enhanced the invasive properties of MDA-MB-231 cells in comparison to control cultures, including TMPs from non-treated MDA-MB-231 cells or serum-free conditions. Furthermore, MDA-MB-231 cells cultured with TMPs from control or PTX-treated LM2-4 cells exhibited the highest invasive ability (Figure 1A,B). Notably, TMPs from PTX-treated cells displayed induced invasion properties regardless of the metastatic potential of the tumor cells, implicating a potent effect of chemotherapy on TMP-induced cell invasion. Similar results were observed when using 4T1 highly metastatic cells and their low metastatic 67NR cells (Figure S2). Of note, TMPs had no effect on cell proliferation (Figure S3). Taken together, these results suggest that TMPs affect tumor cell invasion properties.

### 3.2. TMPs from Highly Metastatic or Chemotherapy-Treated Tumor Cells Inhibit Tumor Cell Seeding

**Figure 2:**
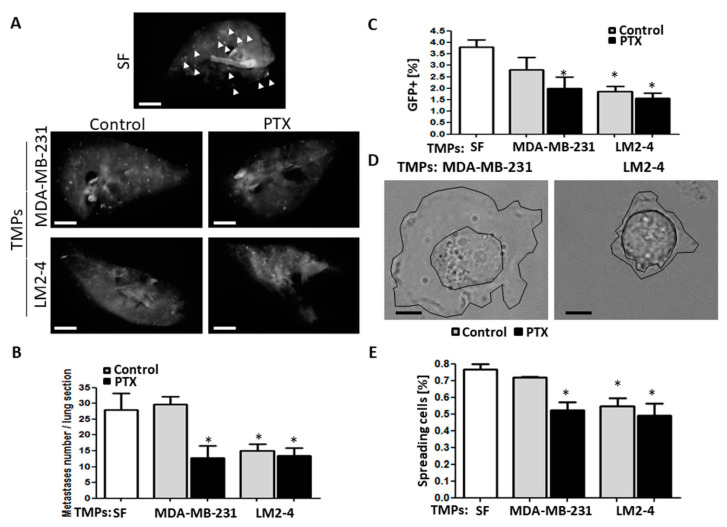
**TMPs from highly metastatic cells or cells exposed to chemotherapy decrease tumor cell seeding.** (**A**–**C**) GFP-positive MDA-MB-231 cells cultured with serum-free medium or TMPs from MDA-MB-231 or LM2-4 cells exposed to PTX or vehicle control were assessed for their seeding properties using the ex vivo pulmonary metastasis assay (PuMA), as described in Materials and Methods. Lung sections (*n* = 4 mice/group) were imaged (**A**) and the number of metastatic foci were counted (**B**). Scale bar, 0.2 cm. White arrows point at metastatic foci only in the serum-free group. The percentage of metastatic cells in the lungs was also assessed by flow cytometry after the lung sections underwent single cell suspension (**C**). (**D**,**E**) Cells (treated as in **A**–**C**) were seeded on fibronectin-coated plates. Cell spreading was then immediately assessed by time-lapse microscopy, in which the area of cell spread was displayed. Representative images are shown in (**D**). Scale bar, 20 µm. Cell cytoplasmic membrane and nucleus are drawn in black lines. The percentage of spreading cells when cultured on fibronectin is shown in (**E**). *n* = 3 repeats/group with the assessment of ~100 cells/repeat. * differences compared to MDA-MB-231 control group, *, *p* < 0.05, as assessed by one-way ANOVA followed by Tukey post-hoc test.

We next sought to test if TMPs affect the seeding of low metastatic cells in distant tissues. We therefore performed an ex vivo pulmonary metastasis assay (PuMA), which primarily evaluates the seeding potential of tumor cells in the lungs [38]. GFP-expressing MDA-MB-231 cells pre-cultured for 24 h with TMPs from vehicle- or PTX-treated MDA-MB-231 or LM2-4 cells were injected through the tail vein of naïve mice to perform the PuMA. The PuMA can be used a method to test the ability of cells to adhere to a tissue, therefore it is suitable to test the early stage of metastasis, when tumor cells detach from the primary tumor microenvironment. We found that tumor cell seeding represented by the number of GFP+ foci in the lungs was reduced when the MDA-MB-231 cells were pre-cultured with TMPs from PTX-treated MDA-MB-231 cells or with TMPs from vehicle- or PTX-treated LM2-4 cells (Figure 2A,B). These results were also supported by flow cytometry analysis. Specifically, a reduced percentage of GFP+ cells in the whole lung tissue was observed in mice injected with MDA-MB-231 cells pre-cultured with TMPs from PTX-treated MDA-MB-231 cells or with TMPs from vehicle- or PTX-treated LM2-4 cells compared to control MDA-MB-231 cells (Figure 2C). Importantly, the short time-interval between cell injection and animal sacrifice suggests that the TMPs lowered the cells’ adhesive ability. These results implicate an early process of metastasis at the primary tumor site where the cells detached from their surrounding microenvironment. To support these results, we found that cells exposed to TMPs from PTX-treated MDA-MB-231 cells or to TMPs from vehicle- or PTX-treated LM2-4 cells spread to a much lower extent when seeded on fibronectin-coated plates, compared to control conditions (Figure 2D,E). This property directly relates to the inability to form strong cell-matrix adhesions [26]. Once again, TMPs from PTX-treated cells displayed similar reduced tumor cell adhesion properties, regardless of the metastatic potential of the tumor cells, therefore suggesting a strong effect of chemotherapy on TMPs which hinder cell adhesion.

### 3.3. TMPs from Highly Metastatic or Chemotherapy-Treated Tumor Cells Inhibit Cellular Adhesion and Destroy Actin Filaments

**Figure 3:**
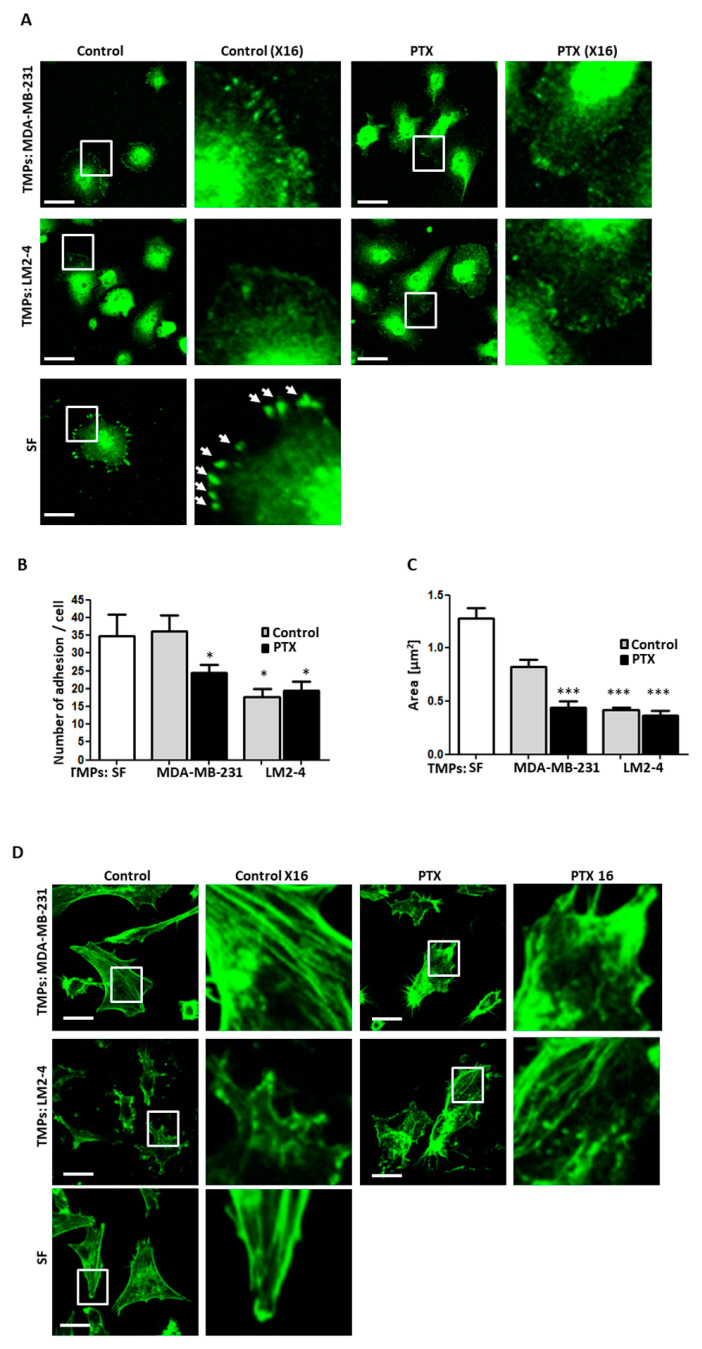
**TMPs from highly metastatic cells or cells exposed to chemotherapy decrease focal adhesions and disrupt filament structure**. (**A**–**C**) MDA-MB-231 cells cultured with TMPs from MDA-MB-231 or LM2-4 cells exposed to PTX or vehicle control were seeded on fibronectin-coated plates to assess focal adhesion plaques by vinculin immunostaining (green). MDA-MB-231 cells cultured with serum-free (SF) medium served as a control. Representative images (left panel) with ×16 zoom micrographs (right panel) are shown. Scale bar, 20 µm. White arrows point at focal adhesion plaques in the serum-free group (**A**). The number of focal adhesions per cell was quantified (**B**). Cell area was calculated (**C**). *n* = 10 fields/group. (**D**) Cells as in A were stained with phalloidin (green) to assess actin filament structure. Representative images are shown (right panel) with ×16 zoom micrographs (left panel). Scale bar 20 µm. *n* = 5 fields/group. * differences compared to MDA-MB-231 control group, *, *p* < 0.05; ***, *p* < 0.001 as assessed by one-way ANOVA followed by Tukey post-hoc test.

Metastatic cells acquire the ability to detach from the primary tumor by inhibiting adhesion molecules and rearranging the actin cytoskeleton to promote the migratory properties [42]. To study the effect of TMPs on the cell adhesion, MDA-MB-231 cells were cultured for 24 h in the presence of TMPs from vehicle- or PTX-treated MDA-MB-231 or LM2-4 cells. Then, cells were seeded on fibronectin-coated plates. After 4 h, cells were fixed and stained with an anti-vinculin antibody and fluorescently-labeled phalloidin to label focal adhesions by vinculin expression and the actin cytoskeleton, respectively. A decreased number of adhesions in cells cultured with TMPs from PTX-treated MDA-MB-231 cells or with TMPs from vehicle- or PTX-treated LM2-4 cells were observed based on vinculin expression (Figure 3A,B). In addition, cell area was reduced in the presence of TMPs from all groups in comparison to control cells cultured in the absence of TMPs (Figure 3C). Lastly, cells cultured with TMPs from PTX-treated MDA-MB-231 cells or with TMPs from vehicle- or PTX-treated LM2-4 cells demonstrated less organized and elongated actin stress fibers. These fibers were displayed with shorter actin filaments positioned in multiple directions, compared to those observed in control cells (Figure 3D). Thus, TMPs support metastatic cell characteristics by means of downregulating the formation of mature adhesion plaques and less organized actin cytoskeleton.

### 3.4. TMPs Increase the Pace of Metastatic Cell Biomechanical Forces

**Figure 4:**
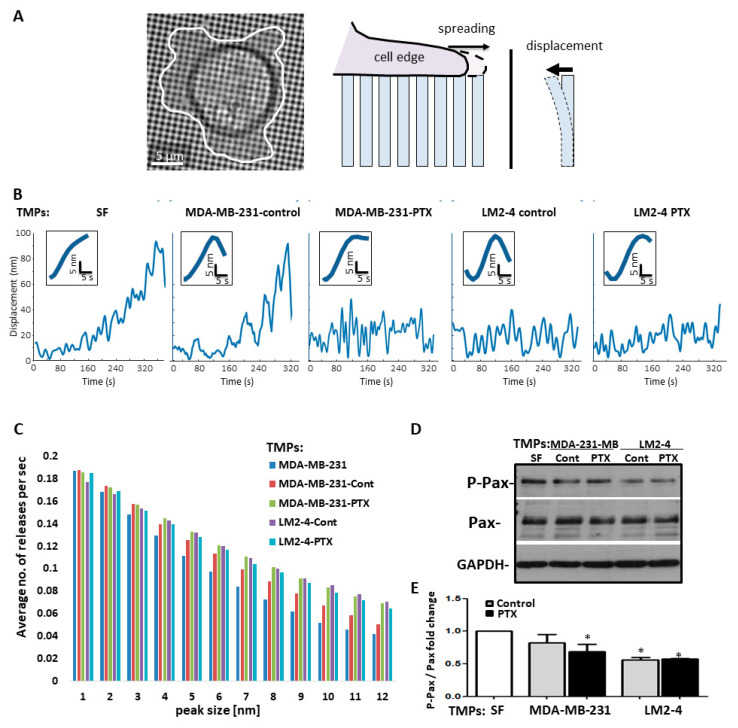
**TMPs from highly metastatic cells or cells exposed to chemotherapy increase the pace of cell biomechanical forces and inhibit focal adhesion signaling**. (**A**–**C**) MDA-MB-231 cells cultured with TMPs from MDA-MB-231 or LM2-4 cells exposed to PTX or vehicle control were applied on a pillar assay, as described in Materials and Methods. MDA-MB-231 cells cultured with serum-free medium (SF) were used as a control. (**A**) Left: Example of a cell spreading on an array of 0.5 µm diameter fibronectin-coated pillars (see illustration on the right). (**B**) Typical pillar displacement curves. (**C**) Histograms presenting the average number of pillar releases per second binned according to the size of the release. Each of the control conditions was significantly different than the conditions in which the cells were exposed to TMPs from LM2-4 cells or from cells exposed to PTX (α < 0.01; Kolmogorov–Smirnov test). *n* = 8–10 repeats/group. (**D**–**E**) Cells treated as above were seeded on fibronectin-coated plates to evaluate phospho-paxillin (P-Pax) and total-paxillin (Pax) expression by Western blot. GAPDH was used as a loading control (**D**). The ratio between P-Pax over Pax was calculated based on densitometry, and presented as fold change, for three biological repeats (**E**). *n* = 3 repeats/group. * differences compared to MDA-MB-231 control group, * *p* < 0.05, as assessed by one-way ANOVA followed by Tukey post-hoc test.

Since there is reciprocal regulation between adhesions and their associated force-producing actin cytoskeleton [43], we sought to determine which of these structures was affected by TMPs, thereby decreasing adhesion and promoting cell motility. To test this, we evaluated pillar displacement when using MDA-MB-231 cells in different conditions, as demonstrated in Figure 4A. Specifically, when MDA-MB-231 cells were cultured with TMPs from LM2-4 or from PTX-exposed cells, they pulled on the pillars at a similar pace compared to control cells. However, they could not maintain their hold, leading to repeated pull/release cycles that occurred at a higher rate in comparison to control cells (Figure 4B), thus demonstrating a significant increase in the average number of pillar releases per second (Figure 4C). Since defects in cytoskeletal assembly lead to changes in the rate of force application, whereas defects in adhesions lead to their breakage [26], we concluded that exposure to TMPs from LM2-4 or from PTX-exposed cells directly decreases cell adhesion properties, rendering the adhesions too weak to withstand cytoskeletal forces and prevent the formation of stress fibers. Furthermore, Western blot analysis of cell lysates revealed significantly lower adhesion-associated paxillin signaling in cells cultured with TMPs from PTX-treated MDA-MB-231 cells or with TMPs from vehicle- or PTX-treated LM2-4 cells (Figure 4D,E). These results suggest that adhesion is reduced in cells exposed to TMPs from PTX-treated cells or highly metastatic cells. Overall, our results demonstrate that TMPs from PTX-treated low metastatic cells or from highly metastatic cells (regardless of chemotherapy treatment) disrupt cell adhesion, which is consistent with cell dissemination and an increased ability to invade.

### 3.5. Proteomic Analysis of TMPs

**Figure 5:**
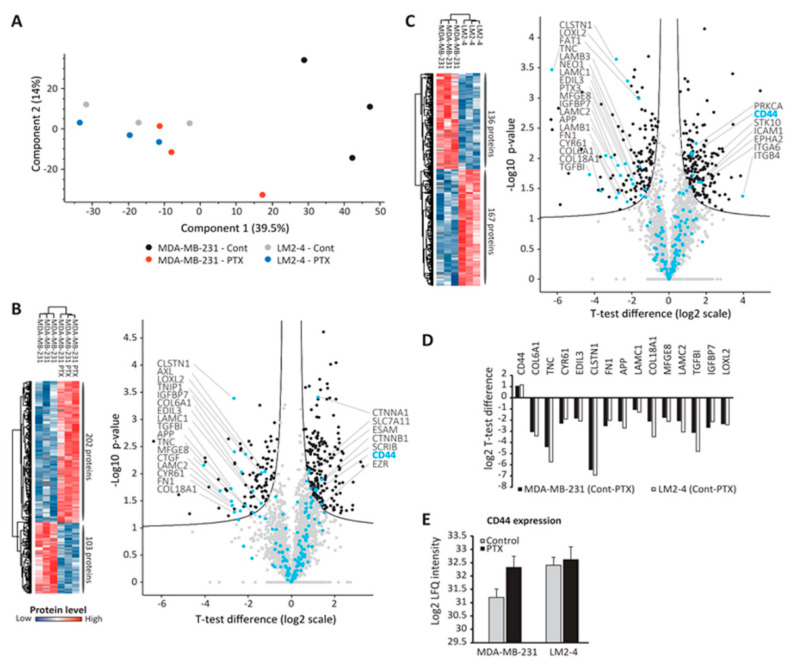
**CD44 is a differentially expressed protein found in highly metastatic and PTX-treated TMPs.** TMPs collected from MDA-MB-231 or LM2-4 cells exposed to PTX, or not, were analyzed by mass spectrometry. (**A**) Principal component analysis shows clear separation between MDA-MB-231 control and the rest of the samples. (**B**,**C**) Heatmap (left) and volcano plot (right) for the comparison between control and PTX-treated MDA-MB-231 (**B**) or control MDA-MB-231 and LM2-4 (**C**). In blue- “cell adhesion” proteins based on Gene Otology Biological Process category. (**D**) *t*-test difference (log2 scale) of the “cell adhesion” proteins that were common to both comparisons. (**E**) CD44 label-free quantification (LFQ)-intensity in MDA-MB-231 and LM2-4 cells with and without PTX. The proteomic analysis was performed when *n* = 3 repeats / group. All the results and graphs presented are statistically significant calculated by *t*-test and false discovery rate (FDR) test described in Materials and Methods.

We next undertook a proteomic approach to identify factors within TMPs that affect cell adhesion, when the major focus was placed on adhesion-related proteins. To this end, TMPs were isolated from MDA-MB-231 and LM2-4 cells (untreated or treated with PTX) and processed for high-resolution mass-spectrometry-based proteomic analysis. Overall, 5209 proteins were identified, with control MDA-MB-231 TMPs exhibiting the lowest number of proteins (Table S1). PCA further reflected the large variation between control MDA-MB-231 TMPs and all other TMP groups (Figure 5A). We then performed two comparisons. In the first, we compared the proteomes of TMPs derived from control and PTX-treated MDA-MB-231 cells. In the second, we compared the proteomes of TMPs derived from control MDA-MB-231 cells and untreated LM2-4 cells. Over 300 significantly altered proteins were detected in each comparison (Figure 5B,C). When specifically focusing on adhesion membrane proteins of which the levels were significantly changed, 15 proteins were common to both comparisons (Figure 5D). Fourteen of these proteins were higher in MDA-MB-231 control TMPs. Interestingly, out of the 15 cell adhesion proteins common to both comparisons, only one, CD44, was significantly increased in TMPs from PTX-treated MDA-MB-231 or untreated LM2-4 cells (Figure 5D). Its intensity in these TMPs was over two-fold higher than in TMPs derived from control MDA-MB-231 cells (Figure 5E). Altogether, these results suggest that CD44, probably among other proteins that are significantly differentially expressed between the various TMP groups, is a possible co-effector that can potentially account for the disruption of cell adhesion induced by TMPs.

### 3.6. CD44-Expressing TMPs Decrease Cell Adhesion and Support Tumor Cell Metastatic Properties

CD44 is a glycoprotein known to participate in cell adhesion and migration [44,45]. Therefore, increased levels of CD44 in TMPs may explain the TMP-induced increased adhesive and invasive properties of tumor cells. To test this, TMPs derived from untreated or PTX-treated MDA-MB-231 or LM2-4 cells were incubated with CD44 blocking antibodies or IgG control antibodies for 24 h. Excess antibodies were removed by rigorous washing when adding a 100-fold volume of PBS to the sample, followed by centrifugation to obtain TMPs. Subsequently, the TMPs were added to MDA-MB-231 cultures, and invasive and adhesive properties were assessed. Pre-treating TMPs with anti-CD44 antibodies restored the invasive properties of TMP-exposed tumor cells to control levels (Figure S4A,B). Similar results were obtained when TMPs from 4T1 cells were pre-treated with anti-CD44 antibodies and subsequently cultured with 67NR (low metastatic) cells (Figure S4C,D).

**Figure 6:**
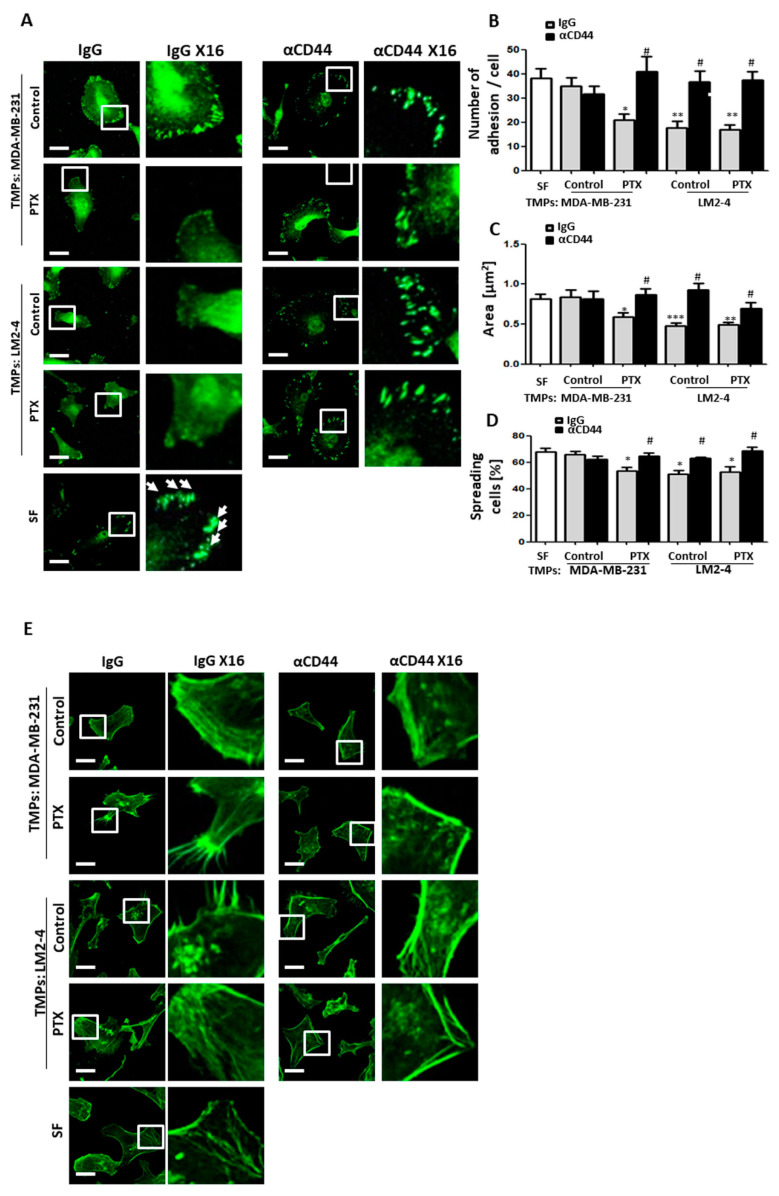
**TMPs expressing CD44 decrease tumor cell adhesion and promote organized actin filament structure.** TMPs from MDA-MB-231 or LM2-4 cells which had been exposed to paclitaxel (PTX) or vehicle control were incubated with anti-CD44 (5 μg/mL) or control IgG and subsequently cultured with MDA-MB-231 cells. As a control, MDA-MB-231 cells were cultured alone in serum-free medium (SF). (**A**–**C**) The cells were seeded on fibronectin-coated plates to assess focal adhesion plaques by vinculin immunostaining (green). Representative images are shown (left panel) with ×16 zoom micrographs (right panel). Scale bar, 20 µm. White arrows point at focal adhesion plaques only in the serum-free group (**A**). The number of focal adhesions per cell was quantified (**B**). Cell area was calculated (**C**). *n* = 10–11 fields/group. (**D**) Cell spreading was analyzed with time-lapse microscopy. The percentages of spreading cells per field are shown. *n* =5 repeats/group. (**E**) Cells as in (**A**–**C**) were stained with phalloidin (green) to assess actin filament structure. Representative images are shown (right panel) and ×16 zoom micrographs (left panel). Scale bar, 20 µm. *n* = 5 fields/group. * differences compared to MDA-MB-231 control group, * *p* < 0.05; ** *p* < 0.01; *** *p* < 0.001, # differences between IgG and anti-CD44 of the same group, as assessed by one-way ANOVA followed by Tukey post-hoc test.

Next, TMPs pre-treated with anti-CD44 were added to MDA-MB-231 cultures, and the number of focal adhesions, assessed by vinculin, cell area, efficiency of cell spreading, and the phenotype of actin stress fibers, was evaluated. Blocking CD44 on TMPs from highly metastatic cells or from cells exposed to chemotherapy restored the conditions found in control MDA-MB-231 cells. Specifically, CD44 inhibition on TMPs increased focal adhesion plaques assessed by vinculin, cell area, and percentage of cell spreading (Figure 6A–D). Notably, the cell size reduction noticed in MDA-MB-231 cells exposed to TMPs from PTX-treated or LM2-4 cells is in line with cell spreading, demonstrated in Figure 2D. Furthermore, TMPs in which CD44 was inhibited resulted in rearrangement of actin stress fibers, mimicking those found in control serum-free conditions (Figure 6E). Similar results were obtained when TMPs from highly metastatic cells were knocked down for CD44 and subsequently were added to MDA-MB-231 cultures. Specifically, clones 1 and 2 demonstrated an increased number of focal adhesion plaques and better organization of actin filaments when compared to scrambled control cells (Figure S5). Taken together, these results further indicate that CD44-expressing TMPs contribute to the inhibition of cell adhesion and cytoskeleton re-arrangement, and thus enhance the metastatic properties of tumor cells.

### 3.7. TMPs Expressing CD44 were Increased in Breast Cancer Patients Treated with Chemotherapy

**Figure 7:**
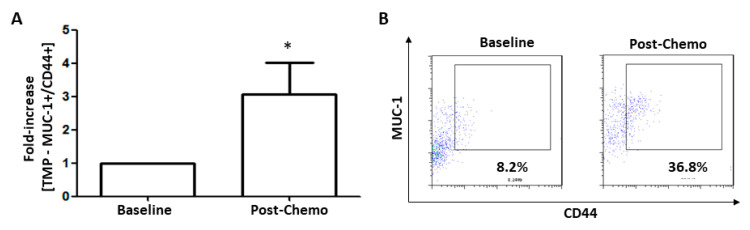
**Elevated CD44 expression in peripheral TMPs from breast cancer patients who underwent chemotherapy**. (**A**,**B**) Blood was collected from breast cancer patients at baseline, and 24 h after the first cycle of paclitaxel therapy (*n* = 15). The percentage of TMPs (expressing both MUC-1 and CD44) was evaluated by flow cytometry in baseline and post-chemotherapy platelet-poor plasma samples. Fold-increase of TMPs (MUC-1+/CD44+) was calculated relative to baseline levels, and found to be statistically significant (*p* < 0.05) using the non-parametrical Wilcoxon signed-rank test (**A**). Representative dot plots of the flow cytometry analysis are shown (**B**). * *p* < 0.05.

To further investigate the possibility that TMPs expressing CD44 could be found in breast cancer patients following chemotherapy, we collected plasma samples from 15 breast cancer patients at baseline or 24 h after PTX chemotherapy administration. We used MUC-1 as an epithelial breast carcinogenic marker, known also to be expressed on TMPs [14]. The number of MUC-1+/CD44+ TMPs detected in plasma samples from 12 out of 15 breast cancer patients post-chemotherapy was 3–4-fold higher than those at baseline (Figure 7A,B). Taken together, the data suggest that chemotherapy induces CD44 expression on TMPs and, as a result, may contribute to tumor cell aggressiveness.

## 4. Discussion

Metastasis is the main cause of death in cancer patients and is still a major obstacle for the success of therapy. Treatment protocols involve chemotherapy, radiation, surgery, or their combination. These protocols have shown clinical benefits, yet resistance is common and recurrence following chemotherapy is usually more aggressive [46,47]. This study provides a suitable explanation for tumor cell aggressiveness in response to chemotherapy. Specifically, we demonstrate that TMPs originating from highly metastatic or chemotherapy-exposed tumor cells mediate a paracrine effect that decreases cell adhesion and rearranges the actin cytoskeleton. These effects promote early-stage metastatic properties in recipient low metastatic tumor cells (Figure 2 and Figure 3). To strengthen the fact that cell adhesion is decreased, we used the ex vivo pulmonary metastasis assay, which specifically tests the seeding properties of tumor cells, namely, their ability to adhere to the lung tissue. Although one would expect an increased number of metastatic foci in the lung sections of the PuMA, the fact that we observed a reduced number of metastasis (Figure 2A) strengthens our assumption that the major effect of TMPs is on reduced cell adhesion properties. Reduced cell adhesion is the early process required for the dissemination of tumor cells from the primary tumor site [48]. These results further demonstrate that TMPs support different stages of the metastatic cascade. Furthermore, we should note that our study focused on the in vitro changes in tumor cell characteristics in the presence of TMPs, yet there are several studies which reported that reduced adhesion of tumor cells supports early stage of metastatic properties [48]. Additional in vivo studies can strengthen these conclusions by testing whether TMPs originating from highly metastatic clones or following chemotherapy within the primary tumor microenvironment, are directly associated with increased metastasis in distant sites. Taken together, our results provide further support for the role of TMPs in promoting metastasis.

To further identify the possible mechanism for TMP-induced tumor cell metastatic properties, we have undertaken an unbiased proteomic approach to analyze the expression of various proteins associated with adhesion. We demonstrate that the pro-metastatic effects of TMPs are associated with the expression of CD44, a non-oncogenic protein (Figure 5). EVs expressing CD44 have been shown to promote tumor cell aggressiveness and metastasis mainly by contributing to the pre-metastatic niche [19,49]. Here we found that TMPs expressing CD44 contribute to metastatic tumor cell characteristics in part by disrupting the actin cytoskeleton filament structure, inhibiting focal adhesion plaques and cell spreading, reducing contractility, and downregulating adhesion-related signaling measured by phospho-paxillin (Figure 3 and Figure 4). While these effects clearly contribute to the understanding of the extracellular function of CD44, further investigation is needed regarding the intracellular domain of CD44. Specifically, it has been shown that the intracellular domain of CD44 recruits actin binding complexes such as ezrin, radixin, moesin (ERM), and ankyrin [50]. These complexes are associated with intracellular signaling via Ras-MAPK, Wnt, and PI3K [19], and can thus contribute to the metastatic characteristics of tumor cells by other mechanisms. Notably, although our studies have focused on specific adhesion molecules, based on the proteomic analysis it is plausible that other molecules expressed by TMPs may affect cell adhesion properties, including specific extracellular matrix-associated enzymes. Thus, additional studies in this direction will provide further insights into the overall effects of TMPs on tumor cell metastatic characteristics.

Elevated CD44 expression levels were also found in TMPs from breast cancer patients treated with PTX (Figure 7). When analyzing their plasma samples, we focused on MUC1+ EVs that are known to originate from tumor cells in various malignancies, suggesting that they are TMPs [14,51]. We have previously demonstrated that the number of TMPs from breast cancer patients increased following chemotherapy [14]. These results are supported by our current study, in which we demonstrate that tumor cells exposed to chemotherapy shed an increased number of TMPs (Figure 1). Clinically, we found that the percentage of TMPs expressing CD44 was increased in plasma samples from breast cancer patients following chemotherapy when compared to baseline levels. Taken together, our in vitro findings support the observations found in breast cancer patients.

In summary, we describe a mechanism whereby CD44-expressing TMPs affect tumor cell metastatic characteristics by inhibiting cell adhesion properties. This effect is augmented in response to chemotherapy, which can explain the potential increase in metastasis. Indeed, breast cancer patients treated with neoadjuvant chemotherapy may exhibit an increased tumor microenvironment of metastasis (TMEM). TMEMs promote the dissemination of tumor cells from the primary tumor site [52]. It is plausible that combining chemotherapy with agents that inhibit CD44 on TMPs may reduce the potential for metastasis, and therefore can serve as a strategy to inhibit the possible risks of chemotherapy-induced metastasis in breast cancer.

## Supplementary Materials

The following are available online at https://www.mdpi.com/2073-4409/9/10/2269/s1, Figures S1–S5, Table S1.
