# Supplementary material for: Breast Cancer-Derived Microparticles Reduce Cancer Cell Adhesion, an Effect Augmented by Chemotherapy
Source: Cells. 2020 Oct 10;9(10):2269. doi: 10.3390/cells9102269 (PMC7650796; doi:10.3390/cells9102269)
Supplement: Supplementary file 1 [file cells-09-02269-s001.zip › cells-956487-supplementary/cells-956487-supplementary Figure.pdf]

Breast cancer-derived microparticles reduce cancer cell adhesion, an effect augmented by chemotherapy

Supplemental Materials and Methods

Quantification of TMPs

TMP quantification was performed using flow cytometry by calculating the ratio between 7.35 μm counting beads (Calbiochem, San Diego, CA) and the number of events collected in the TMP gate (approximately 0.6–0.9 μm) as previously described [1, 2]. Briefly, TMPs were acquired by Cyan flow cytometry using 3 lasers- 405 nm, 488 nm and 635 nm wavelengths. The 488 nm laser was set for the scattering measurements. The voltage for forward scatter was set to 500-600 V and the side scatter to 400-500 V. The forward and side scatter axes were on logarithmic scale. The minimal threshold was set. We used 0.78 μm beads for gating on TMPs size. The pellet of TMPs was resuspended in 300 μL PBS, and 10 μL sample was added to 200 μL PBS along with 30 μL of 7.34 μm beads. The number of ECVs was calculated using the following equation: $Number\ of\ TMPs / \frac{Number\ of\ beads\ counted}{Number\ of\ total\ beads}$ /10= TMPs concentration. All in vitro and in vivo experiments involving TMPs count was based on the flow cytometry analysis.

Quantification and measurement of TMPs by Nanosight NS300 (NonoSigh Ltd., Malvern,UK) was performed as previously described [3]. All TMPs pellets were diluted in PBS to a final volume of 1 mL in order to reach ideal particle presentation in a frame (20–100 particles/frame). The following settings were used according to the manufacturer's software manual (NanoSight NS300 UserManual, MAN0541-01-EN-00, 2017, Malvern instruments, Ltd. Malvern, UK). Camera level was set until all TMPs were visible avoiding oversaturation of more than 20% (all TMPs were set to camera level 12). Autofocus was adjusted to avoid indistinct TMPs. The detection threshold was set to include as many particles as possible. For each TMP measurement, three videos were captured. Capture settings were used as follows: Camera: sCMOS; Laser: 45 mW at 488 nm; Cell temperature: 26.3 °C; Viscosity: (Water) 0.9 cP; Syringe speed: 20 μL/s; Camera Level: 12; Slider Shutter: 1200; Slider Gain: 146; FPS 18.7; Number of Frames: 1124. Following image capture, the videos were analyzed by the NanoSight Software NTA 3.2 Dev Build 3.2.16 with a detection threshold of 5. The number of completed tracks in NTA measurements was always greater than the proposed minimum of 1000 in order to minimize data skewing based on single large particles. All experiments were carried out in at least triplicates.

Modified Boyden Chamber Assay

The invasion properties of MDA-MB-231 or 67NR cells pre-exposed to different TMP conditions were evaluated in Matrigel coated Boyden chambers as previously described [4]. Briefly, serum-starved MDA-MB-231 cells ($2x10^5$ cells in 200 μL medium) were exposed for 24 h to 100,000 TMPs obtained from untreated or PTX-treated MDA-MB-231 cells, or from untreated or PTX-treated LM2-4 cells. In some experiments, TMPs were cultured with MDA-MB-231 cells for 24 h in serum-free medium in the presence of anti-CD44 antibodies (1 μg/mL). The cells were rigorously washed in PBS (100-fold volume), and then seeded on Matrigel coated 8 μm filters (BD Biosciences, San Jose, CA). The lower compartment was filled with DMEM supplemented with 5% FCS. After 6 h, cells that migrated to the bottom filter were stained with 0.1% Crystal violet and counted under an inverted microscope (Leica DMIL LED) per x100 objective-field. Of note, controls consisting samples without TMPs and with antibody-treated cells following rigorous washing as well as samples without TMPs with antibody-treated cells without washing were used to ensure

the removal of excess antibody from the samples (data not shown). All experiments were performed in triplicate.

Cell Viability AlamarBlue™ Assay

The metabolic indicator dye AlamarBlue™ (Serotec Ltd., Oxford, UK) was used to determine cell viability, as previously described [5]. Briefly, MDA-MB-231 cells that had been pre-incubated with 100,000 TMPs were seeded into a 96-well plate (500-1000 cells/well) in their designated medium supplemented with 10% AlamarBlue (AB) solution. Absorbance was acquired daily with an ELISA reader (TECAN infinite M200Pro, Switzerland). The percentage of AlamarBlue reduction was calculated using an appropriate equation, as instructed by the manufacturer. Results were corrected to background values of negative controls. All experiments were performed in triplicate.

Ex vivo pulmonary metastasis assay

Ex-vivo pulmonary metastasis assay (PuMA) was performed as previously described [6, 7]. Briefly, MDA-MB-231-GFP+ cells were cultured with 100,000 TMPs for 24h. Subsequently, the cells were washed extensively in PBS and then injected into the tail vein of 8-week old SCID mice ($2.5x10^5$ cells per mouse). Twenty minutes later, mice were anesthetized, and lungs were perfused with PBS. Next, the trachea was cannulated with a 21G intravenous catheter and attached to a gravity perfusion apparatus. The lungs were filled with heated agarose medium solution containing M-199 media, 7.5% sodium bicarbonate, 0.2 µg/mL hydrocortisone, 2.0 µg/mL bovine insulin, 200 U/mL penicillin/streptomycin and 1% agarose at a ratio of 1:1, agarose to medium (*w*/*v*). The lungs were removed and placed in cold PBS. Transverse 1-2mm thick serial sections were sliced with a scalpel. Each section was incubated on Matrigel covered plates for 7 days at 37 °C in 5% RPMI. The lung slices were analyzed for GFP+ cells using Olympus MVX109 fluorescence stereo microscope. In addition, a portion of the lungs was prepared as a single cell suspension and the number of GFP+ cells was quantified by flow cytometry, as previously described [8].

References

1. Fremder, E.; Munster, M.; Aharon, A.; Miller, V.; Gingis-Velitski, S.; Voloshin, T.; Alishekevitz, D.; Bril, R.; Scherer, S. J.; Loven, D.; et al. Tumor-derived microparticles induce bone marrow-derived cell mobilization and tumor homing: a process regulated by osteopontin. *International journal of cancer. Journal international du cancer* **2014,** *135*, 270–281.
2. Timaner, M.; Kotsofruk, R.; Raviv, Z.; Magidey, K.; Shechter, D.; Kan, T.; Nevelsky, A.; Daniel, S.; de Vries, E. G. E.; Zhang, T.; et al. Microparticles from tumors exposed to radiation promote immune evasion in part by PD-L1. *Oncogene* **2019**, *39*, 187–203.
3. Bachurski, D.; Schuldner, M.; Nguyen, P. H.; Malz, A.; Reiners, K. S.; Grenzi, P. C.; Babatz, F.; Schauss, A. C.; Hansen, H. P.; Hallek, M.; et al. Extracellular vesicle measurements with nanoparticle tracking analysis - An accuracy and repeatability comparison between NanoSight NS300 and ZetaView. *J Extracell Vesicles* **2019,** *8*, 1596016.
4. Gingis-Velitski, S.; Loven, D.; Benayoun, L.; Munster, M.; Bril, R.; Voloshin, T.; Alishekevitz, D.; Bertolini, F.; Shaked, Y. Host response to short-term, single-agent chemotherapy induces matrix metalloproteinase-9 expression and accelerates metastasis in mice. *Cancer Res* **2011,** *71*, 6986–6996.
5. Voloshin, T.; Gingis-Velitski, S.; Bril, R.; Benayoun, L.; Munster, M.; Milsom, C.; Man, S.; Kerbel, R. S.; Shaked, Y. G-CSF supplementation with chemotherapy can promote revascularization and subsequent tumor regrowth: prevention by a CXCR4 antagonist. *Blood* **2011,** *118*, 3426–3435.

6. Mendoza, A.; Hong, S. H.; Osborne, T.; Khan, M. A.; Campbell, K.; Briggs, J.; Eleswarapu, A.; Buquo, L.; Ren, L.; Hewitt, S. M.; et al. Modeling metastasis biology and therapy in real time in the mouse lung. *J Clin Invest* **2010,** *120*, 2979–2988.
7. Rachman-Tzemah, C.; Zaffryar-Eilot, S.; Grossman, M.; Ribero, D.; Timaner, M.; Maki, J. M.; Myllyharju, J.; Bertolini, F.; Hershkovitz, D.; Sagi, I.; et al. Blocking Surgically Induced Lysyl Oxidase Activity Reduces the Risk of Lung Metastases. *Cell reports* **2017,** *19*, 774–784.
8. Timaner, M.; Beyar-Katz, O.; Shaked, Y. Analysis of the Stromal Cellular Components of the Solid Tumor Microenvironment Using Flow Cytometry. *Curr Protoc Cell Biol* **2016,** *70*, 19 18 1–19 18 12.

Supplemental Figures

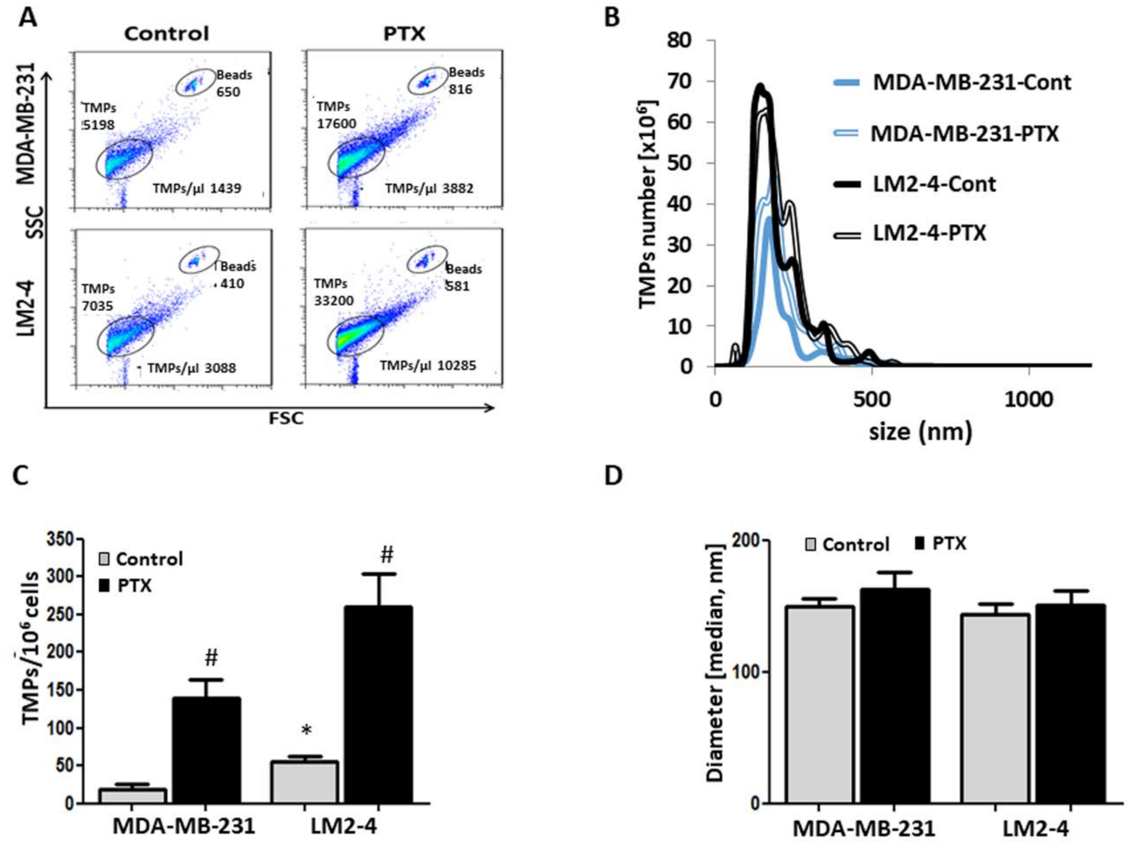

Figure 1. The extraction of TMP from high and low metastatic cells. MDA-MB-231 or LM2-4 cells were cultured for 48 h in the presence of 200 nM paclitaxel (PTX) or vehicle control (*n* = 3 biological repeats/group). (**A**–**E**) TMPs were collected from the cultures and quantified by flow cytometry (**A**, **C**) and NanoSight analysis to determine their size (**B**, **D**). * Differences from control group - * $p < 0.05$; # differences between control and PTX of the same group - # $p < 0.01$, as assessed by One Way ANOVA followed by Tukey post-hoc test.

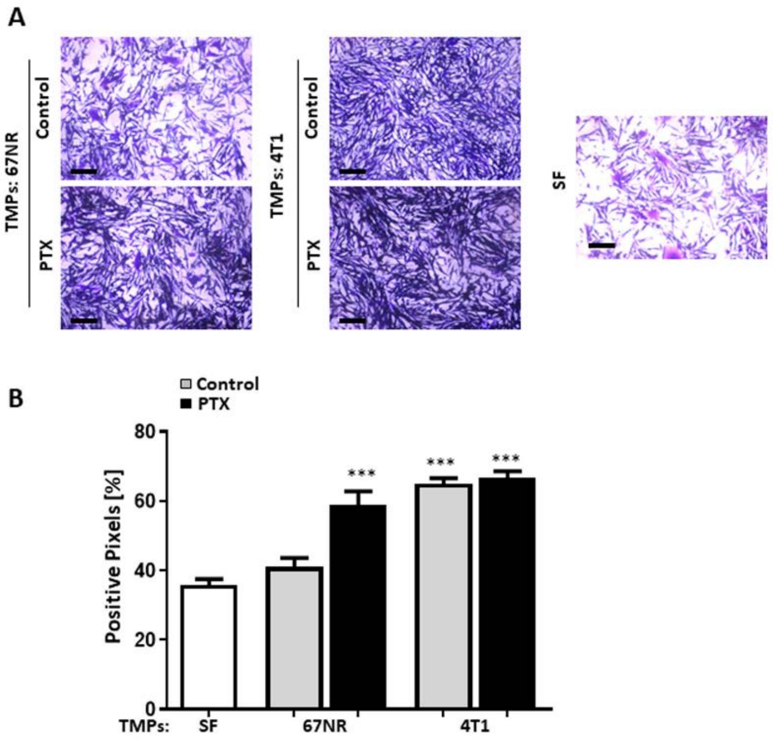

Figure S2. TMPs from highly metastatic cells or cells exposed to chemotherapy increase cell invasion. (**A**–**B**) 67NR cells cultured with serum-free or TMPs from 67NR or 4T1 cells exposed to PTX or vehicle control were assessed for invasion properties using the Boyden chamber assay. Representative images of invading cells are shown in (**A**). Scale bar 200 µm. Quantifications of invading cells are shown in (**B**) ($n = 5$ repeats and 7 images/repeat). *, differences compared to 67NR control. *** $p < 0.001$, as assessed by One Way ANOVA followed by Tukey post-hoc test.

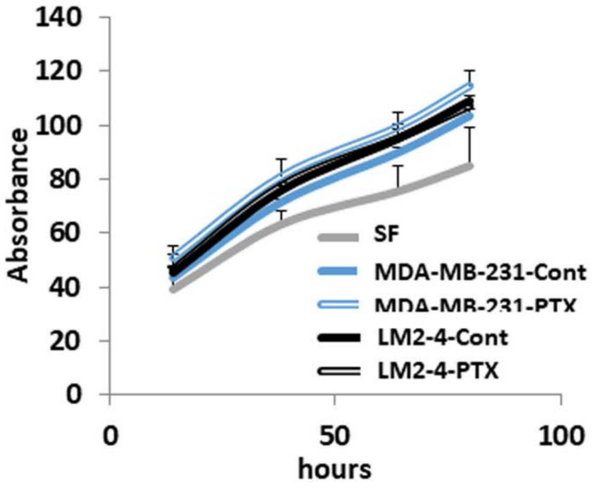

Figure S3. TMPs do not affect cell proliferation. TMPs from MDA-MB-231 or LM2-4 cells exposed to paclitaxel or vehicle control were cultured with MDA-MB-231 cells for the evaluation of cell viability by AlamarBlue assay. As a control, MDA-MB-231 cells were cultured alone in serum-free medium (SF). $n = 5$ repeats/group.

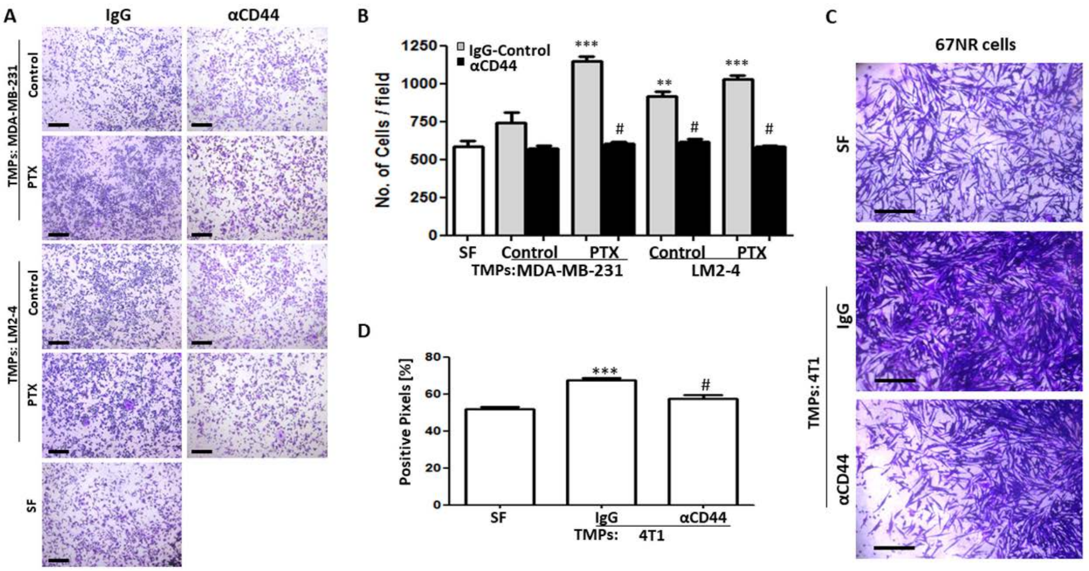

Figure S4. TMPs expressing CD44 decrease tumor cell invasion. (**A**–**B**) TMPs from MDA-MB-231 or LM2-4 cells which had been exposed to paclitaxel or vehicle control were incubated with anti-CD44 (5 μg/mL) or control IgG and subsequently cultured with MDA-MB-231 cells to assess cell invasion by Boyden chamber. As a control, MDA-MB-231 cells were cultured alone in serum-free medium (SF). Representative images of invading cells are shown in (**A**). Scale bar 200 μm. Quantification of invading cells is shown in (B). n = 5 repeats/group and 7 fields/repeat. (**C**–**D**) TMPs collected from 4T1 cells were incubated with anti-CD44 (5μg/mL) or control IgG and subsequently were cultured with 67NR cells to assess invasion properties by Boyden chamber assay. As a control, 67NR cells were cultured alone in serum-free medium (SF). Representative images of invading cells are shown (C). Scale bar 200 μm. The quantification of cell coverage is presented (D). * differences between TMPs treated cells and SF. n = 5 repeats/group and 5 frame/repeat. ** $p < 0.01$; ***, $p < 0.001$, # differences between IgG and anti-CD44, # $p < 0.05$, as assessed by One Way ANOVA followed by Tukey post-hoc test.

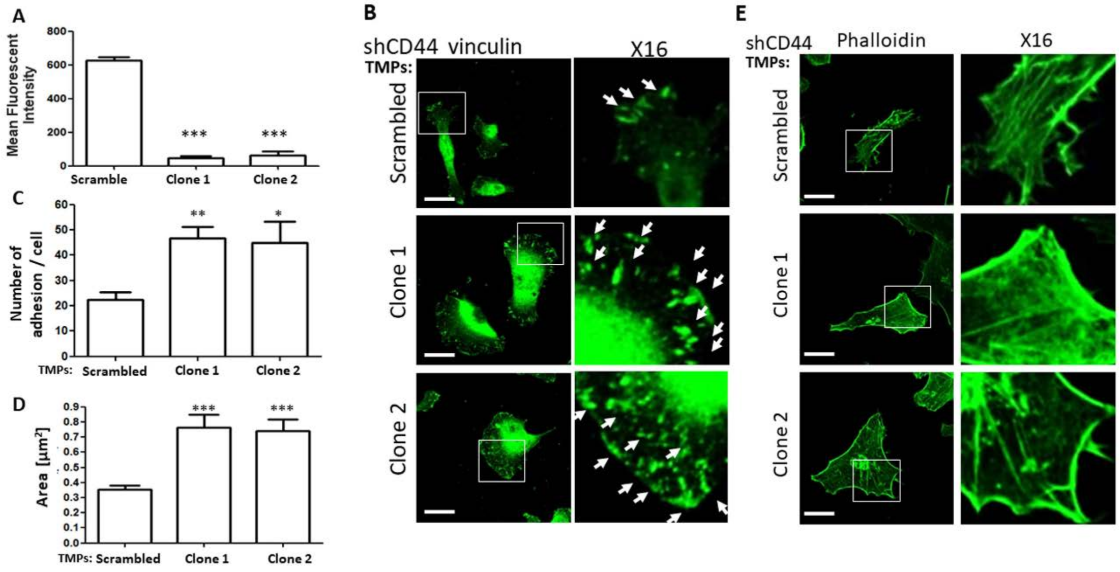

Figure S5. CD44-depleted TMPs do not induce metastatic properties in recipient cells. (A) The expression levels of CD44 in LM2-4 clones stably expressing CD44 shRNA (clone 1 and 2) or a scrambled control sequence were assessed by flow cytometry. (B-D) TMPs harvested from LM2-4 clones stably expressing CD44 shRNA (clone 1 and 2) or a scrambled control sequence, were cultured with MDA-MB-231 cells to assess focal adhesion plaques by vinculin immunostaining (green). Representative images are shown (left panel) and x16 zoom micrographs (right panel). White arrows point at focal adhesion plaques. Scale bar 20 μm (B). The number of focal adhesions per cell was quantified (C). Cell area was calculated (D). n=10-11 fields/group. (E) The cells as in B-D were stained with phalloidin (green) to assess actin filament structure. Representative images are shown (left panel) and x16 zoom micrographs (right panel). Scale bar 20 μm. n=5 fields/group. *differences compared to scrambled, *, $p<0.05$; **, $p<0.01$; ***, $p<0.001$ as assessed by One Way ANOVA followed by Tukey post-hoc test.
