# Supplementary material for: Breast Cancer-Derived Microparticles Reduce Cancer Cell Adhesion, an Effect Augmented by Chemotherapy
Source: Cells. 2020 Oct 10;9(10):2269. doi: 10.3390/cells9102269 (PMC7650796; doi:10.3390/cells9102269)
Supplement: Supplementary file 1 [file cells-09-02269-s001.zip › cells-956487-supplementary/cells-956487-supplementary Table.pdf]

Supplemental Table 1: high-resolution mass-spectrometry-based proteomic analysis of TMPs

LFQ intensity LM1_1	LFQ intensity LM_2	LFQ intensity LM_3	LFQ intensity LM1_1_ptx	LFQ intensity LM_2_ptx	LFQ intensity LM_3_ptx	LFQ intensity MDA_1	LFQ intensity MDA_2	LFQ intensity MDA_3	LFQ intensity MDA_1_ptx	LFQ intensity MDA_2_ptx	LFQ intensity MDA_3_ptx	T: Protein IDs	T: Majority protein IDs	T: Protein names	T: Gene names
NaN	NaN	NaN	NaN	NaN	NaN	25.4332	NaN	NaN	NaN	NaN	NaN	P01023	P01023	Alpha-2-macroglo	A2M
25.1873	24.5558	24.9442	25.1605	24.6585	24.9514	24.5952	NaN	24.4933	25.303	24.6894	25.3033	Q86V21;Q86V21-	Q86V21;Q86V21-	Acetoacetyl-CoA s	AACS
NaN	NaN	NaN	21.7466	NaN	NaN	NaN	NaN	NaN	NaN	NaN	NaN	Q7RTV5;H0Y5J5	Q7RTV5;H0Y5J5	Thioredoxin-like p	AAED1
NaN	NaN	NaN	21.8163	NaN	NaN	NaN	NaN	NaN	NaN	NaN	NaN	Q6PD74	Q6PD74	Alpha- and gamm	AAGAB
NaN	NaN	22.4893	NaN	23.2936	NaN	NaN	NaN	23.4554	NaN	NaN	NaN	Q6ZSR9;A0A096LI	Q6ZSR9;A0A096LI	Uncharacterized p	AAK1
24.3818	24.5276	24.5339	24.387	24.6533	24.5541	24.546	24.6077	24.86	24.1256	24.0593	24.9778	Q2M2I8;E9PG46;(	Q2M2I8;E9PG46;(	AP2-associated pr	AAK1
NaN	NaN	NaN	NaN	NaN	NaN	NaN	NaN	NaN	21.8094	22.1069	NaN	Q9H7C9;E9PNP3;I	Q9H7C9;E9PNP3;I	Mth938 domain-c	AAMDC
25.8845	24.5247	25.2159	25.7264	24.2585	25.1661	25.0199	25.1888	24.2616	25.2391	25.0416	24.5497	Q13685;C9JG97;C	Q13685;C9JG97;C	Angio-associated	AAMP
25.4032	25.2019	25.2531	25.4626	25.4835	25.2537	25.1019	25.1582	25.1826	25.4722	25.9075	25.6071	Q9Y312;A2A2Q9	Q9Y312;A2A2Q9	Protein AAR2 hon	AAR2
29.6145	29.3989	29.3122	29.3291	29.1553	29.1445	29.7959	29.7463	29.4042	29.8257	29.7665	29.5165	P49588;P49588-2	P49588;P49588-2	Alanine--tRNA liga	AARS
21.4686	NaN	21.3273	21.8845	NaN	NaN	NaN	NaN	NaN	20.7888	NaN	NaN	Q5JTZ9	Q5JTZ9	Alanine--tRNA liga	AARS2
26.3145	25.9423	25.9006	26.492	26.3259	25.9017	25.3928	24.3869	25.2625	26.2773	26.1741	24.5623	Q9BTE6-2;C9J5N1	Q9BTE6-2;C9J5N1	Alanyl-tRNA editir	AARSD1;PTGES3L-
26.8333	26.812	26.727	27.021	26.6895	26.9896	26.2565	26.3346	26.7572	27.1492	26.966	26.5235	Q9NRN7;E9PNF3;	Q9NRN7;E9PNF3;	L-aminoadipate-se	AASDHPPT
26.5272	25.9803	25.8755	26.6487	25.9748	26.384	NaN	NaN	NaN	NaN	NaN	NaN	Q9UDR5;F8WAH1	Q9UDR5;F8WAH1	Alpha-aminoadipi	AASS
23.073	NaN	NaN	23.2061	NaN	NaN	NaN	NaN	NaN	NaN	22.5641	NaN	Q9NY61;A0A087V	Q9NY61;A0A087V	Protein AATF	AATF
NaN	24.3547	NaN	NaN	NaN	NaN	NaN	NaN	NaN	NaN	NaN	NaN	Q86UQ4;A0A0A0I	Q86UQ4;A0A0A0I	ATP-binding casse	ABCA13
25.0909	23.9615	24.8959	24.6726	NaN	24.8	23.0602	NaN	NaN	23.4361	22.9478	NaN	Q9NP58;Q9NP58-	Q9NP58;Q9NP58-	ATP-binding casse	ABCB6
28.6869	28.0898	29.2577	29.1711	28.5897	29.4487	28.3769	27.8438	28.4793	29.6262	28.9515	29.195	P33527;P33527-3	P33527;P33527-3	Multidrug resistar	ABCC1
26.9773	25.689	27.1545	27.3753	26.5003	27.2142	25.8415	24.6289	25.6528	27.2218	26.3979	26.7768	O15439;O15439-2	O15439;O15439-2	Multidrug resistar	ABCC4
23.1769	23.1096	23.973	23.2744	23.5537	23.8145	NaN	NaN	NaN	24.0938	23.7285	24.1948	O15440;O15440-5	O15440;O15440-5	Multidrug resistar	ABCC5
25.7378	24.6922	25.6878	25.9509	24.5705	25.5068	25.858	NaN	25.3384	25.754	24.7486	24.5437	P28288;P28288-2	P28288;P28288-2	ATP-binding casse	ABCD3
22.9511	NaN	23.2557	23.0804	22.8321	NaN	NaN	NaN	23.9669	23.1668	23.4809	22.4773	O14678;H0YJ82;H	O14678	ATP-binding casse	ABCD4
29.0314	28.3528	28.4571	28.6832	28.4659	28.5349	28.3386	28.4361	28.3632	29.6779	29.9297	29.0704	P61221;D6R9I9;D	P61221;D6R9I9	ATP-binding casse	ABCE1
29.6729	28.5521	29.1268	29.5176	28.7149	28.8313	28.327	28.5272	28.312	28.8431	28.97	28.1535	Q8NE71;Q8NE71-	Q8NE71;Q8NE71-	ATP-binding casse	ABCF1
28.3345	27.4985	27.9769	28.3702	27.7755	27.9251	27.0605	27.967	27.6282	27.6542	27.9365	27.7066	Q9UG63;Q9UG63	Q9UG63;Q9UG63	ATP-binding casse	ABCF2
26.8013	26.0287	26.0325	26.747	25.9743	25.9636	24.6524	25.3813	NaN	24.8176	25.2682	24.7718	Q9NUQ8;Q9NUQ	Q9NUQ8;Q9NUQ	ATP-binding casse	ABCF3
24.5607	24.6809	25.2237	24.9686	25.1104	25.4843	25.0242	NaN	24.4308	25.1695	24.6215	24.6518	Q9UNQ0;Q9UNQ	Q9UNQ0;Q9UNQ	ATP-binding casse	ABCG2
24.1086	23.5393	23.376	23.4471	23.1292	22.9394	22.7698	NaN	23.5012	23.3561	23.5708	22.7724	Q9NUJ1;Q9NUJ1-	Q9NUJ1;Q9NUJ1-	Mycophenolic aci	ABHD10
21.242	22.2431	NaN	21.5463	NaN	NaN	NaN	NaN	NaN	NaN	NaN	NaN	Q8NFV4;Q8NFV4-	Q8NFV4;Q8NFV4-	Alpha/beta hydro	ABHD11
22.7838	23.0903	22.6532	23.3035	NaN	22.7966	24.1119	NaN	24.176	23.1343	NaN	22.844	Q8N2K0;Q8N2K0-	Q8N2K0;Q8N2K0-	Monoacylglycerol	ABHD12
26.1901	26.4545	26.2437	25.9919	26.5183	26.4001	26.0538	26.1367	26.4353	26.2784	26.4979	26.3619	Q96IU4;F8W9U3;	Q96IU4;F8W9U3;	Alpha/beta hydro	ABHD14B
22.8152	NaN	22.8566	23.1571	22.8168	23.1919	NaN	NaN	NaN	23.2173	22.7143	NaN	O95870;A0A0G2J	O95870;A0A0G2J	Abhydrolase dom	ABHD16A
24.6537	23.8547	24.428	24.4847	23.6853	24.2809	24.3274	NaN	NaN	24.7866	23.7565	23.8844	Q5VST6;Q5VST6-2	Q5VST6;Q5VST6-2	Alpha/beta hydro	ABHD17B
NaN	NaN	NaN	NaN	NaN	NaN	NaN	NaN	NaN	21.3573	NaN	NaN	Q8WTS1;H7BZY9	Q8WTS1;H7BZY9	1-acylglycerol-3-p	ABHD5
26.599	27.1	26.8099	26.1575	26.9546	26.8072	26.8221	26.3555	27.2056	26.8748	27.1737	27.2097	Q8IZP0-2;A0A0A0	Q8IZP0-2;A0A0A0	Abl interactor 1	ABI1
24.5072	24.5556	24.2305	24.1724	24.3253	24.1175	24.3329	NaN	NaN	NaN	24.4266	24.0621	Q9NYB9-2;Q9NYB	Q9NYB9-2;Q9NYB	Abl interactor 2	ABI2
NaN	NaN	NaN	NaN	22.1103	NaN	NaN	NaN	NaN	NaN	NaN	NaN	P00519;P42684;P	P00519;P42684;P	Tyrosine-protein k	ABL1;ABL2
24.2222	23.8234	24.59	24.1336	23.9518	24.6165	23.7423	NaN	NaN	23.4529	23.7978	23.6546	Q12979;Q12979-2	Q12979;Q12979-2	Active breakpoint	ABR
NaN	23.0279	NaN	24.2387	22.8103	NaN	NaN	NaN	NaN	24.3731	23.0859	NaN	Q9P1F3;Q5SZC9	Q9P1F3;Q5SZC9	Costars family pro	ABRACL
23.9131	23.3229	23.3081	24.4736	23.5471	NaN	NaN	NaN	NaN	23.0624	23.3399	NaN	Q9ULW3	Q9ULW3	Activator of basal	ABT1
23.3206	23.5744	23.3784	23.0413	23.5635	23.3545	23.9597	NaN	23.7238	23.852	23.5773	NaN	P09110;H7C131;C	P09110;H7C131;C	3-ketoacyl-CoA th	ACAA1
22.8582	24.3602	23.7948	22.4052	24.6648	23.7038	NaN	24.3067	25.3154	22.7945	24.4255	24.4406	P42765;A0A0B4J2	P42765;A0A0B4J2	3-ketoacyl-CoA th	ACAA2
28.1351	26.7031	27.4011	28.1796	27.3359	27.4934	27.4257	27.843	27.0587	27.885	27.6437	27.0686	Q13085;Q13085-4	Q13085;Q13085-4	Acetyl-CoA carbox	ACACA
22.8841	NaN	23.3066	23.4857	23.4816	NaN	NaN	NaN	NaN	NaN	NaN	NaN	Q9UKU7;Q9UKU7	Q9UKU7;Q9UKU7	Isobutyryl-CoA de	ACAD8
23.2192	23.6052	23.1868	23.8252	23.6693	23.3066	NaN	NaN	23.4456	22.9147	23.4449	NaN	Q9H845;H0Y8Z9;D	Q9H845;H0Y8Z9	Acyl-CoA dehydro	ACAD9
27.3792	27.4034	27.2356	27.4953	27.5559	27.1401	25.3354	26.8547	27.1753	26.7696	26.9804	26.5279	P11310;Q5T4U5;F	P11310;Q5T4U5;F	Medium-chain spe	ACADM
23.1259	23.2946	23.4566	23.7207	23.6744	23.7046	NaN	NaN	NaN	23.3164	24.0459	23.861	P16219;E9PE82	P16219;E9PE82	Short-chain specif	ACADS
23.9704	NaN	23.9804	24.0483	NaN	24.1021	NaN	NaN	NaN	23.9399	24.0349	NaN	P45954;P45954-2	P45954;P45954-2	Short/branched cl	ACADSB
28.6136	28.3211	28.3909	28.5613	28.8067	28.4749	26.9298	27.7008	28.5431	28.7255	29.1036	28.5256	P49748;P49748-2	P49748;P49748-2	Very long-chain sp	ACADVL
23.524	23.1312	23.6424	23.4901	23.4363	23.7424	23.1855	NaN	NaN	NaN	NaN	NaN	Q15057;A0A0U1R	Q15057;A0A0U1R	Arf-GAP with coile	ACAP2
27.9964	28.5658	27.701	27.8193	28.3798	27.7968	27.3381	28.2007	28.5876	27.7853	28.0853	28.0865	P24752;H0YEL7;P	P24752	Acetyl-CoA acetyl	ACAT1
28.0242	28.6183	28.1228	27.6042	28.5067	27.916	29.1454	29.2435	28.6151	28.7045	28.8671	28.7712	Q9BWD1;Q9BWD	Q9BWD1;Q9BWD	Acetyl-CoA acetyl	ACAT2
24.1078	23.8134	23.6401	23.8988	23.987	23.5496	23.2886	NaN	NaN	24.1541	23.1918	22.6101	Q9H3P7	Q9H3P7	Golgi resident pro	ACBD3
NaN	22.5361	NaN	NaN	NaN	NaN	NaN	NaN	NaN	NaN	NaN	NaN	Q5T8D3;Q5T8D3-	Q5T8D3;Q5T8D3-	Acyl-CoA-binding	ACBD5
NaN	NaN	NaN	NaN	NaN	NaN	NaN	NaN	NaN	NaN	NaN	21.4006	Q8N6N7	Q8N6N7	Acyl-CoA-binding	ACBD7
25.8849	24.9423	25.7006	26.2148	25.342	25.5691	24.3172	24.0589	23.6128	25.1938	25.516	24.7455	S4R3H4;Q9UKV3;(	S4R3H4;Q9UKV3;(	Apoptotic chroma	ACIN1
31.1888	30.6325	30.7054	31.1849	31.0683	30.8163	30.8971	31.2438	30.7965	31.404	31.2215	30.8299	P53396;P53396-2	P53396;P53396-2	ATP-citrate syntha	ACLY

28.4194	28.071	28.2698	28.375	28.1391	28.4992	27.5899	27.2754	27.6572	27.506	27.5153	27.6114	P21399	P21399	Cytoplasmic aconi	ACO1
25.7805	26.9152	26.1687	25.8236	26.7231	26.1978	25.4416	26.3889	27.155	25.7498	26.2517	26.6948	Q99798;A2A274	Q99798;A2A274	Aconitate hydrata	ACO2
24.6967	25.4866	25.0935	24.6009	25.5219	25.0191	24.9938	25.3705	26.1215	24.6536	24.9406	25.4178	Q86TX2;A0A087X	Q86TX2;A0A087X	Acyl-coenzyme A	ACOT1;ACOT2
24.7605	24.7036	24.0984	24.4559	24.5231	24.0741	NaN	NaN	NaN	24.1086	23.5694	23.1152	Q9NPJ3;Q9NPJ3-2	Q9NPJ3;Q9NPJ3-2	Acyl-coenzyme A	ACOT13
28.2902	28.316	27.9124	27.7992	28.2673	27.8289	27.9066	27.7307	27.9097	28.3801	28.5874	27.6766	O00154-4;O00154	O00154-4;O00154	Cytosolic acyl coe	ACOT7
23.0264	23.2617	23.1739	22.9387	22.7741	23.0687	NaN	NaN	NaN	NaN	22.9277	NaN	O14734;E9PJN0;H	O14734;E9PJN0;H	Acyl-coenzyme A	ACOT8
29.1145	28.4963	28.7318	29.5875	28.7477	28.8856	27.0411	27.5577	28.4058	28.231	28.0433	27.6585	Q9Y305;Q9Y305-2	Q9Y305;Q9Y305-2	Acyl-coenzyme A	ACOT9
23.6422	24.212	23.4167	23.7419	23.6938	23.735	23.9977	NaN	25.1543	23.2684	22.5436	NaN	Q15067;Q15067-2	Q15067;Q15067-2	Peroxisomal acyl-	ACOX1
26.6637	26.975	26.6471	26.3656	26.7216	26.5301	26.6103	26.1901	26.7524	26.9728	27.0269	26.4086	P24666;G5E9R5;P	P24666;G5E9R5;P	Low molecular we	ACP1
24.5186	24.207	24.7391	24.4653	24.0978	24.8456	24.1306	NaN	24.7426	24.5191	24.2833	NaN	P11117;E9PP23;E	P11117;E9PP23;E	Lysosomal acid ph	ACP2
21.7754	NaN	NaN	NaN	NaN	NaN	NaN	NaN	NaN	NaN	NaN	NaN	Q96CM8;E9PF16;	Q96CM8;E9PF16;	Acyl-CoA syntheta	ACSF2
22.5991	NaN	22.5471	22.8622	22.7453	NaN	22.8228	NaN	NaN	22.7752	NaN	NaN	P33121;E7EPM6;F	P33121;E7EPM6;F	Long-chain-fatty-a	ACSL1
28.1005	27.4265	27.8161	28.2812	27.5034	27.9118	28.6369	28.4549	28.0688	28.7793	28.0921	27.5943	O95573;F5GWH2;	O95573	Long-chain-fatty-a	ACSL3
30.0349	29.6228	30.3911	30.1072	29.6214	30.3908	30.1823	30.0052	29.5831	30.8723	30.1823	29.755	O60488;O60488-2	O60488;O60488-2	Long-chain-fatty-a	ACSL4
26.8748	26.6487	26.6688	27.0514	26.8899	26.7171	27.3796	27.7821	28.0058	27.4071	26.9476	27.5341	Q9ULC5;Q9ULC5-	Q9ULC5;Q9ULC5-	Long-chain-fatty-a	ACSL5
25.7243	25.3979	25.5881	25.5185	25.2175	25.71	26.1184	NaN	25.7974	26.4681	26.0451	25.908	Q9NR19;Q9NR19-	Q9NR19;Q9NR19-	Acetyl-coenzyme	ACSS2
22.7401	NaN	21.1689	22.4923	NaN	NaN	NaN	NaN	NaN	22.9982	22.7334	NaN	Q9H6R3;A0A0B4J	Q9H6R3;A0A0B4J	Acyl-CoA syntheta	ACSS3
NaN	NaN	NaN	27.8683	NaN	NaN	NaN	NaN	NaN	NaN	NaN	NaN	P60709;E7EVS6;G	P60709	Actin, cytoplasmic	ACTB
24.171	26.3226	26.8231	25.235	26.9499	26.469	25.3383	26.2446	25.6147	26.885	26.7478	26.5093	Q562R1	Q562R1	Beta-actin-like pr	ACTBL2
29.7581	31.2681	32.5168	31.9341	31.1621	32.5352	31.8116	31.0679	30.5386	32.5903	32.3599	31.1195	P68032;P68133;P	P68032;P68133;P	Actin, alpha cardi	ACTC1;ACTA1;ACT
36.4639	36.5603	36.8542	36.5344	36.7482	37.1067	36.5224	36.3579	36.2928	37.0264	37.163	36.6594	P63261;I3L3I0;I3L	P63261;I3L3I0;I3L	Actin, cytoplasmic	ACTG1
27.2906	27.2228	26.8905	26.9014	26.9093	27.0349	26.4228	26.5592	25.9151	27.3145	27.5223	26.5088	O96019;O96019-2	O96019;O96019-2	Actin-like protein	ACTL6A
32.5413	32.0145	32.6936	32.692	32.054	32.7859	32.6097	31.6103	31.1849	33.1418	32.5267	31.9418	P12814;P12814-2	P12814;P12814-2	Alpha-actinin-1	ACTN1
NaN	NaN	NaN	22.4918	NaN	NaN	NaN	NaN	NaN	NaN	NaN	NaN	K7EJH8	K7EJH8		ACTN4
32.8682	32.3294	32.9608	32.9822	32.0951	33.0618	33.1895	31.7603	31.408	34.0577	33.1407	32.2027	O43707;O43707-2	O43707;O43707-2	Alpha-actinin-4	ACTN4
24.6639	24.5834	24.6647	24.5352	24.3503	24.7479	25.3276	NaN	NaN	25.7011	25.1145	24.5548	Q9NZ32;V9GYX7;	Q9NZ32;V9GYX7;	Actin-related prot	ACTR10
29.0827	29.3965	29.3691	28.8524	29.7428	29.4341	29.405	29.8198	29.7803	29.732	29.8611	29.4894	P61163;R4GMT0	P61163;R4GMT0	Alpha-centractin	ACTR1A
26.0977	25.9972	25.8429	25.5224	26.4877	25.9981	26.3442	27.0045	26.4045	26.3571	26.7066	26.9923	P42025	P42025	Beta-centractin	ACTR1B
29.6544	29.7192	29.9375	29.6941	29.8379	30.0225	30.1794	29.9446	29.9811	30.4117	30.2442	30.0515	P61160;P61160-2	P61160;P61160-2	Actin-related prot	ACTR2
29.8244	30.1814	29.9296	29.5641	30.0706	30.0603	30.1806	30.2023	29.655	30.7015	30.5713	30.2293	P61158;B4DXW1;	P61158;B4DXW1	Actin-related prot	ACTR3
22.6423	NaN	21.7901	22.4367	NaN	22.1332	NaN	NaN	NaN	NaN	22.2368	NaN	Q9H9F9	Q9H9F9	Actin-related prot	ACTR5
NaN	NaN	NaN	20.7828	NaN	NaN	NaN	NaN	NaN	NaN	NaN	NaN	Q9GZN1;F8W057;	Q9GZN1;F8W057;	Actin-related prot	ACTR6
23.3273	23.3652	23.6349	23.2198	NaN	NaN	NaN	NaN	NaN	23.837	NaN	NaN	Q04771	Q04771	Activin receptor t	ACVR1
25.8806	26.7286	26.0045	25.5379	26.6068	26.2058	26.7242	27.1598	27.5251	26.3463	26.5929	27.3065	Q03154;Q03154-2	Q03154;Q03154-2	Aminoacylase-1	ACY1
25.2969	25.1468	24.8247	24.7595	24.1979	24.9071	25.2928	NaN	NaN	24.8982	23.7945	24.3378	P07311;G3V2U7	P07311;G3V2U7	Acylphosphatase-	ACYP1
22.5789	NaN	23.1844	22.6407	NaN	23.5424	NaN	NaN	NaN	24.2426	22.9122	NaN	P14621;U3KQL2;U	P14621;U3KQL2;U	Acylphosphatase-	ACYP2
28.118	28.0717	28.4964	27.9955	27.7557	28.5773	28.3288	28.0293	29.137	28.1459	27.8184	28.4214	O14672;O14672-2	O14672;O14672-2	Disintegrin and m	ADAM10
NaN	NaN	NaN	NaN	NaN	NaN	NaN	26.3722	NaN	28.7445	25.6349	NaN	O43184;Q5JRP2;C	O43184;Q5JRP2;C	Disintegrin and m	ADAM12
23.397	NaN	24.4937	23.9064	NaN	24.3406	24.6645	24.6631	24.6326	23.9627	23.8634	24.9859	Q13444;Q13444-8	Q13444;Q13444-8	Disintegrin and m	ADAM15
25.2575	25.6026	26.3272	25.4643	25.7756	26.3212	26.2545	26.4837	26.0406	26.9122	26.2869	26.5948	P78536;P78536-2	P78536;P78536-2	Disintegrin and m	ADAM17
26.3411	26.472	26.7111	26.2177	26.8326	26.7001	26.6706	26.1112	27.1684	26.3622	26.1571	26.5125	Q13443;F8WC54;	Q13443;F8WC54;	Disintegrin and m	ADAM9
25.7149	24.4772	25.7978	25.4885	25.005	24.7197	27.6079	27.0408	25.6556	26.1156	26.2181	25.7329	Q9UHI8;E5RJR7;E	Q9UHI8	A disintegrin and	ADAMTS1
24.5999	23.8139	24.8609	25.9406	25.2332	NaN	NaN	NaN	NaN	24.0402	24.31	24.5255	P55265;P55265-5	P55265;P55265-5	Double-stranded	ADAR
24.5892	24.7011	25.9498	24.5334	25.0884	25.4762	25.2701	NaN	NaN	25.1383	25.2765	25.023	O60266;U3KQ91;	O60266;U3KQ91;	Adenylate cyclase	ADCY3
NaN	NaN	22.6239	NaN	NaN	NaN	NaN	NaN	NaN	23.3037	22.5045	NaN	P51828;F5H699;H	P51828;F5H699;H	Adenylate cyclase	ADCY7
NaN	NaN	NaN	NaN	NaN	NaN	NaN	NaN	NaN	22.4064	NaN	NaN	O60503;I3L342;I3	O60503	Adenylate cyclase	ADCY9
24.2383	24.8768	25.4283	24.6387	24.8893	25.0444	24.8955	NaN	25.0283	24.9169	24.5061	24.5547	E7ENY0;P35611-6	E7ENY0;P35611-6	Alpha-adducin	ADD1
23.4747	24.5487	24.8347	24.0228	24.4713	24.6183	24.0608	23.9176	24.0725	23.6659	24.0566	NaN	A0A087WX08;Q9	A0A087WX08;Q9	Gamma-adducin	ADD3
27.8104	27.7696	27.5039	27.5554	27.6783	27.6359	27.526	27.4927	28.117	28.3155	28.4733	28.1282	P11766;H0YAG8;D	P11766	Alcohol dehydrog	ADH5
NaN	NaN	NaN	NaN	NaN	NaN	24.2284	NaN	NaN	NaN	NaN	NaN	P28332;Q8IUN7;D	P28332;Q8IUN7;D	Alcohol dehydrog	ADH6
24.6364	25.4806	24.9596	24.5799	25.3479	25.1871	26.3492	25.2541	26.0126	26.4672	25.3833	25.2286	Q9BV57;Q9BV57-	Q9BV57;Q9BV57-	1,2-dihydroxy-3-k	ADI1
19.8594	NaN	NaN	NaN	NaN	NaN	NaN	NaN	NaN	NaN	NaN	NaN	Q96A54;F8W782;	Q96A54;F8W782;	Adiponectin recep	ADIPOR1
28.0718	27.5843	27.2513	27.2754	27.3482	27.3217	27.1325	26.7567	26.9679	27.8806	27.8112	26.6792	P55263;P55263-3	P55263;P55263-3	Adenosine kinase	ADK
23.7649	25.6513	25.3652	23.4486	24.3623	24.2713	27.0875	28.2798	23.2908	NaN	NaN	NaN	P35318;E9PL83;E	P35318;E9PL83;E	ADM;Adrenomed	ADM
24.6816	NaN	24.1678	24.9457	24.2917	NaN	NaN	NaN	NaN	NaN	NaN	NaN	Q9H2P0;E9PQK8	Q9H2P0	Activity-depender	ADNP
23.4055	23.5066	23.5657	23.2184	23.2407	NaN	24.1108	NaN	NaN	23.9119	23.8731	23.8612	Q96SZ5	Q96SZ5	2-aminoethanethi	ADO
23.5884	23.7727	23.5608	23.6013	23.9919	NaN	NaN	NaN	24.4849	23.4603	23.869	23.9894	Q9BRR6;Q9BRR6-	Q9BRR6;Q9BRR6-	ADP-dependent g	ADPGK
23.6845	24.9977	24.5663	23.9078	24.7636	24.5262	25.0078	24.8717	24.6415	24.7903	24.3966	25.1539	Q9NX46	Q9NX46	Poly(ADP-ribose)	ADPRHL2
24.7878	NaN	25.2175	25.2744	24.3327	26.0025	24.8334	NaN	25.0199	25.7393	25.0504	24.9313	P07550	P07550	Beta-2 adrenergic	ADRB2
23.2884	23.312	23.9873	24.1567	23.3229	24.0523	NaN	NaN	NaN	23.8431	23.7961	NaN	P25098;E9PRV7;P	P25098;E9PRV7	Beta-adrenergic r	ADRBK1

25.1608	26.0611	25.9322	26.0905	26.2736	25.6104	25.9298	25.129	26.333	25.9298	26.4749	26.435	Q16186;A0A087W	Q16186	Proteasomal ubiq	ADRM1
27.8306	27.9773	28.1095	27.5886	28.2849	27.9358	27.9095	28.1938	28.1145	27.7739	28.0857	28.5726	P30566;A0A0A6Y	P30566;A0A0A6Y	Adenylosuccinate	ADSL
26.7464	27.3395	27.2783	26.5774	27.3948	27.1133	27.5825	27.8291	27.5546	27.8282	27.8887	28.0152	P30520	P30520	Adenylosuccinate	ADSS
22.7513	21.9758	22.4708	22.074	NaN	22.721	NaN	NaN	NaN	NaN	NaN	NaN	Q8N556;Q8N556-	Q8N556;Q8N556-	Actin filament-ass	AFAP1
22.7894	NaN	22.8456	22.8555	22.5874	NaN	NaN	NaN	NaN	22.8426	NaN	NaN	Q8TED9;Q8TED9-	Q8TED9;Q8TED9-	Actin filament-ass	AFAP1L1
24.3675	25.0198	24.5791	24.9314	25.0562	25.0877	24.1549	24.3389	25.2522	24.0457	NaN	24.2345	Q9Y4W6	Q9Y4W6	AFG3-like protein	AFG3L2
NaN	NaN	NaN	NaN	NaN	NaN	NaN	21.8026	NaN	NaN	NaN	NaN	P20933	P20933	N(4)-(beta-N-acet	AGA
23.7192	23.852	23.8648	23.6537	23.3144	23.7678	23.6352	NaN	23.6287	NaN	NaN	22.9842	Q96P47;Q96P47-2	Q96P47;Q96P47-2	Arf-GAP with GTP	AGAP3
NaN	23.8597	23.314	NaN	NaN	NaN	23.6558	NaN	NaN	NaN	NaN	NaN	P52594;C9J2I0;B8	P52594;C9J2I0;B8	Arf-GAP domain a	AGFG1
25.2379	25.1912	25.4217	25.0484	25.0891	25.3259	25.3519	25.7797	25.7687	24.6872	25.066	25.163	P35573;P35573-2	P35573;P35573-2	Glycogen debranc	AGL
24.1444	NaN	23.5915	23.9923	23.3685	23.6783	23.9289	NaN	NaN	23.1688	23.1287	NaN	Q9UKV8;Q9UKV8-	Q9UKV8;Q9UKV8-	Protein argonaute	AGO2
NaN	NaN	NaN	21.901	NaN	NaN	NaN	NaN	NaN	NaN	NaN	NaN	Q9NUQ2;H0YC22	Q9NUQ2;H0YC22	1-acyl-sn-glycerol-	AGPAT5
21.7449	NaN	21.8304	NaN	NaN	NaN	NaN	NaN	NaN	22.2455	22.0008	NaN	Q86UL3;E5RIA1;E	Q86UL3	Glycerol-3-phospł	AGPAT6
23.3111	NaN	23.0041	23.1768	22.8253	23.4381	23.698	23.8576	23.9266	23.5213	23.3008	23.7754	Q53EU6	Q53EU6	Glycerol-3-phospł	AGPAT9
25.4015	25.8537	26.1521	26.1137	25.8514	26.1027	25.8516	25.7246	26.254	25.5946	25.5044	25.4919	O00116;B8ZZ81	O00116	Alkyldihydroxyace	AGPS
23.4238	22.4588	22.209	23.6065	22.408	22.5126	NaN	NaN	NaN	NaN	NaN	NaN	O95994;B5MC07;	O95994;B5MC07;	Anterior gradient	AGR2
25.1619	25.5334	25.5861	24.2645	24.5695	24.1149	28.6766	27.9755	26.5375	25.5416	25.7716	26.3375	O00468-6;O00468	O00468-6;O00468	Agrin;Agrin N-terr	AGRN
NaN	NaN	NaN	21.058	NaN	NaN	NaN	NaN	NaN	NaN	NaN	NaN	Q9UPW5;J3KNS1;	Q9UPW5;J3KNS1;	Cytosolic carboxy	AGTPBP1
23.4391	23.1823	22.8817	23.4759	23.0198	22.8614	NaN	NaN	NaN	NaN	NaN	23.2874	Q6RW13;Q6RW13	Q6RW13;Q6RW13	Type-1 angiotensi	AGTRAP
30.6597	31.1646	31.0585	30.6338	31.1172	31.0715	31.1949	31.0381	30.7996	31.116	31.238	31.2321	P23526;P23526-2	P23526;P23526-2	Adenosylhomocys	AHCY
28.0327	27.4209	27.9835	27.9347	27.6716	28.2328	28.0931	27.9969	27.7786	28.9008	28.5474	28.5716	O43865;O43865-2	O43865;O43865-2	Putative adenosyl	AHCYL1
NaN	NaN	NaN	NaN	NaN	NaN	NaN	NaN	NaN	22.8986	NaN	NaN	Q96HN2;Q96HN2	Q96HN2;Q96HN2-	Putative adenosyl	AHCYL2
32.5386	32.7161	32.4727	32.1214	32.4848	32.4127	32.8235	32.3333	31.8688	32.5404	32.3305	32.6945	Q09666;E9PJZ0;E9	Q09666	Neuroblast differe	AHNAK
23.3535	23.0521	23.3196	22.3336	22.6715	22.4028	NaN	NaN	NaN	NaN	21.5893	NaN	Q8IVF2;Q8IVF2-3	Q8IVF2;Q8IVF2-3	Protein AHNAK2	AHNAK2
28.7379	28.4975	28.5216	28.4725	28.3836	28.4168	28.842	27.9681	28.0822	28.5096	28.3209	28.651	O95433;O95433-2	O95433;O95433-2	Activator of 90 kD	AHSA1
24.7514	25.4914	25.9	24.5906	25.1975	25.5766	25.0022	24.6274	NaN	25.7928	25.157	25.0493	Q96BJ3;Q96BJ3-3	Q96BJ3	Axin interactor, d	AIDA
24.563	24.9759	24.4631	24.6494	25.065	24.8085	24.044	25.2053	24.7678	24.3176	24.2713	NaN	O95831;O95831-3	O95831;O95831-3	Apoptosis-inducin	AIFM1
24.8048	23.9456	24.23	24.7376	24.0607	23.9179	24.327	NaN	NaN	24.3418	23.955	NaN	Q9BRQ8;Q9BRQ8	Q9BRQ8;Q9BRQ8-	Apoptosis-inducin	AIFM2
24.1079	23.788	23.229	23.984	24.1203	22.727	NaN	24.2035	23.636	23.7817	24.1523	24.2111	Q9Y4K1;A0A0J9Y	Q9Y4K1;A0A0J9Y	Absent in melano	AIM1
27.8803	28.2652	27.7669	28.0901	28.3745	27.7271	27.4045	27.9547	26.826	27.4419	28.0669	28.0499	Q12904;Q12904-2	Q12904;Q12904-2	Aminoacyl tRNA s	AIMP1
28.4964	27.8845	27.6912	28.1741	28.3908	27.9814	26.962	27.9957	28.3459	27.8266	28.3122	26.9473	Q13155;F8W950;	Q13155;F8W950;	Aminoacyl tRNA s	AIMP2
26.4902	26.6074	26.1713	26.0296	26.2401	26.2999	26.29	26.3161	26.0294	26.2118	26.176	26.4624	O00170;E9PMH2;	O00170;E9PMH2	AH receptor-inter	AIP
23.3094	24.3292	24.1592	23.8313	24.2023	24.2032	23.6923	23.9813	24.1736	24.0238	24.096	25.0508	Q96IF1;H0YJL9	Q96IF1	LIM domain-conta	AJUBA
25.8926	26.5939	26.0758	25.8361	26.1992	26.0827	26.4111	26.3613	26.9512	26.2655	26.0471	26.8088	P00568;Q5T9B7;H	P00568;Q5T9B7;H	Adenylate kinase	AK1
26.2224	27.5552	26.5123	25.9312	27.2479	26.4852	26.396	27.6006	28.131	26.4992	26.9901	26.6029	P54819;F8W1A4;F	P54819;F8W1A4;F	Adenylate kinase	AK2
23.6421	24.5355	24.3504	23.8357	24.756	24.1859	24.032	NaN	25.4729	23.9543	24.8078	25.3677	Q9UIJ7;Q9UIJ7-3;	Q9UIJ7;Q9UIJ7-3	GTP:AMP phosph	AK3
27.0407	26.8014	26.4768	26.8145	26.7401	26.258	25.1418	25.9842	26.7339	26.0001	26.433	26.1509	P27144;D3DQ64	P27144;D3DQ64	Adenylate kinase	AK4
25.8988	25.0178	25.1559	25.7905	25.2937	25.3968	24.7732	NaN	23.9269	25.7563	25.4625	25.978	Q9Y3D8;A0A087V	Q9Y3D8;A0A087V	Adenylate kinase	AK6;TAF9
20.0951	NaN	NaN	NaN	NaN	NaN	NaN	NaN	NaN	NaN	NaN	NaN	Q92667;I3L0W0;I3	Q92667;I3L0W0;I3	A-kinase anchor p	AKAP1
22.5232	NaN	NaN	NaN	NaN	NaN	NaN	NaN	NaN	21.9219	NaN	NaN	O43572;E7EMD6;	O43572;E7EMD6	A-kinase anchor p	AKAP10
24.1964	22.6693	23.605	NaN	22.4556	NaN	24.5617	23.3893	22.8745	22.4707	NaN	NaN	Q9UKA4	Q9UKA4	A-kinase anchor p	AKAP11
23.4721	23.5777	24.5536	23.1402	NaN	23.1054	24.5083	NaN	NaN	23.4087	NaN	NaN	Q02952;Q02952-3	Q02952;Q02952-3	A-kinase anchor p	AKAP12
21.7287	NaN	NaN	21.9294	NaN	NaN	NaN	NaN	NaN	NaN	NaN	NaN	Q02040;X6RAJ1;C	Q02040;X6RAJ1;C	A-kinase anchor p	AKAP17A
27.6903	28.1493	27.2807	26.9736	27.5455	27.0703	27.4081	27.7154	27.3395	26.5035	26.0954	26.6203	Q9Y2D5-6;Q9Y2D	Q9Y2D5-6;Q9Y2D	A-kinase anchor p	AKAP2
NaN	NaN	NaN	NaN	NaN	NaN	NaN	NaN	NaN	NaN	NaN	20.4673	Q9ULX6;Q9ULX6-	Q9ULX6;Q9ULX6-	A-kinase anchor p	AKAP8L
20.1278	NaN	NaN	NaN	NaN	NaN	NaN	NaN	NaN	NaN	NaN	NaN	Q99996;A0A087W	Q99996;A0A087W	A-kinase anchor p	AKAP9
26.438	27.1963	26.8116	26.1667	26.9355	26.8984	26.1692	26.561	26.9072	26.0945	26.2666	26.445	P14550;V9GYG2;V	P14550	Alcohol dehydrog	AKR1A1
26.724	28.0983	27.0673	26.1269	27.7847	27.2085	29.1404	28.7411	29.5807	28.3516	28.7742	29.7722	P15121;E9PCX2;E	P15121;E9PCX2	Aldose reductase	AKR1B1
24.7318	25.6972	25.6252	24.5439	25.2434	25.3331	25.8697	25.989	26.2028	25.4882	25.6049	26.1691	P42330;A0A0A0M	P42330;A0A0A0M	Aldo-keto reducta	AKR1C3;AKR1C1
27.9243	28.1923	27.779	27.5201	28.0027	27.7	27.7591	27.573	28.0044	28.3553	28.5477	28.2603	O43488;H3BLU7;H	O43488;H3BLU7	Aflatoxin B1 aldeh	AKR7A2
25.5833	24.9664	24.9013	25.3773	24.563	24.9336	24.8897	NaN	24.414	25.431	25.0895	24.9573	P31749;P31749-2	P31749;P31749-2	RAC-alpha serine/	AKT1
NaN	21.8668	22.2935	NaN	21.553	NaN	NaN	NaN	NaN	NaN	NaN	NaN	Q96B36;Q96B36-3	Q96B36;Q96B36-3	Proline-rich AKT1	AKT1S1
NaN	NaN	23.3684	NaN	NaN	NaN	23.8329	NaN	NaN	NaN	NaN	NaN	P13716;P13716-2	P13716;P13716-2	Delta-aminolevuli	ALAD
29.7651	29.2752	29.5751	29.9004	29.0341	29.6871	30.2158	30.0383	29.7723	30.431	29.8043	29.7463	Q13740;Q13740-2	Q13740;Q13740-2	CD166 antigen	ALCAM
26.3047	26.7091	26.2421	26.202	26.9536	26.4538	26.3762	26.8418	27.0303	26.3542	26.8767	27.0718	Q8IZ83;Q8IZ83-3;	Q8IZ83;Q8IZ83-3;	Aldehyde dehydro	ALDH16A1
28.3172	27.6628	27.4981	28.6999	28.1026	27.907	26.3002	27.244	27.9232	26.4643	27.1224	26.742	P54886;P54886-2	P54886;P54886-2	Delta-1-pyrroline-	ALDH18A1
24.8456	24.6719	24.6215	25.3363	25.0544	24.1707	NaN	23.7563	24.8909	23.9135	23.6433	24.2792	P30837	P30837	Aldehyde dehydro	ALDH1B1
27.1316	27.6314	27.0363	26.9986	27.8505	27.2323	24.3915	25.9497	26.6886	25.1531	26.3643	26.4078	P05091;P05091-2	P05091;P05091-2	Aldehyde dehydro	ALDH2
23.8096	23.372	24.0634	23.8106	23.8498	24.2429	NaN	NaN	NaN	24.6016	24.0464	NaN	P51648;P51648-2	P51648;P51648-2	Fatty aldehyde de	ALDH3A2
22.8496	NaN	22.2702	NaN	22.7205	NaN	NaN	NaN	NaN	23.248	NaN	NaN	P30038;P30038-2	P30038;P30038-2	Delta-1-pyrroline-	ALDH4A1

26.0209	24.9946	25.1454	25.5988	25.0651	25.4187	24.3392	23.8731	NaN	24.0917	NaN	NaN	Q02252;Q02252-2	Q02252;Q02252-2	Methylmalonate-s	ALDH6A1
29.0089	29.0455	28.8634	28.7729	29.0135	28.9501	29.1311	29.4798	29.7246	29.6189	29.9775	29.6469	P49419-2;P49419;	P49419-2;P49419;	Alpha-aminoadipi	ALDH7A1
28.7063	28.5098	28.3505	28.4237	28.4191	28.3904	28.5024	28.1633	28.2925	28.8159	29.1413	28.5319	P49189;P49189-2	P49189;P49189-2	4-trimethylamino	ALDH9A1
33.3657	34.1419	33.6562	32.9593	33.7151	33.5129	34.2118	34.226	33.5712	33.8224	33.8605	33.8915	P04075;J3KPS3;H3	P04075;J3KPS3;H3	Fructose-bisphosp	ALDOA
NaN	20.3774	NaN	NaN	NaN	NaN	NaN	NaN	NaN	NaN	NaN	NaN	P05062;A0A087W	P05062;A0A087W	Fructose-bisphosp	ALDOB
29.3685	30.1076	29.4444	28.678	29.4066	29.2312	29.6621	29.7772	29.2945	29.0377	29.1142	29.2904	P09972;A8MVZ9;J	P09972;A8MVZ9;J	Fructose-bisphosp	ALDOC
24.0444	22.8147	23.4292	23.6242	23.4392	23.5796	23.7837	24.5091	24.0057	24.3282	23.4037	23.6883	Q9BT22;K7EID2;Q	Q9BT22;K7EID2;Q	Chitobiosyldiphos	ALG1
23.1063	NaN	22.9112	23.1484	22.7284	23.0874	NaN	23.4502	NaN	23.2474	22.7766	22.9867	Q9H553;Q9H553-	Q9H553;Q9H553-	Alpha-1,3/1,6-man	ALG2
NaN	NaN	NaN	20.2219	NaN	NaN	NaN	NaN	NaN	NaN	NaN	NaN	Q92685;F8WF93;I	Q92685;F8WF93;I	Dol-P-Man:Man(5	ALG3
24.7739	24.5138	24.3556	25.0041	24.2462	24.4034	24.9514	NaN	24.4859	25.025	24.4551	24.2188	Q9Y673;Q9Y673-2	Q9Y673;Q9Y673-2	Dolichyl-phosphat	ALG5
NaN	NaN	22.089	22.5761	NaN	NaN	NaN	NaN	NaN	NaN	NaN	NaN	Q9H6U8;A0A087V	Q9H6U8;A0A087V	Alpha-1,2-mannos	ALG9
22.9022	22.6086	22.7936	23.0641	22.8136	22.7344	NaN	NaN	NaN	22.6835	22.6559	22.8604	Q6P6C2;Q6P6C2-1	Q6P6C2;Q6P6C2-1	RNA demethylase	ALKBH5
NaN	NaN	22.0009	NaN	NaN	NaN	NaN	NaN	NaN	NaN	NaN	NaN	P05186;P05186-2	P05186;P05186-2	Alkaline phosphat	ALPL
NaN	NaN	NaN	NaN	NaN	NaN	21.9616	NaN	NaN	NaN	NaN	NaN	P05187;P10696;A	P05187;P10696;A	Alkaline phosphat	ALPP;ALPPL2
26.427	26.5593	26.1983	26.051	26.4293	26.2622	25.8708	27.0495	25.646	25.8872	26.4693	26.5544	Q86V81;E9PB61	Q86V81;E9PB61	THO complex sub	ALYREF
21.0656	NaN	NaN	NaN	NaN	NaN	NaN	NaN	NaN	NaN	NaN	NaN	Q8IY45;Q8IY45-2;	Q8IY45;Q8IY45-2;	Protein AMN1 hor	AMN1
NaN	NaN	NaN	NaN	NaN	NaN	NaN	NaN	NaN	NaN	NaN	21.0417	Q8IY63;Q8IY63-2	Q8IY63;Q8IY63-2	Angiomotin-like p	AMOTL1
23.657	23.6436	23.7713	23.5074	23.4197	23.9506	NaN	NaN	NaN	23.8895	23.6367	NaN	Q9Y2J4;Q9Y2J4-2;	Q9Y2J4;Q9Y2J4-2;	Angiomotin-like p	AMOTL2
25.5456	25.6763	25.6114	25.5661	25.9347	25.5236	26.2976	26.0347	26.0428	25.9123	25.7788	25.7588	Q01433;Q01433-2	Q01433;Q01433-2	AMP deaminase 2	AMPD2
26.1143	24.7126	24.7031	25.6163	24.5003	24.4259	NaN	23.4711	NaN	24.5142	24.0323	23.4835	Q9H1A4;H0Y564;I	Q9H1A4;H0Y564	Anaphase-promot	ANAPC1
24.6449	24.703	24.2068	24.7922	24.4416	NaN	NaN	NaN	NaN	23.9865	24.0098	24.235	Q9UM13;D6R9Q5	Q9UM13;D6R9Q5	Anaphase-promot	ANAPC10
21.8906	NaN	NaN	NaN	NaN	NaN	NaN	NaN	NaN	NaN	NaN	NaN	Q96DE5;A0A087V	Q96DE5;A0A087V	Anaphase-promot	ANAPC16
23.3525	NaN	NaN	22.2672	NaN	NaN	NaN	NaN	NaN	21.9674	NaN	NaN	Q9UJX5;Q9UJX5-3	Q9UJX5;Q9UJX5-3	Anaphase-promot	ANAPC4
23.8768	22.6563	22.6773	24.0567	22.794	23.096	NaN	NaN	NaN	22.7304	22.8316	NaN	Q9UJX4;F5H0F9;C	Q9UJX4;F5H0F9;C	Anaphase-promot	ANAPC5
27.5412	26.0557	26.7864	27.7376	26.6016	26.7794	25.1146	24.7669	25.0657	26.0869	26.0857	24.5489	Q9UJX3;Q9UJX3-2	Q9UJX3;Q9UJX3-2	Anaphase-promot	ANAPC7
NaN	NaN	22.8827	NaN	NaN	NaN	NaN	NaN	NaN	23.5062	NaN	NaN	Q9UKU9;O95841;	Q9UKU9	Angiopoietin-relat	ANGPTL2
NaN	NaN	NaN	NaN	NaN	NaN	NaN	NaN	NaN	NaN	NaN	21.7134	Q12955;Q12955-4	Q12955;Q12955-4	Ankyrin-3	ANK3
26.073	25.2711	25.3497	25.8791	25.2691	24.9631	24.0552	NaN	24.0748	24.7741	24.3889	23.3846	Q9P2R3;Q9P2R3-1	Q9P2R3;Q9P2R3-1	Rabankyrin-5	ANKFY1
24.1096	22.9051	22.832	23.1472	22.6808	22.8099	NaN	NaN	NaN	NaN	NaN	NaN	Q8IWZ3;Q8IWZ3-	Q8IWZ3;Q8IWZ3-	Ankyrin repeat an	ANKHD1
23.6586	23.5429	23.3225	23.2595	23.3877	23.2795	23.4601	NaN	NaN	23.146	NaN	NaN	Q8IZ07;H0YIN8;S4	Q8IZ07;H0YIN8	Ankyrin repeat do	ANKRD13A
26.0964	22.5578	23.1402	23.7618	21.9227	21.8326	NaN	NaN	NaN	21.3032	NaN	NaN	O75179;H0YM23;	O75179;H0YM23;	Ankyrin repeat do	ANKRD17
25.8084	24.5612	25.0665	25.5265	24.926	25.0736	25.2357	25.2195	NaN	25.6186	25.1283	24.7221	O15084;O15084-1	O15084;O15084-1	Serine/threonine-	ANKRD28
22.8745	NaN	22.3152	22.9883	22.4228	NaN	NaN	NaN	23.2915	NaN	22.5247	22.8419	Q9ULJ7;Q9ULJ7-2	Q9ULJ7;Q9ULJ7-2	Ankyrin repeat do	ANKRD50
NaN	NaN	NaN	NaN	NaN	NaN	NaN	NaN	NaN	26.6355	NaN	NaN	Q92625;Q92625-2	Q92625;Q92625-2	Ankyrin repeat an	ANKS1A
26.4266	24.7804	25.8375	26.0674	24.947	25.1366	NaN	NaN	NaN	23.7012	23.6395	NaN	Q9H8Y5;B8ZZS4;C	Q9H8Y5;B8ZZS4	Ankyrin repeat an	ANKZF1
28.4732	28.653	29.7255	29.2021	29.2318	30.0621	29.68	29.0121	29.2881	29.1373	28.8854	29.1625	Q9NQW6;Q9NQW	Q9NQW6;Q9NQW	Actin-binding prot	ANLN
24.9001	23.7492	24.3899	24.6026	23.8006	24.2341	25.896	25.6283	25.1888	25.7844	NaN	NaN	Q9NW15;Q9NW1	Q9NW15;Q9NW1	Anoctamin-10;An	ANO10
26.4429	26.1798	27.1863	26.7975	26.4373	27.6034	26.9927	25.9723	26.8762	27.9775	27.272	27.382	Q4KMQ2;Q4KMQ	Q4KMQ2;Q4KMQ	Anoctamin-6	ANO6
24.7412	25.3325	25.6231	25.348	25.7924	26.1893	26.0143	26.1703	26.3274	26.1318	26.5284	26.8061	P39687;H0YN26;H	P39687;H0YN26	Acidic leucine-rich	ANP32A
25.0614	24.9219	25.3406	25.2905	24.2751	25.3835	25.1795	NaN	NaN	26.0804	25.5957	25.4305	Q92688;Q92688-2	Q92688;Q92688-2	Acidic leucine-rich	ANP32B
26.9552	25.2001	25.8928	26.6218	25.2849	26.3714	NaN	25.3072	25.0842	26.0006	25.7637	25.2194	Q9BTT0;Q9BTT0-3	Q9BTT0;Q9BTT0-3	Acidic leucine-rich	ANP32E
24.4182	24.9036	25.1292	24.8198	24.9034	25.0649	24.5117	NaN	25.0833	24.8496	24.5824	24.9774	Q9H6X2;Q9H6X2-	Q9H6X2;Q9H6X2-	Anthrax toxin rec	ANTXR1
28.5123	28.3191	28.6028	28.4546	28.1339	28.6181	28.1885	28.1823	29.2077	28.418	27.861	28.79	P58335-4;P58335	P58335-4;P58335	Anthrax toxin rec	ANTXR2
32.0876	31.6547	30.8469	32.9837	31.7539	30.6505	31.954	31.7803	31.9629	32.2478	32.1955	32.1729	P04083;Q5T3N1;(	P04083;Q5T3N1	Annexin A1;Anne	ANXA1
29.1548	29.4655	29.5348	29.3502	29.4544	29.5937	29.5229	29.0906	29.6792	29.6062	29.1748	29.6644	P50995;P50995-2	P50995;P50995-2	Annexin A11	ANXA11
35.1668	34.3964	34.4812	36.0731	34.591	34.3665	34.0831	33.8226	34.0764	34.7405	34.2826	33.8869	P07355;P07355-2	P07355;P07355-2	Annexin A2;Anne	ANXA2
29.7927	29.7127	29.1971	29.3371	29.3275	29.1226	30.5762	30.2896	30.5596	31.5298	30.5177	30.5265	P12429;D6RA82;D	P12429;D6RA82;D	Annexin A3;Anne	ANXA3
22.063	NaN	NaN	NaN	NaN	NaN	NaN	NaN	NaN	NaN	NaN	NaN	P09525;P09525-2	P09525;P09525-2	Annexin A4	ANXA4
27.957	28.2059	27.0175	28.777	28.1386	26.7792	27.677	26.0624	27.3838	27.0761	26.3732	26.858	Q6P452;B4E1S2	Q6P452	Annexin	ANXA4
30.4308	31.2204	29.5023	31.66	30.9584	29.4058	31.2901	30.4826	29.9176	30.803	30.4443	29.8345	P08758;D6RBL5;E	P08758;D6RBL5;E	Annexin A5;Anne	ANXA5
26.1674	NaN	25.5458	25.5929	25.346	25.3074	26.1288	NaN	NaN	26.3013	25.4959	NaN	E5RK69;H0YC77	E5RK69	Annexin	ANXA6
34.945	34.8992	34.8515	35.1755	34.8753	34.8953	34.8483	34.1788	34.3792	35.0396	34.3449	34.5351	P08133;P08133-2	P08133;P08133-2	Annexin A6;Anne	ANXA6
28.303	28.5266	27.9436	28.2637	28.3857	27.7516	28.7494	27.8027	28.0645	28.5192	28.0186	28.1666	P20073;P20073-2	P20073;P20073-2	Annexin A7	ANXA7
26.1508	26.5193	26.2848	25.6217	26.3561	26.1415	26.8625	26.5067	25.6118	26.129	25.9641	25.9174	Q10567-3;Q1056	Q10567-3;Q1056	AP-1 complex sub	AP1B1
27.3015	26.2058	27.1217	27.1992	26.2668	27.0823	27.3122	25.3351	26.4509	27.5347	27.1467	26.4936	O43747;O43747-2	O43747;O43747-2	AP-1 complex sub	AP1G1
25.4799	24.9721	25.1173	24.8826	24.3883	24.8819	24.7345	NaN	NaN	24.204	24.5328	23.7709	O75843;G3V532;(	O75843	AP-1 complex sub	AP1G2
26.8883	26.7138	26.9917	26.4735	26.8275	27.0349	26.9337	26.8503	26.2005	26.5019	26.765	26.5826	Q9BXS5;Q9BXS5-2	Q9BXS5;Q9BXS5-2	AP-1 complex sub	AP1M1
NaN	NaN	NaN	21.5194	NaN	NaN	NaN	NaN	NaN	NaN	NaN	NaN	P61966;H7C1E4;P	P61966;H7C1E4;P	AP-1 complex sub	AP1S1
24.1881	24.0201	24.4483	24.2962	23.7581	24.3378	24.0178	23.8364	24.2167	23.9799	23.998	NaN	P56377;F6SFB5;A	P56377;F6SFB5;A	AP-1 complex sub	AP1S2
28.0569	28.1625	28.218	27.8124	28.1634	28.2847	28.0871	27.6391	27.557	27.93	27.9335	27.7407	O95782;O95782-2	O95782;O95782-2	AP-2 complex sub	AP2A1

26.3199	25.8939	25.9671	25.9108	25.8129	25.9356	25.8874	24.8733	24.6302	25.5449	25.2399	25.4257	O94973;O94973-2	O94973;O94973-2	AP-2 complex sub	AP2A2
29.4256	29.3176	29.647	29.2525	28.985	29.5705	29.6098	28.7393	28.9312	29.4588	29.2262	29.0692	P63010;P63010-2	P63010;P63010-2,	AP-2 complex sub	AP2B1
28.2137	27.9809	28.3438	27.8934	28.3067	28.39	28.0986	27.5851	28.1402	27.4733	27.6604	28.2136	Q96CW1;Q96CW1	Q96CW1;Q96CW1	AP-2 complex sub	AP2M1
25.1129	25.2806	25.4579	25.1568	25.5914	25.5278	25.0283	24.9237	25.3441	24.549	24.9653	25.3657	P53680;M0R0N4;	P53680;M0R0N4;	AP-2 complex sub	AP2S1
27.612	26.5039	27.111	27.3407	26.8825	27.3168	26.8772	26.6634	27.0741	27.0644	27.0928	26.6922	O00203;O00203-3	O00203;O00203-3	AP-3 complex sub	AP3B1
26.5265	25.9257	26.4211	26.3931	26.1525	26.3436	25.6546	26.2343	26.2323	26.117	26.1665	26.0849	O14617;O14617-5	O14617;O14617-5	AP-3 complex sub	AP3D1
26.7773	25.5717	26.1846	26.7534	25.9837	25.9502	25.14	24.6167	25.8698	25.1039	25.0237	24.169	Q9Y2T2	Q9Y2T2	AP-3 complex sub	AP3M1
23.5386	22.7784	23.4022	23.3754	22.9983	22.9887	NaN	NaN	NaN	NaN	NaN	NaN	P53677;E5RJ52;H(	P53677;E5RJ52	AP-3 complex sub	AP3M2
25.351	24.7562	25.5119	25.6813	25.2939	25.3705	24.467	24.4567	25.0134	25.359	25.2792	24.4149	Q92572;F5H459	Q92572;F5H459	AP-3 complex sub	AP3S1
21.4844	NaN	NaN	21.0597	NaN	NaN	NaN	NaN	NaN	NaN	NaN	NaN	Q9Y6B7;B1ALD0;E	Q9Y6B7;B1ALD0;E	AP-4 complex sub	AP4B1
NaN	NaN	NaN	NaN	NaN	NaN	NaN	NaN	NaN	NaN	NaN	NaN	Q9UPM8;Q9UPM	Q9UPM8;Q9UPM	AP-4 complex sub	AP4E1
22.3427	NaN	NaN	NaN	NaN	NaN	NaN	NaN	NaN	NaN	NaN	NaN	O00189;C9JWL4;(	O00189;C9JWL4;(	AP-4 complex sub	AP4M1
20.999	NaN	NaN	NaN	NaN	NaN	NaN	NaN	NaN	NaN	NaN	NaN	O43299;O43299-2	O43299;O43299-2	AP-5 complex sub	AP5Z1
23.8333	21.9481	23.4157	24.0737	NaN	NaN	NaN	NaN	NaN	23.1573	NaN	NaN	O14727;O14727-4	O14727;O14727-4	Apoptotic proteas	APAF1
NaN	NaN	NaN	NaN	NaN	NaN	NaN	NaN	NaN	NaN	19.9852	NaN	O96018	O96018	Amyloid beta A4 p	APBA3
22.7547	NaN	22.0986	21.8993	NaN	NaN	NaN	NaN	NaN	NaN	NaN	21.1932	Q7Z5R6	Q7Z5R6	Amyloid beta A4 p	APBB1IP
26.8822	27.8095	27.2376	26.7798	27.7054	27.2681	27.9796	28.7758	28.3176	27.6214	28.0666	28.3697	P13798;C9JIF9;H7	P13798;C9JIF9;H7	Acylamino-acid-re	APEH
26.4944	26.1894	26.3221	26.4223	26.6759	26.1835	25.368	25.4166	NaN	26.4403	26.6266	26.2241	P27695;G3V3M6;	P27695;G3V3M6;	DNA-(apurinic or a	APEX1
NaN	NaN	NaN	NaN	NaN	NaN	NaN	NaN	NaN	21.9166	NaN	NaN	Q96BI3;Q96BI3-2;	Q96BI3;Q96BI3-2	Gamma-secretase	APH1A
24.7992	24.3107	24.5483	24.8428	24.0769	24.5645	24.6423	24.3292	NaN	24.6086	24.8342	24.661	Q9BZZ5-2;Q9BZZ5	Q9BZZ5-2;Q9BZZ5	Apoptosis inhibitc	API5
23.5032	24.3317	23.9522	23.3724	24.082	23.8737	NaN	NaN	NaN	23.4736	NaN	23.996	Q96GX9;S4R3D6;(	Q96GX9;S4R3D6;(	Methylthioribulos	APIP
28.479	28.7839	28.7286	28.0322	28.0361	28.3588	30.5611	29.5919	29.2894	27.8911	27.5525	28.2019	Q06481;Q06481-2	Q06481;Q06481-2	Amyloid-like prote	APLP2
27.4646	27.4319	27.1989	27.5305	27.3883	27.2331	27.8374	27.6223	28.1818	27.6077	27.4036	27.4419	Q9HDC9;H0Y512;	Q9HDC9;H0Y512;	Adipocyte plasma	APMAP
23.8383	24.7644	24.3159	23.5046	24.3529	24.0939	24.1893	NaN	NaN	23.7525	24.0044	NaN	Q8NCW5;Q8NCW	Q8NCW5;Q8NCW	NAD(P)H-hydrate	APOA1BP
25.2004	25.6905	24.0427	25.925	24.7451	23.9519	NaN	NaN	25.6802	24.0121	25.0937	24.7677	P04114;A0A087W	P04114;A0A087W	Apolipoprotein B-	APOB
26.3041	25.1843	25.6685	26.7589	25.6124	25.8443	NaN	NaN	24.3027	25.1442	25.6744	24.6132	Q9UH17;B0QYD3;	Q9UH17;B0QYD3;	DNA dC->dU-editi	APOBEC3B
23.4685	23.8006	NaN	24.0087	24.0812	24.3277	NaN	NaN	23.5413	23.7112	23.8238	24.1641	Q9NRW3	Q9NRW3	DNA dC->dU-editi	APOBEC3C
23.8925	24.5636	24.1848	24.593	24.3277	24.967	NaN	NaN	NaN	24.0195	NaN	24.1462	Q96AK3;Q6ICH2;/	Q96AK3;Q6ICH2	DNA dC->dU-editi	APOBEC3D;bK150(
NaN	NaN	NaN	NaN	NaN	NaN	26.198	NaN	NaN	NaN	NaN	NaN	K7ERI9;P02654;K7	K7ERI9;P02654;K7	Apolipoprotein C-	APOC1
NaN	23.4825	NaN	NaN	24.0532	NaN	24.6769	NaN	NaN	NaN	NaN	NaN	P02656;B0YIW2	P02656;B0YIW2	Apolipoprotein C-	APOC3
NaN	NaN	22.9086	NaN	22.7519	NaN	NaN	NaN	NaN	NaN	NaN	NaN	Q9BQE5;J3KQL8	Q9BQE5;J3KQL8	Apolipoprotein L2	APOL2
26.5046	26.6842	26.8251	26.0674	26.3611	26.1057	30.4226	29.3626	28.0651	27.2982	26.8317	27.3228	P05067;P05067-8	P05067;P05067-8,	Amyloid beta A4 p	APP
26.5961	26.3415	26.4329	25.9076	26.0788	26.0952	26.6237	26.6922	26.2089	26.1218	26.0295	26.2097	Q9UKG1;C9JAB0;(	Q9UKG1	DCC-interacting p	APPL1
26.0025	25.7785	25.6828	25.4053	25.17	25.3554	25.3023	24.6546	24.9663	24.7027	24.8021	25.2588	Q8NEU8;Q8NEU8	Q8NEU8;Q8NEU8	DCC-interacting p	APPL2
29.3138	28.8592	28.5441	28.9824	28.7838	28.4314	28.7192	28.509	29.0781	29.7082	30.1682	29.1575	P07741;P07741-2,	P07741;P07741-2,	Adenine phospho	APRT
25.6174	24.9342	24.9871	26.0929	24.772	25.1158	NaN	NaN	NaN	25.5717	25.2969	24.1748	O60306;H0YH15;I	O60306	Intron-binding prc	AQR
24.6809	24.1897	24.4066	24.5105	23.77	24.651	NaN	NaN	NaN	24.957	24.137	24.5276	P10398;Q96II5;P1	P10398;Q96II5	Serine/threonine-	ARAF
26.7087	26.2405	26.4964	26.8215	26.2253	26.6074	26.5031	26.8711	26.9238	26.6418	26.9222	26.9059	Q96P48-3;Q96P48	Q96P48-3;Q96P48	Arf-GAP with Rho	ARAP1
26.5161	NaN	25.7488	26.5684	25.6572	25.9543	NaN	26.5826	NaN	26.1961	25.9542	NaN	Q8WWN8;G5E9Y3	Q8WWN8;G5E9Y3	Arf-GAP with Rho	ARAP3
29.8074	29.8434	29.7496	29.4501	29.7198	29.6733	29.3675	29.2935	28.9754	28.925	29.2061	29.1746	P48444;B0YIW6;P	P48444;B0YIW6;P	Coatomer subunit	ARCN1
NaN	NaN	NaN	NaN	NaN	NaN	22.5457	NaN	NaN	NaN	NaN	NaN	P15514;D6RFX5	P15514;D6RFX5	Amphiregulin	AREG
30.104	28.9979	29.4087	29.4759	28.5037	29.3712	30.1565	27.9876	28.6108	29.6588	29.1511	28.388	P61204;P84077;F	P61204;P84077;F	ADP-ribosylation f	ARF3;ARF1
29.7562	27.6577	28.1002	28.5851	27.213	27.7732	28.6345	NaN	NaN	28.455	27.9565	NaN	P18085;C9JPM4;(	P18085;C9JPM4;C	ADP-ribosylation f	ARF4
26.7461	NaN	26.0646	25.6674	25.1637	26.116	NaN	NaN	NaN	26.4634	26.4399	23.6934	P84085;C9J1Z8	P84085;C9J1Z8	ADP-ribosylation f	ARF5
30.228	28.7362	29.8706	29.816	28.7448	30.0606	30.4189	28.316	28.5559	30.2349	29.4572	28.8882	P62330	P62330	ADP-ribosylation f	ARF6
26.1375	25.8577	25.8586	26.0142	25.8677	25.838	25.7404	NaN	25.5997	25.5098	25.5278	25.4504	Q8N6T3;Q8N6T3-	Q8N6T3;Q8N6T3-	ADP-ribosylation f	ARFGAP1
23.2679	24.0227	23.7067	23.5007	23.8153	NaN	NaN	NaN	23.8154	23.5485	23.0918	23.3983	Q8N6H7;A0A0D9	Q8N6H7;A0A0D9	ADP-ribosylation f	ARFGAP2
23.6318	NaN	23.2838	23.0951	NaN	23.3617	NaN	NaN	NaN	NaN	NaN	NaN	Q9NP61;Q9NP61-	Q9NP61;Q9NP61-	ADP-ribosylation f	ARFGAP3
22.7485	NaN	NaN	22.5825	NaN	NaN	NaN	NaN	NaN	NaN	NaN	NaN	Q9Y6D6;E5RIF2;E	Q9Y6D6;E5RIF2	Brefeldin A-inhibi	ARFGEF1
27.8896	26.898	27.2724	27.7582	26.7758	27.2536	27.2912	27.2845	26.5239	27.1632	26.68	25.8025	Q9Y6D5;E5RJN9	Q9Y6D5	Brefeldin A-inhibit	ARFGEF2
22.0365	NaN	21.2153	21.7027	NaN	NaN	NaN	NaN	NaN	NaN	NaN	NaN	Q5TH69	Q5TH69	Brefeldin A-inhibit	ARFGEF3
25.7208	26.0473	25.8485	25.5375	25.9321	25.7022	24.555	NaN	NaN	23.8796	24.4482	NaN	P53367;P53367-2	P53367;P53367-2,	Arfaptin-1	ARFIP1
24.7768	25.181	24.6827	23.9359	24.7538	24.4182	NaN	NaN	NaN	23.4695	24.1706	23.757	P53365;A0A087X	P53365;A0A087X	Arfaptin-2	ARFIP2
23.86	NaN	23.3553	23.5022	23.3148	23.389	22.959	NaN	NaN	NaN	NaN	NaN	Q13795;Q13795-4	Q13795;Q13795-4	ADP-ribosylation f	ARFRP1
NaN	22.9313	NaN	NaN	22.7517	NaN	NaN	NaN	NaN	NaN	NaN	NaN	P78540	P78540	Arginase-2, mitoc	ARG2
26.3756	26.2987	26.7997	26.2283	26.4228	26.5435	26.8234	25.0682	26.3267	26.8076	26.366	25.9539	Q07960;H0YE29;E	Q07960;H0YE29;E	Rho GTPase-activ	ARHGAP1
NaN	NaN	21.1622	NaN	NaN	NaN	NaN	NaN	NaN	NaN	NaN	NaN	Q6P4F7;H3BR51;(	Q6P4F7;H3BR51;(	Rho GTPase-activ	ARHGAP11A;ARH(
NaN	NaN	22.9331	22.1849	NaN	NaN	NaN	NaN	NaN	22.8648	NaN	NaN	Q8IWW6-2;Q8IW	Q8IWW6-2;Q8IW	Rho GTPase-activ	ARHGAP12
23.0345	NaN	23.1085	22.8662	NaN	NaN	23.6122	NaN	NaN	23.2578	23.5157	NaN	Q68EM7;Q68EM7	Q68EM7;Q68EM7	Rho GTPase-activ	ARHGAP17
26.8295	26.0539	26.9282	26.5968	25.6687	26.9098	27.141	24.3121	23.5565	26.9918	26.9908	24.7707	Q8N392;Q8N392-	Q8N392;Q8N392-	Rho GTPase-activ	ARHGAP18

27.4637	26.939	27.1059	26.4665	25.9782	26.1261	26.46	25.9231	25.4834	25.8992	25.6276	25.2502	Q52LW3;F8VWZ8	Q52LW3;F8VWZ8	Rho GTPase-activ	ARHGAP29
NaN	NaN	22.0287	NaN	21.7732	NaN	NaN	NaN	NaN	22.7529	NaN	NaN	Q9NRY4;A2RRE5	Q9NRY4;A2RRE5	Rho GTPase-activ	ARHGAP35;GRLF1
NaN	NaN	23.1944	23.5984	22.3601	NaN	23.3949	NaN	NaN	24.7837	23.3754	23.5293	Q9C0H5;Q9C0H5-	Q9C0H5;Q9C0H5-	Rho GTPase-activ	ARHGAP39
22.7393	NaN	23.017	22.5998	22.5714	23.2399	NaN	NaN	NaN	NaN	NaN	NaN	Q13017;Q13017-2	Q13017;Q13017-2	Rho GTPase-activ	ARHGAP5
29.9723	30.8401	30.6915	30.1348	30.348	30.6451	30.5643	29.7013	29.6979	30.7624	30.4432	30.4188	P52565;J3QQX2;J	P52565;J3QQX2;J	Rho GDP-dissocia	ARHGDIA
30.4055	30.8065	30.557	30.1392	30.1601	30.1673	30.257	29.159	29.2064	30.3301	30.4598	29.9727	P52566;H0YGX7;F	P52566;H0YGX7;F	Rho GDP-dissocia	ARHGDIB
28.2964	27.5603	27.938	28.0667	27.6329	27.924	27.5066	27.868	27.5377	27.7808	27.9094	27.634	Q92888;Q92888-3	Q92888;Q92888-3	Rho guanine nucl	ARHGEF1
24.6094	23.3322	24.3484	24.5426	23.6269	23.9384	25.2244	24.7271	24.4179	24.3527	23.8582	NaN	O15013;H0YAN8;(	O15013;H0YAN8;(	Rho guanine nucl	ARHGEF10
21.8923	NaN	NaN	NaN	NaN	NaN	NaN	NaN	NaN	NaN	NaN	NaN	Q9NZN5;Q9NZN5-	Q9NZN5;Q9NZN5-	Rho guanine nucl	ARHGEF12
24.9562	24.4812	24.5496	24.7636	24.5033	24.3717	NaN	NaN	NaN	24.5515	24.3882	NaN	Q6ZSZ5;A0A087W	Q6ZSZ5;A0A087W	Rho guanine nucl	ARHGEF18
28.1564	27.0614	27.4261	27.6917	27.1206	26.9139	26.5608	27.0993	26.9016	26.5105	26.4045	26.4837	Q92974;Q92974-2	Q92974;Q92974-2	Rho guanine nucl	ARHGEF2
25.0176	23.9996	24.5139	24.8552	23.929	24.3703	24.1139	NaN	24.374	23.7895	23.7358	23.5979	Q8N1W1;Q8N1W	Q8N1W1;Q8N1W	Rho guanine nucl	ARHGEF28
23.9869	23.5849	23.7869	23.9023	24.1011	24.1055	NaN	NaN	NaN	NaN	NaN	NaN	Q8TER5;Q8TER5-4	Q8TER5;Q8TER5-4	Rho guanine nucl	ARHGEF40
23.3617	24.006	23.8901	23.2422	23.5793	23.207	24.448	23.9513	NaN	NaN	NaN	NaN	Q14155-1;B1ALK7	Q14155-1;B1ALK7	Rho guanine nucl	ARHGEF7
24.1199	NaN	23.9886	25.0022	23.4757	23.6037	NaN	NaN	NaN	23.6203	24.0342	NaN	O14497;O14497-3	O14497;O14497-3	AT-rich interactive	ARID1A
22.9491	NaN	22.3423	22.7813	NaN	NaN	NaN	NaN	NaN	23.458	22.7552	NaN	Q9Y4X5;A0A087W	Q9Y4X5	E3 ubiquitin-prote	ARIH1
23.9214	NaN	23.1017	23.1937	NaN	24.0014	23.9852	NaN	NaN	24.3414	24.0348	24.3821	O95376;C9JZ71	O95376	E3 ubiquitin-prote	ARIH2
24.0357	25.8801	26.0341	26.2405	25.8555	25.9152	26.1603	25.9239	26.0706	25.235	25.4439	25.4446	P40616;P40616-2,	P40616;P40616-2,	ADP-ribosylation f	ARL1
23.0116	22.5044	23.364	22.9613	23.1644	23.3554	22.5475	NaN	NaN	23.6079	23.0423	NaN	Q3SXY8;Q3SXY8-2	Q3SXY8	ADP-ribosylation f	ARL13B
23.2564	22.624	23.296	23.3572	23.0696	NaN	NaN	22.762	23.5443	23.5342	23.3807	NaN	Q9NXU5;A0A087V	Q9NXU5;A0A087V	ADP-ribosylation f	ARL15
26.5928	26.201	25.8809	26.6687	25.6444	25.4859	24.7662	25.2086	25.2454	25.9296	25.2284	24.689	P36404;V9GYD0;F	P36404;V9GYD0;F	ADP-ribosylation f	ARL2;ARL2-SNX15
NaN	NaN	NaN	NaN	NaN	NaN	NaN	NaN	NaN	NaN	NaN	24.4722	Q9Y2Y0;H3BU49;(	Q9Y2Y0;H3BU49;(	ADP-ribosylation f	ARL2BP
26.122	26.5254	26.2547	25.4036	26.6962	26.3331	25.3038	25.634	26.3677	26.0002	26.5246	26.3669	P36405	P36405	ADP-ribosylation f	ARL3
NaN	NaN	NaN	NaN	NaN	NaN	22.8404	NaN	NaN	NaN	NaN	NaN	Q96KC2;Q9Y689;(	Q96KC2;Q9Y689;(	ADP-ribosylation f	ARL5B;ARL5A
NaN	NaN	21.8333	21.5748	NaN	22.0815	NaN	NaN	NaN	NaN	22.5058	NaN	Q66PJ3;Q66PJ3-2,	Q66PJ3;Q66PJ3-2,	ADP-ribosylation f	ARL6IP4
25.1839	24.168	24.4833	25.0065	23.3683	23.943	25.6106	23.5889	24.5856	25.1646	24.1084	NaN	O75915;F8WF90;(	O75915;F8WF90;(	PRA1 family prote	ARL6IP5
22.4154	NaN	NaN	NaN	NaN	NaN	NaN	NaN	NaN	NaN	NaN	NaN	Q96BM9;A0A087/	Q96BM9;A0A087/	ADP-ribosylation f	ARL8A
26.0305	25.5682	25.222	25.5065	25.2834	25.2675	26.0845	NaN	26.2497	25.7901	25.715	26.5759	Q9NVJ2;Q9NVJ2-2	Q9NVJ2	ADP-ribosylation f	ARL8B
26.5842	26.0744	26.321	26.5175	26.0543	26.4419	26.1681	26.5647	26.2847	26.6479	26.7275	26.7837	Q6NXE6;Q6NXE6-	Q6NXE6;Q6NXE6-	Armadillo repeat-	ARMC6
22.6672	NaN	NaN	23.0941	22.4428	NaN	NaN	NaN	NaN	22.5583	NaN	NaN	Q8IUR7;Q8IUR7-8	Q8IUR7;Q8IUR7-8	Armadillo repeat-	ARMC8
22.1556	NaN	21.2317	22.0498	NaN	21.2951	NaN	NaN	NaN	NaN	NaN	NaN	Q7Z3E5;A0A087X	Q7Z3E5;A0A087X	LisH domain-conti	ARMC9
21.8987	NaN	21.5758	21.9569	NaN	NaN	NaN	NaN	NaN	NaN	NaN	NaN	Q7L311	Q7L311	Armadillo repeat-	ARMCX2
22.2389	NaN	NaN	NaN	NaN	22.2452	NaN	NaN	NaN	NaN	NaN	NaN	Q9UH62	Q9UH62	Armadillo repeat-	ARMCX3
23.2044	24.1402	23.2356	22.6891	23.1322	23.1225	23.2473	NaN	NaN	23.4324	23.0947	NaN	Q9H993;F5GZY1	Q9H993;F5GZY1	Protein-glutamate	ARMT1
27.3222	27.5597	27.6668	27.5211	27.8634	27.7895	27.9443	27.9375	27.563	28.7349	28.6568	27.6282	Q92747;E9PF58;C	Q92747;E9PF58;C	Actin-related prot	ARPC1A
28.6475	28.9392	29.0858	28.9581	29.2137	29.461	29.0971	29.27	28.6744	29.7533	29.6068	29.277	O15143;C9J4Z7;F	O15143	Actin-related prot	ARPC1B
29.5294	29.74	29.6187	29.1759	29.7187	29.7337	29.9598	29.8132	29.4336	30.3251	30.2165	29.9306	O15144;C9JTV5;G	O15144	Actin-related prot	ARPC2
29.3436	29.4889	29.1102	28.9488	29.1403	29.0895	29.6368	29.6269	29.0771	30.2056	29.87	29.3293	O15145;C9JZD1;F	O15145;C9JZD1	Actin-related prot	ARPC3
28.462	28.7272	28.897	28.5925	29.0715	29.1612	28.8815	28.114	28.8272	28.9835	29.3196	29.1894	P59998;P59998-3,	P59998;P59998-3,	Actin-related prot	ARPC4;ARPC4-TTL
27.1412	28.169	27.6707	27.4446	27.8764	27.8229	27.964	28.2756	28.4089	28.3214	28.4679	28.2623	O15511;B1ALC0;C	O15511;B1ALC0;C	Actin-related prot	ARPC5
26.3549	26.478	26.3794	26.2532	26.8167	26.5689	26.064	26.2548	25.939	27.0215	26.9035	26.7001	Q9BPX5	Q9BPX5	Actin-related prot	ARPC5L
NaN	23.8025	23.2677	23.0679	23.3232	23.048	23.9266	23.701	NaN	24.0679	23.6914	23.6604	Q7Z6K5;A0A0A6Y	Q7Z6K5;A0A0A6Y	Arpin	ARPIN;C15orf38-A
24.0842	24.6153	24.3623	23.946	24.7666	25.0475	25.0863	24.6788	25.0705	25.348	25.2131	NaN	P49407;P49407-2,	P49407;P49407-2,	Beta-arrestin-1	ARRB1
23.4334	NaN	22.9199	22.9998	22.6318	NaN	NaN	NaN	NaN	22.9928	23.0593	NaN	P32121;I3L412;K7	P32121;I3L412;K7	Beta-arrestin-2	ARRB2
23.2874	23.4968	23.8074	22.8909	23.4599	23.9831	23.4706	NaN	23.2877	24.1769	24.075	NaN	Q8N5I2;Q5T370;F	Q8N5I2;Q5T370;F	Arrestin domain-c	ARRDC1
21.7293	NaN	NaN	NaN	NaN	NaN	NaN	NaN	NaN	NaN	NaN	NaN	Q96B67	Q96B67	Arrestin domain-c	ARRDC3
23.5558	23.5561	23.1752	22.8611	22.5934	23.0932	24.9875	23.4447	23.6006	23.2084	NaN	23.1237	Q13510;E7EMM4	Q13510;E7EMM4	Acid ceramidase;A	ASAH1
25.8011	25.0299	25.5982	25.4774	24.7661	25.2554	25.0825	24.5437	NaN	24.5753	24.6043	23.6277	Q9ULH1;A0A0A0N	Q9ULH1;A0A0A0N	Arf-GAP with SH3	ASAP1
NaN	NaN	NaN	21.7361	NaN	NaN	NaN	NaN	NaN	NaN	NaN	NaN	O43150;O43150-2	O43150;O43150-2	Arf-GAP with SH3	ASAP2
22.4049	NaN	22.3171	22.4861	22.935	NaN	NaN	NaN	NaN	22.5191	NaN	NaN	Q9NWX5;F6TX30;	Q9NWX5;F6TX30;	Ankyrin repeat an	ASB6
24.0172	NaN	23.2269	23.3889	NaN	NaN	NaN	NaN	NaN	NaN	NaN	NaN	Q9H1I8;Q9H1I8-3	Q9H1I8;Q9H1I8-3	Activating signal c	ASCC2
26.454	24.7829	25.8435	26.0892	25.1587	25.484	24.7081	24.3259	NaN	25.477	25.3576	24.4571	Q8N3C0;Q8N3C0-	Q8N3C0	Activating signal c	ASCC3
22.9226	NaN	23.3864	22.6848	23.6256	NaN	NaN	NaN	NaN	22.8543	NaN	NaN	Q9NVP2;K7ES22;k	Q9NVP2;K7ES22;H	Histone chaperon	ASF1B
23.4937	NaN	22.7524	23.0104	NaN	NaN	NaN	NaN	NaN	23.6407	22.8097	NaN	Q9UBL3;F5H8F7;(	Q9UBL3;F5H8F7;(	Set1/Ash2 histone	ASH2L
24.2007	24.5407	24.4904	24.2701	24.34	24.2916	NaN	NaN	NaN	23.9718	23.5536	NaN	O95671;O95671-2	O95671;O95671-2	N-acetylserotonin	ASMTL
26.4992	26.3258	26.4715	26.4126	26.4461	26.5633	26.8946	26.9752	27.4639	26.5141	26.901	27.2616	O43681;A0A087W	O43681;A0A087W	ATPase ASNA1	ASNA1
28.5609	28.1816	28.0826	27.942	27.8777	27.7834	27.901	27.826	27.4576	29.0743	28.7244	28.0683	P08243;P08243-2,	P08243;P08243-2,	Asparagine synth	ASNS
25.3561	25.2011	25.3682	25.5859	25.1597	25.3783	25.2816	25.8518	26.6227	25.6088	25.446	25.4064	Q12797;Q12797-1	Q12797;Q12797-1	Aspartyl/asparagi	ASPH
24.1969	23.6801	23.4206	23.7756	24.2417	NaN	NaN	NaN	NaN	23.1496	24.2097	NaN	Q9BZE9;Q9BZE9-2	Q9BZE9;Q9BZE9-2	Tether containing	ASPSCR1
24.5766	25.3199	24.3851	23.2545	24.688	24.4463	23.3958	NaN	NaN	NaN	23.5485	NaN	P00966;Q5T6L5;Q	P00966	Argininosuccinate	ASS1

NaN	21.0356	NaN	21.2293	21.4378	NaN	NaN	NaN	NaN	NaN	21.9197	21.431	Q9NVM9;F5H5W	Q9NVM9;F5H5W	Protein asunder h	ASUN
23.1596	NaN	22.9646	23.1187	NaN	NaN	NaN	NaN	NaN	NaN	NaN	NaN	Q8NBU5;Q8NBU5	Q8NBU5;Q8NBU5	ATPase family AA	ATAD1
25.3765	25.9594	25.2364	25.3073	26.1312	25.182	25.6219	26.3091	27.1341	24.5115	25.6714	25.8092	Q9NVI7-2;Q9NVI	Q9NVI7-2;Q9NVI	ATPase family AA	ATAD3A;ATAD3B
24.8871	24.7776	24.7631	25.023	24.9342	24.3467	24.4817	24.5626	25.4005	25.1223	25.3679	25.51	O95260;H0Y5C2;E	O95260;H0Y5C2;E	Arginyl-tRNA--pro	ATE1
23.3097	23.3543	NaN	NaN	NaN	NaN	NaN	NaN	NaN	NaN	NaN	NaN	Q9BSB4;F8VQD9;	Q9BSB4;F8VQD9;	Autophagy-relate	ATG101
22.6411	NaN	22.7345	NaN	22.6167	NaN	22.6301	NaN	NaN	NaN	NaN	NaN	Q676U5-2;Q676U	Q676U5-2;Q676U	Autophagy-relate	ATG16L1
26.5115	24.5731	25.2356	26.1913	25.0246	24.782	24.9543	24.6219	23.4026	24.5118	24.1445	23.0441	Q2TAZ0;Q2TAZ0-	Q2TAZ0;Q2TAZ0-	Autophagy-relate	ATG2A
24.8611	24.5345	24.7026	24.8089	24.7124	24.624	25.2739	25.2852	25.2477	24.3538	24.9449	25.2039	Q9NT62;Q9NT62-	Q9NT62;Q9NT62-	Ubiquitin-like-con	ATG3
NaN	NaN	20.7881	NaN	NaN	NaN	NaN	NaN	NaN	NaN	NaN	NaN	Q9Y4P1;C9JQ72;C	Q9Y4P1;C9JQ72;C	Cysteine protease	ATG4B
24.0407	23.9423	23.7029	23.4142	23.7184	24.1242	NaN	NaN	NaN	23.7914	23.5228	23.5596	Q9H1Y0;L7UQJ2;	Q9H1Y0;L7UQJ2;	Autophagy protei	ATG5
25.0947	24.3424	24.4291	24.9711	24.512	24.2167	24.722	23.7524	25.5124	25.1016	25.0451	25.3131	O95352;O95352-	O95352;O95352-	Ubiquitin-like mo	ATG7
24.2845	23.9171	24.3844	24.126	24.0953	23.9009	24.9587	24.8379	23.9929	24.139	23.2544	24.0807	Q7Z3C6;Q7Z3C6-	Q7Z3C6;Q7Z3C6-	Autophagy-relate	ATG9A
30.4928	30.1825	30.1174	30.3427	30.1833	30.1261	30.2365	29.9313	29.9404	30.4861	30.3827	30.3093	P31939;P31939-2	P31939;P31939-2	Bifunctional purin	ATIC
24.6754	25.2791	25.4212	25.1352	25.7195	25.7362	25.7997	25.9603	26.201	26.0666	25.7398	25.9679	Q8NHH9;Q8NHH	Q8NHH9;Q8NHH	Atlastin-2	ATL2
NaN	NaN	NaN	NaN	NaN	NaN	NaN	NaN	NaN	NaN	20.2068	NaN	F5H6I7	F5H6I7		ATL3
28.072	27.8511	28.3342	28.05	28.0313	28.313	28.9362	29.021	28.956	29.6611	28.8674	28.6941	Q6DD88;F5GWF8	Q6DD88	Atlastin-3	ATL3
24.5748	23.3541	24.5628	25.0308	24.3762	24.9279	NaN	NaN	NaN	23.8642	23.8841	NaN	Q13315;E9PIN0;Q	Q13315	Serine-protein kin	ATM
24.9326	24.1717	24.0713	24.2435	23.8329	NaN	25.5944	NaN	NaN	NaN	NaN	24.094	O00244;E5RGN3;	O00244;E5RGN3;	Copper transport	ATOX1
25.0152	NaN	25.8479	25.2412	25.3151	NaN	25.6265	25.8507	26.0478	26.1702	24.8128	26.0516	P98196;H0Y547;E	P98196;H0Y547;E	Probable phosph	ATP11A
25.4734	24.8515	26.135	25.5028	24.6693	25.4911	24.3429	23.7311	22.8746	24.5906	23.5197	23.9973	Q9Y2G3;H7C4W6	Q9Y2G3;H7C4W6	Probable phosph	ATP11B
24.2842	24.1389	25.1786	24.583	24.4323	25.1539	24.0373	NaN	24.1166	25.5606	24.9866	24.9418	A0A067XG54;Q8	A0A067XG54;Q8	Phospholipid-tran	ATP11C
NaN	NaN	NaN	21.7199	NaN	NaN	NaN	NaN	22.3504	22.2954	22.0745	NaN	Q9HD20;Q9HD20	Q9HD20;Q9HD20	Manganese-trans	ATP13A1
22.1189	NaN	21.5502	NaN	NaN	NaN	NaN	NaN	NaN	NaN	NaN	NaN	Q9NQ11;Q9NQ11	Q9NQ11;Q9NQ11	Probable cation-t	ATP13A2
26.1198	26.1667	26.5133	26.5761	26.2789	26.2938	24.7504	NaN	25.265	25.897	25.7642	25.297	Q9H7F0;A0A087V	Q9H7F0;A0A087V	Probable cation-t	ATP13A3
33.5698	32.9721	33.8183	33.8149	33.151	34.1024	33.6904	32.883	33.5383	34.4158	33.8159	34.0685	P05023;P05023-4	P05023;P05023-4	Sodium/potassiu	ATP1A1
30.4843	29.8045	30.6239	30.5373	29.8564	30.8675	30.4186	29.3249	29.9252	30.9606	30.2931	30.5308	P05026;P05026-2	P05026;P05026-2	Sodium/potassiu	ATP1B1
31.0003	29.9471	31.0514	31.3513	30.2859	31.2802	31.2426	30.5942	30.7961	32.0411	31.4886	31.5798	P54709;C9JA36;P	P54709	Sodium/potassiu	ATP1B3
29.2555	28.7045	28.8874	29.2922	28.573	28.8587	29.4617	29.0771	29.0759	29.3908	28.5637	28.3845	P16615;P16615-5	P16615;P16615-5	Sarcoplasmic/end	ATP2A2
30.6998	30.7392	31.4916	30.8963	31.0757	31.8059	31.2134	30.8612	31.4117	31.8646	31.4432	32.124	P20020;P20020-6	P20020;P20020-6	Plasma membran	ATP2B1
26.7693	26.7991	27.5915	26.6786	27.2553	27.8956	27.2285	NaN	27.8482	27.9659	27.5973	28.4442	Q16720;Q16720-	Q16720;Q16720-	Plasma membran	ATP2B3
26.9385	26.8198	27.6668	27.3605	27.1184	28.0401	27.3848	26.6442	27.4122	28.5309	27.4937	27.8379	P23634;P23634-6	P23634;P23634-6	Plasma membran	ATP2B4
25.5038	25.2693	25.3635	25.242	24.9923	25.3434	25.1297	24.9297	25.4568	24.9665	24.6284	25.2561	P98194;P98194-8	P98194;P98194-8	Calcium-transport	ATP2C1
28.906	29.8779	29.3324	29.5375	29.8698	29.4314	28.9515	30.0989	31.0465	28.3053	29.1346	29.6583	P25705;P25705-2	P25705;P25705-2	ATP synthase sub	ATP5A1
29.3082	30.2075	29.5367	29.6818	30.2606	29.6965	29.0082	30.7204	31.5814	29.1286	29.9963	30.2186	P06576;H0YH81;F	P06576;H0YH81;F	ATP synthase sub	ATP5B
24.4445	25.2953	24.8655	25.0744	25.2362	24.7354	23.6878	25.3567	26.0781	23.7784	23.8898	24.8606	P36542;P36542-2	P36542;P36542-2	ATP synthase sub	ATP5C1
NaN	NaN	NaN	20.1745	NaN	NaN	NaN	NaN	NaN	NaN	NaN	NaN	P30049	P30049	ATP synthase sub	ATP5D
NaN	25.0329	24.5177	24.3179	25.1534	NaN	NaN	NaN	NaN	NaN	NaN	NaN	P56381;Q5VTU8	P56381;Q5VTU8	ATP synthase sub	ATP5E;ATP5EP2
24.315	24.532	24.4359	24.6968	24.6559	24.7695	23.801	24.8895	NaN	23.2149	23.5703	24.4248	P24539;Q5QNZ2	P24539;Q5QNZ2	ATP synthase F(0)	ATP5F1
25.365	26.2261	25.4747	25.7347	26.1158	25.6136	25.4254	26.5469	27.3665	24.1257	25.0735	25.9656	O75947;O75947-	O75947;O75947-	ATP synthase sub	ATP5H
NaN	24.9888	24.4862	24.5564	25.3421	24.7309	NaN	NaN	26.8439	NaN	NaN	NaN	P56385	P56385	ATP synthase sub	ATP5I
22.8212	24.3686	23.7313	23.2228	23.7753	23.8182	23.2005	25.0868	24.7862	NaN	23.1513	23.4046	P18859;A8MUH2;	P18859;A8MUH2;	ATP synthase-cou	ATP5J
NaN	NaN	23.1311	23.6027	NaN	NaN	NaN	23.174	24.3889	NaN	NaN	22.9738	G3V325;P56134-4	G3V325;P56134-4	ATP synthase sub	ATP5J2-PTCD1;ATI
24.7159	25.3349	25.3027	24.9389	25.7378	25.204	NaN	NaN	26.3136	NaN	23.7894	25.3231	O75964;E9PN17	O75964;E9PN17	ATP synthase sub	ATP5L
25.4774	26.6046	26.0769	26.0981	26.7661	26.4271	25.3056	27.2605	28.2752	24.884	26.2311	26.6524	P48047;H7C0C1;H	P48047;H7C0C1	ATP synthase sub	ATP5O
24.9298	24.6997	24.9882	25.0765	24.4468	24.7814	26.0365	NaN	26.2637	25.3051	23.8983	25.7451	Q15904;A0A0C4D	Q15904;A0A0C4D	V-type proton AT	ATP6AP1
24.7679	24.2727	24.6353	23.6299	24.5224	23.595	27.2507	25.9467	25.2004	25.9322	24.1057	23.6991	O75787;O75787-	O75787;O75787-	Renin receptor	ATP6AP2
27.0073	26.5805	26.7643	27.3092	26.5911	26.7686	25.9854	25.5929	26.547	25.9015	25.3453	26.0577	Q93050-3;Q9305	Q93050-3;Q9305	V-type proton AT	ATP6V0A1
25.2096	24.3897	24.669	24.5961	24.4007	24.6031	24.5566	25.2397	24.5053	24.0158	23.7895	24.1937	Q9Y487;F5H5F3;C	Q9Y487;F5H5F3	V-type proton AT	ATP6V0A2
NaN	NaN	NaN	NaN	NaN	NaN	NaN	NaN	23.5972	NaN	NaN	NaN	P27449	P27449	V-type proton AT	ATP6V0C
26.8277	27.0257	26.7312	27.0647	26.9358	26.7772	25.2443	25.369	24.0947	25.685	25.6893	25.84	P61421;F5GYQ1;	P61421;F5GYQ1;	V-type proton AT	ATP6V0D1
NaN	NaN	NaN	NaN	NaN	NaN	22.7839	NaN	NaN	NaN	NaN	NaN	Q8N8Y2	Q8N8Y2	V-type proton AT	ATP6V0D2
28.3405	29.0856	28.5606	28.3989	28.9376	28.7033	28.4101	28.8044	28.6612	28.2104	28.4947	28.5316	P38606;P38606-2	P38606;P38606-2	V-type proton AT	ATP6V1A
27.9565	28.887	28.241	27.8392	28.7153	28.2122	28.1899	28.7588	28.3106	27.8817	28.0724	28.5738	P21281;H0YC04;P	P21281	V-type proton AT	ATP6V1B2
26.2342	25.4093	25.2738	25.9909	25.258	25.4713	25.7647	NaN	25.5182	25.3376	24.9943	25.4378	P21283;E7EV59	P21283;E7EV59	V-type proton AT	ATP6V1C1
24.2931	24.2282	24.2315	24.5993	23.9024	24.2404	23.518	NaN	NaN	23.797	23.1576	NaN	Q9Y5K8;G3V559;	Q9Y5K8;G3V559;	V-type proton AT	ATP6V1D
27.4182	27.7486	27.3241	27.0543	27.5793	27.1656	27.1182	27.4317	27.4233	26.8677	26.9037	27.2808	P36543;P36543-2	P36543;P36543-2	V-type proton AT	ATP6V1E1
NaN	NaN	NaN	NaN	23.2636	NaN	NaN	24.4531	NaN	NaN	NaN	NaN	Q16864;Q16864-	Q16864;Q16864-	V-type proton AT	ATP6V1F
26.1089	26.5093	26.0878	25.5963	26.3365	26.1516	26.1966	26.4816	25.5983	25.66	25.6875	26.065	O75348;O95670;F	O75348	V-type proton AT	ATP6V1G1
26.3358	26.1833	26.5161	26.2682	26.2331	26.543	26.2268	26.6517	26.4769	26.1535	26.216	26.2187	Q9UI12;Q9UI12-2	Q9UI12;Q9UI12-2	V-type proton AT	ATP6V1H
23.1759	NaN	23.6156	23.857	22.872	23.6565	NaN	NaN	NaN	23.5546	23.0606	23.456	O43520;K7EQC4	O43520	Phospholipid-tran	ATP8B1

NaN	NaN	NaN	22.3448	NaN	NaN	NaN	NaN	NaN	22.3972	NaN	NaN		O75110;A0A0A0M	O75110;A0A0A0M	Probable phospho	ATP9A
NaN	NaN	NaN	NaN	NaN	NaN	NaN	NaN	NaN	NaN	19.3528	NaN	O43861;O43861-2	O43861;O43861-2	Probable phospho	ATP9B
NaN	NaN	NaN	NaN	NaN	NaN	29.1053	NaN	NaN	NaN	NaN	NaN	E9PPX6;Q5TC12-3	E9PPX6;Q5TC12-3		ATPAF1
23.5326	25.9422	24.0024	24.0486	26.5839	24.0541	25.8928	26.6384	25.472	24.1402	24.6093	NaN	Q9UII2;A0A0B4J2	Q9UII2;A0A0B4J2	ATPase inhibitor,	ATPIF1
23.2917	NaN	23.3026	23.5264	NaN	NaN	NaN	NaN	NaN	NaN	23.0069	NaN	Q13535;Q13535-2	Q13535;Q13535-2	Serine/threonine-	ATR
NaN	23.9128	NaN	22.8284	NaN	23.9201	24.0078	NaN	23.8056	23.7823	24.2247	23.8279	O75882;O75882-3	O75882;O75882-3	Attractin	ATRN
NaN	NaN	NaN	22.2557	NaN	NaN	NaN	NaN	NaN	NaN	NaN	NaN	P46100;P46100-4,	P46100;P46100-4,	Transcriptional re	ATRX
26.3385	25.2764	25.7429	26.0758	25.1412	25.5487	26.0177	25.0683	25.1026	25.7265	25.5225	25.2118	Q9UBB4;Q9UBB4-	Q9UBB4;Q9UBB4-	Ataxin-10	ATXN10
25.2575	24.8031	NaN	24.5072	24.4039	NaN	NaN	NaN	NaN	NaN	23.9802	NaN	Q99700;H0YH87;F	Q99700;H0YH87;F	Ataxin-2	ATXN2
28.0264	28.218	28.1584	27.1503	28.1006	27.1054	25.9439	26.3663	24.1297	25.6696	26.2862	25.0439	Q8WWM7;Q8WW	Q8WWM7;Q8WW	Ataxin-2-like prote	ATXN2L
22.8287	NaN	22.805	22.6048	NaN	22.7358	22.5721	NaN	NaN	22.7771	NaN	NaN	F5H211	F5H211		ATXN3
24.2031	24.6103	24.9456	24.66	24.3147	24.8565	25.7283	NaN	25.2802	25.3847	NaN	NaN	Q9Y679;Q9Y679-3	Q9Y679;Q9Y679-3	Ancient ubiquitou	AUP1
24.6253	24.7789	26.1539	25.6182	26.1391	26.0162	24.9882	23.5551	24.4782	26.4067	26.7418	26.0691	O14965;A3KFJ0;Q	O14965;A3KFJ0;Q	Aurora kinase A	AURKA
26.074	26.1054	26.4272	26.2446	26.3147	26.2274	27.8895	27.3705	27.7167	26.7552	26.64	26.268	Q96GD4;Q96GD4	Q96GD4;Q96GD4	Aurora kinase B	AURKB
22.4733	NaN	NaN	NaN	NaN	NaN	NaN	NaN	NaN	NaN	NaN	NaN	Q8NBF6;B8ZZW5;	Q8NBF6;B8ZZW5;	Late secretory pat	AVL9
26.0684	26.3142	26.6517	25.9928	25.7745	26.4677	26.971	26.9739	26.7248	26.1287	25.2922	25.5696	P30530;P30530-2,	P30530;P30530-2,	Tyrosine-protein k	AXL
21.6665	NaN	NaN	NaN	NaN	NaN	NaN	NaN	NaN	NaN	NaN	NaN	Q9H6S1;C9JVK8;C	Q9H6S1;C9JVK8;C	5-azacytidine-indu	AZI2
31.0262	32.9621	32.8371	31.8136	33.2352	32.9706	31.9727	33.3378	33.3048	32.0609	32.4963	32.2731	P61769;H0YLF3;F5	P61769;H0YLF3;F5	Beta-2-microglobu	B2M
24.9678	NaN	25.2541	24.6709	NaN	24.8625	25.5208	NaN	NaN	24.834	NaN	NaN	O94766;G3V150;C	O94766;G3V150;C	Galactosylgalacto	B3GAT3
NaN	NaN	NaN	NaN	NaN	NaN	24.7303	NaN	NaN	NaN	NaN	NaN	P15291;P15291-2,	P15291;P15291-2	Beta-1,4-galactosy	B4GALT1
21.5316	NaN	NaN	NaN	21.6337	NaN	24.4319	NaN	NaN	NaN	NaN	NaN	O43286	O43286	Beta-1,4-galactosy	B4GALT5
20.4743	NaN	NaN	NaN	NaN	NaN	NaN	NaN	NaN	NaN	NaN	NaN	O43505	O43505	Beta-1,4-glucuron	B4GAT1
24.8728	24.9132	24.9018	24.5246	24.876	25.1214	25.2237	24.8308	25.0979	NaN	25.0662	24.9197	Q9NWV8;M0R0I0	Q9NWV8;M0R0I0	BRISC and BRCA1-	BABAM1
22.1708	22.5423	NaN	21.2105	NaN	NaN	NaN	NaN	NaN	NaN	NaN	NaN	Q92934;F5GYS3;A	Q92934;F5GYS3;A	Bcl2-associated ag	BAD
28.0879	28.342	28.1638	28.2598	28.5735	28.3109	27.6954	28.7476	28.1493	28.1424	28.4038	28.6878	O95816;O95816-2	O95816;O95816-2	BAG family molec	BAG2
26.9114	27.6169	27.0791	26.5976	27.3565	26.8972	26.9163	27.431	27.3609	26.7925	27.1344	27.493	O95817;C9JFK9	O95817	BAG family molec	BAG3
22.8015	23.6361	23.4878	23.6441	23.8689	23.6957	23.4153	24.3537	24.3196	24.0243	23.7298	24.5203	Q9UL15;Q9UL15-2	Q9UL15;Q9UL15-2	BAG family molec	BAG5
27.8689	27.3853	27.667	28.11	27.5749	27.8333	24.879	27.2577	26.7912	27.2555	26.9747	27.0859	P46379;A0A024R(	P46379;A0A024R(	Large proline-rich	BAG6;BAT3
27.6382	28.1242	28.0867	27.5497	27.9748	28.0482	26.8442	26.2241	27.119	27.8077	27.8574	27.8611	Q9UQB8-5;I3L4C2	Q9UQB8-5;I3L4C2	Brain-specific ang	BAIAP2
NaN	22.5842	22.3295	NaN	22.3797	NaN	NaN	NaN	NaN	NaN	NaN	NaN	Q9UHR4	Q9UHR4	Brain-specific ang	BAIAP2L1
27.8897	29.0432	28.4508	28.3003	28.7867	28.2297	29.2445	28.4229	27.8697	28.8289	29.0199	28.3317	O75531	O75531	Barrier-to-autoint	BANF1
24.8225	24.9034	24.423	24.4974	24.7133	24.346	23.0362	24.1708	NaN	23.9198	23.59	23.7506	Q8IXM2;Q8IXM2-	Q8IXM2;Q8IXM2-	Chromatin comple	BAP18;C17orf49;R
31.9453	31.3178	31.4998	31.3539	30.9009	31.5782	31.9796	29.7052	29.9431	32.3745	31.2243	30.3029	P80723;P80723-2,	P80723;P80723-2	Brain acid soluble	BASP1
25.584	25.301	25.7575	25.4691	25.4498	25.8674	25.7577	25.2242	25.636	25.5227	24.9773	25.3834	Q07812;Q07812-2	Q07812;Q07812-2	Apoptosis regulat	BAX
NaN	NaN	NaN	24.9806	NaN	NaN	NaN	NaN	NaN	NaN	NaN	NaN	Q9NRL2;Q9NRL2-	Q9NRL2;Q9NRL2-	Bromodomain adj	BAZ1A
24.9729	23.417	24.1244	25.1898	23.2933	23.8712	NaN	NaN	NaN	22.9633	23.3924	NaN	Q9UIG0;Q9UIG0-2	Q9UIG0;Q9UIG0-2	Tyrosine-protein k	BAZ1B
22.8109	NaN	23.8542	23.7114	23.5112	NaN	24.7924	23.4347	24.1301	25.2233	24.0469	NaN	P50895;A0A087W	P50895;A0A087W	Basal cell adhesio	BCAM
24.9316	24.3907	24.952	24.9948	24.7331	25.151	25.4475	25.5564	NaN	25.2389	24.7261	24.9682	Q9UHQ4-2;Q9UH	Q9UHQ4-2;Q9UH	B-cell receptor-as	BCAP29
29.0261	28.8063	29.1484	28.4861	28.1942	29.0279	29.1732	28.3109	27.9943	28.8749	27.9002	27.5396	P51572-2;P51572,	P51572-2;P51572	B-cell receptor-as	BCAP31
24.9076	24.7198	25.0194	24.4519	24.7085	25.149	26.0272	NaN	NaN	24.7216	25.2019	NaN	P56945;P56945-3,	P56945;P56945-3,	Breast cancer anti	BCAR1
25.3924	25.4631	25.3459	25.0728	24.9518	25.2812	26.0211	26.0003	24.4893	25.4707	25.8135	25.9313	O75815;O75815-3	O75815;O75815-3	Breast cancer anti	BCAR3
26.8464	27.0993	26.9537	27.1647	26.9366	26.7269	25.8991	NaN	28.2162	26.6674	27.0558	26.6617	O75934	O75934	Pre-mRNA-splicing	BCAS2
21.4981	NaN	NaN	NaN	NaN	NaN	NaN	NaN	NaN	NaN	NaN	NaN	Q9H6U6;Q9H6U6	Q9H6U6;Q9H6U6-	Breast carcinoma-	BCAS3
22.2797	23.1441	22.6812	22.3765	23.8819	22.6506	24.0447	24.0043	24.5107	22.9321	23.6689	24.6341	P54687;P54687-5,	P54687;P54687-5,	Branched-chain-a	BCAT1
23.2145	23.4415	22.9153	22.7584	23.8655	23.0323	NaN	22.6121	23.7475	22.1452	22.5404	NaN	O15382;B3KSI3;M	O15382;B3KSI3;M	Branched-chain-a	BCAT2
22.5398	NaN	NaN	NaN	NaN	NaN	NaN	NaN	NaN	NaN	NaN	NaN	Q9P287;Q9P287-3	Q9P287;Q9P287-3	BRCA2 and CDKN1	BCCIP
26.1723	23.5758	24.4653	25.2487	23.6669	24.4873	24.4743	23.3606	NaN	25.2244	24.5054	23.8508	Q9P287-2	Q9P287-2	BRCA2 and CDKN1	BCCIP
23.8451	24.8861	24.8335	23.538	25.2409	24.2914	24.0668	NaN	NaN	NaN	23.9835	NaN	O95999;A0A087W	O95999;A0A087W	B-cell lymphoma/	BCL10
21.7818	NaN	22.9246	22.2888	NaN	22.4695	NaN	23.2029	NaN	NaN	NaN	NaN	Q07817;Q5QP56;(	Q07817;Q5QP56;(	Bcl-2-like protein	BCL2L1
NaN	23.1895	23.2809	NaN	23.7841	NaN	NaN	NaN	NaN	24.0337	23.4438	NaN	A0A087WSV0;Q9I	A0A087WSV0;Q9I	Bcl-2-like protein	BCL2L12
25.4542	25.6419	25.3258	25.8689	26.006	25.6377	24.6583	25.2033	NaN	25.362	25.9714	25.3197	Q9NYF8;Q9NYF8-2	Q9NYF8;Q9NYF8-2	Bcl-2-associated t	BCLAF1
24.6534	24.2141	24.2885	24.3183	24.2348	24.0786	24.0256	NaN	NaN	24.4204	23.9853	NaN	P11274;P11274-2,	P11274;P11274-2	Breakpoint cluste	BCR
24.3909	23.9852	24.3272	24.9685	24.4813	24.8043	NaN	NaN	NaN	NaN	NaN	NaN	Q02338;E9PCG9;H	Q02338;E9PCG9;H	D-beta-hydroxybu	BDH1
23.1163	23.8978	23.583	22.888	23.6479	23.8844	NaN	NaN	24.5974	24.2768	24.5953	NaN	Q9BUT1;Q9BUT1-	Q9BUT1;Q9BUT1-	3-hydroxybutyrate	BDH2
24.8946	22.6159	23.2128	23.6109	22.8144	22.754	NaN	NaN	NaN	23.2711	22.4194	NaN	Q14457;K7ELY9;E	Q14457;K7ELY9;E	Beclin-1	BECN1
NaN	NaN		21.1263	NaN	NaN	NaN	NaN	NaN	NaN	NaN	NaN	O15155;H7C1N3;(	O15155;H7C1N3;(	BET1 homolog	BET1;DKFZp781C0
NaN	NaN	NaN	NaN	NaN	NaN	21.3032	NaN	NaN	NaN	NaN	NaN	Q96G01;Q96G01-	Q96G01;Q96G01-	Protein bicaudal D	BICD1
25.7436	25.6915	25.1603	25.0639	25.243	25.1748	26.2298	25.8387	24.6847	25.4739	24.7822	24.6552	Q8TD16;Q8TD16-2	Q8TD16;Q8TD16-2	Protein bicaudal D	BICD2
24.6742	24.7236	24.9546	24.8437	24.7323	25.038	24.4274	NaN	NaN	23.9848	23.1405	NaN	P55957;P55957-2,	P55957;P55957-2	BH3-interacting d	BID
24.1391	24.8578	24.5177	23.9398	24.2795	24.4122	25.1275	NaN	NaN	24.1553	NaN	25.1137	O00499-10;O0049	O00499-10;O0049	Myc box-depende	BIN1

25.053	25.3162	24.9122	24.8174	25.0599	24.8422	25.9469	25.4549	24.9754	25.0426	25.1016	24.9788	Q9NQY0;H7BYV6;	Q9NQY0;H7BYV6;	Bridging integrato	BIN3
NaN	22.7364	22.842	NaN	NaN	NaN	25.362	NaN	23.6369	NaN	NaN	NaN	O15392;A0A0B4J1	O15392;A0A0B4J1	Baculoviral IAP re	BIRC5
25.355	23.4276	24.4138	24.3431	23.086	24.0211	NaN	NaN	24.487	23.9634	23.9683	NaN	Q9NR09	Q9NR09	Baculoviral IAP re	BIRC6
25.7146	NaN	NaN	NaN	NaN	NaN	NaN	NaN	NaN	NaN	NaN	NaN	P54132;H0YNU5	P54132;H0YNU5	Bloom syndrome	BLM
25.3248	26.2503	25.7457	25.3651	26.2096	25.4632	25.4392	26.353	26.0034	25.2423	26.1797	25.9293	Q13867;K7ES02;J3	Q13867;K7ES02;J3	Bleomycin hydrol	BLMH
22.2947	22.6863	NaN	NaN	NaN	NaN	NaN	NaN	NaN	NaN	NaN	NaN	Q6QNY1;J3QRU7;	Q6QNY1;J3QRU7;	Biogenesis of lyso	BLOC1S2
NaN	NaN	NaN	NaN	22.877	NaN	NaN	NaN	NaN	NaN	NaN	NaN	Q6QNY0;K7EN58;	Q6QNY0;K7EN58	Biogenesis of lyso	BLOC1S3
25.8786	26.4339	26.293	25.6251	25.9965	26.2385	26.1885	25.3351	25.9611	25.913	25.376	25.7893	P53004;C9J1E1	P53004	Biliverdin reducta	BLVRA
27.0969	27.728	27.2722	27.2453	27.5281	27.6434	27.0763	27.0678	26.6133	26.914	27.1037	27.1843	P30043;M0R192;I	P30043;M0R192	Flavin reductase (	BLVRB
NaN	NaN	24.0921	NaN	NaN	23.0283	24.6339	NaN	NaN	23.2012	NaN	NaN	P13497;P13497-5	P13497;P13497-5	Bone morphogen	BMP1;TLL2
23.5195	NaN	23.1769	23.1982	NaN	22.9292	NaN	NaN	NaN	NaN	NaN	NaN	Q9NSY1;Q9NSY1-	Q9NSY1;Q9NSY1-	BMP-2-inducible	BMP2K
NaN	NaN	NaN	NaN	NaN	NaN	NaN	NaN	NaN	22.0678	NaN	NaN	P12644;H0YLW3	P12644;H0YLW3	Bone morphogen	BMP4
22.6099	22.593	22.6259	22.7319	22.394	NaN	NaN	NaN	NaN	23.8663	22.8941	NaN	P36894;O00238;C	P36894	Bone morphogen	BMPR1A
NaN	NaN	NaN	21.6567	NaN	NaN	NaN	NaN	NaN	NaN	NaN	NaN	Q13873;Q13873-2	Q13873;Q13873-2	Bone morphogen	BMPR2
23.1071	NaN	22.8797	24.0098	22.5923	NaN	NaN	NaN	NaN	22.3014	23.0402	NaN	Q14692	Q14692	Ribosome biogen	BMS1
NaN	NaN	21.7512	NaN	NaN	NaN	NaN	NaN	NaN	NaN	NaN	NaN	Q12981;Q12981-1	Q12981;Q12981-1	Vesicle transport	BNIP1
25.971	25.5744	25.4625	25.1783	24.4156	24.6701	NaN	NaN	NaN	24.7194	NaN	NaN	Q12983;F6RP06;A	Q12983;F6RP06;A	BCL2/adenovirus	BNIP3
NaN	NaN	NaN	22.2952	NaN	NaN	NaN	NaN	NaN	NaN	NaN	NaN	O60238;H0YBC7;A	O60238;H0YBC7;A	BCL2/adenovirus	BNIP3L
27.0964	27.6391	26.9626	26.6959	27.2603	26.9512	27.0473	26.9185	27.1161	26.5355	26.8954	27.3714	Q9H3K6;Q9H3K6-	Q9H3K6;Q9H3K6-	BolA-like protein 2	BOLA2;BOLA2B
24.1539	23.9993	24.4654	25.5169	25.1053	24.706	NaN	NaN	23.3119	24.2173	24.724	24.9523	Q14137;Q14137-2	Q14137;Q14137-2	Ribosome biogen	BOP1
25.1154	25.8275	25.461	24.8947	26.0835	25.5858	25.6143	25.1023	26.1933	25.3815	25.4802	25.8361	O95861;O95861-4	O95861;O95861-4	3(2),5-bisphospha	BPNT1
24.2093	NaN	NaN	NaN	NaN	NaN	NaN	NaN	NaN	NaN	NaN	NaN	Q7Z569;J3KNN7;C	Q7Z569;J3KNN7;C	BRCA1-associated	BRAP
23.6662	NaN	24.1736	24.431	23.7458	24.1679	23.2011	NaN	NaN	24.2389	24.6572	24.3075	Q6PJG6;Q6PJG6-2	Q6PJG6	BRCA1-associated	BRAT1
25.226	25.5606	25.1524	25.3694	25.6364	25.6788	25.1854	25.8066	26.0795	25.7002	25.7398	NaN	P46736-2;P46736	P46736-2;P46736	Lys-63-specific de	BRCC3
23.5141	22.4235	22.7665	23.4514	22.811	23.0722	22.4868	NaN	22.1758	23.1996	23.5595	NaN	O60885;O60885-2	O60885;O60885-2	Bromodomain-co	BRD4
24.3955	23.8919	24.2148	24.3013	24.2492	24.5722	23.8717	24.4707	23.9085	24.3894	23.8183	NaN	Q9NXR7;Q9NXR7-	Q9NXR7;Q9NXR7-	BRCA1-A complex	BRE
28.2957	26.1474	27.0849	28.5462	26.5534	26.7511	24.3191	22.5426	24.6364	25.6352	25.4966	24.0742	Q8TDN6	Q8TDN6	Ribosome biogen	BRIX1
25.7264	26.7244	26.4734	25.6494	26.7378	26.1977	26.8168	27.0115	26.9821	26.1954	27.0941	27.4756	Q8WUW1;Q8WU	Q8WUW1;Q8WU	Protein BRICK1	BRK1
NaN	NaN	21.3483	22.1725	NaN	NaN	NaN	NaN	NaN	NaN	NaN	NaN	Q5PSV4;H0YHD0;	Q5PSV4;H0YHD0;	Breast cancer met	BRMS1L
26.8208	26.0519	26.7166	26.4839	25.3271	26.3715	27.0002	24.6889	25.8167	26.1888	25.0913	25.7161	Q5VW32;Q5VW3	Q5VW32;Q5VW3	BRO1 domain-con	BROX
NaN	NaN	NaN	23.0837	NaN	NaN	NaN	NaN	NaN	NaN	NaN	NaN	Q9ULD4;E9PI60;E	Q9ULD4;E9PI60;E	Bromodomain an	BRPF3
31.2324	30.0594	30.4679	30.6385	29.7611	30.3962	30.5315	29.1514	29.0045	31.1786	30.0849	29.4667	P35613;P35613-2	P35613;P35613-2	Basigin	BSG
25.6386	24.2966	25.939	25.7547	25.079	26.4978	25.8565	NaN	25.303	26.8344	25.6943	25.1762	Q10589;Q10589-2	Q10589;Q10589-2	Bone marrow stro	BST2
25.7027	24.2109	24.8545	25.7652	24.7371	24.9085	NaN	NaN	22.5862	25.1013	24.9474	24.2364	O14981;A0A0A0N	O14981;A0A0A0N	TATA-binding prot	BTAF1
23.4357	NaN	NaN	NaN	NaN	NaN	NaN	NaN	NaN	NaN	NaN	NaN	Q9BSF8;E9PKU9;E	Q9BSF8;E9PKU9;E	BTB/POZ domain-	BTBD10
NaN	NaN	NaN	23.0018	NaN	NaN	NaN	NaN	NaN	NaN	NaN	NaN	P20290;H0Y9Y1	P20290	Transcription fact	BTF3
27.9836	28.1982	28.2787	28.2232	28.3484	28.0723	27.5042	28.9309	28.0309	28.1791	28.4838	28.7474	P20290-2;D6RDG	P20290-2;D6RDG	Transcription fact	BTF3
25.6969	25.9706	25.9071	25.7719	26.3077	25.7509	25.5694	26.4073	25.8181	25.3126	26.2676	26.412	Q96K17;E9PL10;C	Q96K17;E9PL10	Transcription fact	BTF3L4
27.2208	26.9529	28.2468	27.8642	27.7157	28.4203	27.0041	27.1663	26.5409	28.4037	27.7161	27.7981	Q7KYR7;Q7KYR7-5	Q7KYR7;Q7KYR7-5	Butyrophilin subfa	BTN2A1
NaN	23.2272	24.3031	23.7957	23.4522	24.686	NaN	NaN	NaN	23.4326	23.5907	NaN	O00478;O00478-2	O00478;O00478-2	Butyrophilin subfa	BTN3A3;BTN3A1
29.0883	28.3324	28.5313	28.6255	28.2952	28.0772	27.6444	27.7972	27.8485	29.2403	29.4395	28.2969	O43684;J3QT28;C	O43684;J3QT28;C	Mitotic checkpoin	BUB3
21.0964	20.8866	20.814	20.7967	NaN	NaN	NaN	NaN	NaN	21.4644	NaN	NaN	P41223;P41223-2	P41223;P41223-2	Protein BUD31 ho	BUD31
25.6676	22.3794	23.8946	24.6264	22.8494	23.2964	22.3625	21.9908	NaN	23.4459	22.1081	22.3475	Q13895;H7BY94;F	Q13895	Bystin	BYSL
28.2809	27.3342	27.8241	28.1591	27.2343	27.8234	28.0048	27.5358	27.4943	28.0375	27.8272	27.8311	Q7L1Q6;Q7L1Q6-	Q7L1Q6;Q7L1Q6-	Basic leucine zipp	BZW1
23.6085	23.8386	23.9674	24.1742	NaN	23.9113	24.0862	NaN	NaN	24.1444	24.1172	24.173	Q9Y6E2;E7ETZ4;B	Q9Y6E2;E7ETZ4;B	Basic leucine zipp	BZW2
NaN	NaN	NaN	NaN	NaN	NaN	NaN	NaN	NaN	NaN	21.9413	NaN	Q96B45;A0A0B4J	Q96B45;A0A0B4J	UPF0693 protein	C10orf32;C10orf3
NaN	NaN	NaN	22.1575	NaN	NaN	NaN	NaN	NaN	NaN	NaN	NaN	Q96D05;Q96D05-	Q96D05;Q96D05-	Uncharacterized p	C10orf35
23.4897	22.9286	23.665	23.3562	22.737	23.2908	24.1571	NaN	NaN	23.6439	NaN	NaN	Q5T2E6;Q5T2E6-2	Q5T2E6;Q5T2E6-2	UPF0668 protein	C10orf76
25.0961	26.7589	25.6901	24.8977	26.429	25.9919	25.1825	25.6305	25.6866	25.1988	25.4033	25.4526	Q9H0W9;A0A087	Q9H0W9;A0A087	Ester hydrolase C	C11orf54
NaN	NaN	NaN	22.4058	NaN	NaN	NaN	NaN	NaN	23.0122	23.5074	NaN	Q9H3H3;Q9H3H3	Q9H3H3;Q9H3H3	UPF0696 protein	C11orf68
NaN	NaN	NaN	22.2215	NaN	NaN	NaN	NaN	NaN	NaN	22.9403	NaN	A0A0B4J220;E9PR	A0A0B4J220;E9PR	Uncharacterized p	C11orf98
26.915	27.2756	26.668	26.6461	27.1561	26.8179	26.805	27.1768	26.9646	27.1097	27.5354	27.1293	Q9HB07;F8VR84;F	Q9HB07;F8VR84;F	UPF0160 protein	C12orf10
NaN	NaN	NaN	NaN	NaN	NaN	24.0815	NaN	NaN	NaN	NaN	NaN	Q9H741;F8VVN2	Q9H741;F8VVN2	UPF0454 protein	C12orf49
23.6336	24.7146	24.0762	23.6668	24.2302	NaN	24.237	NaN	23.7056	NaN	23.5192	23.816	Q99622;U3KQ85;I	Q99622;U3KQ85;I	Protein C10	C12orf57
NaN	NaN	NaN	NaN	NaN	NaN	21.5998	NaN	NaN	NaN	NaN	NaN	Q9UKR5	Q9UKR5	Probable ergoster	C14orf1
NaN	NaN	NaN	22.3992	NaN	NaN	NaN	NaN	NaN	NaN	NaN	NaN	Q9NWQ9	Q9NWQ9	Uncharacterized p	C14orf119
NaN	NaN	NaN	NaN	NaN	NaN	NaN	NaN	NaN	NaN	20.1824	NaN	Q9BXV9	Q9BXV9	Uncharacterized p	C14orf142
26.6889	25.6555	25.4138	25.819	25.0735	25.3676	25.9295	NaN	NaN	25.9018	25.1694	NaN	Q9Y224;H0YJB9;G	Q9Y224;H0YJB9;G	UPF0568 protein	C14orf166
24.5691	23.9987	23.6171	24.085	23.7458	NaN	NaN	NaN	NaN	NaN	NaN	NaN	Q6ZUT6;Q6ZUT6-	Q6ZUT6;Q6ZUT6-	Uncharacterized p	C15orf52
22.0289	NaN	23.8044	22.9977	22.8843	NaN	24.0432	NaN	NaN	23.1604	NaN	NaN	Q96S19;A0A087W	Q96S19;A0A087W	UPF0585 protein	C16orf13

	24.5929	24.1352	23.7648	24.5667	24.2619	23.8377	23.2227	NaN	23.8283	24.2691	24.0039	NaN	Q9HAS0;J9JIC5;J3	Q9HAS0;J9JIC5	Protein Njmu-R1	C17orf75
NaN	NaN	NaN	NaN	22.8208	NaN	NaN	NaN	NaN	NaN	NaN	NaN	Q53F19	Q53F19	Uncharacterized p	C17orf85
23.5032	NaN	22.5026	22.8918	NaN	NaN	22.9917	NaN	NaN	22.939	NaN	NaN	Q96DM3;K7ENL9;	Q96DM3;K7ENL9;	Uncharacterized p	C18orf8
NaN	23.9054	NaN	23.3619	23.6296	23.2846	NaN	NaN	NaN	NaN	23.6532	NaN	Q9UFG5;E7EP72;I	Q9UFG5;E7EP72	UPF0449 protein (	C19orf25
22.492	23.6788	23.1522	22.6622	24.0067	23.0042	23.3084	24.8407	NaN	23.2931	24.1878	NaN	Q9BQ61;K7ELS0;K	Q9BQ61	Uncharacterized p	C19orf43
NaN	NaN	NaN	NaN	NaN	NaN	25.9046	NaN	NaN	NaN	NaN	NaN	Q13901;J3KPZ4	Q13901;J3KPZ4	Nuclear nucleic ac	C1D
21.5228	NaN	NaN	21.4235	NaN	NaN	NaN	NaN	NaN	NaN	NaN	NaN	Q9NS00;Q9NS00-	Q9NS00;Q9NS00-	Glycoprotein-N-ac	C1GALT1
NaN	NaN	NaN	NaN	NaN	NaN	24.4223	NaN	NaN	NaN	NaN	NaN	Q96EU7	Q96EU7	C1GALT1-specific	C1GALT1C1
23.8297	23.4869	23.2212	23.6823	23.8228	23.6155	NaN	NaN	NaN	NaN	NaN	NaN	Q9NX04;V9GYI2;V	Q9NX04;V9GYI2;V	Uncharacterized p	C1orf109
NaN	NaN	NaN	NaN	NaN	NaN	NaN	NaN	NaN	NaN	NaN	NaN	Q6ZSJ8	Q6ZSJ8	Uncharacterized p	C1orf122
24.4956	25.1398	24.7908	24.6319	25.228	24.7773	25.0922	25.4311	25.1433	24.2724	25.0352	25.8712	Q9NWV4	Q9NWV4	UPF0587 protein (	C1orf123
NaN	NaN	NaN	NaN	NaN	NaN	NaN	20.7937	NaN	NaN	NaN	NaN	Q8IYL3	Q8IYL3	UPF0688 protein (	C1orf174
NaN	NaN	NaN	21.3482	NaN	NaN	NaN	NaN	NaN	NaN	NaN	NaN	Q9H425;E5RI90;Q	Q9H425;E5RI90;Q	Uncharacterized p	C1orf198
23.4311	22.9273	23.6781	23.0138	23.4029	24.0196	NaN	NaN	NaN	24.7129	23.6118	NaN	Q9H246	Q9H246	Uncharacterized p	C1orf21
NaN	NaN	NaN	20.1413	NaN	NaN	NaN	NaN	NaN	NaN	NaN	NaN	A1L170;A1L170-2;	A1L170;A1L170-2;	Uncharacterized p	C1orf226
NaN	NaN	NaN	NaN	NaN	NaN	NaN	NaN	30.4156	NaN	NaN	NaN	Q6PIY5;C9J579;C9	Q6PIY5;C9J579;C9	Uncharacterized p	C1orf228
25.021	25.6024	24.4646	24.6481	24.9886	24.3521	NaN	25.8798	25.8361	24.3765	25.1922	24.4214	Q07021;I3L3Q7;I3	Q07021;I3L3Q7;I3	Complement com	C1QBP
NaN	NaN	NaN	24.0049	NaN	NaN	NaN	NaN	NaN	NaN	NaN	NaN	Q9BUV8;Q9BUV8	Q9BUV8;Q9BUV8	Uncharacterized p	C20orf24
23.7388	23.3074	23.0955	23.5231	NaN	NaN	NaN	NaN	NaN	23.572	NaN	NaN	Q9GZN8;Q9GZN8	Q9GZN8;Q9GZN8	UPF0687 protein (	C20orf27
23.3492	24.2665	23.508	22.9593	24.3759	23.7894	NaN	23.478	24.1707	22.6814	NaN	23.3358	A0A096LPI6;P300	A0A096LPI6;P300	ES1 protein homo	C21orf33
22.9514	23.578	22.9145	23.0194	22.9632	NaN	23.1921	NaN	NaN	23.0609	NaN	NaN	P57076;H7C2R6;C	P57076;H7C2R6;C	UPF0769 protein (	C21orf59
25.3047	24.304	24.9623	25.3677	24.5582	25.062	25.0304	NaN	24.3305	25.2261	25.3356	24.5804	Q86YS7;Q86YS7-3	Q86YS7;Q86YS7-3	C2 domain-contai	C2CD5
NaN	NaN	NaN	NaN	NaN	NaN	NaN	NaN	NaN	NaN	NaN	20.6895	Q8WWC4	Q8WWC4	Uncharacterized p	C2orf47
NaN	22.5069	22.9013	NaN	22.22	NaN	NaN	NaN	22.8697	NaN	NaN	NaN	A6NCS6	A6NCS6	Uncharacterized p	C2orf72
NaN	22.0276	NaN	22.0539	21.9591	NaN	NaN	NaN	NaN	NaN	NaN	NaN	P01024;M0QXZ3	P01024	Complement C3;C	C3
NaN	NaN	NaN	20.499	NaN	NaN	NaN	NaN	NaN	NaN	NaN	NaN	Q9NWY4	Q9NWY4	UPF0609 protein (	C4orf27
NaN	NaN	NaN	21.6432	NaN	NaN	NaN	NaN	NaN	NaN	NaN	NaN	A6NDU8;A0A0C4I	A6NDU8;A0A0C4I	UPF0600 protein (	C5orf51
NaN	NaN	NaN	22.3078	NaN	NaN	NaN	NaN	NaN	NaN	NaN	NaN	Q7Z4R8	Q7Z4R8	UPF0669 protein (	C6orf120
23.3508	NaN	NaN	22.9456	NaN	NaN	NaN	NaN	NaN	NaN	NaN	NaN	Q9BRJ6;C9JQV0;H	Q9BRJ6;C9JQV0;H	Uncharacterized p	C7orf50
23.6607	23.3604	23.1561	23.9077	23.6299	23.0299	NaN	NaN	NaN	22.9899	NaN	NaN	Q9H7E9;Q9H7E9-	Q9H7E9;Q9H7E9-	UPF0488 protein (	C8orf33
23.9464	22.9346	23.3585	24.2674	23.1141	23.2334	NaN	NaN	NaN	22.6458	NaN	22.6387	Q5T280;A0A087W	Q5T280;A0A087W	Putative methyltr	C9orf114
NaN	NaN	23.1399	22.7611	NaN	NaN	NaN	NaN	NaN	NaN	NaN	NaN	Q9BUH6	Q9BUH6	Protein PAXX	C9orf142
24.2434	25.1207	24.4923	23.9514	25.2288	24.6955	25.2792	25.0503	25.0325	24.5015	24.9601	25.5528	Q5T6V5;Q5T6V7;(	Q5T6V5	UPF0553 protein (	C9orf64
21.6889	NaN	NaN	NaN	NaN	NaN	NaN	NaN	NaN	NaN	NaN	NaN	Q96LT7	Q96LT7	Protein C9orf72	C9orf72
NaN	NaN	NaN	20.2162	NaN	NaN	NaN	NaN	NaN	NaN	NaN	NaN	Q9H8G2	Q9H8G2	Caspase activity a	CAAP1
26.029	25.9088	26.0596	25.9391	25.3838	25.8954	26.0374	NaN	NaN	26.1006	25.614	NaN	Q9Y376;A0A087X	Q9Y376;A0A087X	Calcium-binding p	CAB39
22.8791	24.1988	24.0256	23.6545	23.636	24.4803	24.1481	NaN	24.2749	24.0106	23.4297	24.3102	P54289;P54289-4	P54289;P54289-4	Voltage-depender	CACNA2D1
22.2187	NaN	NaN	NaN	NaN	NaN	NaN	NaN	NaN	NaN	NaN	NaN	Q86Y37;Q86Y37-2	Q86Y37;Q86Y37-2	CDK2-associated a	CACUL1
29.6755	29.2384	29.2498	29.6052	29.4474	29.089	29.2236	29.0493	28.624	29.4893	29.1977	28.8838	Q9HB71;Q9HB71-	Q9HB71;Q9HB71-	Calcyclin-binding p	CACYBP
31.2707	30.0835	30.6054	31.3652	30.4788	30.6327	30.4116	30.9334	30.4269	31.0373	30.9003	30.6114	P27708;F8VPD4;H	P27708;F8VPD4	CAD protein;Gluta	CAD
NaN	NaN	NaN	NaN	NaN	NaN	NaN	NaN	NaN	NaN	NaN	24.2619	Q86UW7;Q86UW	Q86UW7;Q86UW	Calcium-depender	CADPS2
NaN	22.9695	22.117	NaN	NaN	NaN	NaN	NaN	NaN	22.6106	22.7449	23.0761	P22676;H3BN14	P22676;H3BN14	Calretinin	CALB2
25.7388	24.749	26.0967	24.1919	23.6747	24.4321	25.7671	25.5462	24.5616	23.5284	NaN	23.7227	Q9P1Z2;Q9P1Z2-2	Q9P1Z2;Q9P1Z2-2	Calcium-binding a	CALCOCO1
28.5852	26.2467	27.3268	26.1382	24.5277	25.9061	26.8872	23.2847	23.202	25.6188	24.5178	NaN	Q13137-4;Q13137	Q13137-4;Q13137	Calcium-binding a	CALCOCO2
27.1987	27.5716	27.1504	26.8121	26.8992	27.0358	27.1386	25.0535	25.6794	26.3132	26.5615	26.3949	Q05682-4;E9PGZ1	Q05682-4;E9PGZ1	Caldesmon	CALD1
25.4992	25.2335	24.7895	24.0259	NaN	24.2231	24.6092	NaN	NaN	25.1807	NaN	NaN	P62158;H0Y7A7;F	P62158;H0Y7A7;F	Calmodulin	CALM1;CALM2;CA
30.8882	30.5504	30.9579	30.6816	30.5182	31.0307	31.5023	31.9725	32.0363	31.339	31.1219	31.0214	P27797;K7EJB9;K7	P27797;K7EJB9	Calreticulin	CALR
27.9328	28.705	28.2297	27.9821	28.3792	28.0798	29.4118	29.3231	29.1962	28.6839	28.385	28.1482	O43852;O43852-3	O43852;O43852-3	Calumenin	CALU
22.8785	23.5748	NaN	22.592	NaN	22.3695	23.7532	23.7093	NaN	22.7151	22.9418	NaN	O43852-4;O43852	O43852-4;O43852	Calumenin	CALU
23.0644	NaN	22.8297	23.1921	22.1972	22.823	23.8526	NaN	22.6124	23.8562	23.2637	22.6858	Q14012;C9JES6;H	Q14012;C9JES6	Calcium/calmodul	CAMK1
27.2614	26.9889	27.021	27.2197	27.4097	27.1552	26.6409	27.2665	27.0592	27.0522	26.9024	27.0544	Q13557;Q13557-8	Q13557;Q13557-8	Calcium/calmodul	CAMK2D
26.0069	25.8449	25.7115	25.9007	26.115	25.9422	24.9977	25.4132	25.677	25.2929	25.5562	24.7087	Q13555;Q13555-5	Q13555;Q13555-5	Calcium/calmodul	CAMK2G;CAMK2B
NaN	NaN	NaN	21.2435	NaN	NaN	NaN	NaN	NaN	NaN	NaN	NaN	Q96RR4;Q96RR4-	Q96RR4;Q96RR4-	Calcium/calmodul	CAMKK2
28.0656	27.4573	27.9719	27.5923	27.4845	27.9785	28.5466	28.0571	28.2763	28.6817	28.1675	28.259	Q86VP6;Q86VP6-	Q86VP6;Q86VP6-	Cullin-associated	CAND1
30.4343	29.8322	30.2175	30.4158	29.8749	30.3015	30.0282	29.9414	30.4144	30.0341	29.3873	29.6989	P27824;P27824-2	P27824;P27824-2	Calnexin	CANX
30.4043	31.116	30.67	30.1693	30.5998	30.5522	31.3557	30.8382	30.5969	31.0688	30.7607	30.7397	Q01518;Q01518-2	Q01518;Q01518-2	Adenylyl cyclase-a	CAP1
26.1804	26.6921	26.496	26.0683	26.3114	26.4806	26.8572	26.6479	NaN	26.8657	26.7505	26.6688	P40123;E9PDI2;A(	P40123;E9PDI2;A(	Adenylyl cyclase-a	CAP2
29.1433	29.1593	29.1913	28.7452	28.7967	29.0045	30.1084	28.5123	28.979	29.6667	29.1033	29.1696	P40121;P40121-2	P40121;P40121-2	Macrophage-capp	CAPG
27.7135	27.753	27.8351	27.5249	27.7067	27.957	28.3225	27.9981	27.861	28.2425	27.9487	28.0529	P07384;E9PRM1;I	P07384	Calpain-1 catalytic	CAPN1
29.2831	28.728	29.2862	29.249	28.5797	29.0498	29.9014	29.7479	29.5623	30.0839	29.8553	29.778	P17655;P17655-2	P17655;P17655-2	Calpain-2 catalytic	CAPN2

25.8357	26.2598	26.9602	26.3569	26.4053	26.9507	26.957	26.5318	27.7081	27.3835	26.7987	27.6214	O15484;A0A087X	O15484;A0A087X	Calpain-5	CAPN5
23.8449	23.1725	23.3316	23.3048	23.0059	23.0014	25.4979	NaN	25.6362	24.7199	24.2695	24.0047	Q9Y6W3;H7C1B7;	Q9Y6W3	Calpain-7	CAPN7
27.9942	27.6917	26.9196	27.6459	27.4413	25.8666	27.7262	27.607	27.5889	27.5728	27.4718	27.579	P04632;A0A0C4D	P04632;A0A0C4D	Calpain small sub	CAPNS1
28.6587	28.5298	28.5413	28.3399	28.1739	28.1917	27.6536	28.0654	27.1117	27.5064	27.9533	27.4533	Q14444;Q14444-2	Q14444;Q14444-2	Caprin-1	CAPRIN1
28.9591	30.0652	29.448	28.8332	29.8236	29.4052	29.8147	30.1545	29.8305	29.4739	29.5024	29.8975	P52907	P52907	F-actin-capping pr	CAPZA1
24.7663	25.9626	25.4424	24.8267	25.6137	25.5353	25.8275	25.9738	25.5803	25.382	25.6384	25.5086	P47755;F8W9N7;	P47755	F-actin-capping pr	CAPZA2
28.4315	29.354	29.0807	28.3662	29.1526	29.1076	29.3849	29.4884	29.2042	29.3589	29.3342	29.5456	P47756-2;B1AK87	P47756-2;B1AK87	F-actin-capping pr	CAPZB
22.2216	22.3917	NaN	21.5002	NaN	NaN	NaN	NaN	NaN	NaN	NaN	NaN	Q9Y2G2;Q9Y2G2-	Q9Y2G2;Q9Y2G2-	Caspase recruitm	CARD8
26.0062	26.5949	26.1298	25.8079	26.2408	26.1117	25.9692	26.5003	26.3961	25.1541	25.6717	26.4376	Q9Y2V2;H3BSW7;	Q9Y2V2;H3BSW7;	Calcium-regulated	CARHSP1
26.4506	26.0083	26.2225	26.3512	26.037	26.1762	26.1197	26.5235	26.3812	26.7046	26.7797	26.9076	Q86X55;Q86X55-1	Q86X55;Q86X55-1	Histone-arginine	CARM1
26.4098	27.0006	26.7253	26.1463	26.8693	26.934	26.4466	26.6123	26.7095	26.4002	26.2039	26.1776	P49589-3;P49589	P49589-3;P49589	Cysteine--tRNA lig	CARS
23.3489	22.8868	22.6363	23.1686	NaN	NaN	23.9518	NaN	NaN	NaN	NaN	NaN	Q6P4E1;Q6P4E1-2	Q6P4E1;Q6P4E1-2	Protein CASC4	CASC4
26.6075	26.704	27.686	26.7593	26.9351	27.7218	27.5452	26.6726	27.0714	28.073	27.1576	27.0904	O14936;O14936-3	O14936;O14936-3	Peripheral plasma	CASK
NaN	22.9154	23.4363	NaN	NaN	NaN	23.1692	NaN	NaN	22.7264	23.3388	NaN	Q8WXE0;Q8WXE0	Q8WXE0;Q8WXE0	Caskin-2	CASKIN2
25.3172	25.7772	25.8378	25.5789	25.5906	25.7395	25.583	24.8316	25.2027	25.5607	25.2517	25.5003	P42574;A8MVM1	P42574;A8MVM1	Caspase-3;Caspas	CASP3
26.1658	24.6915	25.5471	25.7662	25.7059	25.5246	24.0327	24.7936	NaN	24.0523	24.8088	NaN	P49662;A0A087W	P49662;A0A087W	Caspase-4;Caspas	CASP4
23.5447	24.4624	23.6669	23.4712	23.7672	NaN	24.1875	NaN	NaN	24.3655	24.0576	NaN	P55210;A0A0A0M	P55210;A0A0A0M	Caspase-7;Caspas	CASP7
26.6349	24.926	25.3062	26.5439	24.8601	25.2001	25.2924	NaN	NaN	25.5232	25.225	NaN	Q14790;Q14790-4	Q14790;Q14790-4	Caspase-8;Caspas	CASP8
26.5841	27.2457	26.9024	25.939	27.3748	26.6894	27.2656	27.8389	27.2302	27.0197	27.3179	27.941	P20810-5;P20810	P20810-5;P20810	Calpastatin	CAST
26.5041	27.7856	27.5882	26.8604	27.6815	27.6663	27.1687	27.9389	27.9067	27.0049	27.1765	27.415	P04040	P04040	Catalase	CAT
30.2545	29.3255	30.2437	30.8198	29.5224	30.5017	29.78	28.674	28.5382	30.038	29.3215	28.9054	Q03135;E9PCT5;P	Q03135;E9PCT5	Caveolin-1;Caveol	CAV1
26.1384	NaN	26.4534	27.4172	26.0794	26.9255	26.3072	NaN	26.2133	26.9882	26.421	NaN	Q03135-2;C9JKI3	Q03135-2;C9JKI3	Caveolin-1;Caveol	CAV1
23.965	NaN	NaN	NaN	23.2724	NaN	NaN	NaN	NaN	NaN	NaN	NaN	P51636;E9PCT3;P	P51636;E9PCT3;P	Caveolin-2;Caveol	CAV2
25.329	25.0542	25.3338	24.9435	24.8497	25.1301	25.6684	25.9903	25.5756	26.1613	25.6757	25.9237	Q13951-2;Q13951	Q13951-2;Q13951	Core-binding facto	CBFB
24.489	23.6406	24.0402	24.7289	23.573	23.1007	NaN	NaN	NaN	23.4068	NaN	23.066	P22681;A0A0U1R	P22681;A0A0U1R	E3 ubiquitin-prote	CBL
28.0343	28.2185	27.8224	27.7407	28.0486	27.9015	28.2561	27.7808	27.6307	28.2378	28.2346	28.0426	P16152;E9PQ63;A	P16152;E9PQ63;A	Carbonyl reducta	CBR1
25.4377	26.277	25.8054	25.2638	26.3163	26.0493	25.8218	25.5737	26.0791	25.5317	25.9527	26.5437	O75828	O75828	Carbonyl reducta	CBR3
21.0683	NaN	NaN	NaN	NaN	NaN	NaN	NaN	NaN	NaN	NaN	NaN	Q9BRT8;Q8IUF1;C	Q9BRT8;Q8IUF1;C	COBW domain-co	CBWD1;CBWD2;C
23.2485	23.297	23.2935	23.2249	23.8698	NaN	NaN	NaN	NaN	24.104	24.233	23.877	P83916;B5MD17;	P83916;B5MD17;	Chromobox prote	CBX1
26.3889	26.3197	26.2517	26.5382	26.0224	26.4116	26.559	25.8976	25.3427	27.0906	26.712	27.0648	Q13185;B8ZZ43;C	Q13185	Chromobox prote	CBX3
23.9416	NaN	NaN	22.8691	NaN	NaN	23.2893	NaN	NaN	23.5945	24.3191	NaN	P45973;F8VNY3	P45973	Chromobox prote	CBX5
25.2901	24.9058	25.3544	25.0269	24.9299	25.4091	24.5251	25.1027	24.8629	24.3833	24.1787	25.0309	Q6P1N0;Q6P1N0-	Q6P1N0;Q6P1N0-	Coiled-coil and C2	CC2D1A
22.984	23.5013	23.0656	22.9164	23.6971	NaN	NaN	NaN	24.0843	23.8334	23.6271	24.0771	H7C1U3;Q5T0F9-2	H7C1U3;Q5T0F9-2	Coiled-coil and C2	CC2D1B
27.2879	25.6264	26.6342	27.1519	26.5743	26.7909	24.5078	24.8826	22.913	26.4955	26.5929	25.5572	Q8IX12;Q8IX12-2;	Q8IX12;Q8IX12-2;	Cell division cycle	CCAR1
27.095	26.3049	26.6995	26.8311	26.5341	26.7981	25.4816	26.0644	25.8391	26.8917	27.0022	26.8557	Q8N163;Q8N163-	Q8N163;Q8N163-	Cell cycle and apo	CCAR2
21.0092	21.3194	21.1718	NaN	21.3269	NaN	NaN	NaN	NaN	NaN	NaN	NaN	Q16773;B7Z4W5;	Q16773;B7Z4W5;	Kynurenine--oxog	CCBL1
26.1078	26.1313	25.4289	25.5301	26.0791	25.3878	25.9424	25.6731	26.0762	26.018	26.0728	26.068	Q6YP21-3;Q6YP21	Q6YP21-3;Q6YP21	Kynurenine--oxog	CCBL2
25.8527	27.1403	26.5116	25.0659	27.0102	25.4442	26.2621	26.8321	26.3136	25.1595	25.8236	25.9453	Q96CT7;M0R2F5	Q96CT7;M0R2F5	Coiled-coil domai	CCDC124
23.4373	NaN	22.5193	23.0094	21.6708	NaN	NaN	NaN	NaN	23.2549	NaN	NaN	Q96JG6;Q96JG6-3	Q96JG6;Q96JG6-3	Coiled-coil domai	CCDC132
NaN	24.323	NaN	NaN	24.4778	NaN	NaN	NaN	NaN	NaN	NaN	NaN	Q8IYT3	Q8IYT3	Coiled-coil domai	CCDC170
23.7445	23.5505	23.6154	23.5024	23.8375	23.8121	NaN	NaN	NaN	24.0159	23.9733	NaN	O60826	O60826	Coiled-coil domai	CCDC22
22.673	23.9883	23.532	22.6854	23.9056	23.6744	22.9354	NaN	NaN	23.2523	23.5091	23.6174	Q86WR0;Q86WR(	Q86WR0;Q86WR(	Coiled-coil domai	CCDC25
25.9657	25.2046	25.6761	26.0782	25.2318	25.7353	25.1609	NaN	25.7442	25.4782	25.5838	24.3436	Q96A33;Q96A33-1	Q96A33;Q96A33-1	Coiled-coil domai	CCDC47
25.5873	26.6598	26.3641	25.2917	26.434	26.212	25.6016	NaN	25.1045	25.2227	24.706	NaN	Q8IVM0;Q8IVM0-	Q8IVM0;Q8IVM0-	Coiled-coil domai	CCDC50
22.345	22.525	22.2794	22.4665	22.5077	NaN	NaN	NaN	NaN	22.1459	NaN	NaN	Q9Y3C0;F5GWI9;I	Q9Y3C0;F5GWI9;I	WASH complex su	CCDC53
23.1947	23.927	22.8303	23.3133	23.2375	NaN	NaN	NaN	23.4836	23.2648	23.111	23.303	Q4VC31;C9JQ41;I	Q4VC31;C9JQ41;I	Coiled-coil domai	CCDC58
23.4308	NaN	22.8966	23.3156	22.9559	NaN	NaN	NaN	NaN	23.4361	23.0266	NaN	Q9P031;F8VNY5	Q9P031;F8VNY5	Thyroid transcript	CCDC59
26.5802	27.1373	26.7402	26.5298	26.9714	26.6955	27.1104	27.7676	26.7004	26.4756	26.7801	26.9076	Q16204	Q16204	Coiled-coil domai	CCDC6
22.5744	NaN	NaN	NaN	NaN	NaN	NaN	NaN	23.4059	24.613	23.686	23.9633	A6NI79;H0YBY5	A6NI79	Coiled-coil domai	CCDC69
NaN	NaN	NaN	NaN	NaN	NaN	NaN	NaN	NaN	20.9449	NaN	NaN	Q9H0W5	Q9H0W5	Coiled-coil domai	CCDC8
NaN	NaN	NaN	NaN	NaN	NaN	22.1983	NaN	NaN	NaN	NaN	NaN	Q76M96;Q76M96	Q76M96;Q76M96	Coiled-coil domai	CCDC80
22.4085	NaN	22.3499	22.0872	NaN	NaN	NaN	NaN	NaN	NaN	NaN	NaN	Q9H6F5;H0YG79;I	Q9H6F5	Coiled-coil domai	CCDC86
24.5557	25.173	25.2422	24.535	25.1332	24.734	25.2372	24.2575	NaN	24.7192	24.3336	24.7257	Q3V6T2;Q3V6T2-2	Q3V6T2;Q3V6T2-2	Girdin	CCDC88A
NaN	NaN	NaN	NaN	NaN	NaN	NaN	NaN	NaN	NaN	24.1986	NaN	Q9P219	Q9P219	Protein Daple	CCDC88C
NaN	NaN	NaN	NaN	NaN	NaN	24.9032	NaN	NaN	NaN	NaN	NaN	E9PKQ5	E9PKQ5		CCDC90B
23.4491	NaN	23.3664	23.2199	NaN	NaN	NaN	NaN	NaN	23.3348	23.3914	NaN	Q567U6;F8W9X7;	Q567U6;F8W9X7	Coiled-coil domai	CCDC93
NaN	NaN	NaN	20.4059	NaN	NaN	NaN	NaN	NaN	NaN	NaN	NaN	Q96F63	Q96F63	Coiled-coil domai	CCDC97
NaN	NaN	20.6906	NaN	NaN	NaN	NaN	NaN	NaN	NaN	NaN	NaN	Q9BSQ5;Q9BSQ5-	Q9BSQ5;Q9BSQ5-	Cerebral cavernou	CCM2
25.0411	25.1261	25.3044	24.856	25.602	25.2577	24.8941	24.9074	25.6076	25.3121	25.3452	25.3993	P14635;E9PC90;P	P14635;E9PC90;P	G2/mitotic-specifi	CCNB1
NaN	NaN	NaN	NaN	NaN	NaN	NaN	NaN	NaN	22.7495	NaN	NaN	O95067;H1UBN3;	O95067;H1UBN3;	G2/mitotic-specifi	CCNB2;CCNB2V

NaN	NaN	21.2355	21.1333	NaN	NaN	NaN	NaN	NaN	NaN	NaN	NaN	O95273;O95273-2	O95273;O95273-2	Cyclin-D1-binding	CCNDBP1
22.9214	NaN	NaN	23.3589	NaN	22.8741	NaN	NaN	NaN	22.7735	23.0348	NaN	Q9UK58;C9JPL0;C	Q9UK58;C9JPL0;C	Cyclin-L1	CCNL1
21.6129	NaN	NaN	NaN	NaN	NaN	NaN	NaN	NaN	22.1726	22.107	NaN	O60563;O60563-2	O60563;O60563-2	Cyclin-T1	CCNT1
24.3216	24.8233	25.4706	24.742	25.051	25.7862	24.2517	23.6873	NaN	25.3017	24.9715	24.8389	Q8ND76;Q8ND76	Q8ND76;Q8ND76	Cyclin-Y	CCNY
25.0149	24.6798	25.3415	24.9323	24.9908	25.6121	24.7204	NaN	NaN	25.7589	24.8735	25.0162	Q8N7R7;Q8N7R7-	Q8N7R7;Q8N7R7-	Cyclin-Y-like prote	CCNYL1
22.8032	NaN	NaN	21.8538	NaN	NaN	NaN	NaN	NaN	22.3336	NaN	NaN	Q9ULG6;Q9ULG6-	Q9ULG6;Q9ULG6-	Cell cycle progres	CCPG1
24.9849	25.4482	26.4961	25.733	26.1173	26.759	25.5501	NaN	NaN	26.6415	25.8498	NaN	O00421;O00421-2	O00421;O00421-2	C-C chemokine re	CCRL2
23.5271	24.4544	24.293	23.7325	24.5855	NaN	24.743	24.6363	24.4606	24.5396	24.545	24.9885	O14618;J3KNF4;E	O14618;J3KNF4;E	Copper chaperone	CCS
32.5599	32.5009	32.3835	32.5788	32.6761	32.4589	32.3591	32.826	32.2804	32.518	32.6768	32.5274	P78371;P78371-2	P78371;P78371-2	T-complex protein	CCT2
32.8075	32.4915	32.4635	32.8342	32.8503	32.6197	32.4132	32.8991	32.5202	32.6869	32.7367	32.658	P49368;P49368-2	P49368;P49368-2	T-complex protein	CCT3
32.1534	31.8323	31.7809	32.1633	32.2105	31.8602	31.7287	32.22	31.6699	31.9113	32.0097	31.9969	P50991;P50991-2	P50991;P50991-2	T-complex protein	CCT4
31.9753	31.9155	31.7425	32.073	32.0801	31.9655	31.8361	32.25	31.6842	32.1129	32.0838	32.1182	P48643;B7ZAR1;E	P48643;B7ZAR1;E	T-complex protein	CCT5
31.7393	31.8687	31.6841	31.9564	32.1213	31.773	31.7019	32.3011	31.7521	31.9362	32.0433	31.8122	P40227;P40227-2	P40227;P40227-2	T-complex protein	CCT6A
NaN	NaN	NaN	NaN	NaN	NaN	26.185	NaN	NaN	NaN	NaN	NaN	Q92526;Q92526-2	Q92526;Q92526-2	T-complex protein	CCT6B
31.6269	31.5044	31.5155	31.7893	31.8887	31.7725	31.1475	31.7439	31.4248	31.6761	32.0002	31.7916	Q99832;Q99832-3	Q99832;Q99832-3	T-complex protein	CCT7
32.3506	31.7904	31.8603	32.3137	32.1421	32.0322	31.7991	32.3416	31.7635	32.0954	32.1007	32.042	P50990;P50990-2	P50990;P50990-2	T-complex protein	CCT8
24.6042	25.1633	24.2874	24.5735	24.6342	24.4209	24.5614	NaN	NaN	23.9271	24.5808	NaN	P86791;P86790;F	P86791;P86790	Vacuolar fusion p	CCZ1;CCZ1B
26.7768	26.9562	25.4274	26.4268	26.256	25.15	NaN	NaN	NaN	NaN	NaN	NaN	P48509;E9PMR4;E	P48509;E9PMR4;E	CD151 antigen;Te	CD151
22.5346	NaN	NaN	22.8626	NaN	NaN	NaN	NaN	22.8797	23.7095	23.0034	23.3169	P20273;P20273-5	P20273;P20273-5	B-cell receptor CD	CD22
26.4314	26.0651	26.065	26.3115	26.2344	26.4824	26.3767	26.3729	26.3909	25.9566	25.7582	26.4141	Q9NZQ7;Q9NZQ7	Q9NZQ7;Q9NZQ7	Programmed cell	CD274
26.601	26.3405	27.2704	27.0593	26.1863	27.3256	27.0124	NaN	25.2038	27.7084	26.8969	26.4429	Q5ZPR3;A0A0C4D	Q5ZPR3;A0A0C4D	CD276 antigen	CD276
25.9773	26.3217	25.8691	25.5856	25.9161	25.7821	26.6136	26.1765	26.5335	25.106	25.4511	25.439	Q9Y5K6	Q9Y5K6	CD2-associated p	CD2AP
23.2516	22.966	24.001	23.5456	23.3291	NaN	NaN	NaN	NaN	24.2508	24.3572	23.8878	O95400	O95400	CD2 antigen cytop	CD2BP2
23.3611	22.7746	22.6197	NaN	NaN	NaN	NaN	NaN	NaN	NaN	NaN	NaN	Q9NPF0;Q9NPF0-	Q9NPF0;Q9NPF0-	CD320 antigen	CD320
22.3942	22.4496	22.5064	22.7507	22.6229	NaN	NaN	NaN	NaN	NaN	NaN	NaN	O15446;O15446-2	O15446;O15446-2	DNA-directed RNA	CD3EAP
24.3859	24.9685	NaN	25.0841	NaN	NaN	NaN	NaN	NaN	NaN	NaN	NaN	P25942;A0A087X	P25942	Tumor necrosis fa	CD40
32.1172	32.3789	32.7159	32.2573	32.4302	33.1652	31.5549	31.0087	31.0349	32.8042	32.0735	32.0927	P16070;P16070-1	P16070;P16070-1	CD44 antigen	CD44
26.9087	26.7344	27.0182	26.49	26.2692	26.9874	27.0885	26.4907	26.8076	27.8442	27.3179	26.6043	P15529;P15529-1	P15529;P15529-1	Membrane cofact	CD46
24.3155	24.8913	24.7941	24.4956	24.4087	NaN	NaN	NaN	25.369	25.8579	25.4076	25.3452	P15529-4;P15529	P15529-4;P15529	Membrane cofact	CD46
28.5277	27.3649	28.3091	28.6995	27.2583	28.4758	28.3421	26.9415	27.3927	28.7769	27.8828	27.8114	Q08722;Q08722-3	Q08722;Q08722-3	Leukocyte surface	CD47
28.4918	28.2698	25.8493	28.5368	27.3294	25.7146	26.4113	NaN	NaN	NaN	25.8014	26.3213	P08174;P08174-2	P08174;P08174-2	Complement deca	CD55
27.5301	27.2458	27.8209	27.6592	27.3004	28.34	27.8251	27.0592	27.1562	28.6773	27.6067	27.5424	P19256-3;P19256	P19256-3;P19256	Lymphocyte funct	CD58
31.1097	31.6791	28.7139	30.6969	31.0383	29.2511	29.9077	NaN	NaN	NaN	NaN	NaN	P13987;E9PNW4;	P13987;E9PNW4;	CD59 glycoprotei	CD59
26.8515	25.2193	26.3758	26.1011	25.2702	25.9633	27.2487	NaN	26.0967	27.1633	26.0686	25.5388	P08962;F8VNT9;F	P08962;F8VNT9;F	CD63 antigen;Tet	CD63
NaN	NaN	NaN	22.791	NaN	NaN	NaN	NaN	NaN	NaN	NaN	NaN	P34810;P34810-2	P34810;P34810-2	Macrosialin	CD68
26.3003	26.2608	26.9224	26.1107	26.2807	27.0246	27.2093	26.4408	28.1884	26.3241	25.9489	27.0318	P60033;E9PJK1;E	P60033;E9PJK1;E	CD81 antigen;Tet	CD81
25.0262	25.1682	26.3809	24.7058	25.8459	25.7801	NaN	NaN	NaN	NaN	25.038	25.2415	P27701;P27701-2	P27701;P27701-2	CD82 antigen	CD82
30.5838	29.558	30.2422	30.201	29.3298	30.2832	30.2347	28.8061	29.6756	30.5415	29.7312	29.6562	P21926;A6NNI4;G	P21926;A6NNI4;G	CD9 antigen;Tetra	CD9
NaN	NaN	NaN	NaN	NaN	NaN	NaN	NaN	22.9174	23.3226	23.0766	NaN	P40200;Q8WUE2;	P40200;Q8WUE2;	T-cell surface prot	CD96
30.7203	30.9373	30.9725	30.7352	30.5113	31.3325	30.6072	30.3368	30.7407	31.4987	31.1386	31.157	P48960;P48960-2	P48960;P48960-2	CD97 antigen;CD9	CD97
NaN	NaN	21.4172	NaN	NaN	NaN	NaN	NaN	NaN	21.6065	NaN	21.0596	P14209;A8MQT7;	P14209;A8MQT7;	CD99 antigen	CD99
NaN	NaN	NaN	24.571	NaN	NaN	NaN	NaN	NaN	NaN	NaN	NaN	Q8TCZ2;A0A0U1R	Q8TCZ2;A0A0U1R	CD99 antigen-like	CD99L2
25.3102	23.7142	24.3557	24.7121	NaN	24.1059	23.882	24.8928	NaN	24.0875	23.8718	23.7926	O75794;H7BZW7;	O75794;H7BZW7;	Cell division cycle	CDC123
25.8531	25.151	25.4397	25.965	25.4187	25.3961	NaN	NaN	NaN	25.5019	25.2578	NaN	Q13042;Q13042-3	Q13042;Q13042-3	Cell division cycle	CDC16
NaN	NaN	23.5416	NaN	NaN	23.638	NaN	NaN	NaN	NaN	NaN	NaN	Q12834	Q12834	Cell division cycle	CDC20
26.8531	24.9213	25.7171	27.0672	25.7476	25.5275	23.5665	24.1598	23.0382	25.2626	25.1918	24.2989	Q9UJX2;Q9UJX2-3	Q9UJX2;Q9UJX2-3	Cell division cycle	CDC23
22.9814	NaN	NaN	NaN	NaN	NaN	NaN	NaN	NaN	NaN	NaN	NaN	Q8NHZ8	Q8NHZ8	Anaphase-promot	CDC26
NaN	NaN	NaN	22.6238	NaN	NaN	NaN	NaN	NaN	NaN	NaN	NaN	G5EA36;I3L394;I3	G5EA36		CDC27
26.0932	24.9011	25.2118	26.2771	24.8962	24.8699	23.3176	23.8275	NaN	24.4855	24.1923	24.2241	P30260;P30260-2	P30260;P30260-2	Cell division cycle	CDC27
22.7494	22.5583	22.4804	22.5129	22.4018	22.5147	22.4922	NaN	22.9382	22.8286	22.6439	NaN	P49427;U3KQ77;U	P49427;U3KQ77;U	Ubiquitin-conjuga	CDC34
28.3478	28.3871	28.3124	28.0823	27.9974	28.2105	28.7457	28.2059	28.2131	28.1506	28.0651	28.4315	Q16543;K7EKQ2;K	Q16543;K7EKQ2;K	Hsp90 co-chaperc	CDC37
22.8767	NaN	21.6219	22.1201	NaN	21.6414	NaN	NaN	NaN	NaN	NaN	NaN	O60508;Q5SRN1	O60508;Q5SRN1	Pre-mRNA-proces	CDC40
29.0429	29.2735	29.7291	28.8646	29.3016	29.3423	29.4625	29.3364	30.2598	29.6171	29.5647	30.3511	P60953;P60953-1	P60953;P60953-1	Cell division contr	CDC42
24.1416	24.5082	24.3722	23.925	24.2053	24.6597	23.9667	NaN	NaN	24.6993	24.2741	24.7374	Q5VT25;Q5VT25-2	Q5VT25;Q5VT25-2	Serine/threonine-	CDC42BPA
26.4044	26.2349	26.6228	26.5113	26.1307	26.2454	25.1391	NaN	24.5082	25.7265	25.7413	23.6301	Q9Y5S2;H0YLY0;A	Q9Y5S2	Serine/threonine-	CDC42BPB
NaN	NaN	NaN	NaN	NaN	NaN	NaN	NaN	NaN	21.5094	NaN	NaN	Q9H3Q1;Q9H3Q1	Q9H3Q1;Q9H3Q1	Cdc42 effector pr	CDC42EP4
NaN	NaN	23.0303	NaN	NaN	NaN	NaN	NaN	NaN	NaN	NaN	NaN	Q9NRR8	Q9NRR8	CDC42 small effec	CDC42SE1
25.8708	26.2483	26.5188	25.7913	26.0656	26.5695	25.2082	NaN	NaN	25.2761	25.4376	25.7718	Q9NRR3;D6REL0	Q9NRR3;D6REL0	CDC42 small effec	CDC42SE2
25.5888	24.8584	24.9815	25.378	25.4004	24.98	23.6285	NaN	NaN	24.4163	25.1118	24.9991	Q99459	Q99459	Cell division cycle	CDC5L
NaN	NaN	NaN	21.4765	NaN	NaN	NaN	NaN	NaN	NaN	NaN	NaN	O00311;B1AMW7	O00311;B1AMW7	Cell division cycle	CDC7

21.8876	NaN	22.0359	21.8855	21.6922	NaN	NaN	NaN	NaN	NaN	21.3508	NaN	Q6P1J9	Q6P1J9	Parafibromin	CDC73
25.0699	26.2309	26.1573	25.2192	26.1999	26.3462	25.6944	25.9468	25.8012	26.1311	26.2907	27.0192	Q99618;F5GX58;F	Q99618;F5GX58;F	Cell division cycle-	CDCA3
NaN	NaN	NaN	22.2418	NaN	NaN	NaN	NaN	NaN	NaN	NaN	NaN	Q96FF9;B5MBX0	Q96FF9;B5MBX0	Sororin	CDCA5
23.4679	23.8385	23.8274	24.0042	23.7416	24.2348	26.032	25.4447	25.7288	23.2473	24.0411	22.8607	Q53HL2	Q53HL2	Borealin	CDCA8
29.7466	30.5096	30.4624	30.1438	30.235	30.6413	30.5752	30.2026	30.9275	31.2136	30.7969	30.9482	Q9H5V8;Q9H5V8-	Q9H5V8;Q9H5V8-	CUB domain-cont	CDCP1
NaN	NaN	NaN	NaN	NaN	NaN	NaN	21.6747	NaN	NaN	NaN	NaN	P55290;A0A087X(	P55290;A0A087X(	Cadherin-13	CDH13
24.6635	24.4366	24.4404	24.6986	24.32	24.7819	26.477	26.0889	24.482	25.9414	25.2933	25.1205	P55283;A0A087W	P55283;A0A087W	Cadherin-4	CDH4
29.5178	29.4428	29.4338	29.0957	29.4547	29.4065	29.3173	29.1455	29.3214	29.9905	30.9689	29.5578	P06493;A0A024Q	P06493;A0A024Q	Cyclin-dependent	CDK1;CDC2
25.4983	24.1168	24.4868	25.6091	24.1829	24.5336	NaN	NaN	NaN	25.55	25.3145	NaN	P21127;P21127-9	P21127;P21127-9	Cyclin-dependent	CDK11B;CDC2L1;C
23.7876	NaN	23.3725	24.5761	23.2611	23.499	NaN	NaN	NaN	23.625	23.4794	NaN	Q9NYV4;Q9NYV4-	Q9NYV4;Q9NYV4-	Cyclin-dependent	CDK12
22.4698	NaN	NaN	22.5905	NaN	NaN	NaN	NaN	NaN	NaN	NaN	NaN	Q14004;Q14004-2	Q14004;Q14004-2	Cyclin-dependent	CDK13
20.7095	NaN	NaN	NaN	NaN	NaN	NaN	NaN	NaN	NaN	NaN	NaN	Q00536;Q00536-2	Q00536;Q00536-2	Cyclin-dependent	CDK16
23.9796	23.7993	23.8141	23.4692	23.7394	24.1648	23.7123	NaN	NaN	24.2088	NaN	NaN	Q00537;Q00537-2	Q00537;Q00537-2	Cyclin-dependent	CDK17
24.2173	23.5893	24.0293	23.5608	NaN	23.9597	23.7351	NaN	NaN	24.255	24.4868	23.5767	P24941;G3V5T9;P	P24941;G3V5T9;P	Cyclin-dependent	CDK2
23.259	NaN	NaN	22.6915	NaN	NaN	NaN	NaN	NaN	NaN	NaN	NaN	P11802;F8VYH9;F	P11802;F8VYH9;F	Cyclin-dependent	CDK4
26.7019	25.7233	26.1629	26.3465	26.3647	26.2545	25.6168	25.6088	26.0066	26.3324	26.7178	26.0697	Q00535;Q00535-2	Q00535;Q00535-2	Cyclin-dependent	CDK5
25.5616	24.9747	24.1677	24.6368	25.2329	24.2263	22.3889	NaN	23.7995	24.4139	24.4427	24.0655	Q96JB5;Q96JB5-4	Q96JB5;Q96JB5-4	CDK5 regulatory s	CDK5RAP3
24.4252	23.777	24.1599	24.1746	23.6609	23.995	23.8151	NaN	24.1152	24.0358	24.1761	24.2745	Q00534	Q00534	Cyclin-dependent	CDK6
24.4328	24.4154	24.8019	24.7896	24.8977	24.9723	23.3487	NaN	25.6261	24.2679	24.7842	24.8565	P50613;D6R9G1;D	P50613;D6R9G1;D	Cyclin-dependent	CDK7
23.7013	NaN	22.7333	23.2751	NaN	NaN	NaN	NaN	NaN	23.507	23.3151	NaN	P50750;P50750-2	P50750;P50750-2	Cyclin-dependent	CDK9
NaN	NaN	NaN	NaN	NaN	NaN	NaN	NaN	NaN	21.061	20.8901	NaN	Q5VV42;Q5VV42-	Q5VV42;Q5VV42-	Threonylcarbamo	CDKAL1
NaN	NaN	NaN	21.9941	NaN	NaN	NaN	NaN	NaN	NaN	NaN	NaN	Q9NXV6;J3KNE1;D	Q9NXV6;J3KNE1;D	CDKN2A-interacti	CDKN2AIP
22.9664	23.9073	23.479	23.8287	24.0886	23.6005	NaN	NaN	NaN	24.2875	25.0756	24.1077	Q96HQ2-2;Q96H(	Q96HQ2-2;Q96H(	CDKN2AIP N-term	CDKN2AIPNL
25.7469	26.7928	25.9855	25.3318	26.3757	25.5541	25.4474	26.1543	25.7151	25.0029	25.4328	26.35	Q9UKY7;Q9UKY7-	Q9UKY7;Q9UKY7-	Protein CDV3 hon	CDV3
NaN	NaN	NaN	21.753	NaN	NaN	NaN	NaN	NaN	NaN	NaN	NaN	Q9Y232;Q9Y232-2	Q9Y232;Q9Y232-2	Chromodomain Y-	CDYL
22.2434	NaN	NaN	22.4968	NaN	NaN	NaN	NaN	NaN	NaN	NaN	NaN	P17676;P17676-2	P17676;P17676-2	CCAAT/enhancer-	CEBPB
24.025	NaN	23.2521	24.4587	23.2097	23.2237	NaN	NaN	NaN	NaN	NaN	NaN	Q03701;A0A087X	Q03701;A0A087X	CCAAT/enhancer-	CEBPZ
25.8046	24.2846	25.0604	25.8782	24.6	24.8077	NaN	24.0506	24.6008	25.2661	24.9082	23.6776	Q9BXW7;Q9BXW	Q9BXW7;Q9BXW	Cat eye syndrome	CECR5
27.3388	26.8442	26.9455	27.7919	26.9259	27.1945	26.091	25.8232	26.2072	26.3648	26.7461	26.165	Q92879;G5EA30;(	Q92879;G5EA30;(	CUGBP Elav-like fa	CELF1
24.0469	25.0892	24.0284	24.3198	24.2697	NaN	NaN	NaN	NaN	NaN	NaN	NaN	Q02224;A0A087X(	Q02224;A0A087X(	Centromere-asso	CENPE
29.0059	30.6901	NaN	27.5382	NaN	NaN	NaN	31.5782	NaN	28.9205	NaN	NaN	P49454	P49454	Centromere prote	CENPF
23.8445	23.5167	23.7627	24.482	24.1298	23.8564	NaN	NaN	NaN	23.5613	23.8854	NaN	Q7Z7K6;Q7Z7K6-3	Q7Z7K6;Q7Z7K6-3	Centromere prote	CENPV
NaN	NaN	NaN	NaN	NaN	NaN	22.1954	NaN	NaN	NaN	NaN	NaN	Q9UPN4;Q9UPN4	Q9UPN4;Q9UPN4	Centrosomal prot	CEP131
24.2942	25.3275	24.6598	24.1238	24.3901	24.2249	NaN	NaN	NaN	NaN	NaN	NaN	Q5SW79;H0Y2V6;	Q5SW79;H0Y2V6;	Centrosomal prot	CEP170
21.8798	NaN	NaN	NaN	NaN	NaN	NaN	NaN	NaN	NaN	NaN	NaN	Q9BV73;Q9BV73-	Q9BV73;Q9BV73-	Centrosome-asso	CEP250
NaN	NaN	NaN	NaN	NaN	NaN	NaN	NaN	NaN	NaN	NaN	22.2487	Q9BYV8;Q9BYV8-	Q9BYV8;Q9BYV8-	Centrosomal prot	CEP41
28.3293	28.6635	28.7714	28.6724	28.5104	29.1718	30.2008	29.0139	29.3446	29.5653	28.8047	28.8491	Q53EZ4;Q53EZ4-2	Q53EZ4;Q53EZ4-2	Centrosomal prot	CEP55
NaN	NaN	NaN	NaN	NaN	NaN	30.0809	NaN	NaN	NaN	NaN	NaN	Q96MT8;Q96MT8	Q96MT8;Q96MT8	Centrosomal prot	CEP63
22.0956	NaN	NaN	22.135	NaN	NaN	NaN	NaN	NaN	22.2457	NaN	NaN	Q8TAP6;Q8TAP6-	Q8TAP6;Q8TAP6-	Centrosomal prot	CEP76
24.5487	23.6475	23.9165	24.3715	23.7218	23.7019	24.7555	NaN	23.6971	24.036	24.0112	24.0926	Q8IW35;E9PG22;(	Q8IW35;E9PG22;(	Centrosomal prot	CEP97
23.757	NaN	23.902	24.2068	24.29	24.092	24.668	NaN	24.7121	NaN	NaN	NaN	Q96G23;Q5SZE3;I	Q96G23;Q5SZE3;I	Ceramide synthas	CERS2
24.0232	24.6446	24.5895	23.9835	23.6435	NaN	23.8394	24.0673	23.8656	24.1319	24.8468	24.4201	P41208;Q12798	P41208;Q12798	Centrin-2;Centrin-	CETN2;CETN1
21.6371	NaN	22.5952	21.984	NaN	NaN	NaN	NaN	NaN	NaN	NaN	NaN	O15182;E5RK82;E	O15182;E5RK82;E	Centrin-3	CETN3
NaN	NaN	NaN	22.0162	NaN	NaN	NaN	NaN	NaN	20.9171	NaN	NaN	Q9Y6A4	Q9Y6A4	Cilia- and flagella-	CFAP20
NaN	NaN	NaN	NaN	NaN	NaN	NaN	NaN	22.3529	NaN	NaN	NaN	Q96G28;B5MC35;	Q96G28;B5MC35;	Cilia- and flagella-	CFAP36
NaN	NaN	NaN	NaN	NaN	NaN	25.6754	NaN	NaN	NaN	NaN	NaN	P08603;Q5TFM2;/	P08603;Q5TFM2;/	Complement fact	CFH
31.3843	33.0372	32.8887	31.6305	32.6917	32.673	33.1812	33.2167	32.8195	33.024	33.0704	33.3021	P23528;E9PP50;E	P23528;E9PP50;E	Cofilin-1	CFL1
27.389	27.6311	27.3531	26.7239	27.1843	27.3181	27.1758	26.1843	25.511	28.0732	26.683	26.0332	Q9Y281;Q9Y281-3	Q9Y281;Q9Y281-3	Cofilin-2	CFL2
NaN	NaN	NaN	NaN	NaN	NaN	NaN	NaN	26.9692	NaN	26.3851	27.1557	Q9P2M7;Q9P2M7	Q9P2M7;Q9P2M7	Cingulin	CGN
NaN	NaN	NaN	28.6213	NaN	NaN	NaN	NaN	NaN	NaN	NaN	NaN	Q13111;Q13111-2	Q13111;Q13111-2	Chromatin assem	CHAF1A
23.1663	NaN	22.7163	22.6974	22.8204	23.3302	NaN	22.4269	NaN	NaN	NaN	23.8594	Q13112	Q13112	Chromatin assem	CHAF1B
NaN	NaN	NaN	NaN	NaN	NaN	NaN	NaN	NaN	NaN	NaN	20.9496	Q96BP2	Q96BP2	Coiled-coil-helix-c	CHCHD1
NaN	NaN	NaN	NaN	NaN	NaN	24.0926	NaN	NaN	NaN	NaN	NaN	Q9Y6H1;Q5T1J5	Q9Y6H1;Q5T1J5	Coiled-coil-helix-c	CHCHD2;CHCHD2I
NaN	22.8352	22.1447	NaN	22.8663	NaN	NaN	24.6141	24.3523	NaN	NaN	NaN	Q9NX63;C9JRZ6;F	Q9NX63;C9JRZ6;F	MICOS complex su	CHCHD3
24.0625	NaN	23.7528	24.1464	23.7534	NaN	NaN	NaN	NaN	23.6977	24.0338	NaN	O14646;O14646-2	O14646;O14646-2	Chromodomain-h	CHD1
21.4782	NaN	21.1417	22.0805	NaN	NaN	NaN	NaN	NaN		21.1698	NaN	O14647;O14647-2	O14647;O14647-2	Chromodomain-h	CHD2
27.9342	27.0342	27.5193	28.2311	27.7932	27.8482	25.5396	26.0791	25.2816	27.9708	28.1011	27.4103	Q14839;F5GWX5;	Q14839;F5GWX5;	Chromodomain-h	CHD4
21.1607	NaN	NaN	NaN	NaN	NaN	NaN	NaN	NaN	NaN	NaN	NaN	O14757;J3KN87;C	O14757;J3KN87;C	Serine/threonine-	CHEK1
25.4033	24.5795	25.1133	25.675	25.1462	25.4495	23.8701	NaN	23.6733	25.3225	25.275	24.8574	Q8IWX8;J3QK89	Q8IWX8;J3QK89	Calcium homeost	CHERP
25.0331	25.0067	25.1124	24.8099	24.6327	24.7331	24.5496	NaN	NaN	24.3853	24.2112	NaN	Q9UKJ5;H0Y8H1;D	Q9UKJ5;H0Y8H1;D	Cysteine-rich hyd	CHIC2

NaN	NaN	NaN	NaN	NaN	NaN	22.1157	NaN	NaN	NaN	NaN	NaN	Q9BWS9;Q9BWS	Q9BWS9;Q9BWS	Chitinase domain-	CHID1
24.3296	23.0617	23.3806	24.1984	23.0601	23.3585	NaN	24.0134	NaN	23.9188	23.8417	23.4174	P35790;P35790-2	P35790;P35790-2,	Choline kinase alp	CHKA
23.1038	NaN	23.3859	NaN	NaN	NaN	NaN	NaN	NaN	NaN	NaN	NaN	P24386	P24386	Rab proteins gera	CHM
23.1656	NaN	22.9541	NaN	22.756	NaN	23.3355	NaN	NaN	23.4183	23.2821	NaN	P26374	P26374	Rab proteins gera	CHML
27.6902	28.3834	27.8922	27.4142	28.0547	27.9095	28.8095	28.6373	29.3939	28.2405	28.1316	29.1559	Q9HD42;F8VUA2;	Q9HD42;F8VUA2;	Charged multivesi	CHMP1A
27.4425	28.9039	27.8457	26.844	28.21	27.6701	28.9741	28.8002	29.4426	27.4056	27.8437	28.7964	Q7LBR1	Q7LBR1	Charged multivesi	CHMP1B
28.1013	28.4279	28.2963	27.7009	27.8951	28.2938	29.2255	29.0631	29.7737	28.1402	28.1389	28.9986	O43633;M0R1L7;I	O43633;M0R1L7;I	Charged multivesi	CHMP2A
25.8969	25.7064	25.4966	25.052	25.7233	25.3878	27.399	26.8167	27.5142	26.1721	26.0363	27.4106	Q9UQN3;C9J0A7;	Q9UQN3;C9J0A7;	Charged multivesi	CHMP2B
26.8533	26.3739	27.0786	26.3045	25.944	26.8238	27.5864	26.8376	27.4337	26.7568	26.6444	27.4301	Q9Y3E7;Q9Y3E7-3	Q9Y3E7;Q9Y3E7-3	Charged multivesi	CHMP3
25.6813	25.6434	25.8274	25.7749	24.9702	25.6845	26.4843	26.1203	26.1729	26.0028	25.6323	26.3351	E9PSI1;Q9BY43;E9	E9PSI1;Q9BY43;E9	Charged multivesi	CHMP4A
29.0889	29.2626	29.5555	28.6488	29.0107	29.4628	30.6964	30.6542	30.8613	29.5524	29.8769	30.7633	Q9H444;Q96CF2;I	Q9H444	Charged multivesi	CHMP4B
27.0814	26.3967	26.7443	25.9457	25.8955	26.7786	27.761	27.5351	27.8531	26.7428	25.9421	27.3525	Q9NZZ3;Q9NZZ3-	Q9NZZ3;Q9NZZ3-	Charged multivesi	CHMP5
25.5219	25.1865	25.8489	25.4194	25.2228	25.9901	25.7988	NaN	25.7579	26.0249	26.7767	26.1527	Q96FZ7;I3L4A1;I3	Q96FZ7;I3L4A1	Charged multivesi	CHMP6
23.6564	22.7573	23.0764	22.7411	22.968	NaN	23.6374	NaN	NaN	23.5262	22.7459	NaN	Q8WUX9;E5RJI3;E	Q8WUX9	Charged multivesi	CHMP7
25.9713	25.2069	25.2131	25.5735	25.0975	25.2694	25.7513	25.0714	NaN	25.7373	25.7168	25.4441	Q9UHD1;Q9UHD1	Q9UHD1;Q9UHD1	Cysteine and histi	CHORDC1
25.1113	24.6916	24.6883	24.7956	24.0936	24.1806	24.7614	NaN	NaN	25.29	23.986	NaN	Q99653;H0YNG9;I	Q99653;H0YNG9;I	Calcineurin B hom	CHP1
NaN	NaN	NaN	NaN	NaN	NaN	NaN	20.5263	NaN	NaN	NaN	NaN	Q8IZ52;Q8IZ52-2;	Q8IZ52;Q8IZ52-2;	Chondroitin sulfat	CHPF
23.5681	23.2068	23.04	23.2302	23.6761	23.0504	NaN	NaN	NaN	22.9996	23.7095	23.5826	Q9NRG0;E5RGS9	Q9NRG0;E5RGS9	Chromatin access	CHRAC1
NaN	22.1219	NaN	NaN	22.1437	NaN	24.2925	23.2155	NaN	NaN	NaN	NaN	F8VXK3;Q9NPF2-2	F8VXK3;Q9NPF2-2	Carbohydrate sulf	CHST11
22.4618	22.1982	21.6356	22.3675	NaN	21.6059	23.9385	NaN	NaN	21.6945	NaN	NaN	Q8NCH0;H0YN65	Q8NCH0;H0YN65	Carbohydrate sulf	CHST14
24.8411	23.8231	25.5035	25.7458	24.9229	25.2358	NaN	NaN	25.7859	25.3694	25.3772	25.4878	Q8WVB6;Q8WVB	Q8WVB6;Q8WVB	Chromosome tran	CHTF18
NaN	NaN	NaN	NaN	NaN	NaN	NaN	NaN	NaN	NaN	NaN	19.7089	P0CG13;E5RGY5;J	P0CG13;E5RGY5;J	Chromosome tran	CHTF8
24.1917	NaN	23.6721	23.8479	NaN	23.6677	NaN	NaN	NaN	NaN	23.4831	NaN	Q9Y3Y2;Q9Y3Y2-4	Q9Y3Y2;Q9Y3Y2-4	Chromatin target	CHTOP
26.3515	24.211	25.2596	26.1424	24.7126	25.0994	23.7998	NaN	NaN	25.4971	24.7685	NaN	O15111	O15111	Inhibitor of nuclea	CHUK
NaN	NaN	NaN	21.548	NaN	NaN	NaN	NaN	NaN	NaN	NaN	NaN	B4DL54;P49356;P	B4DL54;P49356;P	Protein farnesyltr	CHURC1-FNTB;FNT
25.5	24.6909	24.8737	25.0685	24.3669	24.8172	25.031	NaN	NaN	NaN	25.3135	NaN	O76071	O76071	Probable cytosolic	CIAO1
24.7789	24.5147	24.3369	24.4395	24.3797	24.5765	25.4694	NaN	25.3209	25.158	25.0288	25.5492	Q6FI81;H3BV90;Q	Q6FI81;H3BV90;Q	Anamorsin	CIAPIN1
23.3655	23.3328	23.0811	23.1124	23.5095	23.4605	NaN	NaN	NaN	NaN	NaN	NaN	Q99828;Q99828-2	Q99828;Q99828-2	Calcium and integ	CIB1
22.8435	NaN	22.8432	23.318	22.2714	22.7634	NaN	NaN	NaN	NaN	NaN	NaN	Q9BW66;H0YN33	Q9BW66;H0YN33	Cyclin-dependent	CINP
23.7896	25.4309	24.864	23.557	25.0244	24.8614	24.1862	NaN	25.0604	23.1797	24.8529	24.914	Q14011;K7EJV1;K	Q14011;K7EJV1;K	Cold-inducible RN	CIRBP
26.2239	25.0027	25.2647	26.6922	25.7782	25.5475	24.5996	24.0202	25.6587	26.0157	26.0508	23.6258	Q969X6;H3BSH7;(	Q969X6;H3BSH7;(	Cirhin	CIRH1A
22.5149	23.9175	23.3934	22.8088	NaN	23.1039	23.1659	NaN	NaN	NaN	NaN	NaN	Q9NZ45	Q9NZ45	CDGSH iron-sulfur	CISD1
23.9157	22.7665	23.6072	24.0993	22.9672	NaN	23.8263	NaN	NaN	NaN	NaN	NaN	Q8N5K1;I3L1N9	Q8N5K1;I3L1N9	CDGSH iron-sulfur	CISD2
29.3687	29.3419	30.0606	29.3519	29.451	30.1192	31.59	30.8953	30.6692	29.9414	29.6251	29.9897	O14578-4;O14578	O14578-4;O14578	Citron Rho-interac	CIT
23.5279	23.6417	23.4348	23.4227	23.6523	NaN	NaN	NaN	NaN	NaN	23.6672	NaN	Q8WWK9;Q8WW	Q8WWK9;Q8WW	Cytoskeleton-asso	CKAP2
29.7768	29.9756	29.8246	29.8493	29.7598	29.9359	29.826	30.0062	30.6839	29.6265	29.3799	29.8954	Q07065	Q07065	Cytoskeleton-asso	CKAP4
27.0284	26.7292	26.8753	26.4462	26.6443	26.5422	27.1929	26.7986	27.1367	26.3995	26.5979	26.6761	Q14008;Q14008-2	Q14008;Q14008-2	Cytoskeleton-asso	CKAP5
25.898	25.7951	25.0775	25.514	25.8302	24.9163	26.8912	26.6802	25.9458	26.4678	25.6635	25.6175	P12277;G3V4N7;I	P12277;G3V4N7	Creatine kinase B-	CKB
23.7051	NaN	NaN	23.8391	NaN	NaN	NaN	NaN	NaN	24.7504	NaN	NaN	Q9UBR5;Q9UBR5-	Q9UBR5;Q9UBR5-	Chemokine-like fa	CKLF;CKLF-CMTM1
24.1992	24.211	23.9522	22.9976	23.6152	23.435	24.1859	24.7074	24.6656	23.9281	23.7469	23.9341	P61024;Q5T179;C	P61024;Q5T179;C	Cyclin-dependent	CKS1B
NaN	NaN	24.2489	23.1918	24.3695	24.1253	24.7916	NaN	24.7755	24.9756	24.9188	24.5608	P33552	P33552	Cyclin-dependent	CKS2
23.5413	23.4315	23.506	23.8009	22.697	22.8577	NaN	NaN	23.2659	22.4585	22.5477	23.5046	Q7Z460;Q7Z460-3	Q7Z460;Q7Z460-3	CLIP-associating p	CLASP1
25.5738	25.6255	25.5272	25.5994	25.1105	24.9787	26.0907	26.1165	27.3463	25.4826	25.4438	26.4977	E7ERI8;E7EW49;O	E7ERI8;E7EW49;O	CLIP-associating p	CLASP2
23.7491	23.1488	23.7832	23.7878	23.7573	23.9968	23.3648	23.5035	24.3976	24.3617	24.1056	NaN	P51790;A0A087W	P51790;A0A087W	H(+)/Cl(-) exchang	CLCN3
NaN	NaN	22.7539	22.5904	NaN	NaN	NaN	NaN	NaN	22.8251	NaN	NaN	P51793;G3XAG5;I	P51793;G3XAG5;I	H(+)/Cl(-) exchang	CLCN4
25.3152	24.3083	24.6274	24.6988	24.1007	24.5339	23.8149	24.3019	23.8186	NaN	23.855	24.1027	P51798;P51798-2	P51798;P51798-2	H(+)/Cl(-) exchang	CLCN7
NaN	NaN	22.475	NaN	NaN	NaN	NaN	NaN	NaN	NaN	NaN	NaN	P56749	P56749	Claudin-12	CLDN12
26.015	24.3907	25.6003	25.3265	24.606	25.4991	25.925	NaN	25.3157	26.2162	25.1121	24.6824	Q9NY35;D6RIU2;I	Q9NY35;D6RIU2;I	Claudin domain-co	CLDND1
22.6753	22.1143	23.1212	23.0387	22.5976	23.2109	NaN	NaN	NaN	NaN	NaN	NaN	O14967;D6RAZ4;(	O14967	Calmegin	CLGN
32.0452	32.1405	32.8536	32.3753	32.7417	32.9426	31.9598	31.2994	31.8112	32.576	32.6997	32.6311	O00299	O00299	Chloride intracellu	CLIC1
31.3575	30.8013	32.1228	31.6689	31.2448	32.3258	30.5636	29.4049	29.6141	31.9845	31.4201	30.6258	Q9Y696;O15247;A	Q9Y696	Chloride intracellu	CLIC4
24.8747	24.5581	25.5872	25.3148	24.9679	24.6903	25.3349	23.9474	24.2443	26.1859	25.7004	25.7854	Q96NY7;Q96NY7-	Q96NY7;Q96NY7-	Chloride intracellu	CLIC6
25.3326	25.2868	25.4742	25.216	25.4122	25.2823	25.6784	NaN	24.4945	26.1479	25.7228	24.7115	Q14677;Q14677-3	Q14677;Q14677-3	Clathrin interacto	CLINT1
26.4623	26.9482	26.5122	25.8969	26.4297	26.4812	26.0491	26.7403	25.8473	25.4901	26.1937	26.6952	P30622;P30622-2	P30622;P30622-2	CAP-Gly domain-c	CLIP1
NaN	NaN	NaN	20.9705	NaN	NaN	NaN	NaN	NaN	NaN	NaN	NaN	Q8N3C7;B5MCH3	Q8N3C7;B5MCH3	CAP-Gly domain-c	CLIP4
NaN	NaN	NaN	23.0064	NaN	NaN	NaN	NaN	NaN	NaN	NaN	NaN	P49759;P49759-3	P49759;P49759-3	Dual specificity pr	CLK1;CLK4
22.1988	NaN	22.0955	22.77	NaN	NaN	NaN	NaN	22.2776	NaN	NaN	NaN	P49761;P49761-1	P49761;P49761-1	Dual specificity pr	CLK3
NaN	NaN	22.6454	NaN	22.2257	22.9584	NaN	NaN	NaN	23.618	23.5452	23.8576	Q9H6B4	Q9H6B4	CXADR-like memb	CLMP
22.1525	NaN	NaN	NaN	NaN	NaN	NaN	NaN	NaN	NaN	NaN	NaN	Q13286;Q9UBD8;	Q13286;Q9UBD8;	Battenin	CLN3
26.3069	24.8459	25.6667	25.6232	24.9479	25.5825	25.999	25.4112	25.4651	26.1882	26.1646	25.8292	P54105;E9PMI6;J	P54105;E9PMI6;J	Methylosome sub	CLNS1A

22.5471	22.1207	21.9745	22.881	22.197	NaN	21.8761	NaN	NaN	NaN	22.5554	NaN	Q92989;E9PL17;Q	Q92989;E9PL17;Q	Polyribonucleotid	CLP1
24.857	24.5633	24.4319	24.886	24.4043	24.6303	24.1693	23.8741	NaN	25.0305	25.0328	NaN	Q9H078;H0YGM0	Q9H078;H0YGM0	Caseinolytic pepti	CLPB
25.5581	25.1984	25.0387	25.2966	25.5105	25.2122	NaN	NaN	25.5708	24.7392	25.0337	NaN	Q16740;M0R208;	Q16740;M0R208	ATP-dependent C	CLPP
24.0268	23.6528	24.4257	24.1173	23.9766	24.2937	24.6718	25.3014	25.425	25.1043	24.6937	24.7095	O96005;O96005-4	O96005;O96005-4	Cleft lip and palat	CLPTM1
22.1495	NaN	23.1625	23.1068	23.0112	23.6058	NaN	23.7175	24.605	23.3214	22.5828	23.7629	Q96KA5;Q96KA5-	Q96KA5;Q96KA5-	Cleft lip and palat	CLPTM1L
25.0315	24.8019	23.9185	25.9778	24.9096	24.3609	NaN	NaN	23.8922	22.2452	NaN	NaN	O76031;H0YM48	O76031	ATP-dependent C	CLPX
26.0337	26.1374	25.801	26.4401	25.8456	24.2408	28.8822	28.7804	NaN	26.1934	26.1577	NaN	O94985;O94985-2	O94985;O94985-2	Calsyntenin-1;Solu	CLSTN1
29.6379	30.434	29.7602	29.8879	30.0761	29.8176	29.1764	29.9582	28.7171	29.2738	28.9037	28.9314	P09496-2;P09496	P09496-2;P09496	Clathrin light chai	CLTA
29.2642	29.3993	29.1049	28.9775	29.0071	28.9548	28.6958	29.0968	27.5023	29.2927	28.539	28.1862	P09497-2;P09497	P09497-2;P09497	Clathrin light chai	CLTB
33.1116	32.7947	33.0981	33.3934	32.85	33.2437	32.6044	32.2057	32.4301	32.7589	32.164	32.2568	Q00610;Q00610-2	Q00610;Q00610-2	Clathrin heavy cha	CLTC
25.8156	25.9262	24.9831	24.6827	25.2836	24.6098	27.7149	26.5409	27.1276	27.2646	26.579	25.9597	P10909;P10909-4	P10909;P10909-4	Clusterin;Clusterir	CLU
NaN	NaN	NaN	NaN	NaN	NaN	22.5313	NaN	NaN	NaN	NaN	NaN	I3L2B0;I3L4B5;I3L	I3L2B0		CLUH
27.4754	25.9109	26.2963	27.1606	26.3036	26.1984	26.309	26.5148	26.1536	26.5177	26.5995	26.3944	K7EIG1;O75153;Q	K7EIG1;O75153	Clustered mitocho	CLUH
24.3348	NaN	NaN	NaN	NaN	NaN	NaN	NaN	NaN	NaN	NaN	NaN	Q8NFW8;Q8NFW	Q8NFW8;Q8NFW	N-acylneuraminat	CMAS
23.9146	24.3074	23.6538	23.2877	24.4459	23.433	25.5754	24.0809	NaN	24.246	24.4959	24.4178	Q96DG6	Q96DG6	Carboxymethylen	CMBL
NaN	NaN	NaN	NaN	NaN	NaN	NaN	NaN	NaN	NaN	23.9205	NaN	Q7Z7K0;H7C3D8	Q7Z7K0;H7C3D8	COX assembly mit	CMC1
23.2713	23.3164	23.0882	NaN	22.8986	NaN	23.24	NaN	23.6708	NaN	NaN	23.5775	Q8IY22;Q8IY22-2;	Q8IY22;Q8IY22-2;	C-Maf-inducing pr	CMIP
27.736	28.1121	27.7401	27.5011	27.786	27.8208	27.9332	27.8455	28.0392	27.9785	27.6266	27.9835	P30085;Q5T0D2;F	P30085;Q5T0D2	UMP-CMP kinase	CMPK1
25.4568	24.3994	24.6873	25.7529	24.4101	24.8001	NaN	NaN	NaN	24.0334	24.4659	NaN	Q9BQ75;Q9BQ75-	Q9BQ75;Q9BQ75-	Protein CMSS1	CMSS1
24.25	NaN	24.6235	24.6164	24.7781	24.5027	NaN	NaN	NaN	NaN	NaN	NaN	H3BUD5;Q96MX0	H3BUD5;Q96MX0	CKLF-like MARVEL	CMTM3
23.7515	NaN	NaN	23.7524	NaN	NaN	NaN	NaN	NaN	23.8928	24.0245	NaN	Q8IZR5-3;Q8IZR5-	Q8IZR5-3;Q8IZR5-	CKLF-like MARVEL	CMTM4
26.3437	26.6249	27.4587	27.4026	26.9771	27.3925	26.7827	NaN	27.7331	27.0688	27.277	27.9016	Q9NX76	Q9NX76	CKLF-like MARVEL	CMTM6
NaN	NaN	NaN	26.6391	NaN	NaN	NaN	NaN	NaN	NaN	NaN	NaN	Q96FZ5;F8WDZ3;(	Q96FZ5;F8WDZ3;(	CKLF-like MARVEL	CMTM7
26.6171	24.5336	25.1048	25.9056	25.6315	25.7687	23.5552	23.2737	22.8003	26.3095	25.057	24.155	Q8N1G2;Q5T7F5;	Q8N1G2	Cap-specific mRN/	CMTR1
27.4164	27.9502	27.2528	26.8307	27.506	27.2196	27.6773	27.6676	27.8314	27.4724	27.2883	28.1718	Q96KP4;Q96KP4-:	Q96KP4;Q96KP4-:	Cytosolic non-spe	CNDP2
NaN	NaN	NaN	NaN	NaN	NaN	NaN	NaN	NaN	NaN	NaN	NaN	H3BN40;Q8N9A8-	H3BN40;Q8N9A8-	Nuclear envelope	CNEP1R1
NaN	NaN	NaN	NaN	NaN	NaN	24.0065	NaN	NaN	NaN	NaN	NaN	A6NLH6;Q9P003	A6NLH6;Q9P003	Protein cornichon	CNIH4
NaN	NaN	NaN	NaN	NaN	NaN	NaN	NaN	NaN	21.018	NaN	NaN	Q6P9H4;Q8WXI2;	Q6P9H4;Q8WXI2;	Connector enhanc	CNKSR3;CNKSR2
27.2695	27.0097	27.5744	27.5401	27.0738	27.8199	26.8818	NaN	26.2708	27.6741	27.0394	27.1002	Q99439;B4DDF4;I	Q99439;B4DDF4;I	Calponin-2;Calpor	CNN2
28.6129	29.4312	29.2575	29.0688	29.1176	29.5245	28.767	28.5662	28.4444	28.9574	29.3655	29.3794	Q15417;Q15417-3	Q15417;Q15417-3	Calponin-3;Calpor	CNN3
22.0767	22.3962	22.5009	22.3766	22.601	23.2122	NaN	NaN	NaN	NaN	NaN	NaN	Q9H8M5;Q9H8M!	Q9H8M5;Q9H8M!	Metal transporter	CNNM2
23.8623	23.5518	24.3558	24.1599	23.6407	24.3183	23.8273	24.9778	24.3019	25.0599	24.9078	24.3153	Q8NE01;Q8NE01-	Q8NE01;Q8NE01-	Metal transporter	CNNM3
23.1164	23.1009	23.8104	23.5252	23.9339	24.342	23.1152	NaN	23.8123	24.133	24.0724	24.4092	Q6P4Q7;Q6P4Q7-	Q6P4Q7;Q6P4Q7-	Metal transporter	CNNM4
26.7334	25.3176	25.7957	26.3043	25.574	25.9134	25.5713	26.0265	25.6686	25.8644	25.999	25.6497	A5YKK6;A5YKK6-2	A5YKK6;A5YKK6-2	CCR4-NOT transcr	CNOT1
23.2654	NaN	22.698	23.0993	22.7388	NaN	NaN	22.6353	NaN	22.8617	23.0566	NaN	Q9H9A5;Q9H9A5-	Q9H9A5;Q9H9A5-	CCR4-NOT transcr	CNOT10
NaN	NaN	NaN	21.1678	NaN	NaN	NaN	NaN	NaN	NaN	NaN	NaN	Q9UKZ1	Q9UKZ1	CCR4-NOT transcr	CNOT11
22.6397	23.1095	23.386	22.5921	22.7246	NaN	NaN	24.0262	NaN	NaN	NaN	NaN	Q9NZN8;F8VV52;(	Q9NZN8;F8VV52;(	CCR4-NOT transcr	CNOT2
23.7984	NaN	23.5399	23.5331	23.9421	24.15	NaN	23.8353	NaN	23.8223	24.0463	NaN	O75175;H7C3F5;C	O75175;H7C3F5;C	CCR4-NOT transcr	CNOT3
NaN	NaN	NaN	21.0386	NaN	NaN	NaN	NaN	NaN	NaN	NaN	NaN	Q96LI5;Q9ULM6;I	Q96LI5;Q9ULM6;I	CCR4-NOT transcr	CNOT6L;CNOT6
23.7409	23.6027	23.6946	23.7897	23.8	23.7147	23.4	24.5153	23.7795	NaN	23.6084	NaN	Q9UIV1;Q9UIV1-2	Q9UIV1;Q9UIV1-2	CCR4-NOT transcr	CNOT7
29.6697	29.4438	29.9684	29.67	29.4449	30.0767	30.245	29.2741	29.2524	30.8164	30.2556	30.005	P09543;P09543-2	P09543;P09543-2	2,3-cyclic-nucleoti	CNP
23.6122	25.1383	24.2324	23.8855	25.3887	NaN	24.6473	26.1199	25.6552	24.8424	24.849	24.7633	Q9Y2B0;F8VXJ7;F!	Q9Y2B0;F8VXJ7;F!	Protein canopy hc	CNPY2
23.2949	23.7229	NaN	23.2778	24.013	NaN	23.6751	NaN	24.2128	NaN	NaN	NaN	Q9BT09;A0A0C4D	Q9BT09	Protein canopy hc	CNPY3
NaN	NaN	22.5045	NaN	23.3564	NaN	NaN	NaN	NaN	NaN	22.7759	NaN	Q9Y2R0;K7EPV0	Q9Y2R0;K7EPV0	Cytochrome c oxi	COA3
26.8795	26.6713	26.4199	26.8679	26.7305	26.3394	25.7469	NaN	25.7543	26.3209	26.2219	26.0743	Q13057;Q13057-2	Q13057;Q13057-2	Bifunctional coen:	COASY
NaN	NaN	NaN	NaN	22.3594	NaN	NaN	NaN	NaN	NaN	NaN	NaN	Q53SF7;A0A0B4J1	Q53SF7;A0A0B4J1	Cordon-bleu prote	COBLL1
24.7216	23.7406	24.4865	24.8804	24.6348	24.4725	NaN	NaN	23.9201	23.9626	24.1195	NaN	Q8WTW3;A0A087	Q8WTW3;A0A087	Conserved oligom	COG1
22.9578	NaN	23.4347	23.8643	NaN	NaN	NaN	NaN	NaN	23.6352	23.1803	NaN	Q14746;Q14746-2	Q14746;Q14746-2	Conserved oligom	COG2
25.1085	24.7943	24.4702	24.855	24.9412	23.814	NaN	NaN	NaN	24.2745	24.245	24.4268	Q96JB2;Q96JB2-2	Q96JB2	Conserved oligom	COG3
25.4073	NaN	23.7064	24.8251	24.2109	24.3642	NaN	NaN	23.3973	24.0224	NaN	23.3973	Q9H9E3;A0A0A0N	Q9H9E3;A0A0A0N	Conserved oligom	COG4
24.824	23.85	24.0133	24.8606	23.5497	23.9683	23.2241	NaN	NaN	24.2096	23.8713	NaN	Q9UP83;Q9UP83-	Q9UP83;Q9UP83-	Conserved oligom	COG5
25.2074	23.6015	24.4713	24.8735	24.2251	24.3698	NaN	NaN	NaN	23.8626	23.8339	NaN	Q9Y2V7;Q9Y2V7-:	Q9Y2V7;Q9Y2V7-:	Conserved oligom	COG6
24.1601	23.8014	23.8085	24.3705	23.6363	23.5092	NaN	NaN	NaN	23.4143	NaN	NaN	P83436	P83436	Conserved oligom	COG7
22.2877	NaN	22.6797	23.816	23.3253	22.8156	NaN	NaN	NaN	22.3175	22.5584	NaN	Q96MW5;H3BQV.	Q96MW5;H3BQV.	Conserved oligom	COG8
24.4574	25.5423	25.2842	24.4722	25.3389	25.4466	25.1025	NaN	NaN	NaN	24.6518	NaN	P38432	P38432	Coilin	COIL
25.6495	NaN	22.4539	23.7472	22.2767	22.1745	23.9181	NaN	NaN	NaN	NaN	NaN	Q99715;D6RGG3;	Q99715;D6RGG3;	Collagen alpha-1(:	COL12A1
25.572	24.6793	25.025	25.1298	24.4563	24.8813	24.2428	25.3994	24.9795	24.11	24.1707	NaN	Q5TAT6;Q5TAT6-!	Q5TAT6;Q5TAT6-!	Collagen alpha-1(:	COL13A1
22.8725	24.5954	25.4631	21.4726	22.3394	24.0401	29.028	28.708	26.2272	26.0686	25.0877	24.8819	P39060;P39060-2	P39060;P39060-2	Collagen alpha-1(:	COL18A1
NaN	24.9093	NaN	NaN	23.6734	NaN	24.5763	24.5118	NaN	NaN	NaN	NaN	P08123;A0A087W	P08123;A0A087W	Collagen alpha-2(I	COL1A2
NaN	NaN	NaN	NaN	NaN	NaN	25.3486	NaN	NaN	22.9381	NaN	NaN	P08572	P08572	Collagen alpha-2(I	COL4A2

23.345	23.7122	23.4134	23.1272	23.2901	22.9458	23.9724	23.6957	22.9799	23.7117	NaN	23.727	Q9Y5P4;Q9Y5P4-2	Q9Y5P4;Q9Y5P4-2	Collagen type IV a	COL4A3BP
23.5862	24.5925	25.6594	22.9757	23.3069	24.7828	24.9191	25.9253	24.5096	NaN	NaN	NaN	P20908;A0A087W	P20908;A0A087W	Collagen alpha-1(	COL5A1
24.3131	26.8504	24.0512	23.856	26.0996	24.4367	28.6233	27.8667	27.2159	25.972	25.0341	25.7425	P12109;A0A087X(	P12109;A0A087X(	Collagen alpha-1(	COL6A1
NaN	NaN	NaN	NaN	NaN	NaN	25.3297	NaN	NaN	NaN	NaN	NaN	P12110;P12110-3	P12110;P12110-3	Collagen alpha-2(	COL6A2
26.6933	24.3732	25.9797	26.1863	23.6444	24.6544	27.1417	25.8913	NaN	26.1276	23.2444	NaN	P27658;A0A087W	P27658;A0A087W	Collagen alpha-1(	COL8A1
27.3298	27.6523	27.4168	27.2186	27.7041	27.5134	26.8724	27.5145	28.0238	27.224	27.2722	27.1866	Q8NBJ5;M0QYH0	Q8NBJ5	Procollagen galact	COLGALT1
22.1372	22.1404	22.3763	22.652	22.8443	NaN	22.0364	NaN	22.6522	23.1157	22.9302	22.4465	Q86X83;Q86X83-2	Q86X83;Q86X83-2	COMM domain-cc	COMMD2
NaN	NaN	NaN	22.754	NaN	NaN	NaN	NaN	NaN	22.6871	22.8693	NaN	R4GMX3;Q9UBI1;	R4GMX3;Q9UBI1;	COMM domain-cc	COMMD3-BMI1;C
22.2634	NaN	NaN	NaN	NaN	NaN	NaN	NaN	NaN	NaN	NaN	NaN	Q9H0A8;A0A0B4J	Q9H0A8;A0A0B4J	COMM domain-cc	COMMD4
22.0258	NaN	21.4915	NaN	21.6158	NaN	NaN	NaN	NaN	21.7796	NaN	NaN	Q9GZQ3;H0YEQ6	Q9GZQ3;H0YEQ6	COMM domain-cc	COMMD5
22.5361	NaN	22.7843	22.4233	NaN	22.3258	NaN	NaN	NaN	22.5821	NaN	NaN	Q7Z4G1;Q7Z4G1-	Q7Z4G1;Q7Z4G1-	COMM domain-cc	COMMD6
23.7622	23.5591	23.1489	24.3266	23.528	23.8308	NaN	NaN	NaN	23.9262	NaN	NaN	Q9NX08	Q9NX08	COMM domain-cc	COMMD8
22.718	22.5801	22.3341	NaN	22.664	NaN	NaN	NaN	NaN	NaN	NaN	NaN	Q9P000;Q9P000-2	Q9P000;Q9P000-2	COMM domain-cc	COMMD9
25.355	24.8171	24.9064	24.8061	24.8263	25.3141	25.2862	25.23	24.3677	24.7052	24.6223	24.3103	P21964;P21964-2	P21964;P21964-2	Catechol O-methy	COMT
30.4994	29.6332	30.2449	30.4819	29.9383	30.4064	29.7044	30.0188	29.7831	30.3704	30.1796	29.7646	P53621;P53621-2	P53621;P53621-2	Coatomer subunit	COPA
30.6837	29.5862	30.1517	30.6807	29.824	30.2367	29.5599	29.3174	29.192	29.8267	29.5059	28.9552	P53618;E9PP73;E	P53618	Coatomer subunit	COPB1
29.7598	29.4128	29.5085	29.5618	29.3304	29.5072	29.3962	29.5208	29.1257	29.654	29.431	29.1578	P35606;P35606-2	P35606;P35606-2	Coatomer subunit	COPB2
28.1016	27.8776	27.9081	27.7361	27.4982	27.7886	27.7469	27.7481	27.2047	27.5435	27.6867	27.3514	O14579;M0QXB4;	O14579;M0QXB4;	Coatomer subunit	COPE
30.2963	29.6229	30.0069	30.1499	29.7188	30.1806	29.3629	29.7542	29.574	29.7601	29.8086	29.6856	Q9Y678;H0Y8X7;D	Q9Y678	Coatomer subunit	COPG1
27.6137	26.6672	27.2395	27.4492	26.9841	27.3601	26.1892	26.6773	26.6125	27.0945	26.9051	26.8422	Q9UBF2;Q9UBF2-	Q9UBF2;Q9UBF2-	Coatomer subunit	COPG2
26.7273	26.1402	26.6683	26.7392	26.4276	26.9308	26.1166	26.6893	26.2525	26.4705	26.7599	26.6576	P61201;P61201-2	P61201;P61201-2	COP9 signalosom	COPS2
26.4947	26.0586	26.0763	26.3814	26.3077	26.1838	26.0831	25.7949	26.2298	25.9553	26.3921	26.1498	Q9UNS2;Q9UNS2	Q9UNS2;Q9UNS2	COP9 signalosom	COPS3
26.8608	27.3116	26.9995	26.9547	27.3481	27.1086	27.0515	27.4262	27.0813	27.1151	27.2682	27.2686	Q9BT78;D6RFN0;I	Q9BT78;D6RFN0;I	COP9 signalosom	COPS4
24.9461	25.1635	24.8987	24.8906	24.8014	24.8896	24.9507	25.6026	24.7756	25.0367	25.1845	25.2221	Q92905;E5RHH5;I	Q92905;E5RHH5	COP9 signalosom	COPS5
26.2131	25.6638	25.8821	25.9566	25.7063	25.6896	24.6248	25.1548	25.7814	25.9201	26.06	25.8549	Q7L5N1;E7EM64;	Q7L5N1;E7EM64	COP9 signalosom	COPS6
25.2737	25.2205	24.9133	25.4835	24.8319	24.8371	NaN	NaN	NaN	24.2123	NaN	24.088	Q9UBW8;F5H7C6	Q9UBW8;F5H7C6	COP9 signalosom	COPS7A
NaN	NaN	NaN	23.2902	NaN	NaN	NaN	NaN	NaN	NaN	NaN	NaN	Q9H9Q2;J3KQ34;	Q9H9Q2;J3KQ34;	COP9 signalosom	COPS7B
25.2426	25.5007	25.0721	25.3069	25.3265	25.07	24.4228	24.8085	24.4176	24.9975	24.9374	24.8805	Q99627;E9PGT6;C	Q99627;E9PGT6;C	COP9 signalosom	COPS8
27.038	27.4642	27.7332	27.999	27.3346	27.7266	26.9289	26.8636	25.7661	27.8937	27.263	26.2057	P61923;F8VVA7;P	P61923;F8VVA7;P	Coatomer subunit	COPZ1
NaN	NaN	NaN	20.837	NaN	NaN	NaN	NaN	NaN	NaN	NaN	NaN	Q5HYK3;F8VVX6;F	Q5HYK3;F8VVX6;F	2-methoxy-6-poly	COQ5
22.3367	22.7818	22.5954	22.4142	23.982	NaN	22.9007	NaN	NaN	22.7372	22.5784	NaN	P31146;H3BRY3;H	P31146;H3BRY3	Coronin-1A;Coron	CORO1A
27.6893	28.2243	28.202	27.5984	28.3373	28.5046	28.4308	28.2364	28.2997	28.5198	28.5983	28.5314	Q9BR76;A0A087V	Q9BR76;A0A087V	Coronin-1B;Coron	CORO1B
30.079	30.583	30.7008	30.2526	30.7045	30.7441	30.6886	30.5068	30.1885	31.1908	31.1467	30.7379	Q9ULV4;Q9ULV4-	Q9ULV4;Q9ULV4-	Coronin-1C;Coron	CORO1C
22.8125	22.4615	22.6684	22.6222	NaN	NaN	NaN	NaN	NaN	NaN	22.0779	NaN	Q92828	Q92828	Coronin-2A	CORO2A
24.1329	23.6684	23.6434	23.8391	23.623	23.6767	23.3498	NaN	NaN	23.8215	23.042	NaN	Q9UQ03;Q9UQ03	Q9UQ03;Q9UQ03	Coronin-2B	CORO2B
26.3534	25.7065	26.0083	26.2236	25.8977	26.1228	25.5297	25.4786	25.961	25.9175	26.2961	25.9608	P57737;A0A0A6Y	P57737;A0A0A6Y	Coronin-7;Coronin	CORO7;CORO7-PA
29.7943	29.6524	29.9496	29.6835	29.5094	29.9875	29.8293	28.6366	28.7583	30.7445	30.0598	29.4297	Q14019;H3BT58	Q14019;H3BT58	Coactosin-like pro	COTL1
26.4944	27.0905	26.6298	26.8471	26.8586	26.8248	26.0262	27.2509	27.923	25.4815	26.2391	26.6883	P13073;H3BN72;H	P13073;H3BN72;H	Cytochrome c oxi	COX4I1
26.1226	27.5337	26.9925	26.844	27.4656	27.1238	26.3052	28.4383	29.1288	25.8711	26.796	27.6688	P20674;H3BNX8;H	P20674;H3BNX8;H	Cytochrome c oxi	COX5A
25.1726	26.5641	25.8703	25.6326	26.6596	25.9948	25.2795	27.7533	27.9677	25.0417	26.0433	26.6822	P10606	P10606	Cytochrome c oxi	COX5B
NaN	NaN	23.8419	NaN	NaN	24.4058	NaN	NaN	NaN	NaN	NaN	NaN	P12074	P12074	Cytochrome c oxi	COX6A1
24.1188	NaN	NaN	24.6936	23.8245	NaN	23.178	NaN	NaN	NaN	NaN	NaN	P14854;K7EQD3	P14854	Cytochrome c oxi	COX6B1
25.0563	25.8864	25.7017	25.78	26.1825	25.9781	24.4014	25.907	26.4232	24.3963	25.1005	25.0473	P09669	P09669	Cytochrome c oxi	COX6C
NaN	24.2486	23.5967	NaN	23.7331	24.0641	23.7786	24.721	24.9	NaN	NaN	23.8471	P14406;D6RIE3;D	P14406;D6RIE3;D	Cytochrome c oxi	COX7A2
22.0642	NaN	NaN	21.9351	NaN	NaN	NaN	NaN	NaN	NaN	NaN	NaN	O14548;E5RJZ1;H	O14548;E5RJZ1;H	Cytochrome c oxi	COX7A2L
23.8883	24.2788	23.8111	24.2452	24.0863	24.0227	24.0887	NaN	24.6051	NaN	NaN	23.7422	P15954;D6R9Z7	P15954;D6R9Z7	Cytochrome c oxi	COX7C
26.1776	26.6012	26.9803	25.9724	26.7062	26.6672	25.8629	25.357	26.6999	25.3529	24.6561	24.731	O75976;O75976-2	O75976;O75976-2	Carboxypeptidase	CPD
NaN	21.144	NaN	NaN	NaN	NaN	NaN	NaN	NaN	NaN	NaN	NaN	E5RFP2;Q17RY0-3	E5RFP2;Q17RY0-3	Cytoplasmic polya	CPEB4;CPEB3;CPE
30.273	30.1354	30.14	29.9768	30.3393	30.2023	30.3758	30.6524	30.4379	30.8781	30.7731	30.7764	Q99829;B0QZ18;F	Q99829;B0QZ18;F	Copine-1	CPNE1
24.9042	25.3495	24.9338	25.0029	25.1563	NaN	25.946	24.2728	NaN	25.7272	25.0996	25.2434	Q96FN4;Q96FN4-	Q96FN4;Q96FN4-	Copine-2	CPNE2
29.2708	28.6308	28.7236	29.26	28.4153	28.6888	28.5646	28.04	27.7001	29.0572	28.4244	27.9856	O75131;A0A087W	O75131;A0A087W	Copine-3	CPNE3
23.9373	24.045	23.7239	23.4799	23.196	23.4149	23.8279	24.2815	24.1337	23.8826	NaN	23.7906	Q86YQ8;E7ENV7;(	Q86YQ8;E7ENV7;(	Copine-8	CPNE8
25.0059	26.0367	25.1585	24.8992	25.8276	24.9977	25.2248	25.9483	26.6095	24.902	25.6009	26.1583	P36551;H0YA22;P	P36551	Oxygen-depender	CPOX
23.0406	NaN	NaN	22.1714	NaN	NaN	23.5608	NaN	NaN	NaN	NaN	NaN	Q9BRF8;Q9BRF8-	Q9BRF8;Q9BRF8-	Serine/threonine-	CPPED1
25.3646	NaN	28.8749	NaN	28.2139	NaN	NaN	NaN	NaN	NaN	NaN	24.602	P31327;P31327-3	P31327;P31327-3	Carbamoyl-phosp	CPS1
26.046	25.1305	25.3681	25.9955	25.5922	25.5364	24.2463	NaN	24.0579	25.7364	25.9178	25.5	Q10570;A0A087X	Q10570	Cleavage and poly	CPSF1
23.9207	NaN	23.8169	24.2192	23.6505	23.9334	NaN	NaN	NaN	23.5664	23.657	23.6155	Q9P2I0;H0YJF4	Q9P2I0	Cleavage and poly	CPSF2
24.7401	24.1581	24.215	24.7231	24.0799	24.4292	NaN	NaN	NaN	23.9692	24.2902	24.3992	Q9UKF6;G5E9W3	Q9UKF6;G5E9W3	Cleavage and poly	CPSF3
22.6151	NaN	22.0167	23.2286	22.0388	22.6158	NaN	NaN	NaN	22.1782	NaN	NaN	Q5TA45;Q5TA45-	Q5TA45;Q5TA45-	Integrator comple	CPSF3L
24.9723	24.5183	24.459	24.9965	24.7367	24.5255	24.2298	NaN	NaN	24.6005	24.7554	24.8479	Q16630;Q16630-2	Q16630;Q16630-2	Cleavage and poly	CPSF6

25.6071	25.3767	25.228	25.2738	25.3117	25.113	23.8458	NaN	24.498	25.4724	25.9193	25.4186	Q8N684;F5H669;(	Q8N684;F5H669;(	Cleavage and poly	CPSF7
22.0818	NaN	NaN	23.0266	NaN	NaN	NaN	NaN	23.6678	22.1877	NaN	NaN	P50416;P50416-2	P50416;P50416-2	Carnitine O-palmi	CPT1A
24.4603	23.0775	23.7914	24.6843	23.6044	24.0893	NaN	NaN	NaN	23.6655	23.1548	NaN	P23786	P23786	Carnitine O-palmi	CPT2
23.4828	NaN	NaN	NaN	NaN	NaN	NaN	NaN	NaN	NaN	NaN	NaN	Q9BSW2-2;Q9BSV	Q9BSW2-2;Q9BSV	EF-hand calcium-b	CRACR2A
NaN	NaN	NaN	NaN	NaN	NaN	NaN	NaN	NaN	NaN	21.6349	NaN	P43155;P43155-2	P43155;P43155-2	Carnitine O-acetyl	CRAT
NaN	NaN	NaN	NaN	NaN	NaN	NaN	NaN	NaN	NaN	22.1517	NaN	Q96SW2;Q96SW2	Q96SW2;Q96SW2	Protein cereblon	CRBN
24.5531	24.7225	23.7742	23.5322	23.6136	NaN	NaN	NaN	24.8569	22.9506	22.7988	NaN	Q9NZV1	Q9NZV1	Cysteine-rich mot	CRIM1
25.2952	25.912	26.6126	25.8733	25.9358	26.7121	NaN	NaN	NaN	23.7197	NaN	NaN	P52943;P52943-2,	P52943;P52943-2,	Cysteine-rich prot	CRIP2
NaN	24.0398	23.7936	NaN	24.1713	23.7655	NaN	NaN	NaN	NaN	NaN	NaN	Q9H0B8;H3BTI0;H	Q9H0B8;H3BTI0;H	Cysteine-rich secr	CRISPLD2
26.2927	26.9672	26.6598	25.8931	26.6897	26.228	26.4021	26.629	26.5441	26.0968	25.9767	26.5207	P46108;I3L297	P46108	Adapter molecule	CRK
NaN	NaN	NaN	NaN	23.5443	NaN	NaN	NaN	NaN	NaN	NaN	NaN	P46108-2	P46108-2	Adapter molecule	CRK
25.7198	26.1793	26.2742	25.7276	26.2561	26.3054	26.0423	25.7932	25.8167	26.3537	26.1946	26.2198	P46109	P46109	Crk-like protein	CRKL
23.167	NaN	NaN	23.4566	23.7337	NaN	NaN	NaN	NaN	NaN	NaN	NaN	Q8IUI8;Q8IUI8-2;J	Q8IUI8;Q8IUI8-2	Cytokine receptor	CRLF3
25.8251	24.1578	24.9022	25.9087	24.3113	24.1649	NaN	NaN	NaN	24.9764	24.6358	23.6541	Q9BZJ0;Q5JY65;Q	Q9BZJ0;Q5JY65;Q	Crooked neck-like	CRNKL1
25.2363	25.3533	25.2779	25.0643	25.3611	25.2802	25.7741	26.1946	26.0461	25.7188	25.0958	25.0206	O75718;C9JP16	O75718;C9JP16	Cartilage-associat	CRTAP
24.6316	24.8916	24.3291	23.9447	24.2215	24.2758	25.3854	25.4745	25.243	25.6214	25.6197	NaN	Q08257;Q08257-3	Q08257;Q08257-3	Quinone oxidored	CRYZ
24.3841	24.9849	24.6357	24.344	24.9736	24.3192	25.1017	NaN	NaN	24.6034	24.5991	NaN	O95825;A6NMA8	O95825;A6NMA8	Quinone oxidored	CRYZL1
27.7742	27.607	27.3559	27.5756	27.7371	27.4285	26.5796	27.2636	27.7352	27.3624	28.2845	27.4416	O75390;B4DJV2;A	O75390;B4DJV2;A	Citrate synthase,	CS
29.6648	29.0825	29.1888	29.7367	29.1229	29.2126	28.6298	29.2405	28.5431	28.8393	29.055	28.6663	O75534;O75534-2	O75534;O75534-2	Cold shock domai	CSDE1
30.9199	30.2341	30.4772	30.725	30.3399	30.5704	30.5948	30.2493	30.2273	31.0563	30.885	30.7169	P55060;P55060-3,	P55060;P55060-3,	Exportin-2	CSE1L
23.6541	24.4042	23.5683	23.1315	23.5418	22.8145	26.2367	25.9867	23.1919	22.8487	NaN	22.5789	P09603;E9PJA2;P(	P09603;E9PJA2;P(	Macrophage colo	CSF1
23.0196	NaN	22.605	22.4108	NaN	NaN	NaN	NaN	NaN	23.3828	23.5411	NaN	P09919;J3QRX2;J3	P09919;J3QRX2;J3	Granulocyte color	CSF3
27.381	26.6192	26.8083	27.0824	26.5425	26.6458	26.7919	26.3735	26.6509	27.9955	27.7703	26.8697	P41240;H3BUM9;	P41240	Tyrosine-protein k	CSK
26.2636	25.4121	26.0403	26.3603	25.8724	26.2025	25.8765	26.0093	24.5976	25.9627	25.6765	25.5864	P48729;P48729-3,	P48729;P48729-3,	Casein kinase I iso	CSNK1A1
24.3209	22.708	23.4285	23.7537	NaN	23.4076	22.9184	NaN	NaN	NaN	NaN	NaN	P49674;P48730;P4	P49674;P48730;P4	Casein kinase I iso	CSNK1E;CSNK1D
20.3841	NaN	23.0466	21.7056	NaN	22.8574	NaN	NaN	NaN	22.413	21.9109	NaN	Q9HCP0;U3KQA5;	Q9HCP0;U3KQA5;	Casein kinase I iso	CSNK1G1;hCG_20
22.5649	NaN	23.3518	22.7006	NaN	NaN	NaN	NaN	NaN	23.7873	NaN	NaN	P78368;K7ESB6;A	P78368;K7ESB6	Casein kinase I iso	CSNK1G2
NaN	NaN	NaN	22.4325	NaN	NaN	NaN	NaN	NaN	NaN	NaN	NaN	Q9Y6M4	Q9Y6M4	Casein kinase I iso	CSNK1G3
26.6037	26.7893	27.255	26.9005	26.4932	27.3454	26.715	25.7959	26.3375	27.4568	27.0931	26.8917	Q9Y6M4-3;Q9Y6N	Q9Y6M4-3;Q9Y6N	Casein kinase I iso	CSNK1G3
29.4739	29.7338	29.2918	29.6121	29.5057	29.2673	29.4696	29.5117	29.4585	29.0453	29.5771	29.1595	P68400;E7EU96;Q	P68400;E7EU96;Q	Casein kinase II su	CSNK2A1;CSNK2A
27.2488	27.2117	27.4015	27.7199	27.585	27.4285	27.8081	27.4974	27.4356	27.5747	27.6895	27.64	P19784;H3BSA1;H	P19784;H3BSA1	Casein kinase II su	CSNK2A2
28.1254	27.0218	27.5535	27.6881	27.0683	27.5057	27.7618	27.8681	26.1182	27.647	27.9125	26.5693	P67870;Q5SRQ6;(	P67870;Q5SRQ6;(	Casein kinase II su	CSNK2B;CSNK2B-L
25.3807	26.0216	25.7367	25.4883	26.009	25.7912	27.3271	26.2252	26.0163	27.5785	25.9719	25.8515	Q6UVK1	Q6UVK1	Chondroitin sulfat	CSPG4
25.4639	25.5796	NaN	25.1633	NaN	NaN	NaN	NaN	NaN	NaN	NaN	NaN	P21291;E9PS42;E9	P21291;E9PS42;E9	Cysteine and glyci	CSRP1
23.9975	24.7678	24.4133	23.993	24.2026	24.3121	24.0707	NaN	NaN	24.5965	24.6556	NaN	Q16527;F8VQR7;F	Q16527;F8VQR7;F	Cysteine and glyci	CSRP2
22.6841	24.3811	23.1753	22.756	23.4251	23.4391	29.9829	29.8925	28.2137	25.8285	25.7072	27.4382	P01037	P01037	Cystatin-SN	CST1
NaN	NaN	NaN	NaN	NaN	NaN	24.5417	NaN	NaN	NaN	NaN	NaN	P09228	P09228	Cystatin-SA	CST2
25.8742	27.4036	25.9925	25.906	26.7335	26.1986	31.0177	30.368	28.9355	28.1644	27.7561	27.9576	P01034	P01034	Cystatin-C	CST3
NaN	24.2458	NaN	NaN	NaN	NaN	28.2705	26.7824	26.1842	25.1253	24.2174	25.4111	P01036	P01036	Cystatin-S	CST4
26.7007	26.3965	26.1172	27.0637	26.3019	26.1088	27.1427	26.0093	26.2494	27.2712	26.8955	26.4016	P04080	P04080	Cystatin-B	CSTB
26.8203	26.9754	26.6611	26.9435	27.0882	26.7892	26.1985	26.9246	26.5692	26.5258	26.81	27.032	Q05048;A0A0A0N	Q05048;A0A0A0N	Cleavage stimulat	CSTF1
24.038	23.739	23.6444	24.2845	24.0962	24.1008	NaN	NaN	23.6769	23.7696	24.2008	24.8014	P33240;E7EWR4;F	P33240;E7EWR4;F	Cleavage stimulat	CSTF2
25.8099	25.0458	25.3263	25.6732	25.5547	25.62	NaN	24.5123	24.8233	25.1213	25.0181	NaN	Q9H0L4	Q9H0L4	Cleavage stimulat	CSTF2T
25.4054	25.0941	25.3691	25.4401	25.3282	25.1465	NaN	NaN	24.6397	25.0357	25.0354	25.0878	Q12996;E9PLP8;Q	Q12996	Cleavage stimulat	CSTF3
22.4115	NaN	NaN	NaN	NaN	NaN	NaN	NaN	NaN	NaN	NaN	NaN	G3V599;O15320;(	G3V599;O15320;(	cTAGE family mer	CTAGE5;MIA2
25.3122	25.0236	25.1295	25.5082	25.4436	25.133	24.1926	NaN	25.2291	25.5035	25.9236	25.8415	Q13363;Q13363-2	Q13363;Q13363-2	C-terminal-bindin	CTBP1
27.267	26.4373	26.7235	27.4703	27.0483	26.841	25.7238	25.8162	26.0849	27.3367	27.4895	26.7776	P56545;P56545-2,	P56545;P56545-2,	C-terminal-bindin	CTBP2
NaN	NaN	NaN	NaN	NaN	NaN	26.0154	25.8133	NaN	NaN	NaN	NaN	Q01459	Q01459	Di-N-acetylchitobi	CTBS
NaN	NaN	NaN	23.2262	NaN	NaN	NaN	NaN	NaN	NaN	NaN	NaN	P49711;P49711-2	P49711;P49711-2	Transcriptional re	CTCF
24.0236	24.7918	25.1574	24.4163	24.9126	25.0751	24.1315	NaN	24.552	25.0946	25.1829	25.0442	Q9GZU7;Q9GZU7-	Q9GZU7;Q9GZU7-	Carboxy-terminal	CTDSP1
22.5525	NaN	23.0799	NaN	22.756	NaN	NaN	NaN	NaN	NaN	NaN	NaN	O14595;A0A087W	O14595;A0A087W	Carboxy-terminal	CTDSP2
21.6621	NaN	NaN	22.1005	21.8414	NaN	NaN	NaN	NaN	22.9073	NaN	23.2799	O15194-2;O15194	O15194-2;O15194	CTD small phosph	CTDSPL
21.3287	NaN	NaN	NaN	NaN	NaN	NaN	NaN	NaN	NaN	NaN	NaN	Q05D32;Q05D32-	Q05D32;Q05D32-	CTD small phosph	CTDSPL2
28.8565	28.1418	27.7161	27.9578	26.4829	25.168	29.3206	30.003	27.5137	26.31	25.9442	24.6942	P29279;P29279-2	P29279;P29279-2	Connective tissue	CTGF
24.3124	25.0338	24.5029	23.3247	24.3558	24.0132	NaN	NaN	NaN	NaN	NaN	NaN	P32929;P32929-3,	P32929;P32929-3,	Cystathionine gan	CTH
27.1153	27.2623	28.4677	27.6442	27.6311	28.7059	27.68	27.42	27.3113	28.746	28.6718	28.6804	P35221;P35221-2,	P35221;P35221-2,	Catenin alpha-1	CTNNA1
21.4059	NaN	21.5931	21.4321	NaN	NaN	NaN	NaN	NaN	22.9576	21.4331	NaN	P26232;P26232-2,	P26232;P26232-2,	Catenin alpha-2	CTNNA2
22.3915	NaN	NaN	22.6509	NaN	NaN	NaN	NaN	NaN	NaN	NaN	NaN	Q9UBT7;Q9UBT7-	Q9UBT7;Q9UBT7-	Alpha-catulin	CTNNAL1
27.214	27.2378	28.8927	27.9499	28.0251	29.1344	27.79	27.2813	27.2912	29.2489	29.0191	28.3116	P35222;B4DGU4;E	P35222;B4DGU4	Catenin beta-1	CTNNB1
26.3918	24.5102	25.5081	26.541	25.3338	25.6889	23.6697	NaN	NaN	25.0855	24.9872	NaN	Q8WYA6;A0A087'	Q8WYA6;A0A087'	Beta-catenin-like	CTNNBL1

27.6398	27.8197	28.7224	27.8342	28.0395	29.1487	28.1776	27.4549	27.9621	29.0708	28.5755	28.6463	O60716-3;O6071(	O60716-3;O6071(	Catenin delta-1	CTNND1
28.5838	28.0222	28.0741	28.112	28.0256	28.0254	28.1649	28.1886	28.0669	28.4763	28.5969	28.133	P17812;P17812-2	P17812;P17812-2	CTP synthase 1	CTPS1
23.998	23.7769	23.8685	23.6672	23.8648	23.8057	23.9073	NaN	23.9209	23.7691	23.9097	23.7417	Q9NRF8	Q9NRF8	CTP synthase 2	CTPS2
24.2312	23.5048	23.4047	24.4256	23.5413	24.126	NaN	NaN	NaN	23.8329	NaN	NaN	Q6PD62;H0YCE8	Q6PD62	RNA polymerase-a	CTR9
NaN	NaN	NaN	NaN	NaN	NaN	26.9047	NaN	NaN	NaN	NaN	NaN	P40313;I3NI28;I3I	P40313;I3NI28;I3I	Chymotrypsin-like	CTRL
24.5945	25.3454	24.7466	24.2987	24.6802	24.7445	26.6483	27.1961	26.6565	25.4742	25.5199	26.0753	P10619;X6R5C5;X	P10619;X6R5C5;X	Lysosomal protec	CTSA
23.5189	24.8724	24.3789	23.9	24.2983	24.7324	26.0518	26.4843	25.9032	25.3036	24.7827	24.86	P07858;R4GMQ5;	P07858;R4GMQ5;	Cathepsin B;Cathe	CTSB
25.1198	25.8021	25.1431	24.9213	25.4475	25.0122	27.7251	27.7474	26.1599	26.1706	26.019	25.1934	P53634;H0YCY8;P	P53634;H0YCY8	Dipeptidyl peptida	CTSC
28.5623	29.1791	28.3813	28.1115	28.661	28.3079	30.5355	29.4659	28.806	29.1742	28.7581	28.7577	P07339;C9JH19;F	P07339;C9JH19;F	Cathepsin D;Cathe	CTSD
NaN	NaN	NaN	NaN	NaN	NaN	NaN	23.1565	24.1255	24.4613	23.7308	NaN	Q9UBX1;H0YD65;	Q9UBX1;H0YD65	Cathepsin F	CTSF
23.7367	NaN	NaN	NaN	NaN	NaN	24.8581	NaN	NaN	24.5698	24.0785	NaN	P09668;A0A087X(	P09668;A0A087X(	Pro-cathepsin H;C	CTSH
24.149	26.1641	24.4061	24.2859	25.3548	24.4712	28.0441	28.4217	26.5027	25.5217	24.8383	23.6453	P07711;Q5T8F0;C	P07711;Q5T8F0	Cathepsin L1;Cath	CTSL
25.6873	26.9425	25.4559	25.3547	26.1528	25.1725	28.5116	28.5067	27.1838	26.4826	25.6838	26.4409	Q9UBR2	Q9UBR2	Cathepsin Z	CTSZ
28.2712	28.6358	28.4884	27.8553	28.189	28.489	28.6613	27.8548	26.5433	29.1925	28.6957	28.0458	Q14247;Q14247-3	Q14247;Q14247-3	Src substrate cort	CTTN
23.7019	22.9105	23.3442	23.3835	23.4424	23.3158	NaN	NaN	NaN	NaN	NaN	NaN	Q9P2B4;B1AMN7	Q9P2B4;B1AMN7	CTTNBP2 N-termi	CTTNBP2NL
22.6177	NaN	NaN	22.6567	NaN	NaN	NaN	NaN	NaN	NaN	NaN	NaN	Q7Z7A3	Q7Z7A3	Cytoplasmic tRNA	CTU1
22.1273	NaN	NaN	22.4569	22.2303	NaN	NaN	NaN	NaN	22.8922	22.8953	23.0587	Q2VPK5;H3BSW6;	Q2VPK5;H3BSW6;	Cytoplasmic tRNA	CTU2
26.2429	25.9006	26.3366	26.3432	26.1005	26.5344	26.5437	26.0668	26.2599	26.4885	26.528	26.7869	Q13616;A0A0C4D	Q13616;A0A0C4D	Cullin-1	CUL1
26.7604	26.5718	26.7985	27.0842	27.1977	27.1033	26.1393	26.7959	27.105	27.2559	27.3259	27.6016	Q13617;A0A0A0M	Q13617;A0A0A0M	Cullin-2	CUL2
25.4818	25.182	25.7033	25.4329	25.9774	25.7643	25.4978	25.7776	25.2938	25.5584	25.9301	25.8659	Q13618;Q13618-2	Q13618;Q13618-2	Cullin-3	CUL3
24.6387	NaN	23.5975	24.7903	24.2775	23.8063	NaN	NaN	NaN	24.6877	NaN	NaN	Q13619;A0A0A0M	Q13619;A0A0A0M	Cullin-4A	CUL4A
25.8249	25.2919	25.6852	25.7359	25.0122	25.5425	25.4	24.9119	NaN	25.9242	25.4274	25.023	Q13620-1;Q1362(	Q13620-1;Q1362(	Cullin-4B	CUL4B
26.2156	26.3747	26.8225	26.4139	26.8246	27.001	26.4351	25.8637	26.2224	27.2274	26.7179	26.6795	Q93034;H0YCA0;E	Q93034	Cullin-5	CUL5
22.0483	NaN	21.9417	22.6832	NaN	22.2477	23.0925	NaN	NaN	22.531	NaN	NaN	Q14999;Q14999-2	Q14999;Q14999-2	Cullin-7	CUL7
25.9065	27.3085	26.2514	25.7244	26.7477	26.3305	27.6621	27.8663	27.0245	26.1722	27.1723	26.9563	O60888;O60888-3	O60888;O60888-3	Protein CutA	CUTA
NaN	NaN	21.9394	20.9479	NaN	NaN	NaN	NaN	NaN	NaN	NaN	NaN	P39880-9;Q13948	P39880-9;Q13948	Homeobox protei	CUX1
24.2638	23.1042	23.8394	24.6834	23.0816	22.8443	NaN	NaN	NaN	23.1096	NaN	NaN	Q9HCG8;B7WP74	Q9HCG8;B7WP74	Pre-mRNA-splicing	CWC22
22.7105	23.6409	23.0151	23.066	23.5869	NaN	NaN	NaN	NaN	23.2271	NaN	NaN	Q6UX04;D6REK3;(	Q6UX04;D6REK3;(	Peptidyl-prolyl cis	CWC27
22.0992	NaN	22.0823	22.1645	NaN	22.1168	NaN	NaN	NaN	22.2211	21.8134	21.6322	Q69YN2;Q69YN2-	Q69YN2;Q69YN2-	CWF19-like protei	CWF19L1
NaN	NaN	NaN	21.2933	NaN	NaN	NaN	NaN	NaN	NaN	NaN	NaN	P78310;P78310-5	P78310;P78310-5	Coxsackievirus an	CXADR
23.5919	NaN	NaN	NaN	NaN	NaN	NaN	NaN	NaN	NaN	NaN	NaN	P09341;P19875	P09341;P19875	Growth-regulated	CXCL1;CXCL2
NaN	25.3859	NaN	24.2643	NaN	NaN	NaN	NaN	NaN	NaN	NaN	NaN	C9J4T6;P10145	C9J4T6;P10145	C-X-C motif chem	CXCL8
24.3635	24.2049	24.1196	24.1887	23.8196	24.1127	NaN	NaN	NaN	NaN	NaN	NaN	P61073;P61073-2	P61073;P61073-2	C-X-C chemokine	CXCR4
NaN	NaN	NaN	21.1642	NaN	NaN	NaN	NaN	NaN	NaN	NaN	NaN	O14569;U3KQU4	O14569;U3KQU4	Cytochrome b561	CYB561D2
NaN	NaN	21.8007	NaN	NaN	NaN	NaN	NaN	NaN	NaN	NaN	NaN	P00167;P00167-2	P00167;P00167-2	Cytochrome b5	CYB5A
25.848	24.6907	24.8891	24.9317	25.2388	25.3724	25.8267	26.4804	26.5516	25.0526	24.983	24.8986	J3KNF8;O43169;H	J3KNF8;O43169;H	Cytochrome b5 ty	CYB5B
NaN	NaN	NaN	23.8768	NaN	NaN	NaN	NaN	NaN	NaN	NaN	NaN	Q9UHQ9;H7C0R7	Q9UHQ9;H7C0R7	NADH-cytochrom	CYB5R1
27.6812	27.6523	27.9358	28.0575	27.7784	28.0441	28.1746	28.4769	28.9458	28.1248	27.8977	27.8565	P00387;P00387-2	P00387;P00387-2	NADH-cytochrom	CYB5R3
26.6158	NaN	26.3375	26.7739	26.1428	26.3341	NaN	NaN	NaN	NaN	NaN	NaN	Q7L1T6;A0A0A0M	Q7L1T6	Cytochrome b5 re	CYB5R4
25.5823	24.7127	25.8927	25.3608	24.7514	25.9386	24.8003	NaN	NaN	24.9868	24.9491	NaN	P13498	P13498	Cytochrome b-24!	CYBA
25.4546	NaN	NaN	NaN	NaN	NaN	25.4382	NaN	NaN	NaN	NaN	NaN	Q53TN4;Q53TN4-	Q53TN4;Q53TN4-	Cytochrome b red	CYBRD1
24.4863	25.0378	25.0246	25.3788	25.4127	25.1171	23.7622	25.6969	26.1666	24.2453	24.9521	24.521	P08574	P08574	Cytochrome c1, h	CYC1
27.2357	28.1059	27.5985	27.434	27.974	27.461	27.2464	27.8751	28.2374	27.4045	27.5455	28.0543	P99999;C9JFR7;C(	P99999;C9JFR7	Cytochrome c	CYCS
29.0765	28.5575	29.2078	28.9199	28.8397	29.2435	29.2341	29.103	29.0086	29.5352	29.4995	28.9945	Q7L576;A0A0G2JI	Q7L576;A0A0G2JI	Cytoplasmic FMR	CYFIP1
NaN		NaN	NaN	23.7341	NaN	NaN	NaN	NaN	NaN	NaN	NaN	E7EVJ5;Q96F07-2;	E7EVJ5;Q96F07-2;	Cytoplasmic FMR	CYFIP2
21.1139	NaN	NaN	NaN	NaN	NaN	NaN	NaN	NaN	NaN	NaN	NaN	Q6UW02;E9PHG5	Q6UW02;E9PHG5	Cytochrome P450	CYP20A1
22.4493	NaN	22.5086	22.7781	22.38	23.1013	23.1717	23.9195	23.8577	23.1931	23.3193	NaN	Q16850;A0A0C4D	Q16850;A0A0C4D	Lanosterol 14-alpl	CYP51A1
30.9439	29.9705	31.1002	29.7358	29.4229	29.8383	33.8971	34.4207	31.7915	31.1042	30.9648	29.8248	O00622;A0A087W	O00622;A0A087W	Protein CYR61	CYR61
NaN	NaN	NaN	22.2576	NaN	NaN	NaN	NaN	NaN	NaN	NaN	NaN	A8MQ03;B8A4K4	A8MQ03;B8A4K4	Cysteine-rich tail	CYSRT1
22.3464	NaN	22.5939	21.8483	NaN	NaN	NaN	NaN	NaN	NaN	NaN	NaN	Q9Y4D1;Q9Y4D1-	Q9Y4D1;Q9Y4D1-	Disheveled-associ	DAAM1
NaN	24.6802	23.5419	23.4821	24.1701	23.4278	NaN	NaN	NaN	NaN	NaN	NaN	P98082;P98082-3	P98082;P98082-3	Disabled homolog	DAB2
NaN	22.6216	22.9196	NaN	NaN	NaN	23.0095	NaN	NaN	NaN	NaN	22.4409	P61803;F5GXX5;F	P61803;F5GXX5;F	Dolichyl-diphosph	DAD1
25.7639	25.9404	25.6153	25.5028	25.367	26.2291	28.0149	27.2888	26.4638	26.9144	25.98	26.4613	Q14118;C9JYS1;C	Q14118	Dystroglycan;Alph	DAG1
26.08	25.2471	25.5851	25.9693	25.1407	25.7254	25.3577	25.4583	25.4578	25.1019	24.965	25.2271	Q8NCG7;Q8NCG7	Q8NCG7;Q8NCG7	Sn1-specific diacy	DAGLB
22.3206	22.1985	22.7926	22.6882	22.8659	NaN	23.128	NaN	NaN	23.0348	22.7452	NaN	Q3LXA3;A0A087W	Q3LXA3;A0A087W	Bifunctional ATP-c	DAK;TKFC
NaN	NaN	NaN	NaN	NaN	NaN	25.0513	NaN	NaN	NaN	NaN	NaN	P51397;B4DQ75	P51397;B4DQ75	Death-associated	DAP
21.7425	NaN	NaN	22.6436	21.8055	21.4829	NaN	NaN	NaN	NaN	NaN	NaN	V9GZ03;P51398;P	V9GZ03;P51398;P	28S ribosomal pro	DAP3
25.7282	26.3679	26.0958	26.1987	26.17	26.1787	26.5638	27.0706	26.6931	26.0483	26.0143	25.3559	O43293;O43293-2	O43293	Death-associated	DAPK3
31.1982	30.3735	30.4756	30.9507	30.4564	30.605	29.9131	30.401	29.8829	30.772	30.7191	30.0781	P14868;P14868-2	P14868;P14868-2	Aspartate--tRNA l	DARS
25.8312	24.4009	24.8631	26.0527	24.5968	24.688	NaN	NaN	NaN	24.577	24.3973	23.6018	Q6PI48	Q6PI48	Aspartate--tRNA l	DARS2

23.6823	NaN	23.4278	23.7436	23.3659	NaN	NaN	NaN	NaN	23.2142	23.3727	NaN	Q9UER7;Q9UER7-	Q9UER7;Q9UER7-	Death domain-ass	DAXX
24.4753	24.6731	23.8273	24.5622	24.5987	24.1525	23.894	24.2252	23.9006	24.1831	24.4966	25.0944	Q96EP5;K7EQ02;K	Q96EP5;K7EQ02;K	DAZ-associated pr	DAZAP1
25.5519	25.2604	24.9442	26.0638	25.362	24.6915	25.3796	25.0028	24.0622	26.2461	25.642	NaN	P07108;P07108-5	P07108;P07108-5	Acyl-CoA-binding	DBI
28.2984	28.6915	28.6812	28.2261	28.5817	28.8272	28.971	28.1402	28.3002	29.6167	29.2218	29.5781	Q16643;Q16643-3	Q16643;Q16643-3	Drebrin	DBN1
27.711	28.1344	28.0032	27.727	27.9893	27.9174	27.803	27.647	27.8902	28.0067	27.9473	28.0042	Q9UJU6;H0Y5J4;C	Q9UJU6;H0Y5J4;C	Drebrin-like prote	DBNL
NaN	NaN	NaN	21.9744	NaN	NaN	NaN	NaN	NaN	NaN	NaN	NaN	Q9UJU6-2;Q9UJU	Q9UJU6-2;Q9UJU	Drebrin-like prote	DBNL
24.2907	NaN	23.9406	24.0927	NaN	23.5623	NaN	NaN	23.3496	24.1405	24.3187	NaN	Q9UK59;Q9UK59-	Q9UK59	Lariat debranchin	DBR1
23.166	NaN	NaN	23.1228	NaN	NaN	NaN	NaN	NaN	NaN	NaN	NaN	Q8TEB1;Q8TEB1-2	Q8TEB1;Q8TEB1-2	DDB1- and CUL4-a	DCAF11
23.5664	NaN	NaN	NaN	NaN	NaN	NaN	NaN	NaN	NaN	NaN	NaN	Q9NV06;B3KME9;	Q9NV06;B3KME9;	DDB1- and CUL4-a	DCAF13
22.6973	NaN	NaN	NaN	NaN	NaN	NaN	NaN	NaN	NaN	NaN	NaN	Q9NXF7	Q9NXF7	DDB1- and CUL4-a	DCAF16
25.5354	24.8538	24.943	24.8242	24.7153	24.1362	24.4566	NaN	NaN	24.8743	24.9619	24.1188	P61962;A0A087W	P61962;A0A087W	DDB1- and CUL4-a	DCAF7
25.4663	24.1328	24.1573	25.1964	24.1287	24.2489	24.3397	24.7818	NaN	NaN	24.4665	NaN	Q5TAQ9;G3V3G9;	Q5TAQ9;G3V3G9;	DDB1- and CUL4-a	DCAF8
NaN	NaN	NaN	NaN	NaN	NaN	22.7369	NaN	NaN	NaN	NaN	NaN	Q8N8Z6;Q8N8Z6-	Q8N8Z6;Q8N8Z6-	Discoidin, CUB an	DCBLD1
25.2973	25.3822	25.384	24.9642	25.123	25.642	26.1858	25.2449	25.2073	25.8895	25.5809	25.7138	Q96PD2;Q96PD2-	Q96PD2;Q96PD2-	Discoidin, CUB an	DCBLD2
23.6326	27.0645	24.683	24.0082	24.5318	24.2663	26.0697	25.5153	26.9661	25.6795	25.5571	26.0824	P81605;P81605-2	P81605;P81605-2	Dermcidin;Surviva	DCD
26.6102	26.4034	25.9225	26.3334	26.1981	25.8107	25.2711	25.5075	25.7617	26.8033	26.9759	25.5676	P27707;D6RFG8;D	P27707;D6RFG8;D	Deoxycytidine kin	DCK
25.423	24.5087	24.4619	25.2094	24.3816	24.4005	24.139	24.4718	NaN	24.2321	24.7157	24.3237	Q9NPI6;Q9NPI6-2	Q9NPI6;Q9NPI6-2	mRNA-decapping	DCP1A
25.5065	25.0324	24.8669	25.1818	25.434	25.1411	24.7444	NaN	NaN	25.5492	25.6597	25.4291	Q96C86	Q96C86	m7GpppX diphos	DCPS
24.518	25.2697	25.1834	25.444	25.2283	25.2173	25.293	25.2647	25.6597	25.5754	25.4862	25.7673	P32321;P32321-2	P32321;P32321-2	Deoxycytidylate d	DCTD
28.2449	28.795	28.6493	28.2509	28.7827	28.6131	28.7894	29.4621	28.8128	28.6013	29.0876	28.9609	E7EX90;Q14203-3	E7EX90;Q14203-3	Dynactin subunit	DCTN1;DKFZp686
27.7617	28.659	28.0124	27.3702	28.2502	27.9943	28.0048	29.0751	28.4343	27.4322	27.9375	28.2489	Q13561;Q13561-3	Q13561;Q13561-3	Dynactin subunit	DCTN2
26.3449	26.7735	26.3016	26.5086	26.5455	26.5685	27.1612	27.2436	25.689	27.1745	26.6927	26.8884	O75935;O75935-3	O75935;O75935-3	Dynactin subunit	DCTN3
25.7055	25.7473	25.4406	25.099	25.9606	25.5977	25.9021	26.2455	25.9083	25.784	25.8598	26.0134	Q9UJW0;Q9UJW0	Q9UJW0;Q9UJW0	Dynactin subunit	DCTN4
22.8469	23.1455	22.9396	22.5751	23.4658	NaN	NaN	NaN	NaN	23.2141	23.6844	NaN	Q9BTE1;H3BUD2;	Q9BTE1;H3BUD2;	Dynactin subunit	DCTN5
24.974	25.0172	25.0551	24.7472	25.3956	25.2958	25.2548	25.9593	24.9627	24.5275	NaN	NaN	O00399;E5RK00	O00399	Dynactin subunit	DCTN6
25.162	25.5263	24.9149	24.6406	24.7362	24.8758	24.9785	25.0638	24.3723	25.0403	25.059	24.5711	Q9H773	Q9H773	dCTP pyrophosph	DCTPP1
22.6878	NaN	NaN	22.0726	NaN	NaN	NaN	NaN	NaN	NaN	NaN	NaN	Q96GG9;C9JVE2;C	Q96GG9;C9JVE2;C	DCN1-like protein	DCUN1D1
22.3281	23.8856	24.625	24.0174	23.7966	24.8689	23.7987	23.5312	23.4096	25.3608	24.6996	24.3521	Q8IWE4	Q8IWE4	DCN1-like protein	DCUN1D3
25.5672	24.3863	24.9856	25.8634	24.9208	25.0566	24.1194	NaN	NaN	24.3834	25.2193	NaN	Q9BTE7;H0YCN4;I	Q9BTE7;H0YCN4;I	DCN1-like protein	DCUN1D5
26.2899	25.7122	25.4076	26.0821	25.9242	25.4823	24.7871	24.2994	24.9675	25.5038	25.6127	24.8389	Q7Z4W1;J3KS22;J	Q7Z4W1;J3KS22;J	L-xylulose reducta	DCXR
22.9617	NaN	NaN	NaN	NaN	NaN	NaN	NaN	NaN	NaN	NaN	NaN	Q9BW61	Q9BW61	DET1- and DDB1-a	DDA1
28.5468	29.6986	29.2216	28.5944	29.6552	29.4422	29.2981	29.2884	28.9876	29.577	29.4841	29.827	O94760;B4DYP1;C	O94760;B4DYP1;C	N(G),N(G)-dimeth	DDAH1
24.7722	26.3481	25.9553	24.9594	26.3368	26.1414	25.8287	25.9555	25.742	25.9607	26.0856	26.5003	O95865;Q5SSV3;C	O95865;Q5SSV3;C	N(G),N(G)-dimeth	DDAH2
28.6207	28.712	28.7108	28.6244	28.6522	28.8226	28.5604	28.8884	28.2083	28.721	28.9194	28.8221	Q16531;F5GY55;C	Q16531;F5GY55	DNA damage-binc	DDB1
NaN	NaN	NaN	21.2804	NaN	NaN	NaN	NaN	NaN	NaN	NaN	NaN	Q8NEL9;A0A087X	Q8NEL9;A0A087X	Phospholipase DD	DDHD1
27.0701	26.9033	27.0473	27.4278	26.9714	27.3148	26.9628	26.8751	27.8045	26.9801	26.9589	27.07	P39656;A0A0C4D	P39656;A0A0C4D	Dolichyl-diphosph	DDOST
24.1185	23.7033	24.4678	24.0509	NaN	NaN	23.6099	NaN	NaN	24.1877	24.0271	NaN	Q96HY6;A0A0A0M	Q96HY6;A0A0A0M	DDRGK domain-co	DDRGK1
26.522	26.9637	26.879	26.4345	26.6179	26.8363	26.9933	25.7926	26.6909	26.9956	26.5165	26.6094	P30046;A6NHG4;J	P30046;A6NHG4;J	D-dopachrome de	DDT;DDTL
28.0498	28.3321	28.1259	28.1482	28.5867	28.0435	28.0971	28.3551	28.5433	28.472	28.5389	28.7235	Q92499;F1T0B3;A	Q92499;F1T0B3;A	ATP-dependent R	DDX1
23.7582	NaN	22.9562	24.0109	NaN	23.2432	NaN	NaN	NaN	NaN	NaN	NaN	Q13206;E9PIF2	Q13206;E9PIF2	Probable ATP-dep	DDX10
27.8143	27.1462	27.228	27.9978	27.4567	27.4859	26.76	26.887	26.567	28.0288	28.1967	27.9921	Q92841;H3BLZ8;C	Q92841;H3BLZ8;C	Probable ATP-dep	DDX17
27.2856	25.3806	27.741	28.0166	26.7905	27.8062	24.7921	25.1238	23.9518	27.4107	26.9143	26.6984	Q9NVP1;H7C452	Q9NVP1	ATP-dependent R	DDX18
26.7703	26.3243	26.2808	26.5037	26.3092	26.638	26.5932	26.3823	26.5353	26.9595	27.058	26.7834	Q9NUU7;I3L0H8;I	Q9NUU7;I3L0H8;I	ATP-dependent R	DDX19A;DDX19B
27.948	26.3009	27.1576	28.0782	26.3414	26.9085	24.8411	24.7308	23.9643	26.0041	26.5895	24.3758	Q9UHI6;E9PJ60;Q	Q9UHI6	Probable ATP-dep	DDX20
29.8949	28.7743	29.3783	30.3761	29.3502	29.5996	27.7412	28.5038	28.0129	29.0716	29.4706	29.0903	Q9NR30;Q9NR30-	Q9NR30;Q9NR30-	Nucleolar RNA he	DDX21
24.3028	23.9536	24.4415	24.6683	24.4177	24.6372	24.2966	NaN	NaN	24.4381	24.6526	NaN	Q9BUQ8;H0YI52	Q9BUQ8	Probable ATP-dep	DDX23
26.3358	24.6099	24.551	26.4288	25.2173	23.447	NaN	NaN	NaN	22.8513	23.1105	23.4774	Q9GZR7;G3V529;C	Q9GZR7;G3V529;C	ATP-dependent R	DDX24
26.1221	24.1043	24.4319	25.7092	24.8206	24.5434	NaN	NaN	NaN	23.0487	24.9932	NaN	Q96GQ7;B7Z6D5;	Q96GQ7;B7Z6D5	Probable ATP-dep	DDX27
29.6218	28.6751	29.3205	30.0628	29.1436	29.5493	28.2485	28.3296	28.7406	29.4757	29.6532	29.6594	O00148;O00148-2	O00148;O00148-2	ATP-dependent R	DDX39A
26.8917	26.2278	26.9096	27.4084	26.7082	26.8102	25.961	26.034	26.0911	26.4372	27.0471	27.6317	Q13838;Q13838-2	Q13838;Q13838-2	Spliceosome RNA	DDX39B
30.4292	29.6045	29.7864	30.4054	29.7412	29.7048	29.749	30.1522	29.4425	30.0077	29.8968	29.7069	O00571;A0A0D9S	O00571;A0A0D9S	ATP-dependent R	DDX3X;DDX3Y
24.6995	NaN	23.9911	24.9553	24.2513	24.0294	NaN	NaN	NaN	24.6379	24.5735	24.2613	Q9UJV9;J3KNN5;I	Q9UJV9;J3KNN5	Probable ATP-dep	DDX41
23.2219	23.4564	23.7314	23.3574	23.6426	24.0399	NaN	NaN	NaN	23.8313	24.1329	24.2022	Q86XP3;A0A0A0M	Q86XP3;A0A0A0M	ATP-dependent R	DDX42
28.7572	27.7352	27.953	28.5916	27.9912	28.0837	26.5792	26.7894	27.3241	27.6042	27.9975	27.7971	Q7L014;A0A0C4D	Q7L014;A0A0C4D	Probable ATP-dep	DDX46
27.3415	25.0059	25.7666	27.3589	25.8755	26.0494	24.471	24.4153	23.2982	25.6961	26.3057	24.2999	Q9H0S4;Q9H0S4-2	Q9H0S4;Q9H0S4-2	Probable ATP-dep	DDX47
24.6668	NaN	22.3098	23.967	22.0333	22.1579	NaN	NaN	NaN	21.8477	NaN	NaN	Q9Y6V7;Q9Y6V7-2	Q9Y6V7	Probable ATP-dep	DDX49
30.0906	29.4485	29.6925	30.4299	29.7775	30.0351	28.4804	28.7453	28.1249	29.8715	29.9268	29.3819	P17844;J3KTA4;P	P17844;J3KTA4;P	Probable ATP-dep	DDX5
26.2686	25.532	26.0483	27.1648	26.1306	26.2446	NaN	25.673	24.0426	25.0522	25.6323	25.0388	Q9BQ39;A0A087W	Q9BQ39;A0A087W	ATP-dependent R	DDX50
20.9835	NaN	NaN	NaN	NaN	NaN	NaN	NaN	NaN	NaN	NaN	NaN	Q8N8A6	Q8N8A6	ATP-dependent R	DDX51
23.0247	21.9891	22.5945	23.8714	22.4088	NaN	NaN	NaN	NaN	NaN	NaN	NaN	Q9Y2R4;A0A087X	Q9Y2R4	Probable ATP-dep	DDX52

26.0536	24.2688	24.7482	25.9791	24.6515	24.6694	22.5409	23.7107	NaN	23.6528	23.792	NaN	Q8TDD1;Q8TDD1-	Q8TDD1;Q8TDD1-	ATP-dependent R	DDX54
25.4618	23.1028	24.5164	25.8949	23.5959	24.4678	22.9352	NaN	23.1981	23.3791	23.6717	22.7831	Q8NHQ9;F5H5U2	Q8NHQ9;F5H5U2	ATP-dependent R	DDX55
25.1495	23.8602	24.3351	25.258	25.0392	24.4284	NaN	NaN	NaN	23.556	NaN	NaN	Q9NY93;Q9NY93-	Q9NY93;Q9NY93-	Probable ATP-dep	DDX56
25.7924	24.7488	25.8562	26.0738	25.0989	25.737	22.5502	NaN	24.2536	24.1706	24.289	23.6146	O95786;O95786-2	O95786;O95786-2	Probable ATP-dep	DDX58
28.0443	26.9095	27.2982	27.6741	26.71	26.8096	26.7872	24.0601	25.6063	27.1395	26.6661	26.0603	P26196;Q8IV96	P26196	Probable ATP-dep	DDX6
26.4631	26.5049	26.0606	26.0838	26.3299	26.0323	24.2356	25.3353	25.6806	25.8621	26.4314	25.2348	Q16698;Q16698-2	Q16698;Q16698-2	2,4-dienoyl-CoA re	DECR1
25.7287	25.8991	26.7036	25.7527	26.1625	26.9406	25.0346	NaN	24.2807	26.2494	26.1795	26.2002	Q9H4E7;Q6TDU7;	Q9H4E7	Differentially expr	DEF6
NaN	NaN	NaN	NaN	NaN	NaN	NaN	NaN	NaN	22.5091	NaN	NaN	O15121	O15121	Sphingolipid delta	DEGS1
28.407	26.5188	27.1148	28.6642	26.9654	27.5771	26.8585	25.634	25.4478	27.2677	27.5947	26.6831	P35659;B4DFG0;P	P35659;B4DFG0;P	Protein DEK	DEK
NaN	NaN	NaN	22.3823	NaN	NaN	NaN	NaN	NaN	NaN	NaN	NaN	Q8IWF6	Q8IWF6	Protein DENND6A	DENND6A
26.0301	25.5463	25.1949	24.9317	24.8139	24.805	25.3536	25.2589	NaN	24.5676	24.9989	25.5041	O43583;F8VVL1	O43583;F8VVL1	Density-regulated	DENR
NaN	NaN	NaN	NaN	NaN	NaN	NaN	NaN	NaN	24.0869	NaN	NaN	Q5TB30;Q5TB30-2	Q5TB30;Q5TB30-2	DEP domain-conta	DEPDC1
NaN	NaN	NaN	NaN	NaN	NaN	NaN	NaN	NaN	23.7121	22.6982	NaN	Q8WUY9;Q8WUY	Q8WUY9;Q8WUY	DEP domain-conta	DEPDC1B
24.6669	24.2819	24.9777	24.5171	24.9083	24.7824	24.247	24.7046	25.2104	24.4089	24.6395	24.6318	Q9Y315;G3V158;E	Q9Y315;G3V158;E	Deoxyribose-phos	DERA
24.6753	24.0156	24.0483	24.5486	23.7067	NaN	24.0216	NaN	24.1835	23.8987	23.2791	24.0642	Q9BUN8;B4E1G1;	Q9BUN8;B4E1G1;	Derlin-1	DERL1
23.4261	23.5424	24.1125	24.0469	23.8089	24.2022	23.5541	24.6354	24.8366	23.8209	23.7507	24.4633	Q9GZP9;I3L1T3	Q9GZP9;I3L1T3	Derlin-2	DERL2
23.7282	23.7898	23.0684	23.6297	23.5548	NaN	NaN	NaN	NaN	23.8219	24.5745	NaN	O00273;O00273-2	O00273;O00273-2	DNA fragmentatio	DFFA
26.8557	26.6188	26.7489	26.9922	26.5681	26.8134	26.1193	25.8349	26.2236	26.5142	26.6976	26.598	O60443;H7C3W1;	O60443;H7C3W1	Non-syndromic he	DFNA5
23.1271	22.9281	23.3069	23.2891	23.7351	NaN	NaN	NaN	NaN	NaN	23.5023	NaN	Q13574-7;Q1357	Q13574-7;Q1357	Diacylglycerol kin	DGKZ
22.3449	NaN	22.1944	NaN	NaN	NaN	NaN	NaN	NaN	22.254	NaN	NaN	Q16854;Q16854-2	Q16854;Q16854-2	Deoxyguanosine k	DGUOK
25.2777	24.0049	24.8105	25.6425	24.539	24.8221	26.5203	NaN	23.7484	26.4732	25.1595	24.8326	Q15392;Q3LIE7;Q	Q15392;Q3LIE7;Q	Delta(24)-sterol re	DHCR24;Nbla0364
26.0348	25.6748	25.652	26.1669	25.2441	25.6845	26.712	26.8921	26.6711	27.128	26.2652	NaN	Q9UBM7;E9PM0	Q9UBM7	7-dehydrocholest	DHCR7
25.4203	25.3018	25.597	25.3085	25.5177	25.7246	24.5	NaN	25.0179	25.6073	26.1695	25.3875	P00374;P00374-2	P00374;P00374-2	Dihydrofolate red	DHFR
25.075	25.5569	25.2876	24.9354	25.5023	25.2913	25.6082	25.9094	25.4359	25.744	25.9218	26.0116	P49366;A0A087W	P49366;A0A087W	Deoxyhypusine sy	DHPS
22.2052	22.2549	22.6278	22.9318	23.1291	NaN	22.6741	NaN	NaN	22.7595	22.7154	NaN	Q96LJ7;H0YNC2	Q96LJ7;H0YNC2	Dehydrogenase/r	DHRS1
23.7745	24.0214	23.9513	24.1374	24.0018	24.0669	23.0878	23.7472	23.9027	23.4726	24.3345	23.5662	Q9BTZ2;Q9BTZ2-8	Q9BTZ2;Q9BTZ2-8	Dehydrogenase/r	DHRS4
23.7718	NaN	23.5491	24.0769	22.8561	NaN	NaN	NaN	NaN	24.1761	NaN	NaN	Q9Y394;Q9Y394-2	Q9Y394;Q9Y394-2	Dehydrogenase/r	DHRS7
24.2043	24.034	24.321	24.5218	24.3056	24.3696	24.8899	25.2593	24.9167	24.6372	24.5346	24.6897	Q6IAN0;A0A0C4D	Q6IAN0;A0A0C4D	Dehydrogenase/r	DHRS7B
29.7061	28.9361	29.3904	29.9422	29.4314	29.6131	28.5706	28.877	29.0268	29.8609	29.8334	29.7866	O43143	O43143	Pre-mRNA-splicin	DHX15
25.5857	24.246	25.1601	25.9377	25.1531	25.2991	27.5524	NaN	23.7481	25.807	26.1943	25.4715	O60231;Q5SQH5;	O60231;Q5SQH5	Putative pre-mRN	DHX16
27.1352	26.1406	26.5143	26.9037	26.3212	26.1885	25.6901	25.089	23.7077	26.4811	26.2381	25.6372	Q7Z478;A0A087W	Q7Z478;A0A087W	ATP-dependent R	DHX29
28.5233	26.6546	27.1444	28.8776	27.5032	27.2996	25.0152	24.6669	25.6809	26.8445	27.2222	25.4966	Q7L2E3;Q7L2E3-2	Q7L2E3;Q7L2E3-2	Putative ATP-dep	DHX30
24.3554	NaN	24.3788	23.8764	23.5972	23.3777	NaN	NaN	NaN	23.7447	NaN	NaN	Q7L7V1;Q7L7V1-2	Q7L7V1;Q7L7V1-2	Putative pre-mRN	DHX32
24.1168	NaN	NaN	24.0433	NaN	NaN	NaN	NaN	NaN	22.705	NaN	NaN	Q9H6R0;I3L1L6;Q	Q9H6R0;I3L1L6	Putative ATP-dep	DHX33
22.4977	NaN	21.7925	NaN	NaN	NaN	NaN	NaN	NaN	NaN	21.9686	NaN	Q9H5Z1;Q5THR1;	Q9H5Z1;Q5THR1;	Probable ATP-dep	DHX35
24.0309	NaN	23.6438	24.1109	23.4202	23.4382	NaN	NaN	NaN	24.122	23.7906	24.0556	Q9H2U1;Q9H2U1	Q9H2U1;Q9H2U1	ATP-dependent R	DHX36
24.2006	23.157	23.8366	23.9592	23.4989	23.6934	23.7759	24.1815	NaN	24.4576	24.0595	24.0148	Q92620;Q92620-2	Q92620;Q92620-2	Pre-mRNA-splicin	DHX38
NaN	NaN	23.0115	NaN	NaN	NaN	NaN	NaN	NaN	NaN	NaN	NaN	Q6P158;A0A087W	Q6P158	Putative ATP-dep	DHX57
21.865	NaN	NaN	NaN	NaN	NaN	NaN	NaN	NaN	NaN	NaN	NaN	Q14562;F5H658	Q14562;F5H658	ATP-dependent R	DHX8
31.1834	29.6143	30.1179	31.2315	30.1894	30.4055	29.2533	29.7285	29.0842	30.8625	30.7722	30.1224	Q08211;Q08211-2	Q08211	ATP-dependent R	DHX9
23.3059	NaN	NaN	23.0976	NaN	NaN	NaN	NaN	NaN	23.6205	23.334	NaN	Q9NR28;Q9NR28-	Q9NR28;Q9NR28-	Diablo homolog, r	DIABLO
28.6126	28.2309	28.4779	28.4123	28.3598	28.4772	28.6186	28.7184	28.4493	28.4902	28.6014	28.6658	O60610;A0A0G2J	O60610;A0A0G2J	Protein diaphano	DIAPH1
27.0781	25.9518	26.6996	26.8682	25.8313	26.2621	26.2664	26.3374	25.9496	26.6583	26.0759	25.0359	Q9NSV4;Q9NSV4-	Q9NSV4;Q9NSV4-	Protein diaphano	DIAPH3
23.5868	NaN	23.1817	23.3198	22.7459	NaN	NaN	NaN	NaN	NaN	NaN	NaN	Q9UPY3;Q9UPY3-	Q9UPY3;Q9UPY3-	Endoribonuclease	DICER1
26.4077	26.0458	26.3567	26.4276	26.5995	27.4188	NaN	NaN	NaN	NaN	NaN	NaN	Q9BTC0;Q9BTC0-	Q9BTC0;Q9BTC0-	Death-inducer obl	DIDO1
25.9558	24.2381	24.7422	25.9952	24.6823	24.8803	24.6081	NaN	NaN	24.8104	24.8976	NaN	Q9UNQ2;A0A0C4	Q9UNQ2;A0A0C4	Probable dimethy	DIMT1
24.5811	24.1666	25.3176	24.6273	24.263	25.1822	24.7414	NaN	24.4558	25.307	24.8742	25.0948	Q14689;Q14689-6	Q14689;Q14689-6	Disco-interacting	DIP2A
28.4477	28.3453	28.8737	28.3109	28.2175	29.0349	28.2187	27.1747	28.4016	28.8989	28.4644	28.6672	Q9P265;H0YIU5;H	Q9P265	Disco-interacting	DIP2B
24.5998	25.3489	25.4235	25.735	25.8537	NaN	NaN	NaN	NaN	25.4103	26.3108	25.1966	Q9Y2E4;E7EPU2;A	Q9Y2E4;E7EPU2;A	Disco-interacting	DIP2C
27.0115	25.6844	26.0459	26.9646	26.4245	26.6683	25.3522	23.7687	23.5384	26.9714	26.315	26.1263	Q9Y2L1;G3V1J5;Q	Q9Y2L1;G3V1J5;Q	Exosome complex	DIS3
22.5895	NaN	NaN	22.457	NaN	NaN	NaN	NaN	NaN	NaN	NaN	NaN	Q8TF46;Q8TF46-3	Q8TF46;Q8TF46-3	DIS3-like exonucle	DIS3L
24.5321	23.7719	24.2413	24.4863	24.1633	24.2757	24.1499	NaN	24.1708	24.5655	23.9777	24.4515	Q8IYB7;H7C440;Q	Q8IYB7;H7C440;Q	DIS3-like exonucle	DIS3L2
NaN	NaN	21.4814	NaN	NaN	NaN	NaN	NaN	NaN	NaN	NaN	NaN	A7MBM2	A7MBM2	Protein dispatche	DISP2
28.2236	27.249	27.9393	29.0471	28.1609	28.0703	25.8889	25.9518	25.8428	26.986	27.3274	27.0207	O60832;O60832-2	O60832;O60832-2	H/ACA ribonucleo	DKC1
23.6437	27.2368	26.1848	23.4012	26.0459	24.515	28.9887	29.3273	28.387	23.2043	25.7661	26.254	O94907;Q9UBU2;	O94907	Dickkopf-related p	DKK1
24.9619	25.2756	25.1356	25.6036	25.454	25.4501	24.4814	24.9332	26.6013	23.4246	24.3588	25.4565	P10515;H0YDD4;E	P10515;H0YDD4;E	Dihydrolipoyllysin	DLAT
26.4609	28.0879	26.9357	26.3403	27.7981	26.8454	26.6375	28.1388	28.7614	25.9855	26.7162	27.7436	P09622;E9PEX6;P	P09622;E9PEX6;P	Dihydrolipoyl deh	DLD
28.1881	28.5313	28.7864	28.3271	28.4762	29.0191	29.0309	27.9369	28.0021	29.3849	28.6847	28.4929	Q12959-2;A0A0C4	Q12959-2;A0A0C4	Disks large homol	DLG1
NaN	23.4566	23.7716	22.7266	23.4909	24.0682	NaN	NaN	NaN	23.3224	NaN	NaN	Q92796;Q92796-2	Q92796;Q92796-2	Disks large homol	DLG3
NaN	NaN	NaN	NaN	NaN	NaN	NaN	NaN	NaN	23.5095	24.0015	NaN	Q8TDM6;Q8TDM	Q8TDM6;Q8TDM	Disks large homol	DLG5

25.1147	24.1798	24.2709	24.3689	24.0674	24.2911	24.3305	24.105	NaN	23.808	23.8935	23.4095	Q15398;Q15398-3	Q15398;Q15398-3	Disks large-associ	DLGAP5
26.3395	27.4404	26.7807	27.047	27.3387	26.956	26.2202	27.7387	28.681	25.5977	26.4215	27.2961	P36957;P36957-2,	P36957;P36957-2	Dihydrolipoyllysin	DLST
NaN	NaN	NaN	NaN	NaN	NaN	NaN	NaN	NaN	24.4724	23.7515	NaN	P11532;A0A087W	P11532;A0A087W	Dystrophin	DMD
21.4109	NaN	NaN	NaN	NaN	NaN	NaN	NaN	NaN	NaN	NaN	NaN	Q9NVR5;Q9NVR5-	Q9NVR5;Q9NVR5-	Protein kintoun	DNAAF2
25.5252	24.028	24.5837	25.3657	23.6332	24.5139	NaN	NaN	NaN	24.8261	25.1027	24.2888	Q86Y56;Q86Y56-2	Q86Y56;Q86Y56-2	Dynein assembly f	DNAAF5
NaN	NaN	NaN	NaN	NaN	NaN	NaN	NaN	NaN	NaN	NaN	25.7399	Q9P225;Q9P225-2	Q9P225;Q9P225-2	Dynein heavy cha	DNAH2
NaN	NaN	NaN	NaN	NaN	NaN	NaN	NaN	NaN	NaN	NaN	25.5179	Q96JB1;A0A075B6	Q96JB1;A0A075B6	Dynein heavy cha	DNAH8
29.3475	28.8848	28.8483	29.5416	28.6112	29.2058	29.9473	29.4815	29.3692	29.7521	29.2407	29.1162	P31689;P31689-2	P31689;P31689-2	DnaJ homolog sub	DNAJA1
29.0021	27.8633	27.9895	28.6759	27.6891	28.1611	29.2557	28.2962	28.2874	29.2984	28.8577	28.6092	O60884;A0A087W	O60884	DnaJ homolog sub	DNAJA2
25.4181	25.5842	24.9587	25.8835	26.2726	25.3547	23.3737	24.5231	25.2002	24.3685	25.4703	NaN	Q96EY1;Q96EY1-2	Q96EY1;Q96EY1-2	DnaJ homolog sub	DNAJA3
NaN	24.1871	24.6047	NaN	NaN	NaN	NaN	NaN	NaN	NaN	NaN	NaN	Q8WW22;Q8WW	Q8WW22;Q8WW	DnaJ homolog sub	DNAJA4
28.2828	28.3399	27.9713	27.6958	28.1241	28.058	28.2673	28.6552	28.6066	27.8137	28.2198	28.5475	P25685;P25685-2,	P25685;P25685-2,	DnaJ homolog sub	DNAJB1
26.9427	26.9523	26.3921	26.5428	26.2587	26.215	26.043	26.6645	25.7531	25.5017	25.1768	24.6502	Q9UBS4;H7C2Y5	Q9UBS4;H7C2Y5	DnaJ homolog sub	DNAJB11
21.918	NaN	NaN	NaN	NaN	NaN	NaN	NaN	NaN	NaN	NaN	NaN	Q9NXW2;V9GYN7	Q9NXW2;V9GYN7	DnaJ homolog sub	DNAJB12
22.827	NaN	NaN	NaN	NaN	NaN	NaN	NaN	NaN	NaN	NaN	NaN	P25686;C9JRD2;C	P25686;C9JRD2;C	DnaJ homolog sub	DNAJB2
24.2994	24.5851	24.1319	23.7524	25.1946	24.211	24.3316	24.4312	24.8031	24.2205	24.2723	25.2304	Q9UDY4;C9JUL4	Q9UDY4	DnaJ homolog sub	DNAJB4
23.8696	24.6374	24.187	24.4637	24.1295	24.2023	23.8763	24.2647	NaN	24.1133	23.8924	23.5516	O75190;O75190-3	O75190;O75190-3	DnaJ homolog sub	DNAJB6
22.8812	23.7593	23.2215	23.0478	23.8008	23.4247	NaN	23.9357	24.1865	22.8266	23.5141	23.7642	Q8IXB1;Q8IXB1-2;	Q8IXB1;Q8IXB1-2;	DnaJ homolog sub	DNAJC10
26.0535	25.5614	26.057	26.2896	25.8679	26.1842	25.5118	25.8227	25.9402	25.6904	25.7704	26.0557	O75165;H0Y8Q2;I	O75165	DnaJ homolog sub	DNAJC13
23.8066	NaN	NaN	24.4206	NaN	NaN	NaN	NaN	NaN	24.1754	NaN	NaN	Q99543;Q99543-2	Q99543;Q99543-2	DnaJ homolog sub	DNAJC2
25.7815	26.5869	25.9577	25.5831	26.1402	25.8192	26.5782	27.0476	26.738	25.9462	25.726	25.5931	Q13217;X6R9L0	Q13217;X6R9L0	DnaJ homolog sub	DNAJC3
25.8201	24.316	24.9727	25.722	23.6638	25.5107	24.8049	NaN	23.8817	25.8606	24.4447	NaN	Q9H3Z4;Q9H3Z4-1	Q9H3Z4;Q9H3Z4-1	DnaJ homolog sub	DNAJC5
27.0592	26.521	26.2555	26.7465	26.4262	26.1567	26.4414	26.3705	26.2131	26.9631	26.854	26.9303	Q99615;Q99615-2	Q99615;Q99615-2	DnaJ homolog sub	DNAJC7
23.9622	25.2745	24.8711	24.1096	25.3971	24.9841	24.3909	24.2673	24.4838	24.8237	25.6463	25.9465	O75937;S4R3J5	O75937	DnaJ homolog sub	DNAJC8
25.8227	25.8848	25.9574	25.6121	25.6714	25.9475	25.8095	25.4536	25.4643	26.4935	26.5356	26.3633	Q8WXX5	Q8WXX5	DnaJ homolog sub	DNAJC9
24.1951	24.5582	25.5406	24.4425	24.6009	25.598	24.3666	24.4087	NaN	24.4595	24.513	24.2449	P49184;A6QRJ0;C	P49184;A6QRJ0;C	Deoxyribonucleas	DNASE1L1
22.954	24.9751	23.8434	22.9532	24.2923	NaN	24.7934	24.4197	25.0259	23.2516	23.1398	NaN	O00115;K7ENE5;C	O00115;K7ENE5;C	Deoxyribonucleas	DNASE2
22.4696	22.4243	22.7151	22.1766	NaN	NaN	NaN	NaN	NaN	NaN	NaN	NaN	Q05193;A0A0D9S	Q05193;A0A0D9S	Dynamin-1	DNM1
27.6299	26.6867	26.8789	27.2584	27.14	26.7165	26.9815	26.6012	26.0854	27.3695	27.4594	25.6077	O00429-4;O0042S	O00429-4;O0042S	Dynamin-1-like pr	DNM1L
28.4109	28.2227	28.237	28.3846	28.1751	28.2826	28.1199	28.2865	27.992	28.5345	28.3378	28.2033	P50570-2	P50570-2	Dynamin-2	DNM2
NaN	NaN	NaN	20.8933	NaN	NaN	NaN	NaN	NaN	NaN	NaN	NaN	P50570-3;K7EMQ	P50570-3	Dynamin-2	DNM2
26.1942	25.0982	25.8816	26.3711	25.4725	25.8091	25.829	25.3961	25.9471	25.491	25.6094	25.6282	Q6XZF7;Q6XZF7-2	Q6XZF7;Q6XZF7-2	Dynamin-binding	DNMBP
26.8606	24.4352	25.7078	26.4298	24.4898	25.0763	25.6214	23.5401	21.6635	25.9924	25.7539	NaN	P26358;P26358-2,	P26358;P26358-2,	DNA (cytosine-5)-	DNMT1
24.2597	24.3651	24.4569	24.0955	24.9606	24.435	24.2811	24.4451	24.105	24.5051	24.5926	24.5333	Q9ULA0;E7EMB6;	Q9ULA0;E7EMB6;	Aspartyl aminope	DNPEP
24.5931	24.3027	24.6387	24.5523	24.6448	24.5052	25.0097	25.0311	NaN	25.1591	25.3169	25.2251	O43598;H0Y8X4;C	O43598;H0Y8X4;C	2-deoxynucleosid	DNPH1
24.4596	23.6383	23.6764	24.7009	23.8036	24.2697	NaN	NaN	NaN	23.4561	24.4244	NaN	Q9H147;H7C1M5;	Q9H147;H7C1M5;	Deoxynucleotidylt	DNTTIP1
23.0767	NaN	22.5201	23.7155	22.5386	NaN	NaN	23.5561	NaN	NaN	NaN	NaN	Q5QJE6;J3KP30;E	Q5QJE6;J3KP30	Deoxynucleotidylt	DNTTIP2
26.1665	26.1579	26.0321	25.9375	25.9961	26.0087	25.6723	NaN	27.42	26.5823	25.9231	24.5341	Q14185;A0A096LI	Q14185;A0A096LI	Dedicator of cytol	DOCK1
25.7452	25.7668	26.5292	25.7951	26.1453	26.699	27.0628	26.597	26.1324	28.2506	27.6506	27.4055	Q96BY6;Q96BY6-3	Q96BY6;Q96BY6-3	Dedicator of cytol	DOCK10
25.452	23.9706	24.5949	25.0416	24.663	24.7383	24.4435	25.227	24.7844	24.9357	24.4633	24.2129	Q8N1I0;H0Y599;C	Q8N1I0;H0Y599;C	Dedicator of cytol	DOCK4
25.6786	24.8731	25.4581	25.4232	24.8095	25.3201	24.8726	24.8344	25.0689	25.4001	24.7234	24.8189	Q9H7D0;H0Y7N4;	Q9H7D0;H0Y7N4	Dedicator of cytol	DOCK5
28.1083	27.5422	28.0635	28.2168	28.0072	28.2606	27.5977	27.9478	27.9086	27.942	28.0708	28.1176	Q96N67;Q96N67-	Q96N67;Q96N67-	Dedicator of cytol	DOCK7
NaN	NaN	22.1085	NaN	NaN	NaN	NaN	NaN	NaN	NaN	NaN	NaN	Q9BZ29-5;A0A0A(	Q9BZ29-5;A0A0A(	Dedicator of cytol	DOCK9
28.4264	28.1015	28.4878	28.1305	27.8608	28.2926	28.1825	27.479	27.8459	28.399	28.0618	27.8445	Q9BZ29-4;Q9BZ2S	Q9BZ29-4;Q9BZ2S	Dedicator of cytol	DOCK9
23.9107	24.0058	23.7967	24.0711	23.3914	23.2118	NaN	NaN	NaN	23.1484	23.4535	NaN	Q9BU89;K7ERU8;I	Q9BU89;K7ERU8;I	Deoxyhypusine hy	DOHH
NaN	NaN	NaN	21.659	NaN	NaN	NaN	NaN	NaN	NaN	NaN	NaN	Q99704;Q99704-2	Q99704;Q99704-2	Docking protein 1	DOK1
NaN	NaN	23.026	23.6147	NaN	NaN	NaN	NaN	NaN	NaN	NaN	NaN	Q9H3H5;Q9H3H5-	Q9H3H5;Q9H3H5-	UDP-N-acetylgluc	DPAGT1
23.1489	23.8205	23.2394	23.0747	22.6753	23.3996	23.2605	23.3069	23.7684	23.5559	23.6695	23.4819	Q9H2P9;A0A087V	Q9H2P9;A0A087V	Diphthine synthas	DPH5
24.4241	24.0605	24.2747	24.8746	24.2237	24.6464	24.5612	23.7191	NaN	24.4182	24.2859	NaN	O60762;Q5QPK2;I	O60762;Q5QPK2;I	Dolichol-phosphat	DPM1
NaN	NaN	NaN	NaN	NaN	NaN	NaN	NaN	NaN	NaN	NaN	22.237	Q9P2X0;Q9P2X0-2	Q9P2X0;Q9P2X0-2	Dolichol-phosphat	DPM3
27.3871	28.4513	27.9608	27.2145	28.1862	27.9872	28.3962	28.2353	28.4497	28.0626	28.017	28.6939	Q9NY33;G3V1D3;	Q9NY33;G3V1D3;	Dipeptidyl peptida	DPP3
27.1647	26.8204	26.8621	26.3849	26.4004	26.5115	26.9246	26.8764	26.0836	26.3865	26.373	26.1792	Q86TI2;Q86TI2-2;	Q86TI2;Q86TI2-2;	Dipeptidyl peptida	DPP9
25.6301	26.7218	25.9002	25.735	26.6882	26.0333	25.7383	NaN	NaN	NaN	26.3117	NaN	Q9C005	Q9C005	Protein dpy-30 ho	DPY30
23.9168	23.9466	NaN	NaN	23.8378	NaN	NaN	NaN	NaN	NaN	NaN	NaN	Q12882	Q12882	Dihydropyrimidine	DPYD
29.6088	29.9018	29.7028	28.9271	29.5236	29.5675	29.308	30.0811	29.2798	29.4213	29.3672	29.4333	Q16555;Q16555-2	Q16555;Q16555-2	Dihydropyrimidina	DPYSL2
23.52	24.0654	23.9684	23.7694	24.3859	24.0331	23.8755	24.3024	24.4429	24.0309	24.6253	25.1437	Q01658	Q01658	Protein Dr1	DR1
22.6619	22.5263	23.5412	23.6625	22.483	NaN	22.6868	NaN	NaN	23.9546	23.3062	24.3972	Q14919;Q14919-2	Q14919;Q14919-2	Dr1-associated co	DRAP1
26.5416	25.4978	25.6724	26.2173	25.7058	25.4941	25.8527	25.8436	25.5831	26.1462	26.0828	25.7044	Q9Y295;H0YI06;F	Q9Y295	Developmentally-	DRG1
26.559	25.6435	26.259	26.426	25.8797	25.8583	25.3234	25.1422	25.3255	26.8078	26.7313	25.8431	P55039;A8MZF9;J	P55039;A8MZF9;J	Developmentally-	DRG2
NaN	NaN	NaN	NaN	NaN	NaN	23.5708	NaN	NaN	NaN	NaN	NaN	Q08554;Q08554-2	Q08554;Q08554-2	Desmocollin-1	DSC1

NaN	NaN	NaN	22.601	NaN	NaN	24.2482	24.007	23.929	NaN	NaN	NaN	Q02413;Q02413-2	Q02413	Desmoglein-1	DSG1
27.6155	28.1367	28.341	27.6348	27.9171	28.5215	28.3165	28.2275	28.7222	28.8417	28.179	28.4572	Q14126;J3KSI6	Q14126	Desmoglein-2	DSG2
NaN	NaN	NaN	21.6351	NaN	NaN	NaN	NaN	NaN	NaN	NaN	NaN	Q9H410;Q5JW54;	Q9H410;Q5JW54;	Kinetochore-asso	DSN1
26.2065	25.7666	25.5626	26.6399	26.0476	25.2429	27.7763	24.8889	25.9971	22.8012	25.2288	NaN	P15924;P15924-3	P15924;P15924-3	Desmoplakin	DSP
27.242	26.5769	27.0338	26.915	26.7965	26.9092	26.8994	27.0466	24.5408	27.404	26.8171	26.5415	E9PEB9;F6QMI7;C	E9PEB9;F6QMI7;C	Dystonin	DST
NaN	NaN	NaN	NaN	NaN	NaN	NaN	NaN	24.5119	NaN	NaN	NaN	Q03001-13;A0A0J	Q03001-13	Dystonin	DST
NaN	NaN	NaN	NaN	NaN	NaN	NaN	NaN	NaN	NaN	NaN	24.198	Q03001-3;Q6P0N	Q03001-3;Q6P0N	Dystonin	DST
26.8392	27.4215	26.9936	26.5605	26.9059	27.0168	26.3165	27.0023	27.64	27.6917	27.919	28.2974	P60981;P60981-2	P60981;P60981-2	Destrin	DSTN
25.5734	26.2231	25.737	25.7132	26.1281	25.607	25.9892	NaN	25.9834	26.0601	26.0995	NaN	Q8TEA8;A0A087W	Q8TEA8;A0A087W	D-tyrosyl-tRNA(Ty	DTD1
23.4403	23.3539	22.9145	22.8347	23.3644	23.2026	NaN	NaN	NaN	23.9858	24.1826	NaN	Q9NZJ0;F5GZ90;C	Q9NZJ0;F5GZ90	Denticleless prote	DTL
22.4815	NaN	23.8499	23.1201	23.0246	23.679	23.0485	NaN	NaN	24.309	23.4486	23.5982	Q86UW9;E7ET89;	Q86UW9;E7ET89;	Probable E3 ubiqu	DTX2
NaN	NaN	NaN	19.7265	NaN	NaN	NaN	NaN	NaN	NaN	NaN	NaN	Q8TDB6	Q8TDB6	E3 ubiquitin-prote	DTX3L
28.3969	27.7582	27.764	27.9742	27.8272	27.8693	27.5968	27.8473	27.4331	27.9749	28.0884	27.5906	P23919;P23919-2	P23919;P23919-2	Thymidylate kinas	DTYMK
22.9915	21.8379	21.8397	22.1064	NaN	NaN	NaN	NaN	NaN	22.319	22.7557	NaN	Q9NX74;E7EUN9;	Q9NX74;E7EUN9;	tRNA-dihydrourid	DUS2
23.9943	22.974	22.9914	24.2415	23.4458	23.1272	NaN	NaN	NaN	23.1298	24.0964	NaN	Q96G46;Q96G46-	Q96G46;Q96G46-	tRNA-dihydrourid	DUS3L
22.4329	NaN	NaN	21.3438	NaN	NaN	NaN	NaN	NaN	NaN	NaN	NaN	P28562	P28562	Dual specificity pr	DUSP1
21.4757	NaN	NaN	21.183	NaN	NaN	NaN	NaN	NaN	NaN	NaN	NaN	O75319;H7C0E6;C	O75319;H7C0E6;C	RNA/RNP comple	DUSP11
23.4564	23.4294	22.9842	22.971	23.1698	22.6949	23.042	23.5787	NaN	23.2045	23.6173	23.5824	Q9UNI6;V9GYV5;	Q9UNI6	Dual specificity pr	DUSP12
NaN	NaN	NaN	NaN	NaN	NaN	NaN	NaN	NaN	22.4656	23.3564	NaN	Q9BVJ7	Q9BVJ7	Dual specificity pr	DUSP23
22.9326	23.4704	23.5791	23.4777	23.5571	23.4543	NaN	NaN	NaN	23.3878	NaN	23.887	P51452;K7ES89;P	P51452;K7ES89;P	Dual specificity pr	DUSP3
NaN	NaN	NaN	NaN	NaN	NaN	NaN	NaN	NaN	NaN	NaN	22.296	Q13115	Q13115	Dual specificity pr	DUSP4
26.4969	28.1099	27.2803	26.2695	27.7022	27.086	27.6044	28.4808	28.058	27.6173	28.155	28.7175	P33316;P33316-2	P33316;P33316-2	Deoxyuridine 5-tri	DUT
24.5406	24.2657	24.6406	24.2299	24.2975	24.2142	23.9981	NaN	NaN	24.2081	NaN	23.6454	O14641;I3L2N2;I3	O14641;I3L2N2;I3	Segment polarity	DVL2
33.0406	32.0403	32.5715	33.0396	32.3462	32.6701	32.6361	32.8802	32.6844	33.0673	32.8188	32.7007	Q14204;H0YJ21	Q14204	Cytoplasmic dyne	DYNC1H1
26.9851	27.2194	26.806	26.5049	27.3318	26.8727	27.3371	27.9629	27.8198	26.6385	27.1418	27.5483	Q13409-3;Q1340	Q13409-3;Q1340	Cytoplasmic dyne	DYNC1I2
28.074	28.1923	27.9242	28.0451	28.2229	27.9788	28.2243	29.0151	28.1342	28.6716	28.6928	28.3999	Q9Y6G9;E9PHI6;C	Q9Y6G9;E9PHI6	Cytoplasmic dyne	DYNC1LI1
26.1327	26.4462	26.3232	25.9773	26.2945	26.1153	26.1878	27.0967	26.3813	26.025	26.229	26.3434	O43237;O43237-2	O43237;O43237-2	Cytoplasmic dyne	DYNC1LI2
27.2117	24.6585	24.7208	26.4091	24.4516	24.2359	26.4811	NaN	NaN	26.0362	25.1647	23.2297	P63167;Q96FJ2;F	P63167;Q96FJ2;F	Dynein light chain	DYNLL1;DYNLL2
27.4504	27.556	26.9602	26.4544	27.0331	26.9273	27.9267	27.2705	27.1329	26.7087	27.2732	27.0625	Q9NP97;B1AKR6;	Q9NP97;B1AKR6;	Dynein light chain	DYNLRB1;DYNLRB
25.1746	NaN	NaN	24.0914	NaN	NaN	23.4779	NaN	NaN	NaN	NaN	NaN	P63172;Q5VTU3	P63172;Q5VTU3	Dynein light chain	DYNLT1
31.682	31.3367	31.9373	32.1013	31.6268	32.1548	29.7782	28.9125	29.0111	30.4589	29.5246	28.9816	O75923-8;O7592	O75923-8;O7592	Dysferlin	DYSF
NaN	NaN	NaN	NaN	NaN	NaN	NaN	26.0231	NaN	NaN	NaN	NaN	A0AVK6	A0AVK6	Transcription fact	E2F8
23.1582	NaN	22.5707	23.2881	22.632	NaN	NaN	NaN	NaN	22.6305	22.9428	NaN	Q5JPH6;H3BTB7;C	Q5JPH6;H3BTB7;C	Probable glutama	EARS2
NaN	NaN	NaN	NaN	NaN	NaN	25.9937	NaN	NaN	NaN	NaN	NaN	Q9HAK2;B7Z934	Q9HAK2;B7Z934	Transcription fact	EBF2
25.5584	24.8458	24.3508	25.3107	24.8464	24.5539	NaN	NaN	NaN	23.6601	25.2433	24.5085	Q99848;H7C2Q8	Q99848;H7C2Q8	Probable rRNA-pr	EBNA1BP2
NaN	NaN	NaN	25.6354	NaN	NaN	NaN	NaN	NaN	25.8808	NaN	NaN	Q15125;C9J719;C	Q15125;C9J719;C	3-beta-hydroxyste	EBP
23.9729	23.1639	23.3457	24.0429	23.0376	24.5296	23.6319	NaN	NaN	24.1094	24.6411	NaN	O95905;O95905-3	O95905;O95905-3	Protein SGT1	ECD
27.9252	28.396	29.0619	28.3461	28.5639	29.2666	27.8934	27.5381	28.1246	28.6971	28.1272	28.0617	P42892;P42892-3	P42892;P42892-3	Endothelin-conve	ECE1
26.4278	27.7802	27.0671	26.674	27.9632	27.0765	25.3997	27.4472	28.2827	25.4208	26.5802	27.2006	Q13011;M0R248;	Q13011	Delta(3,5)-Delta(2	ECH1
23.9622	24.8704	24.1204	23.5982	24.3021	23.9322	24.4499	NaN	NaN	23.9629	23.9603	NaN	Q9NTX5;Q9NTX5-	Q9NTX5;Q9NTX5-	Ethylmalonyl-CoA	ECHDC1
25.7489	27.3364	26.2913	25.7119	27.0829	26.3776	25.8476	26.865	27.4579	25.5808	26.3232	26.483	P30084	P30084	Enoyl-CoA hydrat	ECHS1
24.757	26.6931	24.8118	24.5776	26.4796	24.9676	24.1205	NaN	26.3098	24.1815	25.4164	25.2996	P42126;P42126-2	P42126;P42126-2	Enoyl-CoA delta is	ECI1;DCI
26.4605	26.7415	26.385	26.6232	26.8181	26.2525	25.1994	25.1046	26.3089	25.3425	25.1841	25.229	O75521;A0A0C4D	O75521;A0A0C4D	Enoyl-CoA delta is	ECI2
23.6669	25.4586	23.4658	23.486	24.852	23.4135	27.8859	27.1588	24.7872	24.251	25.3637	23.3134	Q16610;Q16610-4	Q16610;Q16610-4	Extracellular matr	ECM1
27.8473	26.6327	27.4347	27.8864	27.2874	27.2454	26.6527	25.3725	26.0593	27.3253	27.2634	27.0141	Q5VYK3;J3KN16;R	Q5VYK3;J3KN16	Proteasome-asso	ECM29;KIAA0368
25.1232	24.159	25.6195	26.0874	24.8087	25.4253	23.6588	NaN	23.4122	25.7899	25.1614	25.5503	Q9H8V3;Q9H8V3-	Q9H8V3;Q9H8V3-	Protein ECT2	ECT2
25.6539	25.2251	24.8738	25.2815	24.7304	24.8013	24.245	NaN	24.3945	25.1247	25.1567	NaN	Q96F86;H3BPW9;	Q96F86	Enhancer of mRN	EDC3
27.2064	26.7488	26.3085	27.025	26.6235	26.2821	25.9665	26.348	25.5064	26.6771	26.6269	26.0828	Q6P2E9;Q6P2E9-2	Q6P2E9;Q6P2E9-2	Enhancer of mRN	EDC4
25.7445	25.9401	25.618	24.9643	25.342	25.4807	26.0444	25.8943	25.754	26.1923	25.9456	26.0607	O60869;O60869-2	O60869;O60869-2	Endothelial differe	EDF1
32.6773	33.5566	34.2408	32.4365	33.1191	34.2891	36.1543	35.3465	35.0525	34.4342	33.4721	33.2898	O43854;O43854-2	O43854;O43854-2	EGF-like repeat ar	EDIL3
28.8764	28.7974	28.165	28.3163	28.1951	27.9707	28.0464	28.502	27.8468	27.7776	27.6308	27.3628	Q15075	Q15075	Early endosome a	EEA1
24.0979	22.7555	23.0682	24.029	22.8925	23.0319	NaN	NaN	NaN	23.0446	23.5254	NaN	O75530;O75530-2	O75530;O75530-2	Polycomb protein	EED
35.2054	34.7137	34.7027	34.9326	34.6705	34.7228	34.7629	34.3554	34.1278	34.968	34.7081	34.3619	P68104;Q5VTE0;A	P68104;Q5VTE0;A	Elongation factor	EEF1A1;EEF1A1P5
24.4234	24.8567	25.059	25.1345	25.1572	25.2835	26.5354	25.8068	25.6616	26.747	26.4061	25.891	Q05639	Q05639	Elongation factor	EEF1A2
28.8741	28.761	28.7165	28.6989	28.6791	28.5101	29.4366	30.4072	29.633	29.2691	29.7607	30.0561	P24534;C9JZW3;F	P24534	Elongation factor	EEF1B2
31.0041	31.2638	31.3378	30.8744	31.3106	31.0533	31.3125	32.0952	30.9797	30.9619	31.4101	31.4675	P29692;E9PK01;P	P29692;E9PK01;P	Elongation factor	EEF1D
NaN	26.573	27.105	NaN	26.9086	26.7354	26.7403	NaN	NaN	27.0689	27.3447	NaN	P29692-3;E9PL71;	P29692-3;E9PL71;	Elongation factor	EEF1D
28.1411	27.3822	27.5746	28.0949	27.502	27.5516	27.0023	27.4213	26.7822	27.7187	27.2826	27.0456	O43324;D6RBD7;	O43324;D6RBD7;	Eukaryotic transla	EEF1E1;EEF1E1-BL
31.2055	30.9725	30.8154	31.1041	31.2084	30.7847	31.0883	31.3306	30.803	31.1736	31.6394	31.1664	P26641;P26641-2	P26641;P26641-2	Elongation factor	EEF1G
32.9033	32.1128	32.2597	32.541	32.3099	32.2728	32.3734	32.2982	32.3247	32.5828	32.5794	32.4268	P13639	P13639	Elongation factor	EEF2

NaN	NaN	21.348	21.809	NaN	NaN	NaN	NaN	NaN	NaN	NaN	NaN	O00418;H3BRH4	O00418;H3BRH4	Eukaryotic elonga	EEF2K
24.2782	NaN	24.1181	24.5634	24.0415	24.1083	NaN	NaN	NaN	24.0266	24.033	NaN	P57772;P57772-2	P57772;P57772-2	Selenocysteine-sp	EEFSEC
25.9012	26.9436	NaN	NaN	26.6828	25.7947	25.6612	NaN	NaN	NaN	NaN	NaN	A4FU69;A4FU69-5	A4FU69;A4FU69-5	EF-hand calcium-b	EFCAB5
25.7133	26.1293	26.2527	25.5345	26.4309	26.4605	25.799	26.1765	25.9518	25.7716	25.8598	26.6296	Q96C19;H0Y4Y4	Q96C19	EF-hand domain-c	EFHD2
22.4176	NaN	23.3226	NaN	NaN	NaN	NaN	NaN	NaN	23.3983	NaN	NaN	P98172	P98172	Ephrin-B1	EFNB1
NaN	NaN	NaN	NaN	NaN	NaN	NaN	NaN	NaN	20.7921	NaN	NaN	P52799	P52799	Ephrin-B2	EFNB2
26.2497	25.8091	26.8689	26.4668	26.4216	26.8887	26.0567	25.2307	25.3265	27.4578	26.3999	25.2402	Q14156;Q14156-2	Q14156;Q14156-2	Protein EFR3 hom	EFR3A
26.7351	26.0715	26.2088	26.6108	26.4371	26.2557	26.3458	26.3596	26.627	26.5247	26.7708	26.6919	Q7Z2Z2;Q7Z2Z2-2	Q7Z2Z2;Q7Z2Z2-2	Elongation factor	EFTUD1
28.881	27.7234	28.3118	28.8989	28.2208	28.4174	27.1881	27.6274	27.2405	28.4535	28.6511	28.2693	Q15029;Q15029-2	Q15029;Q15029-2	116 kDa U5 small	EFTUD2
NaN	NaN	NaN	NaN	NaN	NaN	25.559	NaN	NaN	NaN	NaN	NaN	Q9UHF1;R4GMT3	Q9UHF1	Epidermal growth	EGFL7
30.2023	29.9266	30.6313	30.3384	29.77	30.8666	30.4087	29.6272	30.0966	31.0034	30.4081	30.3803	P00533;E9PFD7;Q	P00533;E9PFD7;Q	Epidermal growth	EGFR
23.8916	23.7617	23.4275	23.6764	23.666	NaN	NaN	NaN	NaN	23.3945	23.619	23.5201	Q9GZT9;Q9GZT9-3	Q9GZT9;Q9GZT9-3	Egl nine homolog	EGLN1
24.3398	24.0696	24.6639	23.8079	NaN	NaN	NaN	NaN	NaN	23.7728	NaN	23.6568	Q8NDI1;Q8NDI1-3	Q8NDI1;Q8NDI1-3	EH domain-bindin	EHBP1
25.255	26.1483	26.1754	24.8455	26.0588	26.2055	26.1526	26.1682	26.2615	25.9529	26.0446	26.4218	Q8N3D4;E9PIH6;A	Q8N3D4;E9PIH6	EH domain-bindin	EHBP1L1
30.6398	31.1568	31.8254	30.5721	30.9673	31.4435	31.2919	30.3532	30.7888	31.6073	31.3575	30.8903	Q9H4M9;A0A024	Q9H4M9;A0A024	EH domain-contai	EHD1
26.767	26.607	27.2794	26.956	26.4023	27.1701	27.7622	27.0019	27.6034	27.7882	27.5153	27.4933	Q9NZN4;Q9NZN4	Q9NZN4;Q9NZN4	EH domain-contai	EHD2
NaN	NaN	20.2514	NaN	NaN	NaN	NaN	NaN	NaN	NaN	NaN	NaN	Q9NZN3;A0A0C4D	Q9NZN3;A0A0C4D	EH domain-contai	EHD3
30.248	30.2534	31.133	30.0583	30.1071	30.7839	30.0129	29.2172	29.1495	30.3898	30.4461	29.7109	Q9H223;A0A087V	Q9H223;A0A087V	EH domain-contai	EHD4
26.4257	27.4348	26.9242	26.0685	26.8606	26.4335	26.5264	NaN	26.5222	26.4041	26.9442	27.0329	P41567;K7EM18;(	P41567;K7EM18;(	Eukaryotic transla	EIF1;EIF1B
NaN	NaN	NaN	NaN	NaN	NaN	23.2864	NaN	NaN	NaN	NaN	NaN	Q8N9N8;E9PQD0;	Q8N9N8;E9PQD0;	Probable RNA-bin	EIF1AD
27.3489	26.1673	26.4152	27.0027	25.6072	26.1584	26.9505	26.4284	NaN	26.6176	26.3507	25.9479	P47813;O14602;A	P47813;O14602;A	Eukaryotic transla	EIF1AX;EIF1AY
27.5177	27.4128	27.1416	27.0928	27.3486	27.0792	26.7412	27.1746	27.2448	26.8394	27.1656	27.5116	Q9BY44;Q9BY44-3	Q9BY44;Q9BY44-3	Eukaryotic transla	EIF2A
27.5921	27.3091	27.0987	27.5561	27.3726	27.2351	26.5677	27.2468	27.0105	26.4597	27.208	27.3939	P19525;P19525-2	P19525;P19525-2	Interferon-induce	EIF2AK2
24.5378	24.0768	24.1116	24.0413	23.9682	NaN	NaN	NaN	NaN	NaN	NaN	NaN	Q9P2K8;Q9P2K8-2	Q9P2K8;Q9P2K8-2	Eukaryotic transla	EIF2AK4
26.5065	25.9363	25.9295	26.4222	25.8912	25.6598	25.3587	24.5909	25.1436	25.6051	25.7134	25.8519	Q14232;F5H0D0;(	Q14232;F5H0D0;(	Translation initiat	EIF2B1
28.1399	27.6538	27.7276	28.0056	28.027	27.4797	27.2358	27.2291	26.0878	27.572	27.5098	27.2683	P49770;G3V5E5;H	P49770;G3V5E5;H	Translation initiat	EIF2B2
26.991	25.9595	26.2649	26.9683	26.4449	26.1885	25.5345	25.4077	24.8956	25.7544	25.9245	26.0032	Q9NR50;Q9NR50-	Q9NR50;Q9NR50-	Translation initiat	EIF2B3
27.5107	26.2706	26.855	27.4563	26.8022	27.0359	25.5361	25.4483	26.0092	26.8415	26.8448	25.0619	Q9UI10;Q9UI10-2	Q9UI10;Q9UI10-2	Translation initiat	EIF2B4
26.5571	25.0943	25.8444	26.5881	25.3853	26.0086	24.8781	24.9081	24.6039	25.5452	25.4802	NaN	Q13144;E9PC74;C	Q13144;E9PC74	Translation initiat	EIF2B5
23.6989	22.9705	23.1168	23.9	NaN	23.1416	NaN	NaN	NaN	23.214	23.6155	NaN	P41214;P41214-2	P41214;P41214-2	Eukaryotic transla	EIF2D
30.0392	29.4537	29.4605	29.8387	29.4568	29.4399	29.4883	29.8545	29.2622	29.4599	29.706	29.526	P05198;G3V4T5;H	P05198;G3V4T5;H	Eukaryotic transla	EIF2S1
29.093	28.3433	28.6814	28.8813	28.668	28.645	28.3618	28.6635	28.4827	28.7756	29.0867	28.9893	P20042	P20042	Eukaryotic transla	EIF2S2
29.5428	29.6589	29.5125	29.5367	29.9995	29.6752	29.4634	29.9943	29.6284	29.6827	30.1045	29.772	P41091;Q2VIR3;F	P41091;Q2VIR3;F	Eukaryotic transla	EIF2S3;EIF2S3L
31.1981	30.7209	31.0677	31.1217	31.1815	30.9841	31.0677	31.5757	31.0735	31.2355	31.4764	31.1002	Q14152;Q14152-2	Q14152;Q14152-2	Eukaryotic transla	EIF3A
29.5018	28.8855	29.6801	29.5268	29.1684	29.6028	29.4745	30.1466	29.303	29.9503	30.0899	29.5296	P55884;P55884-2	P55884;P55884-2	Eukaryotic transla	EIF3B
29.7143	29.0857	29.5164	29.4575	29.4321	29.4835	29.7633	30.1044	29.6302	29.6671	29.7725	29.8362	Q99613;Q99613-2	Q99613;Q99613-2	Eukaryotic transla	EIF3C;EIF3CL
28.9346	28.5292	28.8091	28.8557	28.7443	28.863	28.7421	29.0929	29.0786	28.1688	28.6881	29.0699	O15371;O15371-2	O15371;O15371-2	Eukaryotic transla	EIF3D
29.4289	28.942	29.254	29.5905	29.2391	29.1727	29.1123	29.6322	29.3352	29.6326	29.5049	29.2281	P60228;E5RGA2;H	P60228;E5RGA2	Eukaryotic transla	EIF3E
29.5046	29.2231	29.1944	29.3561	29.7075	29.3935	29.0887	29.5404	29.1409	29.4162	29.3206	29.1262	O00303;H0YDT6;A	O00303	Eukaryotic transla	EIF3F
26.129	26.2985	26.0535	25.7773	25.8386	25.8635	26.2308	26.8769	26.3056	25.6048	26.145	26.5678	O75821;K7ER90;K	O75821;K7ER90;K	Eukaryotic transla	EIF3G
28.0733	28.2132	27.9548	28.0356	28.2983	27.9421	28.3034	28.8968	28.2027	28.1246	28.2225	27.9481	O15372;B3KS98;A	O15372;B3KS98;A	Eukaryotic transla	EIF3H
29.3815	29.713	29.4032	29.1642	29.7947	29.45	29.49	30.8434	29.7057	29.9833	30.6384	29.9813	Q13347;Q5TFK1	Q13347	Eukaryotic transla	EIF3I
24.8808	24.826	24.639	24.3312	24.7168	24.4929	25.8178	25.9703	25.8802	24.8032	25.2302	25.8525	O75822;O75822-3	O75822;O75822-3	Eukaryotic transla	EIF3J
26.5732	26.6535	26.4075	26.4058	26.9385	26.0923	26.4142	27.0683	26.5134	26.3721	27.1727	26.9621	Q9UBQ5;K7ES31;I	Q9UBQ5;K7ES31;I	Eukaryotic transla	EIF3K
29.3249	28.6612	29.0227	29.1624	28.8188	28.9952	29.1253	29.5024	29.1869	29.5346	29.5279	29.176	Q9Y262;B0QY89;(	Q9Y262;B0QY89;(	Eukaryotic transla	EIF3L
27.7582	26.9107	27.2252	27.8775	26.8307	26.8675	27.6259	26.3362	26.2577	27.3324	26.9897	26.1801	Q7L2H7;Q7L2H7-2	Q7L2H7;Q7L2H7-2	Eukaryotic transla	EIF3M
32.9756	32.4808	32.4101	32.9905	32.423	32.5649	32.1416	31.9312	31.8734	32.2929	32.1458	32.0648	P60842;J3KT12;P6	P60842;J3KT12;P6	Eukaryotic initiati	EIF4A1
26.274	26.3858	25.9152	26.1483	26.379	25.908	26.3953	25.471	25.6667	26.1806	26.0677	25.6673	Q14240;Q14240-2	Q14240;Q14240-2	Eukaryotic initiati	EIF4A2
31.1479	30.1847	30.0276	31.0176	30.1237	30.3149	28.9672	29.1036	28.9387	30.5119	30.7338	29.9103	P38919;I3L3H2;E9	P38919	Eukaryotic initiati	EIF4A3
28.3088	28.2976	28.3223	28.0642	28.4384	28.2207	28.2611	27.7212	26.852	28.7211	28.4586	28.1547	P23588;E7EX17;P	P23588;E7EX17;P	Eukaryotic transla	EIF4B
26.5948	25.7702	25.6818	25.8124	25.9102	25.9038	25.4454	25.8343	26.6655	26.4478	26.9403	NaN	P06730;P06730-2	P06730;P06730-2	Eukaryotic transla	EIF4E
24.5922	24.6356	24.2147	24.0327	24.2087	24.3176	23.8681	NaN	NaN	24.0509	24.3208	NaN	O60573;C9JEL3;B	O60573;C9JEL3;B	Eukaryotic transla	EIF4E2
24.9865	25.2568	24.2034	23.2155	24.9202	23.2331	NaN	NaN	23.3288	23.1644	22.8834	23.9098	Q13541	Q13541	Eukaryotic transla	EIF4EBP1
NaN	NaN	NaN	20.1337	NaN	NaN	NaN	NaN	NaN	NaN	NaN	NaN	Q13542	Q13542	Eukaryotic transla	EIF4EBP2
NaN	NaN	NaN	24.3677	NaN	NaN	NaN	NaN	NaN	NaN	NaN	NaN	Q04637-8;E7EX73	Q04637-8;E7EX73	Eukaryotic transla	EIF4G1
29.736	29.0625	29.1752	29.4189	28.9791	29.2369	28.4596	29.288	28.6022	28.503	28.7482	28.9514	Q04637;Q04637-3	Q04637;Q04637-3	Eukaryotic transla	EIF4G1
28.807	27.727	28.2586	28.8423	28.0597	28.4141	27.1519	27.2619	26.9697	27.2223	27.4399	27.4021	P78344;D3DQV9;I	P78344;D3DQV9;I	Eukaryotic transla	EIF4G2
27.3532	27.7427	27.6836	27.0424	27.378	27.6647	28.0252	28.79	26.6883	27.8594	27.9328	27.885	Q15056;Q15056-2	Q15056;Q15056-2	Eukaryotic transla	EIF4H
27.3781	26.5645	27.1126	27.2123	26.573	26.8856	27.0169	27.4364	26.1501	27.1085	26.9141	26.8083	P55010;H0YLZ1;H	P55010	Eukaryotic transla	EIF5
30.28	30.4277	30.0575	30.2005	30.2341	30.1102	30.6386	30.0544	29.8681	30.1496	30.2091	29.9094	P63241;I3L397;I3	P63241;I3L397;I3	Eukaryotic transla	EIF5A;EIF5A2;EIF5

27.2887	26.9484	27.0333	27.1644	27.1261	26.9256	27.5378	27.8126	27.6861	27.3603	27.4416	27.8028	O60841;A0A087W	O60841;A0A087W	Eukaryotic transla	EIF5B
28.276	28.2017	27.903	28.0036	27.8805	27.8395	27.198	26.4359	27.0429	28.1075	28.2189	27.6007	P56537;P56537-2	P56537;P56537-2	Eukaryotic transla	EIF6
25.139	23.6893	24.4992	25.1039	24.4395	24.3847	22.9302	22.7575	22.9528	26.6653	26.6294	23.8605	Q9BQ52;G5E9D5;	Q9BQ52;G5E9D5;	Zinc phosphodiest	ELAC2
27.6808	26.8884	26.4774	27.4377	26.9901	26.7344	25.9302	25.938	23.5425	26.0426	26.2364	26.0634	Q15717;Q15717-2	Q15717;Q15717-2	ELAV-like protein	ELAVL1
21.5849	NaN	NaN	22.0614	NaN	21.4831	NaN	NaN	NaN	NaN	21.8398	NaN	P55199;U3KQ90	P55199;U3KQ90	RNA polymerase I	ELL
26.9587	26.6859	27.0378	26.8324	26.8783	27.019	27.016	26.8301	26.6154	26.948	26.8788	26.7609	Q96JJ3;Q5JVZ5;Q	Q96JJ3;Q5JVZ5;Q	Engulfment and c	ELMO2
22.8837	NaN	NaN	22.6464	NaN	23.1434	NaN	NaN	NaN	22.7396	NaN	NaN	Q8IZ81;D6RBS5;D	Q8IZ81;D6RBS5;D	ELMO domain-cor	ELMOD2
24.6972	NaN	24.6997	25.0777	23.8329	NaN	24.9789	24.878	24.9943	NaN	24.6316	NaN	Q9BW60;Q9BW6(	Q9BW60;Q9BW6(	Elongation of very	ELOVL1
NaN	22.8854	23.5316	22.991	24.5314	24.0435	NaN	25.3907	26.1062	22.8431	23.926	25.2952	Q9NYP7;A0A0A0N	Q9NYP7;A0A0A0N	Elongation of very	ELOVL5
23.6747	NaN	23.6403	23.903	23.8528	NaN	NaN	NaN	NaN	24.3902	24.3893	NaN	Q6IA86;Q6IA86-5	Q6IA86;Q6IA86-5	Elongator comple	ELP2
25.0272	23.6811	24.4607	25.1139	24.2837	24.5167	23.9366	NaN	NaN	24.3448	24.5617	23.3352	Q9H9T3;Q9H9T3-	Q9H9T3;Q9H9T3-	Elongator comple	ELP3
NaN	NaN	NaN	23.1276	NaN	NaN	NaN	NaN	NaN	NaN	NaN	NaN	Q96EB1;G5E9D4;(	Q96EB1;G5E9D4;(	Elongator comple	ELP4
NaN	NaN	NaN	21.2146	NaN	NaN	NaN	NaN	NaN	NaN	NaN	NaN	Q8TE02;Q8TE02-4	Q8TE02;Q8TE02-4	Elongator comple	ELP5
24.6824	NaN	23.8771	24.3174	23.0097	23.758	23.2238	NaN	NaN	23.3892	23.1724	NaN	Q0PNE2;C9IYN7;C	Q0PNE2;C9IYN7;C	Elongator comple	ELP6
23.744	NaN	23.7547	23.8581	NaN	NaN	NaN	NaN	NaN	NaN	NaN	NaN	Q6PCB8;Q6PCB8-	Q6PCB8;Q6PCB8-	Embigin	EMB
25.0207	24.9921	24.7199	24.8689	24.618	24.6531	25.1964	25.2869	25.9295	24.7515	24.5018	25.2084	Q8N766;Q8N766-	Q8N766;Q8N766-	ER membrane prc	EMC1
25.6422	26.1275	25.7217	25.6913	25.8029	25.6343	25.7278	26.2923	26.126	25.8368	25.7193	25.3332	Q15006;H0YAS9	Q15006	ER membrane prc	EMC2
NaN	NaN	NaN	NaN	NaN	NaN	22.4135	NaN	NaN	NaN	NaN	NaN	S4R329;Q5J8M3-3	S4R329;Q5J8M3-3	ER membrane prc	EMC4
NaN	NaN	23.114	23.1946	23.5989	23.2956	NaN	24.2748	24.2819	NaN	23.547	23.7198	Q9NPA0;H0YDT8;	Q9NPA0;H0YDT8;	ER membrane prc	EMC7
23.0934	NaN	23.2559	23.6257	NaN	NaN	23.5097	NaN	NaN	23.5384	23.4037	NaN	O43402;M0R1B0	O43402;M0R1B0	ER membrane prc	EMC8
24.5841	24.7047	24.9888	24.3778	24.3693	25.1714	24.223	24.8216	24.0879	24.6081	24.0233	24.0984	P50402;Q5HY57	P50402;Q5HY57	Emerin	EMD
25.9625	24.005	24.1136	25.7732	24.2852	24.354	23.4285	NaN	NaN	25.8688	26.1318	NaN	Q92979;A0A087W	Q92979;A0A087W	Ribosomal RNA sr	EMG1
24.0647	24.8177	24.6701	23.6658	23.8962	24.2414	23.8339	NaN	25.6288	23.487	23.5387	NaN	O95834-3;O95834	O95834-3;O95834	Echinoderm micro	EML2
25.6929	25.8487	25.838	24.6401	25.2069	25.3928	25.4841	26.3501	26.0542	24.8037	25.2376	25.5945	Q9HC35;B5MBZ0;	Q9HC35;B5MBZ0;	Echinoderm micro	EML4
24.46	25.2957	25.4418	24.1772	24.9805	24.8875	24.7372	NaN	24.6948	24.6019	24.8761	25.3345	Q8N8S7;Q8N8S7-	Q8N8S7;Q8N8S7-	Protein enabled h	ENAH
23.2938	23.2248	23.7895	23.5252	23.5871	23.8454	23.4761	23.5444	NaN	23.7908	23.4003	24.4733	O94919	O94919	Endonuclease dor	ENDOD1
28.6714	28.3625	29.1599	29.0597	28.5646	29.3199	29.1247	28.275	28.791	29.8436	28.9445	28.9607	P17813;P17813-2	P17813;P17813-2	Endoglin	ENG
34.1362	34.6952	34.1947	33.6656	34.2181	34.1488	34.502	34.139	33.8441	34.0992	34.0876	34.1654	P06733;P06733-2	P06733;P06733-2	Alpha-enolase	ENO1
27.9478	29.3659	28.5271	27.656	28.9902	28.6028	28.1055	28.7544	28.693	28.0032	28.4214	28.9805	P09104;P09104-2	P09104;P09104-2	Gamma-enolase;E	ENO2
NaN	NaN	25.3109	NaN	NaN	25.2008	NaN	NaN	NaN	NaN	NaN	NaN	P13929;P13929-3	P13929;P13929-3	Beta-enolase;Eno	ENO3
25.4801	26.6861	25.9422	24.9626	26.2604	25.6905	27.5021	NaN	NaN	25.6828	25.892	NaN	Q9UHY7;A0A0C4E	Q9UHY7;A0A0C4E	Enolase-phosphat	ENOPH1
28.0238	28.7828	29.2163	28.3319	28.818	29.3588	29.3994	28.5342	28.8897	29.6023	28.9828	28.7127	P22413;E9PE72	P22413	Ectonucleotide py	ENPP1
24.9289	23.9802	24.5958	24.904	23.4806	24.4368	24.9401	NaN	NaN	23.6658	23.0343	NaN	Q9Y6X5;Q6UWV6	Q9Y6X5	Bis(5-adenosyl)-tr	ENPP4
NaN	24.4133	24.2122	NaN	24.217	24.1323	NaN	NaN	NaN	NaN	NaN	NaN	O43768;P56211;H	O43768;P56211;H	Alpha-endosulfine	ENSA;ARPP19
23.3332	23.2332	23.499	23.3884	22.943	23.3965	NaN	NaN	NaN	22.9046	NaN	NaN	O75355;O75355-2	O75355;O75355-2	Ectonucleoside tri	ENTPD3
24.1482	25.0999	24.8143	24.0893	25.0702	24.282	24.5384	24.9881	NaN	24.1045	25.1623	25.0036	Q9NPA8;Q9NPA8	Q9NPA8;Q9NPA8	Transcription and	ENY2
NaN	NaN	NaN	22.5157	NaN	NaN	NaN	NaN	NaN	21.3659	NaN	NaN	Q09472;Q92793;C	Q09472	Histone acetyltrar	EP300
23.7523	24.3448	24.2549	23.6061	23.3877	24.1612	NaN	NaN	NaN	24.0393	23.4548	NaN	P11171;P11171-2	P11171;P11171-2	Protein 4.1	EPB41
24.5694	24.7635	25.446	24.5524	24.4904	25.3679	25.0169	NaN	NaN	25.5402	25.407	25.1907	Q9H4G0-2;Q9H4G	Q9H4G0-2;Q9H4G	Band 4.1-like prot	EPB41L1
23.9088	24.7005	25.4903	24.7195	24.9669	25.573	25.2796	24.998	24.5756	26.3418	26.2249	26.026	E9PHY5;O43491-4	E9PHY5;O43491-4	Band 4.1-like prot	EPB41L2
NaN	NaN	NaN	20.6144	NaN	NaN	NaN	NaN	NaN	NaN	NaN	NaN	H0Y5B0;E9PMG5	H0Y5B0		EPB41L2
29.1414	29.7371	30.2718	29.71	30.1624	30.6517	29.4741	28.8714	29.2984	30.5633	30.2527	30.491	O43491;Q6R5J7;C	O43491	Band 4.1-like prot	EPB41L2
NaN	NaN	NaN	22.9043	NaN	NaN	NaN	NaN	NaN	NaN	NaN	NaN	Q9Y2J2;J3QS55;J3	Q9Y2J2	Band 4.1-like prot	EPB41L3
29.4917	29.0572	28.9073	29.348	28.9136	28.9451	27.3914	26.8001	27.1096	27.0454	27.6856	27.9556	Q9Y2J2-2;A8K968	Q9Y2J2-2;A8K968	Band 4.1-like prot	EPB41L3
NaN	NaN	22.3409	21.8036	NaN	22.0874	NaN	NaN	NaN	22.2407	NaN	NaN	Q9H329;Q9H329-	Q9H329;Q9H329-	Band 4.1-like prot	EPB41L4B
23.8502	24.2864	25.1068	24.3275	24.4056	25.2417	24.5317	NaN	23.7689	24.5124	24.2119	25.0198	Q9HCM4;Q9HCM	Q9HCM4;Q9HCM	Band 4.1-like prot	EPB41L5
25.1302	24.4569	25.1227	24.8242	24.3723	25.3065	NaN	NaN	24.836	NaN	NaN	25.1536	P16422;B5MCA4;	P16422;B5MCA4	Epithelial cell adh	EPCAM
NaN	NaN	NaN	21.6702	NaN	NaN	NaN	NaN	NaN	NaN	NaN	NaN	Q9UM22-2;Q9UN	Q9UM22-2;Q9UN	Mammalian epen	EPDR1
26.3915	24.7149	25.3939	25.595	24.3529	24.7066	24.7891	24.5355	NaN	24.5433	23.4339	NaN	Q9HCE0;Q9HCE0-	Q9HCE0;Q9HCE0-	Ectopic P granules	EPG5
31.9246	31.6346	32.3364	32.0313	31.5217	32.5008	31.2577	30.2919	30.8863	31.7526	31.4815	31.1991	P29317;P29317-2	P29317	Ephrin type-A rec	EPHA2
NaN	NaN	NaN	NaN	NaN	NaN	NaN	NaN	NaN	NaN	NaN	21.8991	P54756;F8W9W0;	P54756;F8W9W0;	Ephrin type-A rec	EPHA5;EPHA3
26.0524	26.4607	27.0666	26.342	26.338	27.1745	26.4433	25.0334	25.5492	27.4219	26.3466	25.9377	P29323;P29323-2	P29323;P29323-2	Ephrin type-B rec	EPHB2
22.3024	NaN	23.4343	23.1331	NaN	NaN	NaN	NaN	NaN	22.6447	NaN	NaN	P54753	P54753	Ephrin type-B rec	EPHB3
25.0376	24.896	25.8466	25.3187	25.1612	26.0663	25.3989	NaN	25.0319	26.45	25.9283	25.668	P54760;Q96L35;P	P54760;Q96L35	Ephrin type-B rec	EPHB4
23.3893	23.6893	23.6711	23.6852	23.5783	NaN	24.3837	NaN	24.7396	24.4765	24.0859	23.5629	P07099;B1AQP8;E	P07099	Epoxide hydrolase	EPHX1
NaN	NaN	NaN	22.3854	NaN	NaN	NaN	NaN	NaN	NaN	NaN	NaN	Q8IUS5	Q8IUS5	Epoxide hydrolase	EPHX4
24.3549	23.61	23.9819	24.0187	23.4233	23.5561	24.1185	24.8648	24.237	25.045	25.3502	24.8439	Q7L775;A0A096LI	Q7L775	EPM2A-interactin	EPM2AIP1
23.3494	23.4964	23.3461	22.9527	23.1336	23.2901	23.7191	NaN	23.973	NaN	23.8713	NaN	Q9Y6I3;Q9Y6I3-3;	Q9Y6I3;Q9Y6I3-3;	Epsin-1	EPN1
22.5721	23.4473	23.1753	22.9591	23.2232	23.3473	NaN	NaN	NaN	NaN	NaN	NaN	O95208-2;F6PQP	O95208-2;F6PQP	Epsin-2	EPN2
30.5477	30.0799	30.0812	30.3923	30.2503	30.0475	29.8863	30.2908	29.7998	30.236	30.1649	29.8368	P07814;V9GYZ6;V	P07814;V9GYZ6	Bifunctional gluta	EPRS

23.0229	23.1684	23.0678	NaN	22.8998	NaN	NaN	NaN	NaN	NaN	NaN	NaN	P42566;B1AUU8;I	P42566;B1AUU8;I	Epidermal growth	EPS15
25.1268	25.6206	25.6723	25.1699	25.6657	25.1987	25.4449	25.5017	25.4062	25.2034	25.1054	25.7799	Q9UBC2;Q9UBC2-	Q9UBC2;Q9UBC2-	Epidermal growth	EPS15L1
23.614	23.7866	24.1413	23.4674	23.6455	23.8083	NaN	NaN	NaN	NaN	NaN	NaN	Q12929;Q12929-2	Q12929;Q12929-2	Epidermal growth	EPS8
26.5996	26.4035	26.9268	26.3896	26.3964	26.3748	26.0877	23.3151	NaN	25.9413	25.5288	24.2905	Q9H6S3;Q9H6S3-	Q9H6S3;Q9H6S3-	Epidermal growth	EPS8L2
23.2043	NaN	23.1828	23.4434	NaN	NaN	NaN	NaN	NaN	23.0645	22.7107	NaN	Q9C0D9;A0A087V	Q9C0D9;A0A087V	Ethanolaminepho	EPT1
NaN	NaN	NaN	21.8597	NaN	NaN	NaN	NaN	NaN	NaN	NaN	NaN	O75616;K7EQJ8;J	O75616;K7EQJ8	GTPase Era, mitoc	ERAL1
25.405	26.1678	25.9855	25.2608	25.9404	25.8984	27.1002	26.6874	26.0468	25.2565	25.1294	24.4996	Q9NZ08;Q9NZ08-	Q9NZ08;Q9NZ08-	Endoplasmic retic	ERAP1
25.439	25.5424	25.9337	25.4938	25.3274	26.0727	NaN	NaN	NaN	25.7154	25.5411	25.1821	P04626;B4DTR1;J	P04626;B4DTR1;J	Receptor tyrosine	ERBB2
27.87	28.0888	28.2516	27.7327	27.9142	28.1354	28.2163	27.8747	27.9189	28.4834	28.1886	28.1937	Q96RT1;Q96RT1-8	Q96RT1;Q96RT1-8	Protein LAP2	ERBB2IP
23.8292	24.2614	23.9612	23.5851	23.9985	23.5624	23.6078	24.3441	NaN	23.2604	23.4367	24.197	Q8IUD2;G8JLD3;X	Q8IUD2;G8JLD3;X	ELKS/Rab6-interac	ERC1
22.0534	NaN	NaN	22.462	NaN	22.1015	NaN	NaN	NaN	NaN	NaN	NaN	P18074;E7EVE9;A	P18074;E7EVE9;A	TFIIH basal transc	ERCC2
24.1041	NaN	23.6525	24.5	24.3719	23.9047	NaN	NaN	NaN	23.6434	23.6057	24.4362	P19447;H7C309	P19447	TFIIH basal transc	ERCC3
25.2485	23.7299	24.9082	25.0611	23.7947	24.351	24.1279	24.0346	NaN	24.2371	24.2888	NaN	Q2NKX8;B5MDQ0	Q2NKX8;B5MDQ0	DNA excision repa	ERCC6L
NaN	NaN	NaN	NaN	NaN	NaN	24.2009	NaN	NaN	NaN	NaN	NaN	O14944	O14944	Proepiregulin;Epir	EREG
26.0356	25.0017	25.2121	25.6582	25.2488	25.1532	24.668	24.5308	25.4126	24.9384	24.4181	24.5835	Q969X5;Q969X5-2	Q969X5;Q969X5-2	Endoplasmic retic	ERGIC1
24.3964	23.9313	23.4197	24.0788	23.6093	NaN	NaN	NaN	NaN	23.2867	NaN	NaN	Q96RQ1;A0A087V	Q96RQ1;A0A087V	Endoplasmic retic	ERGIC2
25.3011	24.5588	24.7062	25.0592	24.2727	24.2477	24.4626	NaN	24.1733	24.1604	23.7043	NaN	Q9Y282;H0Y5K5;C	Q9Y282;H0Y5K5;C	Endoplasmic retic	ERGIC3
28.0376	28.4931	27.9866	27.7496	28.0403	28.1204	27.7681	28.445	27.6111	28.4069	28.5621	28.2837	P84090;G3V279	P84090;G3V279	Enhancer of rudim	ERH
23.2595	22.4322	23.0585	22.7677	22.5187	23.0477	22.0488	NaN	NaN	NaN	NaN	NaN	Q8IV48	Q8IV48	3-5 exoribonuclea	ERI1
22.1444	NaN	NaN	22.2445	NaN	22.4757	NaN	NaN	NaN	NaN	NaN	NaN	Q96DZ1;Q96DZ1-	Q96DZ1;Q96DZ1-	Endoplasmic retic	ERLEC1
27.6017	27.7798	27.5099	27.3763	27.5523	27.7015	27.3892	27.381	27.5645	27.6676	27.2589	27.4537	O75477;B0QZ43	O75477;B0QZ43	Erlin-1	ERLIN1
25.3046	26.7881	26.0349	25.4883	26.6492	26.1042	25.6942	26.4445	26.6224	25.2271	25.6571	25.9532	O94905;E5RHW4;	O94905;E5RHW4;	Erlin-2	ERLIN2
28.9866	29.0974	28.9467	28.889	28.9306	28.9346	28.9212	28.93	29.3011	28.8346	28.6186	28.529	Q96HE7;G3V3E6;(	Q96HE7	ERO1-like protein	ERO1L
28.71	29.629	28.8225	28.6993	29.4008	28.7379	29.3937	30.0517	29.6515	29.1335	29.2056	29.0852	P30040;F8VY02;F	P30040;F8VY02	Endoplasmic retic	ERP29
25.7672	26.7376	25.9858	25.797	26.3125	26.1069	26.275	27.0021	27.1525	25.7679	26.0363	26.3224	Q9BS26	Q9BS26	Endoplasmic retic	ERP44
28.9652	29.603	29.4826	29.323	29.5215	29.6482	29.2088	28.976	29.0965	30.1891	29.6481	29.8511	Q96AP7;F8WDW9	Q96AP7;F8WDW9	Endothelial cell-se	ESAM
27.0363	27.6775	27.1999	26.768	27.6209	27.2013	27.166	27.3917	27.7732	27.3878	27.6811	28.1207	P10768;H7BZT7;X	P10768;H7BZT7;X	S-formylglutathio	ESD
21.7569	NaN	NaN	NaN	NaN	NaN	NaN	NaN	NaN	22.227	22.6285	NaN	Q14674;Q14674-2	Q14674;Q14674-2	Separin	ESPL1
28.3089	28.6285	28.5652	28.2745	28.4962	28.5284	28.8023	28.7626	28.8131	28.4987	28.3846	28.4309	Q9BSJ8;Q9BSJ8-2	Q9BSJ8;Q9BSJ8-2	Extended synaptc	ESYT1
27.3926	27.2266	27.6881	27.3543	27.4651	27.8065	27.7424	27.8261	27.9199	27.857	27.5079	27.626	A0FGR8-2;H7BXI1	A0FGR8-2;H7BXI1	Extended synaptc	ESYT2
26.6094	26.3424	26.2822	26.1942	26.5669	26.445	26.504	26.3355	25.9452	26.5727	26.6903	26.399	P62495;B7Z7P8;P	P62495;B7Z7P8;P	Eukaryotic peptid	ETF1
27.1496	26.5845	26.1207	26.9394	26.4916	26.2058	25.2773	24.5543	25.3717	26.1432	26.1882	25.478	P13804;P13804-2	P13804;P13804-2	Electron transfer f	ETFA
26.6544	26.81	26.3392	26.3861	26.826	26.539	25.2886	NaN	25.5446	25.661	26.1915	25.5899	P38117;M0QY67	P38117;M0QY67	Electron transfer f	ETFB
23.6424	NaN	22.7835	23.0966	NaN	NaN	NaN	NaN	NaN	23.1402	23.8382	NaN	O95571;M0QXB5;	O95571;M0QXB5;	Persulfide dioxyge	ETHE1
NaN	NaN	NaN	21.4213	NaN	NaN	NaN	NaN	NaN	NaN	NaN	NaN	Q9HBU6;H0YH69	Q9HBU6;H0YH69	Ethanolamine kina	ETNK1
23.2078	NaN	23.6262	23.731	23.5074	24.2577	NaN	NaN	NaN	24.1645	24.4007	24.9506	Q9H8M9;B8ZZF5;	Q9H8M9;B8ZZF5;	Protein eva-1 hon	EVA1A
24.4885	24.4764	24.8103	24.2315	24.5039	24.7572	24.2654	NaN	25.2003	24.2317	24.4984	25.3009	P22794;J3QQH9;J	P22794;J3QQH9;J	Protein EVI2A	EVI2A
25.2239	24.9083	25.1483	25.0981	25.1584	25.5772	24.8914	24.4076	24.8352	25.3152	25.0226	25.265	P34910;P34910-2	P34910;P34910-2	Protein EVI2B	EVI2B
24.1029	23.822	24.0705	23.9093	NaN	23.3092	NaN	NaN	NaN	NaN	NaN	NaN	O60447;O60447-2	O60447;O60447-2	Ecotropic viral inte	EVI5
26.5593	25.7551	25.5879	26.1645	25.4349	25.6773	24.7333	25.3907	24.9811	26.0119	25.125	25.8114	Q01844;B0QYK0;(	Q01844;B0QYK0;(	RNA-binding prote	EWSR1
27.5895	27.0177	27.1301	27.4464	26.8248	27.0921	26.7117	25.9317	25.6313	27.1641	26.4898	26.3163	Q9NV70;Q9NV70-	Q9NV70;Q9NV70-	Exocyst complex c	EXOC1
27.5715	27.1209	27.2566	27.0232	27.0184	27.3409	26.9796	27.121	28.7046	27.4304	26.9569	26.7507	Q96KP1;Q2MDF5	Q96KP1	Exocyst complex c	EXOC2
26.8616	27.0683	26.7423	26.768	27.2961	26.6652	25.9844	25.0176	25.7236	26.9903	26.3746	25.9148	O60645-3;O60645	O60645-3;O60645	Exocyst complex c	EXOC3
27.2463	26.9535	27.4164	27.3454	27.0233	27.597	26.4939	26.8574	26.6823	27.3681	27.1903	26.9478	Q96A65;Q96A65-	Q96A65	Exocyst complex c	EXOC4
26.5121	26.3465	26.558	26.4124	26.2313	26.5751	26.1993	25.6881	26.0606	26.4273	26.19	26.1977	O00471;A0A0A0M	O00471;A0A0A0M	Exocyst complex c	EXOC5
24.6923	24.208	24.4524	24.4546	24.2315	24.8558	24.0649	NaN	NaN	24.5917	24.5969	23.7495	Q8TAG9;E7EW84;	Q8TAG9;E7EW84;	Exocyst complex c	EXOC6
25.8199	25.6072	26.0586	25.365	25.3552	25.5097	25.4591	25.5216	25.164	25.6101	25.501	24.5708	Q9Y2D4;A0A0U1F	Q9Y2D4;A0A0U1F	Exocyst complex c	EXOC6B
27.5122	28.0408	27.8371	27.3896	27.8489	27.7988	27.1965	27.4175	27.1573	27.3302	27.3072	27.2603	Q9UPT5;A0A0A0M	Q9UPT5;A0A0A0M	Exocyst complex c	EXOC7
27.0799	27.3352	27.3618	27.0059	27.2837	27.447	26.7747	27.1581	26.6948	27.3468	27.0581	26.9438	Q8IYI6	Q8IYI6	Exocyst complex c	EXOC8
25.2927	24.4298	24.1516	25.1827	24.4283	24.3829	23.3998	NaN	23.4668	24.9943	25.2922	24.3173	Q9Y3B2;B1AMU3;	Q9Y3B2;B1AMU3;	Exosome complex	EXOSC1
27.0153	25.9202	26.3204	27.1579	26.2647	26.4727	NaN	NaN	NaN	26.3637	26.8045	24.6601	Q01780;Q01780-2	Q01780;Q01780-2	Exosome compon	EXOSC10
25.0444	24.8542	24.248	24.9322	25.2595	24.6988	23.7692	NaN	NaN	24.961	25.4306	25.3335	Q13868;Q13868-3	Q13868;Q13868-3	Exosome complex	EXOSC2
24.345	23.9199	23.9604	24.8482	24.3357	24.2018	NaN	NaN	NaN	24.3806	24.9292	24.8837	Q9NQT5;Q9NQT5	Q9NQT5;Q9NQT5	Exosome complex	EXOSC3
26.0735	25.8538	25.2747	25.9755	25.9495	25.5608	25.1206	25.8458	24.5267	26.1815	26.2525	25.5076	Q9NPD3;E9PPI9;E	Q9NPD3;E9PPI9;E	Exosome complex	EXOSC4
23.8346	23.226	23.1075	24.2099	23.8732	NaN	22.9032	NaN	NaN	23.7454	24.4531	24.0081	Q9NQT4;M0R050	Q9NQT4;M0R050	Exosome complex	EXOSC5
26.2265	26.1857	25.6425	26.0471	26.4135	25.75	25.02	25.2064	25.3177	26.3844	26.9308	26.366	Q5RKV6	Q5RKV6	Exosome complex	EXOSC6
22.8616	23.2212	23.1039	23.3941	22.7349	23.0303	NaN	23.377	22.1929	23.7221	23.9645	22.8153	Q15024	Q15024	Exosome complex	EXOSC7
23.4616	23.1287	23.2744	23.5018	23.5635	23.5374	NaN	NaN	NaN	23.4107	24.2758	23.7832	Q96B26;H7C581	Q96B26	Exosome complex	EXOSC8
24.184	23.0367	23.5157	23.8992	23.3286	23.5932	NaN	NaN	NaN	NaN	23.9874	NaN	Q06265;Q06265-2	Q06265;Q06265-2	Exosome complex	EXOSC9
22.4381	NaN	22.8182	23.1773	22.6212	NaN	26.1959	23.9912	23.4196	22.6994	NaN	NaN	Q16394;H7C1H6	Q16394	Exostosin-1	EXT1

23.6127	23.6616	23.9232	23.6491	23.1138	23.1946	25.7242	25.9121	23.2333	23.5171	23.3581	NaN	Q93063;Q93063-2	Q93063;Q93063-2	Exostosin-2	EXT2
22.9788	23.8859	22.9423	22.8242	NaN	23.137	24.1297	NaN	NaN	22.8913	23.5627	23.8623	Q9UBQ6;C9JEG3;	Q9UBQ6	Exostosin-like 2;P	EXTL2
23.4527	NaN	NaN	23.3903	NaN	NaN	NaN	NaN	NaN	NaN	NaN	NaN	Q15910;Q15910-2	Q15910;Q15910-2	Histone-lysine N-r	EZH2
32.2953	33.2379	33.7297	32.9151	33.6124	34.1143	32.5009	31.9624	31.8658	34.0472	33.736	32.7171	P15311;E7EQR4	P15311;E7EQR4	Ezrin	EZR
24.1355	25.1668	23.549	24.4212	25.1536	23.5698	24.1839	NaN	26.1751	NaN	NaN	NaN	P00742;F8WBM7;	P00742	Coagulation facto	F10
26.3867	26.5507	27.1954	26.51	26.7581	27.2134	26.1214	25.5515	26.1512	25.9862	25.4294	NaN	Q9Y624;A0A087W	Q9Y624;A0A087W	Junctional adhesic	F11R
NaN	NaN	NaN	NaN	20.8484	NaN	NaN	NaN	NaN	NaN	NaN	NaN	P00488	P00488	Coagulation facto	F13A1
24.2045	24.2351	NaN	23.8169	23.0417	NaN	NaN	NaN	NaN	NaN	NaN	NaN	P25116	P25116	Proteinase-activat	F2R
23.1807	23.2771	23.4077	22.7723	23.3756	23.5262	NaN	NaN	NaN	NaN	NaN	NaN	P55085;D6RJH3	P55085	Proteinase-activat	F2RL1
29.0963	28.481	29.0891	29.2939	28.4522	28.9263	28.2996	27.4949	27.8767	28.7771	27.84	27.6059	P13726;P13726-2	P13726	Tissue factor	F3
NaN	NaN	NaN	NaN	NaN	NaN	NaN	NaN	NaN	26.4371	26.4611	NaN	P15090	P15090	Fatty acid-binding	FABP4
26.5597	27.2308	26.9458	26.6435	26.8165	26.7477	28.228	27.3974	27.6051	27.4117	27.4731	27.5405	Q01469;I6L8B7;A	Q01469	Fatty acid-binding	FABP5
23.8579	23.2511	23.5117	23.4759	22.9307	23.5051	25.0172	23.9249	23.9512	24.3764	23.6356	23.0761	O95864;O95864-3	O95864;O95864-3	Fatty acid desatur	FADS2
23.9137	NaN	23.6464	NaN	NaN	NaN	NaN	NaN	NaN	NaN	NaN	NaN	Q9UNN5;Q9UNN5	Q9UNN5;Q9UNN5	FAS-associated fa	FAF1
25.985	25.8185	25.3259	25.5411	25.2848	25.5301	25.4548	NaN	25.7995	25.239	25.0143	NaN	Q96CS3	Q96CS3	FAS-associated fa	FAF2
25.8485	27.4894	27.0767	25.5117	27.2219	26.5929	26.1462	NaN	23.4214	25.4629	24.4466	25.7469	P16930;P16930-2	P16930;P16930-2	Fumarylacetoacet	FAH
NaN	NaN	NaN	NaN	21.3948	NaN	NaN	NaN	NaN	NaN	NaN	NaN	Q6P587;Q6P587-2	Q6P587;Q6P587-2	Acylpyruvase FAH	FAHD1
24.154	22.7998	22.9814	23.8085	22.9974	22.722	23.3831	23.5841	23.5221	23.6642	NaN	NaN	Q96GK7;C9JGM0;	Q96GK7;C9JGM0	Fumarylacetoacet	FAHD2A
21.7443	NaN	NaN	NaN	NaN	NaN	NaN	NaN	NaN	NaN	NaN	NaN	Q9BTL3	Q9BTL3	RNMT-activating	FAM103A1
NaN	24.7642	24.8027	NaN	24.7191	24.6021	NaN	NaN	NaN	NaN	NaN	NaN	Q9H098;Q9H098-	Q9H098;Q9H098-	Protein FAM107B	FAM107B
24.077	NaN	24.061	24.3274	24.0027	24.0121	NaN	NaN	NaN	NaN	24.415	NaN	Q96PZ2;E9PR18;E	Q96PZ2;E9PR18	Protein FAM111A	FAM111A
22.7852	22.3272	22.1275	22.8851	NaN	NaN	NaN	23.5384	NaN	22.0598	NaN	NaN	Q6SJ93;Q6SJ93-2;	Q6SJ93;Q6SJ93-2	Protein FAM111B	FAM111B
24.7285	25.2122	24.582	24.626	24.6442	24.7063	24.4307	24.4731	24.8318	24.3558	23.8735	24.8246	Q8IWE2	Q8IWE2	Protein NOXP20	FAM114A1
23.168	23.0961	22.8589	22.9787	22.886	22.9572	NaN	NaN	NaN	22.4554	NaN	NaN	Q9NRY5;I6L9D5;E	Q9NRY5;I6L9D5;E	Protein FAM114A	FAM114A2
27.8915	26.9271	27.2706	27.7411	27.3279	27.2514	26.4051	27.273	26.4946	26.9704	26.9738	26.6919	Q9NZB2;Q9NZB2-	Q9NZB2;Q9NZB2-	Constitutive coact	FAM120A
21.9819	NaN	NaN	21.9563	NaN	21.8657	NaN	NaN	NaN	21.8403	22.1307	NaN	Q96EK7;A0A0D9S	Q96EK7;A0A0D9S	Constitutive coact	FAM120B
24.9291	25.0125	25.8435	25.2462	24.9297	25.957	24.9871	NaN	24.8384	25.9315	25.4515	24.8943	Q9BYI3-3;H7C0W	Q9BYI3-3;H7C0W	Hyccin	FAM126A
21.7668	21.4956	21.8587	21.8346	21.7658	21.4924	NaN	NaN	NaN	NaN	NaN	NaN	A6ZKI3;Q17RB0;Q	A6ZKI3	Protein FAM127A	FAM127A
28.6538	28.673	28.7829	27.9535	28.4684	28.5712	28.7541	28.0112	28.4537	27.9402	27.9014	28.1365	Q9BZQ8;H0Y7M9	Q9BZQ8	Protein Niban	FAM129A
28.9647	29.5141	30.0368	29.0586	29.6212	30.2077	29.5571	28.6021	29.0819	29.9646	29.7576	29.8528	Q96TA1;Q96TA1-2	Q96TA1;Q96TA1-2	Niban-like protein	FAM129B
NaN	NaN	22.9646	22.8704	NaN	NaN	23.6533	NaN	23.5094	NaN	NaN	NaN	Q86VR2;K7ENZ6;	Q86VR2;K7ENZ6;	Protein FAM134C	FAM134C
25.0816	25.6492	25.1337	25.141	25.7923	25.2186	25.0508	26.0577	25.866	24.6903	25.3477	25.6576	Q96C01;C9J2Y4;E	Q96C01;C9J2Y4;E	Protein FAM136A	FAM136A
24.1333	23.4747	23.7278	24.0198	23.4059	23.2691	23.439	NaN	NaN	23.5377	23.3077	NaN	Q5W0V3;Q5W0V3	Q5W0V3;Q5W0V3	Protein FAM160B	FAM160B1
25.4763	25.3825	26.0493	25.0723	25.3208	26.1222	25.8906	25.0484	25.3802	26.3199	25.8388	25.9922	Q5VUB5	Q5VUB5	Protein FAM171A	FAM171A1
NaN	NaN	25.2732	NaN	NaN	NaN	NaN	NaN	NaN	NaN	NaN	NaN	A8MVW0;K7EMG	A8MVW0	Protein FAM171A	FAM171A2
NaN	NaN	22.4525	NaN	NaN	NaN	NaN	NaN	NaN	NaN	NaN	NaN	Q6P995	Q6P995	Protein FAM171B	FAM171B
26.0987	25.6293	25.6583	25.7573	25.9436	25.86	25.5586	25.8509	25.841	25.8005	25.7508	25.4168	Q15018	Q15018	BRISC complex su	FAM175B
23.3748	23.0645	23.1405	23.1668	23.2069	23.3588	NaN	NaN	NaN	NaN	NaN	NaN	I3L317;C9JLW8;I3	I3L317;C9JLW8;I3	Protein FAM195B	FAM195B
NaN	NaN	NaN	NaN	NaN	NaN	23.9119	NaN	NaN	NaN	NaN	NaN	Q9UK61;Q9UK61-	Q9UK61;Q9UK61-	Protein FAM208A	FAM208A
25.0459	24.5265	25.0844	25.1379	24.5861	24.7613	25.2185	NaN	NaN	24.7312	24.4986	NaN	Q9BRX8;Q9BRX8-	Q9BRX8;Q9BRX8-	Redox-regulatory	FAM213A
24.0164	24.3155	24.2517	24.1762	24.1017	23.6893	24.6996	23.9143	NaN	24.0692	22.9585	NaN	Q641Q2;F8W7U3	Q641Q2;F8W7U3	WASH complex su	FAM21A;FAM21C
25.6402	26.4334	25.6683	25.5093	25.9407	25.7198	27.6679	27.0562	27.0427	25.6822	25.1112	26.1408	Q92520;C9JP35;C	Q92520	Protein FAM3C	FAM3C
24.1059	24.2058	24.1632	NaN	24.0453	24.0304	24.2116	23.3324	25.0304	NaN	23.2745	24.9044	Q8TCE6;Q8TCE6-2	Q8TCE6;Q8TCE6-2	Protein FAM45A;F	FAM45A;FAM45B
NaN	NaN	NaN	NaN	NaN	NaN	NaN	NaN	NaN	22.0013	NaN	NaN	Q9H0Q0	Q9H0Q0	Protein FAM49A	FAM49A
27.9266	27.9243	28.0304	27.4771	27.7161	28.0611	28.3112	26.9896	27.7864	28.5391	27.8581	28.0423	Q9NUQ9;Q9NUQ	Q9NUQ9;Q9NUQ	Protein FAM49B	FAM49B
25.2401	25.6343	25.6124	24.9152	25.4046	25.9552	NaN	NaN	24.9337	25.9237	25.6923	25.7303	Q14320;B0S8I6;Q	Q14320;B0S8I6	Protein FAM50A	FAM50A
NaN	NaN	NaN	NaN	NaN	NaN	NaN	NaN	NaN	NaN	NaN	22.5671	Q9BSJ6;I3L419;I3	Q9BSJ6;I3L419;I3	Protein FAM64A	FAM64A
25.5183	24.9443	25.5547	25.0934	25.0111	25.3876	25.578	25.383	25.5863	25.5516	25.1665	25.2612	Q6ZS17;Q6ZS17-2	Q6ZS17;Q6ZS17-2	Protein FAM65A	FAM65A
22.5952	NaN	NaN	22.5788	NaN	NaN	NaN	NaN	NaN	NaN	NaN	NaN	Q9H4H8;A0A087V	Q9H4H8;A0A087V	Protein FAM83D	FAM83D
22.7206	NaN	NaN	NaN	NaN	NaN	NaN	NaN	NaN	NaN	NaN	NaN	A6ND36;A6ND36-	A6ND36	Protein FAM83G	FAM83G
26.3896	25.5314	25.786	26.1245	25.3778	25.6105	24.7181	24.5326	24.5806	25.295	24.8607	24.6972	Q658Y4;E7ER68;H	Q658Y4;E7ER68	Protein FAM91A1	FAM91A1
23.6992	NaN	23.0499	23.3076	NaN	NaN	NaN	NaN	NaN	NaN	NaN	NaN	Q9H5X1;H0YKV4;	Q9H5X1;H0YKV4;	MIP18 family prot	FAM96A
26.1618	25.6904	25.4824	25.9909	25.3852	25.4903	25.1787	25.2471	25.0637	25.1483	25.0001	25.2013	Q9Y3D0;H3BNV7;	Q9Y3D0;H3BNV7	Mitotic spindle-as	FAM96B
25.8535	25.1342	24.8451	25.3884	24.8457	25.24	24.4937	NaN	25.0503	24.6131	25.0648	24.3616	Q8NCA5;Q8NCA5	Q8NCA5;Q8NCA5	Protein FAM98A	FAM98A
24.3903	24.072	24.0427	24.3089	24.044	NaN	NaN	23.7624	22.7085	24.5715	24.5738	24.0845	Q52LJ0-2;Q52LJ0;	Q52LJ0-2;Q52LJ0	Protein FAM98B	FAM98B
19.5214	NaN	NaN	NaN	NaN	NaN	NaN	NaN	NaN	NaN	NaN	NaN	Q17RN3;K7EQL1;I	Q17RN3;K7EQL1;I	Protein FAM98C	FAM98C
25.3545	23.9593	24.4439	25.0814	NaN	23.8463	23.9806	NaN	NaN	NaN	NaN	NaN	Q8WVX9;E9PNW8	Q8WVX9;E9PNW8	Fatty acyl-CoA rec	FAR1
26.6508	26.7804	27.7806	26.9792	26.7903	27.9654	26.9601	26.4399	26.063	27.6065	27.0962	26.7589	Q9Y4F1;C9JME2;C	Q9Y4F1;C9JME2;C	FERM, RhoGEF an	FARP1
23.6745	23.8415	24.8688	23.922	23.648	25.0478	NaN	NaN	NaN	24.7414	24.2431	24.0056	O94887;O94887-3	O94887;O94887-3	FERM, RhoGEF an	FARP2
27.7993	27.7783	27.5334	27.7568	28.156	27.6921	26.7891	27.5756	27.1171	27.7211	28.4193	27.121	Q9Y285;K7ER00;C	Q9Y285;K7ER00;C	Phenylalanine--tR	FARSA

28.166	27.6736	27.5102	28.1273	28.1281	27.6277	26.902	27.9394	27.682	27.8707	28.5069	27.8343	Q9NSD9;Q9NSD9-	Q9NSD9;Q9NSD9-	Phenylalanine--tR	FARSB
NaN	NaN	NaN	NaN	NaN	NaN	NaN	NaN	NaN	23.0926	NaN	NaN	P25445;P25445-6	P25445;P25445-6	Tumor necrosis fa	FAS
32.8866	31.9493	32.45	32.8983	32.1176	32.3727	33.0482	33.2725	32.5421	33.0687	32.9198	32.5087	P49327;A0A0U1R	P49327;A0A0U1R	Fatty acid synthas	FASN
22.7516	NaN	NaN	22.4435	NaN	21.4318	NaN	NaN	NaN	NaN	NaN	NaN	Q9NYY8;Q9NYY8-	Q9NYY8;Q9NYY8-	FAST kinase doma	FASTKD2
25.027	24.8057	24.8973	24.4129	24.3316	23.5701	26.8409	26.2223	26.5378	27.4885	26.6103	26.8774	Q14517;A0A087W	Q14517;A0A087W	Protocadherin Fat	FAT1
25.2135	24.5699	25.4007	24.6982	24.7398	24.8382	25.3118	26.7085	25.6579	25.1516	25.982	25.5038	P62861;E9PR30	P62861;E9PR30	40S ribosomal prc	FAU
28.819	28.2194	28.6382	29.5218	29.0632	28.9939	27.0748	27.6167	27.2922	29.1878	29.3115	28.4507	P22087;M0QXL5;I	P22087;M0QXL5;I	rRNA 2-O-methylt	FBL
24.1734	NaN	NaN	24.8977	NaN	NaN	NaN	NaN	NaN	23.668	NaN	NaN	A6NHQ2	A6NHQ2	rRNA/tRNA 2-O-m	FBLL1
NaN	NaN	NaN	NaN	NaN	NaN	NaN	NaN	23.4214	NaN	NaN	NaN	P23142-4;B1AHL2	P23142-4;B1AHL2	Fibulin-1	FBLN1
NaN	NaN	NaN	NaN	NaN	NaN	24.2214	NaN	NaN	NaN	NaN	NaN	P35555	P35555	Fibrillin-1	FBN1
NaN	NaN	NaN	21.3137	NaN	NaN	NaN	NaN	NaN	20.6306	NaN	NaN	Q9NXK8;Q9NXK8-	Q9NXK8;Q9NXK8-	F-box/LRR-repeat	FBXL12
24.736	23.2358	23.7427	24.217	23.353	23.9346	23.2451	NaN	22.5059	23.7426	23.8101	NaN	Q96ME1-4;Q96M	Q96ME1-4;Q96M	F-box/LRR-repeat	FBXL18
NaN	NaN	20.929	20.4288	NaN	NaN	NaN	NaN	NaN	NaN	NaN	NaN	Q96IG2;J3KTA1;Q	Q96IG2;J3KTA1;Q	F-box/LRR-repeat	FBXL20
NaN	NaN	NaN	21.18	NaN	NaN	NaN	NaN	NaN	NaN	NaN	NaN	Q8N531;Q8N531-	Q8N531;Q8N531-	F-box/LRR-repeat	FBXL6
23.0816	NaN	22.6984	22.2364	NaN	22.0549	NaN	NaN	NaN	22.2814	NaN	NaN	O94952;H0YIE9;Q	O94952;H0YIE9;Q	F-box only proteir	FBXO21
24.8942	24.4092	24.49	24.5935	24.4567	24.3498	NaN	NaN	NaN	24.2294	24.5659	23.9727	Q8NEZ5;Q8NEZ5-	Q8NEZ5;Q8NEZ5-	F-box only proteir	FBXO22
22.5016	NaN	22.3474	22.8704	22.8884	22.3942	NaN	NaN	NaN	22.0138	22.1597	NaN	Q6PIJ6;Q6PIJ6-3;(	Q6PIJ6;Q6PIJ6-3;(	F-box only proteir	FBXO38
NaN	NaN	NaN	NaN	20.633	NaN	NaN	NaN	NaN	NaN	NaN	NaN	P0C2W1;C9JLC0	P0C2W1;C9JLC0	F-box/SPRY doma	FBXO45
22.0997	21.8541	22.0287	21.9357	21.9706	NaN	22.3084	NaN	NaN	22.118	NaN	NaN	Q9Y3I1;Q9Y3I1-3;	Q9Y3I1;Q9Y3I1-3;	F-box only proteir	FBXO7
22.7157	NaN	NaN	NaN	NaN	NaN	NaN	NaN	NaN	NaN	NaN	NaN	Q9UKB1;Q9UKB1-	Q9UKB1;Q9UKB1-	F-box/WD repeat-	FBXW11;BTRC
26.8616	27.0022	26.8146	NaN	27.1591	NaN	NaN	NaN	NaN	27.6002	27.4902	NaN	Q0JRZ9;Q0JRZ9-3;	Q0JRZ9;Q0JRZ9-3;	F-BAR domain onl	FCHO2
NaN	NaN	20.7862	NaN	NaN	NaN	NaN	NaN	NaN	NaN	NaN	NaN	Q86WN1;E5RGB1	Q86WN1;E5RGB1	F-BAR and double	FCHSD1
NaN	NaN	NaN	NaN	NaN	NaN	NaN	NaN	NaN	20.2209	NaN	NaN	P37268;E9PNM1;I	P37268;E9PNM1;I	Squalene synthas	FDFT1
28.0174	28.3032	28.3703	27.6139	27.8823	28.0947	28.5471	28.6682	27.9786	28.6034	28.3986	28.008	P14324;P14324-2	P14324;P14324-2	Farnesyl pyrophos	FDPS
24.9363	24.2755	24.4636	25.005	24.5387	24.3381	NaN	NaN	NaN	24.6544	24.7096	NaN	P22570;A0A0A0M	P22570;A0A0A0M	NADPH:adrenodo	FDXR
28.0777	27.8107	27.76	27.9659	28.0569	28.0511	27.1629	27.5968	27.6727	28.3416	28.2046	28.3755	P39748;P39748-2	P39748;P39748-2	Flap endonucleas	FEN1
22.8867	NaN	22.6695	22.5901	NaN	22.4876	NaN	NaN	NaN	NaN	22.6109	NaN	P16591;P16591-2	P16591;P16591-2	Tyrosine-protein k	FER
23.9485	23.5369	23.438	23.5466	23.3668	23.6667	24.1379	23.7081	24.9625	24.4927	24.5065	24.6315	Q9BQL6;G3V1L6;(	Q9BQL6;G3V1L6;(	Fermitin family hc	FERMT1
29.0079	29.429	29.7096	28.8816	29.4039	29.7515	29.5184	29.4075	29.6943	29.6497	29.4159	29.5951	Q96AC1;Q96AC1-	Q96AC1;Q96AC1-	Fermitin family hc	FERMT2
28.6907	27.8348	27.8822	28.505	27.8709	28.0781	28.6356	27.5378	28.0811	29.1231	28.6099	28.4676	Q86UX7;Q86UX7-	Q86UX7;Q86UX7-	Fermitin family hc	FERMT3
NaN	NaN	NaN	29.5506	NaN	NaN	30.1882	30.1624	29.7629	NaN	NaN	28.6499	Q8TAT2	Q8TAT2	Fibroblast growth	FGFBP3
27.5923	28.5718	27.7593	27.4071	28.4139	27.7042	27.8674	28.691	28.6056	27.8075	28.1631	28.4294	P07954;P07954-2	P07954;P07954-2	Fumarate hydrata	FH
NaN	NaN	NaN	21.6645	NaN	NaN	NaN	NaN	NaN	NaN	NaN	NaN	Q13642-1;Q13642	Q13642-1;Q13642	Four and a half LII	FHL1
25.0518	24.1023	24.6457	24.3134	24.4903	24.5945	24.6283	NaN	24.8443	25.0914	24.7539	25.3326	Q9Y613;H3BVE7;J	Q9Y613	FH1/FH2 domain-	FHOD1
25.4333	24.6281	25.1544	25.4113	24.734	24.9779	24.4565	NaN	24.5877	25.1522	25.1827	25.4092	O43427;O43427-2	O43427;O43427-2	Acidic fibroblast g	FIBP
23.8015	NaN	23.9513	24.3919	NaN	NaN	NaN	NaN	NaN	24.8259	NaN	NaN	Q92562;Q5TCS5;H	Q92562;Q5TCS5	Polyphosphoinosi	FIG4
23.1741	22.4245	22.335	23.0641	22.5276	NaN	NaN	NaN	NaN	22.5198	22.3213	NaN	Q6UN15-3;Q6UN1	Q6UN15-3;Q6UN1	Pre-mRNA 3-end-|	FIP1L1
23.3264	23.4308	23.5012	23.0019	NaN	NaN	23.4476	NaN	NaN	22.3927	NaN	NaN	Q9Y3D6	Q9Y3D6	Mitochondrial fiss	FIS1
24.009	24.6179	24.4357	24.1223	24.402	24.2924	25.4191	25.363	25.8033	25.0822	25.1694	24.5735	Q96AY3;H0Y827;k	Q96AY3	Peptidyl-prolyl cis	FKBP10
NaN	NaN	NaN	23.3105	NaN	NaN	NaN	NaN	NaN	NaN	NaN	NaN	Q9NYL4;F8VU90;(	Q9NYL4;F8VU90;(	Peptidyl-prolyl cis	FKBP11
NaN	NaN	NaN	NaN	21.2593	NaN	NaN	NaN	NaN	NaN	NaN	NaN	Q5T1M5;A0A0A0I	Q5T1M5;A0A0A0I	FK506-binding prc	FKBP15
28.5328	29.3875	28.9932	28.0172	28.2887	28.898	29.1654	28.5922	28.6768	29.1581	29.0832	28.9109	P62942;Q5W0X3;	P62942;Q5W0X3;	Peptidyl-prolyl cis	FKBP1A;FKBP12-E:
24.8724	25.4883	25.1281	24.3315	25.6139	25.2625	25.0694	26.9876	26.7077	24.8861	25.7077	25.8514	P26885;F5H0N4	P26885	Peptidyl-prolyl cis	FKBP2
27.2353	28.35	27.5156	27.0164	28.0571	27.5126	26.3111	25.1077	26.1202	26.0963	26.1966	26.4015	Q00688;G3V5F2	Q00688	Peptidyl-prolyl cis	FKBP3
28.5276	28.8324	28.311	28.2077	28.9336	28.2651	28.5397	28.9991	29.1448	28.2914	28.5823	29.4185	Q02790;H0YFG2;f	Q02790	Peptidyl-prolyl cis	FKBP4
26.383	25.9194	25.6108	26.0034	25.6849	25.4774	24.8205	NaN	NaN	25.6729	25.9373	25.9656	Q13451;Q13451-2	Q13451	Peptidyl-prolyl cis	FKBP5
23.5571	23.198	23.5262	23.454	22.9345	23.3339	23.573	24.1483	23.5615	22.6195	22.7744	NaN	Q14318;Q14318-2	Q14318;Q14318-2	Peptidyl-prolyl cis	FKBP8
NaN	NaN	20.9546	21.1505	NaN	NaN	NaN	NaN	NaN	NaN	NaN	NaN	Q8NFF5;Q8NFF5-:	Q8NFF5;Q8NFF5-:	FAD synthase;Mo	FLAD1
22.7544	NaN	22.1435	22.7092	22.1834	22.5429	NaN	NaN	NaN	NaN	NaN	NaN	Q8NFG4;Q8NFG4	Q8NFG4	Folliculin	FLCN
28.0237	27.1094	27.7584	28.1055	27.4286	27.7078	27.7593	27.9637	27.6083	28.1267	28.0461	27.7147	Q13045;Q13045-3	Q13045;Q13045-3	Protein flightless-	FLII
NaN	NaN	NaN	23.2274	NaN	NaN	NaN	NaN	NaN	NaN	NaN	NaN	Q13045-2	Q13045-2	Protein flightless-	FLII
32.5229	32.6557	32.8982	32.3713	32.4995	33.0423	32.9279	32.4495	31.7842	33.0196	32.6374	32.3989	P21333;P21333-2	P21333;P21333-2	Filamin-A	FLNA
26.8258	27.2771	27.6168	27.0711	27.649	28.0295	27.7856	27.7667	27.0869	27.6218	27.9022	28.0372	Q60FE5;A0A087W	Q60FE5;A0A087WWY3		FLNA
26.1149	NaN	25.2809	26.4686	25.6832	25.296	NaN	NaN	NaN	26.5314	26.0961	NaN	O75369;O75369-2	O75369;O75369-2	Filamin-B	FLNB
32.656	32.1429	32.1979	32.7676	32.0828	32.2469	33.4501	32.7358	32.3394	33.5251	33.0742	32.8797	O75369-8	O75369-8	Filamin-B	FLNB
30.6855	30.3223	30.3541	30.7058	30.1713	30.1813	31.2136	30.6806	29.7498	30.7548	30.2564	30.1938	Q14315;Q14315-2	Q14315;Q14315-2	Filamin-C	FLNC
28.4851	28.294	28.507	28.7847	28.5633	28.7808	27.9346	27.3648	28.5413	28.3187	28.1301	28.5523	O75955;O75955-2	O75955;O75955-2	Flotillin-1	FLOT1
28.0034	27.0938	27.4349	27.8093	27.0845	27.5019	26.6937	26.5676	27.3681	27.2361	26.6497	27.1599	Q14254;J3QLD9;E	Q14254;J3QLD9;E	Flotillin-2	FLOT2
22.6014	NaN	23.2194	22.7215	NaN	23.2532	23.5921	NaN	NaN	23.6947	23.6755	NaN	Q9Y5Y0;H7C3Z2;C	Q9Y5Y0;H7C3Z2;C	Feline leukemia vi	FLVCR1
NaN	22.3912	NaN	NaN	NaN	NaN	NaN	NaN	NaN	NaN	NaN	NaN	Q96CP2;I3L1Y9	Q96CP2;I3L1Y9	FLYWCH family m	FLYWCH2

29.2657	29.0509	29.668	28.9002	28.8972	29.7005	29.1119	28.1243	28.6811	29.469	29.2813	29.0569	O95466-2;O9546	O95466-2;O9546	Formin-like protei	FMNL1
26.8964	26.3984	27.0213	26.505	26.3615	27.0392	26.9173	25.8675	25.9631	27.9938	26.7212	26.3511	Q96PY5;Q96PY5-3	Q96PY5;Q96PY5-3	Formin-like protei	FMNL2
24.194	24.4676	25.8616	24.3765	24.8676	25.4437	25.1638	NaN	24.1288	25.8846	25.7548	25.5895	Q8IVF7;Q8IVF7-2;	Q8IVF7;Q8IVF7-2;	Formin-like protei	FMNL3
23.5316	23.8351	23.8514	23.2061	23.5268	23.8764	NaN	NaN	NaN	NaN	NaN	NaN	Q06787;Q06787-7	Q06787;Q06787-7	Fragile X mental r	FMR1
NaN	NaN	23.1523	NaN	NaN	NaN	NaN	NaN	NaN	NaN	NaN	NaN	R9WNI0	R9WNI0		FMR1
27.474	28.0078	28.43	25.7548	27.0677	27.7359	31.3108	30.6042	28.7218	28.4863	27.7351	27.3001	P02751;P02751-5	P02751;P02751-5	Fibronectin;Anast	FN1
25.6299	25.3791	24.6423	25.1822	25.0955	24.8167	24.3254	NaN	NaN	25.6197	25.6879	24.7299	Q9HA64;I3L2G3;I3	Q9HA64	Ketosamine-3-kin	FN3KRP
25.615	25.9383	25.747	25.1868	25.2356	25.1617	25.703	25.7898	25.5953	25.2678	25.6038	25.4766	Q96RU3;Q96RU3-	Q96RU3;Q96RU3-	Formin-binding pr	FNBP1
21.707	NaN	NaN	21.7073	NaN	NaN	NaN	NaN	NaN	NaN	NaN	NaN	Q9Y2H6;Q9Y2H6-	Q9Y2H6;Q9Y2H6-	Fibronectin type-I	FNDC3A
22.3865	22.6028	23.0415	22.243	21.8613	22.6978	NaN	NaN	NaN	NaN	NaN	NaN	Q53EP0;Q53EP0-2	Q53EP0;Q53EP0-2	Fibronectin type I	FNDC3B
NaN	NaN	NaN	NaN	NaN	NaN	NaN	NaN	22.6891	NaN	NaN	NaN	Q8TF40;A0A087W	Q8TF40;A0A087W	Folliculin-interacti	FNIP1
25.5137	24.4047	24.1171	24.6976	24.028	NaN	NaN	NaN	NaN	24.8442	23.9868	NaN	P49354;B3KVN2;E	P49354;B3KVN2;E	Protein farnesyltr	FNTA
NaN	23.2407	23.1565	21.4505	NaN	NaN	NaN	NaN	NaN	NaN	NaN	NaN	P15407;E9PPX2;P	P15407;E9PPX2;P	Fos-related antige	FOSL1
24.6742	24.5421	24.6473	25.1988	24.9125	24.774	NaN	NaN	NaN	24.6077	25.0054	NaN	P85037;P85037-2	P85037;P85037-2	Forkhead box pro	FOXK1
23.2138	23.1196	23.5419	23.7091	23.4837	23.4176	NaN	NaN	NaN	23.4451	23.5548	NaN	Q14331;Q9BZ01;E	Q14331;Q9BZ01;E	Protein FRG1;Prot	FRG1;FRG1B
21.012	NaN	NaN	NaN	NaN	NaN	NaN	NaN	NaN	NaN	NaN	NaN	Q9P2Q2;F8WAN4	Q9P2Q2;F8WAN4	FERM domain-cor	FRMD4A
26.8143	26.2944	27.049	26.7705	26.0122	27.0207	27.0948	25.6871	26.3048	27.4832	26.6921	26.4511	Q96NE9-2;G3V4T	Q96NE9-2;G3V4T	FERM domain-cor	FRMD6
23.0681	24.5056	24.8306	23.3807	24.7633	25.4167	24.4754	24.7295	25.0535	25.4839	24.9426	NaN	Q9BZ67;Q9BZ67-2	Q9BZ67;Q9BZ67-2	FERM domain-cor	FRMD8
27.0029	26.1282	27.0653	27.0389	26.4559	26.4086	23.7989	23.5541	25.5481	25.993	25.9811	25.7422	O94915;F2Z2S2;H	O94915;F2Z2S2	Protein furry hom	FRYL
28.5874	29.1425	28.9534	28.3869	29.0656	28.8377	29.8024	29.4073	29.6675	29.6826	29.7638	29.5974	Q16658;C9JFC0;A	Q16658	Fascin	FSCN1
23.6972	23.0692	24.0144	23.0103	NaN	NaN	28.2231	28.8821	25.1062	24.0754	NaN	NaN	P19883-2;P19883	P19883-2;P19883	Follistatin	FST
25.3619	25.6292	23.9098	24.2876	24.4855	24.0547	27.3468	NaN	23.7711	24.6951	24.199	NaN	Q12841;Q12841-2	Q12841;Q12841-2	Follistatin-related	FSTL1
24.7901	26.0178	25.6105	24.2754	25.3064	25.4279	26.8895	26.9178	NaN	26.2165	26.2431	26.4001	P02794;G3V192;G	P02794;G3V192;G	Ferritin heavy cha	FTH1
NaN	24.1614	NaN	NaN	23.2156	NaN	24.3162	25.0241	25.0098	NaN	25.2068	NaN	P02792;A0A087X1	P02792;A0A087X1	Ferritin light chair	FTL
22.3786	NaN	21.8197	NaN	21.8141	NaN	NaN	NaN	NaN	22.961	22.2595	NaN	Q9C0B1;Q9C0B1-	Q9C0B1	Alpha-ketoglutara	FTO
24.214	23.2894	23.5722	24.6656	NaN	23.8707	23.6748	NaN	NaN	NaN	NaN	23.5995	Q9UET6;Q9UET6-	Q9UET6;Q9UET6-	Putative tRNA (cy	FTSJ1
25.4602	23.9306	24.3735	26.0794	24.8044	24.9497	NaN	NaN	NaN	23.2458	23.6091	NaN	Q8IY81;J3KS74;J3	Q8IY81	pre-rRNA process	FTSJ3
27.901	28.2454	28.201	28.1879	28.347	28.4238	27.2814	27.9605	27.3154	28.3796	28.6665	28.8173	Q96AE4;E9PEB5	Q96AE4;E9PEB5	Far upstream eler	FUBP1
27.1776	26.5609	26.3809	26.913	26.5144	26.5325	25.2509	26.1446	25.6383	25.7969	26.2218	26.18	Q96I24;Q96I24-2	Q96I24	Far upstream eler	FUBP3
NaN	NaN	NaN	NaN	NaN	NaN	21.5229	NaN	NaN	NaN	NaN	NaN	P04066	P04066	Tissue alpha-L-fuc	FUCA1
23.6885	23.0265	22.3786	22.6464	22.4269	23.599	25.6587	23.9782	23.2389	24.1921	23.4054	NaN	Q9BTY2;Q9BTY2-2	Q9BTY2;Q9BTY2-2	Plasma alpha-L-fu	FUCA2
27.7364	26.9633	27.0005	27.2002	26.7303	27.022	26.2543	26.5858	25.6239	27.6269	27.1826	27.3416	P35637;H3BPE7;P	P35637;H3BPE7;P	RNA-binding prot	FUS
26.7643	26.7662	26.7357	26.9293	26.8528	26.9579	26.8492	26.8198	27.0645	26.5234	26.2015	26.5328	Q9BYC5;G3XAD2;	Q9BYC5;G3XAD2;	Alpha-(1,6)-fucosy	FUT8
28.6804	28.3937	28.0016	28.8081	28.2313	28.216	27.0116	27.4158	27.5361	27.3983	27.7496	27.3837	P51114;B4DXZ6;P	P51114;B4DXZ6;P	Fragile X mental r	FXR1
27.9664	26.9898	26.8388	27.6313	26.7769	26.5301	25.7288	25.4154	25.5567	25.6507	25.6183	24.9487	P51116;I3L1Z2;A0	P51116	Fragile X mental r	FXR2
25.7099	25.0314	24.3585	24.7599	25.0918	NaN		24.3079	25.9036	NaN	24.2305	25.3416	Q9BQS8;Q9BQS8-	Q9BQS8;Q9BQS8-	FYVE and coiled-c	FYCO1
23.7472	23.9059	24.45	24.1439	23.4639	24.254	NaN	NaN	NaN	24.2938	23.8899	NaN	P06241;P06241-2	P06241;P06241-2	Tyrosine-protein k	FYN
24.73	24.0897	25.158	24.8703	24.859	25.54	23.9153	NaN	24.3894	25.3277	24.6944	23.9427	Q14332	Q14332	Frizzled-2	FZD2
NaN	NaN	23.5042	NaN	NaN	24.2463	NaN	NaN	NaN	NaN	NaN	NaN	O60353;O60353-2	O60353;O60353-2	Frizzled-6	FZD6
28.7572	28.6266	28.4669	28.1563	28.2274	28.0859	28.4735	29.454	28.2787	28.2397	28.52	28.9304	Q13283;E5RIZ6;E5	Q13283	Ras GTPase-activa	G3BP1
26.8959	25.9245	26.3658	26.6585	25.9969	26.1683	25.6687	27.2048	NaN	26.1248	26.1712	26.8559	Q9UN86-2;Q9UN8	Q9UN86-2;Q9UN8	Ras GTPase-activa	G3BP2
NaN	NaN	NaN	NaN	NaN	NaN	NaN	NaN	NaN	NaN	NaN	NaN	Q9BUM1;K7ESE6	Q9BUM1;K7ESE6	Glucose-6-phosph	G6PC3
28.9026	29.7341	29.9679	28.7346	29.51	29.9889	28.9174	28.6862	28.7177	28.8564	29.0171	28.9097	P11413;P11413-2	P11413;P11413-2	Glucose-6-phosph	G6PD
24.7605	25.221	24.4654	24.3975	24.6991	24.2395	26.2843	26.0645	25.7358	25.2094	25.1761	24.9032	P10253;I3L0S5;I3	P10253	Lysosomal alpha-g	GAA
27.3417	26.1901	26.8635	25.7535	24.602	25.3827	27.2722	NaN	25.5878	24.8091	24.4932	NaN	O95166;H6UMI1;	O95166;H6UMI1;	Gamma-aminobu	GABARAP
23.7459	23.9913	24.6566	NaN	24.4823	NaN	24.571	NaN	NaN	NaN	NaN	NaN	Q9H0R8;F5GZY7;C	Q9H0R8;F5GZY7;C	Gamma-aminobu	GABARAPL1
26.9959	26.403	27.2597	25.7533	25.0023	26.3837	28.5184	27.1446	27.2614	27.171	25.9904	26.465	P60520;H3BSM5;I	P60520;H3BSM5	Gamma-aminobu	GABARAPL2
NaN	NaN	NaN	20.1591	NaN	NaN	NaN	NaN	NaN	NaN	NaN	NaN	Q8TAE8	Q8TAE8	Growth arrest and	GADD45GIP1
23.6436	22.6858	23.1623	23.4495	NaN	22.9689	23.0809	NaN	NaN	22.922	23.0507	NaN	O14976;O14976-2	O14976;O14976-2	Cyclin-G-associate	GAK
NaN	NaN	NaN	NaN	NaN	NaN	25.1251	NaN	NaN	NaN	NaN	NaN	P22466	P22466	Galanin peptides;	GAL
24.5705	NaN	23.9084	23.7232	NaN	NaN	NaN	NaN	NaN	NaN	NaN	NaN	P54803;A0A087W	P54803;A0A087W	Galactocerebrosid	GALC
27.5445	26.7392	27.1633	27.5027	26.7075	27.1179	26.3899	26.3419	26.7415	26.9446	26.7491	26.7756	Q14376;Q14376-2	Q14376;Q14376-2	UDP-glucose 4-ep	GALE
27.7196	27.2033	27.4054	27.4687	27.5325	27.5057	26.5148	26.2918	26.7585	27.1283	27.3821	26.9207	P51570;P51570-2	P51570;P51570-2	Galactokinase	GALK1
22.1331	22.8908	22.4417	21.5444	22.8623	22.4703	23.2807	21.6721	23.1464	23.0556	22.6987	23.2648	Q96C23;H7C320;E	Q96C23;H7C320;E	Aldose 1-epimera	GALM
25.0544	24.8922	25.4234	25.16	25.265	25.4351	25.1345	24.8778	25.3305	24.9512	24.6365	24.8201	Q10471;Q10471-2	Q10471	Polypeptide N-ace	GALNT2
22.3982	22.6378	23.015	22.8963	23.091	NaN	24.9078	NaN	NaN	22.8216	NaN	NaN	Q8NCL4;S4R3S5	Q8NCL4	Polypeptide N-ace	GALNT6
23.5874	23.8646	24.3504	23.7001	24.2013	24.0541	24.9439	NaN	NaN	24.0773	24.2037	NaN	Q86SF2;H0YAH3;I	Q86SF2;H0YAH3	N-acetylgalactosa	GALNT7
NaN	NaN	NaN	NaN	NaN	NaN	NaN	NaN	NaN	22.5372	NaN	NaN	P07902;G3V3U5;C	P07902;G3V3U5;C	Galactose-1-phos	GALT
23.5665	23.1707	23.1308	23.2646	23.5008	NaN	NaN	NaN	NaN	24.3829	24.3255	24.5644	Q14353;Q14353-2	Q14353;Q14353-2	Guanidinoacetate	GAMT
NaN	22.7438	NaN	NaN	NaN	NaN	NaN	NaN	22.4465	NaN	NaN	22.7433	Q9H2C0	Q9H2C0	Gigaxonin	GAN

30.4094	30.8744	30.6868	30.4955	30.9388	30.6746	31.1089	31.7316	31.7387	30.8201	30.8961	30.9543	Q14697;F5H6X6	Q14697;F5H6X6	Neutral alpha-gluc	GANAB
34.1457	34.806	34.4355	34.109	34.5219	34.454	34.7752	34.4732	34.3758	35.076	35.4903	34.4217	P04406;P04406-2	P04406;P04406-2	Glyceraldehyde-3	GAPDH
24.9487	24.9011	24.7821	24.6862	24.9739	25.0449	24.5813	24.738	NaN	24.9135	24.6057	25.0413	Q14C86;Q14C86-(	Q14C86;Q14C86-(	GTPase-activating	GAPVD1
25.0587	23.6992	24.9434	26.0424	24.3863	25.1422	23.3623	NaN	23.5904	24.2133	23.9546	23.7035	Q9NY12;Q9NY12-	Q9NY12;Q9NY12-	H/ACA ribonucleo	GAR1
29.5354	29.5901	29.4886	29.3293	29.3992	29.3658	29.8534	29.7728	29.7259	29.7768	29.639	29.7046	P41250;H7C443	P41250	Glycine--tRNA liga	GARS
30.1536	29.8474	29.8172	30.1218	29.8221	29.8573	29.8218	29.6909	29.697	29.75	29.7622	29.814	P22102;P22102-2	P22102;P22102-2	Trifunctional purir	GART
NaN	NaN	NaN	NaN	NaN	NaN	NaN	NaN	NaN	21.1517	NaN	NaN	Q99501;A0A5E8;(	Q99501;A0A5E8;(	GAS2-like protein	GAS2L1
NaN	NaN	NaN	NaN	NaN	NaN	NaN	NaN	NaN	NaN	20.0006	NaN	Q86XJ1;G3V1N3	Q86XJ1;G3V1N3	GAS2-like protein	GAS2L3
26.0222	25.4605	25.2845	25.7433	25.9012	25.5491	NaN	NaN	NaN	24.6798	25.2163	NaN	Q86YP4;Q86YP4-2	Q86YP4;Q86YP4-2	Transcriptional re	GATAD2A
24.0073	NaN	24.6862	24.2242	NaN	NaN	NaN	NaN	NaN	NaN	NaN	NaN	Q8WXI9;A0A0U1F	Q8WXI9;A0A0U1F	Transcriptional re	GATAD2B
26.5643	25.9188	25.3597	25.2666	25.7037	25.4535	26.7853	26.4899	26.3168	26.3569	26.3917	24.8525	P04062;A0A0G2JI	P04062;A0A0G2JI	Glucosylceramida	GBA
24.5049	24.314	24.4629	24.7648	24.8593	24.9226	NaN	NaN	24.9063	23.7423	24.773	24.3446	O75323;C9K068;H	O75323;C9K068;H	Protein NipSnap h	GBAS
28.9472	29.2793	29.3216	28.7913	29.1935	29.2877	29.7843	29.5282	29.4448	29.6114	29.4766	29.7146	Q04446;E9PGM4	Q04446;E9PGM4	1,4-alpha-glucan-l	GBE1
27.6112	26.528	26.7774	27.5209	26.2695	26.6498	26.9634	26.0375	26.0093	26.2435	26.1408	25.022	Q92538;Q92538-2	Q92538;Q92538-2	Golgi-specific bref	GBF1
25.6519	26.128	25.9211	25.1343	25.6581	26.3572	26.1763	NaN	26.2137	25.8137	25.6032	26.1922	P32455;Q9H0R5;F	P32455	Interferon-induce	GBP1
23.5891	NaN	23.3773	23.1732	NaN	23.3835	22.6223	NaN	NaN	23.3569	23.0027	NaN	P28676;H7C2Z6;H	P28676;H7C2Z6;H	Grancalcin	GCA
24.6622	23.4457	23.479	24.7796	24.4995	23.5723	NaN	NaN	NaN	23.2045	NaN	NaN	Q92947;Q92947-2	Q92947;Q92947-2	Glutaryl-CoA dehy	GCDH
22.6681	NaN	22.0077	22.9497	21.6118	21.8037	NaN	NaN	NaN	21.8293	21.6731	NaN	P16383;P16383-2	P16383;P16383-2	GC-rich sequence	GCFC2
24.5622	25.2209	24.8521	24.3721	25.1665	24.7252	24.696	NaN	24.3294	24.7162	24.8109	24.8804	P48506;E1CEI4;A(	P48506;E1CEI4;A(	Glutamate--cystei	GCLC
26.3254	25.9616	25.8951	25.952	26.126	25.8074	25.6972	25.485	26.0726	26.2345	26.0617	26.551	P48507;P48507-2	P48507;P48507-2	Glutamate--cystei	GCLM
30.8685	29.5535	30.2234	30.8976	29.9704	30.4279	29.6127	30.049	30.0811	30.4933	30.3442	29.9786	Q92616	Q92616	Translational activ	GCN1L1
NaN	NaN	NaN	NaN	NaN	NaN	22.6998	NaN	NaN	NaN	NaN	NaN	Q8N0V5	Q8N0V5	N-acetyllactosami	GCNT2
NaN	23.762	22.6015	21.711	23.1826	22.8103	NaN	NaN	24.0189	22.1378	NaN	22.984	P23434	P23434	Glycine cleavage s	GCSH
23.7118	23.8074	24.0029	23.8453	24.0581	NaN	NaN	24.1118	NaN	23.5723	23.6671	23.7032	Q99988;A0A0A0N	Q99988	Growth/differenti	GDF15
27.1032	28.0248	27.7828	26.3708	27.9576	27.8537	27.1717	27.194	27.866	27.1022	27.1264	27.5429	P31150;G5E9U5	P31150	Rab GDP dissociat	GDI1
30.4722	31.429	31.1974	30.353	31.2548	31.1706	31.3496	31.1972	31.5077	31.3329	31.2235	31.8928	P50395;P50395-2	P50395;P50395-2	Rab GDP dissociat	GDI2
23.7218	23.8306	23.6784	24.3199	23.8846	23.969	NaN	NaN	NaN	NaN	24.1146	NaN	O14893;O14893-2	O14893;O14893-2	Gem-associated p	GEMIN2
28.2046	26.3453	27.159	28.296	26.7216	27.2838	25.7995	25.9602	26.0898	27.4721	27.073	26.0954	P57678;I3L2C7;I3I	P57678;I3L2C7	Gem-associated p	GEMIN4
28.3383	26.6759	27.2447	28.0842	26.9548	27.2734	26.6786	26.6741	27.0683	27.5726	27.3527	27.3583	Q8TEQ6	Q8TEQ6	Gem-associated p	GEMIN5
24.821	24.5527	24.1071	24.5442	24.0623	24.1793	NaN	NaN	NaN	23.8881	NaN	NaN	Q8WXD5;B9A037;	Q8WXD5	Gem-associated p	GEMIN6
23.1208	NaN	NaN	22.9876	NaN	NaN	NaN	NaN	NaN	NaN	NaN	NaN	Q9NWZ8;H7BYE2	Q9NWZ8;H7BYE2	Gem-associated p	GEMIN8
25.1792	24.1217	24.8604	24.9846	24.4482	24.8597	24.2055	NaN	24.9985	25.3701	25.4704	24.909	Q7L5D6;Q7L5D6-2	Q7L5D6;Q7L5D6-2	Golgi to ER traffic	GET4
26.0364	25.3438	24.856	25.2332	25.0161	24.8881	24.4827	NaN	24.474	25.0316	25.6438	27.2646	Q96RP9;Q96RP9-2	Q96RP9;Q96RP9-2	Elongation factor	GFM1
NaN	NaN	NaN	NaN	NaN	NaN	NaN	NaN	27.9688	NaN	23.9445	27.4748	Q969S9;Q969S9-5	Q969S9;Q969S9-5	Ribosome-releasir	GFM2
30.3321	29.601	29.7877	30.2312	29.7872	29.774	29.3897	29.5124	29.4325	29.6137	29.8012	29.4118	Q06210;Q06210-2	Q06210;Q06210-2	Glutamine--fructo	GFPT1
28.1802	27.734	27.9078	28.1577	27.9074	28.0666	28.3772	27.8713	28.5086	28.1504	28.1366	28.2676	O94808;E5RJP4	O94808	Glutamine--fructo	GFPT2
22.7362	23.681	23.8008	22.8737	23.8463	23.2748	NaN	NaN	NaN	23.2272	23.1616	NaN	Q9UJY5;Q9UJY5-2	Q9UJY5;Q9UJY5-2	ADP-ribosylation f	GGA1
NaN	NaN	NaN	NaN	NaN	NaN	NaN	NaN	NaN	20.7812	NaN	NaN	Q9UJY4	Q9UJY4	ADP-ribosylation f	GGA2
24.2056	24.1223	23.821	24.0777	23.6758	23.8277	23.273	NaN	23.9873	23.9175	23.3441	23.587	O75223;M0QZK8;	O75223;M0QZK8;	Gamma-glutamylc	GGCT
22.398	24.5218	22.8304	21.8222	24.0764	23.0796	26.7972	26.6509	24.18	23.2643	NaN	24.7689	Q92820	Q92820	Gamma-glutamyl	GGH
NaN	NaN	NaN	NaN	NaN	NaN	NaN	NaN	NaN	NaN	NaN	21.6346	O95749;C9J6G3;C	O95749;C9J6G3;C	Geranylgeranyl py	GGPS1
25.5156	26.0535	25.8589	25.6472	25.8738	25.8207	NaN	NaN	24.564	NaN	24.7824	25.0511	P19440;E7ET76;P	P19440;E7ET76;P	Gamma-glutamylt	GGT1
26.214	26.9388	27.636	26.947	27.0375	28.0209	26.1145	26.0386	26.6903	26.9095	26.8046	26.607	P36269;P36269-2	P36269;P36269-2	Gamma-glutamylt	GGT5
23.3204	22.6069	23.0224	23.4907	22.8297	22.8682	23.5043	NaN	NaN	23.5503	23.3327	NaN	Q8N2G8;Q8N2G8	Q8N2G8;Q8N2G8	GH3 domain-cont	GHDC
24.2875	24.5281	24.398	24.5987	24.5579	24.4849	24.0831	NaN	NaN	24.5431	24.2716	24.4136	Q9NWU2	Q9NWU2	Glucose-induced c	GID8
NaN	NaN	22.4855	22.18	NaN	NaN	NaN	NaN	NaN	NaN	NaN	NaN	Q6Y7W6;Q6Y7W6	Q6Y7W6;Q6Y7W6	PERQ amino acid-	GIGYF2
NaN	NaN	NaN	NaN	NaN	NaN	NaN	NaN	NaN	NaN	NaN	20.4498	Q14691	Q14691	DNA replication co	GINS1
NaN	NaN	NaN	NaN	NaN	NaN	NaN	NaN	NaN	NaN	NaN	22.1287	Q9BRX5;Q9BRX5-	Q9BRX5;Q9BRX5-	DNA replication co	GINS3
23.3744	24.6954	23.9718	22.8573	23.7805	NaN	NaN	NaN	NaN	24.5356	24.616	24.5412	Q9BRT9;E5RFF9	Q9BRT9;E5RFF9	DNA replication co	GINS4
27.6952	27.9949	27.8886	27.8631	27.8942	27.8848	27.9333	27.4477	28.0731	28.7593	28.2434	28.0757	O14908;O14908-2	O14908;O14908-2	PDZ domain-conta	GIPC1
23.5904	24.1792	23.8759	23.0429	24.1419	NaN	23.6084	NaN	23.7753	23.6841	NaN	23.3657	Q9Y2X7;Q9Y2X7-3	Q9Y2X7;Q9Y2X7-3	ARF GTPase-activa	GIT1
21.619	NaN	NaN	NaN	NaN	NaN	NaN	NaN	NaN	NaN	NaN	NaN	Q14161;R4GNG3;	Q14161;R4GNG3;	ARF GTPase-activa	GIT2
22.5328	NaN	22.2208	NaN	NaN	NaN	24.6138	22.7135	NaN	23.564	23.7082	NaN	P06280;V9GYN5	P06280	Alpha-galactosida	GLA
24.4823	23.9277	23.8964	23.967	23.7328	23.4423	25.1793	24.9126	25.1157	25.5194	25.3418	24.6682	P16278;E7EQ29;P	P16278;E7EQ29;P	Beta-galactosidas	GLB1
26.554	26.1536	26.0917	25.9258	25.866	26.5057	28.4965	26.7074	25.8634	27.1365	25.7584	27.082	Q92896;Q92896-2	Q92896;Q92896-2	Golgi apparatus p	GLG1
NaN	NaN	NaN	NaN	NaN	NaN	NaN	NaN	30.1739	NaN	NaN	NaN	P10070;P10070-1	P10070;P10070-1	Zinc finger protei	GLI2
24.73	25.482	25.6385	25.8982	25.4279	25.7644	25.4159	25.2246	26.3589	26.0447	25.3774	26.1744	P48060;B4E3S5;F	P48060	Glioma pathogen	GLIPR1
25.4788	26.5557	26.7102	26.0213	26.3347	26.5151	26.4226	25.7999	26.4321	26.7351	26.2628	26.6827	Q9H4G4;Q5VZR0;	Q9H4G4;Q5VZR0	Golgi-associated p	GLIPR2
26.3948	25.2197	25.3763	26.0781	25.3151	25.4672	24.9426	25.4264	25.1933	25.5559	25.4998	25.4393	Q92990;Q92990-2	Q92990;Q92990-2	Glomulin	GLMN
28.781	29.7069	29.1836	28.5237	29.2859	28.9964	29.2139	28.9721	28.7888	28.9925	28.9359	29.013	Q04760;Q04760-2	Q04760;Q04760-2	Lactoylglutathion	GLO1

27.0813	27.2829	27.1518	26.6795	26.7728	26.8939	27.0413	26.6271	26.8431	26.714	27.016	27.9008	Q9HC38-2;Q9HC3	Q9HC38-2;Q9HC3	Glyoxalase domai	GLOD4
26.3088	24.5988	NaN	25.2142	24.9689	NaN	25.6258	NaN	NaN	25.3569	24.4054	NaN	P35754	P35754	Glutaredoxin-1	GLRX
26.4633	26.2135	26.5059	26.527	25.769	26.2002	26.8463	NaN	25.0099	26.8382	25.8329	25.6037	O76003	O76003	Glutaredoxin-3	GLRX3
22.645	NaN	22.457	22.4268	NaN	NaN	NaN	NaN	NaN	NaN	NaN	NaN	Q86SX6	Q86SX6	Glutaredoxin-rela	GLRX5
27.3754	26.5067	26.1811	26.8962	26.2575	26.0732	24.7346	25.1294	25.9833	25.6491	25.4984	24.6945	O94925-3;O94925	O94925-3;O94925	Glutaminase kidn	GLS
22.4731	21.6735	21.8749	22.0416	21.7846	NaN	NaN	NaN	NaN	23.1589	22.6844	NaN	Q68CQ7;C9J880;C	Q68CQ7;C9J880;C	Glycosyltransfera	GLT8D1
28.0734	29.0235	28.3773	28.4022	29.0738	28.4057	27.9918	29.3809	29.6554	27.3405	27.93	28.4819	P00367;P00367-3,	P00367;P00367-3,	Glutamate dehyd	GLUD1;GLUD2
22.4821	NaN	NaN	22.6129	NaN	NaN	NaN	NaN	NaN	NaN	NaN	NaN	Q49A26;K7EMM8	Q49A26;K7EMM8	Putative oxidored	GLYR1
NaN	NaN	NaN	NaN	NaN	NaN	24.3964	NaN	NaN	NaN	NaN	NaN	P17900	P17900	Ganglioside GM2	GM2A
27.327	26.2689	26.5319	27.0192	26.2563	26.4473	26.7079	25.8862	26.0448	27.7314	27.6313	26.7283	O60547;O60547-2	O60547;O60547-2	GDP-mannose 4,6	GMDS
27.4305	27.6695	27.8558	27.3079	27.3467	27.7676	28.3158	27.2082	27.1611	28.2577	27.6617	27.8381	P60983;G3V4P8	P60983;G3V4P8	Glia maturation fa	GMFB
24.8915	25.2007	24.8897	24.1348	24.9374	25.0626	24.9956	24.7498	24.5872	25.0572	24.7406	25.1519	O60234;M0R0C1;	O60234;M0R0C1;	Glia maturation fa	GMFG
27.6852	26.5862	26.6387	26.9994	26.7866	26.447	26.5902	26.3457	26.358	27.3198	27.4492	26.5574	Q96IJ6;Q96IJ6-2;A	Q96IJ6;Q96IJ6-2;A	Mannose-1-phosp	GMPPA
26.3809	25.079	25.2152	25.2909	25.1491	24.8472	25.67	25.813	25.9458	26.3993	26.6883	26.4739	Q9Y5P6;Q9Y5P6-2	Q9Y5P6;Q9Y5P6-2	Mannose-1-phosp	GMPPB
25.946	26.2375	25.6963	24.9944	25.9059	25.329	25.9975	24.6818	24.9632	25.7987	25.5554	25.237	Q9P2T1;H0YMB3;	Q9P2T1;H0YMB3;	GMP reductase 2;	GMPR2
28.2688	27.7948	27.5664	27.7626	28.0404	27.9126	27.2744	27.7806	28.0387	28.5643	28.688	28.5368	P49915;P49915-2	P49915;P49915-2	GMP synthase [gl	GMPS
29.5254	29.2169	30.0794	29.664	29.3613	30.1482	29.7563	29.0349	29.4329	30.5963	30.0797	29.7601	P29992;K7EL62;A(	P29992	Guanine nucleotic	GNA11
22.8357	NaN	23.2985	22.6742	23.209	23.6516	NaN	NaN	NaN	23.9065	23.3049	NaN	Q03113;Q03113-2	Q03113;Q03113-2	Guanine nucleotic	GNA12
28.985	28.9881	29.4189	28.9557	29.099	29.7866	28.5282	27.106	28.1267	29.261	28.8011	28.6602	Q14344;Q14344-2	Q14344;Q14344-2	Guanine nucleotic	GNA13
24.9355	25.6395	25.8521	25.3026	25.6414	NaN	24.9057	NaN	NaN	NaN	NaN	24.8099	O95837	O95837	Guanine nucleotic	GNA14
25.59	25.3918	26.4062	25.4875	25.4864	26.574	25.4389	25.3516	26.0365	26.1002	26.1444	25.8704	P30679	P30679	Guanine nucleotic	GNA15
26.0359	25.293	25.7752	25.7316	24.9816	25.6776	25.6214	NaN	NaN	26.1368	25.0789	25.6963	P63096;P63096-2	P63096;P63096-2	Guanine nucleotic	GNAI1
31.8801	31.683	32.0691	31.7571	31.548	32.3106	31.7931	31.3159	31.7133	32.0961	31.7489	31.9169	P04899;P04899-4,	P04899;P04899-4,	Guanine nucleotic	GNAI2
29.9916	29.7761	30.4634	29.8339	29.5115	30.4794	30.0229	29.4882	30.0457	30.3935	30.012	30.372	P08754;P19087	P08754	Guanine nucleotic	GNAI3
27.047	26.3923	26.9103	26.9935	26.267	27.1765	26.1108	23.7843	25.6547	26.8932	26.1777	25.9939	P50148;B1AM21	P50148	Guanine nucleotic	GNAQ
25.9819	NaN	26.4231	26.161	26.5235	27.022	26.7115	NaN	26.3339	27.4436	26.7872	26.4327	P63092;P63092-4,	P63092;P63092-4	Guanine nucleotic	GNAS
23.6327	NaN	24.772	NaN	23.5563	NaN	NaN	NaN	NaN	NaN	NaN	NaN	P63092-2	P63092-2	Guanine nucleotic	GNAS
29.9523	29.8043	30.4926	30.2472	29.5847	30.6096	29.9688	29.3949	29.5504	30.6964	30.4335	30.158	P63092-3;H0Y7F4	P63092-3	Guanine nucleotic	GNAS
31.5976	32.2172	31.9204	31.8588	31.7707	31.8953	31.9343	31.7814	31.6296	32.1372	31.8849	31.7911	P62873;P62873-2,	P62873;P62873-2,	Guanine nucleotic	GNB1
NaN	NaN	NaN	22.3785	NaN	NaN	NaN	NaN	NaN	23.2363	NaN	NaN	Q9BYB4;Q9BYB4-:	Q9BYB4;Q9BYB4-:	Guanine nucleotic	GNB1L
29.1604	29.3916	28.8102	29.2883	28.7222	29.1003	28.9867	28.3996	28.2491	29.2878	28.9589	28.9794	P62879;C9JXA5;C!	P62879;C9JXA5;C!	Guanine nucleotic	GNB2
31.7893	30.4724	30.4013	31.2115	30.4082	30.3168	30.0173	30.507	29.8728	31.1334	31.2519	30.2608	P63244;J3KPE3;D(	P63244;J3KPE3;D(	Guanine nucleotic	GNB2L1
27.6361	29.0464	28.4934	28.2664	28.9114	28.8174	27.0151	27.6149	27.2578	27.1041	28.0053	28.1514	Q9HAV0;C9JD14;I	Q9HAV0	Guanine nucleotic	GNB4
27.8565	26.509	26.7178	27.3247	26.6428	26.6784	26.7773	25.6145	26.0927	27.272	26.6291	26.5969	Q9Y223;Q9Y223-2	Q9Y223;Q9Y223-2	Bifunctional UDP-	GNE
24.818	25.3025	25.4398	24.4043	24.2978	24.6331	23.8842	NaN	24.6958	24.2694	NaN	24.3954	P50151;Q9H1X3-3	P50151	Guanine nucleotic	GNG10
31.4237	30.7418	31.0779	31.2283	30.7697	31.218	30.997	29.8729	30.2652	31.2808	30.5196	30.3645	Q9UBI6	Q9UBI6	Guanine nucleotic	GNG12
29.3652	29.0672	29.6994	29.0173	28.7439	29.6694	28.7762	27.5904	28.6648	29.2055	28.5276	28.6602	P63218	P63218	Guanine nucleotic	GNG5
25.2995	24.5253	24.2799	24.2537	24.4105	24.3301	23.9332	NaN	24.5404	23.9977	23.9781	23.6935	P36915;P36915-2	P36915;P36915-2	Guanine nucleotic	GNL1
24.7027	23.6396	24.4074	25.7532	24.1469	24.6293	NaN	NaN	NaN	23.8475	23.9722	22.0728	Q13823;H0YG10	Q13823	Nucleolar GTP-bin	GNL2
25.6874	24.8089	25.3424	26.7186	25.569	25.5936	NaN	NaN	NaN	25.0506	25.4037	23.9841	Q9BVP2;Q9BVP2-	Q9BVP2;Q9BVP2-	Guanine nucleotic	GNL3
23.5	NaN	23.0028	23.9454	23.1975	NaN	NaN	NaN	NaN	22.4361	23.0642	NaN	Q9NVN8	Q9NVN8	Guanine nucleotic	GNL3L
26.8848	27.7019	27.2445	26.6602	27.3252	27.0216	27.5929	27.149	26.519	27.2123	27.0685	27.0708	P46926;D6RAY7;D	P46926;D6RAY7;D	Glucosamine-6-ph	GNPDA1
25.3122	25.553	25.6196	24.9066	25.3016	25.0586	25.0481	24.9148	25.4969	24.8881	24.8034	25.7294	Q96EK6;G3V4W4;	Q96EK6;G3V4W4;	Glucosamine 6-ph	GNPNAT1
24.1521	24.8388	24.4079	24.1569	24.541	24.5533	26.0288	26.2633	25.4124	23.9629	23.6802	24.1717	P15586;H7C3P4;F	P15586;H7C3P4;F	N-acetylglucosam	GNS
24.6836	23.6701	24.3155	24.8929	23.9615	24.0912	23.3229	23.9179	NaN	23.422	NaN	NaN	Q08379;A0A0C4D	Q08379;A0A0C4D	Golgin subfamily /	GOLGA2
25.0131	25.4908	24.6235	24.318	25.1036	24.3604	24.5659	NaN	NaN	24.5543	24.8769	NaN	Q08378;Q08378-2	Q08378;Q08378-2	Golgin subfamily /	GOLGA3
24.4422	24.5097	23.6409	24.2281	NaN	NaN	NaN	NaN	NaN	NaN	NaN	NaN	Q13439;Q13439-5	Q13439;Q13439-5	Golgin subfamily /	GOLGA4
21.42	NaN	NaN	NaN	NaN	NaN	NaN	NaN	NaN	NaN	NaN	NaN	Q8TBA6;Q8TBA6-	Q8TBA6;Q8TBA6-	Golgin subfamily /	GOLGA5
27.4437	27.371	27.8109	27.045	27.0114	27.2857	27.7054	26.882	27.927	27.6834	26.8957	27.649	Q7Z5G4;Q7Z5G4-	Q7Z5G4;Q7Z5G4-	Golgin subfamily /	GOLGA7
NaN	NaN	NaN	NaN	23.7768	NaN	NaN	NaN	NaN	NaN	NaN	NaN	Q2TAP0;M0R0R5	Q2TAP0;M0R0R5	Golgin subfamily /	GOLGA7B
24.12	24.1971	24.3693	23.5416	23.3076	24.8149	NaN	NaN	NaN	23.1159	NaN	NaN	Q14789;Q14789-2	Q14789;Q14789-2	Golgin subfamily I	GOLGB1
28.1161	27.8327	28.1969	28.0989	27.4835	27.9855	28.965	29.6789	30.627	27.8728	NaN	25.8209	O00461;F8W785	O00461;F8W785	Golgi integral mer	GOLIM4
24.4759	24.1845	24.3769	24.0671	24.5123	NaN	26.5136	25.9515	25.4109	24.8885	24.3975	25.0204	Q8NBJ4;Q8NBJ4-2	Q8NBJ4;Q8NBJ4-2	Golgi membrane p	GOLM1
23.1168	23.5467	23.7996	23.0275	23.0569	23.1331	24.3669	23.0118	NaN	23.3538	23.0917	22.3332	Q9H4A6	Q9H4A6	Golgi phosphopro	GOLPH3
25.1674	24.0989	24.7654	25.378	24.2805	24.504	NaN	NaN	NaN	24.7816	23.8186	NaN	Q9Y3E0;G3V1U5;I	Q9Y3E0;G3V1U5;I	Vesicle transport	GOLT1B
24.9474	24.3044	24.5701	24.9114	24.2021	24.7323	23.85	NaN	NaN	24.314	24.2726	NaN	Q9HD26;Q9HD26	Q9HD26;Q9HD26	Golgi-associated P	GOPC
NaN	NaN	NaN	22.9521	NaN	NaN	NaN	NaN	NaN	NaN	NaN	NaN	Q9BQQ3;H7C0J2;!	Q9BQQ3;H7C0J2;!	Golgi reassembly-	GORASP1
26.2664	26.0588	25.7668	25.9363	25.2313	25.1388	25.523	NaN	24.0769	25.5803	24.5022	24.5567	Q9H8Y8;Q9H8Y8-	Q9H8Y8;Q9H8Y8-	Golgi reassembly-	GORASP2
24.8737	25.3932	24.5973	24.4953	25.006	24.6554	25.3943	NaN	NaN	24.368	24.601	NaN	E9PCW1;O95249;	E9PCW1;O95249;	Golgi SNAP recept	GOSR1
28.6271	29.7379	29.075	28.2071	29.4002	29.0629	29.2662	29.2748	29.2239	28.9228	28.9536	29.3718	P17174;P17174-2	P17174;P17174-2	Aspartate aminot	GOT1

27.7933	29.4242	28.558	27.8997	29.254	28.4505	28.6632	29.8057	30.3029	28.7035	29.3802	29.6064	P00505;P00505-2	P00505;P00505-2	Aspartate aminot	GOT2
22.5145	NaN	22.5403	22.8988	NaN	NaN	NaN	NaN	NaN	22.5592	22.4722	NaN	O43292;O43292-2	O43292;O43292-2	Glycosylphosphat	GPAA1
NaN	NaN	NaN	NaN	NaN	NaN	NaN	NaN	NaN	NaN	20.0542	NaN	Q8N954;A0A0A0N	Q8N954;A0A0A0N	G patch domain-c	GPATCH11
23.0137	23.2474	24.7813	22.4231	23.2775	22.6095	28.8965	27.3458	25.5447	25.6699	26.0685	24.3694	P35052;H7C410;H	P35052;H7C410;H	Glypican-1;Secret	GPC1
NaN	NaN	22.2943	NaN	NaN	NaN	23.7535	NaN	NaN	NaN	NaN	NaN	Q9Y625;A0A087W	Q9Y625	Glypican-6;Secret	GPC6
NaN	NaN	21.9938	22.2469	NaN	NaN	NaN	NaN	NaN	22.7913	22.3632	23.2404	Q8N335;C9JFA7;C	Q8N335;C9JFA7;C	Glycerol-3-phosp	GPD1L
23.4574	24.2821	24.2568	23.9881	24.272	24.2299	23.5486	24.456	25.3886	NaN	23.3964	NaN	P43304;P43304-2	P43304;P43304-2	Glycerol-3-phosp	GPD2
25.2541	25.5177	25.2622	25.2165	25.37	24.9851	25.5799	25.6637	25.3841	24.74	25.4503	25.7605	Q9NQX3;Q9NQX3	Q9NQX3;Q9NQX3	Gephyrin;Molybd	GPHN
30.939	31.3853	31.267	30.674	31.259	31.2453	31.5385	31.4837	31.5644	31.2982	31.3971	31.6647	P06744;A0A0A0M	P06744;A0A0A0M	Glucose-6-phosph	GPI
NaN	NaN	NaN	NaN	22.8208	NaN	NaN	NaN	NaN	NaN	NaN	23.734	Q92917	Q92917	G patch domain a	GPKOW
NaN	NaN	22.9843	NaN	NaN	NaN	NaN	NaN	NaN	NaN	NaN	NaN	Q9HCN4;Q9HCN4	Q9HCN4;Q9HCN4	GPN-loop GTPase	GPN1
23.3276	NaN	22.948	NaN	NaN	NaN	NaN	NaN	NaN	NaN	NaN	NaN	Q9UHW5;Q9UHW	Q9UHW5;Q9UHW	GPN-loop GTPase	GPN3
24.2107	24.7323	24.3248	24.0388	24.658	24.471	NaN	NaN	NaN	24.1438	23.7291	NaN	Q5VW38;Q5VW38	Q5VW38;Q5VW38	Protein GPR107	GPR107
23.1957	23.4625	23.7311	23.1496	23.183	NaN	24.0485	NaN	23.7551	23.5807	23.3639	NaN	Q86SQ4;Q86SQ4-	Q86SQ4;Q86SQ4-	G-protein couplec	GPR126
NaN	NaN	NaN	NaN	NaN	NaN	NaN	22.0547	NaN	NaN	NaN	NaN	Q86V85	Q86V85	Integral membrar	GPR180
NaN	NaN	21.8453	NaN	NaN	NaN	NaN	NaN	NaN	NaN	NaN	NaN	O43194	O43194	G-protein couplec	GPR39
22.8996	24.6547	23.8633	22.9661	24.4333	22.2352	26.4304	25.7982	26.2344	26.2405	25.2427	25.7081	Q9Y653;Q9Y653-2	Q9Y653;Q9Y653-2	G-protein couplec	GPR56;ADGRG1
25.9018	24.0315	24.5452	24.5811	NaN	23.7446	NaN	NaN	23.6256	NaN	NaN	NaN	Q8NFJ5;F5GWG3	Q8NFJ5	Retinoic acid-indu	GPRC5A
NaN	22.6744	23.0436	NaN	22.6837	23.3899	NaN	NaN	NaN	NaN	NaN	NaN	Q9NQ84;A8MXZ4	Q9NQ84;A8MXZ4	G-protein couplec	GPRC5C
25.2079	26.3896	26.1301	25.3713	25.7698	26.1215	25.8301	NaN	NaN	26.4152	25.9456	NaN	Q7Z2K8;Q7Z2K8-2	Q7Z2K8;Q7Z2K8-2	G protein-regulat	GPRIN1
26.5153	26.1622	26.4538	26.309	26.3618	26.6889	26.1063	26.3275	26.3609	26.39	26.3536	26.6637	Q13098;C9JFE4;Q	Q13098;C9JFE4;Q	COP9 signalosom	GPS1
28.2159	28.2914	28.3784	28.0652	27.9945	28.2643	NaN	NaN	NaN	NaN	NaN	NaN	A0A0A0MSK4;Q8	A0A0A0MSK4;Q8	G-protein-signalin	GPSM1
22.5187	NaN	25.181	24.3571	25.0059	25.5937	24.9445	NaN	NaN	24.5114	25.4364	NaN	P81274;H0Y4A4;C	P81274	G-protein-signalin	GPSM2
NaN	NaN	NaN	NaN	22.1777	NaN	NaN	NaN	NaN	NaN	NaN	NaN	Q8TED1;E7ETY7;J	Q8TED1;E7ETY7	Probable glutathi	GPX8
26.9489	26.8872	26.9428	26.4718	26.3514	26.8767	26.6603	25.8142	26.3162	26.9842	26.5589	26.5081	P62993;P62993-2	P62993;P62993-2	Growth factor rec	GRB2
26.0568	25.6246	25.7146	25.6949	25.8768	25.9718	25.8866	25.8078	25.9544	26.3597	26.4447	26.0808	Q9UBQ7;U3KQ56	Q9UBQ7;U3KQ56	Glyoxylate reduct	GRHPR
24.1158	23.9399	23.6788	23.5754	23.3314	NaN	NaN	NaN	NaN	NaN	NaN	NaN	Q4V328;A0A087V	Q4V328;A0A087V	GRIP1-associated	GRIPAP1
24.3764	24.2466	24.8611	24.5804	24.4363	24.9843	23.988	NaN	NaN	24.7647	24.6569	24.5853	P43250-2;P43250	P43250-2;P43250	G protein-couplec	GRK6
21.9533	23.3502	22.3922	NaN	22.8643	NaN	NaN	25.2274	24.212	NaN	NaN	22.7527	P28799;K7EQ05;P	P28799;K7EQ05;P	Granulins;Acrogra	GRN
26.0187	27.2166	26.0854	26.0191	26.7753	25.8309	26.2098	26.5649	26.8841	26.2927	26.325	26.41	Q9HAV7	Q9HAV7	GrpE protein hom	GRPEL1
25.6593	25.6802	24.1853	25.4019	25.3374	NaN	24.3703	NaN	NaN	NaN	NaN	NaN	Q12849;H0Y8R1;F	Q12849;H0Y8R1;F	G-rich sequence f	GRSF1
26.6132	24.237	24.8655	25.397	24.3418	24.882	24.8114	NaN	NaN	25.5437	25.4118	25.1243	Q9BQ67;M0QX71	Q9BQ67;M0QX71	Glutamate-rich W	GRWD1
24.23	23.6599	24.0451	23.9406	23.9822	24.3108	NaN	NaN	NaN	23.6853	23.4885	24.0776	P57764;G3V1A6;E	P57764;G3V1A6;E	Gasdermin-D	GSDMD
25.0733	23.7385	24.1752	24.0174	23.4839	23.865	23.7257	NaN	23.8283	24.3175	24.1262	24.4975	P49840;A8MT37;I	P49840;A8MT37	Glycogen synthas	GSK3A
25.7883	24.7356	25.3882	25.6271	24.6178	24.8187	25.2545	24.6059	24.9766	25.7517	25.3213	NaN	P49841;P49841-2	P49841;P49841-2	Glycogen synthas	GSK3B
24.3491	24.3988	24.5337	24.2365	23.8089	24.2217	24.4349	NaN	NaN	23.3697	23.6063	NaN	P06396;A0A0A0M	P06396;A0A0A0M	Gelsolin	GSN
29.0264	28.4826	28.489	28.7599	28.4379	28.4901	29.123	28.4842	28.4644	28.9683	28.8054	28.6661	P15170-3;P15170	P15170-3;P15170	Eukaryotic peptid	GSPT1;GSPT2
26.4375	27.3317	26.679	26.0513	27.0133	26.6471	26.558	26.7927	26.973	26.4637	26.8272	27.0473	P00390;P00390-2	P00390;P00390-2	Glutathione reduc	GSR
28.3492	29.6272	28.8131	28.077	29.2865	28.8739	29.3611	29.6085	29.6192	28.7284	29.0416	29.7848	P48637;P48637-2	P48637;P48637-2	Glutathione synth	GSS
24.9194	25.1798	24.8818	24.6689	24.9453	24.7868	24.3813	24.6779	25.1095	24.103	24.5027	NaN	Q9Y2Q3;E9PFN5;(	Q9Y2Q3;E9PFN5;(	Glutathione S-trar	GSTK1
25.9349	24.9674	25.2465	25.4668	24.5239	25.2071	24.8653	NaN	24.4656	25.3604	24.997	24.8753	P09488;P09488-2	P09488;P09488-2	Glutathione S-trar	GSTM1;GSTM4
28.2062	27.4797	27.7157	28.1031	27.1246	27.8334	27.1116	NaN	27.5544	27.9479	27.1276	27.2964	P21266;A0A0A0M	P21266;A0A0A0M	Glutathione S-trar	GSTM3
29.6718	30.0725	29.3873	29.052	29.5571	29.474	30.2009	29.5098	29.2373	29.7191	29.7338	29.7477	P78417;Q5TA02;P	P78417;Q5TA02;P	Glutathione S-trar	GSTO1
29.1891	29.6003	29.4051	29.1782	29.198	29.3287	30.2408	29.1637	29.3872	29.7322	29.5866	29.4527	P09211;A8MX94;/	P09211;A8MX94	Glutathione S-trar	GSTP1
22.6507	23.4401	23.4511	22.9191	23.8935	23.6161	23.1172	NaN	23.5165	22.9747	23.1478	NaN	P0CG30;P0CG29;(	P0CG30;P0CG29;(	Glutathione S-trar	GSTT2B;GSTT2;Em
26.8959	26.607	26.3579	26.3317	26.7871	26.0483	25.5864	NaN	25.9708	26.1285	26.2913	26.236	G3V5T0;A0A0C4D	G3V5T0;A0A0C4D	Maleylacetoaceta	GSTZ1
24.0354	NaN	23.7421	23.8789	23.6887	23.6812	NaN	24.2339	NaN	23.3637	23.7032	24.2585	P52655;J3KNC0;P	P52655;J3KNC0;P	Transcription initi	GTF2A1
25.0814	23.9339	NaN	25.3516	23.8772	NaN	NaN	NaN	NaN	NaN	NaN	NaN	P52657;A8MYR4;I	P52657;A8MYR4;I	Transcription initi	GTF2A2
22.5161	NaN	NaN	22.6386	NaN	NaN	NaN	NaN	NaN	NaN	NaN	NaN	Q00403;B1APE1;E	Q00403;B1APE1;E	Transcription initi	GTF2B
NaN	NaN	NaN	21.6253	21.3999	21.9174	NaN	NaN	NaN	NaN	NaN	NaN	P29083;E9PER7;C	P29083;E9PER7;C	General transcript	GTF2E1
21.3374	NaN	NaN	NaN	NaN	NaN	NaN	NaN	NaN	NaN	NaN	NaN	P29084;E5RH41	P29084	Transcription initi	GTF2E2
23.8529	23.8625	24.1407	23.5806	24.2551	24.2878	23.601	23.9983	NaN	23.9266	24.1787	24.7253	P35269;M0R0Z3;I	P35269;M0R0Z3;I	General transcript	GTF2F1
23.5065	23.8395	23.4777	23.4454	24.0636	23.6403	NaN	NaN	NaN	NaN	NaN	NaN	P13984	P13984	General transcript	GTF2F2
22.9624	NaN	NaN	NaN	NaN	NaN	NaN	NaN	NaN	NaN	NaN	NaN	Q6P1K8;Q13888;I	Q6P1K8;Q13888;I	General transcript	GTF2H2C;GTF2H2
NaN	NaN	NaN	21.8329	22.9	NaN	NaN	NaN	NaN	NaN	NaN	NaN	Q13889;F5H6X0;F	Q13889;F5H6X0;F	General transcript	GTF2H3
22.5599	NaN	NaN	NaN	NaN	NaN	NaN	NaN	NaN	NaN	NaN	NaN	Q92759;Q92759-2	Q92759;Q92759-2	General transcript	GTF2H4
NaN	NaN	NaN	NaN	NaN	NaN	NaN	NaN	NaN	NaN	20.9529	NaN	Q6ZYL4	Q6ZYL4	General transcript	GTF2H5
25.6731	24.6115	25.3934	25.9038	25.1197	25.6161	23.2594	25.6727	23.8154	26.5233	25.8323	25.8231	P78347;P78347-2	P78347;P78347-2	General transcript	GTF2I
24.0885	NaN	22.748	24.0132	23.4751	22.9545	NaN	NaN	NaN	23.0328	23.4585	NaN	Q9UKN8;F2Z356	Q9UKN8	General transcript	GTF3C4
24.0352	NaN	23.6458	24.1952	23.5982	23.807	NaN	NaN	NaN	24.0703	23.9093	NaN	Q9Y5Q8;Q9Y5Q8-	Q9Y5Q8;Q9Y5Q8-	General transcript	GTF3C5

25.9862	24.4796	25.1491	25.6217	24.7883	24.7958	24.6675	24.5638	24.5657	24.6014	24.7516	24.8385	O00178;B0QY59;F	O00178	GTP-binding prote	GTPBP1
23.8652	23.4464	23.3936	23.7312	24.1647	23.8866	NaN	NaN	NaN	NaN	NaN	NaN	A4D1E9;A4D1E9-2	A4D1E9;A4D1E9-2	GTP-binding prote	GTPBP10
NaN	NaN	22.4814	NaN	22.0263	NaN	NaN	NaN	NaN	NaN	NaN	NaN	Q9BX10;Q9BX10-2	Q9BX10;Q9BX10-2	GTP-binding prote	GTPBP2
27.8054	26.8427	27.7434	28.6564	27.8021	27.771	25.7061	26.0546	25.9535	27.7558	27.7498	27.6052	Q9BZE4;Q9BZE4-2	Q9BZE4;Q9BZE4-2	Nucleolar GTP-bin	GTPBP4
NaN	NaN	NaN	22.3602	NaN	NaN	NaN	NaN	NaN	NaN	NaN	NaN	O43824;H0Y2S1	O43824;H0Y2S1	Putative GTP-binc	GTPBP6
NaN	22.1839	22.2717	21.7029	23.2144	NaN	NaN	NaN	NaN	21.8207	22.8003	NaN	Q9NYZ3	Q9NYZ3	G2 and S phase-e	GTSE1
25.5131	24.4658	NaN	24.7849	NaN	NaN	NaN	NaN	NaN	NaN	NaN	NaN	Q8N442	Q8N442	Translation factor	GUF1
23.5581	24.4898	23.7242	23.6291	24.281	23.8201	23.897	NaN	24.0623	23.73	23.7547	24.5858	Q16774;B1ANH5;	Q16774;B1ANH5;	Guanylate kinase	GUK1
NaN	NaN	21.7233	NaN	NaN	NaN	NaN	NaN	NaN	NaN	22.0117	NaN	Q9UBP9;Q9UBP9-	Q9UBP9;Q9UBP9-	PTB domain-conta	GULP1
NaN	NaN	NaN	NaN	NaN	NaN	23.5	NaN	NaN	NaN	NaN	NaN	P08236;P08236-3	P08236;P08236-3	Beta-glucuronidas	GUSB
24.4081	24.7626	24.1804	23.7219	24.7515	24.0817	24.4332	25.1343	24.6663	24.122	24.4449	24.5942	P46976;P46976-2	P46976;P46976-2	Glycogenin-1	GYG1
25.927	24.6945	25.0353	25.8492	24.4561	24.8523	23.1576	25.0261	25.2225	25.1061	25.0825	25.1014	P13807;P13807-2	P13807;P13807-2	Glycogen [starch]	GYS1
26.0336	26.3427	26.1767	27.7761	26.5896	26.4071	27.2233	NaN	NaN	26.3057	27.5107	26.4822	P07305;P07305-2	P07305;P07305-2	Histone H1.0;Hist	H1F0
24.0219	23.8419	23.7874	26.2091	23.7318	23.4335	23.3141	NaN	NaN	23.8319	26.0298	NaN	Q92522	Q92522	Histone H1x	H1FX
27.1824	27.5201	26.6839	28.3549	27.1349	26.8449	27.5448	26.5831	24.8844	26.8451	26.7003	26.0352	Q71UI9;P0C0S5;C	Q71UI9;P0C0S5;C	Histone H2A.V;His	H2AFV;H2AFZ
NaN	NaN	NaN	NaN	NaN	NaN	NaN	NaN	NaN	NaN	23.4109	NaN	P16104	P16104	Histone H2AX	H2AFX
25.8493	25.5563	25.4043	26.6734	25.583	25.1374	27.1643	24.4822	NaN	25.9098	27.1891	26.9527	O75367;O75367-2	O75367;O75367-2	Core histone mac	H2AFY
NaN	NaN	NaN	NaN	NaN	NaN	NaN	NaN	22.8109	NaN	NaN	NaN	O95479;R4GMU1	O95479;R4GMU1	GDH/6PGL endop	H6PD
NaN	NaN	NaN	NaN	NaN	NaN	NaN	NaN	NaN	NaN	23.5636	NaN	Q6Y1H2	Q6Y1H2	Very-long-chain (3	HACD2
24.8251	24.4324	24.8244	25.0549	24.815	25.1522	25.1912	25.2804	25.6923	25.419	24.9548	24.5933	Q9P035;H3BS72;H	Q9P035;H3BS72;H	Very-long-chain (3	HACD3
25.0935	26.1599	25.3321	25.2225	26.0644	24.9494	25.0016	25.6826	26.7572	25.8004	25.9354	26.0135	Q16836;E9PF18;A	Q16836;E9PF18;A	Hydroxyacyl-coen	HADH
26.9784	26.6679	26.5117	27.1388	27.0395	26.6252	26.3913	27.121	27.5969	26.0724	26.2334	26.2937	P40939;H0YFD6;P	P40939	Trifunctional enzy	HADHA
26.8572	27.2789	26.8504	27.478	27.6776	27.003	26.5059	27.8686	28.4166	26.3727	26.8711	27.2132	P55084;P55084-2	P55084;P55084-2	Trifunctional enzy	HADHB
24.5234	25.6361	25.0351	24.4526	25.2508	24.949	24.5689	NaN	NaN	25.2836	24.8273	24.5427	Q16775;H3BPK3;(	Q16775;H3BPK3;(	Hydroxyacylglutat	HAGH
NaN	NaN	NaN	NaN	NaN	NaN	NaN	NaN	22.9819	23.182	NaN	NaN	Q96S86;H3BTH8;H	Q96S86;H3BTH8;H	Hyaluronan and p	HAPLN3
27.5336	27.8429	27.4553	27.1123	27.4563	27.3637	28.0187	28.1112	27.7279	27.4587	27.7546	27.9554	P12081;P12081-4	P12081;P12081-4	Histidine--tRNA lig	HARS
21.8879	NaN	NaN	NaN	NaN	NaN	NaN	NaN	NaN	NaN	NaN	NaN	P49590;P49590-2	P49590;P49590-2	Probable histidine	HARS2
25.4221	24.9384	25.0906	25.1021	25.0475	24.9436	24.5725	24.8498	24.3131	24.6101	25.1687	25.2734	O14929;O14929-2	O14929;O14929-2	Histone acetyltrar	HAT1
22.6865	NaN	NaN	NaN	NaN	NaN	NaN	NaN	NaN	NaN	NaN	NaN	Q96CS2;Q96CS2-2	Q96CS2;Q96CS2-2	HAUS augmin-like	HAUS1
23.2208	NaN	23.1184	23.2847	23.1587	23.0724	NaN	NaN	NaN	NaN	23.118	NaN	O94927;O94927-2	O94927	HAUS augmin-like	HAUS5
21.4182	21.656	NaN	21.8723	NaN	NaN	21.5238	NaN	NaN	NaN	NaN	NaN	Q9BT25;Q9BT25-2	Q9BT25;Q9BT25-2	HAUS augmin-like	HAUS8
NaN	NaN	NaN	NaN	NaN	NaN	NaN	NaN	NaN	29.0023	NaN	NaN	P69905	P69905	Hemoglobin subu	HBA1
NaN	NaN	NaN	22.1632	NaN	NaN	NaN	NaN	NaN	NaN	NaN	NaN	Q99075	Q99075	Proheparin-bindin	HBEGF
25.9779	24.9333	25.385	25.6084	25.0869	25.2983	24.8906	25.3977	25.1604	24.9234	25.2752	25.1873	Q9Y450;B7Z524;H	Q9Y450;B7Z524;H	HBS1-like protein	HBS1L
NaN	NaN	22.6036	23.2284	NaN	NaN	NaN	NaN	NaN	22.5095	NaN	NaN	P53701	P53701	Cytochrome c-typ	HCCS
26.0052	25.387	25.7367	25.7056	25.5143	25.7738	24.6644	24.0918	NaN	25.7403	25.427	24.1894	P51610;P51610-4	P51610;P51610-4	Host cell factor 1;	HCFC1
25.4448	24.9709	25.2648	24.4196	24.6571	24.7727	24.7847	25.1319	24.5756	23.5587	NaN	24.553	P14317;E7EVW7;F	P14317;E7EVW7	Hematopoietic lin	HCLS1
28.6419	27.254	28.2063	28.8095	27.7431	28.4926	27.1161	27.5597	26.7665	28.9022	28.753	27.8504	Q13547;Q5TEE2	Q13547;Q5TEE2	Histone deacetyla	HDAC1
NaN	NaN	NaN	NaN	NaN	NaN	NaN	NaN	NaN	21.1512	NaN	NaN	Q96DB2;F8WF94;	Q96DB2;F8WF94;	Histone deacetyla	HDAC11
26.9116	25.6297	26.4362	26.7308	25.748	26.5492	25.8292	25.9305	25.2364	27.6197	27.3593	26.5993	Q92769;Q92769-3	Q92769;Q92769-3	Histone deacetyla	HDAC2
23.1066	NaN	22.5146	22.7831	NaN	22.7566	NaN	NaN	NaN	NaN	NaN	NaN	O15379;O15379-2	O15379;O15379-2	Histone deacetyla	HDAC3
24.1056	23.5352	24.1556	25.0976	24.418	24.173	NaN	NaN	23.2898	24.2232	24.0509	23.6572	Q9UBN7;Q9UBN7	Q9UBN7;Q9UBN7	Histone deacetyla	HDAC6
23.3809	23.536	23.4563	23.7776	23.8835	NaN	23.6633	NaN	NaN	23.8078	24.2317	NaN	Q7Z4H3;V9GYS5;V	Q7Z4H3;V9GYS5;V	HD domain-contai	HDDC2
25.6637	26.025	25.9078	25.505	25.8714	26.2114	25.9986	26.2389	25.849	26.5852	26.7809	26.5457	P51858;P51858-3	P51858;P51858-3	Hepatoma-derive	HDGF
29.0538	28.1829	28.2206	28.6329	28.0338	28.0848	27.8362	27.8766	28.3067	26.166	27.026	25.9232	A0A024R4E5;Q00	A0A024R4E5;Q00	Vigilin	HDLBP
26.2737	23.6835	25.5754	27.486	25.3269	25.6479	NaN	NaN	NaN	25.7253	24.8	NaN	Q9H583;Q5T3Q7;	Q9H583;Q5T3Q7	HEAT repeat-cont	HEATR1
22.5396	NaN	22.2833	22.7044	22.2045	21.9977	NaN	NaN	NaN	22.6272	NaN	NaN	Q7Z4Q2;Q7Z4Q2-	Q7Z4Q2	HEAT repeat-cont	HEATR3
NaN	NaN	23.4481	NaN	NaN	23.0111	NaN	NaN	NaN	NaN	NaN	NaN	Q86XA9;F5H619;H	Q86XA9;F5H619;H	HEAT repeat-cont	HEATR5A
24.2452	23.3919	23.6372	23.9249	23.3917	23.0207	NaN	NaN	23.5451	23.7127	23.2072	23.3541	Q9P2D3;Q9P2D3-	Q9P2D3;Q9P2D3-	HEAT repeat-cont	HEATR5B
23.5881	NaN	22.6184	23.2801	NaN	NaN	NaN	NaN	NaN	NaN	NaN	NaN	Q6AI08;A0A087W	Q6AI08;A0A087W	HEAT repeat-cont	HEATR6
26.2948	27.6834	27.196	26.2165	27.3954	27.059	27.7184	27.6317	27.7013	27.4677	27.5916	27.9065	Q9NRV9;F5GWX2	Q9NRV9;F5GWX2	Heme-binding pro	HEBP1
25.5091	25.7556	25.076	24.1281	25.6176	24.7069	26.0835	25.5862	25.3414	25.0563	25.679	25.4541	Q9Y5Z4;Q9Y5Z4-2	Q9Y5Z4;Q9Y5Z4-2	Heme-binding pro	HEBP2
25.9477	24.7904	25.2531	25.7596	24.7195	25.138	25.3672	23.6747	25.0634	25.0438	24.7687	24.9761	Q9ULT8;A0A087X	Q9ULT8;A0A087X	E3 ubiquitin-prote	HECTD1
22.9263	22.9336	23.1077	23.3457	NaN	23.5464	NaN	NaN	NaN	NaN	23.4956	NaN	Q5T447;Q5T447-2	Q5T447	E3 ubiquitin-prote	HECTD3
NaN	NaN	NaN	NaN	NaN	22.1675	NaN	NaN	NaN	NaN	NaN	NaN	Q9ULI3;H7C4K2;H	Q9ULI3;H7C4K2;H	Protein HEG hom	HEG1
27.1272	NaN	NaN	NaN	NaN	NaN	NaN	NaN	NaN	NaN	NaN	NaN	Q9NRZ9;A0A087V	Q9NRZ9;A0A087V	Lymphoid-specific	HELLS
26.3995	27.0256	26.4281	26.1187	26.4208	26.0883	26.7956	26.2959	26.236	26.0194	25.4085	25.4016	V9HW75;A0A087V	V9HW75;A0A087V	Nucleobindin-2;N	HEL-S-109;NUCB2;
26.98	23.9272	25.3715	26.8043	25.4319	25.5329	23.4104	NaN	23.4908	24.7783	24.7687	24.4178	Q9BYK8;Q9BYK8-2	Q9BYK8;Q9BYK8-2	Helicase with zinc	HELZ2
25.9199	23.5708	25.1546	25.4867	23.7602	24.2224	NaN	NaN	NaN	22.9428	24.1833	NaN	O95714;A0A0J9Y	O95714;A0A0J9Y	E3 ubiquitin-prote	HERC2
26.654	25.7314	26.509	26.3477	25.6397	26.1273	26.1791	25.7752	24.6037	26.1159	26.0744	25.5447	Q5GLZ8;Q5GLZ8-3	Q5GLZ8;Q5GLZ8-3	Probable E3 ubiqu	HERC4

25.6203	23.8541	24.7255	25.3073	24.0059	24.7082	23.3302	NaN	NaN	23.9289	24.3438	NaN	Q9UII4;E9PBL0	Q9UII4	E3 ISG15--protein	HERC5
24.1704	25.4397	24.4712	24.1119	24.6673	24.2761	26.8333	26.3004	25.4124	23.884	23.81	22.9795	P06865;H3BS10;H	P06865;H3BS10;H	Beta-hexosaminid	HEXA
27.6364	28.1561	27.2197	27.2022	27.3985	27.1936	29.1359	28.7958	28.3374	27.6012	27.3325	26.9922	P07686;Q5URX0;I	P07686;Q5URX0	Beta-hexosaminid	HEXB
23.2643	NaN	NaN	NaN	NaN	NaN	NaN	NaN	NaN	21.9805	NaN	NaN	O94992	O94992	Protein HEXIM1	HEXIM1
23.8508	24.5881	24.8013	24.0837	24.7906	25.5161	NaN	NaN	NaN	NaN	NaN	23.7249	Q30201;F8W7W8	Q30201;F8W7W8	Hereditary hemoc	HFE
24.6178	23.7947	23.804	24.5158	23.807	23.8596	23.4476	NaN	NaN	24.2469	24.3294	24.6044	Q9BTY7;E9PIX0	Q9BTY7	Protein HGH1 hor	HGH1
27.2288	27.5867	27.0957	26.8045	27.0223	26.8742	26.9788	25.6176	27.4137	26.4652	26.353	26.9751	O14964;O14964-2	O14964;O14964-2	Hepatocyte growt	HGS
23.2107	24.4725	23.1524	23.0685	24.0109	NaN	NaN	NaN	23.8133	NaN	23.078	24.1301	P31937;H7BZL2	P31937;H7BZL2	3-hydroxyisobuty	HIBADH
NaN	23.3919	22.6867	22.4068	22.8617	NaN	NaN	NaN	23.9659	22.4906	22.0202	23.1139	Q6NVY1;B8ZZZ0;A	Q6NVY1;B8ZZZ0;A	3-hydroxyisobuty	HIBCH
NaN	23.2105	NaN	NaN	NaN	NaN	NaN	NaN	NaN	NaN	NaN	NaN	Q96JB3;Q96JB3-2	Q96JB3;Q96JB3-2	Hypermethylated	HIC2
22.6572	NaN	NaN	NaN	NaN	NaN	NaN	NaN	NaN	NaN	NaN	NaN	Q8IV36;Q8IV36-2;	Q8IV36;Q8IV36-2;	Protein HID1	HID1
NaN	22.3927	21.9265	NaN	23.3109	NaN	NaN	NaN	NaN	21.7242	NaN	NaN	Q9NWT6;E9PL41	Q9NWT6	Hypoxia-inducible	HIF1AN
NaN	NaN	NaN	NaN	NaN	NaN	NaN	NaN	23.3286	NaN	NaN	NaN	Q9Y241;C9JAW5;(	Q9Y241;C9JAW5;(	HIG1 domain fam	HIGD1A
28.2417	30.1817	29.5459	28.8732	30.1981	29.7317	29.9146	29.5554	29.6325	30.0521	30.0688	30.3721	P49773;D6REP8;D	P49773	Histidine triad nuc	HINT1
24.2001	25.4635	23.9757	23.9871	24.9983	24.0142	24.5092	24.8424	NaN	24.3456	24.8092	NaN	Q9BX68	Q9BX68	Histidine triad nuc	HINT2
23.134	24.4102	24.1198	23.7699	23.8715	NaN	NaN	NaN	NaN	23.4947	NaN	NaN	Q9NQE9	Q9NQE9	Histidine triad nuc	HINT3
26.5101	26.7405	27.261	27.08	27.0328	27.1144	26.2481	25.8989	NaN	26.9259	26.9126	25.6934	O75146;O75146-2	O75146;O75146-2	Huntingtin-interac	HIP1R
26.2759	26.6218	26.0348	27.1389	25.7572	26.4559	27.8081	26.4998	25.4192	26.2935	27.0878	26.8215	P16403	P16403	Histone H1.2	HIST1H1C
29.6426	30.2379	30.0487	31.3146	30.4316	30.4452	31.2919	30.4195	29.2233	30.1389	31.7252	31.4571	P10412;P16402;Q	P10412;P16402	Histone H1.4;Hist	HIST1H1E;HIST1H1
27.4768	26.7832	26.0083	27.9814	26.4117	27.1969	27.1507	NaN	NaN	26.2591	26.6895	25.6894	Q93077;Q7L7L0;P	Q93077;Q7L7L0;P	Histone H2A type	HIST1H2AC;HIST3I
32.3873	32.1613	31.7414	33.2689	31.9671	31.9229	32.3265	30.8817	29.6563	31.2556	31.955	31.702	P0C0S8;Q99878;C	P0C0S8;Q99878;C	Histone H2A type	HIST1H2AG;HIST1I
NaN	NaN	24.0119	NaN	26.9637	NaN	NaN	NaN	NaN	NaN	26.0166	NaN	P62807;B2R4S9	P62807;B2R4S9	Histone H2B type	HIST1H2BC;HIST1I
NaN	NaN	NaN	NaN	NaN	NaN	NaN	NaN	26.6963	NaN	NaN	NaN	P58876	P58876	Histone H2B type	HIST1H2BD
32.9998	33.3759	33.0283	34.0464	33.612	33.3271	33.6925	32.7224	31.6303	32.5365	33.8086	33.947	Q93079;Q5QNW6	Q93079;Q5QNW6	Histone H2B type	HIST1H2BH;HIST2I
NaN	NaN	NaN	23.8915	25.4143	NaN	NaN	26.6075	25.5728	NaN	25.4785	NaN	O60814;P57053	O60814;P57053	Histone H2B type	HIST1H2BK;H2BFS
NaN	NaN	NaN	25.3464	NaN	NaN	NaN	NaN	NaN	NaN	NaN	NaN	Q99879	Q99879	Histone H2B type	HIST1H2BM
NaN	NaN	NaN	NaN	NaN	NaN	NaN	NaN	NaN	NaN	25.1202	NaN	Q99877;U3KQK0	Q99877;U3KQK0	Histone H2B type	HIST1H2BN
28.9298	29.1029	29.0635	30.171	29.4795	29.331	29.2614	28.8517	27.6385	28.7618	30.1036	30.057	P23527	P23527	Histone H2B type	HIST1H2BO
32.6324	32.8606	32.209	33.6048	32.7267	32.494	33.4554	31.5552	31.4482	31.6209	32.919	32.9988	P62805	P62805	Histone H4	HIST1H4A
NaN	22.977	24.3234	27.2004	NaN	26.737	NaN	NaN	NaN	NaN	23.4264	23.0073	Q6FI13;Q16777	Q6FI13;Q16777	Histone H2A type	HIST2H2AA3;HIST2
32.4332	32.8934	32.0901	33.0924	33.2935	32.6481	33.0232	32.2962	31.9937	31.4826	33.4027	33.8364	Q71DI3;P68431;P	Q71DI3;P68431;P	Histone H3.2;Hist	HIST2H3A;HIST1H3
27.6842	27.5615	26.741	29.0333	27.1792	24.1143	28.3694	26.6557	26.3811	27.5801	28.5327	28.4466	Q5TEC6	Q5TEC6	Histone H3	HIST2H3PS2
NaN	NaN	NaN	22.7741	NaN	NaN	NaN	NaN	NaN	NaN	22.9497	NaN	Q8NCD3;C9JWC4;	Q8NCD3;C9JWC4;	Holliday junction r	HJURP
26.3174	26.4172	26.4088	26.7656	26.573	26.5557	26.7352	27.5987	28.0954	25.9322	25.9742	26.7776	P19367;P19367-4,	P19367;P19367-4,	Hexokinase-1	HK1
29.4624	27.931	28.344	28.9052	27.7946	27.9819	28.2742	28.4693	28.1453	28.0038	27.8304	27.5063	P52789;E9PB90;P	P52789;E9PB90	Hexokinase-2;Hex	HK2
32.5238	33.0615	33.5502	32.8656	33.4004	33.8599	32.7105	32.6213	32.9818	33.4817	33.0405	32.8169	P01892;A0A0G2JI	P01892;A0A0G2JI	HLA class I histocc	HLA-A
NaN	NaN	NaN	23.9131	NaN	NaN	NaN	NaN	NaN	NaN	NaN	NaN	P10316;P01891	P10316;P01891	HLA class I histocc	HLA-A
NaN	NaN	NaN	NaN	NaN	NaN	22.8774	NaN	NaN	NaN	NaN	NaN	P30459;Q9GJ45;P	P30459;Q9GJ45;P	HLA class I histocc	HLA-A
NaN	NaN	NaN	NaN	NaN	NaN	NaN	NaN	NaN	NaN	NaN	NaN	P01889;F6U0H7	P01889;F6U0H7	HLA class I histocc	HLA-B
30.882	31.389	32.0816	31.5052	31.4821	32.2697	30.8135	30.0981	30.9078	31.3921	30.6542	30.6168	P30479;Q31611;A	P30479	HLA class I histocc	HLA-B
NaN	NaN	NaN	22.7142	NaN	NaN	NaN	NaN	NaN	NaN	NaN	NaN	P30488;P30483;P	P30488;P30483;P	HLA class I histocc	HLA-B
26.1087	27.069	27.5107	27.1679	27.936	27.6325	26.6095	27.1395	27.667	27.3758	27.3328	27.147	Q04826;P30485;P	Q04826;P30485;P	HLA class I histocc	HLA-B
24.0168	24.5148	24.645	24.2184	24.1019	25.3359	NaN	NaN	NaN	NaN	24.9237	NaN	P10321;A2AEA2	P10321;A2AEA2	HLA class I histocc	HLA-C
NaN	NaN	NaN	22.6993	NaN	NaN	NaN	NaN	NaN	NaN	NaN	NaN	P30508;P30510;Q	P30508;P30510;Q	HLA class I histocc	HLA-C
27.5901	27.8775	28.3723	27.7434	27.7598	28.3799	28.0156	27.6562	28.1905	28.2887	27.6041	27.3257	Q95604;A0A0G2JI	Q95604	HLA class I histocc	HLA-C
NaN	NaN	NaN	NaN	NaN	NaN	20.8408	NaN	NaN	NaN	NaN	NaN	P13747;Q6DU44	P13747;Q6DU44	HLA class I histocc	HLA-E
26.6527	25.8255	26.3794	26.8281	25.8157	26.4576	26.9329	26.2568	27.0951	27.2182	26.2679	26.6263	Q8TCT9;Q8TCT9-5	Q8TCT9;Q8TCT9-5	Minor histocompa	HM13
23.8944	24.5026	24.2284	23.9809	24.7376	24.6213	23.9085	NaN	NaN	24.3283	NaN	NaN	P08397;P08397-3,	P08397;P08397-3,	Porphobilinogen c	HMBS
NaN	NaN	NaN	22.6069	NaN	NaN	NaN	NaN	NaN	NaN	NaN	NaN	Q96FZ2;D6RAZ3;E	Q96FZ2;D6RAZ3;E	Embryonic stem c	HMCES
29.2054	28.5826	29.1496	30.2731	29.2687	29.3846	28.2184	27.1596	27.2807	29.0446	29.2797	28.3001	P17096;P17096-3	P17096;P17096-3	High mobility grou	HMGA1
25.9073	NaN	NaN	26.2033	25.8464	NaN	NaN	NaN	NaN	26.0266	25.8834	NaN	P17096-2	P17096-2	High mobility grou	HMGA1
NaN	23.7255	NaN	NaN	23.5291	NaN	NaN	NaN	NaN	NaN	23.1578	NaN	P52926;F5H2U8;F	P52926;F5H2U8;F	High mobility grou	HMGA2
28.288	28.4556	28.2428	28.4132	28.5089	28.5847	28.8422	28.8826	28.5794	28.6415	29.1928	29.2411	P09429;Q5T7C4;B	P09429;Q5T7C4;B	High mobility grou	HMGB1;HMGB1P1
26.3439	26.2745	26.0467	26.421	26.1784	26.1574	26.6225	27.0022	26.3611	26.4985	26.7145	27.1557	P26583;D6R9A6	P26583;D6R9A6	High mobility grou	HMGB2
23.9302	NaN	24.0914	23.5978	24.0118	24.6418	NaN	NaN	NaN	23.9492	24.7617	24.7146	O15347;E9PES6;E	O15347;E9PES6;E	High mobility grou	HMGB3
21.5491	NaN	NaN	NaN	NaN	NaN	NaN	NaN	NaN	NaN	NaN	NaN	P35914;P35914-3,	P35914;P35914-3,	Hydroxymethylglu	HMGCL
25.0188	24.7808	24.8961	24.819	23.7399	24.4738	26.2199	25.811	24.4932	26.0862	26.142	25.2837	Q01581;D6RIW1	Q01581	Hydroxymethylglu	HMGCS1
NaN	NaN	NaN	25.3434	NaN	NaN	NaN	NaN	NaN	NaN	NaN	NaN	P05114;F2Z2W6;F	P05114;F2Z2W6;F	Non-histone chro	HMGN1
24.7128	24.7628	23.7525	24.3791	23.7597	NaN	NaN	NaN	NaN	24.7239	23.7261	NaN	P05204;Q15651;A	P05204	Non-histone chro	HMGN2
23.6591	24.1555	23.9123	24.1762	24.3023	NaN	NaN	NaN	NaN	23.9575	24.0684	NaN	O00479	O00479	High mobility grou	HMGN4

26.238	25.3118	25.9586	25.7815	25.4749	25.4718	24.2528	NaN	NaN	24.3996	24.2326	NaN	Q92619;K7ES98;K	Q92619;K7ES98;K	Minor histocompa	HMHA1
21.7458	NaN	NaN	NaN	NaN	NaN	NaN	NaN	NaN	NaN	NaN	NaN	O75330;O75330-4	O75330;O75330-4	Hyaluronan media	HMMR
26.3236	26.6968	26.5489	26.2253	26.6267	26.1943	26.4457	27.0996	26.4518	26.6387	26.144	25.3074	P30519;A0A087W	P30519;A0A087W	Heme oxygenase	HMOX2
26.0263	27.5249	26.9024	25.3886	27.2583	26.7322	26.1698	27.5269	26.7053	25.7572	26.2216	26.5688	Q9UK76;Q9UK76-	Q9UK76;Q9UK76-	Hematological an	HN1
25.3827	26.5577	25.8591	25.0409	26.1608	25.7828	25.439	26.2896	25.013	25.1074	25.669	25.7992	Q9H910;Q9H910-	Q9H910;Q9H910-	Hematological an	HN1L
23.788	23.3765	23.6808	23.1603	23.462	23.9833	23.8436	NaN	NaN	23.8072	23.6514	23.8765	P50135;P50135-2	P50135	Histamine N-meth	HNMT
26.7226	27.5064	27.1884	27.1029	27.1645	27.1288	25.864	27.71	NaN	27.0591	27.531	27.0119	Q13151	Q13151	Heterogeneous nu	HNRNPA0
30.6179	31.056	30.9663	31.2242	30.9727	30.9974	30.6128	31.0032	30.4362	31.1807	31.4065	30.9253	P09651;F8W6I7;P	P09651;F8W6I7;P	Heterogeneous nu	HNRNPA1;HNRNP.
30.3531	30.8559	30.6875	30.6764	31.1092	30.8163	30.1832	31.001	30.5473	30.493	30.9587	30.8605	P22626;A0A087W	P22626;A0A087W	Heterogeneous nu	HNRNPA2B1
29.3546	28.8663	28.5793	29.1097	28.5784	28.881	28.0908	28.1834	27.6155	28.7217	28.7948	28.0249	P51991;H7C1J8	P51991	Heterogeneous nu	HNRNPA3
25.7614	25.5498	25.886	25.8356	25.8262	26.5996	25.3303	NaN	NaN	NaN	26.5393	NaN	P51991-2	P51991-2	Heterogeneous nu	HNRNPA3
28.4528	28.0946	27.8823	28.0263	28.0814	27.9069	27.9795	28.0797	27.1554	28.4163	29.1081	28.6123	Q99729-3;Q9972	Q99729-3;Q9972	Heterogeneous nu	HNRNPAB
31.3933	30.7388	30.9632	31.6666	30.8903	31.2487	29.5849	30.384	29.7803	30.3227	31.0019	30.6107	P07910-2;G3V4W	P07910-2;G3V4W	Heterogeneous nu	HNRNPC
NaN	NaN	NaN	23.1169	NaN	NaN	NaN	NaN	NaN	NaN	NaN	NaN	P07910;G3V2Q1;	P07910;G3V2Q1;	Heterogeneous nu	HNRNPC
29.1751	29.1877	28.9714	29.6339	28.9934	29.0393	28.8984	28.1948	28.0098	29.3963	29.2636	28.9068	Q14103;Q14103-3	Q14103;Q14103-3	Heterogeneous nu	HNRNPD
26.3749	25.4078	25.1337	26.0822	24.5897	24.87	23.8106	NaN	NaN	25.3228	25.0639	24.711	O14979-3;O1497	O14979-3;O1497	Heterogeneous nu	HNRNPDL
30.6456	29.5812	29.6963	30.4072	29.3328	29.9723	28.2193	28.1646	27.7677	29.3021	28.9471	28.6405	P52597	P52597	Heterogeneous nu	HNRNPF
29.7717	28.6926	28.5818	29.3752	28.7077	28.7565	28.0694	28.6756	28.0085	28.4541	28.4592	28.7231	P31943;G8JLB6;E	P31943;G8JLB6;E	Heterogeneous nu	HNRNPH1
25.9374	25.3773	24.94	25.7919	25.1177	25.0418	NaN	25.0456	NaN	24.3514	24.6483	24.6537	P55795	P55795	Heterogeneous nu	HNRNPH2
28.1664	28.5475	28.1432	28.3228	28.5545	28.587	27.7189	28.8183	27.896	28.7362	29.1594	28.8387	P31942;P31942-2	P31942;P31942-2	Heterogeneous nu	HNRNPH3
31.4167	30.691	30.8642	31.3344	30.9349	31.0308	29.9944	30.3798	29.5119	31.1276	31.0641	30.7183	P61978-3;P61978	P61978-3;P61978	Heterogeneous nu	HNRNPK
30.1639	29.1834	29.1338	29.9745	29.3634	29.3067	28.443	28.6727	28.5637	28.93	29.2977	28.7137	P14866;M0QXS5;	P14866;M0QXS5;	Heterogeneous nu	HNRNPL
25.3325	23.6523	24.2337	25.2084	24.115	24.7789	NaN	NaN	NaN	24.5532	24.4119	NaN	Q8WVV9;B7WPG	Q8WVV9;B7WPG	Heterogeneous nu	HNRNPLL;HNRPLL
NaN	NaN	NaN	NaN	NaN	NaN	NaN	NaN	NaN	NaN	NaN	22.3422	M0QZM1	M0QZM1		HNRNPM
31.1494	31.1137	31.0911	31.3342	31.4345	31.2303	29.6608	30.8354	29.9874	30.7835	31.2269	31.1347	P52272;P52272-2	P52272;P52272-2	Heterogeneous nu	HNRNPM
27.468	27.7727	27.4425	27.8311	27.8386	27.5522	26.1799	26.3684	26.8808	26.9078	27.2768	27.5766	O43390;O43390-2	O43390;O43390-2	Heterogeneous nu	HNRNPR
32.1735	31.3773	31.8248	32.6298	31.7747	32.177	30.0242	30.7857	30.3337	31.3237	31.8206	31.4807	Q00839;Q00839-2	Q00839;Q00839-2	Heterogeneous nu	HNRNPU
29.0956	27.8749	28.3145	29.118	28.3172	28.4989	26.5773	26.9873	26.0179	28.3548	28.4693	28.0704	Q9BUJ2;Q9BUJ2-2	Q9BUJ2;Q9BUJ2-2	Heterogeneous nu	HNRNPUL1
28.0657	27.2584	27.9874	28.4603	27.8813	27.8898	26.1493	26.1382	24.7083	27.596	27.9269	27.616	Q1KMD3;H3BQZ7	Q1KMD3;H3BQZ7	Heterogeneous nu	HNRNPUL2;HNRNI
23.2454	23.9067	22.8631	22.967	23.6629	NaN	NaN	24.0109	NaN	NaN	23.2907	NaN	Q9NSC5;M0R2U7	Q9NSC5;M0R2U7	Homer protein ho	HOMER3
27.5987	27.8737	27.5245	27.5846	27.655	27.5779	28.1358	27.6542	27.2782	26.9523	27.6608	27.7636	Q86VS8;H0YDM4	Q86VS8	Protein Hook hom	HOOK3
24.5718	24.5191	24.3661	25.1856	24.6034	24.6341	24.9025	24.461	23.8863	24.0493	25.731	25.4571	Q5SSJ5;B0QZK4;C	Q5SSJ5;B0QZK4;C	Heterochromatin	HP1BP3
27.7767	25.5357	26.2532	26.57	25.4671	25.6466	26.44	NaN	NaN	26.5875	25.973	24.8784	P37235;P84074;E	P37235;P84074;E	Hippocalcin-like p	HPCAL1;HPCA
27.2782	27.7733	27.6486	26.7879	27.661	27.6862	27.2423	26.8802	26.8542	26.9494	27.0491	26.9616	P00492	P00492	Hypoxanthine-gua	HPRT1
23.0966	NaN	22.0443	22.1164	NaN	NaN	NaN	NaN	NaN	22.3865	22.1343	NaN	Q969F9;Q969F9-2	Q969F9;Q969F9-2	Hermansky-Pudla	HPS3
23.5025	23.3823	23.3253	23.8394	23.691	23.6211	23.4939	NaN	24.4541	23.6371	23.7957	24.1956	Q86YV9	Q86YV9	Hermansky-Pudla	HPS6
26.1762	26.3413	26.9398	26.1891	26.6611	27.2047	26.3345	26.2357	26.6292	27.316	27.1109	27.0435	P01112;P01112-2	P01112;P01112-2	GTPase HRas;GTP	HRAS
23.1792	23.7645	23.6075	23.5353	23.5669	24.4841	NaN	NaN	23.7165	24.7792	NaN	24.4329	P35367	P35367	Histamine H1 rece	HRH1
NaN	NaN	NaN	NaN	NaN	NaN	NaN	NaN	NaN	NaN	NaN	20.7634	P52758	P52758	Ribonuclease UK1	HRSP12
25.3231	24.5621	24.9083	25.0872	24.5413	24.8364	25.3127	NaN	NaN	24.4053	24.1537	NaN	Q7LGA3;Q7LGA3-	Q7LGA3;Q7LGA3-	Heparan sulfate 2	HS2ST1
NaN	NaN	NaN	NaN	NaN	NaN	NaN	21.6952	22.6762	22.3754	22.2378	NaN	O14792	O14792	Heparan sulfate g	HS3ST1
22.1864	NaN	NaN	NaN	NaN	NaN	NaN	NaN	NaN	NaN	NaN	NaN	O75506	O75506	Heat shock factor	HSBP1
27.8711	27.9426	27.7402	28.1482	27.9916	27.794	26.5219	27.2327	27.9928	27.4313	27.822	27.5123	Q99714;Q99714-2	Q99714;Q99714-2	3-hydroxyacyl-Co	HSD17B10
25.1759	24.543	24.8902	25.5405	24.493	25.2684	25.0478	24.9604	25.3184	25.4478	24.5296	24.9445	Q8NBQ5;D6RCD0	Q8NBQ5;D6RCD0	Estradiol 17-beta-	HSD17B11
26.9665	26.1588	26.8061	27.206	26.3319	26.9729	27.3283	27.3209	27.4485	26.9855	26.7758	26.5626	Q53GQ0;E9PI21;C	Q53GQ0	Very-long-chain 3	HSD17B12
26.5117	26.8181	26.7706	26.6013	26.6092	26.823	26.4426	27.3496	27.4147	26.5545	26.5588	26.6327	P51659;E7EWE5;F	P51659;E7EWE5;F	Peroxisomal multi	HSD17B4
NaN	NaN	NaN	NaN	NaN	NaN	NaN	NaN	23.6069	NaN	NaN	NaN	Q3SXM5;Q3SXM5	Q3SXM5;Q3SXM5	Inactive hydroxys	HSDL1
26.4428	25.796	25.5935	26.1478	25.3759	24.396	NaN	24.6575	25.4429	25.5211	25.2714	25.4488	Q6YN16;Q6YN16-	Q6YN16;Q6YN16-	Hydroxysteroid de	HSDL2
32.2114	32.0208	32.3618	32.2198	32.0745	32.3035	32.6621	32.8322	32.1316	32.5035	32.6432	32.5461	P07900;P07900-2	P07900;P07900-2	Heat shock protei	HSP90AA1
34.3661	33.5502	34.0115	34.0155	33.6973	34.0119	34.1063	34.2103	33.8455	34.2432	34.2853	34.0462	P08238;Q58FF7	P08238	Heat shock protei	HSP90AB1
27.3767	27.2651	27.8892	28.1619	27.2574	27.9479	28.4609	28.2374	27.6888	27.9341	28.0681	27.7642	Q58FF8	Q58FF8	Putative heat sho	HSP90AB2P
31.9758	31.7188	32.0932	32.0087	31.82	32.0919	31.9646	32.6177	32.7686	31.6977	31.8225	31.9866	P14625;Q96GW1;	P14625	Endoplasmin	HSP90B1
24.7676	25.2304	24.9239	24.6995	24.969	25.0783	26.1757	25.8077	25.3058	25.0534	25.0113	24.7661	P48723	P48723	Heat shock 70 kDa	HSPA13
25.4422	24.739	24.912	25.8222	25.3625	25.1416	23.237	NaN	NaN	24.1541	24.5009	NaN	Q0VDF9;H7C2A1;	Q0VDF9;H7C2A1	Heat shock 70 kDa	HSPA14
31.5296	32.0536	31.4006	30.9814	31.6379	31.3659	31.9287	31.8999	32.1774	31.7302	31.7157	32.2158	P0DMV8;A0A0G2	P0DMV8;A0A0G2	Heat shock 70 kDa	HSPA1A;HSPA1B
NaN	21.8622	21.7384	NaN	NaN	NaN	NaN	NaN	NaN	NaN	NaN	NaN	P54652	P54652	Heat shock-relate	HSPA2
30.3126	30.3439	30.1426	30.0804	30.1119	30.1565	30.658	30.5918	30.291	30.6872	30.5955	30.5138	P34932;A0A087W	P34932;A0A087W	Heat shock 70 kDa	HSPA4
27.6662	27.4198	27.5345	27.8062	27.6331	27.7434	27.1707	27.8528	27.4031	27.3789	27.4783	27.7362	O95757;E9PDE8;D	O95757;E9PDE8;D	Heat shock 70 kDa	HSPA4L
32.5026	33.0611	32.5199	32.3034	32.8263	32.5679	32.0789	32.9021	32.917	31.883	31.9504	32.0875	P11021	P11021	78 kDa glucose-re	HSPA5
NaN	30.0104	29.4552	28.4975	29.7483	29.6688	29.4122	30.0251	28.9686	29.0967	29.9887	29.8602	P17066;P48741	P17066;P48741	Heat shock 70 kDa	HSPA6;HSPA7

25.0365	25.3372	25.0416	24.6751	25.5286	25.1427	26.9691	27.0558	25.8012	27.0633	26.8792	26.9837	E9PN89	E9PN89		HSPA8
33.9135	34.6543	34.2368	33.8406	34.5723	34.3992	34.6002	34.5669	34.4108	34.6788	34.7128	34.8511	P11142;E9PKE3;P	P11142;E9PKE3;P	Heat shock cogna	HSPA8
31.0356	31.8081	31.0142	31.0739	31.8648	31.1229	30.8364	31.6918	31.9621	31.3168	31.4303	31.6473	P38646;D6RJI2;D6	P38646	Stress-70 protein,	HSPA9
28.071	28.2711	27.9675	28.2597	28.4298	28.0634	30.0204	30.246	29.7128	29.9347	30.1266	29.8808	P04792;F8WE04;C	P04792;F8WE04	Heat shock protei	HSPB1
25.8099	25.733	25.9133	25.7738	25.6099	25.8784	25.4144	NaN	25.6929	25.7898	NaN	NaN	A6NIR2;X6R7Y7	A6NIR2;X6R7Y7		HSPB11
25.5657	23.6357	25.1848	25.1898	24.6668	24.956	24.7442	24.6048	25.0504	25.2636	26.0107	NaN	Q9NZL4;Q9NZL4-3	Q9NZL4;Q9NZL4-3	Hsp70-binding pro	HSPBP1
31.1322	32.4163	31.6214	31.6873	32.4668	31.6292	31.2126	32.7553	33.0699	30.1388	31.0429	31.5333	P10809;E7EXB4;E	P10809	60 kDa heat shock	HSPD1
29.1067	30.5445	29.5586	29.265	30.1131	29.5302	30.0561	30.7721	30.7916	29.4357	30.0227	30.1343	P61604;B8ZZL8;B8	P61604;B8ZZL8	10 kDa heat shock	HSPE1
24.9966	25.5864	24.8977	24.8424	25.6114	25.0307	25.1848	26.0037	NaN	25.5629	25.7419	25.419	S4R3N1;B4DM50;	S4R3N1		HSPE1-MOB4
23.0716	23.2257	24.6039	22.4655	23.8968	22.7419	28.7755	27.8869	NaN	22.4212	NaN	23.8209	P98160;H7BYA5;A	P98160	Basement membr	HSPG2
30.2226	29.6081	29.6954	29.9535	29.5724	29.5694	30.0734	30.0977	29.9825	30.1828	29.8307	29.9877	Q92598;Q92598-2	Q92598;Q92598-2	Heat shock protei	HSPH1
24.2242	25.2443	25.0549	24.4598	25.2427	24.8981	24.268	NaN	24.9071	24.7982	25.0355	25.7548	Q9BUP3;Q9BUP3-	Q9BUP3;Q9BUP3-	Oxidoreductase H	HTATIP2
24.5326	24.1132	24.806	24.7276	24.8528	25.2532	NaN	NaN	NaN	23.5669	23.9425	NaN	O43719;Q5H919;(	O43719;Q5H919;(	HIV Tat-specific fa	HTATSF1
NaN	NaN	NaN	NaN	NaN	NaN	24.8101	24.4643	NaN	23.4368	24.1402	23.5982	Q92743;H0Y7G9	Q92743	Serine protease H	HTRA1
23.2243	23.4385	22.8646	23.2011	23.4169	NaN	NaN	NaN	NaN	NaN	23.443	23.261	O43464;A0A0C4D	O43464;A0A0C4D	Serine protease H	HTRA2
22.433	NaN	22.6988	22.9738	NaN	NaN	NaN	NaN	NaN	22.8231	22.4551	NaN	P42858	P42858	Huntingtin	HTT
22.1151	NaN	NaN	NaN	NaN	NaN	NaN	NaN	NaN	NaN	NaN	NaN	O60921;O60921-2	O60921;O60921-2	Checkpoint protei	HUS1
28.2521	27.1344	28.0247	28.3125	27.5904	27.9952	27.0926	27.1131	26.6964	27.5522	27.5219	26.7032	Q7Z6Z7;Q7Z6Z7-2	Q7Z6Z7;Q7Z6Z7-2	E3 ubiquitin-prote	HUWE1
22.9969	NaN	NaN	NaN	NaN	NaN	NaN	NaN	NaN	NaN	NaN	NaN	Q96D96;F8VPF7;C	Q96D96;F8VPF7;C	Voltage-gated hyd	HVCN1
23.5424	23.3244	23.8203	23.4331	23.4411	NaN	NaN	NaN	NaN	24.0248	24.3764	NaN	Q12891	Q12891	Hyaluronidase-2	HYAL2
25.2311	25.9497	24.991	25.0052	25.4705	NaN	26.158	26.4414	26.6461	25.9958	25.6153	26.7927	Q5T013;F6UJY1;J9	Q5T013;F6UJY1;J9	Putative hydroxyp	HYI
30.6399	30.451	30.1972	30.4881	30.2438	30.1725	29.8698	30.6624	30.5114	29.8779	29.6992	29.7154	Q9Y4L1;A0A087X(	Q9Y4L1;A0A087X(	Hypoxia up-regula	HYOU1
NaN	23.4367	NaN	NaN	NaN	NaN	23.1126	NaN	NaN	NaN	NaN	NaN	Q9NX55	Q9NX55	Huntingtin-interac	HYPK
23.1865	23.8532	23.5405	22.8039	23.7339	23.1871	NaN	NaN	NaN	23.5722	23.3719	NaN	Q2TAA2;H7C5G1;	Q2TAA2;H7C5G1;	Isoamyl acetate-h	IAH1
30.0413	28.9114	29.246	29.8129	29.3008	29.4239	28.7717	29.2929	28.8881	29.5498	29.3714	28.9013	P41252;A0A0A0M	P41252;A0A0A0M	Isoleucine--tRNA l	IARS
25.9392	25.3549	25.2282	25.6456	25.5113	25.2455	24.7314	24.3766	26.0284	25.2676	25.5645	25.312	Q9NSE4	Q9NSE4	Isoleucine--tRNA l	IARS2
NaN	NaN	20.5858	20.8746	NaN	NaN	NaN	NaN	NaN	21.0184	21.4178	NaN	Q5T440	Q5T440	Putative transfera	IBA57
28.677	29.3875	29.5002	28.9422	29.2529	29.7889	27.9347	26.8483	27.9957	28.1661	27.5632	27.9863	P05362;K7EKL8;E	P05362	Intercellular adhe	ICAM1
22.6152	NaN	NaN	NaN	NaN	NaN	NaN	NaN	NaN	23.3441	NaN	NaN	P13598;J3QKR4;J3	P13598;J3QKR4;J3	Intercellular adhe	ICAM2
22.3762	NaN	21.8926	22.7231	NaN	NaN	NaN	NaN	NaN	NaN	NaN	NaN	Q14197;J3KS15	Q14197;J3KS15	Peptidyl-tRNA hyd	ICT1
25.6453	25.9644	26.0212	25.6149	25.9937	25.7387	26.2019	26.4424	25.9077	26.0258	26.5238	26.6129	P14735;P14735-2	P14735;P14735-2	Insulin-degrading	IDE
27.7659	28.3964	28.068	27.5128	27.9935	28.0044	28.7691	28.364	28.1774	28.4605	28.3971	28.1135	O75874;C9J4N6;C	O75874	Isocitrate dehydro	IDH1
27.7448	27.4934	26.9923	27.699	27.4315	27.0589	25.763	26.1717	27.0611	25.9088	26.432	26.3946	P48735;P48735-2	P48735;P48735-2	Isocitrate dehydro	IDH2
27.6854	27.1817	26.8423	27.4834	27.7349	27.45	26.0871	27.2427	27.3583	27.3677	27.6605	26.7971	P50213;H0YL72;P	P50213;H0YL72;P	Isocitrate dehydro	IDH3A
27.6378	27.2533	26.8617	27.7154	27.3532	27.0198	26.1591	25.5714	27.3668	27.3856	27.1996	26.6589	O43837;A0A087W	O43837;A0A087W	Isocitrate dehydro	IDH3B
26.0364	25.7054	25.9316	25.8017	26.2223	26.3349	NaN	23.6678	25.2177	25.4031	24.8013	23.9712	P51553;P51553-2	P51553;P51553-2	Isocitrate dehydro	IDH3G
24.0532	24.0781	24.2895	24.0132	23.4851	23.8991	24.6566	24.9338	24.5633	24.7133	24.3778	24.1992	Q13907;Q13907-2	Q13907;Q13907-2	Isopentenyl-diphc	IDI1
NaN	NaN	NaN	NaN	NaN	NaN	NaN	NaN	21.7167	NaN	NaN	NaN	P22304;P22304-3	P22304;P22304-3	Iduronate 2-sulfat	IDS
NaN	NaN	NaN	21.1554	NaN	NaN	NaN	NaN	NaN	NaN	NaN	NaN	Q9Y5U9	Q9Y5U9	Immediate early r	IER3IP1
27.3018	26.8837	26.7338	27.6939	27.1365	26.8541	25.7187	25.9838	25.9776	26.9417	27.2525	26.9745	Q16666;Q16666-3	Q16666;Q16666-3	Gamma-interfero	IFI16
NaN	NaN	NaN	NaN	NaN	NaN	25.555	NaN	NaN	NaN	NaN	NaN	P13284;M0QZG3	P13284;M0QZG3	Gamma-interfero	IFI30
23.8656	24.4969	23.8849	23.5302	24.4403	23.4606	23.9845	NaN	23.6079	23.7987	23.9353	23.4382	P80217;P80217-2	P80217;P80217-2	Interferon-induce	IFI35
NaN	NaN	NaN	18.8414	NaN	NaN	NaN	NaN	NaN	NaN	NaN	NaN	Q9BYX4;Q9BYX4-2	Q9BYX4;Q9BYX4-2	Interferon-induce	IFIH1
26.8406	26.059	26.3325	26.6154	26.1376	26.3695	27.1663	26.699	26.796	26.7345	26.4729	26.3076	P09914;P09914-2	P09914;P09914-2	Interferon-induce	IFIT1
26.9848	26.2994	26.4247	26.5042	26.3021	26.3673	26.6186	25.3052	26.7766	25.6722	25.683	25.9813	P09913;A0A087X2	P09913;A0A087X2	Interferon-induce	IFIT2
26.4987	26.423	26.4482	26.326	26.0396	26.2725	27.0704	26.4758	26.8397	25.9738	25.8742	26.2364	O14879	O14879	Interferon-induce	IFIT3
25.4892	24.5764	25.1079	25.3497	24.8287	24.7664	24.0801	NaN	NaN	24.7796	24.5656	24.3687	Q13325;Q13325-2	Q13325;Q13325-2	Interferon-induce	IFIT5
25.807	26.0621	26.3685	25.6045	25.6467	26.1884	NaN	NaN	NaN	24.6929	NaN	NaN	P13164;Q01629;C	P13164;Q01629;C	Interferon-induce	IFITM1;IFITM2;IFIT
23.5053	22.4919	23.328	23.3088	22.972	NaN	NaN	NaN	NaN	23.1099	NaN	NaN	P15260;Q5TFC9;F	P15260;Q5TFC9;F	Interferon gamma	IFNGR1
23.2227	22.3663	22.5236	23.2117	22.5048	22.5712	NaN	NaN	NaN	22.2862	NaN	NaN	Q12894;A0A0R4J2	Q12894;A0A0R4J2	Interferon-relatec	IFRD2
23.4936	23.2464	23.8575	23.3382	24.2469	23.1031	23.1802	NaN	NaN	22.5646	22.9061	23.65	Q9BW83;Q9BW83	Q9BW83;Q9BW83	Intraflagellar trans	IFT27
NaN	NaN	23.9774	NaN	NaN	NaN	NaN	NaN	NaN	NaN	NaN	NaN	Q96LB3;Q96LB3-2	Q96LB3	Intraflagellar trans	IFT74
24.1471	24.7315	24.5161	24.4527	24.5624	24.6839	NaN	25.1683	NaN	24.9049	24.4851	24.4903	P78318	P78318	Immunoglobulin-l	IGBP1
26.5132	26.36	27.2181	26.8213	26.2217	27.3646	26.3426	25.3724	25.8267	27.5325	27.0821	27.0858	P08069;C9J5X1;H	P08069;C9J5X1	Insulin-like growtl	IGF1R
NaN	NaN	NaN	NaN	NaN	NaN	24.6132	25.4821	25.1752	NaN	23.3826	NaN	P01344;P01344-3	P01344;P01344-3	Insulin-like growtl	IGF2
28.0885	27.6624	27.4548	28.0264	27.806	27.5799	25.8034	26.6455	26.2097	26.0704	26.1907	26.0162	Q9Y6M1;F8W930	Q9Y6M1;F8W930	Insulin-like growtl	IGF2BP2
27.3306	25.9284	25.9793	27.3716	26.6167	25.9375	23.6524	24.3799	23.5694	24.7368	24.6422	23.9515	O00425;O00425-2	O00425	Insulin-like growtl	IGF2BP3
27.5995	27.4969	27.3577	27.7707	27.1147	27.6243	27.7226	26.8361	26.8638	27.2248	26.8863	26.2484	P11717;S4R328	P11717	Cation-independe	IGF2R
23.1115	23.8125	NaN	22.4845	22.9251	NaN	28.9703	28.1133	24.6989	23.702	24.2148	26.0689	P08833;C9JXF9;C9	P08833;C9JXF9;C9	Insulin-like growtl	IGFBP1
NaN	23.8573	NaN	NaN	22.2119	NaN	26.8424	28.9593	28.1262	23.1582	24.3374	24.5214	P17936;H0Y485;H	P17936;H0Y485;H	Insulin-like growtl	IGFBP3

25.1934	26.7036	25.9568	25.9609	25.5683	NaN	28.8343	28.0097	27.5591	NaN	NaN	24.3748	P22692;P22692-2	P22692;P22692-2	Insulin-like growtl	IGFBP4
NaN	NaN	NaN	NaN	NaN	NaN	23.8042	NaN	NaN	NaN	NaN	NaN	P24592;F8VYK9	P24592;F8VYK9	Insulin-like growtl	IGFBP6
24.6425	26.6657	25.3176	23.7661	26.0429	25.6664	28.1604	28.7838	27.9371	25.6594	25.6345	NaN	Q16270;Q16270-2	Q16270;Q16270-2	Insulin-like growtl	IGFBP7
NaN	NaN	NaN	NaN	NaN	NaN	NaN	23.4799	23.2009	NaN	NaN	NaN	Q8WX77	Q8WX77	Insulin-like growtl	IGFBPL1
22.9257	NaN	22.7531	22.9735	NaN	NaN	NaN	NaN	NaN	NaN	22.916	NaN	P38935	P38935	DNA-binding prot	IGHMBP2
23.9301	23.5483	23.6912	23.8197	NaN	23.7961	NaN	NaN	NaN	NaN	NaN	NaN	O75054;O75054-2	O75054;O75054-2	Immunoglobulin s	IGSF3
26.3533	26.8109	27.1152	26.0551	26.6146	26.9767	27.4148	27.3214	28.1952	26.4843	26.3695	27.3307	Q969P0;Q969P0-3	Q969P0;Q969P0-3	Immunoglobulin s	IGSF8
24.7164	23.7807	24.1611	24.4285	24.2495	24.5369	NaN	NaN	NaN	23.5364	NaN	NaN	Q13123;Q9H4P6;I	Q13123;Q9H4P6;I	Protein Red	IK
27.6441	28.2425	27.8009	27.8533	27.8199	27.8073	27.5639	28.4344	28.4572	27.4054	27.2156	27.463	Q70UQ0;Q70UQ0	Q70UQ0;Q70UQ0	Inhibitor of nuclea	IKBIP
27.1785	27.4761	27.1098	27.1977	27.0202	27.1156	26.8597	27.421	27.6759	27.1346	26.6133	26.6709	Q70UQ0-4	Q70UQ0-4	Inhibitor of nuclea	IKBIP
27.3621	25.408	25.9206	27.0756	25.4155	26.091	25.2697	25.3537	23.412	26.3404	25.8833	24.3158	O95163;F5H2T0;H	O95163;F5H2T0	Elongator comple	IKBKAP
26.2464	24.3137	25.1351	26.1525	24.8579	24.9991	23.269	NaN	NaN	25.1375	24.6649	NaN	O14920;O14920-2	O14920;O14920-2	Inhibitor of nuclea	IKBKB
24.1942	23.867	23.699	24.0098	25.0372	24.1006	NaN	24.7428	24.0043	23.142	23.8696	NaN	Q9Y6K9;A0A087X	Q9Y6K9;A0A087X	NF-kappa-B essen	IKBKG
NaN	NaN	NaN	23.4165	NaN	NaN	NaN	NaN	NaN	NaN	NaN	NaN	C9J2V2	C9J2V2		IKBKG
NaN	NaN	NaN	NaN	NaN	NaN	NaN	NaN	NaN	22.6688	NaN	NaN	Q08334;H0Y3Z8;F	Q08334;H0Y3Z8;F	Interleukin-10 rec	IL10RB
NaN	NaN	NaN	NaN	NaN	NaN	23.8704	NaN	NaN	NaN	NaN	NaN	P20809-2;K7ESD5	P20809-2;K7ESD5	Interleukin-11	IL11
27.2847	27.4166	26.8577	27.1378	27.156	26.718	25.694	NaN	NaN	26.1088	25.3673	24.9875	Q14116;Q14116-2	Q14116;Q14116-2	Interleukin-18	IL18
24.0068	NaN	NaN	NaN	NaN	NaN	NaN	NaN	NaN	NaN	NaN	NaN	P01584;C9JWV2;C	P01584;C9JWV2;C	Interleukin-1 beta	IL1B
24.4312	24.311	25.6198	25.1388	24.5144	25.5716	23.6426	NaN	24.1548	25.1505	NaN	24.9928	Q9NPH3;Q9NPH3	Q9NPH3;Q9NPH3	Interleukin-1 rece	IL1RAP
24.8185	25.119	24.5157	24.4122	24.1316	24.2187	25.3579	25.2967	25.8131	23.8006	23.83	24.8627	P40189;P40189-3	P40189;P40189-3	Interleukin-6 rece	IL6ST
28.5264	27.1564	27.7224	28.478	27.4512	27.9005	26.6988	26.764	26.5013	27.1634	27.6742	26.758	Q12905;B4DY09;K	Q12905;B4DY09;K	Interleukin enhan	ILF2
29.7735	28.8949	29.077	30.0859	29.3504	29.5778	27.6755	28.4799	28.2948	28.6043	29.4177	29.2927	Q12906-7;Q12906	Q12906-7;Q12906	Interleukin enhan	ILF3
28.2997	27.8399	28.2611	28.3062	28.1119	28.4167	28.1915	27.9935	28.2173	28.4441	28.4694	28.3117	Q13418;A0A0A0N	Q13418;A0A0A0N	Integrin-linked pro	ILK
24.9551	24.5063	24.6721	25.0197	24.9075	24.4158	23.6565	24.4432	NaN	25.0996	25.2747	24.7355	Q9H0C8;F8SNU7;I	Q9H0C8;F8SNU7	Integrin-linked kir	ILKAP;ILKAP3
25.049	24.7675	25.022	25.3667	24.8394	25.1771	24.9087	NaN	NaN	25.3469	25.074	24.5884	A1L0T0;M0R026;E	A1L0T0;M0R026	Acetolactate synt	ILVBL
26.1332	26.8037	26.4538	26.3376	27.1004	26.4209	26.4366	28.0305	28.0076	26.3264	26.8796	26.3372	Q16891;Q16891-2	Q16891;Q16891-2	MICOS complex su	IMMT
23.5151	21.9525	22.4336	23.5984	22.5635	22.1683	NaN	NaN	NaN	21.823	22.0553	NaN	Q9NV31	Q9NV31	U3 small nucleola	IMP3
24.3796	23.5747	23.1767	24.2491	23.4681	23.2928	NaN	NaN	NaN	NaN	23.2402	NaN	Q96G21;B9A008;E	Q96G21;B9A008;E	U3 small nucleola	IMP4
25.6236	26.8844	26.1505	25.6253	26.4835	26.0943	26.5345	26.1111	25.9906	26.2692	26.4387	26.7119	P29218;P29218-3	P29218;P29218-3	Inositol monopho	IMPA1
22.7542	23.5692	23.0489	21.6303	24.3228	NaN	26.693	26.0305	22.9134	23.0951	NaN	21.8166	Q9NX62;H0YBS3	Q9NX62;H0YBS3	Inositol monopho	IMPAD1
26.2552	26.6183	26.1635	26.691	26.866	25.9438	26.44	27.0273	26.682	26.4984	26.6428	26.7344	P20839;P20839-6	P20839;P20839-6	Inosine-5-monoph	IMPDH1
31.7334	32.0337	31.6722	32.5471	32.27	31.8403	31.2287	32.0736	31.582	31.6274	31.7865	31.5888	P12268;H0Y4R1;E	P12268;H0Y4R1	Inosine-5-monoph	IMPDH2
NaN	NaN	NaN	NaN	NaN	NaN	24.6359	NaN	NaN	NaN	NaN	NaN	Q9GZP8;Q9GZP8-	Q9GZP8;Q9GZP8-	Immortalization u	IMUP
25.6948	26.0616	26.4081	25.1457	25.3225	25.4472	28.3224	27.3159	27.2179	25.3664	25.1077	25.2834	Q9NQS7;Q9NQS7	Q9NQS7;Q9NQS7	Inner centromere	INCENP
27.4596	26.4125	27.4095	26.8846	25.6527	26.8127	26.856	25.8723	27.165	25.955	25.6711	25.8047	Q27J81;Q27J81-2	Q27J81;Q27J81-2	Inverted formin-2	INF2
21.3003	NaN	NaN	NaN	NaN	NaN	NaN	NaN	NaN	NaN	NaN	NaN	O15327;E9PHC0;E	O15327;E9PHC0;E	Type II inositol 3,4	INPP4B
23.047	23.208	24.0518	23.3991	NaN	NaN	24.2707	NaN	NaN	25.28	NaN	NaN	Q14642;Q5T1B5;H	Q14642;Q5T1B5;H	Type I inositol 1,4,	INPP5A
24.801	24.3247	24.5057	24.1657	24.3272	24.3778	23.4555	24.8257	24.9365	24.1627	23.5844	NaN	Q9Y2H2;Q9Y2H2-	Q9Y2H2;Q9Y2H2-	Phosphatidylinosi	INPP5F
22.492	NaN	22.5556	22.9698	21.7943	NaN	NaN	NaN	NaN	21.7585	NaN	NaN	Q9BT40;Q9BT40-2	Q9BT40;Q9BT40-2	Inositol polyphosp	INPP5K
23.9083	23.4028	24.1108	23.7985	23.6881	24.0372	24.2399	24.3094	24.4778	24.0386	23.946	24.2754	O15357;A0A0A0N	O15357;A0A0A0N	Phosphatidylinosi	INPPL1
23.9829	24.2782	25.3875	24.9452	24.3032	25.3875	25.0219	NaN	24.9228	25.4684	24.6779	24.8057	P06213;P06213-2	P06213;P06213-2	Insulin receptor;Ir	INSR
21.5147	NaN	21.9268	22.1673	NaN	NaN	NaN	NaN	NaN	21.7585	21.9025	NaN	Q8N201	Q8N201	Integrator comple	INTS1
24.0315	NaN	23.0111	23.6513	22.7326	23.1145	NaN	NaN	NaN	23.0996	NaN	NaN	Q9H0H0;J3KMZ7	Q9H0H0;J3KMZ7	Integrator comple	INTS2
23.7598	NaN	22.799	23.5652	23.2659	23.0077	NaN	NaN	NaN	NaN	NaN	NaN	Q68E01;Q68E01-2	Q68E01;Q68E01-2	Integrator comple	INTS3
22.3448	NaN	22.5325	22.8656	NaN	NaN	NaN	NaN	NaN	22.2223	NaN	NaN	Q96HW7;Q96HW	Q96HW7;Q96HW	Integrator comple	INTS4
25.7774	NaN	26.4299	26.5823	26.2659	NaN	26.296	NaN	NaN	26.5282	26.2105	NaN	Q6P9B9	Q6P9B9	Integrator comple	INTS5
21.649	NaN	NaN	21.0629	21.03	NaN	NaN	NaN	NaN	NaN	NaN	NaN	Q9UL03;C9JVX2;C	Q9UL03;C9JVX2;C	Integrator comple	INTS6
27.0891	NaN	26.171	26.5227	NaN	NaN	27.144	NaN	NaN	26.3612	26.3205	NaN	Q9NVH2;Q9NVH2	Q9NVH2;Q9NVH2	Integrator comple	INTS7
NaN	NaN	NaN	NaN	NaN	NaN	NaN	21.7019	NaN	NaN	NaN	NaN	Q9NV88;H0YBH8;	Q9NV88;H0YBH8;	Integrator comple	INTS9
25.5964	24.4289	25.3003	25.4944	25.1073	25.2898	25.2325	24.8069	24.6889	25.7826	25.4847	25.3167	Q9UI26;Q9UI26-2	Q9UI26;Q9UI26-2	Importin-11	IPO11
NaN	NaN	NaN	NaN	NaN	NaN	NaN	NaN	NaN	20.3683	NaN	NaN	O94829;Q5T4X2	O94829;Q5T4X2	Importin-13	IPO13
27.0408	26.0645	26.7048	26.9986	26.0845	26.6309	26.9717	26.8735	27.0076	26.9995	26.6725	27.046	Q8TEX9;Q8TEX9-2	Q8TEX9;Q8TEX9-2	Importin-4	IPO4
29.1787	28.7936	29.2539	28.8975	28.6268	29.2098	29.6347	29.3769	29.1222	29.4063	29.1432	29.2613	O00410;O00410-3	O00410;O00410-3	Importin-5	IPO5
29.3232	28.1504	28.7172	28.7676	28.0998	28.7591	28.9502	28.5072	28.0359	28.5761	28.1396	28.1263	O95373;E9PLJ0;E	O95373	Importin-7	IPO7
23.9512	23.0451	23.3355	23.7287	22.9751	23.3769	23.2905	NaN	NaN	24.2356	23.9031	23.1909	O15397;O15397-2	O15397;O15397-2	Importin-8	IPO8
26.8879	26.2146	26.6584	26.472	25.9956	26.7659	27.2728	27.0871	27.4252	27.1914	26.6734	27.0169	Q96P70	Q96P70	Importin-9	IPO9
31.8335	32.0365	32.5965	32.0927	32.1885	32.8349	32.5853	31.603	31.7571	32.8407	32.5798	32.4691	P46940;H0YLE8;A	P46940;H0YLE8;A	Ras GTPase-activa	IQGAP1
27.9475	27.6183	28.6211	28.4207	27.8823	28.8698	29.238	28.5614	28.4321	28.66	28.3546	28.3727	Q86VI3;F2Z2E2;E	Q86VI3;F2Z2E2	Ras GTPase-activa	IQGAP3
22.8615	NaN	22.8128	22.6841	NaN	NaN	NaN	NaN	NaN	22.8706	23.1924	NaN	Q6DN90;A0A0C4E	Q6DN90;A0A0C4E	IQ motif and SEC7	IQSEC1
23.3806	NaN	23.3915	23.0066	NaN	NaN	NaN	NaN	NaN	22.7283	23.2032	NaN	P51617;P51617-4	P51617;P51617-4	Interleukin-1 rece	IRAK1

NaN	NaN	NaN	22.0454	NaN	NaN	NaN	NaN	NaN	NaN	NaN	NaN	Q9NWZ3;A0A0R4	Q9NWZ3;A0A0R4	Interleukin-1 rece	IRAK4
24.9128	24.6853	24.4209	24.6794	24.7628	24.6003	24.4414	24.1968	NaN	24.001	NaN	NaN	P48200;A0A0A6Y	P48200;A0A0A6Y	Iron-responsive el	IREB2
22.6288	22.5102	22.6771	22.8084	23.0244	NaN	NaN	NaN	NaN	NaN	22.9282	NaN	Q8IU81	Q8IU81	Interferon regulat	IRF2BP1
23.334	23.3953	23.5838	23.067	23.3899	23.6167	23.3546	NaN	NaN	23.6908	23.7127	NaN	Q9H1B7	Q9H1B7	Interferon regulat	IRF2BPL
22.5598	NaN	NaN	22.2485	NaN	NaN	NaN	NaN	NaN	NaN	NaN	NaN	Q14653;M0QYT9;	Q14653;M0QYT9;	Interferon regulat	IRF3
24.8743	25.5607	25.2883	24.3625	25.5203	25.4927	26.8589	25.8036	25.6857	25.4895	24.8147	26.082	Q8WZA9;M0QZP8	Q8WZA9	Immunity-related	IRGQ
23.4069	23.2147	23.2728	23.3193	22.5959	23.2324	NaN	NaN	NaN	NaN	22.6838	NaN	Q9Y4H2	Q9Y4H2	Insulin receptor su	IRS2
27.2072	26.962	27.3344	27.0276	26.8934	27.3947	28.0234	26.9304	27.3997	27.9243	27.448	26.7131	P05161;A0A096LI	P05161;A0A096LI	Ubiquitin-like prot	ISG15
24.6713	25.2858	24.4786	24.0349	24.7129	24.3657	24.8489	24.5316	24.9809	24.6024	24.016	24.4111	Q96AZ6;H0YMM4	Q96AZ6;H0YMM4	Interferon-stimula	ISG20
24.3108	24.8404	24.2185	24.1355	24.6012	24.5065	23.7503	23.9786	NaN	23.8459	24.0951	24.4764	Q96CN7;D6RGE2	Q96CN7;D6RGE2	Isochorismatase c	ISOC1
24.2076	NaN	23.6777	23.4737	24.3462	NaN	22.5294	NaN	NaN	23.0538	NaN	NaN	Q96AB3;Q96AB3-	Q96AB3;Q96AB3-	Isochorismatase c	ISOC2
28.0354	28.3174	28.3391	27.563	27.4532	28.1848	29.2088	28.4979	29.0564	28.1547	27.7242	28.9358	P53990;P53990-2	P53990;P53990-2	IST1 homolog	IST1
23.3545	22.6472	22.999	23.3795	22.5818	23.4855	NaN	NaN	NaN	NaN	23.9562	NaN	Q9ULR0-1;Q9ULR	Q9ULR0-1;Q9ULR	Pre-mRNA-splicing	ISY1
NaN	24.6205	25.0634	24.2013	24.9793	NaN	NaN	NaN	NaN	NaN	NaN	NaN	Q9NPH2;Q9NPH2	Q9NPH2;Q9NPH2	Inositol-3-phosph	ISYNA1
27.0324	26.4585	26.7811	26.4546	25.9683	26.3364	26.7821	25.635	26.5083	26.7613	26.101	26.2747	Q96J02;Q96J02-2	Q96J02;Q96J02-2	E3 ubiquitin-prote	ITCH
21.6977	NaN	22.5698	22.1124	22.3632	NaN	NaN	NaN	NaN	22.1271	NaN	NaN	Q8TB96;H3BUJ1;F	Q8TB96;H3BUJ1;F	T-cell immunomo	ITFG1
25.17	25.354	26.265	25.4924	25.2514	26.3253	25.8829	25.0417	26.0923	25.9868	25.6145	26.0059	Q9H0X4;Q9H0X4-	Q9H0X4;Q9H0X4-	Protein ITFG3	ITFG3
26.2429	26.577	27.3843	26.4669	26.4266	27.8375	26.0191	NaN	27.0134	26.3168	26.0022	26.7637	P56199	P56199	Integrin alpha-1	ITGA1
27.6265	26.7366	27.8662	27.0506	26.9243	28.0671	27.2054	27.2403	28.1543	27.4048	27.4007	28.2815	E7ESP4;E7EMF1;E	E7ESP4;E7EMF1;E9PB77		ITGA2
31.6525	32.1322	32.7662	32.0573	32.3957	32.9257	32.3633	32.0707	33.1788	32.8388	32.5723	33.2272	P17301	P17301	Integrin alpha-2	ITGA2
31.3557	32.0169	32.4554	31.6611	31.5941	32.5065	31.6238	31.271	31.8288	31.7985	31.6735	31.7458	P26006;P26006-1	P26006;P26006-1	Integrin alpha-3;In	ITGA3
27.5673	28.1514	28.185	28.025	27.8757	28.2034	27.8126	27.3901	28.2577	28.1355	27.5544	27.9132	P08648;H0YIV7	P08648	Integrin alpha-5;In	ITGA5
29.8112	30.0496	30.2805	29.7978	29.5447	30.3048	28.5181	27.3997	29.017	28.6609	28.2422	28.5949	P23229-2;P23229	P23229-2;P23229	Integrin alpha-6;In	ITGA6
27.7141	28.1919	28.5719	27.7724	27.9669	28.5935	27.7506	27.0396	28.0329	28.2681	27.4558	27.4751	P06756;P06756-3	P06756;P06756-3	Integrin alpha-V;In	ITGAV
32.0645	32.1765	32.6102	31.9718	32.0042	32.6548	31.9472	31.9052	32.8821	32.1615	32.0351	32.7906	P05556;P05556-2	P05556;P05556-2	Integrin beta-1	ITGB1
24.6795	25.5453	25.8069	24.7047	25.2648	25.9447	24.3887	NaN	25.7805	24.7857	25.0644	25.895	P05106;H3BM21;	P05106;H3BM21;	Integrin beta-3;In	ITGB3
29.908	30.3918	30.8416	29.9848	29.8815	30.9866	26.5332	24.0789	28.6439	27.44	25.766	25.5126	P16144-2;P16144	P16144-2;P16144	Integrin beta-4	ITGB4
25.3506	25.5737	26.971	26.2569	25.7991	27.3251	25.6676	24.9664	25.1081	26.6472	25.6382	25.627	P18084;H7C4W1;	P18084;H7C4W1	Integrin beta-5;In	ITGB5
NaN	NaN	NaN	NaN	NaN	NaN	20.7198	NaN	NaN	NaN	NaN	NaN	Q8WWU7;Q8WW	Q8WWU7;Q8WW	Intelectin-2	ITLN2
23.5666	24.0838	24.3192	23.7596	23.8023	24.1587	NaN	NaN	24.7269	23.6359	NaN	NaN	Q9Y287;Q9Y287-2	Q9Y287;Q9Y287-2	Integral membran	ITM2B
22.9542	23.0276	22.7053	22.7025	NaN	NaN	22.8566	NaN	NaN	22.653	NaN	NaN	Q9NQX7;C9JG41;	Q9NQX7;C9JG41;	Integral membran	ITM2C
25.4315	25.3182	24.7431	25.02	25.2601	24.9901	25.3744	NaN	25.7096	26.0506	26.086	NaN	Q9BY32;Q9BY32-3	Q9BY32;Q9BY32-3	Inosine triphospha	ITPA
NaN	NaN	NaN	23.5358	NaN	NaN	NaN	NaN	NaN	NaN	NaN	NaN	Q13572;Q13572-2	Q13572;Q13572-2	Inositol-tetrakispl	ITPK1
25.9844	25.726	25.8725	26.0404	25.738	25.4823	26.0652	25.9134	25.3274	26.3078	25.9852	26.2316	Q14573;Q14643;C	Q14573	Inositol 1,4,5-trisp	ITPR3
NaN	23.0878	22.956	NaN	22.7806	NaN	NaN	NaN	NaN	NaN	NaN	NaN	Q9NZM3;A0A087	Q9NZM3;A0A087	Intersectin-2	ITSN2
27.2448	26.7849	26.6729	27.4805	26.8535	26.7471	24.825	NaN	25.9676	26.8119	26.8092	24.618	P26440;A0A0A0M	P26440;A0A0A0M	Isovaleryl-CoA del	IVD
NaN	NaN	NaN	21.4802	NaN	NaN	NaN	NaN	NaN	NaN	21.7272	NaN	Q96ST2;Q96ST2-2	Q96ST2;Q96ST2-2	Protein IWS1 hom	IWS1
23.386	23.2724	23.3807	NaN	NaN	NaN	NaN	NaN	NaN	24.4595	NaN	NaN	P78504;P78504-2	P78504;P78504-2	Protein jagged-1	JAG1
23.6877	NaN	24.2788	24.3941	NaN	NaN	NaN	NaN	NaN	25.2868	24.8696	23.8078	Q8N5M9;A0A087	Q8N5M9;A0A087	Protein jagunal ho	JAGN1
26.4867	26.9506	27.3464	26.2127	25.8329	26.9035	26.4729	24.4884	26.8555	26.612	25.1651	25.058	P23458;O60674	P23458	Tyrosine-protein k	JAK1
23.4852	NaN	NaN	23.5885	NaN	22.6238	NaN	NaN	NaN	NaN	22.5679	NaN	Q6NYC1;B2WTI3;	Q6NYC1;B2WTI3;	Bifunctional argin	JMJD6
23.3703	NaN	NaN	NaN	NaN	NaN	NaN	NaN	NaN	NaN	NaN	NaN	Q8N9B5;Q8N9B5-	Q8N9B5;Q8N9B5-	Junction-mediatin	JMY
23.3581	NaN	22.3924	23.4253	22.8659	NaN	NaN	NaN	NaN	NaN	NaN	NaN	P05412;P17535	P05412	Transcription facto	JUN
24.513	25.1256	25.747	25.5992	25.5688	26.1488	26.7437	24.9142	25.5875	25.9661	25.401	24.4087	P14923;C9JTX4;C9	P14923	Junction plakoglol	JUP
NaN	NaN	NaN	NaN	NaN	NaN	22.4245	NaN	NaN	NaN	NaN	NaN	P23352;A0A087W	P23352;A0A087W	Anosmin-1	KAL1
20.9613	NaN	NaN	NaN	NaN	NaN	NaN	NaN	NaN	NaN	NaN	NaN	Q63ZY3;Q63ZY3-2	Q63ZY3;Q63ZY3-2	KN motif and anky	KANK2
28.7608	28.5317	28.4421	28.4273	28.5533	28.4321	29.044	29.2163	28.6501	29.1761	29.2295	28.7833	Q15046;Q15046-2	Q15046;Q15046-2	Lysine--tRNA ligas	KARS
NaN	NaN	NaN	22.6251	NaN	NaN	NaN	NaN	NaN	NaN	NaN	NaN	O95251;O95251-2	O95251;O95251-2	Histone acetyltrar	KAT7
23.5642	NaN	NaN	23.9562	NaN	NaN	NaN	NaN	NaN	NaN	NaN	NaN	Q9H7Z6;Q9H7Z6-	Q9H7Z6;Q9H7Z6-	Histone acetyltrar	KAT8
23.3362	23.1279	22.7047	23.0151	23.0394	NaN	NaN	NaN	NaN	22.9398	22.92	NaN	O75449;O75449-2	O75449;O75449-2	Katanin p60 ATPa	KATNA1
NaN	NaN	NaN	NaN	NaN	NaN	27.1787	NaN	NaN	NaN	NaN	NaN	Q8IYT4;K7EM02;K	Q8IYT4;K7EM02;K	Katanin p60 ATPa	KATNAL2
22.9195	NaN	22.0011	22.28	NaN	22.0202	NaN	NaN	NaN	NaN	NaN	NaN	Q9BVA0;H3BT67;	Q9BVA0	Katanin p80 WD4	KATNB1
NaN	NaN	NaN	NaN	NaN	NaN	NaN	NaN	NaN	21.0766	NaN	NaN	Q8IY47;A0A087W	Q8IY47;A0A087W	Kelch repeat and	KBTBD2
NaN	NaN	NaN	23.5822	NaN	NaN	NaN	NaN	NaN	NaN	NaN	NaN	Q9P0J7;C9J3I2	Q9P0J7;C9J3I2	E3 ubiquitin-prote	KCMF1
24.6948	25.4868	25.3682	25.2142	25.624	25.5684	25.8191	26.2642	25.9591	25.8295	26.046	26.0497	Q13303-2;Q1330	Q13303-2;Q1330	Voltage-gated pot	KCNAB2
NaN	NaN	22.8776	21.9543	22.2456	NaN	NaN	NaN	22.4837	23.4857	22.4276	NaN	O15554;D1MQ08	O15554;D1MQ08	Intermediate cond	KCNN4
22.8878	NaN	23.2365	22.9757	22.8666	23.1977	NaN	NaN	24.0708	22.5305	NaN	NaN	Q8NC54	Q8NC54	Keratinocyte-asso	KCT2
20.1951	NaN	NaN	NaN	NaN	NaN	NaN	NaN	NaN	NaN	NaN	NaN	F5GWA4	F5GWA4		KCTD10
24.8858	24.7295	24.4748	24.8024	24.8891	24.5083	24.9061	NaN	NaN	25.547	25.1491	NaN	Q9H3F6;Q9H3F6-	Q9H3F6;Q9H3F6-	BTB/POZ domain-	KCTD10
25.4676	25.5984	25.4705	25.2952	25.2427	25.6913	25.6603	25.6089	25.2914	25.6334	25.4438	25.8865	Q96CX2	Q96CX2	BTB/POZ domain-	KCTD12

25.0584	24.2251	24.7317	25.1207	24.3389	24.9548	23.4948	24.2593	23.5733	24.0409	23.9118	24.0973	Q9BQ13;Q9BQ13-	Q9BQ13;Q9BQ13-	BTB/POZ domain-	KCTD14
23.8827	24.101	23.982	24.2314	24.2924	24.3908	24.145	25.4252	NaN	24.0844	24.7548	NaN	Q9NXV2;H3BSS5	Q9NXV2	BTB/POZ domain-	KCTD5
NaN	NaN	NaN	20.8682	NaN	NaN	NaN	NaN	NaN	NaN	NaN	NaN	Q7L273	Q7L273	BTB/POZ domain-	KCTD9
22.6414	23.775	23.4201	NaN	23.7999	23.8103	23.9895	NaN	24.2917	23.638	23.6025	23.5575	Q7Z4H8;Q7Z4H8-	Q7Z4H8;Q7Z4H8-	KDEL motif-contai	KDELC2
23.944	NaN	23.7012	24.1089	23.8169	23.85	NaN	NaN	NaN	23.8194	24.5878	NaN	O60341;O60341-2	O60341;O60341-2	Lysine-specific his	KDM1A
21.741	NaN	NaN	NaN	NaN	NaN	NaN	NaN	NaN	NaN	NaN	NaN	Q9Y2K7;Q9Y2K7-5	Q9Y2K7;Q9Y2K7-5	Lysine-specific de	KDM2A
21.5371	NaN	NaN	21.9482	NaN	NaN	NaN	NaN	NaN	NaN	NaN	NaN	Q7LBC6;Q7LBC6-3	Q7LBC6;Q7LBC6-3	Lysine-specific de	KDM3B
22.2876	NaN	NaN	NaN	23.0486	NaN	22.729	NaN	NaN	22.5501	NaN	NaN	Q06136;K7EQS7;K	Q06136;K7EQS7;K	3-ketodihydrosph	KDSR
28.6736	26.9118	27.5951	26.298	24.554	26.7514	28.0179	28.7106	31.1026	27.6634	28.2872	30.2408	Q14145;K7ESE0;K	Q14145	Kelch-like ECH-ass	KEAP1
28.0174	28.2554	27.9081	28.1393	28.3578	28.1561	26.9459	27.6845	27.3314	28.1062	28.6445	28.254	Q07666;Q07666-3	Q07666;Q07666-3	KH domain-contai	KHDRBS1
NaN	NaN	NaN	NaN	23.2209	NaN	NaN	NaN	NaN	NaN	NaN	NaN	P50053-2;P50053	P50053-2;P50053	Ketohexokinase	KHK
28.9202	29.1441	29.1057	29.0897	29.3736	29.3377	28.5692	29.0722	28.7541	29.252	29.8319	29.9925	Q92945;A0A087W	Q92945;A0A087W	Far upstream eler	KHSRP
27.5088	25.458	26.7162	27.6357	26.3745	26.6378	24.9188	NaN	NaN	25.9876	26.4153	24.3254	Q15397;S4R3K8	Q15397	Pumilio domain-c	KIAA0020
25.0088	23.8773	24.4776	25.3782	24.2537	24.0031	23.4605	NaN	24.4585	24.1123	23.449	24.0714	Q12768;E7EQI7;E	Q12768;E7EQI7	WASH complex su	KIAA0196
NaN	NaN	NaN	NaN	22.0508	NaN	NaN	NaN	NaN	NaN	NaN	NaN	Q92628	Q92628	Uncharacterized p	KIAA0232
24.5244	24.8206	24.7336	24.5467	24.9032	25.1114	24.5174	25.6037	25.8341	24.832	24.5875	24.7819	Q8IZA0;E7EN73;Q	Q8IZA0;E7EN73;Q	Dyslexia-associate	KIAA0319L
22.3444	NaN	NaN	22.6768	NaN	NaN	NaN	NaN	NaN	NaN	NaN	NaN	O15091;O15091-2	O15091;O15091-2	Mitochondrial rib	KIAA0391
NaN	NaN	NaN	20.2652	NaN	NaN	NaN	NaN	NaN	NaN	NaN	NaN	Q9Y4F3;Q9Y4F3-5	Q9Y4F3;Q9Y4F3-5	Meiosis arrest fen	KIAA0430
25.1684	24.2817	24.5403	25.4156	24.0282	24.8273	24.1776	24.4793	23.5643	24.1972	23.3669	23.0996	Q2M389;A0A087	Q2M389;A0A087	WASH complex su	KIAA1033
27.3408	26.3407	26.572	26.8226	26.1607	26.6495	26.3131	26.0506	25.9568	27.6546	27.0247	26.7159	Q96EK5;A0A0D9S	Q96EK5	KIF1-binding prot	KIAA1279
NaN	NaN	NaN	23.7848	NaN	NaN	NaN	NaN	NaN	NaN	NaN	NaN	Q69YN4;Q69YN4-	Q69YN4;Q69YN4-	Protein virilizer hc	KIAA1429
24.1963	23.3941	24.0304	24.449	23.642	24.1547	NaN	NaN	NaN	23.9001	23.9263	NaN	A2RU67;Q69YM1	A2RU67;Q69YM1	Uncharacterized p	KIAA1467;DKFZp7
19.6863	NaN	NaN	NaN	NaN	NaN	NaN	NaN	NaN	NaN	NaN	NaN	Q9P260;A0A075B	Q9P260;A0A075B	LisH domain and	KIAA1468
NaN	NaN	21.3055	NaN	NaN	NaN	NaN	NaN	NaN	NaN	NaN	NaN	Q9P206-2;Q9P20	Q9P206-2;Q9P20	Uncharacterized p	KIAA1522
26.4287	25.0865	25.2509	26.2596	25.3427	25.5949	24.6121	NaN	24.5253	25.5491	25.7258	25.4345	Q8TCG1;A0A087X	Q8TCG1;A0A087X	Protein CIP2A	KIAA1524
23.3561	24.3305	24.0068	23.5829	24.6234	23.5477	NaN	NaN	NaN	NaN	23.3817	NaN	A0MZ66;A0MZ66-	A0MZ66;A0MZ66-	Shootin-1	KIAA1598
22.4247	NaN	22.5794	NaN	NaN	NaN	NaN	NaN	22.4916	NaN	NaN	NaN	Q8IYS2;Q8IYS2-2	Q8IYS2;Q8IYS2-2	Uncharacterized p	KIAA2013
23.5381	23.0475	23.5563	23.5732	23.2235	23.1895	23.383	NaN	23.6583	24.1642	23.1827	24.0834	Q9ULH0;Q9ULH0-	Q9ULH0;Q9ULH0-	Kinase D-interacti	KIDINS220
27.0681	27.3469	26.8367	26.7704	27.5995	26.8894	26.7652	27.4396	27.3355	26.9488	27.3007	27.2925	P52732	P52732	Kinesin-like protei	KIF11
25.3157	24.1026	24.9102	24.8165	24.5444	24.6593	23.6917	NaN	NaN	24.0838	23.5249	23.8159	Q9NQT8;E7ERX9;	Q9NQT8;E7ERX9	Kinesin-like protei	KIF13B
28.3183	28.1191	28.8967	28.2726	27.8124	28.6534	30.6545	29.8056	29.65	28.8194	28.0592	28.4202	Q15058	Q15058	Kinesin-like protei	KIF14
24.8869	23.2855	23.5413	23.5118	23.0073	NaN	24.1813	24.249	23.4079	NaN	23.4358	23.3869	Q9NS87;Q9NS87-	Q9NS87;Q9NS87-	Kinesin-like protei	KIF15
NaN	NaN	20.7054	21.6759	NaN	NaN	NaN	NaN	NaN	NaN	NaN	NaN	Q96L93;Q96L93-5	Q96L93;Q96L93-5	Kinesin-like protei	KIF16B
NaN	NaN	NaN	NaN	NaN	NaN	NaN	NaN	NaN	NaN	NaN	24.1077	Q12756;Q12756-3	Q12756;Q12756-3	Kinesin-like protei	KIF1A
21.1693	NaN	NaN	NaN	NaN	NaN	NaN	NaN	NaN	NaN	NaN	NaN	O60333-2;O6033	O60333-2;O6033	Kinesin-like protei	KIF1B
24.4109	23.782	23.9727	23.8844	23.8994	23.7131	23.7957	24.1608	NaN	24.0918	NaN	NaN	O43896	O43896	Kinesin-like protei	KIF1C
26.3959	26.3932	26.7075	26.9713	26.563	26.8809	28.5859	28.1587	28.3099	27.4328	27.501	27.2962	O95235;O95235-2	O95235;O95235-2	Kinesin-like protei	KIF20A
26.852	28.23	27.6543	27.6301	28.2422	27.5745	29.0013	28.6859	28.9938	27.5362	28.238	28.8963	Q96Q89;Q96Q89-	Q96Q89;Q96Q89-	Kinesin-like protei	KIF20B
23.3613	NaN	23.1015	23.8007	22.8915	23.2177	23.2407	NaN	NaN	NaN	NaN	NaN	Q7Z4S6;Q7Z4S6-5	Q7Z4S6;Q7Z4S6-5	Kinesin-like protei	KIF21A
20.514	NaN	NaN	21.093	NaN	NaN	NaN	NaN	NaN	NaN	NaN	NaN	Q14807;H3BTH5;	Q14807;H3BTH5;	Kinesin-like protei	KIF22
30.4338	30.4743	30.943	30.3826	29.9538	30.7871	32.7996	31.9387	31.7875	31.2375	30.4695	30.899	Q02241;H7BYN4;	Q02241;H7BYN4	Kinesin-like protei	KIF23
23.2218	23.003	23.3101	23.0196	22.636	23.3363	25.1655	24.6731	24.6492	22.7118	21.5013	23.8151	Q02241-2	Q02241-2	Kinesin-like protei	KIF23
23.8832	23.7743	NaN	23.6233	NaN	NaN	NaN	NaN	NaN	NaN	NaN	NaN	Q02241-3	Q02241-3	Kinesin-like protei	KIF23
26.7256	25.7273	25.8546	26.0268	25.509	25.3166	27.1458	26.9078	26.7332	25.5479	25.6797	25.9915	O00139-2;O0013	O00139-2;O0013	Kinesin-like protei	KIF2A
27.3231	26.4344	26.5348	26.7965	26.661	26.2831	26.5325	26.7997	26.7304	27.3284	27.1855	26.8105	Q99661;Q99661-2	Q99661;Q99661-2	Kinesin-like protei	KIF2C
25.5605	24.8666	25.3781	25.4288	25.4249	24.842	25.0839	NaN	NaN	24.8386	NaN	NaN	Q9Y496;J3KPF9;A	Q9Y496;J3KPF9;A	Kinesin-like protei	KIF3A
26.2484	26.2599	26.1894	25.9438	25.901	25.7699	25.3115	NaN	26.1485	25.2511	25.5275	25.2448	O15066;O15066-2	O15066	Kinesin-like protei	KIF3B
26.9872	26.6488	27.0858	26.6185	26.0606	26.4557	28.1754	27.4435	28.2724	26.9125	26.3706	26.8742	O95239;O95239-2	O95239;O95239-2	Chromosome-ass	KIF4A
30.3562	29.9649	30.0338	30.1162	29.8765	29.9053	30.1283	30.3437	29.9459	30.3997	30.2463	29.8467	P33176;Q12840;J	P33176	Kinesin-1 heavy c	KIF5B
22.5737	21.7546	22.4728	22.118	21.78	21.6525	22.134	NaN	NaN	NaN	NaN	NaN	Q92845;Q92845-2	Q92845;Q92845-2	Kinesin-associate	KIFAP3
25.8772	25.3683	25.2535	25.6817	25.6526	25.3183	NaN	25.1178	24.6147	25.7276	26.2347	25.8321	Q9BW19;A0A087	Q9BW19	Kinesin-like protei	KIFC1
24.6599	24.8589	25.828	25.3955	25.3826	25.9768	25.0088	24.2286	25.12	25.9384	25.5731	25.7624	Q96J84;B4DN67;C	Q96J84;B4DN67;C	Kin of IRRE-like pr	KIRREL
23.2663	24.7131	24.3507	NaN	23.9675	23.1689	25.6548	27.2814	NaN	NaN	NaN	NaN	Q15726;A0A0D9S	Q15726;A0A0D9S	Metastasis-suppr	KISS1
27.366	26.7098	26.5239	26.9949	26.5708	26.3038	26.7674	26.6281	26.2203	26.8003	26.477	25.217	Q07866-4;Q0786	Q07866-4;Q0786	Kinesin light chain	KLC1
26.9862	27.2541	26.8225	26.775	27.0838	26.8564	27.4179	28.0981	27.6722	27.0758	27.3892	27.6121	Q9H0B6;Q9H0B6-	Q9H0B6;Q9H0B6-	Kinesin light chain	KLC2
23.7375	24.0624	24.1181	23.0884	23.94	23.8619	23.3178	24.6611	NaN	23.2203	23.6025	23.6532	Q9NSK0;Q9NSK0-	Q9NSK0;Q9NSK0-	Kinesin light chain	KLC4
NaN	NaN	23.2536	NaN	NaN	NaN	NaN	NaN	NaN	NaN	NaN	NaN	Q03164;Q03164-2	Q03164;Q03164-2	Histone-lysine N-r	KMT2A
24.721	24.444	23.8534	24.3082	23.7211	24.057	NaN	NaN	NaN	24.0074	23.8224	NaN	Q9Y448;V9GY01;C	Q9Y448;V9GY01	Small kinetochore	KNSTRN
26.0893	25.1954	25.2513	25.7464	24.9469	25.425	25.3163	24.6242	24.9668	25.4425	25.3426	24.6805	P50748;E7ES84;P	P50748;E7ES84	Kinetochore-asso	KNTC1
25.8954	25.099	25.4873	25.9729	25.3145	25.9043	25.6323	25.9637	24.8731	25.1596	25.7294	25.4631	P52294;C9JYI4;C9	P52294;C9JYI4	Importin subunit a	KPNA1

29.0113	28.8568	29.0269	29.0744	29.3065	29.1474	28.3848	28.2678	28.3891	28.7744	29.1042	29.2625	P52292;J3QLL0;J3	P52292;J3QLL0;J3	Importin subunit a	KPNA2
24.8614	25.0893	24.8713	24.7942	24.8074	25.0867	25.0535	26.1377	NaN	24.1988	24.554	24.8497	O00505;H0Y4S9	O00505	Importin subunit a	KPNA3
25.7248	25.431	25.978	25.7128	25.7166	25.8198	26.1015	NaN	26.182	25.5979	25.7507	25.3481	O00629;H7C4F6	O00629	Importin subunit a	KPNA4
26.5434	26.2859	26.4968	26.6421	26.4666	26.3919	26.0734	26.2438	26.2668	26.3968	26.4796	26.4865	O60684;Q5TFJ7;S	O60684;Q5TFJ7	Importin subunit a	KPNA6
31.4874	30.9259	31.5141	31.1651	30.9448	31.5404	31.4576	31.6603	31.3561	31.0619	31.2427	31.597	Q14974;Q14974-2	Q14974;Q14974-2	Importin subunit b	KPNB1
NaN	NaN	NaN	23.4646	NaN	NaN	NaN	25.4376	NaN	NaN	NaN	NaN	Q5T749	Q5T749	Keratinocyte proli	KPRP
26.5362	26.2988	26.4819	26.3646	26.2531	26.8495	27.0539	26.1586	26.9851	28.031	27.4757	27.5958	P01116-2	P01116-2	GTPase KRas;GTP	KRAS
24.93	24.0096	24.2617	25.1028	24.1445	24.5281	NaN	NaN	NaN	23.5897	22.9169	NaN	Q13601;A0A087W	Q13601;A0A087W	KRR1 small subun	KRR1
29.2071	30.5222	29.7178	29.8752	30.5854	30.0276	29.8862	31.0247	30.2785	30.0571	29.9983	29.7133	P05783;F8VZY9;C	P05783;F8VZY9	Keratin, type I cyt	KRT18
29.1533	31.1606	29.5864	29.1871	30.571	28.7719	31.2756	32.0618	31.3221	28.7698	30.7274	30.1425	P35908	P35908	Keratin, type II cy	KRT2
22.8787	NaN	NaN	25.678	NaN	NaN	26.136	NaN	NaN	NaN	NaN	NaN	P04259	P04259	Keratin, type II cy	KRT6B
23.1837	22.8746	23.1339	23.4418	22.9781	23.3283	NaN	NaN	NaN	23.4333	23.7438	NaN	Q96EK9	Q96EK9	Protein KTI12 hon	KTI12
29.6218	30.2632	29.3372	29.1636	29.6155	29.0504	28.5987	29.3835	28.0513	27.7619	28.0019	26.949	Q86UP2;Q86UP2-	Q86UP2;Q86UP2-	Kinectin	KTN1
23.0806	23.3084	23.4383	22.4616	23.2453	23.4994	23.1135	NaN	23.7996	NaN	NaN	22.8715	Q16719;Q16719-2	Q16719;Q16719-2	Kynureninase	KYNU
25.9449	27.204	27.0385	25.888	26.794	26.9416	26.1836	25.8641	25.4828	25.5917	25.0616	NaN	P32004;P32004-2	P32004;P32004-2	Neural cell adhesi	L1CAM
22.6118	22.0992	22.3134	22.825	22.3838	22.765	NaN	NaN	NaN	NaN	NaN	NaN	Q9H9P8;C9JVN9;C	Q9H9P8;C9JVN9;C	L-2-hydroxyglutar	L2HGDH
NaN	22.8504	NaN	NaN	NaN	NaN	NaN	NaN	NaN	NaN	NaN	NaN	Q96EM0;D6RC46;	Q96EM0;D6RC46;	Trans-3-hydroxy-L	L3HYPDH
22.9423	23.4154	22.8416	23.5386	23.6827	23.3352	23.5595	23.5157	23.5886	23.0802	NaN	NaN	P83111;P83111-2	P83111	Serine beta-lactar	LACTB
24.8748	25.1034	24.8455	24.767	NaN	24.6733	25.7171	NaN	NaN	25.9453	25.3065	NaN	Q53H82	Q53H82	Beta-lactamase-lil	LACTB2
NaN	NaN	NaN	24.2971	NaN	NaN	NaN	NaN	NaN	NaN	NaN	NaN	Q14657	Q14657	EKC/KEOPS compl	LAGE3
NaN	NaN	NaN	NaN	NaN	NaN	25.1953	NaN	NaN	NaN	NaN	NaN	Q16363;A0A0A0M	Q16363;A0A0A0M	Laminin subunit a	LAMA4
26.2208	25.9696	26.2027	25.226	24.975	25.7345	28.0446	27.0861	26.3174	25.9543	25.9585	25.1369	O15230;O15230-2	O15230	Laminin subunit a	LAMA5
22.7755	23.3358	22.7511	21.3095	23.2811	22.9594	27.528	26.3653	25.1386	24.7075	24.5483	22.4561	P07942;G3XAI2;E	P07942;G3XAI2	Laminin subunit b	LAMB1
23.1342	23.7213	23.3736	22.7771	23.6583	23.1854	25.7768	22.1236	23.56	22.5266	23.6529	22.6998	P55268;F5H520;A	P55268	Laminin subunit b	LAMB2
24.6629	25.5722	24.9175	24.6637	24.9806	24.7442	27.0321	26.1435	26.6938	26.2352	25.0738	25.0056	Q13751;X1WI29;C	Q13751	Laminin subunit b	LAMB3
24.8314	25.6595	25.2981	24.2146	24.5957	24.1045	27.6085	26.6244	26.0829	25.3122	25.4221	25.3826	P11047	P11047	Laminin subunit g	LAMC1
24.4035	25.2807	23.7726	23.8883	24.6222	22.4892	28.5201	26.8264	27.394	25.3469	23.4255	25.6679	Q13753;Q13753-2	Q13753;Q13753-2	Laminin subunit g	LAMC2
28.9247	29.9544	29.6707	29.273	29.3282	29.7184	29.2098	28.715	29.5786	29.4128	29.1482	29.569	P11279;P11279-2	P11279;P11279-2	Lysosome-associa	LAMP1
25.4666	26.3693	26.1367	26.0445	25.4669	26.2833	26.2472	25.341	25.7192	25.7799	24.9227	25.3077	P13473-2;P13473	P13473-2;P13473	Lysosome-associa	LAMP2
26.5541	26.7149	26.7229	25.9986	26.0498	26.0031	25.4239	25.7856	26.5132	25.1556	24.918	26.0134	Q6IAA8;F5GX19;F	Q6IAA8;F5GX19;F	Ragulator comple	LAMTOR1
24.0966	24.2065	NaN	23.2485	NaN	NaN	NaN	NaN	NaN	NaN	NaN	NaN	Q9Y2Q5;Q9Y2Q5-	Q9Y2Q5;Q9Y2Q5-	Ragulator comple	LAMTOR2
24.5267	25.123	24.3898	23.9849	23.8411	23.6459	24.012	23.4038	NaN	NaN	NaN	NaN	Q9UHA4;Q9UHA4	Q9UHA4;Q9UHA4	Ragulator comple	LAMTOR3
23.2881	23.9328	22.8638	NaN	23.264	NaN	NaN	NaN	NaN	NaN	NaN	NaN	Q0VGL1;A0A087V	Q0VGL1;A0A087V	Ragulator comple	LAMTOR4
24.4235	25.3783	24.5122	24.1413	24.5454	24.2611	24.6676	NaN	NaN	23.9532	NaN	24.3529	O43504;R4GMU8	O43504;R4GMU8	Ragulator comple	LAMTOR5
25.3588	25.0498	25.2565	25.3435	24.6367	25.3518	26.0023	NaN	25.578	26.0026	25.6122	25.4531	O43813;E9PHS0;F	O43813;E9PHS0;F	LanC-like protein	LANCL1
24.4705	23.268	NaN	23.7871	NaN	23.7412	NaN	NaN	NaN	23.5241	23.1656	NaN	Q9NS86	Q9NS86	LanC-like protein	LANCL2
28.5577	28.5522	28.3858	28.434	28.5887	28.486	28.5138	28.5096	28.7349	28.6721	28.522	28.8617	P28838-2;P28838	P28838-2;P28838	Cytosol aminopep	LAP3
NaN	NaN	24.2638	24.0266	NaN	24.3332	NaN	NaN	NaN	NaN	NaN	NaN	Q15012	Q15012	Lysosomal-associa	LAPTM4A
NaN	NaN	NaN	23.2386	NaN	NaN	NaN	NaN	NaN	NaN	NaN	NaN	Q13571	Q13571	Lysosomal-associa	LAPTM5
28.6633	28.1579	28.2043	28.4639	27.9962	27.8198	26.8494	27.8716	25.4078	27.2134	26.9466	27.7758	Q6PKG0;Q6PKG0-	Q6PKG0;Q6PKG0-	La-related protein	LARP1
25.1354	NaN	23.8561	24.4099	23.1569	NaN	NaN	NaN	NaN	NaN	NaN	NaN	Q71RC2;Q96J85;C	Q71RC2;Q96J85;C	La-related protein	LARP4
25.0421	24.7849	24.0335	24.9259	24.2612	24.4565	NaN	NaN	NaN	23.6961	23.7659	NaN	Q92615;H0Y641;H	Q92615	La-related protein	LARP4B
22.6965	NaN	23.0308	23.8525	NaN	NaN	NaN	NaN	24.3132	NaN	NaN	24.2724	Q9BRS8;Q9BRS8-2	Q9BRS8	La-related protein	LARP6
23.4182	NaN	23.1842	23.4742	23.7304	NaN	NaN	NaN	NaN	NaN	23.585	23.0082	Q4G0J3;D6RFF0;C	Q4G0J3;D6RFF0;C	La-related protein	LARP7
30.7288	29.467	29.7677	30.5097	29.8145	29.8375	29.2285	29.5279	29.3101	29.8796	29.6585	29.2608	Q9P2J5;Q9P2J5-2	Q9P2J5;Q9P2J5-2	Leucine--tRNA liga	LARS
21.9288	NaN	NaN	NaN	NaN	NaN	NaN	NaN	NaN	NaN	NaN	NaN	Q15031;E9PHM2	Q15031;E9PHM2	Probable leucine--	LARS2
25.9891	23.4007	25.3899	26.2586	24.9336	25.1392	NaN	NaN	NaN	24.5947	24.4755	24.1391	Q9Y4W2;Q9Y4W2	Q9Y4W2;Q9Y4W2	Ribosomal biogen	LAS1L
29.4394	30.3952	29.7414	29.1233	30.0888	29.8083	29.2114	29.6601	29.2569	28.9807	28.8851	29.6023	Q14847;Q14847-2	Q14847;Q14847-2	LIM and SH3 dom	LASP1
24.9659	25.3371	25.6534	24.8114	25.3328	25.9542	NaN	NaN	NaN	NaN	22.8964	NaN	Q9GZY6;C9JA24;C	Q9GZY6;C9JA24;C	Linker for activatio	LAT2
NaN	NaN	NaN	NaN	NaN	NaN	22.5524	NaN	NaN	NaN	NaN	NaN	Q6UX15;Q6UX15-	Q6UX15;Q6UX15-	Layilin	LAYN
23.2277	NaN	22.7138	23.2565	NaN	NaN	NaN	NaN	NaN	22.4847	22.9902	NaN	Q14739;C9JXK0;C	Q14739;C9JXK0	Lamin-B receptor	LBR
NaN	NaN	22.0649	25.1324	23.4478	NaN	22.9416	NaN	NaN	NaN	NaN	NaN	Q6UWP7;Q6UWP	Q6UWP7;Q6UWP	Lysocardiolipin ac	LCLAT1
NaN	NaN	23.0805	22.3906	NaN	NaN	NaN	NaN	NaN	NaN	23.4911	NaN	Q9UIC8;Q9UIC8-3	Q9UIC8;Q9UIC8-3	Leucine carboxyl r	LCMT1
24.2052	NaN	23.4344	23.7764	NaN	NaN	22.498	NaN	NaN	23.6511	23.8596	23.564	O60294;H3BU01	O60294	tRNA wybutosine-	LCMT2
28.6903	28.8784	29.3896	28.506	29.0828	29.4672	27.8763	27.7575	27.911	28.7516	28.6852	28.5861	P13796;Q5TBN3;F	P13796	Plastin-2	LCP1
33.3494	33.498	33.8193	33.2376	33.4948	33.7064	33.3283	32.4451	32.519	33.191	33.1746	32.9026	P00338;P00338-3	P00338;P00338-3	L-lactate dehydro	LDHA
31.7294	31.9416	31.6365	31.4406	31.9629	31.7863	32.4531	32.4125	32.3262	32.2532	32.4993	32.7089	P07195;A8MW50	P07195;A8MW50	L-lactate dehydro	LDHB
27.4067	27.4989	27.2454	27.0901	26.8261	27.0707	27.2025	27.3484	26.9822	26.9472	26.6798	26.5101	P01130;J3KMZ9;P	P01130;J3KMZ9;P	Low-density lipop	LDLR
NaN	NaN	NaN	NaN	NaN	NaN	NaN	NaN	NaN	NaN	NaN	19.7515	Q5SW96	Q5SW96	Low density lipop	LDLRAP1
22.3825	NaN	NaN	22.5491	NaN	NaN	NaN	NaN	NaN	NaN	NaN	NaN	Q8NC56;H0Y9B7;I	Q8NC56	LEM domain-cont	LEMD2

NaN	NaN	NaN	NaN	NaN	NaN	NaN	NaN	NaN	NaN	27.4442	29.1087	Q9Y2U8	Q9Y2U8	Inner nuclear mer	LEMD3
22.6278	NaN	21.6296	22.623	NaN	NaN	NaN	NaN	NaN	NaN	NaN	NaN	Q96PV6;Q96PV6-:	Q96PV6;Q96PV6-:	Leukocyte recepto	LENG8
23.3031	NaN	23.1065	23.1456	23.2797	23.6619	NaN	NaN	NaN	23.5557	23.9419	NaN	Q8WVC0;Q8WVC	Q8WVC0;Q8WVC	RNA polymerase-a	LEO1
26.0951	26.3963	25.9867	25.6156	26.2563	25.6465	26.2756	27.2405	27.2605	25.5568	25.8729	26.4845	Q32P28;Q32P28-4	Q32P28;Q32P28-4	Prolyl 3-hydroxyla	LEPRE1
24.005	23.5015	23.4786	24.5474	23.5861	23.3855	23.0553	23.3516	NaN	22.5919	22.6715	22.8824	O95202;O95202-2	O95202	LETM1 and EF-har	LETM1
31.2405	30.6444	30.0247	30.5634	29.4999	29.6869	30.7597	28.6944	28.5877	30.2828	29.3326	28.4553	P09382;F8WEI7	P09382	Galectin-1	LGALS1
27.7762	28.2431	28.477	27.8985	27.9912	28.5676	28.2293	27.22	27.5684	28.4401	27.9071	27.9753	P17931;G3V3R6	P17931;G3V3R6	Galectin-3;Galecti	LGALS3
29.7178	29.9488	28.8573	28.9949	29.2846	28.5255	31.1694	30.9257	30.2254	30.2488	30.1617	29.2768	Q08380;K7EP36;K	Q08380	Galectin-3-binding	LGALS3BP
27.8391	27.4727	27.7672	27.0303	26.7094	27.1355	27.2152	27.0657	26.7425	26.9446	26.7704	25.9239	O00214-2;O00214	O00214-2;O00214	Galectin-8;Galecti	LGALS8
22.5636	24.228	NaN	NaN	23.2437	NaN	26.365	26.4392	24.2623	22.138	NaN	NaN	Q99538;Q99538-3	Q99538;Q99538-3	Legumain	LGMN
23.3996	23.99	24.1363	23.8479	24.0351	23.4757	24.1694	24.954	NaN	24.4983	23.9042	24.1148	Q6ZUX7	Q6ZUX7	Lipoma HMGIC fu	LHFPL2
23.3276	NaN	23.4664	23.3961	23.7678	NaN	NaN	NaN	NaN	24.9614	25.2127	23.9419	P18858-3;P18858	P18858-3;P18858	DNA ligase 1;DNA	LIG1
23.1473	NaN	22.8569	23.7546	22.8572	22.467	NaN	NaN	NaN	NaN	NaN	NaN	P49916;P49916-3	P49916;P49916-3	DNA ligase 3	LIG3
21.9405	NaN	21.3525	NaN	NaN	NaN	NaN	NaN	NaN	22.1825	NaN	NaN	P49917;A0A0C4D	P49917;A0A0C4D	DNA ligase 4;DNA	LIG4
27.751	28.4214	28.4948	27.8845	28.5526	28.6091	26.8779	24.5319	26.9997	28.2994	28.2608	28.7873	Q9UHB6;Q9UHB6	Q9UHB6;Q9UHB6	LIM domain and a	LIMA1
23.1716	23.7403	23.6114	22.9097	23.5793	NaN	NaN	NaN	NaN	NaN	NaN	NaN	Q9UPQ0-3;Q9UP(	Q9UPQ0-3;Q9UP(	LIM and calponin	LIMCH1
NaN	22.4191	22.2319	NaN	NaN	22.2106	NaN	NaN	NaN	NaN	NaN	NaN	Q9UGP4;C9JRJ5	Q9UGP4;C9JRJ5	LIM domain-conta	LIMD1
NaN	NaN	21.714	NaN	NaN	NaN	NaN	NaN	NaN	NaN	NaN	NaN	Q9BT23;J3QQM5;	Q9BT23;J3QQM5;	LIM domain-conta	LIMD2
27.1699	28.242	28.2619	27.3516	27.9716	28.4446	28.3393	27.8638	27.7029	28.7058	28.3677	28.0754	Q9NUP9;G3V1D4	Q9NUP9;G3V1D4	Protein lin-7 hom	LIN7C
NaN	NaN	NaN	NaN	NaN	NaN	25.363	NaN	NaN	NaN	NaN	NaN	P38571;A0A0A0N	P38571;A0A0A0N	Lysosomal acid lip	LIPA
21.9503	NaN	NaN	NaN	NaN	NaN	NaN	NaN	NaN	NaN	NaN	NaN	Q9Y5X9;J3QQQ0;	Q9Y5X9;J3QQQ0;	Endothelial lipase	LIPG
27.127	27.1392	27.4331	27.0341	26.8547	27.4311	27.1876	26.6886	27.1752	27.0324	27.0498	26.7816	Q15334;A0A087V	Q15334;A0A087V	Lethal(2) giant lar	LLGL1
24.9569	25.0037	26.161	24.9986	25.0713	25.5029	NaN	NaN	NaN	22.9047	NaN	NaN	Q6P1M3;J3QRV5;	Q6P1M3;J3QRV5;	Lethal(2) giant lar	LLGL2
27.4006	27.3883	27.6842	27.6426	27.5088	27.867	27.1973	27.6582	28.2874	26.9252	26.8028	27.7922	P49257	P49257	Protein ERGIC-53	LMAN1
27.928	27.3915	27.6402	28.0175	27.0215	27.3637	28.6218	27.2703	27.4782	27.7357	26.5689	26.5852	Q12907;D6RBV2;	Q12907;D6RBV2;	Vesicular integral-	LMAN2
22.8871	22.6902	23.1501	22.9243	NaN	NaN	NaN	NaN	NaN	NaN	NaN	NaN	Q9H0V9;Q9H0V9-	Q9H0V9;Q9H0V9-	VIP36-like protein	LMAN2L
23.1374	NaN	23.5051	24.0422	NaN	NaN	NaN	NaN	NaN	23.3815	NaN	NaN	Q68DH5	Q68DH5	LMBR1 domain-co	LMBRD2
23.4654	23.3215	23.8604	23.8684	23.407	NaN	24.637	23.8717	24.9257	24.0325	24.1729	24.1069	Q9BU23;Q9BU23-	Q9BU23;Q9BU23-	Lipase maturation	LMF2
22.4924	NaN	NaN	23.321	NaN	NaN	NaN	NaN	NaN	NaN	NaN	NaN	P02545;P02545-6	P02545;P02545-6	Prelamin-A/C;Lam	LMNA
30.0243	30.7243	30.6375	30.3332	30.8234	30.9918	30.2038	30.4742	29.9894	31.0378	31.2628	31.2363	P02545-2;Q3BDU	P02545-2;Q3BDU	Prelamin-A/C;Lam	LMNA
29.1561	29.3009	29.0815	29.3396	29.2065	29.492	28.5624	28.8469	28.424	29.637	29.8212	29.598	P20700;E9PBF6;A	P20700;E9PBF6;A	Lamin-B1	LMNB1
26.8067	27.6748	27.4721	27.2745	28.1798	27.64	27.0298	27.4126	27.4639	28.1719	29.0542	28.6468	Q03252;J9JID7	Q03252;J9JID7	Lamin-B2	LMNB2
NaN	NaN	NaN	21.7296	NaN	NaN	NaN	NaN	NaN	NaN	NaN	NaN	Q8WWI1-3;E9PM	Q8WWI1-3;E9PM	LIM domain only	LMO7
27.2395	27.9527	28.1719	27.5256	27.8203	28.4132	26.5437	25.565	25.8232	27.2886	27.113	26.8726	F8WD26	F8WD26		LMO7
NaN	NaN	NaN	21.3126	NaN	NaN	NaN	NaN	NaN	NaN	NaN	NaN	Q9C0E8;Q9C0E8-2	Q9C0E8;Q9C0E8-2	Protein lunapark	LNP
28.8632	27.9319	28.6336	28.9043	28.0845	28.6858	27.4973	23.6083	25.8422	27.9819	26.2354	26.2887	Q9UIQ6;Q9UIQ6-	Q9UIQ6;Q9UIQ6-	Leucyl-cystinyl am	LNPEP
27.7332	26.7384	26.8616	27.5196	26.9807	27.1365	25.18	26.7353	27.3452	26.0043	26.2385	26.5669	P36776;K7EKE6;P	P36776;K7EKE6;P	Lon protease hom	LONP1
26.3354	26.5759	26.2873	25.3577	25.6075	24.7695	27.5818	30.0328	28.9269	24.7129	25.0775	24.781	P28300;Q08397	P28300	Protein-lysine 6-o	LOX
26.8094	27.0297	27.3426	26.0246	26.8276	26.3939	29.3706	29.5063	28.9876	26.8811	26.823	27.8339	Q9Y4K0;H0YAP6;I	Q9Y4K0	Lysyl oxidase hom	LOXL2
25.5177	24.5603	25.3829	25.2288	24.1983	25.4686	24.2347	NaN	24.3154	26.084	25.4782	24.914	Q92633;Q6GPG7;	Q92633;Q6GPG7;	Lysophosphatidic	LPAR1
23.8597	NaN	22.3628	23.8724	22.5657	23.1733	23.6363	NaN	NaN	24.7154	NaN	NaN	Q8NF37;A0A0G2J	Q8NF37;A0A0G2J	Lysophosphatidyl	LPCAT1
25.3918	26.6077	26.8075	25.6241	26.696	26.8749	25.7701	24.7978	26.336	26.5676	26.1174	26.4141	O95490;O95490-2	O95490;O95490-2	Latrophilin-2	LPHN2;ADGRL2
NaN	NaN	NaN	NaN	NaN	NaN	26.0807	NaN	NaN	NaN	NaN	NaN	Q92539	Q92539	Phosphatidate ph	LPIN2
24.3919	23.8032	23.8201	23.5883	23.0826	23.9931	NaN	NaN	NaN	23.3037	23.0212	NaN	Q93052;A0A087V	Q93052;A0A087V	Lipoma-preferred	LPP
23.5367	23.3562	23.1988	22.8812	23.6683	22.9242	NaN	NaN	NaN	24.2517	NaN	NaN	O60711;O60711-2	O60711;O60711-2	Leupaxin	LPXN
26.8187	25.71	26.3323	26.7244	26.3093	26.5141	NaN	NaN	23.5968	25.1824	24.556	NaN	P50851-2;P50851	P50851-2;P50851	Lipopolysaccharid	LRBA
24.7137	23.9112	23.5572	24.504	23.8679	23.5766	23.6484	NaN	24.1781	24.2255	23.5616	NaN	Q9Y2L9;Q9Y2L9-2	Q9Y2L9;Q9Y2L9-2	Leucine-rich repe	LRCH1
23.9538	22.9069	23.2095	23.9868	22.841	NaN	NaN	NaN	NaN	23.7815	23.3174	NaN	Q96II8;Q96II8-3;C	Q96II8;Q96II8-3;C	Leucine-rich repe	LRCH3
24.8812	24.9175	24.5546	24.4022	23.9965	24.4463	NaN	NaN	25.3109	24.4763	24.0468	24.897	Q7Z4F1;H0YHW7;	Q7Z4F1;H0YHW7;	Low-density lipop	LRP10
NaN	NaN	21.7716	NaN	NaN	NaN	NaN	NaN	NaN	NaN	NaN	NaN	Q9Y561;Q9Y561-2	Q9Y561;Q9Y561-2	Low-density lipop	LRP12
22.951	22.7132	22.3469	22.5751	23.0862	22.5841	NaN	NaN	NaN	23.3362	23.2594	NaN	P30533	P30533	Alpha-2-macroglo	LRPAP1
30.8832	30.0331	30.1581	31.3386	30.404	30.3738	28.4201	28.9594	29.5843	29.2248	29.2158	28.8159	P42704;B8ZZ38;A	P42704	Leucine-rich PPR r	LRPPRC
24.9328	24.5009	25.1608	24.7752	24.4982	25.2011	24.8493	23.9173	NaN	25.3661	24.5505	24.2826	Q9BTT6;Q5T0G3;	Q9BTT6	Leucine-rich repe	LRRC1
NaN	NaN	NaN	NaN	NaN	NaN	NaN	NaN	NaN	20.181	NaN	NaN	Q15048;E9PNL0;E	Q15048;E9PNL0;E	Leucine-rich repe	LRRC14
26.0901	25.7749	25.2197	25.4259	25.8307	24.9679	25.7729	25.0912	24.6666	25.6117	25.4099	25.1618	Q8TCA0;Q8TCA0-	Q8TCA0;Q8TCA0-	Leucine-rich repe	LRRC20
22.9556	NaN	22.573	22.6956	22.8971	NaN	NaN	NaN	NaN	22.1817	22.8533	22.6276	Q9H9A6	Q9H9A6	Leucine-rich repe	LRRC40
23.7685	NaN	NaN	NaN	NaN	NaN	NaN	NaN	NaN	NaN	NaN	NaN	A0A087WTU1;Q1	A0A087WTU1;Q1	Leucine-rich repe	LRRC41
26.0076	26.136	25.8145	25.6817	26.0333	25.9503	25.8494	25.8549	26.452	25.9559	26.2788	26.4696	Q8N1G4	Q8N1G4	Leucine-rich repe	LRRC47
24.6096	25.0912	25.3945	24.7888	25.1885	25.4791	24.9317	NaN	25.0201	25.1545	25.23	25.3709	Q8N9N7;H3BSW0	Q8N9N7;H3BSW0	Leucine-rich repe	LRRC57
26.1284	26.1455	25.8604	26.0911	25.97	25.8236	26.1286	26.1972	26.3158	25.7483	25.6478	25.6998	Q96AG4	Q96AG4	Leucine-rich repe	LRRC59

NaN	26.1629	24.6993	24.0712	24.1015	24.143	NaN	27.0654	28.0381	26.6302	26.5776	27.5299	Q96NW7;A0A075	Q96NW7;A0A075	Leucine-rich repea	LRRC7
26.6252	26.2434	27.1144	26.8313	26.6709	27.3112	26.418	25.9175	26.6216	26.6625	26.4411	26.547	Q8IWT6	Q8IWT6	Volume-regulatec	LRRC8A
26.7694	26.2373	27.0863	27.0072	26.5855	27.169	26.0811	25.1116	26.3456	26.8474	26.4263	26.5398	Q8TDW0;A6NED7	Q8TDW0	Volume-regulatec	LRRC8C
24.5943	24.1527	24.7086	24.7198	24.1059	24.7633	24.3704	NaN	NaN	24.6433	24.2844	24.0294	Q7L1W4;E9PMF9	Q7L1W4	Volume-regulatec	LRRC8D
25.3654	25.5333	26.1286	25.9129	26.1722	25.8535	25.8071	25.5969	26.481	26.0447	25.5428	26.3144	Q6NSJ5;M0QZ48;	Q6NSJ5	Volume-regulatec	LRRC8E
25.8994	25.6291	25.3286	25.2606	24.8012	25.1151	25.4547	25.3366	25.1755	24.7084	24.8953	25.1814	Q32MZ4-3	Q32MZ4-3	Leucine-rich repea	LRRFIP1
24.183	24.806	24.4352	24.03	25.0136	24.5603	24.5912	25.837	24.5879	24.3662	25.1247	24.4974	Q9Y608-2;Q9Y608	Q9Y608-2;Q9Y608	Leucine-rich repea	LRRFIP2
NaN	NaN	NaN	NaN	NaN	NaN	NaN	NaN	NaN	NaN	26.3753	NaN	Q38SD2;E9PK39;I	Q38SD2	Leucine-rich repea	LRRK1
NaN	23.1438	23.1742	NaN	22.9625	NaN	NaN	NaN	NaN	22.866	NaN	23.9631	Q6UWE0;Q6UWE	Q6UWE0;Q6UWE	E3 ubiquitin-prote	LRSAM1
23.7452	22.9758	23.0456	23.5507	22.6333	22.2994	NaN	NaN	NaN	22.6274	22.8118	NaN	Q9UFC0;H7C5S6;I	Q9UFC0;H7C5S6	Leucine-rich repea	LRWD1
25.8633	24.2192	24.7414	25.7086	24.5372	24.396	24.1637	NaN	22.7879	23.7433	NaN	NaN	Q9H089;H7C2X7;I	Q9H089	Large subunit GTF	LSG1
23.3088	23.9152	23.3985	22.8205	23.7775	23.3777	23.4992	NaN	NaN	NaN	23.4145	24.237	O15116;E5RJ47;E!	O15116;E5RJ47;E!	U6 snRNA-associa	LSM1
25.2047	25.7349	25.2034	24.796	25.3644	25.085	24.8849	24.9887	NaN	23.7648	24.6022	25.1688	Q3MHD2;Q3MHD	Q3MHD2;Q3MHD	Protein LSM12 ho	LSM12
22.6935	23.1588	NaN	NaN	23.0879	NaN	NaN	NaN	NaN	NaN	NaN	NaN	Q8ND56;Q8ND56	Q8ND56;Q8ND56	Protein LSM14 ho	LSM14A
23.7545	24.0511	23.8455	23.9593	24.1458	23.4677	NaN	24.1812	NaN	22.9456	NaN	23.4863	Q9BX40;Q9BX40-:	Q9BX40;Q9BX40-:	Protein LSM14 ho	LSM14B
25.0047	25.1283	24.821	24.8475	24.972	24.8299	24.8675	24.6932	24.7709	25.1271	25.0678	25.2678	Q9Y333	Q9Y333	U6 snRNA-associa	LSM2
24.152	24.9827	24.357	24.17	25.1094	24.7419	24.7984	25.2322	24.6354	24.6018	25.0929	25.2578	P62310	P62310	U6 snRNA-associa	LSM3
25.433	25.2944	24.9426	25.2021	25.097	25.0896	25.1059	NaN	NaN	25.831	25.6512	NaN	Q9Y4Z0;V9GZ56;L	Q9Y4Z0;V9GZ56;L	U6 snRNA-associa	LSM4
25.91	26.2952	25.9559	25.7852	26.2342	25.975	25.8696	25.5791	25.2839	26.8916	26.667	26.2505	P62312	P62312	U6 snRNA-associa	LSM6
23.3617	23.6576	24.0088	23.5237	25.3015	23.2225	25.5518	NaN	23.78	25.1136	24.3929	25.1373	Q9UK45;K7ENY5;I	Q9UK45;K7ENY5;I	U6 snRNA-associa	LSM7
24.3821	24.7375	24.567	24.6324	24.4891	24.7643	24.5005	NaN	NaN	24.9001	24.8942	24.6647	O95777;F2Z2Y6;C	O95777;F2Z2Y6;C	U6 snRNA-associa	LSM8
28.6076	29.0223	29.6796	28.8478	28.9799	29.8265	28.6454	28.2451	28.726	29.5955	29.1198	29.1748	Q86X29;S4R3V8;C	Q86X29;S4R3V8;C	Lipolysis-stimulat	LSR
NaN	NaN	NaN	22.535	NaN	NaN	NaN	NaN	NaN	NaN	NaN	NaN	Q86X29-3	Q86X29-3	Lipolysis-stimulat	LSR
25.3342	25.0849	25.4261	25.3717	24.8248	25.0657	24.6106	25.9261	25.1608	25.3628	24.6912	23.6066	P48449;P48449-3	P48449;P48449-3	Lanosterol syntha	LSS
27.7636	28.213	28.054	27.5724	28.046	28.1499	28.2322	27.7129	27.7773	28.3497	28.1584	28.0837	P09960;P09960-4	P09960;P09960-4	Leukotriene A-4 h	LTA4H
NaN	NaN	NaN	NaN	NaN	NaN	26.607	27.7824	27.9573	24.2262	24.3473	25.7943	Q14766;Q14766-4	Q14766;Q14766-4	Latent-transformi	LTBP1
NaN	NaN	NaN	NaN	NaN	NaN	25.5875	26.7705	NaN	NaN	NaN	NaN	Q8N2S1;Q8N2S1-	Q8N2S1;Q8N2S1-	Latent-transformi	LTBP4
25.5358	23.915	24.2079	24.2269	23.6154	24.2716	NaN	NaN	NaN	23.995	23.5577	23.4804	O94822;O94822-3	O94822;O94822-3	E3 ubiquitin-prote	LTN1
23.7559	22.1622	22.5137	23.432	22.3698	22.6447	NaN	NaN	NaN	22.4709	NaN	NaN	Q96GA3;A0A075E	Q96GA3;A0A075E	Protein LTV1 hom	LTV1
27.3915	26.4213	27.0272	27.8642	26.4362	28.2467	26.7676	26.0386	NaN	28.0104	27.7209	27.4091	Q9NQ29;Q9NQ29	Q9NQ29;Q9NQ29	Putative RNA-binc	LUC7L
28.3761	26.9485	27.6752	28.0808	27.3642	27.9093	27.2706	27.3184	25.9203	28.7128	28.2232	27.5108	Q9Y383;A0A0A6Y	Q9Y383;A0A0A6Y	Putative RNA-binc	LUC7L2;C7orf55-LI
26.5243	24.4352	25.729	26.2357	25.4723	25.5466	24.274	24.7971	NaN	26.1797	25.7846	25.1948	O95232;J3KPP4;D	O95232;J3KPP4	Luc7-like protein 3	LUC7L3
25.9679	26.6085	26.4044	25.8361	26.4941	26.2826	26.6634	27.1735	26.718	25.8309	25.926	26.3152	Q86V48;Q86V48-:	Q86V48;Q86V48-:	Leucine zipper prc	LUZP1
23.8623	24.6071	23.5694	23.1931	23.7726	NaN	NaN	NaN	23.5157	23.639	22.4201	NaN	Q9BS40	Q9BS40	Latexin	LXN
27.02	25.9802	26.1935	27.491	27.2438	26.0846	25.3187	24.855	24.2788	28.019	27.196	26.3315	Q9NX58	Q9NX58	Cell growth-regula	LYAR
27.7285	27.2705	27.9233	27.7729	26.9842	27.8444	27.6644	26.9127	27.5067	28.2141	27.6477	27.4326	P07948;E5RJ37;P(	P07948	Tyrosine-protein I	LYN
NaN	NaN	23.0522	NaN	NaN	NaN	NaN	NaN	NaN	NaN	NaN	NaN	P07948-2	P07948-2	Tyrosine-protein I	LYN
22.7623	23.4334	23.222	23.0118	23.6189	23.9135	23.0819	NaN	NaN	NaN	NaN	NaN	O75608;E5RGR0;(	O75608;E5RGR0;(	Acyl-protein thioe	LYPLA1
25.2562	24.984	24.686	24.5142	24.4737	NaN	25.4778	NaN	NaN	25.4372	24.9575	NaN	O95372;Q5QPQ0;	O95372;Q5QPQ0;	Acyl-protein thioe	LYPLA2
23.3594	23.711	23.6958	22.9889	23.9065	23.7417	NaN	NaN	NaN	23.2376	23.2024	NaN	Q5VWZ2;Q5VWZ2	Q5VWZ2;Q5VWZ2	Lysophospholipas	LYPLAL1
22.9601	NaN	NaN	NaN	NaN	NaN	NaN	NaN	NaN	NaN	NaN	NaN	Q5U5X0	Q5U5X0	Complex III assem	LYRM7
NaN	NaN	NaN	NaN	NaN	NaN	NaN	NaN	24.6436	NaN	NaN	24.037	P61626;F8VV32;A	P61626;F8VV32;A	Lysozyme C;Lysoz	LYZ
24.1502	24.146	24.0719	23.7161	23.8908	23.8689	24.1269	24.4681	NaN	23.5935	24.3651	NaN	Q9NQ48;Q9NQ48	Q9NQ48;Q9NQ48	Leucine zipper tra	LZTFL1
27.796	27.1858	27.4431	28.2612	27.222	27.682	27.108	27.4811	26.4448	27.061	26.6081	26.3069	P20645;F5GX30;H	P20645;F5GX30	Cation-dependent	M6PR
22.8485	23.076	23.6621	23.4248	22.8208	23.6354	NaN	NaN	NaN	NaN	NaN	23.3073	A0A0A6YYJ5;O948	A0A0A6YYJ5		MACF1
28.6867	28.7145	28.8661	28.6124	28.9066	28.9625	29.126	29.5883	29.3036	28.9133	28.834	29.2549	H3BPE1;H3BQK9;(	H3BPE1;H3BQK9;(	Microtubule-actin	MACF1
23.4767	NaN	NaN	23.6609	NaN	NaN	NaN	NaN	NaN	NaN	NaN	NaN	Q9BQ69	Q9BQ69	O-acetyl-ADP-ribc	MACROD1
NaN	NaN	NaN	NaN	26.5039	NaN	NaN	NaN	NaN	NaN	NaN	NaN	Q9Y6D9;C9JJ38;Q	Q9Y6D9;C9JJ38;Q	Mitotic spindle as	MAD1L1
25.6006	25.1647	25.2801	25.4054	24.9163	25.1507	25.5656	NaN	24.7566	25.6138	25.4151	25.0628	Q13257;Q13257-2	Q13257	Mitotic spindle as	MAD2L1
25.4203	24.3283	25.0677	24.8161	24.3052	24.5962	24.8656	NaN	NaN	24.5014	24.7363	NaN	Q8WXG6-3;Q8WX	Q8WXG6-3;Q8WX	MAP kinase-activa	MADD
23.7209	23.0319	23.3504	23.9449	23.1356	23.3363	NaN	NaN	NaN	23.427	23.2701	NaN	Q7L5Y9;B4DVN3;I	Q7L5Y9;B4DVN3;I	Macrophage eryth	MAEA
23.6736	24.0666	22.8449	NaN	23.1558	22.9614	NaN	NaN	NaN	NaN	NaN	NaN	Q9ULX9;B0QY70;(	Q9ULX9;B0QY70;(	Transcription fact	MAFF
26.9336	26.3749	26.6189	27.1105	26.3727	26.542	26.0431	25.421	25.5201	26.1058	26.6132	25.6829	Q9UNF1;Q5H909;	Q9UNF1;Q5H909;	Melanoma-associ	MAGED2
NaN	NaN	NaN	22.2605	NaN	NaN	NaN	NaN	NaN	NaN	NaN	NaN	Q96QZ7;Q96QZ7-	Q96QZ7;Q96QZ7-	Membrane-associ	MAGI1
27.8126	27.4799	27.471	27.738	27.74	27.651	26.5743	26.8363	26.6402	26.9781	27.595	27.8474	P61326;P61326-2	P61326;P61326-2	Protein mago nas	MAGOH
24.2769	24.4489	24.856	24.8111	24.6544	NaN	24.9093	25.2839	26.3109	25.1742	25.0079	25.3333	Q9H0U3;A0A087V	Q9H0U3;A0A087V	Magnesium transp	MAGT1
NaN	NaN	21.9941	22.4034	21.5139	NaN	NaN	NaN	NaN	21.2313	21.4732	NaN	Q9BXY0;H0YBV6	Q9BXY0;H0YBV6	Protein MAK16 hc	MAK16
22.7669	NaN	22.8298	22.8357	NaN	NaN	NaN	NaN	NaN	NaN	NaN	NaN	O60476;H0Y7H1;I	O60476;H0Y7H1	Mannosyl-oligosa	MAN1A2
NaN	NaN	NaN	22.2628	NaN	NaN	NaN	NaN	22.7837	NaN	NaN	NaN	Q9UKM7;H0YG20	Q9UKM7;H0YG20	Endoplasmic retic	MAN1B1
26.7883	26.3004	26.5186	26.7749	26.3514	26.6302	27.3332	25.7974	25.9561	26.2411	25.6819	25.7056	Q16706	Q16706	Alpha-mannosida	MAN2A1

24.359	25.4611	24.4605	24.064	24.7589	24.1091	25.2141	25.8822	25.2892	24.3745	23.4974	24.3498	O00754;O00754-2	O00754;O00754-2	Lysosomal alpha-r	MAN2B1
26.1779	27.0473	26.4408	26.0355	26.9931	26.3915	26.5574	28.2454	27.5651	26.4305	26.5895	27.0843	P55145;A8K878;H	P55145;A8K878	Mesencephalic as	MANF
NaN	NaN	NaN	NaN	NaN	NaN	NaN	NaN	31.6676	NaN	28.0665	29.224	P78559;E9PGC8;P	P78559;E9PGC8;P	Microtubule-asso	MAP1A
27.9761	28.101	27.8775	27.046	27.1469	26.8765	27.5868	27.9209	27.9489	26.8666	26.7411	26.9743	P46821;D6RA32;D	P46821	Microtubule-asso	MAP1B
28.4475	27.7709	28.2952	27.2672	26.5082	27.4217	28.976	28.5378	28.6713	28.231	27.1904	27.9618	Q9GZQ8;A6NCE7;	Q9GZQ8;A6NCE7;	Microtubule-asso	MAP1LC3B;MAP1I
26.8289	26.0714	25.8806	26.334	25.9938	25.8747	25.7705	25.6754	25.8998	26.4306	25.8257	25.645	Q66K74;Q66K74-2	Q66K74;Q66K74-2	Microtubule-asso	MAP1S
28.9736	28.7112	28.7903	28.8661	28.6417	28.8428	28.6439	28.1005	28.5346	29.1736	29.4136	28.4535	Q02750;Q02750-2	Q02750;Q02750-2	Dual specificity m	MAP2K1
27.7721	27.3411	27.5263	27.5487	27.5486	27.6155	27.559	27.3328	27.5143	28.1141	28.4566	27.3959	P36507;G5E9C7;N	P36507;G5E9C7	Dual specificity m	MAP2K2
27.0822	26.1887	26.4214	26.9898	26.4519	26.2726	25.949	26.7464	26.6861	26.5059	26.4782	26.225	P46734;P46734-3	P46734;P46734-3	Dual specificity m	MAP2K3
23.8775	23.6593	23.7695	24.0897	23.7565	24.0618	NaN	NaN	NaN	23.9592	23.926	23.7177	P45985;P45985-2	P45985;P45985-2	Dual specificity m	MAP2K4
NaN	NaN	NaN	21.3959	NaN	NaN	NaN	NaN	NaN	NaN	NaN	NaN	Q13163;A6NK28;(	Q13163;A6NK28;(	Dual specificity m	MAP2K5
22.1731	NaN	NaN	21.5485	NaN	NaN	NaN	NaN	NaN	NaN	NaN	NaN	Q9Y6R4;Q9Y6R4-2	Q9Y6R4;Q9Y6R4-2	Mitogen-activatec	MAP3K4
23.1001	NaN	23.1007	NaN	NaN	NaN	NaN	22.8432	NaN	NaN	22.6708	NaN	O43318;O43318-2	O43318;O43318-2	Mitogen-activatec	MAP3K7;DKFZp58
27.2507	28.0473	27.067	26.1566	26.7231	26.1862	26.1785	27.823	27.3903	25.137	25.9706	26.6043	P27816;E7EVA0;P	P27816;E7EVA0;P	Microtubule-asso	MAP4
23.5863	23.2263	23.194	23.3076	23.1269	23.1975	22.9171	23.0907	23.2338	23.3316	23.1621	23.1546	Q8IVH8;Q8IVH8-3	Q8IVH8;Q8IVH8-3	Mitogen-activatec	MAP4K3
28.781	28.8679	29.3542	28.7866	28.6257	29.3526	28.8922	28.6059	29.2403	29.1786	28.9207	28.9495	G5E948;A0A0D9S	G5E948;A0A0D9S	Mitogen-activatec	MAP4K4
27.1393	26.0102	26.4383	26.9264	26.2084	26.2448	25.7464	25.3875	26.6831	26.8216	27.1426	25.6515	Q9Y4K4;A0A0A0N	Q9Y4K4;A0A0A0N	Mitogen-activatec	MAP4K5
26.0215	25.6726	26.1707	25.6033	25.8746	25.865	NaN	NaN	NaN	NaN	NaN	NaN	Q3KQU3;Q3KQU3	Q3KQU3;Q3KQU3	MAP7 domain-cor	MAP7D1
24.5289	24.5354	24.6177	24.7254	24.8697	25.0768	NaN	NaN	NaN	NaN	NaN	NaN	Q8IWC1;Q8IWC1-	Q8IWC1;Q8IWC1-	MAP7 domain-cor	MAP7D3
27.4111	27.2728	27.2366	27.1033	27.1852	27.3709	27.2166	26.3147	27.5887	27.4901	27.5042	27.7701	P28482;P28482-2	P28482;P28482-2	Mitogen-activatec	MAPK1
20.9649	NaN	NaN	NaN	NaN	NaN	NaN	NaN	NaN	22.0678	NaN	NaN	O15264;O15264-2	O15264	Mitogen-activatec	MAPK13
26.3932	26.3032	26.2693	26.4504	26.3028	26.5014	26.4854	26.215	26.1694	26.3687	26.4824	26.6664	Q16539;Q16539-3	Q16539;Q16539-3	Mitogen-activatec	MAPK14
NaN	NaN	NaN	NaN	NaN	NaN	24.822	NaN	NaN	NaN	NaN	NaN	Q8NDC0	Q8NDC0	MAPK-interacting	MAPK1IP1L
25.9629	25.019	25.8482	25.6526	25.1641	25.8058	25.4919	24.9019	24.7724	26.6577	26.6189	25.5004	P27361;P27361-3	P27361;P27361-3	Mitogen-activatec	MAPK3
25.6179	23.8197	24.6	25.5001	23.6973	24.4735	24.2879	NaN	23.4377	24.1049	23.9217	NaN	P45983-3;P45983	P45983-3;P45983	Mitogen-activatec	MAPK8;MAPK10
23.5262	23.413	23.5076	23.7462	23.8446	NaN	NaN	NaN	24.9084	22.7105	NaN	24.4543	Q9BPZ7;Q9BPZ7-2	Q9BPZ7;Q9BPZ7-2	Target of rapamyc	MAPKAP1
21.7505	NaN	NaN	21.8238	NaN	NaN	NaN	NaN	NaN	22.024	22.302	NaN	P49137;P49137-2	P49137;P49137-2	MAP kinase-activa	MAPKAPK2
24.2189	23.2078	23.9006	24.0486	23.8343	NaN	NaN	23.6176	NaN	24.3061	24.0833	NaN	Q16644;C9J8E1;C	Q16644;C9J8E1	MAP kinase-activa	MAPKAPK3
29.2523	28.9762	29.0636	29.0289	28.5002	28.7882	28.6598	28.3639	28.3269	28.4683	28.3315	28.8183	Q15691	Q15691	Microtubule-asso	MAPRE1
24.7379	25.19	25.4002	25.5429	25.4128	25.4621	24.488	NaN	25.2614	25.7175	25.693	25.4641	Q15555;Q15555-4	Q15555;Q15555-4	Microtubule-asso	MAPRE2
29.7194	29.8194	29.8823	28.8931	28.9494	29.2429	30.2157	29.4892	29.5428	29.7474	29.4652	29.8384	P29966;A0A087W	P29966;A0A087W	Myristoylated alar	MARCKS
27.48	28.9274	29.0915	27.8173	29.0112	29.1401	28.9359	28.7743	29.2587	29.2814	29.3736	29.6417	P49006	P49006	MARCKS-related p	MARCKSL1
26.7466	26.9397	27.2616	26.9777	27.2348	27.4675	27.1726	26.5009	26.9993	27.5527	27.5887	27.673	E9PC69;Q7KZI7-1	E9PC69;Q7KZI7-1	Serine/threonine-	MARK2
23.9258	24.1337	24.3036	24.2329	24.1363	24.7858	24.0334	24.8139	NaN	24.3882	24.6312	24.7406	P27448;P27448-3	P27448;P27448-3	MAP/microtubule	MARK3
29.8034	28.4875	29.12	29.892	29.0764	29.2877	28.2029	28.6909	28.6448	29.0245	29.103	28.2499	P56192;P56192-2	P56192;P56192-2	Methionine--tRNA	MARS
28.2691	28.5	28.4468	28.3905	28.6794	28.7064	28.3158	28.4555	28.1474	29.3839	29.7695	29.5416	P31153;P31153-2	P31153;P31153-2	S-adenosylmethic	MAT2A
26.3512	26.5352	26.0681	26.1029	26.421	26.0498	26.4085	26.9308	26.1686	27.0021	26.6082	26.7442	Q9NZL9;H7C0X7;(	Q9NZL9;H7C0X7;(	Methionine adenc	MAT2B
NaN	NaN	NaN	22.4478	NaN	NaN	NaN	NaN	NaN	NaN	NaN	NaN	Q9NZL9-2;Q9NZL9	Q9NZL9-2;Q9NZL9	Methionine adenc	MAT2B
29.5423	28.9109	29.1986	29.6527	29.2841	29.3675	27.6939	29.0974	28.1739	29.2433	29.5117	29.2837	P43243;A8MXP9;/	P43243;A8MXP9;/	Matrin-3	MATR3
23.9889	24.0532	23.5557	23.8099	NaN	23.8634	NaN	NaN	NaN	NaN	24.3882	NaN	Q7Z434;Q7Z434-5	Q7Z434;Q7Z434-5	Mitochondrial ant	MAVS
26.3692	25.2936	25.7148	26.096	25.3717	25.0112	23.3104	24.7096	25.0554	23.7155	24.8686	23.7018	Q8N884;Q8N884-	Q8N884;Q8N884-	Cyclic GMP-AMP s	MB21D1
23.1788	22.825	23.0089	23.8496	23.603	22.8771	NaN	NaN	NaN	22.929	23.2664	NaN	O95983;O95983-2	O95983;O95983-2	Methyl-CpG-bindi	MBD3
24.9886	24.7327	25.0236	25.4809	25.3431	25.3982	25.8061	24.689	25.7442	25.7491	25.831	26.1106	Q68D91	Q68D91	Metallo-beta-lact	MBLAC2
25.2052	NaN	NaN	25.2928	23.9549	NaN	24.4282	NaN	NaN	23.6986	24.0605	NaN	Q9NR56;C9JP00;A	Q9NR56;C9JP00;A	Muscleblind-like p	MBNL1;MBNL3
25.5266	25.3722	25.6544	25.6481	25.1439	25.3848	25.6288	25.12	NaN	25.477	25.2108	24.9084	Q96N66;Q96N66-	Q96N66;Q96N66-	Lysophospholipid	MBOAT7
24.2683	25.395	25.5931	24.5054	25.0623	25.7818	24.5699	NaN	NaN	NaN	NaN	NaN	J3QQK6;P02686;P	J3QQK6;P02686;P	Myelin basic prot	MBP
NaN	NaN	NaN	NaN	NaN	NaN	21.7834	NaN	NaN	22.6748	21.6132	NaN	Q14703;H3BV53	Q14703	Membrane-bounc	MBTPS1
26.8253	26.8696	27.2273	26.7489	26.8269	27.2414	28.0754	27.323	26.5358	28.39	27.4296	26.8221	P43121;P43121-2	P43121	Cell surface glycop	MCAM
24.9239	24.4934	24.4184	24.9209	24.3266	23.3264	NaN	NaN	23.777	23.7979	23.731	NaN	Q96RQ3;F5GYT8;I	Q96RQ3;F5GYT8;I	Methylcrotonoyl-(	MCCC1
24.1992	25.2484	24.7225	24.8165	25.5686	24.5589	23.6934	25.3549	25.2543	23.5965	24.0444	24.2212	Q9HCC0;Q9HCC0-	Q9HCC0;Q9HCC0-	Methylcrotonoyl-(	MCCC2
29.159	27.3385	28.491	28.755	28.1318	28.7573	26.602	26.2086	26.6041	29.1481	28.7866	28.1001	P49736;H0Y8E6	P49736;H0Y8E6	DNA replication li	MCM2
28.9997	27.7548	28.5322	29.0572	28.2253	28.6613	26.6622	27.6644	26.9277	28.9073	28.7767	28.0506	P25205;P25205-2	P25205;P25205-2	DNA replication li	MCM3
28.7376	27.9462	28.4787	28.7738	28.5841	28.6321	26.3173	27.0884	26.0731	28.7406	28.8303	28.304	P33991;E5RG31;E	P33991	DNA replication li	MCM4
28.6704	27.6624	28.4664	28.6784	28.2996	28.6884	26.1266	26.7011	24.5838	28.7185	29.0574	28.1578	P33992;B1AHB1;E	P33992;B1AHB1	DNA replication li	MCM5
28.1983	26.9247	27.8032	28.2745	27.5424	27.8626	25.875	26.5828	26.4183	28.5069	28.1559	27.658	Q14566	Q14566	DNA replication li	MCM6
29.207	28.5581	29.4764	29.586	29.274	29.6846	27.4395	28.5563	27.7566	29.4575	29.6877	29.3167	P33993;P33993-3	P33993;P33993-3	DNA replication li	MCM7
27.0664	26.2479	26.3406	26.8674	26.7622	26.48	25.6688	25.6958	23.9741	26.575	26.4448	26.1923	Q9BTE3;Q9BTE3-2	Q9BTE3;Q9BTE3-2	Mini-chromosome	MCMBP
27.674	27.8569	27.566	27.6356	27.5571	27.3444	28.0801	27.0339	26.1555	27.0616	27.2404	27.3075	Q9ULC4;Q9ULC4-	Q9ULC4;Q9ULC4-	Malignant T-cell-a	MCTS1
24.9822	NaN	24.4987	24.6704	NaN	NaN	NaN	NaN	NaN	NaN	NaN	NaN	Q14676;Q14676-3	Q14676;Q14676-3	Mediator of DNA	MDC1
29.1871	29.8421	29.4607	28.7747	29.6215	29.4221	30.1046	29.5438	29.4323	29.5541	29.4832	29.6991	P40925;P40925-3	P40925;P40925-3	Malate dehydroge	MDH1

28.8826	29.7269	28.7986	28.3721	29.5174	28.7297	28.6344	29.6733	30.5002	28.582	28.8954	29.7065	P40926;G3XAL0;P	P40926;G3XAL0;P	Malate dehydrog	MDH2
22.0951	NaN	NaN	NaN	NaN	NaN	NaN	NaN	NaN	NaN	NaN	NaN	P21741;E9PPJ5;E9	P21741;E9PPJ5;E9	Midkine	MDK
25.0669	NaN	23.7155	25.4693	NaN	NaN	NaN	NaN	NaN	22.6723	22.9953	NaN	Q9NU22;Q5T795;	Q9NU22	Midasin	MDN1
24.2507	25.5383	24.9155	24.3764	25.1547	24.7952	25.4665	24.8542	NaN	24.6582	24.6059	25.1582	P48163;P48163-2	P48163;P48163-2	NADP-dependent	ME1
23.6003	23.0914	23.0425	23.7679	23.2142	23.1065	NaN	NaN	NaN	NaN	NaN	NaN	P23368;P23368-2	P23368;P23368-2	NAD-dependent r	ME2
NaN	23.4282	23.4311	22.9366	23.1651	NaN	22.9158	24.3863	NaN	23.1834	NaN	NaN	Q16626	Q16626	Male-enhanced a	MEA1
23.8832	NaN	NaN	23.5509	23.7557	NaN	NaN	NaN	NaN	NaN	23.4456	NaN	Q15648;Q15648-3	Q15648;Q15648-3	Mediator of RNA	MED1
23.6367	24.1631	23.7029	23.8575	24.3327	23.8197	NaN	NaN	NaN	NaN	24.2541	NaN	Q9P086;I3L3E8;I3	Q9P086	Mediator of RNA	MED11
NaN	NaN	NaN	20.3558	NaN	NaN	NaN	NaN	NaN	NaN	NaN	NaN	Q93074;Q7Z3Z5;C	Q93074;Q7Z3Z5;C	Mediator of RNA	MED12;TNRC11
23.4212	NaN	NaN	23.3992	NaN	NaN	NaN	NaN	NaN	NaN	NaN	NaN	O60244;H7C3E5;H	O60244	Mediator of RNA	MED14
22.7555	NaN	22.6704	23.7049	23.0272	23.0614	NaN	NaN	NaN	22.5923	NaN	NaN	Q96RN5;G3V1P5;	Q96RN5;G3V1P5;	Mediator of RNA	MED15
22.4791	NaN	NaN	22.7088	NaN	NaN	NaN	NaN	NaN	22.0449	NaN	NaN	Q9Y2X0;Q9Y2X0-2	Q9Y2X0;Q9Y2X0-2	Mediator of RNA	MED16
24.0219	23.6728	23.1158	24.0936	23.5524	NaN	NaN	NaN	NaN	23.0997	23.4522	NaN	Q9NVC6;E9PJZ4;E	Q9NVC6	Mediator of RNA	MED17
24.6541	24.5612	24.4842	24.6058	24.9834	24.6144	24.103	NaN	NaN	24.59	24.8045	NaN	Q9BUE0	Q9BUE0	Mediator of RNA	MED18
NaN	NaN	NaN	20.4892	NaN	NaN	NaN	NaN	NaN	NaN	NaN	NaN	A0JLT2;J3KR33;A0	A0JLT2;J3KR33;A0	Mediator of RNA	MED19
24.5352	24.3693	24.3571	24.552	24.6301	24.5051	NaN	NaN	NaN	NaN	NaN	NaN	Q9H944;B7ZBQ3;A	Q9H944;B7ZBQ3;A	Mediator of RNA	MED20
NaN	NaN	NaN	21.8323	NaN	NaN	NaN	NaN	NaN	NaN	NaN	NaN	Q13503;F5H872	Q13503;F5H872	Mediator of RNA	MED21
24.1831	24.2647	23.9545	24.768	24.1492	NaN	23.2666	NaN	NaN	23.8704	24.0559	NaN	Q15528;E9PGW7;	Q15528;E9PGW7;	Mediator of RNA	MED22
23.2356	21.0736	22.1167	23.3847	22.0581	23.0616	NaN	NaN	NaN	21.7656	21.3187	NaN	Q9ULK4;Q5JWT2;	Q9ULK4;Q5JWT2;	Mediator of RNA	MED23
21.4574	NaN	21.6511	22.1339	NaN	NaN	NaN	NaN	NaN	NaN	NaN	NaN	O75448;F5GY88;A	O75448;F5GY88;A	Mediator of RNA	MED24
NaN	NaN	22.9449	NaN	NaN	NaN	NaN	NaN	NaN	NaN	NaN	NaN	Q6P2C8;Q6P2C8-2	Q6P2C8;Q6P2C8-2	Mediator of RNA	MED27
21.7693	NaN	NaN	NaN	NaN	21.8614	NaN	NaN	NaN	NaN	NaN	NaN	Q9NX70;B4DUA7;	Q9NX70;B4DUA7;	Mediator of RNA	MED29;IXL
22.65	NaN	NaN	NaN	NaN	NaN	NaN	NaN	NaN	NaN	NaN	NaN	Q96HR3;Q96HR3-	Q96HR3;Q96HR3-	Mediator of RNA	MED30
22.28	NaN	NaN	21.9426	NaN	NaN	NaN	NaN	NaN	NaN	NaN	NaN	Q9Y3C7;I3L2J1;I3I	Q9Y3C7;I3L2J1	Mediator of RNA	MED31
NaN	21.2157	NaN	22.2571	NaN	NaN	NaN	NaN	NaN	21.4098	NaN	NaN	Q9NPJ6;Q5T911;C	Q9NPJ6;Q5T911	Mediator of RNA	MED4
22.8552	22.6759	22.4483	22.8624	22.9929	22.6239	NaN	NaN	NaN	22.7575	NaN	NaN	Q96G25;Q96G25-	Q96G25;Q96G25-	Mediator of RNA	MED8
NaN	NaN	NaN	22.381	NaN	NaN	NaN	NaN	NaN	NaN	NaN	NaN	Q9NWA0	Q9NWA0	Mediator of RNA	MED9
22.2029	NaN	22.0884	NaN	NaN	NaN	NaN	NaN	NaN	NaN	NaN	NaN	Q14680;Q14680-7	Q14680;Q14680-7	Maternal embryo	MELK
24.2838	24.5183	24.2424	24.0552	24.6597	24.5369	24.4062	NaN	24.6303	25.0132	24.6699	24.9757	Q9Y316;Q9Y316-3	Q9Y316;Q9Y316-3	Protein MEMO1	MEMO1
24.8827	24.3413	24.5275	25.5408	24.9878	24.7752	NaN	NaN	NaN	24.3486	24.6832	24.0755	Q7L2J0;Q7L2J0-2	Q7L2J0;Q7L2J0-2	7SK snRNA methy	MEPCE
NaN	NaN	NaN	25.3838	NaN	NaN	NaN	NaN	NaN	NaN	NaN	NaN	Q12866;E9PHX8;H	Q12866;E9PHX8;H	Tyrosine-protein k	MERTK
23.5966	24.3026	23.8258	23.8216	24.2419	23.5464	24.4305	25.3398	24.8104	23.9186	24.2922	NaN	Q14696;H0YLI4;Q	Q14696	LDLR chaperone M	MESDC2
25.7268	25.6344	25.8575	25.3911	25.0564	25.6013	27.6598	25.97	25.7353	25.8511	24.9899	24.9901	P08581;P08581-2	P08581;P08581-2	Hepatocyte growt	MET
25.2504	24.9881	24.9224	25.4641	24.8351	25.2924	24.3224	NaN	NaN	24.9361	25.7451	NaN	P53582;H0Y955;H	P53582;H0Y955	Methionine amin	METAP1
26.9445	26.1246	26.5054	26.7659	25.8148	26.2309	26.6027	26.5172	25.9629	26.439	26.6682	26.543	P50579;P50579-3	P50579;P50579-3	Methionine amin	METAP2
NaN	NaN	NaN	NaN	NaN	NaN	NaN	NaN	NaN	20.7471	NaN	NaN	Q641Q3;Q641Q3-	Q641Q3;Q641Q3-	Meteorin-like pro	METRNL
23.7349	22.8168	23.0073	23.654	23.0336	22.8841	NaN	NaN	NaN	23.0104	22.9107	23.2169	Q8N6R0;Q8N6R0-	Q8N6R0;Q8N6R0-	Methyltransferas	METTL13
24.1023	NaN	24.025	24.0972	NaN	24.0185	NaN	NaN	NaN	NaN	NaN	NaN	Q6P1Q9;Q6P1Q9-	Q6P1Q9;Q6P1Q9-	Methyltransferas	METTL2B
23.2522	22.8263	23.4788	23.3335	23.3657	NaN	NaN	NaN	NaN	23.5074	24.0318	NaN	Q86U44;F5H6D8;I	Q86U44;F5H6D8;I	N6-adenosine-me	METTL3
NaN	NaN	24.6732	23.9412	NaN	NaN	NaN	NaN	NaN	NaN	23.1253	NaN	P55082;P55082-2	P55082;P55082-2	Microfibril-associa	MFAP3;MFAP3L
NaN	23.4262	NaN	21.9679	22.5639	NaN	22.6361	NaN	NaN	NaN	NaN	22.6836	P55083;K7ES70;P5	P55083;K7ES70;P5	Microfibril-associa	MFAP4
32.0223	32.7037	32.9592	31.6965	32.3618	32.9887	35.4069	34.3898	33.71	33.4996	32.6407	32.0428	Q08431;F5GZN3;C	Q08431;F5GZN3;C	Lactadherin;Lacta	MFGE8
25.9179	26.9775	28.326	26.167	26.7646	27.7614	23.2808	NaN	24.4147	23.5081	24.0323	24.4651	P08582;P08582-2	P08582	Melanotransferrin	MFI2
NaN	NaN	NaN	NaN	NaN	NaN	NaN	NaN	21.4972	NaN	NaN	NaN	O95140;O95140-2	O95140;O95140-2	Mitofusin-2	MFN2
NaN	NaN	NaN	NaN	NaN	NaN	NaN	NaN	NaN	NaN	NaN	23.7491	Q9H3U5;Q9H3U5	Q9H3U5;Q9H3U5	Major facilitator s	MFSD1
NaN	NaN	NaN	21.6414	NaN	NaN	NaN	NaN	NaN	NaN	NaN	NaN	Q6ZSS7;H7C284	Q6ZSS7;H7C284	Major facilitator s	MFSD6
NaN	NaN	22.3785	22.5172	NaN	NaN	NaN	NaN	NaN	NaN	NaN	NaN	Q8NHS3;E7ERQ4	Q8NHS3;E7ERQ4	Major facilitator s	MFSD8
24.422	24.5185	25.0275	24.9843	24.8666	24.6306	24.7487	NaN	24.7023	24.3084	23.3061	24.4978	P26572;D6RF69;D	P26572	Alpha-1,3-mannos	MGAT1
23.5523	23.2028	23.201	23.4725	NaN	23.2397	24.1988	NaN	NaN	23.6282	23.3977	NaN	Q10469	Q10469	Alpha-1,6-mannos	MGAT2
NaN	NaN	NaN	NaN	NaN	NaN	NaN	NaN	NaN	22.5777	21.879	NaN	Q9UQ53;Q9UQ53	Q9UQ53;Q9UQ53	Alpha-1,3-mannos	MGAT4B
25.2402	24.7428	24.7985	25.0403	24.8376	24.7634	24.5671	25.2831	24.849	24.7893	24.8777	24.9433	O60502;O60502-4	O60502;O60502-4	Protein O-GlcNAc	MGEA5
23.3644	22.8391	23.2581	22.7656	22.3357	22.7678	NaN	NaN	NaN	22.9119	NaN	NaN	Q99685;A0A0C4D	Q99685;A0A0C4D	Monoglyceride lip	MGLL
NaN	NaN	NaN	NaN	22.6461	NaN	NaN	NaN	NaN	NaN	NaN	NaN	Q9BQP7;Q5QPE7;	Q9BQP7;Q5QPE7;	Mitochondrial ger	MGME1
NaN	NaN	NaN	22.2598	NaN	NaN	NaN	NaN	NaN	NaN	NaN	NaN	O60291;O60291-3	O60291;O60291-3	E3 ubiquitin-prote	MGRN1
NaN	23.028	23.6806	22.4339	23.9926	NaN	22.7534	24.7064	NaN	NaN	23.0239	NaN	P10620;F5H7F6;F5	P10620;F5H7F6;F5	Microsomal glutat	MGST1
NaN	NaN	NaN	NaN	NaN	NaN	23.6703	NaN	NaN	NaN	NaN	NaN	Q99735;Q99735-2	Q99735;Q99735-2	Microsomal glutat	MGST2
NaN	NaN	25.8279	25.2437	NaN	NaN	25.7732	NaN	NaN	NaN	NaN	NaN	Q5JRA6;Q5JRA6-2	Q5JRA6;Q5JRA6-2	Melanoma inhibit	MIA3
24.4061	23.8343	23.8306	23.9305	23.3873	23.8655	23.8162	NaN	NaN	23.9224	NaN	NaN	Q86YT6	Q86YT6	E3 ubiquitin-prote	MIB1
NaN	NaN	NaN	NaN	NaN	NaN	NaN	NaN	NaN	21.7676	NaN	NaN	Q29983;Q29983-2	Q29983;Q29983-2	MHC class I polyp	MICA
24.7515	24.1485	23.477	23.776	23.6726	23.5696	27.116	26.9979	NaN	25.3089	23.705	NaN	Q96QC4;A0A024F	Q96QC4;A0A024RCL3;A0A0G2JK11		MICA

22.3251	22.4843	22.9944	22.0223	NaN	NaN	23.0218	NaN	NaN	23.1174	23.3881	NaN	Q8TDZ2;Q8TDZ2-4	Q8TDZ2;Q8TDZ2-4	Protein-methionir	MICAL1
23.0103	22.3719	22.2706	22.5336	22.4035	22.4641	NaN	NaN	NaN	NaN	22.7989	NaN	O94851;O94851-5	O94851;O94851-5	Protein-methionir	MICAL2
NaN	NaN	21.1504	21.1705	NaN	NaN	NaN	NaN	NaN	NaN	NaN	NaN	Q7RTP6;Q7RTP6-5	Q7RTP6;Q7RTP6-5	Protein-methionir	MICAL3
NaN	NaN	NaN	21.4782	NaN	NaN	NaN	NaN	NaN	NaN	NaN	NaN	Q29980;A0A0G2JI	Q29980;A0A0G2JI	MHC class I polyp	MICB
20.8335	NaN	NaN	NaN	NaN	NaN	NaN	NaN	NaN	NaN	NaN	NaN	Q9BPX6;F6XEV2;A	Q9BPX6;F6XEV2;A	Calcium uptake pr	MICU1
NaN	NaN	NaN	21.7891	NaN	NaN	NaN	NaN	NaN	NaN	NaN	NaN	Q8IYU8	Q8IYU8	Calcium uptake pr	MICU2
25.2882	24.7399	24.8848	25.5895	24.8201	24.772	NaN	NaN	23.9891	24.9365	24.224	NaN	O15344;C9J453;A	O15344;C9J453;A	E3 ubiquitin-prote	MID1
28.642	29.9278	29.0962	28.5216	29.8295	29.1474	29.0265	29.5649	30.2216	28.8074	29.4355	30.4978	P14174	P14174	Macrophage migr	MIF
24.1347	23.5612	23.3307	24.0941	23.5085	23.4986	NaN	NaN	NaN	23.7887	24.5763	NaN	Q8IUF8;Q8IUF8-4	Q8IUF8;Q8IUF8-4	Bifunctional lysine	MINA
25.0334	25.5877	26.2169	25.1444	25.6113	26.2599	25.2064	NaN	25.2082	26.2272	26.0668	26.3694	Q8N4C8;Q8N4C8-	Q8N4C8;Q8N4C8-	Misshapen-like kir	MINK1
NaN	NaN	NaN	NaN	NaN	NaN	22.1693	NaN	NaN	NaN	NaN	NaN	Q9UNW1;Q9UNW	Q9UNW1;Q9UNW	Multiple inositol p	MINPP1
NaN	NaN	NaN	20.9386	NaN	NaN	NaN	NaN	NaN	NaN	NaN	NaN	Q9NXC5;Q9NXC5-	Q9NXC5;Q9NXC5-	WD repeat-contai	MIOS
26.9497	27.7308	27.5247	26.6197	26.9698	27.4451	28.2272	27.5668	28.319	26.8681	26.5888	27.1299	Q8WV92;B8ZZL5;I	Q8WV92;B8ZZL5;I	MIT domain-conta	MITD1
28.0756	28.6008	27.7942	28.035	28.1248	27.8633	26.6686	27.9658	26.443	26.9809	27.6844	27.6634	P46013;A0A087W	P46013;A0A087W	Antigen KI-67	MKI67
NaN	NaN	NaN	22.6205	NaN	NaN	NaN	NaN	NaN	NaN	NaN	NaN	P46013-2	P46013-2	Antigen KI-67	MKI67
21.8231	NaN	21.9759	22.1426	22.0546	NaN	NaN	NaN	NaN	21.8623	22.0408	NaN	Q9UL63;C9J7E8;Q	Q9UL63;C9J7E8;Q	Muskelin	MKLN1
25.6933	25.4161	25.7477	25.8754	25.2469	25.6911	25.6415	25.1683	25.9966	25.915	25.142	25.4757	Q14165;F5H1S8;H	Q14165;F5H1S8;H	Malectin	MLEC
23.771	23.4259	23.3221	NaN	NaN	NaN	NaN	NaN	NaN	NaN	NaN	NaN	Q15773;F5H0Y3;F	Q15773;F5H0Y3	Myeloid leukemia	MLF2
22.328	NaN	NaN	22.5682	NaN	NaN	NaN	NaN	NaN	NaN	NaN	NaN	P40692;H0Y818;P	P40692;H0Y818;P	DNA mismatch re	MLH1
26.2606	25.4757	25.5934	25.7053	25.7437	25.1645	26.0421	24.0627	26.8989	27.0914	26.9965	26.9132	Q8NB16;I3L2T9;Q	Q8NB16;I3L2T9	Mixed lineage kin	MLKL
26.5562	27.2182	27.736	26.5898	27.1795	27.6239	27.1311	24.8572	25.9019	27.9215	27.2942	27.0124	J3KN01;P55196;P	J3KN01;P55196;P	Afadin	MLLT4
24.1343	24.11	24.0447	23.9877	24.0888	NaN	24.0794	NaN	23.844	24.391	24.3645	24.2295	Q9BVC4;H3BM50	Q9BVC4;H3BM50	Target of rapamyc	MLST8
23.3108	24.0691	23.2172	23.2795	23.9518	23.4237	NaN	NaN	NaN	23.2748	23.2683	NaN	Q96EY8;S4R3P5;F	Q96EY8;S4R3P5	Cob(I)yrinic acid a	MMAB
NaN	NaN	NaN	NaN	NaN	NaN	NaN	NaN	NaN	NaN	19.8655	NaN	Q9Y4U1;A0A0C4D	Q9Y4U1;A0A0C4D	Methylmalonic ac	MMACHC
NaN	NaN	23.8677	23.9216	23.8929	NaN	NaN	NaN	NaN	23.8969	23.7932	NaN	Q8N4V1;Q8N4V1-	Q8N4V1;Q8N4V1-	Membrane magne	MMGT1
32.555	32.4644	31.0079	32.0583	32.0418	30.4405	31.9381	31.674	31.4772	29.8521	29.3218	30.3765	P03956	P03956	Interstitial collage	MMP1
29.2379	29.7529	30.1958	29.7372	29.6677	30.3292	30.0437	29.7421	30.4428	30.5421	30.0309	30.3807	P50281;F8W1B7;(	P50281	Matrix metallopro	MMP14
28.3687	26.212	26.743	28.264	26.3345	26.9141	26.6796	26.2912	25.4734	27.5739	26.933	24.9411	Q96T76;Q96T76-8	Q96T76;Q96T76-8	MMS19 nucleotid	MMS19
NaN	NaN	NaN	20.8784	NaN	NaN	NaN	NaN	NaN	NaN	NaN	NaN	Q6ZRQ5;E2QRD4	Q6ZRQ5;E2QRD4	Protein MMS22-li	MMS22L
NaN	NaN	NaN	24.0313	NaN	NaN	NaN	NaN	NaN	24.3357	23.5942	NaN	Q9BU76;Q9BU76-	Q9BU76;Q9BU76-	Multiple myeloma	MMTAG2
22.8601	NaN	22.9816	23.0485	23.0322	NaN	NaN	NaN	NaN	22.8652	23.3938	NaN	P51948;P51948-2	P51948;P51948-2	CDK-activating kir	MNAT1
26.5341	26.3752	26.843	25.9033	25.5099	26.1647	26.7388	25.033	25.3656	25.8804	25.9413	25.1216	Q9H8S9;Q7L9L4;(	Q9H8S9;Q7L9L4;(	MOB kinase activa	MOB1A;MOB1B
NaN	23.1512	NaN	NaN	NaN	NaN	NaN	NaN	NaN	NaN	NaN	NaN	A0A087WVS8;Q7(	A0A087WVS8;Q7(	MOB kinase activa	MOB2
25.1735	NaN	25.0644	24.36	NaN	24.6938	25.0292	24.7776	24.8916	NaN	NaN	NaN	Q96BX8;K7EPX7;K	Q96BX8;K7EPX7;K	MOB kinase activa	MOB3A
25.51	24.6022	24.9739	25.1116	24.6814	25.0914	24.0562	23.7179	23.65	24.0472	24.326	23.2929	Q96EN8	Q96EN8	Molybdenum cofa	MOCOS
21.8423	22.1409	22.0699	22.0681	21.9345	NaN	NaN	NaN	NaN	NaN	NaN	NaN	Q9NZB8;Q9NZB8-	Q9NZB8;Q9NZB8-	Molybdenum cofa	MOCS1
NaN	23.4624	NaN	NaN	22.9899	NaN	NaN	NaN	NaN	NaN	NaN	23.2995	O96033;E9PKT9	O96033;E9PKT9	Molybdopterin sy	MOCS2
22.4827	22.7054	22.6328	22.5323	NaN	NaN	22.5343	NaN	NaN	22.1891	22.4102	22.9466	O96007	O96007	Molybdopterin sy	MOCS2
26.0246	25.9064	26.0459	26.0021	26.2855	26.2883	25.7065	26.025	26.9888	25.5102	25.4352	25.6856	Q13724;Q13724-2	Q13724;Q13724-2	Mannosyl-oligosa	MOGS
24.8455	23.4411	24.1511	24.8412	23.6352	24.2421	23.5237	NaN	23.7685	24.349	24.3642	23.8482	Q7Z3U7;Q7Z3U7-	Q7Z3U7;Q7Z3U7-	Protein MON2 ho	MON2
21.6142	NaN	NaN	21.7007	NaN	NaN	NaN	NaN	NaN	21.0386	NaN	NaN	Q9Y6X9;Q9Y6X9-2	Q9Y6X9;Q9Y6X9-2	MORC family CW-	MORC2
21.949	NaN	21.9351	21.9654	NaN	NaN	NaN	NaN	NaN	22.245	22.1878	NaN	Q14149	Q14149	MORC family CW-	MORC3
26.3832	24.9496	25.2275	26.5789	25.1346	25.3546	24.2458	NaN	NaN	24.8682	24.3012	23.5476	Q9HCE1;Q5JR04;(	Q9HCE1;Q5JR04;(	Putative helicase	MOV10
21.3983	NaN	21.0731	21.2687	NaN	NaN	NaN	NaN	NaN	23.464	NaN	NaN	Q6UVY6;Q6UVY6-	Q6UVY6;Q6UVY6-	DBH-like monoox	MOXD1
26.0868	24.8638	24.9742	25.8625	24.8175	25.1019	NaN	25.5033	NaN	25.5172	NaN	NaN	O75352;J3QS48;I3	O75352;J3QS48;I3	Mannose-P-dolich	MPDU1;HBEBP2BI
23.3148	22.7574	23.0482	22.8051	22.9614	22.9224	NaN	23.767	23.2039	22.9317	NaN	NaN	O75970;F5H1U9;(	O75970;F5H1U9;(	Multiple PDZ dom	MPDZ
23.6981	NaN	23.1799	23.6449	23.251	NaN	NaN	22.5865	NaN	24.3805	25.0805	NaN	P29372;A2IDA3;P	P29372;A2IDA3;P	DNA-3-methylade	MPG
NaN	NaN	NaN	22.3313	NaN	NaN	NaN	NaN	NaN	NaN	NaN	NaN	O00566	O00566	U3 small nucleola	MPHOSPH10
23.9889	23.4108	23.7669	23.8332	23.6262	NaN	NaN	NaN	NaN	NaN	NaN	NaN	Q99547;H3BSB3;H	Q99547;H3BSB3;H	M-phase phospho	MPHOSPH6
25.2176	24.8077	25.1281	25.1603	24.7754	25.4207	24.7112	NaN	24.836	24.726	24.9976	24.7767	P34949;H3BPB8;F	P34949;H3BPB8;F	Mannose-6-phosp	MPI
23.3361	24.1319	24.092	23.7895	23.7238	NaN	23.7517	NaN	NaN	NaN	NaN	NaN	Q14168;Q14168-3	Q14168;Q14168-3	MAGUK p55 subfa	MPP2
22.6455	NaN	NaN	22.5007	NaN	NaN	NaN	NaN	NaN	22.3129	NaN	NaN	Q96JB8;F8WBH8;,	Q96JB8;F8WBH8;,	MAGUK p55 subfa	MPP4
24.2186	24.4995	23.9646	23.805	24.1362	NaN	NaN	NaN	24.256	23.6926	23.6177	NaN	Q8N3R9;Q8N3R9-	Q8N3R9;Q8N3R9-	MAGUK p55 subfa	MPP5
28.3402	27.8269	28.303	28.0677	27.9883	28.2598	28.1859	26.7497	27.5485	29.2447	28.7544	28.6082	Q9NZW5;B8ZZG1;	Q9NZW5;B8ZZG1	MAGUK p55 subfa	MPP6
24.7085	25.0382	24.4527	24.8633	24.821	24.7206	24.6234	24.8346	25.1917	25.7606	24.6378	25.2903	Q6WCQ1;Q6WCQ	Q6WCQ1;Q6WCQ	Myosin phosphata	MPRIP
26.759	26.1875	25.5859	25.6006	25.9685	25.5165	26.0604	25.7426	26.4759	26.5676	26.5047	26.4257	P25325-2;P25325	P25325-2;P25325	3-mercaptopyruva	MPST
27.103	26.754	27.2135	26.873	26.2966	27.0709	26.9328	NaN	26.7858	26.8703	26.4771	26.0154	O95297;O95297-2	O95297;O95297-2	Myelin protein ze	MPZL1
23.5495	NaN	NaN	NaN	NaN	NaN	NaN	NaN	NaN	NaN	NaN	NaN	O95297-3;Q9UEL6	O95297-3;Q9UEL6	Myelin protein ze	MPZL1
26.7955	26.5351	26.7086	26.9642	27.0693	26.5066	24.8999	24.7594	24.6086	25.9526	26.0194	25.4992	P49959;F8W7U8;I	P49959;F8W7U8;I	Double-strand bre	MRE11A
NaN	NaN	22.7869	23.0323	NaN	NaN	NaN	23.1631	NaN	NaN	22.9438	NaN	Q9Y605;A0A087W	Q9Y605;A0A087W	MORF4 family-ass	MRFAP1

NaN	22.3981	NaN	NaN	22.2703	NaN	NaN	NaN	NaN	NaN	22.7967	NaN	Q9NV56	Q9NV56	MRG/MORF4L-bir	MRGBP
23.1746	NaN	NaN	23.6183	NaN	NaN	NaN	NaN	NaN	23.3452	23.3724	NaN	Q9BV20;Q9BV20-	Q9BV20;Q9BV20-	Methylthioribose-	MRI1
22.1676	NaN	NaN	22.5387	NaN	NaN	NaN	NaN	NaN	24.1228	NaN	NaN	Q8NDA8;E9PHY8;	Q8NDA8;E9PHY8;	Maestro heat-like	MROH1
24.4125	24.1538	24.3756	25.0183	24.6149	24.0341	NaN	NaN	NaN	23.9655	23.6312	NaN	Q9BYD6;H0Y8N7	Q9BYD6;H0Y8N7	39S ribosomal prc	MRPL1
24.6946	24.2159	23.4206	24.9402	24.0779	24.3734	NaN	NaN	NaN	22.8871	22.7589	22.9636	Q9Y3B7;Q9Y3B7-2	Q9Y3B7;Q9Y3B7-2	39S ribosomal prc	MRPL11
24.7443	24.4494	24.3949	25.3933	24.8469	24.6222	NaN	NaN	23.8836	23.5132	23.7904	23.3577	Q9BYD1;E5RJI7;E5	Q9BYD1;E5RJI7	39S ribosomal prc	MRPL13
22.8888	22.8633	23.1959	24.1033	24.2212	23.2718	NaN	NaN	NaN	NaN	NaN	NaN	Q9P015;E5RHF4;E	Q9P015;E5RHF4;E	39S ribosomal prc	MRPL15
23.5581	23.5423	23.5327	23.9553	24.0847	23.4761	NaN	NaN	NaN	23.0367	22.9772	NaN	Q9NRX2;E9PKV2	Q9NRX2;E9PKV2	39S ribosomal prc	MRPL17
23.1426	NaN	NaN	23.1805	NaN	NaN	NaN	NaN	NaN	NaN	NaN	NaN	Q9H0U6	Q9H0U6	39S ribosomal prc	MRPL18
24.4288	23.3381	23.9349	25.2109	23.3123	24.1215	NaN	NaN	NaN	22.9744	23.0603	NaN	P49406;S4R3W9;I	P49406;S4R3W9	39S ribosomal prc	MRPL19
NaN	NaN	NaN	NaN	NaN	NaN	NaN	NaN	NaN	NaN	NaN	20.1788	Q5T653;C9IY40	Q5T653;C9IY40	39S ribosomal prc	MRPL2
NaN	22.6541	22.582	23.4072	22.7764	NaN	NaN	NaN	23.1744	NaN	NaN	NaN	Q7Z2W9;F5H7V8;	Q7Z2W9;F5H7V8;	39S ribosomal prc	MRPL21
23.4656	23.1967	23.4749	23.9444	23.6726	23.3562	NaN	NaN	NaN	22.837	22.9411	NaN	Q9NWU5;E7ESL0;	Q9NWU5;E7ESL0;	39S ribosomal prc	MRPL22
22.449	NaN	NaN	23.0449	NaN	NaN	NaN	NaN	NaN	NaN	NaN	NaN	Q16540;H7C2P7;A	Q16540;H7C2P7;A	39S ribosomal prc	MRPL23
23.5404	23.4131	23.2223	23.9837	23.6153	23.3026	NaN	NaN	NaN	22.501	22.7982	NaN	Q96A35;X6RJ73;X	Q96A35;X6RJ73	39S ribosomal prc	MRPL24
NaN	NaN	NaN	21.7414	NaN	NaN	NaN	NaN	NaN	NaN	NaN	NaN	Q9P0M9;D6RAN8	Q9P0M9;D6RAN8	39S ribosomal prc	MRPL27
23.8905	23.2494	23.4968	24.2515	23.3277	NaN	NaN	NaN	NaN	NaN	NaN	NaN	P09001;H0Y9G6;E	P09001;H0Y9G6;E	39S ribosomal prc	MRPL3
NaN	NaN	NaN	22.5052	NaN	NaN	NaN	NaN	NaN	NaN	NaN	NaN	Q8TCC3;Q8TCC3-1	Q8TCC3;Q8TCC3-1	39S ribosomal prc	MRPL30
23.5323	22.4022	22.9949	23.1796	23.0269	NaN	NaN	NaN	NaN	NaN	NaN	NaN	Q9BZE1;S4R369;H	Q9BZE1;S4R369;H	39S ribosomal prc	MRPL37
23.9272	25.2287	NaN	24.471	24.3674	NaN	NaN	NaN	NaN	NaN	NaN	NaN	Q96DV4;Q96DV4-	Q96DV4	39S ribosomal prc	MRPL38
25.7962	25.44	25.2499	26.0472	25.4251	25.3626	NaN	NaN	24.8523	25.3215	25.6221	25.6028	Q9NYK5;Q9NYK5-	Q9NYK5;Q9NYK5-	39S ribosomal prc	MRPL39
23.0648	NaN	NaN	23.729	22.5425	22.724	NaN	NaN	NaN	NaN	NaN	NaN	Q9BYD3;K7ELQ0;I	Q9BYD3;K7ELQ0;I	39S ribosomal prc	MRPL4
NaN	NaN	NaN	22.6026	21.8519	NaN	NaN	NaN	NaN	NaN	NaN	NaN	Q9NQ50	Q9NQ50	39S ribosomal prc	MRPL40
21.78	NaN	22.7668	NaN	NaN	NaN	NaN	NaN	NaN	NaN	NaN	NaN	Q8N983-4;Q8N98	Q8N983-4;Q8N98	39S ribosomal prc	MRPL43
22.2321	NaN	22.1438	22.8644	21.7573	NaN	NaN	NaN	NaN	21.6284	21.6256	NaN	Q9H9J2	Q9H9J2	39S ribosomal prc	MRPL44
25.2895	24.8807	25.0078	25.5274	25.4989	25.0074	25.2118	NaN	NaN	24.0382	24.111	NaN	Q9BRJ2;A0A087X	Q9BRJ2;A0A087X	39S ribosomal prc	MRPL45
24.7355	24.7873	24.6603	25.1902	24.6356	24.1692	NaN	23.6281	23.7715	24.3193	23.9989	23.8985	Q9H2W6;A0A087	Q9H2W6	39S ribosomal prc	MRPL46
24.2243	24.2544	23.9294	24.4601	24.3542	24.3288	NaN	NaN	NaN	23.8017	23.9394	NaN	Q96GC5;F5H702;(	Q96GC5;F5H702;(	39S ribosomal prc	MRPL48
NaN	NaN	NaN	22.2753	NaN	NaN	NaN	NaN	NaN	NaN	NaN	NaN	Q13405;E9PI78;H	Q13405;E9PI78;H	39S ribosomal prc	MRPL49
22.0419	NaN	22.0275	22.1757	NaN	NaN	NaN	NaN	NaN	NaN	NaN	NaN	Q8N5N7;Q8N5N7	Q8N5N7;Q8N5N7	39S ribosomal prc	MRPL50
23.4386	NaN	NaN	23.3588	NaN	NaN	NaN	NaN	NaN	NaN	NaN	NaN	P82912;P82912-2	P82912;P82912-2	28S ribosomal prc	MRPS11
23.3178	NaN	21.7293	23.601	NaN	NaN	NaN	NaN	NaN	NaN	NaN	NaN	Q9Y3D3;A6ND22;	Q9Y3D3;A6ND22;	28S ribosomal prc	MRPS16
23.0925	NaN	NaN	23.2488	NaN	NaN	NaN	NaN	NaN	NaN	NaN	NaN	Q9Y2R5;E9PE17;I3	Q9Y2R5;E9PE17;I3	28S ribosomal prc	MRPS17;hCG_198
21.6224	NaN	NaN	NaN	NaN	NaN	NaN	NaN	NaN	NaN	NaN	NaN	Q9Y676;A0A0G2JI	Q9Y676;A0A0G2JI	28S ribosomal prc	MRPS18B
23.9553	23.1335	23.4635	24.5241	23.8214	23.7424	NaN	NaN	23.0892	22.6884	23.0613	NaN	Q9Y399;Q5T8A0	Q9Y399	28S ribosomal prc	MRPS2
NaN	NaN	NaN	NaN	NaN	NaN	NaN	NaN	20.6368	NaN	NaN	NaN	P82921;A0A075B	P82921;A0A075B	28S ribosomal prc	MRPS21
25.6362	25.6622	25.4559	25.9616	25.3029	25.4338	NaN	NaN	24.4792	NaN	NaN	NaN	P82650;G5E9W7;(	P82650;G5E9W7;(	28S ribosomal prc	MRPS22
23.0757	23.7262	23.6967	24.4672	23.9319	23.5258	22.7514	22.4649	23.4904	22.8455	23.4449	22.7846	Q9Y3D9;J3QLR8	Q9Y3D9;J3QLR8	28S ribosomal prc	MRPS23
24.5179	23.949	24.4167	25.5179	24.4753	24.2481	23.6865	NaN	NaN	23.3801	23.9166	NaN	P82663;E7EPW2;F	P82663;E7EPW2;F	28S ribosomal prc	MRPS25
23.7253	NaN	24.2451	24.1479	NaN	NaN	NaN	NaN	NaN	NaN	NaN	NaN	Q9BYN8	Q9BYN8	28S ribosomal prc	MRPS26
24.6629	23.4182	24.0588	25.0888	23.4701	23.1073	NaN	NaN	23.3846	23.3472	23.4064	NaN	Q92552;G5EA06;(	Q92552;G5EA06;(	28S ribosomal prc	MRPS27
23.0908	22.6008	22.4577	22.8449	23.0717	NaN	NaN	23.0827	NaN	NaN	NaN	NaN	Q9Y2Q9;H0YAT2;I	Q9Y2Q9;H0YAT2;I	28S ribosomal prc	MRPS28
NaN	NaN	NaN	23.6696	NaN	NaN	NaN	NaN	NaN	NaN	NaN	NaN	Q9NP92;A0A087V	Q9NP92;A0A087V	28S ribosomal prc	MRPS30
21.985	NaN	21.9682	22.9808	NaN	NaN	NaN	NaN	NaN	NaN	NaN	NaN	Q92665	Q92665	28S ribosomal prc	MRPS31
24.6138	23.4854	24.1391	25.1949	24.0885	24.2733	NaN	NaN	NaN	23.9638	23.6822	NaN	P82930;A0A087W	P82930;A0A087W	28S ribosomal prc	MRPS34
NaN	NaN	NaN	NaN	NaN	NaN	NaN	NaN	NaN	NaN	20.8777	NaN	P82673;H0YG82;P	P82673;H0YG82;P	28S ribosomal prc	MRPS35
23.001	24.4245	23.7542	NaN	24.5349	23.5201	NaN	25.0457	25.6407	NaN	22.8169	23.9888	P82909	P82909	28S ribosomal prc	MRPS36
23.9362	NaN	NaN	22.4317	NaN	NaN	NaN	NaN	NaN	NaN	NaN	NaN	P82675;P82675-2	P82675	28S ribosomal prc	MRPS5
NaN	23.3676	23.5986	23.7385	23.636	23.327	NaN	NaN	NaN	NaN	NaN	NaN	P82932	P82932	28S ribosomal prc	MRPS6
23.5293	23.3807	23.3199	23.8664	23.5341	23.5139	NaN	NaN	23.3061	NaN	NaN	NaN	Q9Y2R9;J3QLS3;J3	Q9Y2R9;J3QLS3;J3	28S ribosomal prc	MRPS7
21.7162	NaN	NaN	NaN	NaN	NaN	NaN	NaN	NaN	NaN	NaN	NaN	P82933	P82933	28S ribosomal prc	MRPS9
29.4873	26.4417	27.8909	29.6178	27.2385	28.0495	25.0844	23.821	23.3545	27.6476	25.8949	25.5219	Q9UKD2	Q9UKD2	mRNA turnover p	MRTO4
28.0763	26.4371	27.4084	28.2567	27.4153	27.5774	25.0944	25.1672	24.7973	27.5374	27.4003	26.498	P43246;P43246-2	P43246;P43246-2	DNA mismatch re	MSH2
23.6004	NaN	22.9076	23.3276	23.1534	23.375	NaN	NaN	NaN	23.1043	23.3284	NaN	P20585	P20585	DNA mismatch re	MSH3
27.6986	26.2262	27.1765	27.9731	26.949	27.2717	NaN	25.9091	24.1145	27.0909	27.4699	26.5848	P52701;P52701-3	P52701;P52701-3	DNA mismatch re	MSH6
NaN	NaN	NaN	NaN	NaN	NaN	NaN	NaN	NaN	21.3904	NaN	NaN	Q15800;D6RDP9;I	Q15800;D6RDP9;I	Methylsterol mon	MSMO1
34.6346	35.3193	35.9716	35.2503	35.7159	36.2639	35.0644	34.015	33.8226	36.4186	35.9362	35.0692	P26038;V9GZ54;E	P26038	Moesin	MSN
23.9672	23.5845	23.6016	23.4004	23.1509	NaN	NaN	NaN	NaN	24.3488	24.0947	23.8148	Q9UJ68;Q9UJ68-2	Q9UJ68;Q9UJ68-2	Mitochondrial pe	MSRA
22.1825	22.0584	22.3117	22.4281	22.5375	22.8811	NaN	NaN	NaN	22.8294	22.3308	NaN	Q04912;Q04912-2	Q04912;Q04912-2	Macrophage-stim	MST1R
24.5334	23.285	23.8167	24.4342	23.5558	23.4951	NaN	NaN	NaN	23.4099	23.6762	23.0936	Q9BUK6;Q9BUK6-	Q9BUK6;Q9BUK6-	Protein misato ho	MSTO1

27.5594	26.6498	26.8968	27.649	26.8926	27.1145	25.9197	26.4724	25.7359	27.1634	27.413	26.9148	O94776;O94776-2	O94776;O94776-2	Metastasis-associ	MTA2
23.6032	24.0261	24.3815	24.2651	24.6401	24.074	NaN	NaN	NaN	23.8563	23.9457	NaN	Q9BTC8;Q9BTC8-:	Q9BTC8;Q9BTC8-:	Metastasis-associ	MTA3
NaN	NaN	24.2351	NaN	22.5926	23.8609	NaN	NaN	NaN	NaN	NaN	NaN	Q13126;J3QSB7;B	Q13126;J3QSB7;B	S-methyl-5-thioac	MTAP
NaN	NaN	NaN	23.2144	NaN	NaN	NaN	NaN	NaN	NaN	NaN	NaN	P00846	P00846	ATP synthase sub	MT-ATP6
23.9429	24.2153	23.9822	24.1217	24.2648	24.2227	23.6824	24.8146	25.8835	23.4817	23.8338	23.6492	Q9Y6C9;E9PIE4	Q9Y6C9;E9PIE4	Mitochondrial car	MTCH2
26.0083	24.3144	25.06	25.1737	24.096	23.7691	24.5298	NaN	NaN	24.1957	23.0775	NaN	Q9Y4B5;Q9Y4B5-2	Q9Y4B5;Q9Y4B5-2	Microtubule cross	MTCL1
24.9442	24.887	25.5025	25.2774	24.9937	25.4129	24.088	25.2866	26.8901	23.5634	23.5849	25.5198	P00403	P00403	Cytochrome c oxi	MT-CO2
25.1045	24.2874	24.5056	24.7137	24.3917	24.3459	23.9348	NaN	24.6701	23.785	23.7043	24.1791	Q86UE4;E5RJU9;H	Q86UE4;E5RJU9	Protein LYRIC	MTDH
32.6601	32.1382	32.0761	32.3738	32.3885	32.1157	31.9197	32.3287	32.0609	32.8169	32.86	32.2589	P11586;F5H2F4;V	P11586;F5H2F4	C-1-tetrahydrofol	MTHFD1
27.0976	26.0625	26.1927	27.5891	25.7397	25.6968	22.8733	24.4264	23.5128	25.5837	25.5266	NaN	A0A087WVM4;B7	A0A087WVM4;B7	Monofunctional C	MTHFD1L
NaN	NaN	NaN	23.1248	NaN	NaN	NaN	NaN	NaN	NaN	NaN	NaN	P13995;B9A062;B	P13995;B9A062;B	Bifunctional meth	MTHFD2
23.3724	23.0757	22.9593	24.3558	23.4457	23.4278	NaN	NaN	NaN	NaN	NaN	NaN	P46199;H7C213	P46199;H7C213	Translation initiat	MTIF2
21.8108	NaN	22.8552	21.7425	21.7663	NaN	NaN	NaN	NaN	22.7285	23.0384	NaN	Q13496;Q13496-2	Q13496;Q13496-2	Myotubularin	MTM1
24.2014	23.9942	24.6766	23.8518	24.1555	24.4601	23.6536	NaN	NaN	24.4421	24.0803	24.0333	Q13613;F8WA39;	Q13613;F8WA39;	Myotubularin-rela	MTMR1
NaN	NaN	22.5059	22.4627	23.0588	22.7187	NaN	NaN	NaN	22.4784	23.386	NaN	Q9C0I1;Q9C0I1-3;	Q9C0I1;Q9C0I1-3;	Myotubularin-rela	MTMR12
21.4503	NaN	NaN	NaN	NaN	NaN	NaN	NaN	NaN	NaN	NaN	NaN	Q8NCE2;Q8NCE2-	Q8NCE2;Q8NCE2-	Myotubularin-rela	MTMR14
NaN	NaN	22.9513	NaN	NaN	NaN	NaN	NaN	NaN	NaN	NaN	NaN	Q13614;C9JEX3;Q	Q13614;C9JEX3;Q	Myotubularin-rela	MTMR2
22.7545	NaN	NaN	NaN	NaN	NaN	NaN	NaN	NaN	NaN	NaN	NaN	Q9NYA4;J3QR65;J	Q9NYA4;J3QR65	Myotubularin-rela	MTMR4
23.1879	NaN	22.9562	23.1541	NaN	NaN	NaN	NaN	NaN	23.2912	NaN	NaN	Q9Y217;Q9Y217-2	Q9Y217;Q9Y217-2	Myotubularin-rela	MTMR6
27.8955	26.8311	27.7452	27.9675	27.1373	27.7612	27.073	26.5995	26.4867	27.6308	27.3906	27.4847	P42345;B1AKP8;B	P42345	Serine/threonine-	MTOR
23.0882	NaN	NaN	23.3987	NaN	NaN	NaN	NaN	NaN	NaN	NaN	NaN	Q9NVV4-2;Q9NV	Q9NVV4-2;Q9NV	Poly(A) RNA polyr	MTPAP
26.475	27.2108	26.8514	26.1213	26.5976	26.576	26.2227	26.5786	27.1728	26.9825	26.8099	27.3348	P58546;C9JL85	P58546;C9JL85	Myotrophin	MTPN
23.8667	23.153	23.5152	23.5316	22.7509	22.9861	22.0213	23.1708	NaN	22.7137	22.6089	NaN	Q99707;Q99707-2	Q99707;Q99707-2	Methionine synth	MTR
23.4536	NaN	23.2541	23.4715	NaN	NaN	NaN	NaN	NaN	23.4339	NaN	NaN	Q9UBK8;Q9UBK8-	Q9UBK8;Q9UBK8-	Methionine synth	MTRR
NaN	23.5286	23.1556	NaN	23.3727	NaN	NaN	NaN	NaN	NaN	NaN	NaN	O75431;O75431-2	O75431;O75431-2	Metaxin-2	MTX2
23.5081	23.5803	23.944	24.0891	23.6687	23.266	NaN	NaN	NaN	NaN	NaN	NaN	P22033	P22033	Methylmalonyl-Co	MUT
25.0491	26.0748	25.5955	25.0656	25.4991	25.6082	25.8566	26.0714	26.6976	24.6545	24.7508	25.8934	Q96EY5;Q96EY5-2	Q96EY5;Q96EY5-2	Multivesicular bo	MVB12A
24.9866	24.8162	24.6726	24.4717	24.4561	24.5228	25.6787	25.0418	25.5919	25.8982	25.5337	25.5224	P53602;H3BP35;A	P53602;H3BP35	Diphosphomevalo	MVD
25.4085	23.9883	24.7179	25.1215	24.0276	24.5462	24.7154	NaN	24.137	25.628	25.7313	23.9775	Q03426;F5H8H2;F	Q03426;F5H8H2;F	Mevalonate kinas	MVK
29.1308	30.075	29.4687	28.7667	29.9979	29.3976	29.556	30.0492	30.7363	29.7513	29.1064	30.1489	Q14764;H3BQK6;I	Q14764	Major vault prote	MVP
NaN	NaN	NaN	NaN	NaN	NaN	NaN	NaN	NaN	21.2692	NaN	NaN	P20591;F8W8T1;F	P20591;F8W8T1;F	Interferon-induce	MX1
24.1599	23.3596	24.0396	23.6174	22.7191	23.812	23.8123	NaN	NaN	23.6772	NaN	NaN	P84157-2;K7EQX8	P84157-2;K7EQX8	Matrix-remodelin	MXRA7
25.6245	24.6497	25.1415	25.168	24.3146	24.6429	24.5473	23.665	NaN	24.639	24.2224	24.426	Q96S97;C9JJV6;C9	Q96S97;C9JJV6;C9	Myeloid-associate	MYADM
28.2218	26.4872	27.1741	28.8246	27.2781	27.1972	25.2475	25.4035	24.3884	27.4955	27.6494	26.1009	Q9BQG0;Q9BQG0	Q9BQG0;Q9BQG0	Myb-binding prot	MYBBP1A
25.4049	25.9621	25.1483	25.2206	25.1002	25.2323	26.0035	NaN	25.1658	25.0199	25.168	NaN	Q99417;A0A087W	Q99417;A0A087W	C-Myc-binding pr	MYCBP
27.4562	28.2454	26.9085	26.2918	26.5348	25.7896	26.5607	NaN	26.3427	25.6755	26.9431	NaN	O75592;O75592-2	O75592;O75592-2	E3 ubiquitin-prote	MYCBP2
24.5551	24.2426	24.1987	24.082	24.281	24.5345	24.6072	NaN	23.8183	25.143	25.2766	NaN	Q99836;A0A0A0N	Q99836;A0A0A0N	Myeloid differenti	MYD88
27.0156	28.398	27.4507	27.1022	28.0437	27.6601	27.4587	28.6301	28.6401	27.7588	27.7409	27.3032	Q969H8;M0QXF7,	Q969H8;M0QXF7,	Myeloid-derived g	MYDGF
24.1648	24.2971	24.2888	24.3322	24.4611	24.3941	NaN	NaN	NaN	23.1334	24.4753	24.9253	A0A0A0MR39;Q9	A0A0A0MR39;Q9	Myelin expression	MYEF2
30.2291	29.9471	30.4555	30.9006	30.2583	30.7222	30.3444	30.7351	30.4096	30.5062	30.6357	30.532	P35580;P35580-3	P35580;P35580-3	Myosin-10	MYH10
25.936	26.9835	27.5241	27.135	27.3322	27.8037	25.5187	25.696	26.5364	26.6181	26.919	26.723	Q7Z406;Q7Z406-6	Q7Z406;Q7Z406-6	Myosin-14	MYH14
34.7368	34.4714	35.0216	35.0841	34.6543	35.2916	34.3499	34.592	34.1747	34.2682	34.3607	34.244	P35579;P35579-2	P35579;P35579-2	Myosin-9	MYH9
29.8174	29.6078	29.393	29.5654	29.2001	29.3886	29.2201	28.5993	28.0073	29.0234	28.8165	28.4654	O14950;P19105;J	O14950;P19105;J	Myosin regulatory	MYL12B;MYL12A
28.736	29.3339	28.9046	28.6817	28.9273	29.3526	29.3376	28.7748	27.6271	29.2183	28.8903	28.5166	P08590;P05976;P	P08590;P05976;P	Myosin light chair	MYL3;MYL1
31.7778	32.7577	32.4568	31.9554	32.9336	32.3771	31.8837	32.5998	31.8944	32.3851	32.3668	32.3759	P60660;F8W1R7;J	P60660;F8W1R7;J	Myosin light polyp	MYL6
NaN	NaN	NaN	21.4654	NaN	NaN	NaN	NaN	NaN	NaN	NaN	NaN	P60660-2;G8JLA2;	P60660-2;G8JLA2	Myosin light polyp	MYL6
NaN	NaN	22.4658	NaN	23.6438	NaN	NaN	NaN	NaN	22.5318	NaN	NaN	P14649;F8W1I5	P14649;F8W1I5	Myosin light chair	MYL6B
23.9088	24.3867	24.3884	24.0263	NaN	NaN	NaN	NaN	NaN	23.0267	NaN	NaN	P24844;P24844-2	P24844;P24844-2	Myosin regulatory	MYL9
24.0599	NaN	24.1958	24.1365	24.4438	24.4767	NaN	NaN	NaN	NaN	NaN	NaN	Q15746;Q15746-9	Q15746;Q15746-9	Myosin light chair	MYLK
24.5249	23.7285	25.8818	24.6317	24.2576	25.2554	27.1142	26.2583	26.3316	25.5685	24.8495	24.4999	Q9HD67;A0A0A0I	Q9HD67;A0A0A0I	Unconventional m	MYO10
25.225	24.3339	26.1746	25.5827	25.3459	26.3608	24.0307	24.3063	NaN	26.0641	25.9494	24.6919	Q92614;Q92614-4	Q92614;Q92614-4	Unconventional m	MYO18A
NaN	NaN	NaN	NaN	NaN	NaN	NaN	NaN	NaN	NaN	NaN	22.7816	Q8IUG5;A0A075B	Q8IUG5;A0A075B	Unconventional m	MYO18B
30.1328	30.1285	30.9883	30.6285	30.4061	31.3456	29.9523	29.459	29.9114	31.2695	30.9307	30.8593	O43795;E9PDF6;E	O43795;E9PDF6	Unconventional m	MYO1B
25.3817	25.3421	25.8888	25.461	25.4386	NaN	NaN	NaN	25.8526	26.6975	26.0976	NaN	O00159-2;I3L204;	O00159-2	Unconventional m	MYO1C
31.8946	31.8363	32.7763	32.1047	32.1098	33.0447	32.0634	31.7794	31.9792	33.0575	32.7388	32.7208	O00159-3;O00159	O00159-3;O00159	Unconventional m	MYO1C
28.0399	27.6077	28.4531	27.9409	27.1967	28.0654	26.1752	24.5133	26.3222	27.5069	26.9287	26.4753	O94832;J3QRN6;K	O94832;J3QRN6;K	Unconventional m	MYO1D
27.4026	26.4155	27.1527	27.2183	26.3987	26.8551	25.7457	25.6771	25.3644	26.4221	26.466	26.3452	Q12965;H0YNQ8;	Q12965	Unconventional m	MYO1E
25.9779	25.5347	26.1432	26.1473	25.4654	25.9958	25.5356	26.0832	25.8526	25.5976	25.7885	25.9154	Q9Y4I1;G3V394;F	Q9Y4I1;G3V394;F	Unconventional m	MYO5A
24.0883	NaN	24.2348	24.0488	24.1572	24.1451	NaN	NaN	NaN	NaN	NaN	NaN	Q9NQX4;Q9NQX4	Q9NQX4	Unconventional m	MYO5C
28.8983	28.4728	28.9189	29.1832	28.5772	29.0687	29.0149	28.605	28.9013	29.1345	29.0486	28.7919	A0A0A0MRM8;Q9	A0A0A0MRM8;Q9	Unconventional m	MYO6

25.6426	25.5146	26.1821	26.269	25.1058	25.53	25.3536	23.1793	24.6468	25.7536	25.1444	25.6568	Q13459;M0R0P8;	Q13459;M0R0P8;	Unconventional m	MYO9B
33.5011	32.819	33.2586	33.7369	32.804	33.4389	32.5976	32.1158	32.1211	33.4725	32.7669	32.4335	Q9NZM1;Q9NZM	Q9NZM1;Q9NZM	Myoferlin	MYOF
NaN	NaN	NaN	21.0978	NaN	NaN	NaN	NaN	NaN	NaN	NaN	NaN	Q6NZ67;Q6P582;I	Q6NZ67;Q6P582;I	Mitotic-spindle or	MZT2B;MZT2A
26.9004	27.4647	26.7621	26.8701	27.4983	26.9971	26.7528	27.7498	27.439	27.247	27.7009	27.6999	P41227;P41227-2	P41227;P41227-2,	N-alpha-acetyltra	NAA10;NAA11
29.1164	28.2026	28.5048	28.7733	28.5397	28.5099	28.4494	28.8867	28.8139	29.1088	29.2639	28.7896	Q9BXJ9;A0A0B4J1	Q9BXJ9;A0A0B4J1	N-alpha-acetyltra	NAA15
24.8121	23.6834	24.1356	24.6246	23.6612	24.2753	NaN	NaN	NaN	23.3836	23.7458	NaN	Q6N069;Q6N069-	Q6N069	N-alpha-acetyltra	NAA16
23.1828	23.9864	23.5699	24.3344	24.0251	23.7066	23.1934	NaN	NaN	NaN	23.6387	24.0846	P61599;A8MZB2;I	P61599;A8MZB2	N-alpha-acetyltra	NAA20
23.8295	24.0846	23.9965	23.8154	24.1612	23.4458	24.6155	24.2104	24.7131	23.957	24.1219	23.733	Q14CX7;Q14CX7-:	Q14CX7;Q14CX7-:	N-alpha-acetyltra	NAA25
24.6467	23.9571	24.095	24.5482	23.8778	24.0924	NaN	NaN	NaN	24.2867	24.2165	NaN	Q147X3;Q147X3-2	Q147X3;Q147X3-2	N-alpha-acetyltra	NAA30
26.5419	25.1448	25.6431	26.2282	25.5923	25.1644	25.0604	23.8531	25.2322	26.7327	26.6638	25.5057	Q5VZE5;Q5VZE5-2	Q5VZE5	N-alpha-acetyltra	NAA35
NaN	NaN	NaN	22.1811	NaN	NaN	NaN	NaN	NaN	NaN	NaN	NaN	Q86UY6;Q86UY6-	Q86UY6;Q86UY6-	N-alpha-acetyltra	NAA40
25.8944	25.8936	25.9468	25.7446	26.0339	25.6269	25.5662	25.7596	26.9093	25.9121	26.2538	26.3895	Q9GZZ1;Q9GZZ1-:	Q9GZZ1;Q9GZZ1-:	N-alpha-acetyltra	NAA50
27.5075	26.7427	27.0506	27.4574	26.3922	26.9719	27.2034	26.9519	25.8631	27.5092	27.0315	26.4195	E9PAV3;F8VZJ2;H	E9PAV3;F8VZJ2;H	Nascent polypept	NACA
22.464	NaN	NaN	NaN	NaN	NaN	NaN	NaN	NaN	NaN	NaN	NaN	Q96RE7;K7ENW4	Q96RE7;K7ENW4	Nucleus accumbe	NACC1
24.9214	23.8848	23.3182	24.3288	24.3444	23.4291	NaN	NaN	NaN	24.0027	24.2468	NaN	Q4G0N4;Q4G0N4	Q4G0N4;Q4G0N4	NAD kinase 2, mit	NADK2
21.1935	NaN	NaN	21.249	NaN	NaN	NaN	NaN	NaN	NaN	NaN	NaN	Q6IA69;H0YCQ6;E	Q6IA69;H0YCQ6;E	Glutamine-depen	NADSYN1
25.4396	25.8726	25.6664	25.6768	26.0994	26.0217	25.9873	25.4928	25.3833	26.3715	26.6216	26.3884	Q13564;Q13564-4	Q13564;Q13564-4	NEDD8-activating	NAE1
NaN	NaN	NaN	21.0354	NaN	NaN	NaN	NaN	NaN	NaN	NaN	NaN	Q96HR8;Q96HR8-	Q96HR8;Q96HR8-	H/ACA ribonucleo	NAF1
NaN	NaN	NaN	NaN	NaN	NaN	NaN	NaN	NaN	20.649	NaN	NaN	P17050	P17050	Alpha-N-acetylgal	NAGA
26.6122	26.3705	26.1176	25.9325	26.103	25.9534	26.2742	26.1384	25.9901	26.2932	26.1904	26.4361	Q9UJ70;Q9UJ70-2	Q9UJ70;Q9UJ70-2	N-acetyl-D-glucos	NAGK
30.6504	30.3398	29.8355	29.7128	29.8937	29.6668	30.2181	29.5434	29.7301	30.5086	29.9362	29.9286	P43490;A0A0C4D	P43490;A0A0C4D	Nicotinamide pho	NAMPT
29.4004	30.1174	29.3508	28.906	29.9106	29.3415	29.4784	29.2279	29.4905	30.1886	30.1982	29.5633	Q9NR45;Q5TBR0;	Q9NR45	Sialic acid synthas	NANS
30.9507	30.2923	30.7866	30.9227	30.1221	30.786	30.64	30.8252	29.8981	30.6281	30.4889	30.481	P55209;F5H4R6;P	P55209;F5H4R6;P	Nucleosome asser	NAP1L1
24.9377	23.1476	24.5793	25.1808	23.9154	24.9458	24.2951	NaN	NaN	23.8698	24.2615	23.748	F8VV59;B7Z9C2;P	F8VV59;B7Z9C2;P	Nucleosome asser	NAP1L1
27.3561	26.8925	27.2571	26.921	26.6432	26.6591	27.0372	27.436	26.2662	26.3104	26.4617	27.1683	Q99733;Q99733-2	Q99733;Q99733-2	Nucleosome asser	NAP1L4
27.1766	27.3493	27.2351	26.9607	26.7743	27.0891	27.682	26.734	27.2667	27.5026	26.9106	27.2743	P54920;M0R0Y2;I	P54920;M0R0Y2;I	Alpha-soluble NSF	NAPA
NaN	NaN	21.0079	NaN	NaN	NaN	NaN	NaN	NaN	22.2492	NaN	NaN	Q9H115;A0A087V	Q9H115;A0A087V	Beta-soluble NSF	NAPB
27.1003	27.7273	27.2975	26.7832	27.6095	27.2678	27.4481	27.4535	27.6117	27.2546	27.226	27.7143	Q99747;Q99747-2	Q99747;Q99747-2	Gamma-soluble N	NAPG
22.0702	NaN	22.7479	22.1627	22.1991	22.441	NaN	NaN	NaN	NaN	NaN	NaN	Q6XQN6;Q6XQN6	Q6XQN6;Q6XQN6	Nicotinate phosph	NAPRT
26.4559	26.295	26.2962	26.2452	26.5172	26.3068	26.7933	26.8081	26.5523	26.5531	26.7281	26.9166	O43776;K7EIU7;O	O43776	Asparagine--tRNA	NARS
23.6499	NaN	22.5433	23.5379	NaN	NaN	NaN	NaN	23.1252	23.216	23.2181	NaN	Q96I59;E9PRK2;H	Q96I59;E9PRK2	Probable asparagi	NARS2
26.1952	26.2103	26.5692	26.2989	26.3869	26.9347	25.9199	26.2353	25.2913	26.5181	26.7469	27.057	P49321;P49321-3	P49321;P49321-3,	Nuclear autoantig	NASP
NaN	NaN	NaN	NaN	NaN	NaN	NaN	NaN	22.1561	21.624	NaN	NaN	P18440;F5H5R8	P18440;F5H5R8	Arylamine N-acety	NAT1
28.134	27.1025	27.5204	29.3598	27.7312	27.2767	24.0086	24.2071	24.3022	26.2723	26.8243	25.9298	Q9H0A0;Q9H0A0-	Q9H0A0;Q9H0A0-	N-acetyltransfera	NAT10
NaN	NaN	NaN	NaN	NaN	NaN	21.4401	NaN	NaN	NaN	NaN	NaN	Q93015;Q93015-2	Q93015;Q93015-2	N-acetyltransfera	NAT6
22.559	NaN	22.3981	22.7588	22.6307	NaN	22.6684	NaN	NaN	22.4147	NaN	NaN	Q8IVL0;H0YHA8;C	Q8IVL0;H0YHA8;C	Neuron navigator	NAV3
NaN	NaN	NaN	21.6514	NaN	NaN	NaN	NaN	NaN	NaN	NaN	NaN	A2RRP1;A2RRP1-2	A2RRP1;A2RRP1-2	Neuroblastoma-a	NBAS
23.544	NaN	22.2081	23.2577	NaN	NaN	22.5177	NaN	NaN	22.886	22.7231	NaN	Q6ZNJ1;Q6ZNJ1-2	Q6ZNJ1;Q6ZNJ1-2	Neurobeachin-like	NBEAL2
28.0844	26.1018	28.2173	26.1236	24.9514	26.1803	27.6234	26.6296	25.7311	25.3381	24.396	23.6291	Q14596;Q14596-2	Q14596;Q14596-2	Next to BRCA1 ge	NBR1
28.6124	27.0413	27.7623	28.3884	27.0723	27.7518	27.0449	27.3363	26.6085	27.6296	27.2408	26.7693	Q15021;E7EN77;F	Q15021;E7EN77	Condensin comple	NCAPD2
26.1449	NaN	26.1568	26.3249	NaN	NaN	NaN	NaN	NaN	NaN	26.0046	NaN	P42695;G3V1A9;E	P42695;G3V1A9	Condensin-2 comp	NCAPD3
26.9436	25.3645	26.7015	27.3148	25.7754	26.4365	25.6023	24.7759	25.6117	26.0828	26.0629	24.4286	Q9BPX3;D6RA93;I	Q9BPX3	Condensin comple	NCAPG
24.9902	NaN	24.0864	24.9057	23.7708	24.275	NaN	NaN	24.6455	23.862	23.625	NaN	Q86XI2;Q86XI2-2;	Q86XI2;Q86XI2-2;	Condensin-2 comp	NCAPG2
26.562	25.8803	25.9389	26.328	25.6797	25.9862	25.7262	26.5448	26.2014	25.711	25.3107	24.8608	Q15003;E9PHA2;(	Q15003;E9PHA2;(	Condensin comple	NCAPH
23.013	NaN	22.1981	23.014	NaN	22.3805	NaN	NaN	NaN	NaN	NaN	NaN	Q6IBW4;A0A0A6\	Q6IBW4;A0A0A6\	Condensin-2 comp	NCAPH2
26.2223	24.1182	25.0652	25.4842	24.562	24.9555	23.8983	NaN	NaN	25.301	25.3696	24.961	Q09161;X6R941;F	Q09161	Nuclear cap-bindi	NCBP1
24.171	24.2561	23.6805	23.7986	24.3444	NaN	NaN	NaN	NaN	23.8375	NaN	NaN	P52298;P52298-3	P52298;P52298-3,	Nuclear cap-bindi	NCBP2
23.7942	23.3273	23.4987	23.802	23.6786	NaN	23.7665	NaN	NaN	NaN	NaN	NaN	Q9UBB6;Q9UBB6-	Q9UBB6;Q9UBB6-	Neurochondrin	NCDN
28.4037	27.9106	28.1274	28.6487	28.0118	28.3821	28.0459	28.006	28.8846	28.1421	27.7533	28.0858	Q6PIU2;A0A0R4J2	Q6PIU2;A0A0R4J2	Neutral cholester	NCEH1
23.4164	23.2296	23.2447	22.9449	23.1897	23.1818	23.0597	NaN	23.0358	22.9157	22.9307	NaN	P16333;P16333-2	P16333;P16333-2,	Cytoplasmic prote	NCK1
28.5279	28.1985	28.8267	28.4144	28.2957	28.8328	28.6372	28.4512	28.5927	29.1566	29.0408	28.7403	Q9Y2A7;Q9Y2A7-:	Q9Y2A7;Q9Y2A7-:	Nck-associated pr	NCKAP1
23.9434	23.7236	23.8398	24.1999	23.618	23.7486	24.4041	24.5195	24.0882	24.0807	25.574	24.3417	Q9NZQ3;Q9NZQ3	Q9NZQ3;Q9NZQ3	NCK-interacting p	NCKIPSD
31.9987	30.4304	30.7306	31.7186	30.6106	30.929	29.6745	30.3349	29.4119	30.466	30.5131	30.7246	P19338;H7BY16;C	P19338;H7BY16	Nucleolin	NCL
24.5722	24.6432	24.7703	25.1191	25.0256	24.7304	24.781	25.567	26.0476	24.8212	24.7606	24.9202	Q969V3;Q969V3-:	Q969V3;Q969V3-:	Nicalin	NCLN
NaN	NaN	NaN	NaN	NaN	NaN	NaN	NaN	NaN	NaN	NaN	26.8288	Q15596;E7EWM1	Q15596;E7EWM1	Nuclear receptor	NCOA2
26.5505	25.3268	25.9315	25.0294	24.2583	25.3364	26.2888	23.9498	24.9727	24.9992	24.653	24.0349	Q13772;Q13772-3	Q13772;Q13772-3	Nuclear receptor	NCOA4
23.9193	23.6674	23.7879	23.4328	23.6819	24.0782	24.3874	NaN	22.9729	24.2535	24.6869	24.7232	Q9HCD5	Q9HCD5	Nuclear receptor	NCOA5
27.3208	27.0175	27.5869	27.4782	26.9903	27.6678	27.1984	26.5173	26.7652	27.8949	27.1133	26.9548	Q92542;Q92542-2	Q92542;Q92542-2	Nicastrin	NCSTN
NaN	NaN	NaN	23.0901	NaN	NaN	NaN	NaN	NaN	23.3333	23.417	NaN	Q9BTX1;Q9BTX1-(	Q9BTX1;Q9BTX1-(	Nucleoporin NDC:	NDC1
26.3525	24.6947	25.2243	26.1043	24.5521	25.4734	22.6666	NaN	NaN	25.5949	25.181	25.3411	O14777;I3L4G3;V	O14777	Kinetochore prote	NDC80

22.7443	NaN	NaN	21.8449	NaN	NaN	NaN	NaN	NaN	NaN	NaN	NaN	Q9NXR1;Q9NXR1-	Q9NXR1;Q9NXR1-	Nuclear distributio	NDE1
NaN	NaN	24.3178	NaN	NaN	NaN	NaN	NaN	NaN	NaN	NaN	NaN	Q9GZM8;A6NIZ0;	Q9GZM8;A6NIZ0;	Nuclear distributio	NDEL1
25.5201	25.6363	25.4652	24.9185	24.8519	24.779	24.8074	NaN	NaN	24.0603	23.5068	24.8871	Q9NV92;H7C5F7;I	Q9NV92;H7C5F7;I	NEDD4 family-inte	NDFIP2
28.9313	30.204	30.0657	28.9468	29.9104	30.2832	30.4349	30.5402	30.7356	30.5189	30.1339	30.6826	Q92597;Q92597-3	Q92597;Q92597-3	Protein NDRG1	NDRG1
27.3587	27.6372	27.7562	26.9321	27.3551	27.5426	27.457	27.6136	27.7638	26.6375	26.4015	27.0339	Q9UGV2;Q5TH30;	Q9UGV2;Q5TH30;	Protein NDRG3	NDRG3
NaN	22.3	NaN	23.3645	NaN	NaN	NaN	NaN	NaN	NaN	NaN	NaN	O95299;A0A087W	O95299;A0A087W	NADH dehydroger	NDUFA10
22.7563	NaN	NaN	22.9029	NaN	NaN	NaN	NaN	NaN	NaN	22.1905	NaN	O00483	O00483	Cytochrome c oxi	NDUFA4
22.5605	22.9501	22.6421	23.1022	23.7114	23.493	NaN	NaN	NaN	NaN	NaN	NaN	Q16718;A0A087X	Q16718;A0A087X	NADH dehydroger	NDUFA5
23.3924	23.7889	23.6589	23.9326	23.82	23.8233	NaN	NaN	NaN	NaN	NaN	NaN	P56556;A0A0C4D	P56556;A0A0C4D	NADH dehydroger	NDUFA6
25.5343	26.9008	25.935	25.8313	26.75	25.6124	24.7393	24.3372	25.3003	25.4252	25.1322	24.939	Q8N183;D6RA56;	Q8N183	Mimitin, mitochor	NDUFAF2
NaN	NaN	NaN	NaN	22.9076	NaN	NaN	NaN	NaN	NaN	NaN	NaN	O75438;O75438-2	O75438;O75438-2	NADH dehydroger	NDUFB1
22.6223	24.357	23.7141	23.6942	24.1047	24.5113	NaN	24.6748	25.3787	22.3841	23.2143	23.8654	O96000;H3BPJ9;H	O96000;H3BPJ9;H	NADH dehydroger	NDUFB10
NaN	NaN	NaN	NaN	NaN	NaN	NaN	NaN	NaN	NaN	21.85	NaN	O95168;C9JXQ9;C	O95168;C9JXQ9;C	NADH dehydroger	NDUFB4
NaN	NaN	NaN	NaN	NaN	22.1738	NaN	NaN	NaN	NaN	NaN	NaN	Q9Y6M9;E9PH64;	Q9Y6M9;E9PH64;	NADH dehydroger	NDUFB9
24.4459	24.5536	24.3001	24.7316	24.9806	24.1624	NaN	NaN	23.9936	NaN	NaN	NaN	P28331;P28331-4	P28331;P28331-4	NADH-ubiquinone	NDUFS1
24.4992	25.0103	24.6118	25.0889	25.4133	24.4479	NaN	24.6383	NaN	NaN	24.2379	24.0894	O75489;E9PS48;O	O75489	NADH dehydroger	NDUFS3
23.5176	NaN	NaN	23.8425	22.9873	23.1776	NaN	NaN	NaN	NaN	NaN	NaN	P49821;G3V0I5;P	P49821;G3V0I5;P	NADH dehydroger	NDUFV1
23.7281	23.5974	23.9072	23.744	24.1341	23.6912	24.1883	23.5789	23.5359	23.5577	24.0568	23.7944	Q9NVZ3;Q9NVZ3-	Q9NVZ3;Q9NVZ3-	Adaptin ear-bindi	NECAP2
NaN	NaN	21.7106	NaN	NaN	NaN	NaN	NaN	NaN	NaN	NaN	NaN	Q8NHV4;Q8NHV4	Q8NHV4;Q8NHV4	Protein NEDD1	NEDD1
26.1119	25.9194	26.0289	25.9837	25.6084	26.0336	26.4453	26.2546	26.9136	25.9706	25.6927	26.5015	P46934-4;H0Y8H4	P46934-4	E3 ubiquitin-prote	NEDD4
26.9067	25.6892	25.868	26.121	24.6414	25.3382	25.2966	23.456	23.8941	24.9731	23.8232	23.2607	Q96PU5;Q96PU5-	Q96PU5;Q96PU5-	E3 ubiquitin-prote	NEDD4L
27.0077	27.8326	27.9886	27.2247	27.3884	27.8171	27.6611	25.8679	26.3621	28.5125	28.1117	27.556	Q15843;S4R3E9;F	Q15843;S4R3E9	NEDD8	NEDD8;NEDD8-MI
NaN	NaN	NaN	NaN	NaN	NaN	NaN	NaN	NaN	22.3017	22.3714	NaN	E9PL57;Q86V88;C	E9PL57;Q86V88	Magnesium-depe	NEDD8-MDP1;MD
26.0126	25.0772	25.6843	25.8845	24.9181	25.5421	24.9957	NaN	25.7035	25.6439	25.6372	25.5044	Q8TDX7;F8WAG2	Q8TDX7;F8WAG2	Serine/threonine-	NEK7
26.7956	26.4841	26.7475	26.3543	26.2534	26.37	27.0621	26.8604	27.5871	26.5481	26.6675	27.035	Q8TD19;G3V2Z5;(	Q8TD19	Serine/threonine-	NEK9
21.7262	NaN	21.7213	22.0423	NaN	21.8016	NaN	NaN	NaN	NaN	NaN	NaN	Q9H3P2;A0A0C4E	Q9H3P2;A0A0C4E	Negative elongati	NELFA
24.6983	23.6954	24.0138	24.724	24.5381	24.5346	NaN	NaN	NaN	24.9034	24.5778	NaN	A0A0X1KG71;Q8V	A0A0X1KG71;Q8V	Negative elongati	NELFB
24.0693	22.5332	23.5384	24.0062	23.0881	23.6721	NaN	NaN	NaN	23.223	23.2911	NaN	Q8IXH7;Q8IXH7-4	Q8IXH7;Q8IXH7-4	Negative elongati	NELFCD;TH1L
22.8347	NaN	23.3028	23.576	NaN	NaN	NaN	NaN	NaN	NaN	22.4409	NaN	P18615;P18615-4	P18615;P18615-4	Negative elongati	NELFE
26.6675	25.9465	26.1942	26.7974	26.0114	26.3074	25.0697	25.0403	25.7206	25.8875	26.2613	25.6342	O60524;O60524-4	O60524;O60524-4	Nuclear export m	NEMF
23.6464	25.2648	24.2792	23.5596	25.06	24.2434	24.7445	25.8553	25.1918	24.2611	24.6114	24.3243	Q9UMX5	Q9UMX5	Neudesin	NENF
23.6909	24.0885	24.8579	23.4789	23.9313	24.2814	25.6968	25.6296	25.09	25.2385	24.2603	24.7882	Q92859;Q92859-4	Q92859;Q92859-4	Neogenin	NEO1
24.1743	24.4325	23.9904	24.5465	24.1999	23.8997	24.3865	25.3546	24.1	24.0177	NaN	24.9844	P48681	P48681	Nestin	NES
22.8744	22.9598	NaN	22.1831	23.2791	NaN	23.9773	24.3586	24.1777	NaN	23.8471	23.4581	Q99519;E9PIF4	Q99519;E9PIF4	Sialidase-1	NEU1
25.3369	24.8655	25.3256	25.2145	24.7209	25.3923	25.427	25.5284	25.1229	25.8069	25.3307	25.4919	Q0ZGT2;H7BXY5;(	Q0ZGT2;H7BXY5;(	Nexilin	NEXN
22.9395	NaN	22.5615	22.2192	NaN	NaN	NaN	NaN	NaN	NaN	NaN	NaN	Q13469;Q13469-3	Q13469;Q13469-3	Nuclear factor of a	NFATC2
25.5274	25.0078	25.1219	25.5713	25.3677	25.3334	24.6273	25.2659	NaN	25.245	25.6452	25.322	P19838;P19838-2	P19838;P19838-2	Nuclear factor NF-	NFKB1
26.9302	26.4777	26.6894	26.8603	26.7166	26.9519	26.4084	26.944	27.2261	26.4811	26.7442	27.0868	Q00653;Q00653-4	Q00653;Q00653-4	Nuclear factor NF-	NFKB2
23.1999	NaN	23.1052	23.115	NaN	22.9914	NaN	NaN	NaN	23.0363	NaN	NaN	Q15653;I3L4X3;G	Q15653;I3L4X3;G	NF-kappa-B inhibi	NFKBIB
23.8444	23.9973	23.622	23.7623	23.7298	23.547	NaN	23.9933	NaN	NaN	23.5228	NaN	O00221;A0A087W	O00221;A0A087W	NF-kappa-B inhibi	NFKBIE
25.1119	24.638	24.3429	25.0966	24.8707	24.7488	NaN	24.1884	24.9121	24.0474	24.3708	24.1357	Q9Y697;Q9Y697-2	Q9Y697;Q9Y697-2	Cysteine desulfur	NFS1
20.3718	NaN	NaN	NaN	NaN	NaN	NaN	NaN	NaN	NaN	NaN	NaN	Q9UMS0;C9J8Q1;	Q9UMS0;C9J8Q1;	NFU1 iron-sulfur c	NFU1
22.5296	22.5645	22.6311	22.8284	22.9394	23.2551	NaN	NaN	NaN	22.7544	22.8844	NaN	Q13952;Q5T6K7;E	Q13952;Q5T6K7;E	Nuclear transcript	NFYC
24.3762	NaN	23.1847	24.7907	NaN	NaN	NaN	NaN	NaN	NaN	NaN	NaN	Q8NEJ9;Q8NEJ9-2	Q8NEJ9;Q8NEJ9-2	Neuroguidin	NGDN
22.6243	NaN	23.0648	22.7261	22.2373	NaN	23.3909	NaN	NaN	NaN	23.1433	NaN	Q96IV0;Q96IV0-5;	Q96IV0;Q96IV0-5;	Peptide-N(4)-(N-a	NGLY1
26.7089	26.5975	26.5705	26.5625	26.3709	26.5221	26.7713	26.6461	25.4615	27.3586	27.2694	26.5773	Q8NBF2;Q8NBF2-	Q8NBF2;Q8NBF2-	NHL repeat-conta	NHLRC2
24.849	24.5137	24.3212	25.6017	25.451	25.0798	NaN	NaN	NaN	NaN	NaN	24.2933	Q9NX24;D6RC52;I	Q9NX24;D6RC52;I	H/ACA ribonucleo	NHP2
27.1907	26.9639	26.7432	27.6557	27.4172	26.9064	25.9829	NaN	25.862	26.5869	27.1998	26.6838	P55769;B1AHD1	P55769;B1AHD1	NHP2-like protein	NHP2L1
NaN	NaN	NaN	NaN	NaN	NaN	NaN	NaN	NaN	21.4245	NaN	NaN	Q6T4R5;Q6T4R5-2	Q6T4R5;Q6T4R5-2	Nance-Horan sync	NHS
24.5244	25.6562	25.0494	24.2876	25.4901	24.884	24.8911	25.3691	25.529	24.7653	25.2593	25.8281	Q9GZT8;Q9GZT8-	Q9GZT8;Q9GZT8-	NIF3-like protein 1	NIF3L1
25.3738	23.678	24.4289	25.4707	24.2914	24.6695	NaN	NaN	NaN	23.211	23.0282	NaN	Q9BYG3;C9J6C5;C	Q9BYG3;C9J6C5	MKI67 FHA domai	NIFK
26.2801	25.7949	26.0324	27.4237	26.5446	26.0946	25.2349	24.5997	NaN	25.6342	26.1029	NaN	Q9Y221;Q9Y221-2	Q9Y221;Q9Y221-2	60S ribosome sub	NIP7
25.1553	NaN	NaN	24.9365	24.2426	NaN	NaN	NaN	NaN	24.7403	NaN	NaN	Q6KC79;Q6KC79-	Q6KC79;Q6KC79-	Nipped-B-like pro	NIPBL
24.5687	24.975	24.787	24.9096	25.3079	24.7212	23.5062	24.3663	25.7726	23.7657	24.9066	24.545	Q9BPW8;C9JDV8;	Q9BPW8	Protein NipSnap h	NIPSNAP1
NaN	24.0323	NaN	NaN	23.3376	NaN	NaN	NaN	NaN	NaN	NaN	NaN	Q9UFN0	Q9UFN0	Protein NipSnap h	NIPSNAP3A
NaN	NaN	NaN	22.08	NaN	NaN	NaN	NaN	NaN	NaN	NaN	NaN	Q9Y2I1;C9J715;Q	Q9Y2I1;C9J715;Q	Nischarin	NISCH
24.8552	24.7235	24.4403	24.0759	24.7407	24.4702	24.1465	24.8269	24.2942	24.7458	24.4837	24.5308	Q86X76;Q86X76-2	Q86X76;Q86X76-2	Nitrilase homolog	NIT1
25.9222	25.98	25.5983	25.128	25.5871	25.4594	25.793	25.13	25.8403	25.6111	25.704	25.443	Q9NQR4;H7C579;	Q9NQR4;H7C579	Omega-amidase N	NIT2
NaN	NaN	NaN	21.0688	NaN	NaN	NaN	NaN	NaN	NaN	21.3418	NaN	Q9NYS0;G5E9P3	Q9NYS0;G5E9P3	NF-kappa-B inhibi	NKIRAS1
22.5042	22.5245	22.4591	22.5824	23.5442	NaN	NaN	NaN	NaN	NaN	NaN	NaN	Q9NYR9;K7ERG2;I	Q9NYR9;K7ERG2;I	NF-kappa-B inhibi	NKIRAS2

24.5734	23.8678	24.4452	25.0914	24.3176	24.4105	22.7761	24.175	NaN	23.8519	24.6405	23.8779	O15226;O15226-2	O15226;O15226-2	NF-kappa-B-repre	NKRF
24.1092	24.1784	24.1061	24.6236	24.4891	24.1049	24.4723	NaN	NaN	24.5958	24.7956	24.6087	Q9NVX2;K7EN33;	Q9NVX2;K7EN33;	Notchless protein	NLE1
24.2114	24.1739	23.718	24.3253	24.0676	23.661	23.6491	NaN	24	23.4224	NaN	NaN	Q9BYT8;E9PCB6;H	Q9BYT8;E9PCB6	Neurolysin, mitoc	NLN
22.8347	NaN	NaN	22.4877	NaN	NaN	NaN	NaN	NaN	NaN	NaN	NaN	Q9C000;I3L2G5;Q	Q9C000;I3L2G5;Q	NACHT, LRR and P	NLRP1
26.6794	25.5221	26.5311	26.3246	25.7196	26.4228	NaN	NaN	NaN	NaN	NaN	28.036	Q9NX02;A0A0G2J	Q9NX02;A0A0G2J	NACHT, LRR and P	NLRP2
25.3974	NaN	24.4677	25.474	23.7778	24.6162	24.2574	NaN	NaN	25.1138	24.5216	25.0015	Q96D46;C9JA08;C	Q96D46;C9JA08;C	60S ribosomal exp	NMD3
24.8231	24.589	24.0582	23.7079	NaN	24.2974	NaN	NaN	NaN	24.6109	24.5795	NaN	P15531;P15531-2	P15531;P15531-2	Nucleoside diphos	NME1
31.3765	31.9618	31.5891	31.2082	31.4239	31.5476	31.6512	31.088	30.6888	31.7748	31.3572	31.2878	P22392-2;Q32Q12	P22392-2;Q32Q12	Nucleoside diphos	NME2;NME1-NME
24.8065	24.2336	24.2633	24.4758	23.7852	24.1781	24.4397	NaN	23.8945	24.5479	24.482	NaN	Q9Y5B8;E9PNU1;I	Q9Y5B8;E9PNU1;I	Nucleoside diphos	NME7
24.3993	24.0299	23.8646	23.5881	24.0334	23.8627	23.7844	NaN	NaN	23.2549	23.2552	NaN	Q13287	Q13287	N-myc-interactor	NMI
24.7945	24.8011	24.4767	25.1437	25.0825	24.6901	NaN	NaN	NaN	24.1487	23.9837	NaN	Q9HAN9;B1AN62	Q9HAN9;B1AN62	Nicotinamide/nicc	NMNAT1
NaN	NaN	NaN	NaN	NaN	NaN	NaN	NaN	NaN	22.6153	23.6584	NaN	Q9HBL8;I3L3Z0;I3	Q9HBL8;I3L3Z0;I3	NmrA-like family c	NMRAL1
26.8583	25.8922	26.3432	26.1785	26.0727	25.8806	24.8249	23.7867	25.4497	25.757	26.1851	25.7308	P30419;P30419-2	P30419;P30419-2	Glycylpeptide N-te	NMT1
24.597	23.1222	23.3836	24.1681	22.561	NaN	NaN	NaN	NaN	23.2532	23.1833	NaN	O60551;Q5VUC6	O60551;Q5VUC6	Glycylpeptide N-te	NMT2
24.9732	25.0215	25.0708	25.6031	25.383	25.1896	24.4442	25.3558	26.2227	24.0142	24.3885	24.6606	Q13423;E9PCX7;D	Q13423;E9PCX7;D	NAD(P) transhydro	NNT
25.6144	25.0201	24.9682	26.1943	25.5643	25.4411	23.3615	NaN	NaN	25.3544	25.3813	NaN	Q9H6W3;Q9H6W	Q9H6W3;Q9H6W	Bifunctional lysine	NO66
NaN	NaN	NaN	22.977	NaN	NaN	NaN	NaN	NaN	22.969	23.0141	NaN	Q9ULX3;H3BUR4	Q9ULX3;H3BUR4	RNA-binding prote	NOB1
25.5629	23.8725	23.7316	25.864	24.4203	24.5634	NaN	NaN	NaN	23.6173	24.0924	NaN	Q9Y3T9	Q9Y3T9	Nucleolar comple:	NOC2L
24.6393	23.8938	24.3662	25.0364	24.2353	24.5778	NaN	NaN	NaN	24.4843	24.4003	24.1628	Q9BVI4;F5H303	Q9BVI4	Nucleolar comple:	NOC4L
26.4947	24.9939	25.7702	24.855	NaN	NaN	26.4498	26.1878	NaN	24.5682	NaN	NaN	Q13253	Q13253	Noggin	NOG
24.7342	NaN	23.2952	25.0882	NaN	NaN	NaN	NaN	NaN	23.8959	NaN	NaN	Q9BSC4;Q9BSC4-	Q9BSC4;Q9BSC4-	Nucleolar protein	NOL10
24.6794	23.6556	24.2421	24.9603	23.9536	23.8189	NaN	NaN	NaN	24.1272	23.8801	NaN	Q9H8H0;Q9H8H0	Q9H8H0	Nucleolar protein	NOL11
23.2027	23.304	23.2076	22.9342	22.7114	22.9125	24.1106	23.343	NaN	23.9396	23.8168	24.6089	O60936;H3BM67;	O60936;H3BM67;	Nucleolar protein	NOL3
25.2043	22.9863	24.2353	25.5357	24.2933	24.7699	NaN	NaN	NaN	24.1908	24.7304	NaN	Q9H6R4;Q9H6R4-	Q9H6R4;Q9H6R4-	Nucleolar protein	NOL6
24.4528	23.4193	24.6686	25.2794	24.4221	24.7148	NaN	NaN	NaN	23.984	23.7291	NaN	Q5SY16	Q5SY16	Polynucleotide 5-l	NOL9
25.2203	24.5491	24.8319	25.6331	24.7841	24.7767	25.6359	NaN	NaN	25.3739	25.4811	26.091	Q14978;A0A0A0N	Q14978;A0A0A0N	Nucleolar and coil	NOLC1
23.9239	23.0837	23.7536	24.3795	23.6594	23.9809	NaN	NaN	NaN	NaN	23.9104	NaN	Q5C9Z4	Q5C9Z4	Nucleolar MIF4G c	NOM1
NaN	NaN	NaN	NaN	NaN	NaN	NaN	NaN	NaN	NaN	NaN	22.4694	Q15155;A0A0G2JI	Q15155;A0A0G2JI	Nodal modulator	NOMO1
25.7954	26.0295	26.0074	25.75	26.082	26.0192	25.8065	26.5841	26.5754	25.934	25.9444	25.9193	Q5JPE7;P69849;J3	Q5JPE7;P69849;J3	Nodal modulator	NOMO2;NOMO3
30.0148	29.8894	29.9894	30.1972	30.3342	30.2255	28.3594	29.6373	29.4462	30.0691	30.4737	30.27	Q15233;Q15233-2	Q15233;Q15233-2	Non-POU domain	NONO
26.7313	27.2401	27.0926	27.7369	28.1209	26.96	25.3031	26.6849	NaN	26.3489	27.3128	26.6889	Q9NPE3;H0YM60	Q9NPE3	H/ACA ribonucleo	NOP10
24.178	22.164	22.9339	24.1613	22.0385	22.6411	NaN	NaN	NaN	22.4065	22.3662	NaN	P78316;E9PFK5;P	P78316;E9PFK5;P	Nucleolar protein	NOP14
23.42	22.9877	22.7296	23.7972	23.3782	23.1028	NaN	NaN	NaN	NaN	22.8174	NaN	Q9Y3C1;Q9Y3C1-3	Q9Y3C1;Q9Y3C1-3	Nucleolar protein	NOP16
27.9847	27.0648	27.3703	28.6869	28.1861	27.5943	25.8706	24.2817	25.2843	27.4346	27.7929	26.8345	P46087;A0A087W	P46087;A0A087W	Probable 28S rRN	NOP2
29.148	28.0669	28.8577	30.028	29.0749	29.087	27.1421	24.285	26.4516	28.3228	29.0159	28.1517	O00567;H0YDU4;I	O00567	Nucleolar protein	NOP56
28.2194	27.0593	27.9414	29.1472	28.2675	27.8773	25.7047	NaN	23.7265	27.7581	27.9778	26.8735	Q9Y2X3;H7BZ72;F	Q9Y2X3	Nucleolar protein	NOP58
NaN	NaN	NaN	19.9285	NaN	NaN	NaN	NaN	NaN	NaN	NaN	NaN	Q86U38	Q86U38	Nucleolar protein	NOP9
24.788	24.1975	25.8993	25.3841	25.0441	25.8516	24.5881	NaN	NaN	26.4617	25.9252	25.702	O75052;O75052-3	O75052;O75052-3	Carboxyl-terminal	NOS1AP
25.5956	25.4954	24.9547	25.1794	24.6571	24.9983	NaN	24.8359	24.3759	NaN	NaN	NaN	Q9Y314;A0A075B	Q9Y314;A0A075B	Nitric oxide syntha	NOSIP
23.1422	23.3123	23.6341	23.1755	23.7151	23.5185	NaN	NaN	23.8848	25.093	24.4681	23.3547	P46531;M0QX38	P46531	Neurogenic locus	NOTCH1
26.3342	26.7141	26.9304	26.4139	26.6942	27.1498	26.8498	26.0997	26.4459	27.2215	26.646	26.7747	Q04721	Q04721	Neurogenic locus	NOTCH2
27.6237	27.9087	27.8941	27.6788	27.3323	27.9246	27.7362	27.5323	28.8588	27.5136	27.3406	28.2114	O15118;K7EQ23;C	O15118;K7EQ23;C	Niemann-Pick C1	NPC1
23.5154	23.4732	22.7815	23.2698	NaN	NaN	26.8042	23.8464	NaN	NaN	NaN	NaN	P61916;J3KMY5;H	P61916;J3KMY5;H	Epididymal secret	NPC2
28.2869	29.0089	28.8517	28.1315	28.8976	28.8805	28.4862	28.3852	28.6292	28.4201	28.575	28.7837	P55786;E9PLK3;P	P55786;E9PLK3;P	Puromycin-sensiti	NPEPPS
26.714	26.2181	26.2867	26.213	26.1326	26.3491	26.2252	NaN	26.5564	26.1131	26.0939	26.4666	Q8TAT6;Q8TAT6-2	Q8TAT6;Q8TAT6-2	Nuclear protein lo	NPLOC4
32.6496	32.3174	32.7546	33.0762	32.8679	32.9403	31.727	32.5402	31.4637	32.3342	32.9029	32.6318	P06748;P06748-2	P06748;P06748-2	Nucleophosmin	NPM1
NaN	25.2182	NaN	NaN	26.8163	NaN	NaN	25.9845	NaN	NaN	NaN	NaN	P06748-3;E5RI98;	P06748-3	Nucleophosmin	NPM1
27.098	25.8743	26.976	27.4917	27.1475	26.4349	25.0119	NaN	25.8666	25.7423	26.3797	26.8959	O75607	O75607	Nucleoplasmin-3	NPM3
28.3271	28.4971	28.469	28.3543	28.5944	28.3138	28.867	27.501	28.3624	28.9668	28.1968	28.3377	Q9Y639;Q9Y639-1	Q9Y639;Q9Y639-1	Neuroplastin	NPTN;DKFZp566H
25.4419	27.0832	25.2647	24.3842	26.2688	25.4507	25.9392	25.2909	25.6902	24.8943	25.3643	25.2635	P16083;Q5TD07;C	P16083;Q5TD07;C	Ribosyldihydronic	NQO2
23.9876	23.3465	23.19	23.873	23.2521	23.2244	23.3291	NaN	NaN	22.9951	23.2443	NaN	Q86WQ0;Q86WQ	Q86WQ0;Q86WQ	Nuclear receptor :	NR2C2AP
23.337	NaN	22.9977	22.9674	22.5466	22.9211	NaN	NaN	NaN	NaN	NaN	NaN	P04150;P04150-9	P04150;P04150-9	Glucocorticoid rec	NR3C1
28.1547	28.6106	29.0163	28.2545	28.7245	29.1085	28.955	28.6238	28.9771	29.0811	28.9418	29.2138	P01111	P01111	GTPase NRas	NRAS
22.5943	NaN	NaN	NaN	NaN	NaN	NaN	NaN	NaN	NaN	NaN	NaN	Q96F24;A0A087W	Q96F24;A0A087W	Nuclear receptor-	NRBF2
25.6068	25.0513	25.3745	25.2221	24.9012	24.9843	25.6557	25.4821	24.9969	25.3241	24.8775	25.25	Q9UHY1;F8W6G1	Q9UHY1;F8W6G1	Nuclear receptor-	NRBP1
24.6215	24.5722	24.9848	24.862	24.8553	25.0941	25.0039	25.0944	24.578	24.7759	24.9145	24.869	O43847;O43847-2	O43847;O43847-2	Nardilysin	NRD1
28.3371	28.3411	28.9009	28.2858	28.1023	29.0034	29.3923	29.059	28.9134	29.8557	29.292	29.3533	O14786;E9PEP6;C	O14786;E9PEP6;C	Neuropilin-1	NRP1
NaN	NaN	NaN	20.9227	NaN	NaN	NaN	NaN	NaN	NaN	NaN	NaN	Q9GZP1;A0A087V	Q9GZP1;A0A087V	Neurensin-2	NRSN2
23.8548	NaN	23.2137	24.1045	NaN	NaN	22.7621	NaN	NaN	NaN	22.9527	22.7117	O95478;A0A0A0N	O95478	Ribosome biogene	NSA2
24.5087	24.6124	24.6676	25.1958	24.6181	24.6168	25.2671	25.042	25.2622	24.5176	24.3574	24.3262	Q15738;C9JDR0	Q15738;C9JDR0	Sterol-4-alpha-car	NSDHL

28.8192	28.2314	28.5084	28.8986	28.5127	28.6105	27.7914	27.9507	27.8813	27.8724	28.0749	27.4855	P46459;I3L0N3;P4	P46459;I3L0N3;P4	Vesicle-fusing ATF	NSF
26.4461	27.1801	26.8064	26.4507	26.9602	26.9197	27.357	27.1414	27.1379	27.2503	27.4315	27.4744	Q9UNZ2;Q9UNZ2	Q9UNZ2;Q9UNZ2	NSFL1 cofactor p4	NSFL1C
22.8613	22.8656	22.328	NaN	22.656	NaN	NaN	NaN	NaN	23.951	22.3236	NaN	Q92636;Q92636-2	Q92636;Q92636-2	Protein FAN	NSMAF
22.8221	22.1668	22.2438	22.4684	22.3935	NaN	NaN	NaN	NaN	21.836	22.3511	NaN	Q8WV22;H3BSL0;	Q8WV22;H3BSL0;	Non-structural ma	NSMCE1
28.7505	27.4781	27.8112	28.5723	27.8175	27.9222	26.6306	27.4056	26.7338	28.5085	28.1198	27.5237	Q08J23;Q08J23-2;	Q08J23;Q08J23-2;	tRNA (cytosine(34	NSUN2
22.6038	NaN	NaN	22.4268	NaN	NaN	NaN	NaN	NaN	NaN	NaN	NaN	Q96P11;Q96P11-2	Q96P11;Q96P11-2	Probable 28S rRN	NSUN5;NSUN5P2
NaN	NaN	NaN	NaN	NaN	NaN	NaN	NaN	NaN	NaN	NaN	19.8661	Q8TEA1;U3KQU2	Q8TEA1	Putative methyltr	NSUN6
23.2098	23.7394	24.237	23.2911	24.326	NaN	NaN	NaN	NaN	23.5768	23.8647	NaN	Q8TCD5;J3KSY6;J3	Q8TCD5;J3KSY6;J3	5(3)-deoxyribonuc	NT5C
24.3617	23.9396	24.2137	24.0759	23.9822	24.1252	24.7546	NaN	23.5247	24.3299	23.7063	23.461	P49902;P49902-2	P49902;P49902-2	Cytosolic purine 5	NT5C2
22.5726	22.7998	22.5843	22.5679	22.8749	NaN	NaN	NaN	NaN	22.3428	21.6934	NaN	Q9H0P0;Q9H0P0-	Q9H0P0;Q9H0P0-	Cytosolic 5-nuclec	NT5C3A
23.2525	24.3806	23.8617	23.2593	23.7004	23.4215	22.6794	NaN	NaN	23.259	23.8662	NaN	Q5TFE4;Q5TFE4-2	Q5TFE4;Q5TFE4-2	5-nucleotidase do	NT5DC1
22.5174	NaN	NaN	22.1681	NaN	NaN	NaN	NaN	NaN	NaN	NaN	NaN	Q9H857-2;Q9H85	Q9H857-2;Q9H85	5-nucleotidase do	NT5DC2
32.5899	32.3847	33.1841	32.7775	32.3787	33.0971	32.5879	31.8288	32.5815	33.0536	32.502	32.6784	P21589;P21589-2	P21589;P21589-2	5-nucleotidase	NT5E
25.6403	24.8477	25.0036	25.2912	25.2712	25.0458	24.8056	24.9469	NaN	25.8905	25.5984	24.0398	Q9BV86;Q9BV86-	Q9BV86;Q9BV86-	N-terminal Xaa-Pr	NTMT1
24.5798	24.4019	24.2252	24.593	24.4987	24.4699	NaN	NaN	NaN	23.9968	24.2458	23.9002	Q9BSD7;Q5TDF0	Q9BSD7;Q5TDF0	Cancer-related nu	NTPCR
24.1675	23.2309	23.6754	23.9138	23.4979	23.9522	NaN	NaN	NaN	23.1276	23.0683	NaN	Q9Y5A7;H3BM14;	Q9Y5A7;H3BM14;	NEDD8 ultimate b	NUB1
24.4717	23.5592	23.6961	24.0364	NaN	NaN	NaN	NaN	NaN	NaN	NaN	NaN	P53384;P53384-2	P53384;P53384-2	Cytosolic Fe-S clus	NUBP1
24.1584	NaN	23.6081	24.2201	23.9903	23.4437	NaN	NaN	NaN	NaN	NaN	NaN	H3BNF0;Q9Y5Y2;H	H3BNF0;Q9Y5Y2;H	Cytosolic Fe-S clus	NUBP2
NaN	NaN	NaN	22.2668	NaN	NaN	NaN	NaN	NaN	NaN	NaN	NaN	Q8TB37;F8W061;(	Q8TB37;F8W061;(	Iron-sulfur protein	NUBPL
27.9664	29.0086	28.4915	27.767	29.0044	28.6956	30.4957	29.9091	29.7292	28.5947	28.5942	28.9836	Q02818;H7BZI1;C	Q02818;H7BZI1;C	Nucleobindin-1	NUCB1
25.1019	25.0663	25.5284	25.4134	25.9339	25.8521	NaN	NaN	25.2477	25.3513	25.9222	26.6996	Q9H1E3;Q9H1E3-	Q9H1E3;Q9H1E3-	Nuclear ubiquitou	NUCKS1
29.4172	29.6445	29.4968	29.0335	29.3916	29.2902	29.784	30.0409	29.9787	29.5714	29.9067	30.1419	Q9Y266;A0A0A0N	Q9Y266	Nuclear migration	NUDC
28.716	28.4324	28.3924	28.5794	28.4858	28.3809	28.1934	28.3682	28.0408	28.2716	28.2826	28.2634	Q96RS6;Q96RS6-2	Q96RS6;Q96RS6-2	NudC domain-con	NUDCD1
25.7013	25.9631	25.546	25.4798	25.6251	25.7103	25.6826	26.1653	25.2858	25.4352	25.6759	25.8425	Q8WVJ2;E5RFP0	Q8WVJ2;E5RFP0	NudC domain-con	NUDCD2
NaN	NaN	NaN	23.6591	NaN	NaN	NaN	NaN	NaN	NaN	NaN	NaN	P36639;P36639-4	P36639;P36639-4	7,8-dihydro-8-oxo	NUDT1
22.2568	NaN	22.4831	NaN	22.6066	NaN	NaN	NaN	NaN	22.737	23.0839	NaN	Q96DE0;Q96DE0-	Q96DE0;Q96DE0-	U8 snoRNA-decap	NUDT16
23.0358	NaN	NaN	23.2842	NaN	NaN	NaN	NaN	NaN	NaN	NaN	NaN	P50583	P50583	Bis(5-nucleosyl)-te	NUDT2
25.9869	26.4757	26.5114	26.4817	26.989	26.6895	25.363	26.5621	26.0414	27.059	27.5014	27.4543	O43809;H3BND3;	O43809;H3BND3	Cleavage and poly	NUDT21
26.2304	24.7539	25.0205	25.276	24.2452	24.2327	25.2053	NaN	NaN	24.9165	25.0015	NaN	Q9NZJ9-2;Q9NZJ9	Q9NZJ9-2;Q9NZJ9	Diphosphoinosito	NUDT4;NUDT11;N
27.0073	28.1483	27.2501	26.6492	27.9459	27.2208	28.0253	28.8593	28.1805	27.5387	27.9884	28.4138	Q9UKK9;A6NFX8;	Q9UKK9;A6NFX8;	ADP-sugar pyroph	NUDT5
22.5468	22.749	22.6201	21.9662	23.0123	22.7201	23.6041	NaN	NaN	22.7955	23.0507	23.3488	Q9BW91;Q9BW9	Q9BW91;Q9BW9	ADP-ribose pyrop	NUDT9
24.911	24.4046	24.7461	24.9984	24.0918	24.6205	23.63	23.9804	NaN	24.9846	24.5644	NaN	Q9BZD4;E9PQC4;I	Q9BZD4;E9PQC4;I	Kinetochore prote	NUF2
24.9955	24.7196	24.5115	24.6545	24.2621	24.1587	NaN	NaN	NaN	NaN	24.1272	NaN	Q7Z417	Q7Z417	Nuclear fragile X r	NUFIP2
NaN	NaN	NaN	NaN	NaN	NaN	NaN	NaN	NaN	NaN	NaN	24.9721	Q7Z417-2	Q7Z417-2	Nuclear fragile X r	NUFIP2
26.1899	26.3743	27.1561	26.687	26.9293	26.9362	25.9905	26.4993	23.9556	26.8606	27.0618	27.626	Q14980;Q14980-2	Q14980;Q14980-2	Nuclear mitotic ap	NUMA1
26.4254	27.2521	27.2546	27.0717	27.3929	27.6828	27.0157	26.5782	26.6488	27.7359	27.4238	27.4975	P49757;P49757-2	P49757;P49757-2	Protein numb hor	NUMB
25.4212	23.9144	24.9958	25.5062	24.7815	25.2792	NaN	NaN	24.5685	25.1567	25.2594	24.8711	P57740;P57740-2	P57740;P57740-2	Nuclear pore com	NUP107
24.612	23.774	24.8803	25.6445	24.7311	25.0261	24.3535	NaN	24.3664	24.8043	25.026	24.0744	Q8WUM0	Q8WUM0	Nuclear pore com	NUP133
22.9018	22.9372	22.862	22.819	23.1364	NaN	NaN	NaN	NaN	22.6682	22.888	NaN	P49790;P49790-2	P49790;P49790-2	Nuclear pore com	NUP153
26.4413	25.5575	26.2558	26.3052	25.9094	26.1509	25.7216	26.6722	26.1963	26.2737	26.3608	26.1785	O75694;E9PF10;O	O75694;E9PF10;O	Nuclear pore com	NUP155
24.8847	24.1756	24.536	25.2109	24.2865	24.7223	NaN	NaN	NaN	24.6328	24.653	NaN	Q12769;G3V198;E	Q12769;G3V198;E	Nuclear pore com	NUP160
23.6405	NaN	22.7911	23.5676	23.124	21.8523	NaN	NaN	NaN	22.9314	23.0383	22.1023	Q5SRE5;Q5SRE5-2	Q5SRE5;Q5SRE5-2	Nucleoporin NUP1	NUP188
25.8885	24.4377	25.6946	26.6867	25.5781	25.8149	24.4091	NaN	24.7162	25.988	26.0632	25.8398	Q92621;U3KPX2;U	Q92621	Nuclear pore com	NUP205
NaN	NaN	NaN	22.0826	NaN	NaN	NaN	NaN	NaN	NaN	NaN	NaN	Q8TEM1;Q8TEM1	Q8TEM1;Q8TEM1	Nuclear pore men	NUP210
23.473	23.757	23.0326	22.3445	23.2124	NaN	NaN	NaN	NaN	23.0395	23.3597	NaN	P35658;P35658-3	P35658;P35658-3	Nuclear pore com	NUP214
25.7564	25.6399	25.2213	25.5215	25.7686	25.2258	25.0702	25.826	25.6433	25.8649	26.2924	25.6234	Q8NFH4;F8VTY2	Q8NFH4;F8VTY2	Nucleoporin Nup3	NUP37
24.5052	24.3205	24.3864	24.4903	24.3084	24.1929	NaN	NaN	23.9135	24.5162	24.9886	24.2094	Q8NFH3;Q8NFH3-	Q8NFH3;Q8NFH3-	Nucleoporin Nup4	NUP43
24.1124	24.671	23.869	24.0365	24.0396	24.0715	NaN	NaN	NaN	NaN	NaN	24.9439	Q9UKX7;Q9UKX7-	Q9UKX7;Q9UKX7-	Nuclear pore com	NUP50
23.309	NaN	22.683	22.9586	23.1013	NaN	NaN	NaN	NaN	NaN	NaN	23.8219	Q7Z3B4;Q7Z3B4-3	Q7Z3B4;Q7Z3B4-3	Nucleoporin p54	NUP54
23.9622	24.6858	24.0921	24.3629	24.7773	24.0846	23.9736	NaN	NaN	24.1725	NaN	24.6603	P37198;M0QXN5	P37198;M0QXN5	Nuclear pore glyc	NUP62
26.9824	24.9727	25.6563	26.3655	25.3785	25.6537	NaN	NaN	NaN	26.0203	26.0352	24.7901	Q9BW27;Q9BW27	Q9BW27;Q9BW27	Nuclear pore com	NUP85
26.3245	25.4837	25.8106	26.2023	25.8425	25.8627	25.0796	25.5096	25.3984	25.676	25.3583	25.6426	Q99567;J3KMX1;I	Q99567;J3KMX1	Nuclear pore com	NUP88
26.3644	25.7884	26.2473	26.4939	26.1085	26.4342	26.348	26.2707	26.3568	26.7394	27.0163	26.7863	Q8N1F7;H3BVG0;	Q8N1F7;H3BVG0;	Nuclear pore com	NUP93
24.711	24.5875	24.9732	25.1024	25.1273	25.0186	24.6206	NaN	NaN	24.5747	25.0915	24.6968	P52948;P52948-5	P52948;P52948-5	Nuclear pore com	NUP98
26.0541	NaN	25.9237	25.7082	NaN	NaN	NaN	NaN	NaN	NaN	26.4166	26.1134	Q9BVL2;Q5JRG1;I	Q9BVL2;Q5JRG1;I	Nucleoporin p58/p	NUPL1
27.3842	27.9377	27.2539	26.7558	27.6246	27.5107	28.1829	27.8621	28.3179	27.9576	28.1647	27.9055	P61970;H3BRV9	P61970;H3BRV9	Nuclear transport	NUTF2
21.3706	NaN	NaN	NaN	NaN	NaN	NaN	NaN	NaN	NaN	NaN	NaN	O15381;O15381-3	O15381;O15381-3	Nuclear valosin-cc	NVL
25.7988	25.0923	25.528	26.1034	25.1162	25.4358	24.0526	NaN	24.0458	25.9506	25.6558	25.4538	Q9UBU9;E9PIN3;(	Q9UBU9;E9PIN3;(	Nuclear RNA expc	NXF1
24.8888	24.8942	24.4727	24.7349	24.5716	24.7713	24.9464	24.9205	24.8005	24.5157	25.1717	25.2111	Q6DKJ4;Q6DKJ4-2	Q6DKJ4;Q6DKJ4-2	Nucleoredoxin	NXN
22.2203	NaN	NaN	22.7071	NaN	NaN	NaN	NaN	NaN	NaN	NaN	NaN	Q9UKK6	Q9UKK6	NTF2-related expc	NXT1

NaN	NaN	NaN	NaN	NaN	NaN	24.9238	NaN	NaN	NaN	NaN	NaN	E9PJ29;Q86UD1	E9PJ29;Q86UD1	Out at first protei	OAF
25.328	25.9176	25.5788	25.4052	25.9113	25.7186	25.7117	25.443	25.3071	25.7338	26.3858	25.762	Q9Y530;C9JNE2;C	Q9Y530;C9JNE2;C	O-acetyl-ADP-ribo	OARD1
25.0903	23.5211	23.6994	24.7261	23.8367	23.7559	NaN	NaN	NaN	NaN	23.22	NaN	Q9Y6K5;F8VWK9;	Q9Y6K5	2-5-oligoadenylat	OAS3
23.6859	NaN	22.7848	23.3842	23.4504	23.0413	NaN	NaN	NaN	22.6277	NaN	NaN	Q15646;Q15646-3	Q15646;Q15646-3	2-5-oligoadenylat	OASL
29.2229	28.9885	28.8561	29.3349	29.0249	28.8197	27.6224	28.1386	28.816	29.4013	29.7198	28.1256	P04181;P04181-2	P04181;P04181-2	Ornithine aminotr	OAT
26.8243	26.3333	27.1332	26.7027	26.678	26.9022	25.359	NaN	NaN	27.7914	26.5475	NaN	Q8TAD7;F8VQD4	Q8TAD7;F8VQD4	Overexpressed in	OCC1;C12orf75
21.2106	21.2202	21.778	22.1612	21.8537	NaN	NaN	NaN	NaN	20.9769	NaN	20.4153	Q56VL3	Q56VL3	OCIA domain-con	OCIAD2
25.4981	25.9254	26.0272	25.6549	25.7952	26.2064	24.7087	24.625	24.9454	25.2069	24.8212	24.9952	Q16625;Q16625-3	Q16625;Q16625-3	Occludin	OCLN
24.9946	NaN	24.5949	25.1723	24.6309	NaN	24.9328	NaN	24.6427	24.9666	24.9352	NaN	Q01968;Q01968-2	Q01968;Q01968-2	Inositol polyphosp	OCRL
23.1211	NaN	NaN	NaN	NaN	NaN	23.1277	NaN	NaN	NaN	NaN	NaN	Q5SWX8;Q5SWX8	Q5SWX8;Q5SWX8	Protein odr-4 hom	ODR4
26.7068	26.7401	26.7388	27.3106	27.4252	26.6902	24.5671	26.9738	27.657	25.239	26.0841	26.5979	Q02218;A0A0D9S	Q02218;A0A0D9S	2-oxoglutarate de	OGDH
22.0443	NaN	21.94	21.8456	NaN	NaN	NaN	23.308	22.6388	22.3352	22.5336	22.8961	Q8N543;H3BUA6;	Q8N543;H3BUA6;	Prolyl 3-hydroxyla	OGFOD1
26.843	25.5849	26.3189	26.5501	25.9061	26.2362	25.6424	26.1477	25.9323	26.3795	26.3617	25.6096	Q9NZT2;Q9NZT2-	Q9NZT2;Q9NZT2-	Opioid growth fac	OGFR
NaN	NaN	22.3212	NaN	NaN	NaN	NaN	NaN	NaN	NaN	NaN	NaN	Q5TC84	Q5TC84	Opioid growth fac	OGFRL1
25.3528	24.4458	24.5029	25.5974	25.1333	24.7364	24.3111	23.7048	24.5477	25.0918	25.4431	24.7762	O15294;O15294-3	O15294;O15294-3	UDP-N-acetylgluc	OGT
NaN	NaN	NaN	NaN	NaN	NaN	NaN	NaN	NaN	NaN	21.0061	NaN	O43482	O43482	Protein Mis18-bet	OIP5
28.7993	28.8831	28.7836	28.4161	28.7395	28.6919	28.689	28.263	28.3461	29.0063	28.7301	28.7406	Q9NTK5;J3KQ32;(	Q9NTK5;J3KQ32;(	Obg-like ATPase 1	OLA1
NaN	NaN	NaN	NaN	NaN	NaN	NaN	26.6099	28.4166	NaN	25.1308	28.1222	H7BZX2;Q96E52-2	H7BZX2;Q96E52-2		OMA1
26.6882	27.1599	26.4569	26.6223	26.9878	26.3922	26.3024	26.1591	26.3255	25.8414	25.9117	26.1572	O60313;O60313-2	O60313;O60313-2	Dynamin-like 120	OPA1
22.3458	22.327	22.7361	22.4902	22.5711	23.0208	NaN	23.6105	22.8644	22.5596	23.4803	NaN	O14841	O14841	5-oxoprolinase	OPLAH
25.5502	25.5687	26.3183	24.9811	24.3726	25.0848	25.7974	24.415	24.966	24.3638	23.775	NaN	Q96CV9;Q96CV9-	Q96CV9;Q96CV9-	Optineurin	OPTN
24.8201	24.9623	25.4019	24.7077	25.2203	26.3182	25.4356	NaN	26.2022	25.079	25.3529	25.4403	A0A0B4J2E8;Q96I	A0A0B4J2E8;Q96I	Calcium release-a	ORAI1
25.2652	NaN	23.7812	24.9675	24.211	24.0354	NaN	NaN	NaN	24.0858	NaN	NaN	Q9UBD5;Q9UBD5	Q9UBD5;Q9UBD5	Origin recognition	ORC3
22.0179	NaN	NaN	22.6215	NaN	NaN	NaN	NaN	NaN	21.6424	22.0927	21.9326	O43929;O43929-2	O43929;O43929-2	Origin recognition	ORC4
23.1767	NaN	NaN	23.273	22.3314	NaN	NaN	NaN	NaN	21.8515	NaN	NaN	O43913;O43913-2	O43913;O43913-2	Origin recognition	ORC5
NaN	NaN	NaN	NaN	NaN	NaN	NaN	NaN	NaN	NaN	21.5897	NaN	Q9Y5N6;H3BT22	Q9Y5N6;H3BT22	Origin recognition	ORC6
23.2212	22.9217	23.2778	NaN	22.9138	NaN	NaN	NaN	NaN	NaN	NaN	NaN	Q9P0S3;Q53FV1;F	Q9P0S3;Q53FV1;F	ORM1-like protein	ORMDL1;ORMDL2
NaN	NaN	NaN	22.5199	NaN	NaN	NaN	NaN	NaN	NaN	NaN	NaN	Q8N138;Q8N138-	Q8N138;Q8N138-	ORM1-like protein	ORMDL3
23.6277	23.9762	23.1408	23.2013	NaN	22.8635	24.9605	NaN	NaN	NaN	NaN	NaN	Q13438;B4E321;C	Q13438;B4E321;C	Protein OS-9	OS9
27.167	26.7526	26.8738	26.187	26.1613	26.2651	26.0671	25.7411	25.1145	26.1313	26.6017	26.3194	P22059;H0YCV6;C	P22059	Oxysterol-binding	OSBP
24.9901	24.0908	24.5173	24.8364	24.2584	24.5635	24.8588	24.7509	NaN	24.9849	25.0051	24.8398	Q9BXB5;Q9BXB5-	Q9BXB5;Q9BXB5-	Oxysterol-binding	OSBPL10
22.5319	NaN	22.9916	22.6554	NaN	NaN	NaN	NaN	NaN	NaN	NaN	NaN	Q9BXB4	Q9BXB4	Oxysterol-binding	OSBPL11
27.8361	26.5988	26.95	27.7551	26.4887	26.9072	26.5761	26.1943	25.5864	26.6144	26.5948	25.4545	Q9H4L5;Q9H4L5-3	Q9H4L5;Q9H4L5-3	Oxysterol-binding	OSBPL3
24.7189	24.0992	24.3241	24.4377	23.6452	23.9021	24.3306	24.1602	NaN	24.0671	23.7547	NaN	Q9BZF1;Q9BZF1-3	Q9BZF1;Q9BZF1-3	Oxysterol-binding	OSBPL8
24.6259	24.1988	24.5662	24.4396	24.2802	24.4403	24.5963	NaN	23.9475	24.9341	24.7217	NaN	Q96SU4;Q96SU4-	Q96SU4;Q96SU4-	Oxysterol-binding	OSBPL9
26.4879	26.0446	26.1595	26.3336	26.3056	26.3951	25.3624	25.8884	25.5188	26.5199	26.6922	26.322	Q9NPF4;G3V445;(	Q9NPF4	Probable tRNA N6	OSGEP
22.6626	23.2957	23.4668	22.7978	23.4257	NaN	24.6474	NaN	NaN	23.1465	22.964	NaN	Q99650;Q99650-2	Q99650	Oncostatin-M-spe	OSMR
NaN	NaN	NaN	22.4932	NaN	NaN	NaN	NaN	NaN	NaN	NaN	NaN	Q9NRP0;A0A087V	Q9NRP0;A0A087V	Oligosaccharyltra	OSTC
25.6721	25.8134	26.0954	25.3156	25.5034	25.9566	25.9503	NaN	25.767	26.0887	25.8015	26.4019	Q92882	Q92882	Osteoclast-stimul	OSTF1
26.2136	26.7533	26.691	26.1888	26.6242	26.6701	26.8557	27.103	27.5407	26.6451	27.1777	27.5095	Q96FW1;J3KR44;F	Q96FW1;J3KR44;F	Ubiquitin thioeste	OTUB1
23.2802	NaN	23.0692	23.1808	23.2277	NaN	NaN	NaN	NaN	22.9698	22.9322	NaN	Q01804;Q01804-5	Q01804;Q01804-5	OTU domain-cont	OTUD4
NaN	NaN	NaN	23.898	NaN	NaN	NaN	NaN	NaN	NaN	NaN	NaN	Q8N6M0;A0A087	Q8N6M0;A0A087	OTU domain-cont	OTUD6B
NaN	NaN	23.0556	NaN	23.2193	NaN	NaN	NaN	NaN	23.6123	NaN	NaN	Q6GQQ9;Q6GQQ	Q6GQQ9;Q6GQQ	OTU domain-cont	OTUD7B
24.2208	23.9391	23.8901	23.848	23.8133	NaN	24.1738	NaN	NaN	24.1987	23.9977	NaN	Q96BN8;H0Y8S4;I	Q96BN8	Ubiquitin thioeste	OTULIN
NaN	NaN	NaN	NaN	NaN	NaN	NaN	18.7635	NaN	NaN	NaN	NaN	Q8WZ82	Q8WZ82	Ovarian cancer-as	OVCA2
24.7276	25.0668	24.4056	24.4854	25.0411	23.929	NaN	NaN	NaN	24.3579	24.1409	NaN	P55809;E9PDW2;I	P55809;E9PDW2	Succinyl-CoA:3-ke	OXCT1
23.0951	NaN	NaN	NaN	NaN	NaN	NaN	NaN	NaN	NaN	NaN	NaN	Q8N573-5;Q8N57	Q8N573-5;Q8N57	Oxidation resistar	OXR1
28.996	27.2238	27.6238	28.5356	27.6188	27.707	27.9284	27.6318	27.5489	28.3143	28.0511	27.8457	O95747;C9JIG9	O95747;C9JIG9	Serine/threonine-	OXSR1
23.6693	NaN	23.2981	23.1172	23.1063	23.2648	NaN	NaN	NaN	NaN	NaN	NaN	Q99571;F6SFZ6;Q	Q99571;F6SFZ6;Q	P2X purinoceptor	P2RX4
27.0711	27.7572	27.2764	27.0595	27.4751	27.0395	27.0755	28.3631	28.2178	26.8868	26.8032	27.1869	P13674	P13674	Prolyl 4-hydroxyla	P4HA1
NaN	25.1638	25.2053	24.8781	25.1634	25.0883	25.3215	NaN	NaN	NaN	NaN	NaN	P13674-2;P13674	P13674-2;P13674	Prolyl 4-hydroxyla	P4HA1
24.3335	24.8984	24.4912	24.1324	24.749	24.6865	NaN	25.5011	25.7282	23.6606	23.5945	24.2907	O15460;O15460-2	O15460;O15460-2	Prolyl 4-hydroxyla	P4HA2
30.3382	29.9303	29.9136	30.028	29.6022	29.9386	30.058	30.4031	30.2763	29.955	29.439	29.5335	P07237;H7BZ94;H	P07237;H7BZ94	Protein disulfide-i	P4HB
29.194	29.2672	28.7438	28.959	29.1891	28.6781	29.0061	29.1437	29.009	28.6824	29.1161	29.025	Q9UQ80;Q9UQ80	Q9UQ80;Q9UQ80	Proliferation-asso	PA2G4
24.8142	24.4223	24.3679	24.4483	24.5301	24.2289	23.0138	NaN	NaN	23.7837	23.9323	23.6346	Q9BRP4;F5H0C4;(	Q9BRP4;F5H0C4;(	Proteasomal ATPa	PAAF1
31.6478	30.9265	30.9652	31.3761	30.7553	31.0515	30.396	30.4752	29.8167	30.5969	30.549	30.3291	P11940;E7EQV3;A	P11940;E7EQV3;A	Polyadenylate-bir	PABPC1
28.7529	28.0364	27.9897	28.7572	28.3117	28.0053	26.7706	26.7307	26.7723	27.3008	27.5481	27.0413	Q13310;Q13310-2	Q13310;Q13310-2	Polyadenylate-bir	PABPC4
22.1974	NaN	23.8215	23.7636	23.5465	NaN	NaN	24.5127	NaN	23.1325	22.7615	24.6715	Q86U42;Q86U42-	Q86U42;Q86U42-	Polyadenylate-bir	PABPN1
23.7669	23.1803	23.5863	23.9469	23.0799	23.4704	NaN	NaN	NaN	NaN	23.1943	NaN	Q6VY07;Q6VY07-2	Q6VY07;Q6VY07-2	Phosphofurin acid	PACS1
26.6861	27.5614	27.7627	26.7884	27.483	27.9415	27.5	27.5224	27.5474	27.8276	27.8987	28.0073	Q9UNF0;Q9UNF0	Q9UNF0;Q9UNF0	Protein kinase C a	PACSIN2

25.7489	26.9988	26.9898	26.2278	26.3714	26.8942	26.4096	26.1391	26.3655	27.2887	26.9461	26.2351	Q9UKS6;A0A0C4D	Q9UKS6;A0A0C4D	Protein kinase C a	PACSIN3
NaN	NaN	NaN	NaN	NaN	NaN	23.1951	NaN	NaN	NaN	NaN	NaN	P09466;H0Y6A4;H	P09466;H0Y6A4;H	Glycodelin	PAEP
23.7097	NaN	23.2497	23.6246	23.2093	NaN	NaN	NaN	23.4064	23.4674	23.9364	23.6138	Q8N7H5;Q8N7H5	Q8N7H5;Q8N7H5	RNA polymerase I	PAF1
28.3701	28.5582	28.1562	28.097	28.3612	28.2628	28.83	28.5008	28.4527	28.3548	28.2168	28.1751	P43034;I3L495;I3I	P43034	Platelet-activating	PAFAH1B1
27.7455	28.6626	28.2091	27.8227	28.3319	28.1671	28.3386	28.1383	28.0447	28.2161	28.1186	28.2496	P68402;J3KNE3;P(	P68402;J3KNE3	Platelet-activating	PAFAH1B2
27.6348	28.2455	27.9064	27.6051	28.1883	28.1618	28.2933	28.1036	28.4675	28.3499	28.458	28.6294	Q15102;M0R389;	Q15102;M0R389;	Platelet-activating	PAFAH1B3
23.7708	24.5346	25.0968	23.9125	24.9277	25.0609	24.3004	NaN	NaN	25.8102	25.1733	25.5615	Q9NWQ8	Q9NWQ8	Phosphoprotein a	PAG1
30.5385	30.7334	30.65	30.5085	30.635	30.4755	30.358	30.5009	30.3783	30.2305	30.4023	30.3428	P22234;P22234-2	P22234;P22234-2	Multifunctional pr	PAICS
24.7511	23.6316	22.7913	24.478	23.5551	22.7605	NaN	NaN	NaN	23.2621	NaN	NaN	Q9H074-3;Q9H07	Q9H074-3;Q9H07	Polyadenylate-bir	PAIP1
NaN	NaN	21.4099	NaN	NaN	NaN	NaN	NaN	NaN	22.2976	NaN	NaN	Q13153;E9PM17;I	Q13153;E9PM17;I	Serine/threonine-	PAK1
28.6228	29.213	29.0797	28.7343	29.0649	29.0799	28.2658	28.3198	28.1273	28.4394	28.6748	28.6151	Q13177;H7C1X3;E	Q13177	Serine/threonine-	PAK2
25.0214	25.2868	26.311	25.6637	25.6207	26.2159	25.5037	22.7857	25.0823	26.7005	26.1571	25.7236	O96013;O96013-4	O96013;O96013-4	Serine/threonine-	PAK4
26.3048	26.2132	26.3042	25.6258	25.8911	25.9414	26.0354	25.1848	25.0354	26.0325	25.604	24.2942	Q8WX93;Q8WX93	Q8WX93;Q8WX93	Palladin	PALLD
NaN	NaN	NaN	NaN	NaN	NaN	NaN	NaN	23.3928	NaN	NaN	NaN	H7BYD9;H0Y9I4	H7BYD9		PAM
21.9097	23.2325	23.0093	20.5751	21.4199	22.0142	29.7821	30.0292	27.1624	23.0881	22.4375	22.3397	P19021;P19021-5	P19021;P19021-5	Peptidyl-glycine a	PAM
NaN	NaN	NaN	NaN	NaN	NaN	NaN	NaN	NaN	NaN	23.614	NaN	Q504Q3;Q504Q3-	Q504Q3;Q504Q3-	PAB-dependent p	PAN2
22.9892	NaN	23.0761	22.6411	22.5098	22.8444	NaN	NaN	NaN	NaN	NaN	NaN	Q9BZ23-4;Q9BZ2	Q9BZ23-4;Q9BZ2	Pantothenate kin	PANK2
21.3743	NaN	21.1654	20.9209	20.9213	NaN	NaN	NaN	NaN	21.2315	21.2708	NaN	Q9H999	Q9H999	Pantothenate kin	PANK3
26.3869	25.4456	25.4957	25.8744	25.3632	25.3568	24.8183	NaN	NaN	25.2164	25.6518	23.6505	Q9NVE7;A0A0G2J	Q9NVE7;A0A0G2J	Pantothenate kin	PANK4
22.9367	NaN	23.0494	23.0451	NaN	NaN	NaN	NaN	NaN	22.7607	NaN	NaN	Q96RD7;Q96RD7-	Q96RD7;Q96RD7-	Pannexin-1	PANX1
23.7595	23.122	23.5725	23.6089	NaN	23.5663	NaN	NaN	NaN	22.967	23.5493	NaN	P51003;G3XAH6;A	P51003;G3XAH6;A	Poly(A) polymeras	PAPOLA
26.3498	26.1979	25.9126	25.856	26.3308	26.1637	26.0607	26.4462	25.9669	27.2041	26.7042	25.6112	O43252	O43252	Bifunctional 3-pho	PAPSS1
29.1184	28.7071	28.525	28.5648	28.815	28.4941	28.4639	28.424	28.1919	28.9718	28.8905	28.7032	O95340;O95340-2	O95340;O95340-2	Bifunctional 3-pho	PAPSS2
23.1339	23.5173	23.2248	22.6956	23.0912	23.3768	NaN	NaN	NaN	22.9325	23.1293	23.2352	Q8TEW0;Q8TEW0	Q8TEW0;Q8TEW0	Partitioning defec	PARD3
NaN	NaN	NaN	NaN	NaN	NaN	NaN	NaN	NaN	21.1114	NaN	NaN	Q8TEW8;Q8TEW8	Q8TEW8;Q8TEW8	Partitioning defec	PARD3B
24.2173	24.5387	24.8984	24.1524	24.7626	24.7873	24.6961	NaN	24.7234	24.702	24.6786	NaN	Q9BYG5;Q9BYG4;	Q9BYG5	Partitioning defec	PARD6B
22.3623	NaN	22.1777	21.8864	NaN	NaN	NaN	NaN	NaN	NaN	NaN	NaN	Q86W56;Q86W56	Q86W56;Q86W56	Poly(ADP-ribose)	PARG
29.2	29.8893	29.3266	28.6748	29.2593	29.3278	29.4867	29.3725	29.2558	29.4441	29.1346	29.2897	Q99497;K7ELW0;I	Q99497;K7ELW0;I	Protein deglycase	PARK7
24.2716	24.3951	24.6428	24.6549	24.9905	24.6564	NaN	NaN	NaN	23.7956	24.9646	NaN	O95453;O95453-2	O95453;O95453-2	Poly(A)-specific ril	PARN
28.3982	27.6969	27.5605	28.3597	27.9416	27.5632	26.8833	25.9514	26.6531	27.2479	27.333	27.4426	P09874;Q5VX84;C	P09874	Poly [ADP-ribose]	PARP1
24.973	24.4794	25.0193	25.5496	24.4138	24.4646	24.1665	24.7686	25.4263	24.6419	23.6973	24.5323	Q9UKK3	Q9UKK3	Poly [ADP-ribose]	PARP4
NaN	NaN	NaN	22.1435	NaN	NaN	NaN	NaN	NaN	NaN	NaN	NaN	Q7L3T8	Q7L3T8	Probable proline--	PARS2
25.2642	25.5705	26.0537	25.1883	25.3955	25.5949	26.2789	25.0811	25.1752	25.8928	25.1291	25.4487	Q9NVD7;J3KNQ4;	Q9NVD7;J3KNQ4;	Alpha-parvin	PARVA
23.6609	NaN	23.9034	23.4003	23.4039	23.7411	NaN	NaN	NaN	NaN	NaN	NaN	Q86TB9;Q86TB9-4	Q86TB9;Q86TB9-4	Protein PAT1 hom	PATL1
25.7383	25.9804	25.7417	24.9833	25.1967	25.2169	25.3138	24.3744	NaN	24.4643	23.7197	NaN	Q96IZ0;A0A0B4J2	Q96IZ0	PRKC apoptosis W	PAWR
22.609	NaN	NaN	22.6658	NaN	NaN	NaN	NaN	NaN	NaN	22.4964	NaN	Q9Y5B6;Q9Y5B6-2	Q9Y5B6;Q9Y5B6-2	PAX3- and PAX7-b	PAXBP1
25.4584	26.0109	25.5567	25.0895	25.772	25.5826	26.4169	26.2819	26.0155	25.8296	26.4854	26.4925	Q9BVG4;A6NDF3	Q9BVG4;A6NDF3	Protein PBDC1	PBDC1
24.9618	24.9624	25.0757	25.1857	24.8725	25.0453	25.2106	NaN	25.0395	24.8661	24.6045	25.2927	Q96KB5;Q96KB5-1	Q96KB5;Q96KB5-1	Lymphokine-activ	PBK
22.4388	NaN	NaN	22.5211	NaN	22.2887	NaN	NaN	NaN	NaN	NaN	NaN	Q86U86;Q86U86-	Q86U86;Q86U86-	Protein polybrom	PBRM1
27.7013	27.103	27.0342	27.9298	27.2096	27.0665	26.0665	27.0576	27.7365	27.035	27.2985	26.7739	P11498;P11498-2	P11498	Pyruvate carboxyl	PC
25.258	26.1926	25.596	25.0358	26.0021	25.4942	25.8575	25.6901	26.5028	26.0401	26.2132	25.4761	P61457	P61457	Pterin-4-alpha-car	PCBD1
30.4395	29.905	30.1637	30.3509	29.955	30.2604	29.6374	29.6349	29.5209	30.1819	30.2543	30.3091	Q15365	Q15365	Poly(rC)-binding p	PCBP1
29.0289	27.8064	28.1479	28.9031	28.123	28.1463	27.5461	26.9859	27.631	27.8113	27.3538	26.99	Q15366-3;Q15366	Q15366-3;Q15366	Poly(rC)-binding p	PCBP2
NaN	NaN	NaN	22.7075	NaN	NaN	NaN	NaN	NaN	NaN	NaN	NaN	Q15366-4;Q15366	Q15366-4;Q15366	Poly(rC)-binding p	PCBP2
NaN	NaN	NaN	22.6327	NaN	NaN	NaN	NaN	NaN	NaN	NaN	NaN	F8VZX2;H3BRU6;F	F8VZX2;H3BRU6;F	Poly(rC)-binding p	PCBP2;PCBP3
NaN	NaN	NaN	NaN	NaN	NaN	NaN	25.6716	NaN	NaN	NaN	NaN	Q9Y5H1;Q9Y5H1-	Q9Y5H1;Q9Y5H1-	Protocadherin gar	PCDHGA2
23.9209	23.6032	23.5937	24.317	23.2734	23.6234	23.6178	NaN	NaN	24.0097	NaN	NaN	Q5JVF3;Q5JVF3-3	Q5JVF3;Q5JVF3-3	PCI domain-conta	PCID2
21.9097	NaN	NaN	22.4439	NaN	NaN	NaN	NaN	NaN	NaN	NaN	NaN	Q9H4Z3;A0A087V	Q9H4Z3	Phosphorylated C	PCIF1
NaN	NaN	NaN	NaN	NaN	NaN	NaN	NaN	NaN	NaN	20.6079	NaN	H0YM31;A0A0A0I	H0YM31;A0A0A0I	Phosphoenolpyru	PCK2
27.1657	26.1054	25.9998	26.4499	26.1982	25.9763	24.5806	25.596	26.3873	26.7432	27.3069	25.631	Q16822;B4DW73;	Q16822;B4DW73;	Phosphoenolpyru	PCK2
27.4026	27.0978	27.4835	26.9415	26.9239	26.7778	27.4513	27.284	26.4033	26.4895	26.1836	25.949	E7ETA6	E7ETA6		PCM1
27.6225	28.3385	27.6946	27.4913	28.1313	27.9661	28.4554	28.3304	28.5354	28.1385	27.8379	28.6509	P22061;F6S8N6;A	P22061;F6S8N6;A	Protein-L-isoaspa	PCMT1
29.0751	29.1693	28.9168	28.953	29.2755	29.0599	28.8954	29.0202	29.2159	29.2984	29.7539	30.0201	P12004	P12004	Proliferating cell r	PCNA
25.6632	26.9897	26.2679	25.9467	26.9533	26.4533	26.4189	27.2364	26.5715	26.2252	26.9668	27.8497	Q8WW12;Q8WW	Q8WW12;Q8WW	PEST proteolytic s	PCNP
NaN	NaN	NaN	NaN	NaN	NaN	24.7454	NaN	NaN	NaN	NaN	NaN	Q9UHG2	Q9UHG2	ProSAAS;KEP;Big S	PCSK1N
NaN	NaN	23.2644	NaN	NaN	NaN	27.5834	26.2793	NaN	23.8273	NaN	NaN	Q8NBP7;Q8NBP7-	Q8NBP7	Proprotein conver	PCSK9
26.9869	27.6176	27.3107	27.3094	27.3373	27.3471	27.0201	27.2325	27.7572	26.8743	26.736	26.6214	Q9UHG3;Q9UHG3	Q9UHG3;Q9UHG3	Prenylcysteine ox	PCYOX1
22.6128	23.5762	23.2871	23.2338	22.8775	NaN	NaN	NaN	NaN	23.0128	NaN	NaN	P49585;C9J050;C9	P49585;C9J050;C9	Choline-phosphat	PCYT1A
26.0245	25.4399	25.4414	25.2115	25.6462	24.3772	24.45	24.6197	23.9366	26.0578	25.7268	NaN	Q99447;Q99447-3	Q99447;Q99447-3	Ethanolamine-pho	PCYT2
24.8545	25.1048	25.259	24.458	24.5904	25.079	24.0255	NaN	25.3211	24.9886	25.1661	25.6584	Q13442;F8WBW6	Q13442	28 kDa heat- and	PDAP1

26.1248	27.1941	27.1716	26.403	27.2983	27.0969	26.6531	26.436	27.0117	26.5434	26.8458	26.9184	Q9BUL8;C9J5C3;C	Q9BUL8;C9J5C3;C	Programmed cell	PDCD10
25.4945	23.0237	23.7942	25.7026	23.8119	24.2195	NaN	NaN	NaN	NaN	22.7856	NaN	Q14690	Q14690	Protein RRP5 hom	PDCD11
24.3266	23.1557	23.5001	23.8383	23.2638	23.6731	22.7546	NaN	NaN	23.1647	22.929	NaN	Q9BRP1	Q9BRP1	Programmed cell	PDCD2L
26.6959	24.0898	24.7753	25.0649	23.9283	23.5623	24.7819	23.5539	23.4706	23.3927	23.9158	22.6928	Q53EL6;Q53EL6-2	Q53EL6;Q53EL6-2	Programmed cell	PDCD4
27.0167	27.4336	27.1203	26.6865	27.3815	27.1819	27.1327	26.9576	27.045	26.8548	27.4426	27.4278	O14737;K7EQA1;(	O14737;K7EQA1;(	Programmed cell	PDCD5
28.023	27.2772	27.0422	27.2829	26.2225	26.6134	27.1799	25.8139	25.6462	26.5719	26.1591	25.539	O75340;O75340-2	O75340;O75340-2	Programmed cell	PDCD6
31.3616	31.5827	31.4099	31.1875	31.0135	31.4143	32.542	32.0077	32.5268	31.8395	31.708	32.3673	Q8WUM4;Q8WUI	Q8WUM4;Q8WUI	Programmed cell	PDCD6IP
22.8581	NaN	NaN	NaN	NaN	NaN	NaN	NaN	NaN	NaN	NaN	NaN	Q9H2J4;H7BZP2	Q9H2J4	Phosducin-like pro	PDCL3
26.3153	25.4525	25.6271	26.3407	25.4616	25.3912	26.0743	25.7695	25.4242	26.2728	26.0789	25.8872	Q6L8Q7;Q6L8Q7-	Q6L8Q7;Q6L8Q7-	2,5-phosphodieste	PDE12
23.4367	24.0297	25.0494	23.6931	24.2589	25.2959	NaN	NaN	NaN	24.1694	24.4691	24.3699	O60658;O60658-2	O60658;O60658-2	High affinity cAMI	PDE8A
23.1846	NaN	22.9619	NaN	NaN	NaN	26.3055	25.0869	24.6919	24.2808	23.6854	NaN	Q9NRA1;Q9NRA1	Q9NRA1;Q9NRA1	Platelet-derived g	PDGFC
NaN	25.7465	26.0406	NaN	25.8014	26.0136	NaN	NaN	NaN	25.6828	NaN	NaN	P09619	P09619	Platelet-derived g	PDGFRB
27.5592	27.4775	27.1077	27.8425	27.9727	27.3075	26.4819	27.787	28.2852	27.7707	28.4391	27.3777	P08559;P08559-2	P08559;P08559-2	Pyruvate dehydro	PDHA1
27.135	26.1792	26.1404	27.2416	26.6009	26.4014	25.1536	26.5819	26.8875	25.7969	25.8882	25.8533	P11177;P11177-2	P11177;P11177-2	Pyruvate dehydro	PDHB
NaN	NaN	NaN	NaN	NaN	22.0405	NaN	NaN	NaN	NaN	NaN	NaN	O00330;O00330-3	O00330;O00330-3	Pyruvate dehydro	PDHX
NaN	NaN	27.1601	NaN	NaN	NaN	NaN	NaN	NaN	NaN	NaN	NaN	H7BZJ3	H7BZJ3		PDIA3
31.1577	32.1604	31.5321	31.1506	31.9994	31.6566	31.7164	32.5416	32.5503	31.3272	31.5289	31.8394	P30101	P30101	Protein disulfide-i	PDIA3
29.856	29.8448	30.0652	29.7902	29.8748	30.1806	29.8046	30.7029	30.7453	29.7278	29.7838	29.8293	P13667;C9JMN9	P13667	Protein disulfide-i	PDIA4
NaN	NaN	NaN	21.1782	NaN	NaN	NaN	NaN	22.3584	21.6695	21.6385	NaN	Q14554;Q14554-2	Q14554;Q14554-2	Protein disulfide-i	PDIA5
30.1509	30.1523	30.0428	30.0132	30.1817	30.0448	30.1121	30.5046	30.3523	30.1242	29.9955	29.5405	Q15084;Q15084-5	Q15084;Q15084-5	Protein disulfide-i	PDIA6
NaN	NaN	NaN	24.6224	NaN	NaN	NaN	NaN	NaN	NaN	NaN	NaN	Q15118;Q15118-2	Q15118;Q15118-2	[Pyruvate dehydro	PDK1
23.804	23.5169	23.6824	23.8587	23.5873	23.4908	NaN	NaN	23.6241	23.0557	23.6109	NaN	Q15120;Q15120-2	Q15120;Q15120-2	[Pyruvate dehydro	PDK3
27.2528	28.2792	27.8754	27.1617	28.1551	28.0026	27.0338	27.2769	27.3756	26.963	27.2153	27.6843	O00151	O00151	PDZ and LIM dom	PDLIM1
23.6086	23.4349	NaN	NaN	23.1986	NaN	NaN	NaN	NaN	NaN	NaN	NaN	Q96JY6;Q96JY6-5	Q96JY6;Q96JY6-5	PDZ and LIM dom	PDLIM2
22.6325	23.6344	22.8919	NaN	23.6656	23.5599	NaN	23.9029	23.5272	NaN	NaN	NaN	Q53GG5;Q53GG5	Q53GG5;Q53GG5	PDZ and LIM dom	PDLIM3
24.851	25.6086	25.1104	24.531	25.5015	25.3346	25.0755	25.3694	24.9105	25.803	25.3319	25.5092	P50479;P50479-2	P50479;P50479-2	PDZ and LIM dom	PDLIM4
28.6878	28.4596	28.381	28.2495	28.0241	28.2772	28.748	27.2603	27.5654	28.3392	27.9477	27.6583	Q96HC4;Q96HC4-	Q96HC4;Q96HC4-	PDZ and LIM dom	PDLIM5
26.8667	27.7197	27.7847	27.341	27.9225	28.2051	27.4338	26.9543	27.4356	28.0385	28.1026	28.4023	Q9NR12;Q9NR12-	Q9NR12;Q9NR12-	PDZ and LIM dom	PDLIM7
23.7319	23.1592	23.3884	23.6632	23.9662	23.5753	NaN	NaN	NaN	23.1423	NaN	NaN	Q9P0J1;Q9P0J1-2	Q9P0J1;Q9P0J1-2	[Pyruvate dehydro	PDP1
23.7316	23.1305	23.0899	23.2718	22.5212	NaN	NaN	NaN	NaN	23.3914	23.1452	NaN	O15530;E9PER6;C	O15530;E9PER6;C	3-phosphoinositid	PDPK1;PDPK2P
22.3351	22.3824	22.2779	22.0594	21.8403	NaN	NaN	22.4855	22.5301	22.9607	22.826	22.7873	Q8NCN5;A8MT40	Q8NCN5;A8MT40	Pyruvate dehydro	PDPR
27.4007	25.7976	26.4411	27.6676	26.2346	26.5053	23.3058	24.2807	24.4835	26.4156	26.6116	25.8873	Q29RF7;Q29RF7-3	Q29RF7	Sister chromatid c	PDS5A
27.2962	25.5301	26.7713	27.4965	25.9942	26.5795	24.1984	23.9066	NaN	27.0944	26.0401	24.1305	Q9NTI5;Q9NTI5-2	Q9NTI5;Q9NTI5-2	Sister chromatid c	PDS5B
24.8239	24.1593	24.8735	24.0047	23.6452	24.1533	24.6488	NaN	NaN	23.8691	NaN	NaN	Q6P996;Q6P996-4	Q6P996;Q6P996-4	Pyridoxal-depend	PDXDC1
26.7434	26.7037	26.2792	26.1347	26.2248	26.2158	26.5374	26.2902	26.1495	26.7148	26.4384	26.1983	O00764;F2Z2Y4;O	O00764;F2Z2Y4;O	Pyridoxal kinase	PDXK
22.5808	23.7391	23.1726	NaN	23.6185	NaN	NaN	NaN	NaN	NaN	22.9577	NaN	Q96GD0	Q96GD0	Pyridoxal phospha	PDXP
22.2737	21.8061	22.8357	22.2156	NaN	NaN	NaN	NaN	NaN	22.0705	NaN	NaN	Q5EBL8;Q5EBL8-2	Q5EBL8;Q5EBL8-2	PDZ domain-conta	PDZD11
23.9035	25.1359	24.9087	24.1153	24.3597	24.3244	25.5076	24.5864	24.5619	24.6152	NaN	24.447	Q15121;B1AKZ5;C	Q15121;B1AKZ5;C	Astrocytic phosph	PEA15
22.0435	22.4433	22.6865	22.3407	NaN	NaN	22.8315	NaN	NaN	22.5236	NaN	NaN	Q9H792;H3BUZ5;	Q9H792	Pseudopodium-en	PEAK1
29.0732	29.3957	28.8058	28.4592	28.8154	28.9186	29.9661	29.4014	28.6795	29.612	29.3132	29.0077	P30086	P30086	Phosphatidyletha	PEBP1
27.0378	26.8949	27.1299	26.8785	26.718	26.5009	27.0486	26.1548	26.1648	26.9362	26.7163	26.9641	Q9UBV8	Q9UBV8	Peflin	PEF1
26.242	25.9751	25.3292	26.2439	26.3519	25.1314	25.6618	26.9873	25.6128	25.1585	25.4892	24.7955	Q86TG7;A0A087V	Q86TG7;A0A087V	Retrotransposon-	PEG10
27.7988	26.8098	27.3895	27.8212	27.0525	27.3389	26.7166	27.7372	27.3731	27.1337	27.4988	27.362	Q9BRX2	Q9BRX2	Protein pelota ho	PELO
24.6571	23.5463	24.5477	25.3981	24.6026	24.5478	NaN	NaN	NaN	23.7426	23.9233	NaN	Q8IZL8;C9JFV4;E7	Q8IZL8;C9JFV4;E7	Proline-, glutamic	PELP1
25.6493	25.8805	25.1693	25.3334	25.5294	25.1308	26.5168	26.669	26.1116	25.8179	25.5686	25.5827	P12955;P12955-3	P12955;P12955-3	Xaa-Pro dipeptida	PEPD
26.9913	25.2892	26.1947	27.3498	26.3116	26.6068	24.8852	NaN	24.686	26.0478	26.2343	25.5169	O00541;B5MCF9;	O00541;B5MCF9;	Pescadillo homolo	PES1
NaN	NaN	NaN	NaN	NaN	NaN	NaN	NaN	NaN	NaN	20.5368	NaN	Q92968	Q92968	Peroxisomal mem	PEX13
22.019	NaN	21.7869	22.2807	NaN	NaN	NaN	NaN	NaN	NaN	NaN	NaN	O75381;O75381-2	O75381;O75381-2	Peroxisomal mem	PEX14
NaN	NaN	NaN	NaN	NaN	NaN	NaN	NaN	NaN	20.5482	NaN	NaN	P40855;Q5QNY5;	P40855;Q5QNY5;	Peroxisomal bioge	PEX19
22.0804	NaN	21.7665	NaN	NaN	NaN	NaN	NaN	NaN	NaN	NaN	NaN	P56589;Q7Z6V3	P56589;Q7Z6V3	Peroxisomal bioge	PEX3
23.6772	23.9276	23.8247	NaN	NaN	NaN	NaN	NaN	NaN	22.8965	24.0849	24.434	P50542;P50542-4	P50542;P50542-4	Peroxisomal targe	PEX5
27.8713	28.2121	28.1232	27.7787	28.3558	28.1071	28.3666	28.7448	28.5881	28.2153	28.5582	29.0132	O15067;H0YGH1;	O15067	Phosphoribosylfor	PFAS
26.7384	28.1477	27.5725	26.573	28.029	27.4237	27.9094	28.4657	27.5974	27.2783	27.6916	27.9029	O60925;E5RGS4;D	O60925;E5RGS4	Prefoldin subunit	PFDN1
28.006	29.1107	28.5791	27.7872	28.8415	28.4273	28.7373	28.9515	27.9989	28.2681	28.5753	28.4419	Q9UHV9	Q9UHV9	Prefoldin subunit	PFDN2
25.7936	26.2353	25.494	24.899	25.5721	25.5358	26.0266	25.9797	24.9151	25.5837	25.5628	25.9732	Q9NQP4;E9PQY2	Q9NQP4;E9PQY2	Prefoldin subunit	PFDN4
27.0903	27.9559	27.5321	26.6756	27.5549	27.4341	27.6216	27.4121	26.9487	27.3291	27.4305	27.395	Q99471;H3BPF6;(	Q99471;H3BPF6;(	Prefoldin subunit	PFDN5
26.6413	28.2022	27.5818	26.6732	27.9108	27.5967	27.8391	27.995	27.2311	27.4196	27.8991	27.7461	O15212;A2AB88	O15212	Prefoldin subunit	PFDN6
22.337	NaN	NaN	21.6935	NaN	NaN	NaN	NaN	NaN	NaN	NaN	NaN	O60825;O60825-2	O60825;O60825-2	6-phosphofructo-	PFKFB2
22.6258	NaN	22.83	23.0431	22.2438	22.1553	NaN	NaN	NaN	NaN	NaN	NaN	Q16875;F2Z2I2;Q	Q16875;F2Z2I2;Q	6-phosphofructo-	PFKFB3
22.7771	NaN	22.4819	23.0542	NaN	NaN	NaN	NaN	NaN	NaN	23.0027	NaN	Q16877;C9K0D8;(	Q16877;C9K0D8;(	6-phosphofructo-	PFKFB4

29.3214	28.4077	29.0911	29.3493	28.6857	29.3303	28.3112	28.7594	28.5145	28.9785	29.031	28.6908	P17858;P17858-2	P17858;P17858-2	ATP-dependent 6-	PFKL
27.5662	26.5861	27.2204	27.2956	27.0051	27.3515	26.9785	27.1897	27.4237	27.69	27.9138	27.5757	P08237;P08237-3	P08237;P08237-3	ATP-dependent 6-	PFKM
31.6731	30.7758	31.3817	31.5671	31.1037	31.4934	30.6663	30.8998	30.9319	31.3744	31.3683	30.8311	Q01813;Q01813-2	Q01813;Q01813-2	ATP-dependent 6-	PFKP
32.3813	33.1146	32.4393	32.0513	32.5799	32.446	33.1636	32.4578	32.0836	32.7162	32.5829	32.4413	P07737;K7EJ44;I3	P07737;K7EJ44	Profilin-1	PFN1
26.8135	27.5678	27.3405	26.6866	27.4826	27.4028	26.9929	26.8179	27.0165	26.6831	27.3335	27.1129	P35080-2;C9J0J7;(	P35080-2;C9J0J7;(	Profilin-2;Profilin	PFN2
31.4835	32.3876	32.0882	31.553	32.2873	32.1195	32.3946	32.2669	32.2545	32.3416	32.4503	32.5206	P18669;Q8N0Y7;F	P18669;Q8N0Y7	Phosphoglycerate	PGAM1;PGAM4
27.8626	27.6113	26.9642	27.5744	27.0939	26.7836	25.7838	26.8891	26.9572	27.1726	26.8234	25.8802	Q96HS1;Q96HS1-:	Q96HS1;Q96HS1-:	Serine/threonine-	PGAM5
28.5524	29.0201	28.986	28.4788	28.8294	28.7483	29.3113	28.5715	28.5008	29.0778	28.813	29.0586	P52209;P52209-2	P52209;P52209-2	6-phosphoglucona	PGD
23.3789	NaN	NaN	22.7027	NaN	NaN	NaN	NaN	NaN	NaN	22.8363	NaN	P53609;P53609-2	P53609;P53609-2	Geranylgeranyl tr	PGGT1B
32.2941	32.9672	32.6737	31.9911	32.5132	32.5648	32.8255	32.4162	32.4674	32.5086	32.5996	32.815	P00558;P00558-2	P00558;P00558-2	Phosphoglycerate	PGK1
26.7353	27.5702	27.3517	26.6581	27.4405	27.413	27.0598	27.1778	27.2636	26.9508	27.3777	27.5518	O95336;M0R261;	O95336;M0R261;	6-phosphogluconi	PGLS
28.3635	28.5765	28.6756	27.9889	28.3027	28.7143	28.9315	27.7981	28.2893	28.5289	28.5057	28.4691	P36871;P36871-2	P36871;P36871-2	Phosphoglucomut	PGM1
27.7678	28.4457	28.0216	27.5549	28.5568	28.0817	28.1873	27.4301	28.2449	28.0616	28.2811	28.3831	Q96G03;E7ENQ8;	Q96G03	Phosphoglucomut	PGM2
26.0217	26.7069	26.3403	25.6529	26.5966	26.4617	26.2758	26.2119	26.4429	25.904	26.2134	26.3545	O95394;O95394-3	O95394;O95394-3	Phosphoacetylglu	PGM3
24.2692	25.049	24.6748	24.3149	24.9369	24.4253	25.5441	25.814	26.6216	25.4235	25.6346	25.6384	A6NDG6;H3BV17	A6NDG6	Phosphoglycolate	PGP
NaN	NaN	NaN	NaN	23.431	NaN	NaN	NaN	NaN	NaN	NaN	NaN	Q9NXJ5;U3KQ24;I	Q9NXJ5;U3KQ24;I	Pyroglutamyl-pep	PGPEP1
26.433	26.0773	26.5898	26.3911	26.3452	26.7287	27.1766	27.249	27.3058	27.538	27.0665	26.7511	O00264;O00264-2	O00264;O00264-2	Membrane-associ	PGRMC1
25.5687	24.9409	25.2096	25.5673	24.8564	25.0856	25.1911	25.426	24.8415	25.1255	24.9063	24.6532	O15173;O15173-2	O15173;O15173-2	Membrane-associ	PGRMC2
NaN	NaN	24.1983	22.8058	NaN	NaN	NaN	NaN	NaN	NaN	NaN	NaN	J3KP75;O75167;O	J3KP75;O75167;O	Phosphatase and	PHACTR2
22.8573	NaN	23.461	NaN	NaN	22.9987	NaN	NaN	23.3052	24.7109	23.814	23.7514	Q8IZ21;Q8IZ21-3;	Q8IZ21;Q8IZ21-3;	Phosphatase and	PHACTR4
23.0141	NaN	23.0704	23.5863	22.6813	23.6961	NaN	NaN	NaN	22.9952	NaN	NaN	Q9H814	Q9H814	Phosphorylated a	PHAX
28.1235	29.4781	28.6436	28.8327	29.6497	28.9228	27.3841	29.7108	30.5695	27.2679	28.4315	29.2021	P35232;C9JW96;E	P35232;C9JW96;E	Prohibitin	PHB
28.2368	29.166	28.9237	28.908	29.5163	29.0806	27.5113	29.1072	30.1192	27.2319	28.2105	28.8656	Q99623;F5GY37;J	Q99623;F5GY37;J	Prohibitin-2	PHB2
23.162	NaN	22.8228	23.3266	NaN	NaN	NaN	NaN	NaN	22.9175	NaN	NaN	Q8IXK0;B3KPJ4;A(	Q8IXK0;B3KPJ4;A(	Polyhomeotic-like	PHC2
21.9061	NaN	NaN	22.0023	21.8017	NaN	NaN	NaN	NaN	NaN	NaN	NaN	O43189;O43189-2	O43189;O43189-2	PHD finger protei	PHF1
NaN	NaN	21.5164	22.7934	21.544	NaN	NaN	NaN	NaN	NaN	NaN	NaN	O94880;O94880-2	O94880;O94880-2	PHD finger protei	PHF14
24.4151	24.4463	24.7445	24.0316	23.9488	NaN	NaN	NaN	NaN	24.0268	NaN	NaN	Q7RTV0	Q7RTV0	PHD finger-like dc	PHF5A
24.6681	24.5078	25.3507	25.7977	25.5026	25.4318	23.5799	NaN	NaN	25.5545	25.5215	25.0004	Q8IWS0;Q8IWS0-	Q8IWS0;Q8IWS0-	PHD finger protei	PHF6
28.2946	27.8738	28.0228	27.9658	28.0368	28.0944	28.2619	28.5126	28.2716	28.5915	28.8361	28.4007	O43175;Q5SZU1	O43175;Q5SZU1	D-3-phosphoglyce	PHGDH
23.2887	NaN	NaN	23.1158	NaN	22.7067	NaN	NaN	NaN	23.0848	NaN	NaN	P46019	P46019	Phosphorylase b k	PHKA2
23.6701	NaN	23.4248	23.9276	23.9065	23.928	23.1893	NaN	NaN	23.8018	23.5974	NaN	Q93100-4;Q9310(	Q93100-4;Q9310(	Phosphorylase b k	PHKB
24.7886	24.2761	24.1275	24.8569	NaN	24.3083	24.4396	NaN	NaN	NaN	NaN	NaN	P15735;J3KNN3;P	P15735;J3KNN3;P	Phosphorylase b k	PHKG2
27.2209	27.4389	27.873	27.4154	27.52	28.2136	27.4387	26.7979	27.0401	28.3465	27.9771	27.4853	Q8WV24;R4GND3	Q8WV24;R4GND3	Pleckstrin homolo	PHLDA1
25.2447	26.3173	26.2664	25.436	26.1738	26.0862	25.8668	26.002	26.3244	26.6583	26.1586	26.2507	Q53GA4	Q53GA4	Pleckstrin homolo	PHLDA2
21.4512	NaN	NaN	NaN	NaN	NaN	NaN	NaN	NaN	NaN	NaN	NaN	Q86UU1;Q86UU1	Q86UU1;Q86UU1	Pleckstrin homolo	PHLDB1
23.3786	NaN	22.9215	NaN	NaN	NaN	NaN	NaN	NaN	NaN	NaN	NaN	Q86SQ0;Q86SQ0-	Q86SQ0;Q86SQ0-	Pleckstrin homolo	PHLDB2
25.5517	26.1643	26.0188	25.1937	26.0285	26.1717	25.5188	26.5227	NaN	25.7827	26.2684	26.2982	Q9NRX4;Q9NRX4-	Q9NRX4	14 kDa phosphohi	PHPT1
NaN	NaN	NaN	21.7418	NaN	NaN	NaN	NaN	NaN	NaN	NaN	NaN	Q5SRE7;Q5SRE7-2	Q5SRE7;Q5SRE7-2	Phytanoyl-CoA dic	PHYHD1
28.0388	27.856	27.9958	28.0343	27.5576	27.9783	27.5739	27.3846	28.0756	27.7165	27.2268	27.7889	Q9BTU6;E9PAM4	Q9BTU6;E9PAM4	Phosphatidylinosi	PI4K2A
23.0388	NaN	22.6542	22.9769	22.5771	23.1191	NaN	NaN	NaN	22.728	22.5899	NaN	Q8TCG2;G5E9Z4	Q8TCG2;G5E9Z4	Phosphatidylinosi	PI4K2B
26.6934	26.3773	27.5788	27.0535	26.6002	27.75	26.5684	25.8265	25.6493	27.9972	27.102	27.0953	P42356;J3KN10;P	P42356;J3KN10	Phosphatidylinosi	PI4KA
23.058	22.998	NaN	NaN	NaN	NaN	NaN	NaN	NaN	NaN	NaN	NaN	Q8WXW3;A0A087	Q8WXW3;A0A087	Progesterone-ind	PIBF1
24.91	25.4362	25.7227	24.71	25.6292	25.8135	NaN	NaN	NaN	24.8193	24.3851	NaN	Q13492;Q13492-3	Q13492;Q13492-3	Phosphatidylinosi	PICALM
NaN	NaN	NaN	NaN	NaN	NaN	NaN	NaN	NaN	NaN	NaN	21.4029	Q92508;H0YB49;E	Q92508	Piezo-type mecha	PIEZO1
NaN	NaN	NaN	NaN	NaN	NaN	23.6357	NaN	25.31	NaN	NaN	25.1404	Q92643;A6NEM5;	Q92643;A6NEM5;	GPI-anchor transa	PIGK
24.003	22.9965	23.8439	23.8939	23.383	23.5918	23.2414	NaN	24.5696	23.357	23.4588	23.654	Q96S52;Q96S52-2	Q96S52;Q96S52-2	GPI transamidase	PIGS
23.2368	22.7842	22.9258	22.789	22.802	23.0644	22.9644	NaN	NaN	NaN	NaN	22.6158	Q969N2;Q969N2-	Q969N2;Q969N2-	GPI transamidase	PIGT
23.9538	24.2598	23.8169	23.6211	24.0634	NaN	NaN	NaN	NaN	23.4806	24.2807	NaN	Q9NWS0;M0QYA:	Q9NWS0;M0QYA:	PIH1 domain-cont	PIH1D1
25.2538	23.4322	24.3008	25.3003	24.0376	24.069	NaN	NaN	NaN	22.3935	23.6691	NaN	O00443;A0A0C4D	O00443	Phosphatidylinosi	PIK3C2A
26.1873	25.4125	25.8923	25.6486	25.3469	25.2672	25.5263	25.9431	24.1923	25.6854	25.3226	24.7278	Q8NEB9;A8MYT4;	Q8NEB9;A8MYT4	Phosphatidylinosi	PIK3C3
NaN	NaN	NaN	21.2482	NaN	NaN	NaN	NaN	NaN	NaN	NaN	NaN	P42338	P42338	Phosphatidylinosi	PIK3CB
22.2028	22.9332	22.6649	22.5692	23.3019	22.7028	21.9314	NaN	NaN	21.9689	NaN	NaN	O00459;E9PFP1;V	O00459;E9PFP1	Phosphatidylinosi	PIK3R2
25.9362	24.4674	25.0687	25.2572	24.3438	24.7825	24.4878	24.5029	23.7911	25.0291	25.401	22.9827	Q99570;D6RJ98;D	Q99570	Phosphoinositide	PIK3R4
25.2951	25.6103	25.5579	24.8578	25.2224	25.4538	24.8824	24.9656	NaN	24.9603	25.3726	25.1981	Q13526;K7EN45;k	Q13526;K7EN45;k	Peptidyl-prolyl cis	PIN1
24.389	25.5033	25.3275	NaN	25.5261	25.1412	25.5582	26.389	25.6598	23.5035	25.4479	25.9339	Q9Y237;Q9Y237-2	Q9Y237;Q9Y237-2	Peptidyl-prolyl cis	PIN4
NaN	21.7449	NaN	NaN	NaN	NaN	NaN	NaN	23.5574	NaN	21.9702	23.1876	P12273	P12273	Prolactin-inducibl	PIP
25.3513	25.3685	25.5879	25.1552	25.2608	26.0332	25.6835	25.3687	25.383	26.1645	25.8882	26.1362	P48426;P48426-2	P48426;P48426-2	Phosphatidylinosi	PIP4K2A
23.5893	23.7433	24.33	24.1551	23.8691	24.3576	23.9569	NaN	24.2052	24.638	24.3299	24.4991	P78356;P78356-2	P78356;P78356-2	Phosphatidylinosi	PIP4K2B
24.1777	23.929	24.1672	23.9802	24.0108	23.9757	23.9974	NaN	NaN	24.3242	23.6412	NaN	Q8TBX8;Q8TBX8-:	Q8TBX8;Q8TBX8-:	Phosphatidylinosi	PIP4K2C
26.2068	26.0417	26.2274	26.0212	26.072	26.6402	26.052	25.8424	25.9714	26.8983	26.7322	26.9817	Q99755-2;A6PW5	Q99755-2;A6PW5	Phosphatidylinosi	PIP5K1A

NaN	NaN	22.7554	NaN	NaN	NaN	NaN	NaN	NaN	NaN	NaN	NaN	O60331;O60331-4	O60331;O60331-4	Phosphatidylinosi	PIP5K1C
22.3623	23.863	23.059	22.1352	23.1712	23.2524	NaN	NaN	NaN	NaN	NaN	NaN	O00625	O00625	Pirin	PIR
24.3761	24.4502	24.4028	24.4579	24.6338	24.8345	NaN	NaN	NaN	23.7477	NaN	24.9435	Q9GZP4;Q9GZP4-	Q9GZP4;Q9GZP4-	PITH domain-cont	PITHD1
25.1546	26.5945	25.9733	25.2938	26.3331	26.1309	26.261	25.9379	26.4784	26.4628	26.498	26.1516	Q00169;F5GWE5;	Q00169;F5GWE5;	Phosphatidylinosi	PITPNA
26.8065	27.6809	27.5553	26.8433	27.8001	27.6497	27.3674	27.2105	27.4614	27.8745	27.814	27.7255	P48739;A0A0A0M	P48739;A0A0A0M	Phosphatidylinosi	PITPNB
23.8258	24.9515	23.8553	23.6636	24.3732	23.8516	23.0522	NaN	24.6896	24.0596	23.9671	24.3367	Q5JRX3;Q5JRX3-2	Q5JRX3;Q5JRX3-2	Presequence prot	PITRM1
33.6017	33.6877	33.6376	33.26	33.7124	33.6627	33.9815	34.0915	33.8837	33.9506	34.2565	34.0398	P14618;P14618-3,	P14618;P14618-3,	Pyruvate kinase P	PKM
24.6116	25.8511	25.132	24.3551	25.8376	25.3908	26.5317	26.9877	27.0476	26.1609	26.3784	26.946	P14618-2;H3BTN5	P14618-2;H3BTN5	Pyruvate kinase P	PKM
25.3948	25.2738	25.6876	25.3822	25.5789	25.7707	25.7493	25.4007	25.8934	25.4467	25.398	25.7662	Q16512;Q16512-2	Q16512;Q16512-2	Serine/threonine-	PKN1
26.6562	26.1851	26.9331	26.7502	26.6695	27.0867	26.5114	26.5163	27.0127	26.6693	26.5062	26.8458	Q16513;Q16513-2	Q16513;Q16513-2	Serine/threonine-	PKN2
NaN	NaN	23.2661	NaN	NaN	NaN	NaN	NaN	NaN	22.9753	22.8657	23.3119	Q6P5Z2	Q6P5Z2	Serine/threonine-	PKN3
NaN	NaN	NaN	NaN	NaN	NaN	26.3849	NaN	NaN	NaN	NaN	NaN	Q13835;Q13835-2	Q13835;Q13835-2	Plakophilin-1	PKP1
27.7056	27.8406	28.5223	27.8616	27.6585	28.4729	27.64	26.9755	27.4734	28.6969	28.0645	27.9943	Q99959-2;Q99959	Q99959-2;Q99959	Plakophilin-2	PKP2
23.6899	24.3903	24.4968	23.6574	23.9522	24.8916	23.9992	NaN	NaN	23.9261	NaN	24.3795	Q9Y446;Q9Y446-2	Q9Y446;Q9Y446-2	Plakophilin-3	PKP3
22.1674	NaN	22.6425	22.3497	NaN	22.5737	NaN	NaN	NaN	22.5012	NaN	NaN	Q99569;Q99569-2	Q99569;Q99569-2	Plakophilin-4	PKP4
26.2045	25.3785	25.422	25.3888	25.3473	25.363	25.0447	25.0161	25.3804	25.4144	25.3458	25.2193	Q9Y263;E5RIM3;H	Q9Y263;E5RIM3	Phospholipase A-2	PLAA
30.519	29.3319	30.3496	29.0769	28.2987	29.2599	30.2207	29.8767	29.3667	27.5824	26.6216	26.5084	P00750;B4DNJ1;B	P00750;B4DNJ1;B	Tissue-type plasm	PLAT
26.2384	26.9744	27.0915	26.0514	26.6615	26.9943	27.2516	NaN	26.1243	26.9838	26.4724	25.9579	P00749;E7ET40;P(	P00749;E7ET40;P(	Urokinase-type pl	PLAU
26.4901	26.2031	25.1314	26.1129	25.4531	25.9954	27.1418	NaN	26.7619	26.642	26.1067	26.2111	Q03405;M0R1I2;(	Q03405;M0R1I2;(	Urokinase plasmir	PLAUR
NaN	NaN	NaN	NaN	NaN	NaN	NaN	NaN	NaN	22.6802	22.1243	NaN	Q6P4A8	Q6P4A8	Phospholipase B-l	PLBD1
23.8268	24.1642	23.6603	23.9677	23.0703	23.8401	25.335	24.9216	NaN	23.4941	23.0221	NaN	Q8NHP8;Q8NHP8	Q8NHP8;Q8NHP8	Putative phospho	PLBD2
26.5479	27.0289	27.3223	26.3687	26.8218	27.2818	27.4121	27.0551	27.1746	27.1319	27.4101	27.5823	Q01970;Q01970-2	Q01970;Q01970-2	1-phosphatidylinc	PLCB3
24.2423	24.8716	25.4246	24.3853	24.8148	25.4249	25.0945	24.1467	25.12	25.8223	25.2853	25.1695	P51178;P51178-2	P51178;P51178-2	1-phosphatidylinc	PLCD1
26.1747	25.9825	26.9589	26.387	26.1544	26.9993	25.9603	25.5131	25.4516	27.1928	26.4247	26.1421	Q8N3E9;F5GXC1;I	Q8N3E9	1-phosphatidylinc	PLCD3
26.411	26.1909	26.2811	26.1167	26.4873	26.3569	26.6747	26.6399	25.2022	26.3212	26.1601	26.3663	P19174;V9GY71;V	P19174	1-phosphatidylinc	PLCG1
21.4619	NaN	NaN	NaN	NaN	NaN	NaN	NaN	NaN	NaN	NaN	NaN	P19174-2	P19174-2	1-phosphatidylinc	PLCG1
22.9388	23.1192	23.0452	22.9073	23.308	23.1946	NaN	22.9283	NaN	NaN	NaN	NaN	Q13393;Q13393-2	Q13393;Q13393-2	Phospholipase D1	PLD1
23.1554	22.535	23.146	22.8516	22.669	23.0665	NaN	NaN	NaN	23.4549	23.0734	NaN	O14939;O14939-4	O14939;O14939-4	Phospholipase D2	PLD2
25.0518	25.7151	25.0789	24.6072	25.0304	24.9259	25.192	25.4778	26.3931	24.5281	24.3427	25.7348	Q8IV08;M0R1F7;I	Q8IV08	Phospholipase D3	PLD3
22.8279	22.5483	23.0354	23.1089	23.0256	23.0586	NaN	NaN	NaN	NaN	22.9464	NaN	Q15149;Q15149-2	Q15149;Q15149-2	Plectin	PLEC
31.6016	32.0473	31.8486	31.5819	32.0784	31.96	31.5525	32.1933	31.9371	31.5593	31.9014	32.0597	Q15149-4	Q15149-4	Plectin	PLEC
23.0389	23.8867	24.2149	23.517	24.1914	NaN	NaN	NaN	NaN	24.0125	24.0349	24.9251	Q9NYT0;H0YIZ5;G	Q9NYT0	Pleckstrin-2	PLEK2
NaN	NaN	NaN	20.5595	NaN	NaN	NaN	NaN	NaN	NaN	NaN	NaN	Q9HB19;A0A087V	Q9HB19;A0A087V	Pleckstrin homolo	PLEKHA2
20.3474	NaN	NaN	20.4839	NaN	NaN	NaN	NaN	NaN	NaN	NaN	NaN	Q9HB20;F8WCR7	Q9HB20;F8WCR7	Pleckstrin homolo	PLEKHA3
25.2859	25.084	25.9472	25.2946	25.4755	25.8772	NaN	24.492	24.1021	26.4199	26.0337	25.8079	Q9HAU0;Q9HAU(	Q9HAU0;Q9HAU(	Pleckstrin homolo	PLEKHA5
22.8177	NaN	22.9007	22.7047	22.2853	22.7224	NaN	NaN	NaN	22.901	NaN	NaN	Q96S99;K7EIX0;K7	Q96S99;K7EIX0;K7	Pleckstrin homolo	PLEKHF1
NaN	NaN	NaN	NaN	NaN	NaN	NaN	NaN	NaN	23.665	NaN	NaN	Q494U1;Q494U1-	Q494U1;Q494U1-	Pleckstrin homolo	PLEKHN1
25.1381	25.0374	25.0403	24.5295	25.1179	24.9122	25.1397	NaN	NaN	24.9695	NaN	25.1934	Q8TD55;C9J4A7;C	Q8TD55;C9J4A7;C	Pleckstrin homolo	PLEKHO2
27.5753	28.3152	27.8386	27.3366	27.6792	28.0041	28.0212	27.5137	27.7376	27.8679	27.5067	27.7804	O60664;O60664-4	O60664;O60664-4	Perilipin-3	PLIN3
24.4012	24.2243	25.4591	25.2525	25.261	25.7846	26.0777	25.5142	25.9647	26.4702	26.8717	25.1894	P53350;I3L387;I3I	P53350	Serine/threonine-	PLK1
27.0408	27.3691	27.1597	26.9302	27.1906	27.1235	27.8222	27.3871	27.9261	27.4499	27.0029	27.0045	Q02809;Q02809-2	Q02809;Q02809-2	Procollagen-lysine	PLOD1
26.5594	26.6443	26.3871	25.9949	26.5829	26.4472	26.9278	27.3679	27.4935	26.4228	26.1018	26.4842	O00469-2;E7ETU9	O00469-2;E7ETU9	Procollagen-lysine	PLOD2
25.3108	25.9853	25.532	25.5854	26.1733	25.7095	26.2063	27.085	26.9769	25.9238	25.9053	26.1556	O60568;H7C2V1;I	O60568;H7C2V1	Procollagen-lysine	PLOD3
30.4452	30.3083	NaN	30.6264	30.1079	NaN	NaN	NaN	NaN	NaN	NaN	NaN	Q04941;Q04941-2	Q04941;Q04941-2	Proteolipid protei	PLP2
24.9039	24.3111	24.1991	24.5681	24.3783	24.4074	NaN	25.0031	NaN	24.9367	24.9107	24.5316	O43660;O43660-2	O43660;O43660-2	Pleiotropic regula	PLRG1
25.4855	25.4393	26.067	25.2095	25.6762	26.1085	25.5242	NaN	24.7274	25.8747	25.9496	25.6831	Q14651;C9JAM8	Q14651	Plastin-1	PLS1
29.0954	28.8286	29.6196	29.1905	29.1315	29.773	29.5171	28.7315	28.6799	30.7905	30.135	29.5203	P13797;P13797-3	P13797;P13797-3	Plastin-3	PLS3
26.507	26.8341	27.238	26.7247	26.8934	27.4927	26.8945	27.1073	27.9886	27.2752	27.0572	28.1513	Q9NRY6;I3L161;I3	Q9NRY6;I3L161;I3	Phospholipid scra	PLSCR3;TMEM256
26.7819	27.457	28.3354	27.1524	27.4725	28.3529	27.2654	26.7884	27.5346	28.1527	27.411	27.9583	Q9UIW2	Q9UIW2	Plexin-A1	PLXNA1
23.632	23.7787	24.1093	23.6214	23.1192	24.1319	24.5555	23.4778	24.2877	24.8268	24.469	NaN	O75051;Q9HCM2	O75051	Plexin-A2	PLXNA2
28.9968	29.2189	29.6348	29.1386	29.1413	29.8171	29.264	28.4536	29.0258	30.0239	29.4748	29.6042	O15031;E2PU09;A	O15031	Plexin-B2	PLXNB2
22.806	23.038	23.2276	22.9833	22.9082	23.4821	NaN	NaN	23.3947	23.3376	NaN	23.0929	Q9Y4D7;Q9Y4D7-	Q9Y4D7;Q9Y4D7-	Plexin-D1	PLXND1
24.5021	25.0206	24.8047	24.5699	25.1183	NaN	NaN	NaN	NaN	24.8366	NaN	24.8723	Q8IYS1	Q8IYS1	Peptidase M20 dc	PM20D2
24.6412	25.1688	25.2289	25.3446	25.9759	25.3954	24.9637	25.7804	26.0489	25.8284	26.1821	26.094	P29590;P29590-4	P29590;P29590-4	Protein PML	PML
22.5745	23.4174	22.6512	22.3087	22.5576	NaN	NaN	NaN	NaN	NaN	22.8965	NaN	Q92871	Q92871	Phosphomannom	PMM1
24.7377	24.2991	24.3922	24.2141	24.4342	NaN	NaN	NaN	24.5121	24.5457	24.3059	24.8129	O15305;H3BRM0;	O15305;H3BRM0;	Phosphomannom	PMM2
26.1204	24.5304	24.7938	25.7322	24.6195	24.6139	23.5693	NaN	23.6375	25.879	26.1313	24.6358	Q10713;Q10713-2	Q10713;Q10713-2	Mitochondrial-prc	PMPCA
24.7974	24.161	24.1118	24.9665	24.5652	24.361	NaN	NaN	NaN	24.5328	25.0186	NaN	O75439;G3V0E4;F	O75439;G3V0E4	Mitochondrial-prc	PMPCB
NaN	NaN	22.2607	NaN	NaN	NaN	NaN	NaN	NaN	NaN	NaN	NaN	Q13670	Q13670	Putative postmeic	PMS2P11
26.6378	25.7598	25.8996	26.039	26.1121	26.0555	24.2478	NaN	24.626	25.3559	25.755	25.7412	Q15126	Q15126	Phosphomevalona	PMVK

	22.9094	NaN	NaN	23.1483	NaN	23.3377	NaN	NaN	NaN	23.7661	23.9899	NaN	Q96T60;M0QYH2;	Q96T60;M0QYH2;	Bifunctional polyn	PNKP
NaN	NaN	24.0378	NaN	NaN	NaN	NaN	NaN	NaN	NaN	NaN	NaN	Q9UL42	Q9UL42	Paraneoplastic an	PNMA2
27.1811	26.5932	26.797	27.5672	26.4214	26.6861	25.4275	26.6568	24.5835	25.872	26.6763	25.6484	Q9H307;Q9H307-	Q9H307;Q9H307-	Pinin	PNN
24.9494	22.364	23.4296	25.2739	22.5232	24.0848	21.738	NaN	23.5083	22.7979	NaN	NaN	Q9NRX1;F8WBJ6	Q9NRX1;F8WBJ6	RNA-binding prot	PNO1
28.9695	29.9317	29.3418	28.8757	29.4802	29.0855	29.7887	29.4194	29.5197	29.4794	29.4295	29.6207	P00491;G3V5M2;	P00491;G3V5M2	Purine nucleoside	PNP
21.9032	NaN	NaN	20.9427	NaN	NaN	NaN	NaN	NaN	NaN	NaN	NaN	Q96AD5;A0A087V	Q96AD5;A0A087V	Patatin-like phosp	PNPLA2
24.3709	24.6518	24.0542	24.716	24.309	NaN	NaN	24.6612	NaN	24.3808	24.3008	NaN	Q8IY17;Q8IY17-2;	Q8IY17;Q8IY17-2;	Neuropathy targe	PNPLA6
23.6174	25.1881	24.6802	23.67	24.8281	24.6154	NaN	24.4359	NaN	23.6939	24.3085	24.1622	Q9NVS9;Q9NVS9-	Q9NVS9;Q9NVS9-	Pyridoxine-5-phos	PNPO
25.9998	25.8547	25.5576	26.1903	26.4315	25.7279	24.5601	25.7768	26.1551	24.7996	25.3314	25.2586	Q8TCS8;H7BXF6;F	Q8TCS8	Polyribonucleotid	PNPT1
28.3257	28.1669	28.7427	28.9695	28.251	29.1496	28.9859	27.9459	28.2945	29.9352	29.1643	28.45	O00592;O00592-2	O00592;O00592-2	Podocalyxin	PODXL
24.9484	25.3305	25.2852	25.0726	25.2497	25.4527	24.9768	25.7122	26.6429	25.6408	25.5581	25.4058	Q9H488;Q9H488-	Q9H488;Q9H488-	GDP-fucose prote	POFUT1
22.4396	NaN	22.0703	23.0862	21.9846	22.1572	NaN	NaN	NaN	22.801	22.4548	NaN	Q7Z3K3;Q7Z3K3-5	Q7Z3K3;Q7Z3K3-5	Pogo transposabl	POGZ
26.2382	NaN	25.1773	24.629	NaN	NaN	NaN	NaN	26.1018	25.3434	26.1714	NaN	P09884;A0A087W	P09884;A0A087W	DNA polymerase	POLA1
23.7694	NaN	23.8937	24.2125	23.3537	23.854	NaN	NaN	NaN	24.2697	24.6561	NaN	P28340;M0R2B7;I	P28340;M0R2B7;I	DNA polymerase	POLD1
23.2256	22.667	22.8147	23.5162	23.225	23.4423	NaN	NaN	NaN	23.9948	23.928	NaN	P49005;F8W8R3;A	P49005;F8W8R3;A	DNA polymerase	POLD2
NaN	NaN	NaN	21.6088	NaN	NaN	NaN	NaN	NaN	NaN	NaN	NaN	Q15054	Q15054	DNA polymerase	POLD3
23.8839	24.4498	23.7067	23.9503	24.2773	23.6127	23.615	24.0454	24.8101	23.3151	23.9398	23.951	Q9Y2S7;B4DEM9	Q9Y2S7;B4DEM9	Polymerase delta-	POLDIP2
24.0279	23.9659	23.7408	23.898	24.3118	23.9216	NaN	NaN	NaN	NaN	NaN	NaN	Q9BY77;Q9BY77-2	Q9BY77;Q9BY77-2	Polymerase delta-	POLDIP3
NaN	NaN	NaN	NaN	NaN	NaN	NaN	NaN	NaN	NaN	20.4973	NaN	P56282;P56282-2,	P56282;P56282-2,	DNA polymerase	POLE2
22.4958	22.9665	22.9748	22.7363	23.4095	NaN	NaN	24.1037	23.6465	23.8291	23.8462	24.2023	Q9NRF9	Q9NRF9	DNA polymerase	POLE3
26.1622	23.4175	24.1077	25.5317	23.8022	23.5658	NaN	NaN	NaN	23.3981	22.4557	NaN	O95602;B9ZVN9	O95602;B9ZVN9	DNA-directed RN	POLR1A
23.4749	NaN	22.9132	23.5798	NaN	23.021	NaN	NaN	NaN	NaN	NaN	NaN	Q9H9Y6;Q9H9Y6-	Q9H9Y6;Q9H9Y6-	DNA-directed RN	POLR1B
26.5157	26.0424	25.9572	26.541	26.3315	26.066	25.6232	26.347	25.807	26.4791	26.4648	26.116	O15160;E7EQB9;C	O15160;E7EQB9;C	DNA-directed RN	POLR1C
NaN	NaN	NaN	22.4871	NaN	NaN	NaN	NaN	NaN	NaN	NaN	NaN	Q9Y2S0;Q9Y2S0-2	Q9Y2S0;Q9Y2S0-2	DNA-directed RN	POLR1D
23.9445	NaN	NaN	23.9973	NaN	NaN	NaN	NaN	NaN	NaN	NaN	NaN	Q9GZS1-2;Q9GZS	Q9GZS1-2;Q9GZS	DNA-directed RN	POLR1E
27.4809	24.4751	25.4647	26.1269	24.4777	25.3281	24.8248	NaN	22.8642	23.8812	NaN	NaN	P24928;A0A0C4D	P24928;A0A0C4D	DNA-directed RN	POLR2A
27.4139	25.6829	26.5285	27.1807	26.1838	26.1628	25.2286	NaN	24.8132	26.109	25.8047	24.5156	P30876;C9J2Y9;C	P30876;C9J2Y9;C	DNA-directed RN	POLR2B
24.5738	24.7468	23.9498	24.2188	23.9675	23.7246	NaN	24.5463	NaN	NaN	23.9419	24.0313	P19387;H3BRR2	P19387	DNA-directed RN	POLR2C
26.5698	26.6417	26.3216	26.6122	26.9864	26.3069	25.3435	27.03	25.7657	25.8313	26.5111	26.2188	P19388;A0A087W	P19388;A0A087W	DNA-directed RN	POLR2E
26.6118	27.1968	26.8859	26.8373	27.0871	26.9996	25.7227	NaN	26.3141	25.3248	26.324	26.8924	P52434;C9JLU1;P5	P52434;C9JLU1;P5	DNA-directed RN	POLR2H
24.4727	24.5158	23.9676	24.2255	24.5462	23.9173	NaN	NaN	NaN	24.2713	24.576	NaN	P52435;Q9GZM3;	P52435;Q9GZM3;	DNA-directed RN	POLR2J;POLR2J2;P
NaN	NaN	NaN	23.7467	NaN	NaN	NaN	NaN	NaN	NaN	NaN	NaN	P62875	P62875	DNA-directed RN	POLR2L
24.0282	NaN	23.2833	23.4898	NaN	22.6357	NaN	NaN	NaN	23.1936	NaN	NaN	O14802;Q7Z755;F	O14802	DNA-directed RN	POLR3A
NaN	NaN	NaN	21.4783	NaN	NaN	NaN	NaN	NaN	NaN	NaN	NaN	Q9NW08;Q9NW0	Q9NW08;Q9NW0	DNA-directed RN	POLR3B
NaN	NaN	NaN	NaN	21.9283	NaN	NaN	NaN	NaN	NaN	NaN	NaN	Q9BUI4;E9PHH9	Q9BUI4;E9PHH9	DNA-directed RN	POLR3C
NaN	NaN	NaN	NaN	NaN	NaN	NaN	NaN	NaN	21.2437	NaN	NaN	E7ERZ2;F8WDV1;I	E7ERZ2;F8WDV1;I	DNA-directed RN	POLR3H
NaN	NaN	NaN	21.7128	NaN	NaN	NaN	NaN	NaN	NaN	NaN	NaN	O00411;K7EMH3	O00411;K7EMH3	DNA-directed RN	POLRMT
23.2453	23.2481	23.2925	23.8488	24.0816	23.3537	NaN	NaN	NaN	23.4785	23.5683	NaN	Q99575;E5RK39	Q99575	Ribonucleases P/I	POP1
24.2337	23.9746	23.7204	24.3437	24.1812	23.6406	NaN	NaN	NaN	24.2612	24.5742	24.0219	O75817;C9JYM0	O75817;C9JYM0	Ribonuclease P pr	POP7
25.5494	24.99	24.9937	25.5108	24.8336	24.9757	26.0056	25.8505	25.9177	26.1335	25.7709	24.965	P16435;H0Y4R2;E	P16435;H0Y4R2;E	NADPH--cytochro	POR
NaN	NaN	NaN	NaN	NaN	NaN	23.7811	NaN	NaN	NaN	NaN	NaN	Q6S8J3	Q6S8J3	POTE ankyrin don	POTEE
NaN	24.0668	25.0383	NaN	24.6978	25.4905	22.9588	24.6224	NaN	25.1174	24.9353	24.8059	Q9BYX7	Q9BYX7	Putative beta-acti	POTEKP
22.396	NaN	NaN	NaN	NaN	NaN	NaN	NaN	NaN	NaN	NaN	NaN	P14859;P14859-5,	P14859;P14859-5,	POU domain, clas	POU2F1;POU2F2;F
28.4506	29.0567	28.5882	28.3188	28.9401	28.4693	29.0685	28.2887	28.8385	28.4509	28.7519	29.2208	Q15181;Q5SQT6	Q15181;Q5SQT6	Inorganic pyropho	PPA1
NaN	NaN	NaN	NaN	23.6981	NaN	NaN	NaN	NaN	NaN	NaN	NaN	A0A0C4DGB9	A0A0C4DGB9		PPA2
24.6288	24.3385	24.1602	24.2427	24.0418	24.176	NaN	NaN	24.7374	23.984	24.0065	24.4015	Q9H2U2;Q9H2U2	Q9H2U2;Q9H2U2	Inorganic pyropho	PPA2
25.052	24.3472	24.4691	25.3422	24.1986	24.4098	23.6474	NaN	NaN	23.9699	24.8644	NaN	Q9NQ55;A0A0A6Y	Q9NQ55;A0A0A6Y	Suppressor of SW	PPAN;PPAN-P2RY1
25.2956	24.2647	25.6125	24.9616	24.9301	24.8304	24.8326	NaN	NaN	26.0933	24.8207	NaN	O14494;O14494-2	O14494;O14494-2	Lipid phosphate p	PPAP2A
NaN	NaN	NaN	NaN	NaN	NaN	21.6886	NaN	NaN	NaN	NaN	NaN	O14495	O14495	Lipid phosphate p	PPAP2B
24.4722	24.6241	24.7465	24.6384	24.7672	24.8739	24.5725	24.6186	25.1193	24.9761	25.1216	24.9143	Q06203;D6RCC8;I	Q06203	Amidophosphorib	PPAT
23.8252	23.5207	23.9843	23.5754	24.4941	23.9466	NaN	NaN	NaN	23.7126	NaN	NaN	Q9HAB8;Q9HAB8	Q9HAB8;Q9HAB8	Phosphopantothe	PPCS
23.6033	23.6446	23.8427	23.3809	23.2323	24.0776	24.1678	24.083	NaN	23.4659	23.3687	24.362	Q13136;Q13136-2	Q13136;Q13136-2	Liprin-alpha-1	PPFIA1
24.8172	24.9412	24.9394	24.4668	24.5506	24.4897	25.5297	24.9205	25.1788	24.4321	24.3147	24.7096	Q86W92;Q86W92	Q86W92;Q86W92	Liprin-beta-1	PPFIBP1
NaN	NaN	NaN	23.0163	NaN	NaN	NaN	NaN	NaN	NaN	NaN	NaN	Q8NEY8;Q8NEY8-	Q8NEY8;Q8NEY8-	Periphilin-1	PPHLN1
31.8716	33.0195	32.3533	31.7579	32.7319	32.6843	32.42	32.4988	32.5664	32.4639	32.6285	32.9642	P62937;F8WE65;C	P62937;F8WE65;C	Peptidyl-prolyl cis	PPIA
29.6229	29.7382	29.9068	29.8536	29.7051	30.053	29.429	29.5422	30.2479	29.7682	29.5151	29.3388	P23284	P23284	Peptidyl-prolyl cis	PPIB
27.5853	27.268	27.2598	27.1082	27.0235	27.2768	26.9786	27.4362	27.1765	27.1009	27.1048	27.0837	Q08752;H0Y8J0	Q08752	Peptidyl-prolyl cis	PPID
23.3953	24.3857	23.321	23.3546	23.93	23.3742	NaN	NaN	23.7626	24.2225	24.0389	24.3862	Q9UNP9;Q9UNP9	Q9UNP9;Q9UNP9	Peptidyl-prolyl cis	PPIE
24.7024	26.7501	25.2932	25.0083	26.577	25.0452	24.8006	25.6763	26.8051	24.4903	26.0083	26.3539	P30405;R4GN99;H	P30405;R4GN99;H	Peptidyl-prolyl cis	PPIF
24.494	NaN	NaN	24.4754	NaN	NaN	NaN	NaN	NaN	NaN	NaN	NaN	Q13427;C9JN15;C	Q13427;C9JN15;C	Peptidyl-prolyl cis	PPIG

23.8712	24.7537	24.3903	23.9878	24.9393	24.7691	24.1611	NaN	NaN	24.5654	24.9579	25.0187	O43447;C9JQD4;	O43447;C9JQD4;	Peptidyl-prolyl cis	PPIH
27.1642	27.1939	27.2716	27.0898	27.0099	27.213	26.2519	NaN	26.1204	27.4434	27.3471	27.2959	Q9Y3C6	Q9Y3C6	Peptidyl-prolyl cis	PPIL1
24.2741	24.8944	24.3207	24.2525	24.5002	24.3556	24.1033	NaN	NaN	24.2864	23.9413	24.3707	Q9H2H8;H7BZ14;	Q9H2H8;H7BZ14;	Peptidyl-prolyl cis	PPIL3
NaN	22.9663	NaN	23.3535	NaN	NaN	NaN	NaN	NaN	22.7061	22.7951	22.6087	Q8WUA2	Q8WUA2	Peptidyl-prolyl cis	PPIL4
25.0362	23.5939	24.802	25.5542	23.8489	24.2716	23.4496	NaN	23.5361	24.681	24.4203	NaN	O43314;A0A087W	O43314;A0A087W	Inositol hexakisph	PPIP5K2
23.5279	24.3244	24.2845	23.7444	24.1045	23.9983	24.0859	NaN	24.1434	NaN	23.8074	24.4457	P35813;P35813-3	P35813;P35813-3	Protein phosphat	PPM1A
25.397	25.3619	25.3857	24.6768	25.1278	25.1985	25.2612	25.3833	25.2913	25.2896	25.0938	25.4456	O75688;B8ZZF0;C	O75688;B8ZZF0;C	Protein phosphat	PPM1B
20.9272	NaN	NaN	NaN	NaN	NaN	NaN	NaN	NaN	NaN	NaN	NaN	O75688-2	O75688-2	Protein phosphat	PPM1B
26.2766	26.7646	26.6164	26.33	26.628	26.6387	26.9242	26.6905	27.0897	26.8092	26.8997	27.5339	P49593;P49593-2	P49593;P49593-2	Protein phosphat	PPM1F
27.8364	27.4349	27.9586	28.1689	27.8992	28.1518	27.4231	27.9571	27.5703	28.2604	28.7294	28.7001	O15355	O15355	Protein phosphat	PPM1G
27.0407	27.0755	27.0177	26.722	27.0757	27.0874	27.1826	26.5335	25.9007	26.73	26.6744	26.55	Q9Y570;Q9Y570-4	Q9Y570;Q9Y570-4	Protein phosphat	PPME1
NaN	NaN	21.469	22.3534	NaN	21.8251	NaN	NaN	NaN	21.4075	21.2255	NaN	P50336;H0YFP3	P50336	Protoporphyrinog	PPOX
29.6727	29.2125	29.8886	29.6953	29.7798	30.063	28.5535	28.724	29.0779	30.4162	30.6051	29.9237	P62136;P62136-2	P62136;P62136-2	Serine/threonine-	PPP1CA
27.8981	27.5846	27.8398	27.7838	27.8271	28.0918	27.3271	27.2429	27.6051	28.1417	28.5433	27.7331	P62140;E7ETD8;C	P62140;E7ETD8	Serine/threonine-	PPP1CB
26.9578	26.2399	26.4103	26.4116	26.4946	26.3298	26.2315	26.8576	26.2744	26.6417	26.7567	26.9734	P36873;P36873-2	P36873;P36873-2	Serine/threonine-	PPP1CC
NaN	NaN	NaN	NaN	NaN	NaN	21.7433	NaN	NaN	NaN	NaN	NaN	O60927	O60927	Protein phosphat	PPP1R11
29.6639	29.3904	29.4056	28.9101	29.2742	29.1742	30.0999	29.811	27.1942	28.9563	28.9328	28.8837	O14974;O14974-4	O14974;O14974-4	Protein phosphat	PPP1R12A
21.4853	NaN	NaN	NaN	21.747	NaN	NaN	NaN	NaN	NaN	NaN	NaN	Q9BZL4;Q9BZL4-3	Q9BZL4;Q9BZL4-3	Protein phosphat	PPP1R12C
24.6595	24.7607	24.7621	24.3129	24.7653	24.536	23.9513	24.3038	24.0716	24.0361	24.1962	24.5057	Q8WUF5;A0A087	Q8WUF5	RelA-associated ir	PPP1R13L
NaN	26.4426	25.6256	24.557	25.8139	25.1283	25.911	NaN	25.3065	24.9679	NaN	26.2001	Q96C90;Q8TAE6	Q96C90	Protein phosphat	PPP1R14B
NaN	23.5727	24.1489	23.2358	24.2274	NaN	NaN	NaN	23.638	24.382	23.8679	23.889	Q96I34;A0A087W	Q96I34	Protein phosphat	PPP1R16A
27.0062	27.9813	28.1247	27.2347	28.3839	28.2056	27.2365	27.524	27.2597	28.3443	28.0002	28.3689	Q6NYC8;A0A0G2J	Q6NYC8;A0A0G2J	Phostensin	PPP1R18
25.0156	24.5387	24.8029	24.3186	NaN	24.3514	24.8028	NaN	NaN	24.015	24.1242	NaN	P41236;E7EMN6;	P41236;E7EMN6;	Protein phosphat	PPP1R2;PPP1R2P3
20.6619	NaN	NaN	NaN	NaN	NaN	NaN	NaN	NaN	NaN	NaN	NaN	Q6ZMI0;Q6ZMI0-	Q6ZMI0;Q6ZMI0-	Protein phosphat	PPP1R21
25.2551	25.7989	25.527	24.7733	25.6114	25.6333	24.9322	24.3508	NaN	25.0035	25.0408	25.1648	Q15435;Q15435-2	Q15435;Q15435-2	Protein phosphat	PPP1R7
22.948	NaN	22.8646	23.2598	NaN	NaN	NaN	NaN	NaN	23.0665	NaN	NaN	Q12972;A0A0A0N	Q12972	Nuclear inhibitor	PPP1R8
24.8208	24.0366	25.0962	24.7778	24.5823	24.9534	24.2676	NaN	NaN	23.9497	23.9548	NaN	Q96SB3;D3DTX6	Q96SB3;D3DTX6	Neurabin-2	PPP1R9B
29.2195	28.2142	28.4403	28.7214	28.4112	28.4186	28.3864	28.3178	27.8483	28.8798	29.0027	27.8436	P67775;P67775-2	P67775;P67775-2	Serine/threonine-	PPP2CA
29.6857	29.6546	29.7503	29.5186	29.7061	29.6378	29.8079	30.0158	29.9401	29.8892	29.9443	30.0641	P30153;C9J9C1;B	P30153;C9J9C1;B	Serine/threonine-	PPP2R1A
27.3155	26.3419	26.7234	27.0634	26.6773	26.7176	26.1574	26.4989	25.4426	26.4233	26.5559	26.3016	P30154;P30154-2	P30154;P30154-2	Serine/threonine-	PPP2R1B
27.8192	27.5777	27.7574	27.7207	27.0806	27.5598	28.116	27.6949	27.4208	28.2348	28.2454	27.2737	P63151;P63151-2	P63151;P63151-2	Serine/threonine-	PPP2R2A
NaN	NaN	NaN	NaN	NaN	NaN	22.367	NaN	NaN	NaN	NaN	NaN	Q66LE6	Q66LE6	Serine/threonine-	PPP2R2D
24.7218	24.9924	25.1871	24.3731	25.1918	25.1959	25.1135	NaN	NaN	25.1703	24.9603	25.7207	Q15257;A6PVN5;	Q15257;A6PVN5;	Serine/threonine-	PPP2R4;DKFZp781
NaN	24.0008	NaN	NaN	24.3972	NaN	NaN	NaN	NaN	NaN	NaN	NaN	Q15172;Q15172-2	Q15172;Q15172-2	Serine/threonine-	PPP2R5A
25.233	23.4564	24.5078	24.1139	23.2994	NaN	23.2697	NaN	22.9999	NaN	NaN	NaN	Q13362;Q13362-4	Q13362;Q13362-4	Serine/threonine-	PPP2R5C
26.8062	26.0144	26.4943	26.2219	25.9784	26.5012	26.2112	25.7381	26.0207	26.3113	26.2448	26.3042	Q14738;E9PFR3;C	Q14738;E9PFR3;C	Serine/threonine-	PPP2R5D
26.1095	25.4599	25.8986	25.8764	25.513	25.9285	25.7565	NaN	25.2676	25.8491	25.8307	25.761	Q16537;Q16537-2	Q16537;Q16537-2	Serine/threonine-	PPP2R5E
26.8916	27.3833	27.5708	27.0734	27.2417	27.6894	27.9416	27.6502	27.4067	28.4203	28.1928	27.8633	Q08209-2;Q0820	Q08209-2;Q0820	Serine/threonine-	PPP3CA
23.2791	23.7472	23.9749	23.4904	23.8333	23.9021	24.0638	23.7484	NaN	23.8385	23.6141	24.4041	P16298;P16298-3	P16298;P16298-3	Serine/threonine-	PPP3CB
25.7316	24.9675	24.9802	24.8654	25.3075	25.119	24.4003	25.379	24.7723	25.0212	25.0227	24.4685	P60510;H3BTA2;H	P60510;H3BTA2;H	Serine/threonine-	PPP4C
25.7508	24.9729	25.2223	25.5919	25.2532	25.4428	25.0998	25.833	25.5912	25.8729	25.6995	25.5068	Q8TF05;Q8TF05-2	Q8TF05;Q8TF05-2	Serine/threonine-	PPP4R1
24.6423	23.9474	24.2874	24.7002	24.8043	24.523	23.7495	NaN	NaN	24.4242	24.8589	24.8039	Q9NY27;C9IZ04;Q	Q9NY27;C9IZ04;Q	Serine/threonine-	PPP4R2
26.3425	26.5972	26.5616	26.3761	26.4908	26.3935	27.0081	27.0567	27.2359	26.7167	26.7868	26.9726	P53041;H0YDU8;A	P53041;H0YDU8;A	Serine/threonine-	PPP5C
27.3485	26.9845	26.9914	27.2244	27.2063	27.2608	26.697	27.2707	27.5416	27.3164	27.396	27.2385	O00743;O00743-2	O00743;O00743-2	Serine/threonine-	PPP6C
25.0295	24.4233	24.4767	24.8778	25.2813	24.6831	24.8673	25.2187	24.7578	24.7165	25.2577	24.8735	Q9UPN7;K7EM28	Q9UPN7	Serine/threonine-	PPP6R1
21.1797	NaN	21.3781	NaN	NaN	NaN	NaN	NaN	NaN	NaN	NaN	NaN	O75170;O75170-2	O75170;O75170-2	Serine/threonine-	PPP6R2
26.2235	25.211	25.6989	25.7369	25.0091	25.4418	25.6217	NaN	26.3	25.8318	25.9488	26.4312	Q5H9R7;H7BXH2;	Q5H9R7;H7BXH2;	Serine/threonine-	PPP6R3
24.4996	25.0909	24.1815	24.4561	24.376	24.3102	27.1614	26.1074	26.6341	25.5445	25.801	25.4423	P50897;E9PIA8;E	P50897;E9PIA8;E	Palmitoyl-protein	PPT1
24.2738	24.0546	24.0493	23.9718	24.5522	24.2889	NaN	NaN	NaN	24.1786	24.5553	24.1341	Q96BP3;F5H7P7;	Q96BP3;F5H7P7;	Peptidylprolyl isor	PPWD1
22.4829	NaN	22.7085	22.433	23.2464	NaN	NaN	NaN	NaN	22.4778	23.3828	NaN	O60828;O60828-3	O60828;O60828-3	Polyglutamine-bir	PQBP1
NaN	NaN	NaN	23.7091	NaN	NaN	NaN	NaN	NaN	NaN	NaN	NaN	O60831;A6NP52	O60831;A6NP52	PRA1 family prote	PRAF2
26.7127	27.0594	27.3275	26.4728	26.5879	26.9631	28.8396	28.6501	28.7162	27.4159	27.0128	27.4113	O43663-4;O4366	O43663-4;O4366	Protein regulator	PRC1
24.6539	25.6388	24.5212	24.2381	25.1167	24.3912	27.8154	27.3716	27.1248	26.2823	26.1382	26.378	P42785;P42785-2	P42785;P42785-2	Lysosomal Pro-X c	PRCP
31.4349	32.1195	31.705	31.3468	32.0461	31.7648	32.151	32.2621	32.2619	32.0729	32.3039	32.5059	Q06830;A0A0A0N	Q06830;A0A0A0N	Peroxiredoxin-1	PRDX1
30.0632	30.7673	30.3646	29.6242	30.4164	30.1887	30.4152	30.7071	30.7851	30.2916	30.833	31.1902	P32119;A6NIW5;F	P32119;A6NIW5	Peroxiredoxin-2	PRDX2
28.1739	28.5327	28.1669	28.0261	28.1968	28.1499	28.0135	28.1511	28.963	26.9542	28.0839	27.8481	P30048;P30048-2	P30048;P30048-2	Thioredoxin-depe	PRDX3
27.2677	28.0446	27.1553	27.1855	28.1678	27.4835	27.86	28.918	28.6619	27.3286	27.674	28.1316	Q13162;H7C3T4;A	Q13162;H7C3T4	Peroxiredoxin-4	PRDX4
28.0427	29.0246	28.2433	27.6773	28.4937	28.1912	28.837	28.752	29.2333	29.1055	28.8207	28.9675	P30044-2;P30044	P30044-2;P30044	Peroxiredoxin-5, r	PRDX5
28.7598	28.5416	28.6333	28.533	28.4291	28.6944	28.851	28.0292	28.4068	28.8875	28.5445	28.5075	P30041	P30041	Peroxiredoxin-6	PRDX6
24.1395	23.5072	23.3741	24.2506	23.8464	23.8612	24.231	NaN	23.8679	23.9722	23.8887	24.1521	Q9HCU5;B5MC98	Q9HCU5;B5MC98	Prolactin regulato	PREB

26.1781	26.2123	26.4468	25.9183	26.0412	26.383	26.4024	26.5025	26.831	26.4937	26.2687	26.7012	P48147;H0Y5Y0	P48147	Prolyl endopeptid	PREP
NaN	NaN	NaN	22.1905	NaN	NaN	NaN	NaN	NaN	NaN	NaN	NaN	Q4J6C6;Q4J6C6-4	Q4J6C6;Q4J6C6-4	Prolyl endopeptid	PREPL
23.8462	22.6058	23.1402	23.2346	NaN	22.7936	NaN	NaN	NaN	23.5811	22.2764	NaN	P49642;F8VNY2;F	P49642;F8VNY2	DNA primase sma	PRIM1
22.0388	NaN	NaN	NaN	NaN	NaN	NaN	NaN	NaN	22.3646	22.1771	NaN	P49643;P49643-2	P49643	DNA primase larg	PRIM2
26.952	26.5899	26.7142	26.8474	26.6039	26.8093	26.6242	NaN	26.2557	26.7662	26.7674	26.4237	Q13131;Q13131-2	Q13131;Q13131-2	5-AMP-activated	PRKAA1
24.3809	24.3556	23.9625	23.9439	24.3475	23.982	NaN	NaN	24.2966	23.3242	23.7648	23.9934	Q9Y478;O43741;F	Q9Y478	5-AMP-activated	PRKAB1
26.0552	25.7099	26.2542	25.2389	25.1743	25.5361	26.3725	25.9356	26.1111	25.4671	24.7684	NaN	P17612;P17612-2	P17612;P17612-2	cAMP-dependent	PRKACA;KIN27
27.0256	26.4645	27.0666	26.2777	25.9203	26.6546	27.6478	26.6839	27.0074	26.9431	26.6477	26.5852	P22694;P22694-2	P22694;P22694-2	cAMP-dependent	PRKACB
25.2136	24.295	24.9821	25.1649	24.3584	24.4438	NaN	NaN	23.792	24.322	24.3415	23.6484	P54619;P54619-3	P54619;P54619-3	5-AMP-activated	PRKAG1
NaN	NaN	NaN	22.6394	NaN	NaN	NaN	NaN	NaN	NaN	22.4749	NaN	Q9UGJ0;E9PGP6;(	Q9UGJ0;E9PGP6;(	5-AMP-activated	PRKAG2
28.9447	29.0181	29.3446	28.2094	28.4609	28.3884	29.371	29.2067	28.3343	27.884	27.8851	28.1538	P10644;P10644-2	P10644;P10644-2	cAMP-dependent	PRKAR1A
21.1018	NaN	21.6725	NaN	NaN	NaN	21.9807	NaN	NaN	21.6744	NaN	NaN	P31321;H7BYW5;	P31321;H7BYW5;	cAMP-dependent	PRKAR1B
28.112	27.9942	28.111	28.3139	28.2655	28.2403	28.5783	28.8847	28.8883	28.4754	28.6948	29.2413	P13861;P13861-2	P13861;P13861-2	cAMP-dependent	PRKAR2A
22.3787	NaN	NaN	21.4227	NaN	NaN	NaN	NaN	NaN	NaN	NaN	NaN	P31323	P31323	cAMP-dependent	PRKAR2B
29.2156	28.5921	29.0387	29.4837	28.9617	29.0726	27.8436	27.1643	27.454	28.264	27.6308	27.3087	P17252;J3KRN5;P	P17252	Protein kinase C a	PRKCA
26.0991	26.0558	25.9096	25.7837	26.2107	25.9649	26.165	25.6025	26.4895	26.6946	26.4788	26.6721	Q05655;Q05655-2	Q05655;Q05655-2	Protein kinase C d	PRKCD
26.9548	27.6435	27.4878	27.5686	27.4257	27.6044	26.6373	27.467	26.9406	27.0787	27.4058	26.6906	Q969G5;E9PIE3	Q969G5;E9PIE3	Protein kinase C d	PRKCDBP
27.2575	26.0247	26.5012	26.4994	26.2306	26.3921	25.8712	26.0867	26.3888	26.0886	25.8895	26.476	P41743;Q05513;E	P41743	Protein kinase C i	PRKCI
28.0882	28.492	28.6313	28.2575	28.5985	28.8288	29.3133	30.5622	30.3111	29.0667	29.3463	29.6185	K7ELL7;P14314;P1	K7ELL7;P14314;P1	Glucosidase 2 sub	PRKCSH
21.4695	NaN	21.799	21.7778	NaN	21.2753	NaN	NaN	NaN	21.371	21.8097	NaN	O94806;C9JKP8;H	O94806	Serine/threonine-	PRKD3
30.7438	29.327	30.0641	30.9165	29.8034	30.2815	28.9898	28.1735	28.7355	30.0992	30.004	29.3038	P78527;P78527-2	P78527;P78527-2	DNA-dependent p	PRKDC
25.4734	24.7472	25.1946	25.6804	24.8788	24.9528	NaN	NaN	NaN	23.9895	24.5702	23.9994	O75569;O75569-3	O75569;O75569-3	Interferon-induci	PRKRA
29.1818	27.9126	27.8657	28.4952	27.7073	28.0189	27.7036	27.5368	27.4883	28.7507	28.4938	27.6705	Q99873;Q99873-4	Q99873;Q99873-4	Protein arginine N	PRMT1
26.1915	24.8741	25.3877	25.5822	25.1671	25.0118	24.9832	24.9138	NaN	24.5063	24.7339	24.5937	O60678;A0A0A0N	O60678;A0A0A0N	Protein arginine N	PRMT3
28.935	28.0834	28.2627	28.8165	28.5395	28.4403	27.8287	28.6849	28.3317	28.3549	28.6086	28.4453	O14744;O14744-2	O14744;O14744-2	Protein arginine N	PRMT5
NaN	NaN	NaN	21.2132	NaN	NaN	NaN	NaN	NaN	NaN	NaN	NaN	Q96LA8;Q96LA8-2	Q96LA8;Q96LA8-2	Protein arginine N	PRMT6
23.0262	NaN	21.921	22.3517	NaN	NaN	NaN	NaN	NaN	23.1667	NaN	NaN	Q9NVM4;Q9NVM	Q9NVM4;Q9NVM	Protein arginine N	PRMT7
NaN	NaN	NaN	NaN	NaN	NaN	NaN	NaN	NaN	22.3551	NaN	NaN	Q6P2P2;Q6P2P2-2	Q6P2P2;Q6P2P2-2	Putative protein a	PRMT9
25.3837	NaN	NaN	24.9677	23.7513	NaN	NaN	NaN	NaN	NaN	NaN	NaN	P04156;A2A2V1;P	P04156;A2A2V1;P	Major prion prote	PRNP
23.5303	24.192	23.5451	23.4551	23.977	NaN	25.4963	25.2934	NaN	23.3765	NaN	23.9179	Q9UNN8;A0A0U1	Q9UNN8;A0A0U1	Endothelial protei	PROCR
24.4301	24.9771	24.6893	24.4787	24.865	NaN	24.7201	24.6481	24.492	24.4761	24.2037	25.1001	O94903;E5RG77;E	O94903;E5RG77;E	Proline synthase c	PROSC
26.4358	26.8904	26.4366	26.1153	26.2127	26.2007	25.8054	25.8027	NaN	25.9494	25.1871	24.8263	Q86WR7;D3DRR9	Q86WR7;D3DRR9	Proline and serine	PROSER2;C10orf4
29.3844	28.9759	28.9269	29.3723	29.4169	29.146	27.8746	28.2967	28.0893	28.9095	29.0745	28.8452	Q9UMS4;F5GY56;	Q9UMS4;F5GY56	Pre-mRNA-proces	PRPF19
24.7005	24.0136	24.2129	25.0344	24.7234	24.4174	23.1263	24.1162	NaN	23.2332	23.7958	NaN	O43395;O43395-3	O43395	U4/U6 small nucle	PRPF3
26.7301	25.3285	25.921	26.7652	25.849	26.0831	24.2541	NaN	23.9875	26.2099	25.9551	24.5309	Q8WWY3;E7EVX8	Q8WWY3;E7EVX8	U4/U6 small nucle	PRPF31
23.8663	NaN	23.4573	23.8959	NaN	NaN	NaN	NaN	NaN	NaN	23.5323	NaN	Q8NAV1	Q8NAV1	Pre-mRNA-splicin	PRPF38A
24.3054	23.1703	23.4636	23.8461	23.7953	23.7153	NaN	NaN	NaN	24.1837	24.1013	NaN	Q5VTL8;A0A0A0N	Q5VTL8;A0A0A0N	Pre-mRNA-splicin	PRPF38B
27.2291	25.9767	26.2155	26.6484	26.7729	26.2996	25.8496	25.2537	25.3368	27.8704	28.1717	26.309	O43172;O43172-2	O43172;O43172-2	U4/U6 small nucle	PRPF4
27.7717	26.191	26.7286	27.7762	26.3712	26.9858	25.676	24.9235	23.5585	26.9859	27.0156	26.0855	O75400;O75400-2	O75400;O75400-2	Pre-mRNA-proces	PRPF40A
23.9781	NaN	23.7452	24.4373	23.9518	23.946	NaN	NaN	23.2156	23.9043	23.8529	23.6401	Q13523;H0YDJ3	Q13523;H0YDJ3	Serine/threonine-	PRPF4B
27.8038	26.5324	27.4612	28.4313	27.568	27.0978	23.6735	24.5006	23.2831	27.7266	27.5902	26.0235	O94906;O94906-2	O94906;O94906-2	Pre-mRNA-proces	PRPF6
30.3791	28.6834	29.5869	30.5138	29.2866	29.7459	28.18	28.0504	27.826	29.816	29.6363	29.144	Q6P2Q9;I3L3Z8;I3	Q6P2Q9	Pre-mRNA-proces	PRPF8
28.5128	27.9576	27.9735	28.1681	27.7173	28.1545	28.3099	27.5454	27.2249	28.3831	27.9584	27.4832	P60891;B1ALA9;P	P60891;B1ALA9;P	Ribose-phosphate	PRPS1
27.1515	27.018	26.7903	26.9552	26.5635	27.0279	26.5372	26.6964	26.4489	27.2206	27.2764	26.896	P11908;P11908-2	P11908;P11908-2	Ribose-phosphate	PRPS2
26.5766	25.7288	25.8689	26.3989	25.8633	25.9629	25.5726	25.2991	25.4738	26.366	26.6455	25.9643	Q14558;Q14558-2	Q14558;Q14558-2	Phosphoribosyl p	PRPSAP1
26.705	26.3165	26.1746	26.4081	26.3215	26.1404	26.598	25.7118	25.7208	26.7012	26.1755	26.8618	O60256;O60256-4	O60256;O60256-4	Phosphoribosyl p	PRPSAP2
NaN	NaN	26.37	NaN	NaN	NaN	NaN	NaN	NaN	NaN	NaN	NaN	Q5THK1;C9J9V0;H	Q5THK1;C9J9V0;H	Protein PRR14L	PRR14L
27.4151	27.462	27.2864	27.0188	27.2228	27.1483	27.0492	26.8694	26.4957	26.4261	26.7824	26.436	Q96M27;Q96M27	Q96M27;Q96M27	Protein PRRC1	PRRC1
25.8006	25.361	24.645	24.6804	24.5531	24.1976	NaN	24.0632	NaN	NaN	24.825	NaN	P48634;P48634-2	P48634;P48634-2	Protein PRRC2A	PRRC2A
26.2821	25.7197	25.529	25.035	25.5606	24.7936	25.2903	26.3198	NaN	24.3083	24.904	24.3858	E7EPN9;Q9Y520-7	E7EPN9;Q9Y520-7	Protein PRRC2C	PRRC2C
26.9581	26.9598	27.7803	26.1588	26.6536	27.3079	28.8401	27.2947	28.6218	28.1568	27.1372	26.7907	O95084;O95084-2	O95084;O95084-2	Serine protease 2	PRSS23
22.9823	NaN	22.944	22.712	NaN	NaN	NaN	NaN	NaN	23.5914	NaN	NaN	Q9NRG1;Q9NRG1	Q9NRG1;Q9NRG1	Phosphoribosyltra	PRTFDC1
25.7134	24.4962	NaN	24.0038	NaN	NaN	28.0782	24.2463	NaN	NaN	NaN	NaN	P07602;C9JIZ6;P0	P07602;C9JIZ6;P0	Prosaposin;Sapos	PSAP
27.9086	28.0644	27.8037	27.3372	27.821	27.5063	27.9392	27.4277	27.1342	28.0525	28.0374	27.7508	Q9Y617;Q9Y617-2	Q9Y617;Q9Y617-2	Phosphoserine an	PSAT1
NaN	NaN	NaN	NaN	NaN	22.8074	NaN	NaN	NaN	NaN	NaN	NaN	Q9NYI0;E5RJ29;Q	Q9NYI0;E5RJ29;Q	PH and SEC7 dom	PSD3
26.2905	25.73	26.5159	26.3125	26.0953	27.0888	25.5894	25.3772	25.2825	26.4425	25.7722	26.0468	P49768;P49768-2	P49768;P49768-2	Presenilin-1;Prese	PSEN1
NaN	NaN	NaN	21.7931	NaN	NaN	NaN	NaN	NaN	NaN	NaN	NaN	P49810;B1AP22;P	P49810;B1AP22;P	Presenilin-2;Prese	PSEN2
26.1583	NaN	25.5453	25.9916	NaN	NaN	NaN	NaN	NaN		25.9462	NaN	O75475;O75475-2	O75475;O75475-2	PC4 and SFRS1-int	PSIP1
29.0292	30.1928	29.6567	28.9346	30.2527	29.5386	29.3769	30.2421	30.1705	29.4511	29.8701	30.0938	P25786;P25786-2	P25786;P25786-2	Proteasome subu	PSMA1
28.7741	30.0121	29.3576	28.7058	29.874	29.2106	29.258	30.2148	30.188	28.9658	29.413	29.9424	P25787;A0A024R	P25787;A0A024R	Proteasome subu	PSMA2

28.564	29.1626	28.9863	28.4737	28.9714	28.9722	28.8427	29.7659	29.7437	28.7557	29.2997	29.617	P25788;P25788-2	P25788;P25788-2	Proteasome subu	PSMA3
28.5202	28.4966	28.3212	27.8831	27.9799	28.3089	27.9118	28.0541	27.9894	28.0533	28.2624	27.5125	P25789;H0YN18;H	P25789;H0YN18;H	Proteasome subu	PSMA4
28.9185	29.9811	29.557	28.991	29.7167	29.5019	29.2379	30.0852	29.7684	29.1585	29.61	30.0161	P28066;P28066-2	P28066;P28066-2	Proteasome subu	PSMA5
28.5816	30.0377	29.106	28.4152	29.7782	29.0543	29.2016	29.8102	29.7689	29.0973	29.2667	29.7056	P60900;G3V5Z7;G	P60900;G3V5Z7;G	Proteasome subu	PSMA6
28.9259	30.0227	29.275	28.8313	29.7086	29.4147	29.2699	30.0321	29.9618	29.051	29.515	29.722	O14818;O14818-2	O14818;O14818-2	Proteasome subu	PSMA7
27.7294	28.7167	28.2391	27.7453	28.4329	28.2466	27.8644	28.1053	28.4037	27.6606	27.9322	28.3987	P20618	P20618	Proteasome subu	PSMB1
23.506	24.7078	24.5497	23.6989	25.5392	24.1817	NaN	25.0389	NaN	23.6646	24.0831	NaN	P40306;J3QQN1	P40306;J3QQN1	Proteasome subu	PSMB10
27.9654	28.4543	28.158	27.7233	28.4109	28.0935	28.121	28.1612	28.165	28.1036	28.2105	28.4247	P49721;A0A087W	P49721	Proteasome subu	PSMB2
27.2806	28.1338	27.69	27.2316	27.8293	27.5333	27.3264	27.9934	27.8718	26.973	27.4739	27.8011	P49720;A0A087W	P49720;A0A087W	Proteasome subu	PSMB3
28.4016	29.2481	28.6246	28.0529	28.9166	28.4956	28.6154	28.9068	28.7698	28.1739	28.5855	28.8261	P28070	P28070	Proteasome subu	PSMB4
28.7369	29.5467	29.0886	28.4476	29.2734	28.9331	28.8944	29.6749	29.5438	28.9068	29.2874	29.4718	P28074;P28074-3	P28074;P28074-3	Proteasome subu	PSMB5
28.2823	29.5829	28.8629	28.1769	29.3465	28.8461	28.9344	29.8633	29.527	28.8289	29.2936	28.9975	P28072;J3L3X7;A0	P28072	Proteasome subu	PSMB6
27.8508	28.1893	27.3654	27.28	27.813	27.3736	27.5907	27.4383	27.339	27.4953	27.9023	26.8313	Q99436;Q5TBG5;	Q99436	Proteasome subu	PSMB7
25.0494	27.0715	26.1074	24.6939	26.9465	25.4847	26.341	26.9335	26.7843	23.8862	24.4805	26.6637	P28062;P28062-2	P28062;P28062-2	Proteasome subu	PSMB8;PSM8
24.8572	26.4306	25.6387	24.6159	26.5136	25.7116	24.9961	25.7734	25.4678	24.3147	24.7448	24.8685	P28065;A2ACR1;P	P28065;A2ACR1;P	Proteasome subu	PSMB9
30.224	30.1884	30.072	30.273	30.3398	30.1285	29.486	29.9418	29.3309	29.9747	30.0324	29.6463	P62191;P62191-2	P62191;P62191-2	26S protease regu	PSMC1
30.9307	30.3617	30.3375	30.9401	30.577	30.4976	29.6232	30.2662	29.7389	30.4667	30.3366	29.8208	P35998;P35998-2	P35998;P35998-2	26S protease regu	PSMC2
30.659	30.2107	30.0437	30.447	30.238	30.03	29.6119	30.187	29.5134	29.9739	30.0103	29.6842	P17980;R4GNH3;I	P17980;R4GNH3;I	26S protease regu	PSMC3
30.3119	30.1407	29.9568	30.4451	30.3353	30.1244	29.4319	30.3929	29.6869	30.3174	30.4335	29.8845	P43686;P43686-2	P43686;P43686-2	26S protease regu	PSMC4
30.1831	30.2144	30.0526	30.6663	30.424	30.126	29.4621	30.2527	29.6617	30.1084	30.1747	29.802	P62195;J3QQM1;	P62195;J3QQM1	26S protease regu	PSMC5
NaN	NaN	NaN	22.2363	NaN	NaN	NaN	NaN	NaN	NaN	NaN	NaN	P62195-2;J3QSA9	P62195-2;J3QSA9	26S protease regu	PSMC5
30.8737	30.5019	30.5033	30.9424	30.5543	30.7078	29.9474	30.4787	30.0405	30.8706	30.4973	30.0921	P62333;A0A087X	P62333;A0A087X	26S protease regu	PSMC6
30.6258	29.2093	29.9222	30.6218	29.524	30.0947	29.0937	29.421	28.8351	30.262	30.1562	29.2169	Q99460;A0A087W	Q99460;A0A087W	26S proteasome r	PSMD1
24.2336	24.1259	24.5806	24.1359	24.3569	24.7543	24.2141	NaN	24.1384	24.4588	25.1234	NaN	O75832;B1AJY7;B	O75832;B1AJY7;B	26S proteasome r	PSMD10
30.9568	30.6233	30.8454	31.3106	31.0646	31.1034	29.7996	30.5913	30.2048	30.7437	30.8516	30.4411	O00231;O00231-2	O00231;O00231-2	26S proteasome r	PSMD11
30.0946	29.2816	29.4928	30.2421	29.4618	29.6983	28.811	29.2829	28.7761	29.6069	29.456	29.0546	O00232;O00232-2	O00232;O00232-2	26S proteasome r	PSMD12
30.0022	28.9644	29.3954	30.0488	29.4422	29.5813	28.486	29.2183	28.8112	29.3968	29.5316	28.9738	Q9UNM6;Q9UNM	Q9UNM6;Q9UNM	26S proteasome r	PSMD13
29.4065	29.4415	29.2741	29.6168	29.5846	29.4452	28.7216	29.3883	28.8386	29.5909	29.8027	29.045	O00487;C9JW37	O00487	26S proteasome r	PSMD14
31.7794	30.5308	31.2447	31.6697	30.821	31.3217	30.5258	30.7927	29.7384	31.0333	30.9799	30.0918	Q13200;Q13200-3	Q13200;Q13200-3	26S proteasome r	PSMD2
30.4766	29.2097	29.8852	30.6964	29.6377	29.947	29.3707	29.2598	29.3659	30.1036	29.8705	29.2565	O43242;O43242-2	O43242;O43242-2	26S proteasome r	PSMD3
28.0578	27.7651	27.3295	27.8544	27.4201	27.5765	26.4645	27.109	26.0918	27.0843	27.3922	26.7363	P55036;Q5VWC4;	P55036;Q5VWC4;	26S proteasome r	PSMD4
27.088	26.4011	26.7724	26.8485	26.1474	26.6053	26.9106	25.5329	25.7536	26.7833	26.3909	26.1431	Q16401;Q16401-2	Q16401;Q16401-2	26S proteasome r	PSMD5
29.8137	29.0625	29.3009	29.8068	29.3385	29.4178	28.7122	29.1428	28.6599	29.571	29.599	28.8537	Q15008;Q15008-4	Q15008;Q15008-4	26S proteasome r	PSMD6
29.4983	28.3921	29.1241	29.6346	28.9826	29.3211	28.417	28.5311	28.2672	28.992	29.3065	28.0322	P51665;H3BNT7;H	P51665;H3BNT7;H	26S proteasome r	PSMD7
29.2493	28.1728	28.4619	29.4069	28.5152	28.6366	27.3981	27.6589	27.3616	28.6114	28.6624	27.6103	P48556;R4GMR5;	P48556;R4GMR5;	26S proteasome r	PSMD8
28.0738	28.2885	28.0465	27.8908	28.0061	27.9009	28.1899	28.4587	27.4573	28.468	28.4178	27.9494	O00233;J3KN29;F	O00233;J3KN29;F	26S proteasome r	PSMD9
28.3399	29.4266	29.2207	28.4465	29.0918	29.2277	28.925	28.4904	28.3348	28.7569	28.7264	28.5347	Q06323;Q06323-2	Q06323;Q06323-2	Proteasome activ	PSME1
28.9959	30.1571	29.8178	28.951	29.9413	29.7885	29.4391	29.7833	29.7537	29.253	29.4989	29.778	Q9UL46;A0A087X	Q9UL46;A0A087X	Proteasome activ	PSME2
26.9614	26.9328	26.5888	27.1896	27.3787	26.7519	23.8359	26.2915	26.2025	26.6546	27.3361	27.2482	P61289;P61289-2	P61289;P61289-2	Proteasome activ	PSME3
27.3617	25.8437	26.6598	27.4474	26.2929	26.9265	26.0337	NaN	25.4365	26.285	26.2824	25.8901	Q14997;Q14997-2	Q14997;Q14997-2	Proteasome activ	PSME4
23.3515	NaN	22.4706	23.0466	23.6306	NaN	23.3573	NaN	NaN	23.2225	NaN	23.6516	Q92530;Q5QPM7	Q92530;Q5QPM7	Proteasome inhib	PSMF1
24.8387	24.4579	24.3055	24.3665	24.673	24.2213	NaN	NaN	25.0014	24.578	25.3252	25.4664	O95456;O95456-2	O95456;O95456-2	Proteasome asser	PSMG1
24.4908	23.7994	24.4188	24.7999	24.6138	NaN	23.9492	24.1485	24.2868	24.5048	24.3542	24.1427	Q969U7;Q969U7-	Q969U7;Q969U7-	Proteasome asser	PSMG2
23.3941	23.2478	22.9153	23.3059	23.4034	23.1975	NaN	NaN	NaN	NaN	NaN	NaN	Q9BT73	Q9BT73	Proteasome asser	PSMG3
NaN	NaN	22.6673	22.2424	22.7383	22.7058	NaN	NaN	NaN	22.6538	23.1904	NaN	Q5JS54;Q5JS54-2;	Q5JS54;Q5JS54-2;	Proteasome asser	PSMG4
27.0344	27.0107	27.1758	27.2033	27.4679	27.2752	26.6761	27.39	27.1554	27.5741	27.8269	27.969	Q8WXF1;Q8WXF1	Q8WXF1;Q8WXF1	Paraspeckle comp	PSPC1
24.8661	25.1972	24.9831	23.7409	24.3268	24.6247	25.1858	NaN	24.3359	25.0579	24.717	24.7187	P78330;C9JBI3;C9	P78330;C9JBI3	Phosphoserine ph	PSPH
29.3188	28.6481	28.5353	28.8233	28.5795	28.7105	28.387	28.3003	27.9953	29.2347	29.111	28.8265	P26599;P26599-2	P26599;P26599-2	Polypyrimidine tra	PTBP1
24.9252	24.826	24.142	24.9309	24.5236	NaN	NaN	NaN	NaN	23.9681	24.08	NaN	O95758;O95758-2	O95758;O95758-2	Polypyrimidine tra	PTBP3
22.4594	NaN	22.4313	23.5563	22.4593	22.374	NaN	NaN	NaN	NaN	NaN	NaN	Q96EY7;Q96EY7-2	Q96EY7;Q96EY7-2	Pentatricopeptide	PTCD3
NaN	NaN	NaN	20.8293	NaN	NaN	NaN	NaN	NaN	NaN	NaN	NaN	P48651;P48651-3	P48651;P48651-3	Phosphatidylserin	PTDSS1
NaN	NaN	NaN	22.4901	NaN	NaN	NaN	NaN	NaN	NaN	NaN	NaN	Q9BVG9;E9PS47;E	Q9BVG9;E9PS47;E	Phosphatidylserin	PTDSS2
22.6444	NaN	22.8145	22.5008	NaN	NaN	NaN	NaN	NaN	NaN	22.6164	NaN	P60484;A0A0U1R	P60484;A0A0U1R	Phosphatidylinosi	PTEN
22.3764	NaN	22.3214	21.9927	22.5899	NaN	NaN	22.6684	NaN	24.0266	24.4432	22.9583	Q96BW5;Q96BW5	Q96BW5;Q96BW5	Phosphotriesteras	PTER
24.6993	24.8504	24.5737	24.947	24.9732	24.2255	24.1931	NaN	NaN	24.9591	25.3618	25.1506	Q9H7Z7;A6NHH0;	Q9H7Z7;A6NHH0;	Prostaglandin E sy	PTGES2
29.0526	27.882	28.2185	28.5592	28.3156	27.8935	28.8817	29.2674	28.3865	28.3541	28.6342	28.5475	Q15185;Q15185-4	Q15185;Q15185-4	Prostaglandin E sy	PTGES3
24.8673	24.5048	26.0429	25.0011	24.057	25.1943	NaN	NaN	NaN	22.8362	23.2385	23.4279	Q9P2B2	Q9P2B2	Prostaglandin F2 r	PTGFRN
24.4273	25.6164	25.1606	24.4538	25.2876	25.2209	24.7668	NaN	24.5436	24.5897	24.6071	25.059	Q14914;Q14914-2	Q14914;Q14914-2	Prostaglandin red	PTGR1
22.3711	NaN	22.2123	22.2724	NaN	NaN	NaN	NaN	NaN	22.1778	NaN	NaN	Q8N8N7;G3V3Y1;	Q8N8N7;G3V3Y1;	Prostaglandin red	PTGR2
22.2148	NaN	22.4059	22.6544	22.9653	NaN	24.8997	24.7144	25.4773	24.1989	23.1954	23.6137	P35354;Q6ZYK7	P35354	Prostaglandin G/H	PTGS2

25.4079	25.2239	25.8447	25.2257	24.9043	25.5624	25.85	24.2198	NaN	25.7282	25.1936	25.0157	Q05397;Q05397-5	Q05397;Q05397-5	Focal adhesion kir	PTK2
27.9072	28.8464	28.9592	27.9636	28.5252	28.9737	29.0416	28.0167	28.5268	29.2077	28.421	28.2434	Q13308;Q13308-6	Q13308;Q13308-6	Inactive tyrosine-p	PTK7
27.0709	27.0285	27.7193	26.4159	27.3348	27.507	25.9922	28.3896	27.9376	27.3763	28.2208	29.9517	P06454;P06454-2	P06454;P06454-2	Prothymosin alph	PTMA
25.9613	NaN	25.9935	25.6068	25.8217	26.362	NaN	25.6958	NaN	27.2365	27.3752	NaN	P20962;F5H7R9;F	P20962;F5H7R9;F	Parathymosin	PTMS
23.3785	NaN	23.4822	23.4083	NaN	NaN	NaN	NaN	NaN	23.8083	23.5964	NaN	Q93096;A0A0D9S	Q93096;A0A0D9S	Protein tyrosine p	PTP4A1
24.3817	23.974	24.4922	24.1145	24.0015	24.5147	24.5908	NaN	NaN	24.9619	24.5405	NaN	Q12974;Q12974-4	Q12974;Q12974-4	Protein tyrosine p	PTP4A2
27.5595	27.198	27.0427	26.9367	27.2514	27.0543	27.0904	27.5454	27.884	27.8934	27.679	27.069	P18031;B4DSN5	P18031;B4DSN5	Tyrosine-protein p	PTPN1
27.0583	27.7297	27.6186	27.0104	27.6091	27.5288	28.021	27.7858	28.071	27.6346	27.7834	27.9569	Q06124;Q06124-2	Q06124;Q06124-2	Tyrosine-protein p	PTPN11
24.4975	NaN	23.3133	23.8159	23.3309	NaN	23.8211	NaN	NaN	23.3231	NaN	NaN	Q05209;Q05209-2	Q05209;Q05209-2	Tyrosine-protein p	PTPN12
NaN	NaN	NaN	NaN	NaN	NaN	NaN	NaN	NaN	NaN	NaN	25.3793	Q12923;Q12923-3	Q12923;Q12923-3	Tyrosine-protein p	PTPN13
25.5963	25.7837	26.9889	25.8405	26.0266	26.9693	26.3814	23.9791	25.7243	27.1007	26.7844	26.4173	Q15678;E2J9M0	Q15678	Tyrosine-protein p	PTPN14
25.9542	25.5206	25.538	25.7854	25.3161	25.3144	25.3483	25.6689	25.5495	26.2468	26.5851	25.4585	P17706;P17706-2	P17706;P17706-2	Tyrosine-protein p	PTPN2
26.4021	26.2564	25.8158	25.7539	25.5728	25.3554	25.8529	26.0393	26.7459	25.6443	25.5814	26.1513	Q9H3S7;R4GMM7	Q9H3S7	Tyrosine-protein p	PTPN23
NaN	NaN	NaN	NaN	NaN	NaN	NaN	NaN	22.7744	NaN	NaN	NaN	P43378;A0A0A0M	P43378;A0A0A0M	Tyrosine-protein p	PTPN9
26.6828	26.389	27.3773	26.7268	26.3142	27.5263	26.9539	26.3824	26.8025	27.9745	27.3531	27.1867	P18433;P18433-2	P18433;P18433-2	Receptor-type tyr	PTPRA
26.4748	26.51	27.2072	26.5575	26.6734	27.2717	27.2167	27.0911	27.675	27.9596	27.7354	27.5687	P23469;P23469-2	P23469;P23469-2	Receptor-type tyr	PTPRE
25.1513	25.3169	25.3798	24.7991	24.9286	25.4411	25.4121	24.7907	25.6102	25.3537	24.9838	25.1308	P10586;P10586-2	P10586;P10586-2	Receptor-type tyr	PTPRF
26.0926	25.4589	26.2946	26.0052	25.5695	26.4126	26.1306	25.6734	25.8925	27.3091	26.5291	26.5282	P23470;A0A087W	P23470;A0A087W	Receptor-type tyr	PTPRG
26.8333	26.7667	27.5921	26.6572	26.8072	27.7745	25.9145	23.9686	25.5029	26.9113	25.684	26.0845	Q12913;A0A087W	Q12913;A0A087W	Receptor-type tyr	PTPRJ
23.8755	23.9996	24.8841	24.1703	23.6609	24.9743	24.4412	24.4128	NaN	24.6888	24.4025	24.2095	Q5TG12;E9PGC5;(	Q5TG12;E9PGC5;(	Protein-tyrosine-p	PTPRK
24.6883	24.8577	25.374	24.6961	24.5548	25.572	24.9981	NaN	25.0917	25.1202	24.6627	NaN	P28827;E7EPS8;P2	P28827;E7EPS8;P2	Receptor-type tyr	PTPRM
NaN	22.5318	NaN	NaN	NaN	NaN	NaN	NaN	NaN	25.1495	NaN	NaN	Q13332;Q13332-6	Q13332;Q13332-6	Receptor-type tyr	PTPRS
30.0213	29.3945	29.625	29.8494	29.0247	29.6155	29.2228	29.9574	28.7929	29.0942	28.7478	28.5349	Q6NZI2;Q6NZI2-2	Q6NZI2;Q6NZI2-2	Polymerase I and	PTRF
25.8772	25.2372	25.1694	25.6402	25.1118	24.7827	25.1014	24.53	25.192	24.7528	24.0063	24.2121	Q9Y3E5;J3KQ48	Q9Y3E5;J3KQ48	Peptidyl-tRNA hyc	PTRH2
23.8006	24.5797	23.7604	23.8107	24.6051	23.0893	24.4723	24.3892	NaN	23.9421	24.0754	NaN	Q6GMV3	Q6GMV3	Putative peptidyl-	PTRHD1
25.6785	26.0933	26.3965	25.7087	25.7091	26.4251	25.514	25.6257	26.738	25.8	25.9933	27.2137	P53801;A8MXQ1;	P53801;A8MXQ1	Pituitary tumor-tr	PTTG1IP
26.5264	27.2126	26.163	26.3805	26.8385	25.9144	29.6432	29.1095	28.139	27.4546	26.9734	26.9906	P26022	P26022	Pentraxin-related	PTX3
26.4533	25.625	26.2044	26.8993	26.0412	26.2349	24.5183	24.0729	24.2528	25.7574	26.0081	25.9902	Q9UHX1-6;A0A0J	Q9UHX1-6;A0A0J	Poly(U)-binding-sp	PUF60
25.3698	24.6571	24.7065	24.8381	24.1968	24.0938	23.9509	25.1462	23.8446	23.6665	24.1909	NaN	Q14671-2;Q5T1Z4	Q14671-2;Q5T1Z4	Pumilio homolog	PUM1
22.4533	NaN	NaN	21.6534	NaN	NaN	NaN	NaN	NaN	NaN	NaN	NaN	Q8TB72;Q8TB72-4	Q8TB72;Q8TB72-4	Pumilio homolog	PUM2
28.4834	28.5091	28.3452	28.7298	28.8294	28.3685	27.7912	29.0397	28.616	28.1463	28.57	28.7203	Q00577	Q00577	Transcriptional ac	PURA
26.9004	26.4722	26.5507	26.8157	26.6791	26.7313	26.5149	26.8843	27.0478	26.5968	26.9126	26.9369	Q96QR8;Q9UJV8;	Q96QR8	Transcriptional ac	PURB
23.4136	22.4756	22.7161	23.1607	22.8802	22.6158	NaN	NaN	NaN	22.9763	22.8744	22.7822	Q9Y606;F5H1S9;G	Q9Y606;F5H1S9;G	tRNA pseudouridi	PUS1
23.192	NaN	22.8667	22.9858	NaN	23.0872	NaN	NaN	NaN	23.0581	22.9776	NaN	Q96PZ0;E7EUH7;I	Q96PZ0;E7EUH7	Pseudouridylate s	PUS7
NaN	NaN	21.3439	22.3279	NaN	NaN	NaN	NaN	NaN	NaN	NaN	NaN	Q8N0Z8;J3KTG4;C	Q8N0Z8;J3KTG4;C	tRNA pseudouridi	PUSL1
28.5464	28.2615	28.7674	28.6177	27.9003	28.4731	29.4186	28.7955	28.7031	29.0498	28.4871	28.5248	P15151;A0A0C4D	P15151;A0A0C4D	Poliovirus recepto	PVR
23.6677	23.44	24.4042	23.6454	23.5085	23.9491	NaN	NaN	NaN	NaN	23.4901	23.3347	Q92692	Q92692	Nectin-2	PVRL2
26.6331	26.4831	26.9788	26.6439	26.4831	27.108	27.6298	26.8919	26.757	27.8672	27.0822	26.8418	Q92692-2;K7EKE8	Q92692-2	Nectin-2	PVRL2
NaN	NaN	23.3708	NaN	NaN	NaN	NaN	NaN	NaN	NaN	NaN	NaN	Q9NQS3;Q9NQS3	Q9NQS3;Q9NQS3	Nectin-3	PVRL3
26.0532	24.8129	25.3662	26.1354	25.4448	25.3031	23.5782	25.9407	NaN	23.7531	25.2091	24.1837	Q13610;B4DJV5;C	Q13610;B4DJV5	Periodic tryptopha	PWP1
25.1193	23.5837	24.7125	26.4497	25.0477	24.538	22.5338	NaN	NaN	25.0596	25.3016	NaN	Q15269;A0A0B4J2	Q15269;A0A0B4J2	Periodic tryptopha	PWP2
28.8649	28.7632	28.3444	27.8795	28.4678	28.0466	30.1399	29.1356	30.0584	29.5615	28.8115	28.3852	Q92626;H7C1W1;	Q92626	Peroxidasin homo	PXDN
25.7276	25.4175	24.8858	24.3687	25.258	25.0825	25.11	NaN	24.7927	23.9668	24.1508	25.0154	P49023;F5GZ78;P	P49023;F5GZ78;P	Paxillin	PXN
24.7144	25.2575	23.7436	25.1252	25.0913	24.572	23.6823	NaN	25.3201	22.909	23.9856	24.4583	P32322;P32322-3	P32322;P32322-3	Pyrroline-5-carbo	PYCR1
25.8353	25.4103	25.3076	26.0354	25.8102	25.3007	NaN	24.5339	25.1785	23.5991	24.4855	NaN	Q96C36;A0A087W	Q96C36;A0A087W	Pyrroline-5-carbo	PYCR2
25.8239	25.5416	25.5535	25.9569	26.0858	25.7403	25.2002	25.8906	NaN	25.4761	25.8457	25.9194	Q53H96;Q53H96-	Q53H96;Q53H96-	Pyrroline-5-carbo	PYCRL
29.8965	29.3917	29.5628	29.4249	29.3332	29.4885	30.0132	29.7582	30.0483	30.5253	30.2384	30.1034	P11216;H0Y4Z6	P11216	Glycogen phosph	PYGB
29.6145	29.211	29.301	29.1917	28.999	29.1757	29.1458	28.6147	28.6566	29.2566	28.9424	28.7127	P06737;P06737-2	P06737;P06737-2	Glycogen phosph	PYGL
29.4712	28.8914	28.9924	29.4962	29.3088	29.17	28.9067	28.9233	29.0801	29.3877	29.4425	29.3366	P47897;P47897-2	P47897;P47897-2	Glutamine--tRNA	QARS
24.5859	24.8248	24.3619	24.0869	24.8011	24.2585	24.357	25.3241	25.3631	25.0524	25.681	25.824	P09417;P09417-2	P09417;P09417-2	Dihydropteridine	QDPR
NaN	NaN	NaN	23.1206	NaN	NaN	NaN	NaN	NaN	NaN	NaN	NaN	Q5XKP0	Q5XKP0	Protein QIL1	QIL1
26.1698	25.8639	25.1861	24.9506	25.1721	24.6082	NaN	NaN	NaN	23.699	NaN	NaN	Q96PU8;Q96PU8-	Q96PU8;Q96PU8-	Protein quaking	QKI
25.4244	24.3097	24.4385	25.1931	24.172	24.6724	NaN	NaN	NaN	24.6902	24.7479	NaN	Q2TAL8	Q2TAL8	Glutamine-rich pr	QRICH1
26.9779	27.2473	26.6935	26.4641	26.5302	26.0984	29.775	28.8514	27.7046	27.2363	26.8206	26.6116	O00391;O00391-2	O00391;O00391-2	Sulfhydryl oxidase	QSOX1
23.5243	23.7086	23.5658	23.6767	23.693	23.8929	23.9239	23.9635	23.8576	24.2056	23.389	NaN	Q6ZRP7;A0A087X	Q6ZRP7;A0A087X	Sulfhydryl oxidase	QSOX2
24.6482	23.5367	23.5399	23.9586	23.8596	23.738	23.2689	23.8424	24.1119	24.4856	24.5101	24.1245	Q9BXR0;Q9BXR0-	Q9BXR0;Q9BXR0-	Queuine tRNA-rib	QTRT1
24.9581	24.7766	24.3045	24.4468	24.3247	24.4504	25.1743	NaN	NaN	24.7313	25.4078	23.8829	Q9H974;Q9H974-	Q9H974;Q9H974-	Queuine tRNA-rib	QTRTD1
NaN	NaN	NaN	22.1158	NaN	NaN	NaN	NaN	NaN	NaN	NaN	NaN	Q96D70;K7EIG9	Q96D70;K7EIG9	R3H domain-cont	R3HDM4
30.0847	29.8931	30.1631	29.9355	29.8561	30.1842	30.4746	30.1081	30.4262	30.5131	30.0249	30.3192	P61026;P59190;P	P61026	Ras-related protei	RAB10
30.0312	30.1332	30.3666	29.9556	30.0394	30.4725	30.5655	29.7655	30.2076	30.5605	30.1696	30.0198	Q15907;Q15907-2	Q15907;Q15907-2	Ras-related protei	RAB11B;RAB11A

25.5262	25.3722	25.9482	25.223	25.3771	25.4191	24.4598	NaN	23.9649	24.7245	24.6302	NaN	Q6WKZ4;Q6WKZ4	Q6WKZ4;Q6WKZ4	Rab11 family-inte	RAB11FIP1
25.7229	26.2673	26.4747	25.4516	26.2142	25.8539	23.7939	24.6723	24.9637	25.308	25.2958	25.5074	Q9BXF6	Q9BXF6	Rab11 family-inte	RAB11FIP5
26.0393	25.4325	25.8784	25.6916	25.3489	25.7425	25.9315	25.3195	25.2959	25.5748	25.1733	25.7314	Q6IQ22	Q6IQ22	Ras-related protei	RAB12
28.3217	28.7947	29.3047	28.5762	29.085	29.3137	28.4492	28.1063	28.9297	29.1837	29.2147	29.1003	P51153;A0A087W	P51153	Ras-related protei	RAB13
28.6795	28.7403	28.9066	28.5467	28.6293	28.8446	28.448	28.159	28.7242	29.0557	29.149	28.7852	P61106;X6RFL8	P61106;X6RFL8	Ras-related protei	RAB14
27.1749	26.8671	27.1646	26.9892	26.8788	27.1064	27.4134	27.2619	27.2575	27.4339	27.2372	27.1408	Q9NP72;A0A087X	Q9NP72;A0A087X	Ras-related protei	RAB18
30.9666	30.4497	30.7182	30.3518	30.4013	30.5637	30.9993	30.8654	30.9814	30.4403	30.3316	30.8154	P62820;E7END7;P	P62820;E7END7;P	Ras-related protei	RAB1A
29.3743	29.3621	29.8378	29.4322	29.0198	29.4404	30.3934	29.774	29.7887	30.2034	29.6578	29.7016	Q9H0U4;E9PLD0;	Q9H0U4;E9PLD0	Ras-related protei	RAB1B
22.1902	NaN	21.573	NaN	NaN	NaN	NaN	NaN	NaN	NaN	NaN	NaN	Q9NX57	Q9NX57	Ras-related protei	RAB20
27.9259	27.5722	27.9775	27.8336	27.1895	27.8803	28.2318	27.2304	27.4975	28.1772	27.8604	27.3097	Q9UL25	Q9UL25	Ras-related protei	RAB21
25.9657	25.8805	26.4067	25.5901	25.6364	26.2111	26.3247	26.575	27.199	26.0124	25.8809	27.0417	Q9UL26	Q9UL26	Ras-related protei	RAB22A
27.2704	27.2504	27.4837	27.0024	27.0964	27.5281	27.5751	27	27.3134	28.2577	27.8912	27.8221	Q9ULC3	Q9ULC3	Ras-related protei	RAB23
23.3232	22.2153	22.9988	22.8312	22.8267	22.4882	NaN	NaN	NaN	NaN	22.7997	NaN	Q969Q5;D6RFW3	Q969Q5;D6RFW3	Ras-related protei	RAB24
25.2899	25.4922	25.4181	25.1812	25.136	25.1193	25.2284	NaN	24.6916	25.3315	24.8551	25.2443	P51159;H3BN55;H	P51159;H3BN55;H	Ras-related protei	RAB27A
21.629	21.9372	22.192	21.9455	21.6014	NaN	22.8838	NaN	21.9359	22.5076	21.904	NaN	O00194;K7ES41	O00194;K7ES41	Ras-related protei	RAB27B
24.2498	NaN	24.0396	24.3331	NaN	24.1465	24.7683	NaN	NaN	NaN	NaN	NaN	O14966;O14966-2	O14966	Ras-related protei	RAB29
28.5295	28.1404	28.7164	28.5328	27.9446	28.5915	28.6269	28.0346	28.2373	28.6578	28.28	28.1452	P61019;P61019-2	P61019;P61019-2	Ras-related protei	RAB2A
22.7122	NaN	NaN	22.6818	NaN	NaN	NaN	NaN	NaN	22.8188	NaN	NaN	Q8WUD1;Q5HYI5	Q8WUD1;Q5HYI5	Ras-related protei	RAB2B;DKFZp313C
21.5817	NaN	21.3702	21.2307	NaN	NaN	NaN	NaN	NaN	22.1052	NaN	NaN	Q15771;E9PRF7;E	Q15771;E9PRF7;E	Ras-related protei	RAB30
23.7856	24.1368	24.5855	24.0161	24.1119	24.7162	24.2401	25.2192	25.147	24.4036	24.3867	24.7673	Q13636;J3QR51	Q13636;J3QR51	Ras-related protei	RAB31
25.9723	25.0431	25.6815	25.8534	24.8161	25.9452	25.896	NaN	24.5467	26.2132	25.5534	24.9787	Q13637	Q13637	Ras-related protei	RAB32
NaN	NaN	NaN	NaN	NaN	21.7357	NaN	NaN	NaN	NaN	NaN	NaN	Q14088	Q14088	Ras-related protei	RAB33A
22.9104	23.7403	23.0828	NaN	22.1847	NaN	NaN	22.9105	NaN	22.1245	NaN	NaN	P0DI83	P0DI83	Ras-related protei	RAB34
27.6365	27.7413	27.9932	27.6167	27.7216	28.1207	27.9197	27.9469	27.4884	28.5468	28.2322	27.826	Q9BZG1;A8MYQ9	Q9BZG1;A8MYQ9	Ras-related protei	RAB34
29.5213	29.446	30.2856	29.5509	29.5992	30.4671	30.1072	29.6859	29.6375	30.834	30.4234	30.1788	Q15286;F5H157;C	Q15286;F5H157;C	Ras-related protei	RAB35
21.5283	NaN	22.1112	21.4658	NaN	NaN	NaN	NaN	NaN	23.045	22.3779	NaN	P57729;H0YEA4;H	P57729;H0YEA4	Ras-related protei	RAB38
26.1032	26.1482	26.5623	26.4966	26.205	27.0871	NaN	25.1104	26.0668	27.2162	26.7907	26.196	Q14964	Q14964	Ras-related protei	RAB39A
26.2467	25.863	25.7133	26.2154	25.6548	25.6351	26.0844	26.2759	25.4046	25.6085	25.9374	25.3254	Q15042;Q15042-3	Q15042;Q15042-3	Rab3 GTPase-acti	RAB3GAP1
26.623	26.1057	26.389	26.5486	26.2	26.1864	26.4182	26.9175	26.3074	26.5073	26.4646	25.8003	Q9H2M9;Q9H2M9	Q9H2M9	Rab3 GTPase-acti	RAB3GAP2
23.8919	NaN	23.9447	23.1346	23.2545	NaN	NaN	NaN	NaN	23.461	NaN	NaN	Q86YS6;Q86YS6-2	Q86YS6;Q86YS6-2	Ras-related protei	RAB43
22.4097	22.2614	22.4705	22.4835	22.4231	22.8102	NaN	NaN	22.8717	22.9254	22.9658	22.9467	P20338;A0A087W	P20338	Ras-related protei	RAB4A
27.0556	27.3233	27.0695	26.7421	27.3261	27.0141	28.1372	27.7698	28.9146	27.9335	27.3724	28.217	P20339;P20339-2	P20339;P20339-2	Ras-related protei	RAB5A
25.5979	25.3865	25.9315	25.0733	25.6343	25.7455	26.0166	25.7617	26.6341	26.1176	25.6424	25.7607	P61020;P61020-2	P61020;P61020-2	Ras-related protei	RAB5B
29.7002	29.6101	29.7488	29.5426	29.7574	29.7495	30.1725	29.7086	30.2342	30.0176	29.8217	30.151	P51148;P51148-2	P51148;P51148-2	Ras-related protei	RAB5C
30.0129	29.6872	30.1752	29.9693	29.6475	30.1616	30.8409	30.5085	30.154	30.6217	30.3265	29.8575	P20340-2;P20340	P20340-2;P20340	Ras-related protei	RAB6A
30.028	29.4676	29.6366	29.543	28.8703	29.3709	30.5766	29.6756	29.7645	30.1783	29.8131	29.8418	P51149;C9J592;C9	P51149;C9J592;C9	Ras-related protei	RAB7A
29.0983	28.838	29.2823	28.7971	28.9696	29.0793	29.6559	28.9254	28.936	29.577	29.0334	28.6314	P61006;P61006-2	P61006;P61006-2	Ras-related protei	RAB8A
28.2565	28.4627	28.5342	28.2453	28.4737	28.6897	28.875	27.5268	28.021	28.8601	28.555	28.7367	Q92930;H0YNE9	Q92930;H0YNE9	Ras-related protei	RAB8B
25.2048	24.7201	24.6445	24.4109	24.2466	24.0728	24.5934	24.5778	25.2968	24.6315	24.4036	24.8259	P51151;Q9NP90	P51151	Ras-related protei	RAB9A
25.55	25.0711	25.2545	25.5037	NaN	25.0065	NaN	24.8969	25.0517	24.7489	NaN	24.6463	Q9UI14;M0R3D4;	Q9UI14;M0R3D4;	Prenylated Rab ac	RABAC1
23.7588	23.9584	23.9808	23.1242	23.3868	NaN	23.8067	24.2679	23.9187	23.6843	24.0323	23.9371	Q15276;Q15276-2	Q15276;Q15276-2	Rab GTPase-bindi	RABEP1
24.2893	23.1368	24.0849	23.9458	23.3476	23.6851	23.8391	NaN	NaN	24.1657	23.8761	23.0798	Q9Y3P9;Q9Y3P9-2	Q9Y3P9;Q9Y3P9-2	Rab GTPase-activa	RABGAP1
25.0122	25.8992	24.2742	25.1088	25.5022	24.7595	25.5846	NaN	24.2612	NaN	25.3861	24.3969	Q9UJ41;Q96MP8;	Q9UJ41	Rab5 GDP/GTP ex	RABGEF1
25.9114	25.3265	25.3397	25.4656	25.1655	25.2204	25.0854	NaN	25.3371	25.2996	24.991	24.9263	Q92696;H0YLH3;H	Q92696;H0YLH3	Geranylgeranyl tra	RABGGTA
24.753	24.5394	24.766	24.3865	24.1499	24.551	24.1781	NaN	NaN	NaN	24.5089	NaN	P53611;Q5T4U8	P53611;Q5T4U8	Geranylgeranyl tra	RABGGTB
22.9309	23.3752	23.4655	22.5877	23.4067	NaN	NaN	NaN	NaN	23.4855	NaN	23.5426	P47224	P47224	Guanine nucleotic	RABIF
24.8958	24.3621	23.7373	23.9897	24.4605	24.3501	23.9453	24.2703	NaN	23.842	NaN	NaN	Q5HYI8;F8WF50;F	Q5HYI8;F8WF50;F	Rab-like protein 3	RABL3
NaN	NaN	NaN	20.5909	NaN	NaN	NaN	NaN	NaN	NaN	NaN	NaN	Q3YEC7;F2Z2T0;A	Q3YEC7;F2Z2T0;A	Rab-like protein 6	RABL6
29.8523	29.7359	30.3146	29.7988	29.8159	30.3901	29.9956	29.2903	30.3016	30.5495	30.694	30.7458	P63000;P63000-2	P63000;P63000-2	Ras-related C3 bo	RAC1
28.5242	28.1157	28.4166	28.2515	28.4198	28.7668	27.9763	27.6749	28.1062	28.9553	28.7196	28.4474	P15153;B1AH77;B	P15153;B1AH77;B	Ras-related C3 bo	RAC2
NaN	NaN	NaN	NaN	NaN	NaN	NaN	NaN	NaN	NaN	22.8365	NaN	P60763;J3KSC4;J3	P60763;J3KSC4;J3	Ras-related C3 bo	RAC3
29.3311	29.4745	29.9879	29.2065	29.2159	29.8192	31.5711	30.8613	30.8553	29.9673	29.2886	29.7594	Q9H0H5;F8VRD2;	Q9H0H5;F8VRD2	Rac GTPase-activa	RACGAP1
NaN	NaN	NaN	NaN	NaN	NaN	NaN	NaN	NaN	18.6201	NaN	NaN	O60671;O60671-2	O60671;O60671-2	Cell cycle checkpo	RAD1
24.3705	23.7538	23.9704	24.1309	23.8242	24.4061	NaN	24.3513	NaN	23.0049	24.1137	24.021	O60216;E5RI01;E5	O60216	Double-strand-bre	RAD21
24.5231	24.3426	24.2165	23.9423	23.3561	24.1948	24.6842	NaN	NaN	24.5559	24.3683	NaN	P54725;P54725-2	P54725;P54725-2	UV excision repair	RAD23A
27.4246	27.4118	27.5066	26.9164	26.5986	27.3641	28.0251	26.4756	26.1628	27.4604	27.0674	26.7163	P54727;Q5W0S5;	P54727;Q5W0S5;	UV excision repair	RAD23B
28.5788	26.9466	27.9181	29.3054	28.8556	28.5673	22.9755	22.4644	23.287	26.8571	26.4312	25.0237	Q92878;Q92878-2	Q92878;Q92878-2	DNA repair protei	RAD50
NaN	NaN	NaN	22.6741	NaN	NaN	NaN	NaN	NaN	NaN	NaN	NaN	Q09MP3	Q09MP3	RAD51-associated	RAD51AP2
20.5491	NaN	NaN	NaN	NaN	NaN	NaN	NaN	NaN	NaN	NaN	NaN	O75771;K7EJ58;K7	O75771;K7EJ58;K7	DNA repair protei	RAD51D;hCG_203
27.4691	27.4992	27.1443	27.2047	27.4308	27.1552	27.0527	27.5818	26.5982	27.6772	28.2223	27.4443	P78406;E9PQ57;E	P78406;E9PQ57	mRNA export fact	RAE1

26.2823	25.4765	26.1333	26.3692	25.9873	26.0376	26.18	26.0411	26.9584	27.1942	26.7953	27.1887	P04049;P04049-2	P04049;P04049-2	RAF proto-oncoge	RAF1
NaN	NaN	NaN	NaN	NaN	NaN	NaN	29.1689	NaN	NaN	NaN	NaN	Q7Z5J4;A8MXE8;(	Q7Z5J4;A8MXE8;(	Retinoic acid-indu	RAI1
26.2413	26.2069	25.7135	25.8648	25.8756	25.6815	26.1579	26.3155	26.2879	25.4977	25.486	26.1085	Q9P0K7-4;Q9P0K7	Q9P0K7-4;Q9P0K7	Ankycorbin	RAI14
28.8728	29.1617	29.2115	28.9102	28.8555	29.3152	29.4105	28.332	28.789	29.9094	29.0697	29.1013	P11233;H7C3P7;C	P11233;H7C3P7	Ras-related protei	RALA
26.2548	27.2266	27.0256	26.4223	26.9188	27.2454	27.6474	27.7044	27.0873	27.9924	27.1532	26.9986	P11234;P11234-2	P11234;P11234-2	Ras-related protei	RALB
24.7079	23.9754	24.1307	24.6171	23.434	24.4605	23.9457	24.3166	24.1551	24.2935	24.0537	24.6073	Q15311	Q15311	RalA-binding prot	RALBP1
22.4782	NaN	NaN	22.4388	NaN	21.7942	NaN	NaN	NaN	NaN	NaN	NaN	Q6GYQ0;Q6GYQ0	Q6GYQ0;Q6GYQ0	Ral GTPase-activa	RALGAPA1
23.6199	23.7024	23.9733	23.7427	23.9455	23.7454	NaN	NaN	NaN	23.6338	23.381	NaN	Q86X10;Q86X10-3	Q86X10;Q86X10-3	Ral GTPase-activa	RALGAPB
29.3346	28.943	28.9395	29.5169	29.296	29.1406	27.3714	28.177	27.2892	28.195	28.7294	28.0935	Q9UKM9;Q9UKM	Q9UKM9;Q9UKM	RNA-binding prote	RALY
30.8677	30.9282	30.7033	30.5472	31.1903	30.9246	31.1781	30.9311	30.895	32.7198	33.3391	31.5526	P62826;B5MDF5;	P62826;B5MDF5;	GTP-binding nucle	RAN
28.8515	28.4961	28.3479	28.4167	28.475	28.2407	28.691	28.9247	28.9618	28.2681	28.5511	29.1382	P43487;P43487-2	P43487;P43487-2	Ran-specific GTPa	RANBP1
22.9543	NaN	NaN	23.0689	NaN	NaN	NaN	NaN	NaN	23.2647	23.5594	NaN	Q6VN20;A0A0D9S	Q6VN20;A0A0D9S	Ran-binding prote	RANBP10
26.4749	25.3235	25.2674	26.0523	25.5115	24.8043	23.736	25.7017	25.2629	24.993	25.0434	25.4383	P49792;P0DJD0;P	P49792	E3 SUMO-protein	RANBP2
23.3337	23.6174	22.9835	23.072	23.8953	23.0891	NaN	23.207	NaN	23.3842	23.3406	23.6465	Q9H6Z4;Q9H6Z4-	Q9H6Z4;Q9H6Z4-	Ran-binding prote	RANBP3
24.8141	24.5433	24.57	25.014	25.1107	24.8399	NaN	24.3209	24.8976	24.3808	24.7411	24.7536	Q96S59;Q96S59-2	Q96S59;Q96S59-2	Ran-binding prote	RANBP9
27.7954	27.4713	27.8398	27.947	27.5849	27.9085	27.9259	27.5686	27.9498	28.1168	28.5026	28.5403	P46060;H0Y4Q3;E	P46060	Ran GTPase-activa	RANGAP1
22.3258	NaN	NaN	NaN	NaN	NaN	NaN	NaN	NaN	NaN	NaN	NaN	Q9HD47;Q9HD47	Q9HD47;Q9HD47	Ran guanine nucle	RANGRF
25.7261	25.1296	25.4712	25.2311	24.0139	NaN	24.857	NaN	NaN	26.116	24.6076	23.8587	P62834;A0A075B6	P62834;A0A075B6	Ras-related protei	RAP1A
30.2618	29.8846	30.3678	29.9753	29.6702	30.0775	30.5525	29.3244	29.6939	30.3915	29.7704	29.8851	P61224;P61224-3	P61224;P61224-3	Ras-related protei	RAP1B
24.8272	24.5034	24.7032	24.8052	24.3715	24.463	25.2665	NaN	24.7257	25.1373	24.7709	24.8083	P52306;P52306-2	P52306;P52306-2	Rap1 GTPase-GDF	RAP1GDS1
27.7453	27.4605	27.7475	27.7111	27.3521	28.1132	27.9801	27.3603	28.1426	28.3811	27.7407	28.1122	P61225	P61225	Ras-related protei	RAP2B
22.6104	23.4832	24.3939	23.3167	23.808	NaN	22.0066	NaN	NaN	22.1776	23.1519	23.6215	Q9Y3L5;A0A087X	Q9Y3L5;A0A087X	Ras-related protei	RAP2C
25.1086	23.8478	24.0926	24.5707	23.8715	NaN	23.068	24.295	24.0274	24.2347	24.2653	24.104	Q8TEU7;E9PCH4;	Q8TEU7;E9PCH4;	Rap guanine nucle	RAPGEF6
24.3596	25.1519	25.4722	24.2022	24.6905	24.5039	23.7737	NaN	24.7301	24.2254	24.2064	23.7657	Q70E73;C9K0J5;C	Q70E73;C9K0J5;C	Ras-associated an	RAPH1
29.9208	29.3128	29.5945	30.0453	29.5622	29.7386	29.0934	29.3215	29.5143	29.5132	29.5697	29.5625	P54136;P54136-2	P54136;P54136-2	Arginine--tRNA lig	RARS
26.1822	25.3162	25.7407	26.0841	25.1107	25.7517	25.3233	24.6426	25.2965	25.6271	25.3869	25.1135	P20936;E9PGC0;P	P20936;E9PGC0;P	Ras GTPase-activa	RASA1
24.4271	24.2227	25.1057	24.7279	25.6863	25.0143	NaN	NaN	NaN	25.3279	25.3125	25.1954	Q15283;A0A0A0N	Q15283;A0A0A0N	Ras GTPase-activa	RASA2
25.2034	24.9228	26.3118	25.6813	25.4239	26.0107	25.8519	24.4967	25.5368	27.8256	27.1418	26.5191	Q14644;Q14644-2	Q14644;Q14644-2	Ras GTPase-activa	RASA3
24.094	24.7048	24.4974	23.9558	24.5628	24.6176	25.4059	NaN	24.4526	25.3832	25.1283	25.306	Q9UJF2-2;Q9UJF2	Q9UJF2-2;Q9UJF2	Ras GTPase-activa	RASAL2
23.5238	NaN	23.2013	22.7387	23.4092	23.0079	NaN	NaN	NaN	23.0179	NaN	NaN	P50749;P50749-2	P50749;P50749-2	Ras association dc	RASSF2
NaN	22.1325	NaN	NaN	NaN	NaN	NaN	NaN	NaN	NaN	NaN	NaN	Q02833;H0YEI0;A	Q02833;H0YEI0;A	Ras association dc	RASSF7
23.9417	NaN	23.3952	23.6082	23.2584	NaN	NaN	NaN	NaN	23.495	23.4519	NaN	Q8IY67;Q8IY67-2;	Q8IY67;Q8IY67-2;	Ribonucleoprotein	RAVER1
25.3281	23.1668	24.4841	25.1336	23.6782	24.1441	NaN	NaN	NaN	24.6509	24.3122	NaN	P06400	P06400	Retinoblastoma-a	RB1
25.2288	NaN	25.2984	NaN	NaN	NaN	NaN	NaN	NaN	NaN	NaN	NaN	Q8TDY2;Q8TDY2-	Q8TDY2;Q8TDY2-	RB1-inducible coil	RB1CC1
25.3821	25.7724	25.3176	25.1985	25.5885	25.5526	24.5032	24.618	NaN	24.9282	25.48	25.2974	Q09028;Q09028-3	Q09028;Q09028-3	Histone-binding p	RBBP4
23.2734	NaN	23.261	23.6307	NaN	23.0722	NaN	NaN	NaN	23.5258	23.5292	NaN	Q15291;Q15291-2	Q15291;Q15291-2	Retinoblastoma-b	RBBP5
NaN	NaN	NaN	21.3429	NaN	NaN	NaN	NaN	NaN	NaN	NaN	NaN	Q7Z6E9;Q7Z6E9-2	Q7Z6E9;Q7Z6E9-2	E3 ubiquitin-prote	RBBP6
28.367	28.1128	28.0095	28.2843	28.2148	28.2208	27.3592	27.9059	27.6055	28.7763	28.9317	28.7217	Q16576;E9PC52;C	Q16576;E9PC52;C	Histone-binding p	RBBP7
24.2638	24.4591	24.3981	23.9379	24.351	24.0323	25.1245	NaN	NaN	25.0216	25.1937	24.2576	O75884;A0A087W	O75884;A0A087W	Putative hydrolase	RBBP9
23.8587	23.7897	23.7933	23.8928	23.7089	23.6457	23.5814	NaN	23.586	23.8671	NaN	23.6739	Q9BYM8;Q9BYM8	Q9BYM8;Q9BYM8	RanBP-type and C	RBCK1
21.6375	NaN	NaN	NaN	NaN	NaN	NaN	NaN	NaN	NaN	NaN	NaN	O43251;O43251-6	O43251;O43251-6	RNA binding prote	RBFOX2
NaN	22.9933	22.0153	NaN	22.0653	22.3797	NaN	NaN	NaN	NaN	NaN	NaN	Q9H477-2;Q9H47	Q9H477-2;Q9H47	Ribokinase	RBKS
NaN	22.6727	NaN	23.0204	NaN	23.4201	NaN	NaN	NaN	NaN	NaN	NaN	P98175;P98175-2	P98175;P98175-2	RNA-binding prote	RBM10
25.1396	24.7386	24.8931	25.2548	24.7677	25.0936	24.1794	NaN	24.2425	25.0385	24.998	25.467	Q9NTZ6	Q9NTZ6	RNA-binding prote	RBM12
21.9337	NaN	22.1225	22.9733	22.2002	22.2786	NaN	NaN	NaN	NaN	22.4499	NaN	Q8IXT5;B9ZVT1	Q8IXT5;B9ZVT1	RNA-binding prote	RBM12B
26.8316	26.7841	26.9556	27.6829	27.2087	27.3873	25.6845	24.0223	26.5767	27.1028	28.0894	27.0817	Q96PK6;Q96PK6-	Q96PK6	RNA-binding prote	RBM14
23.3661	24.1282	23.2587	23.858	23.6625	23.6402	NaN	NaN	NaN	NaN	23.2271	23.5886	Q96PK6-5	Q96PK6-5	RNA-binding prote	RBM14
25.1904	25.1649	25.5941	25.5701	25.9234	25.4307	NaN	24.657	24.8631	25.6824	25.7764	26.3525	Q96T37-3;Q96T37	Q96T37-3;Q96T37	Putative RNA-binc	RBM15
23.9308	24.1744	23.5766	23.7762	24.1028	24.0091	NaN	NaN	NaN	NaN	23.5387	NaN	Q96I25;Q5W011;(	Q96I25	Splicing factor 45	RBM17
24.1027	23.7898	23.6129	23.7766	23.7588	NaN	NaN	NaN	NaN	23.8147	23.8375	23.9542	Q9NW64-2;Q9NW	Q9NW64-2;Q9NW	Pre-mRNA-splicing	RBM22
27.539	26.3187	26.7657	27.5932	26.4563	26.8135	25.6974	25.281	24.82	26.8947	26.8724	26.1657	P49756;P49756-2	P49756	RNA-binding prote	RBM25
NaN	NaN	NaN	NaN	NaN	NaN	NaN	NaN	NaN	NaN	28.1716	NaN	Q5T8P6;Q5T8P6-3	Q5T8P6;Q5T8P6-3	RNA-binding prote	RBM26
24.4596	NaN	22.6561	23.7698	23.4865	NaN	NaN	NaN	NaN	NaN	22.4649	NaN	Q9NW13;Q9NW1	Q9NW13;Q9NW1	RNA-binding prote	RBM28
24.6676	26.2785	26.0815	24.9806	25.8025	25.7725	25.2292	26.758	25.5972	24.7568	25.9054	26.1281	P98179	P98179	RNA-binding prote	RBM3
NaN	NaN	NaN	NaN	NaN	NaN	NaN	NaN	19.9923	NaN	NaN	NaN	P42696;A2A2V2;C	P42696;A2A2V2	RNA-binding prote	RBM34
28.069	26.2495	26.9079	27.9985	26.5518	26.9828	25.0862	26.2847	25.9491	27.2552	27.3711	27.4223	Q14498;Q14498-2	Q14498;Q14498-2	RNA-binding prote	RBM39
22.4414	NaN	NaN	22.4562	NaN	NaN	NaN	NaN	NaN	NaN	NaN	NaN	Q9BWF3;E9PB51;	Q9BWF3;E9PB51;	RNA-binding prote	RBM4;RBM4B
NaN	22.6558	NaN	NaN	NaN	NaN	NaN	NaN	NaN	NaN	NaN	22.9425	Q9BTD8;K7EQ03;I	Q9BTD8;K7EQ03;I	RNA-binding prote	RBM42
21.013	NaN	NaN	NaN	NaN	NaN	NaN	NaN	NaN	NaN	NaN	NaN	P52756;C9JFQ5;P	P52756;C9JFQ5;P	RNA-binding prote	RBM5
22.9026	NaN	22.4212	22.6431	NaN	NaN	NaN	NaN	NaN	22.1355	NaN	NaN	Q9Y580;J3KPD3;G	Q9Y580;J3KPD3;G	RNA-binding prote	RBM7

27.0338	26.6638	26.4234	26.9905	26.5026	27.1688	26.2808	26.7681	26.1364	27.1077	27.2092	27.6419	Q9Y5S9;Q9Y5S9-2	Q9Y5S9;Q9Y5S9-2	RNA-binding prot	RBM8A
NaN	NaN	NaN	21.9286	NaN	NaN	NaN	NaN	NaN	NaN	NaN	NaN	P29558;E7EPF2;E7	P29558;E7EPF2;E7	RNA-binding moti	RBMS1;RBMS3
23.5768	23.4319	23.3673	NaN	NaN	NaN	NaN	NaN	NaN	NaN	NaN	NaN	Q15434;F8W1T6;I	Q15434;F8W1T6	RNA-binding moti	RBMS2
28.118	28.1296	28.2111	28.2979	28.044	28.4131	26.8863	27.975	27.2398	28.0025	28.4618	28.3196	P38159;H3BT71;P	P38159;H3BT71;P	RNA-binding moti	RBMX
23.7852	NaN	25.9027	24.3537	25.6888	NaN	NaN	NaN	NaN	NaN	25.2891	NaN	Q96E39	Q96E39	RNA binding moti	RBMXL1
22.7577	26.2784	23.7484	22.9767	25.9316	NaN	26.5982	27.3316	25.4706	NaN	NaN	NaN	P02753;Q5VY30;A	P02753;Q5VY30;A	Retinol-binding pr	RBP4
24.7241	24.9977	24.4154	24.3147	24.6398	24.8433	25.2743	25.2893	NaN	25.0927	25.3591	25.347	P62877	P62877	E3 ubiquitin-prote	RBX1
27.5568	25.693	25.522	27.2974	25.7869	25.261	25.5672	24.8351	24.7835	26.3503	26.2094	25.4009	P18754;P18754-2	P18754;P18754-2	Regulator of chro	RCC1
27.0709	26.7874	26.7512	26.6865	27.0475	26.8554	26.2985	26.5618	26.6296	27.3319	27.5779	27.1977	Q9P258	Q9P258	Protein RCC2	RCC2
22.7892	NaN	NaN	22.8542	NaN	NaN	NaN	NaN	NaN	24.3896	24.0178	23.4955	A6NED2;G3V2I3	A6NED2;G3V2I3	RCC1 domain-con	RCCD1
23.6689	NaN	22.7688	23.3426	NaN	NaN	NaN	NaN	NaN	22.7212	22.7548	NaN	Q9Y2P8;Q5VYW8	Q9Y2P8	RNA 3-terminal pl	RCL1
26.1674	27.5421	26.5736	26.3858	27.2193	26.6258	26.4516	27.4342	26.7847	26.6845	26.1275	25.2724	Q15293;Q15293-2	Q15293;Q15293-2	Reticulocalbin-1	RCN1
25.52	25.9701	25.6414	25.44	25.197	25.5361	25.9005	26.3671	25.854	25.3302	25.0406	25.2978	Q14257;Q14257-2	Q14257;Q14257-2	Reticulocalbin-2	RCN2
21.867	NaN	22.6113	21.5506	23.1972	NaN	NaN	NaN	NaN	NaN	22.7591	22.6779	Q9UKL0;J3KN32;C	Q9UKL0;J3KN32	REST corepressor	RCOR1
27.7013	27.0997	27.4186	27.7884	27.2343	27.6304	27.6809	26.9423	27.4937	28.2063	27.429	26.7709	Q8TC12;Q8TC12-2	Q8TC12;Q8TC12-2	Retinol dehydroge	RDH11
23.0292	NaN	23.3052	23.0125	NaN	NaN	NaN	NaN	NaN	23.1576	NaN	NaN	Q9HBH5;C9J2C7	Q9HBH5	Retinol dehydroge	RDH14
30.7338	31.2533	32.1171	31.5101	31.5832	32.4107	30.4304	28.6792	28.8454	32.5914	31.5645	30.7089	P35241;P35241-5	P35241;P35241-5	Radixin	RDX
25.7133	24.0281	24.6641	25.5725	24.4042	24.5356	NaN	NaN	NaN	25.4541	25.3387	24.265	P46063;F8WA66;I	P46063	ATP-dependent D	RECQL
NaN	NaN	22.2214	21.4851	NaN	NaN	NaN	NaN	NaN	NaN	NaN	NaN	Q6NUK4;A0A0U1I	Q6NUK4;A0A0U1I	Receptor expressi	REEP3
21.7263	NaN	22.0015	NaN	NaN	NaN	NaN	NaN	NaN	NaN	NaN	NaN	Q9H6H4;E5RGS2;	Q9H6H4;E5RGS2;	Receptor expressi	REEP4
26.2026	24.3071	25.1508	25.627	24.2658	24.8328	25.5088	NaN	NaN	26.6043	24.2449	23.9319	Q00765;E2QRG8;I	Q00765	Receptor expressi	REEP5
24.1809	23.0251	23.2954	23.3005	23.0285	NaN	NaN	NaN	NaN	22.7186	22.5626	NaN	Q04864;Q04864-2	Q04864;Q04864-2	Proto-oncogene c	REL
26.8034	26.4857	26.7336	26.9539	26.8433	26.8482	26.4046	26.7299	26.6803	26.6878	26.997	26.8199	Q04206;Q2TAM5;	Q04206;Q2TAM5;	Transcription fact	RELA
NaN	NaN	NaN	20.321	NaN	NaN	NaN	NaN	NaN	NaN	NaN	NaN	Q01201;D6R992;I	Q01201;D6R992;I	Transcription fact	RELB
25.5499	26.4212	26.6971	25.9482	26.4883	26.7579	25.4106	25.1505	NaN	26.0626	25.9107	26.6799	Q8IUW5;D6RBN9	Q8IUW5;D6RBN9	RELT-like protein	RELL1
NaN	NaN	NaN	21.6797	NaN	NaN	NaN	NaN	NaN	NaN	NaN	NaN	Q9BWE0;Q9BWE0	Q9BWE0;Q9BWE0	Replication initiat	REPIN1
24.8846	25.5512	25.0111	24.7211	25.5355	25.1137	25.1836	25.9542	25.3445	25.5765	25.4153	25.7033	Q96D71;Q96D71-	Q96D71;Q96D71-	RalBP1-associated	REPS1
NaN	22.4596	22.2254	NaN	NaN	NaN	NaN	NaN	NaN	NaN	22.575	NaN	Q8NFH8;Q8NFH8	Q8NFH8;Q8NFH8	RalBP1-associated	REPS2
25.1227	NaN	24.256	25.1919	24.1729	24.2351	24.2105	NaN	NaN	24.5736	23.9607	NaN	O15258;Q9P0H9;	O15258;Q9P0H9;	Protein RER1	RER1
23.2707	NaN	22.9447	23.0611	22.9235	NaN	NaN	NaN	NaN	23.3672	22.8989	NaN	Q6NUM9;Q6NUM	Q6NUM9;Q6NUM	All-trans-retinol 1	RETSAT
NaN	NaN	NaN	NaN	NaN	NaN	NaN	NaN	NaN	NaN	NaN	21.8212	Q9Y3B8;H0YGR4;I	Q9Y3B8;H0YGR4;I	Oligoribonuclease	REXO2
25.853	27.0325	26.3102	25.7214	26.8574	26.4021	26.5646	26.3742	26.7791	26.3459	26.6846	27.0195	Q9Y3B8-3;H0YG5	Q9Y3B8-3;H0YG5	Oligoribonuclease	REXO2
21.7525	NaN	NaN	NaN	NaN	NaN	NaN	NaN	NaN	NaN	NaN	NaN	P35251;P35251-2	P35251;P35251-2	Replication factor	RFC1
27.6568	26.722	27.021	27.6978	26.772	26.5822	24.6513	24.9588	NaN	25.9707	26.1212	26.1759	P35250;P35250-2	P35250;P35250-2	Replication factor	RFC2
27.2777	25.9413	26.5229	27.7295	26.7363	26.9063	25.7114	26.0019	23.9858	26.9995	26.8069	26.5563	P40938;P40938-2	P40938;P40938-2	Replication factor	RFC3
28.2617	27.7128	27.7343	28.2496	27.7988	27.6435	26.159	26.7773	24.8961	26.8492	27.1732	26.1038	P35249;C9JZI1;P3	P35249;C9JZI1;P3	Replication factor	RFC4
28.9293	28.0371	28.5537	29.1994	28.3443	28.4562	27.0117	27.0784	25.9662	27.8386	27.7199	27.8188	P40937;P40937-2	P40937;P40937-2	Replication factor	RFC5
NaN	22.0977	NaN	NaN	NaN	NaN	NaN	NaN	NaN	NaN	NaN	NaN	Q969G6;H7C4G0	Q969G6;H7C4G0	Riboflavin kinase	RFK
29.8883	29.6049	30.2708	29.9833	29.6199	30.3344	30.4851	29.739	30.1251	31.4427	30.8911	30.8072	Q14699;G3XAJ6;C	Q14699;G3XAJ6;C	Raftlin	RFTN1
23.5902	23.1351	24.1362	23.4925	23.4953	23.458	23.5293	NaN	NaN	23.5883	23.199	NaN	O15211;O15211-2	O15211;O15211-2	Ral guanine nucle	RGL2
NaN	22.2526	NaN	NaN	NaN	NaN	NaN	NaN	NaN	NaN	NaN	NaN	A6NKT7;J3KNE0	A6NKT7;J3KNE0	RanBP2-like and G	RGPD3
26.4318	26.4424	26.6779	26.7665	26.5825	26.6339	26.0378	24.9436	25.8889	28.1169	26.7706	26.8831	O43665-3;O43665	O43665-3;O43665	Regulator of G-pr	RGS10
NaN	NaN	21.6517	NaN	NaN	22.2794	NaN	NaN	NaN	21.9853	NaN	NaN	O14924;O14924-5	O14924;O14924-5	Regulator of G-pr	RGS12
22.9216	NaN	23.2673	22.3691	23.1717	23.1445	23.4423	NaN	23.7157	23.8386	22.8048	23.5739	Q9UGC6	Q9UGC6	Regulator of G-pr	RGS17
24.7414	24.7608	25.8003	25.0519	24.928	25.6115	24.9291	24.3388	25.0687	25.6308	25.1056	25.3441	P49795	P49795	Regulator of G-pr	RGS19
NaN	NaN	21.6333	NaN	NaN	21.8919	NaN	NaN	NaN	NaN	NaN	NaN	O76081;O76081-5	O76081;O76081-5	Regulator of G-pr	RGS20
22.6998	22.4009	23.9183	22.5999	22.9247	23.6078	NaN	NaN	NaN	24.6958	22.9176	22.2143	Q96CC6;A2IDA2	Q96CC6	Inactive rhomboic	RHBDF1
24.5511	25.7478	26.5346	25.6072	25.8328	26.4956	24.8291	NaN	25.3256	26.7488	25.7578	25.4385	Q6PJF5;Q6PJF5-2;	Q6PJF5;Q6PJF5-2	Inactive rhomboic	RHBDF2
28.6295	28.1489	28.6561	28.6066	28.2174	28.8042	28.3675	27.2868	28.0256	28.6393	28.6175	28.5181	Q15382;C9J931;C	Q15382;C9J931	GTP-binding prote	RHEB
30.1386	29.294	29.7524	29.6628	29.2024	29.8193	30.2673	28.422	29.4733	30.2542	29.8648	29.8425	P61586;C9JX21;C9	P61586;C9JX21;C9	Transforming prot	RHOA
24.4252	25.0437	25.7499	24.678	25.0929	NaN	NaN	NaN	NaN	26.4928	25.7848	26.3141	P62745	P62745	Rho-related GTP-l	RHOB
22.6859	NaN	NaN	NaN	NaN	NaN	NaN	NaN	NaN	NaN	NaN	NaN	O94955	O94955	Rho-related BTB d	RHOBTB3
27.4784	27.488	28.2968	27.1242	28.1048	28.2458	26.8622	26.5295	28.238	27.4788	27.7579	28.051	P08134;Q5JR08;E9	P08134;Q5JR08;E9	Rho-related GTP-l	RHOC
26.4375	25.4134	26.8246	26.8982	25.5193	26.9375	25.7641	NaN	25.2101	27.4125	26.4164	26.0432	Q9HBH0;Q9HBH0	Q9HBH0;Q9HBH0	Rho-related GTP-l	RHOF
27.9292	27.9573	28.4158	27.8571	27.9246	28.6227	28.0177	27.3569	27.843	28.3061	27.8197	28.1447	P84095	P84095	Rho-related GTP-l	RHOG
NaN	NaN	21.7483	21.9591	21.9528	NaN	NaN	22.4292	NaN	NaN	NaN	NaN	Q8IXI1;H3BST5;I3	Q8IXI1;H3BST5;I3	Mitochondrial Rho	RHOT2
26.7797	26.0181	26.7027	26.869	26.351	26.6851	26.3046	25.7581	26.4681	26.8007	26.7354	27.0404	Q9NPQ8;Q9NPQ8	Q9NPQ8;Q9NPQ8	Synembryn-A	RIC8A
26.649	26.293	26.7962	26.338	26.645	26.7997	26.477	26.658	27.3042	26.6966	26.3111	27.3502	Q6R327;Q6R327-3	Q6R327;Q6R327-3	Rapamycin-insens	RICTOR
23.376	22.219	22.7321	23.5647	22.337	22.5647	NaN	NaN	NaN	22.728	22.645	NaN	Q5UIP0;Q5UIP0-2	Q5UIP0;Q5UIP0-2	Telomere-associat	RIF1
25.0003	24.0638	24.4487	24.9661	24.8533	24.8769	24.8611	NaN	24.9322	25.0119	25.2072	25.1864	Q13671;Q13671-2	Q13671;Q13671-2	Ras and Rab inter	RIN1

23.0402	NaN	NaN	22.4946	NaN	NaN	NaN	NaN	NaN	NaN	NaN	NaN	Q8WYP3;Q8WYP3	Q8WYP3;Q8WYP3	Ras and Rab intera	RIN2
NaN	23.6074	23.9209	24.0342	23.9065	NaN	NaN	NaN	NaN	24.0875	23.5774	NaN	Q06587;Q06587-2	Q06587;Q06587-2	E3 ubiquitin-prote	RING1
21.6936	NaN	21.897	22.2742	NaN	NaN	NaN	22.2851	NaN	22.2662	21.9297	NaN	Q6NUQ1;C9J5S3	Q6NUQ1	RAD50-interacting	RINT1
25.3776	22.5659	23.6748	24.734	22.382	22.8803	NaN	NaN	NaN	22.9497	22.3026	NaN	Q9BRS2	Q9BRS2	Serine/threonine-	RIOK1
24.1615	23.1476	23.3655	24.2866	23.5784	23.2313	NaN	NaN	NaN	23.7876	23.6523	23.7792	Q9BVS4;Q9BVS4-2	Q9BVS4;Q9BVS4-2	Serine/threonine-	RIOK2
24.2093	23.7772	23.8138	23.854	23.5189	23.6003	23.8854	NaN	NaN	24.0156	23.9557	NaN	Q13546;Q13546-2	Q13546;Q13546-2	Receptor-interact	RIPK1
22.8124	23.6579	23.6798	23.0699	23.9807	23.5267	23.1604	NaN	23.3153	23.9637	23.2221	24.1939	Q92963;Q92963-3	Q92963;Q92963-3	GTP-binding prote	RIT1
22.6946	23.5688	22.9011	22.6756	23.3794	22.5354	NaN	22.8737	NaN	22.7212	23.3488	NaN	Q96DB5;Q96DB5-	Q96DB5;Q96DB5-	Regulator of micro	RMDN1
NaN	NaN	NaN	NaN	NaN	NaN	NaN	NaN	NaN	24.4941	NaN	NaN	Q9H871;Q96G75;	Q9H871	Protein RMD5 hom	RMND5A
NaN	NaN	NaN	NaN	NaN	NaN	23.0886	NaN	NaN	NaN	NaN	NaN	P34096	P34096	Ribonuclease 4	RNASE4
23.296	NaN	NaN	22.7534	NaN	NaN	NaN	NaN	NaN	23.2651	23.7054	23.8187	O75792	O75792	Ribonuclease H2 s	RNASEH2A
22.655	NaN	NaN	22.7798	NaN	NaN	NaN	NaN	NaN	NaN	22.6259	NaN	Q5TBB1;A0A087W	Q5TBB1;A0A087W	Ribonuclease H2 s	RNASEH2B
NaN	NaN	NaN	NaN	NaN	NaN	NaN	NaN	NaN	NaN	NaN	22.7328	Q8TDP1;H0YEF3;E	Q8TDP1;H0YEF3;E	Ribonuclease H2 s	RNASEH2C
NaN	23.6505	NaN	NaN	22.7409	NaN	24.7404	24.6518	23.8466	NaN	22.4198	22.7959	O00584;D6REQ6;I	O00584;D6REQ6;I	Ribonuclease T2	RNASET2
24.473	NaN	24.478	24.3614	24.5328	24.4884	24.5127	24.0346	25.0634	24.9049	24.7032	24.8482	P61587;E9PFH1;Q	P61587;E9PFH1;Q	Rho-related GTP-b	RND3;ARHE
23.0143	22.6823	22.6209	22.5638	22.4677	NaN	NaN	NaN	NaN	22.6464	NaN	NaN	O15541	O15541	RING finger prote	RNF113A
24.4222	24.8418	24.4042	24.2936	24.583	NaN	24.363	NaN	NaN	24.5983	24.6176	NaN	Q9Y508;A0A096LI	Q9Y508;A0A096LI	E3 ubiquitin-prote	RNF114
NaN	NaN	22.0475	22.1366	NaN	NaN	NaN	NaN	NaN	22.0796	NaN	NaN	Q5XPI4;Q5XPI4-2;	Q5XPI4;Q5XPI4-2;	E3 ubiquitin-prote	RNF123
22.2881	22.5047	NaN	22.4537	NaN	NaN	NaN	NaN	NaN	NaN	NaN	NaN	Q8NC42;F8WCD0	Q8NC42;F8WCD0	E3 ubiquitin-prote	RNF149
24.9792	23.5552	23.952	24.4808	23.911	23.5947	NaN	NaN	NaN	24.2024	24.1807	24.3815	Q5VTR2;C9J0A5;C	Q5VTR2	E3 ubiquitin-prote	RNF20
26.7423	NaN	25.9748	26.5534	25.4599	25.517	25.7936	NaN	NaN	25.9896	NaN	25.626	Q63HN8;A0A0A0I	Q63HN8;A0A0A0I	E3 ubiquitin-prote	RNF213
22.0561	NaN	21.6912	NaN	NaN	NaN	NaN	NaN	NaN	NaN	NaN	NaN	Q96EP0;A0A0B4J2	Q96EP0;A0A0B4J2	E3 ubiquitin-prote	RNF31
23.9026	24.0964	23.8435	24.2728	24.2912	24.3166	NaN	NaN	NaN	23.8129	24.242	24.2002	O75150;H3BP71;C	O75150;H3BP71;C	E3 ubiquitin-prote	RNF40
NaN	NaN	NaN	NaN	NaN	21.5387	NaN	NaN	NaN	NaN	NaN	NaN	Q99942	Q99942	E3 ubiquitin-prote	RNF5
NaN	NaN	NaN	23.0432	NaN	NaN	NaN	NaN	NaN	NaN	NaN	NaN	Q9UBF6;F8WEU8;	Q9UBF6;F8WEU8;	RING-box protein	RNF7
26.4902	26.5544	26.7941	27.0467	28.2	26.8627	NaN	NaN	NaN	27.3782	27.8269	NaN	O60942;O60942-2	O60942;O60942-2	mRNA-capping en	RNGTT
28.6466	28.1603	28.1069	28.406	27.6913	28.0742	26.9992	26.5959	26.6707	27.648	27.4665	27.3483	P13489;E9PIM9;H	P13489	Ribonuclease inhil	RNH1
25.9173	24.7528	25.0666	25.5463	24.7617	25.0427	23.9725	24.3631	NaN	25.6427	25.4186	24.4236	O43148;O43148-2	O43148;O43148-2	mRNA cap guanin	RNMT
26.9536	27.0349	27.1412	26.3966	27.0349	27.03	27.3893	26.3833	27.1485	27.6964	27.553	27.5684	Q9H4A4;A6NKB8;	Q9H4A4;A6NKB8	Aminopeptidase B	RNPEP
26.5726	25.981	26.3374	26.8225	26.0232	26.5943	24.5328	25.9424	24.2519	26.1927	26.4118	25.3523	Q15287;H3BV80;H	Q15287;H3BV80;H	RNA-binding prote	RNPS1
NaN	NaN	22.9768	22.2051	NaN	NaN	NaN	NaN	NaN	23.4187	NaN	NaN	Q9Y6N7;A0A0A0M	Q9Y6N7;A0A0A0M	Roundabout hom	ROBO1
25.5176	25.2911	25.5645	25.4282	25.4115	25.769	26.1796	25.531	25.7976	25.8637	25.8655	25.9789	Q13464;A0A0U1R	Q13464;A0A0U1R	Rho-associated pr	ROCK1
27.5945	27.2519	27.5941	27.3909	27.4775	27.889	28.055	28.1315	27.9504	28.1807	27.9867	28.258	O75116;E9PF63;Q	O75116;E9PF63;Q	Rho-associated pr	ROCK2
NaN	NaN	NaN	22.4092	NaN	NaN	NaN	NaN	NaN	NaN	NaN	NaN	P60602;P60602-2	P60602;P60602-2	Reactive oxygen s	ROMO1
22.8632	23.1229	24.2386	22.8701	23.0237	NaN	NaN	NaN	NaN	23.2633	22.6507	NaN	Q01973;A0A0A0M	Q01973;A0A0A0M	Tyrosine-protein k	ROR1
28.2287	27.3122	28.4453	27.9423	27.3427	28.6091	28.1799	25.4817	26.8472	28.934	28.0981	27.5023	O75695	O75695	Protein XRP2	RP2
29.2681	27.8703	28.3318	28.8643	28.0945	28.1287	27.0488	26.6738	24.2371	29.042	28.4201	27.3305	P27694;I3L4R8;I3I	P27694;I3L4R8	Replication protei	RPA1
25.3451	25.2194	24.8767	24.8406	25.1052	24.9734	NaN	NaN	NaN	25.3694	25.4787	25.7023	P15927;P15927-2	P15927;P15927-2	Replication protei	RPA2
25.134	25.2427	24.9696	25.0347	25.0803	25.0347	24.4644	25.1349	24.8258	24.9508	25.1605	26.0494	P35244;B5MC59	P35244;B5MC59	Replication protei	RPA3
21.7952	NaN	NaN	NaN	NaN	NaN	NaN	NaN	NaN	NaN	NaN	NaN	Q9BWH6;Q9BWH	Q9BWH6;Q9BWH	RNA polymerase I	RPAP1
NaN	NaN	NaN	19.7814	NaN	NaN	NaN	NaN	NaN	NaN	NaN	NaN	Q8IXW5;Q8IXW5-	Q8IXW5;Q8IXW5-	Putative RNA poly	RPAP2
28.0208	28.5589	28.1792	27.9642	28.272	28.0043	28.0541	NaN	28.2431	27.8738	28.057	NaN	Q9H6T3;Q9H6T3-	Q9H6T3;Q9H6T3-	RNA polymerase I	RPAP3
NaN	23.348	22.6013	22.3848	22.7946	NaN	23.3705	NaN	23.0278	NaN	NaN	22.9209	Q96AT9;C9J6A7;C	Q96AT9;C9J6A7;C	Ribulose-phospha	RPE
23.4073	22.8428	23.5447	24.9057	24.3726	NaN	NaN	NaN	NaN	21.9983	23.7204	NaN	Q9H9Y2	Q9H9Y2	Ribosome produc	RPF1
26.1279	25.3792	25.5956	26.7591	25.888	25.6761	23.9856	NaN	NaN	25.262	25.7367	25.3103	Q9H7B2;Q5VXN0	Q9H7B2;Q5VXN0	Ribosome produc	RPF2
24.3509	24.7267	24.6426	23.9203	24.5758	23.9806	24.1655	24.8054	24.9089	24.4449	24.5055	24.819	P49247	P49247	Ribose-5-phospha	RPIA
26.493	26.6047	27.5316	28.0567	27.615	27.6771	25.2604	27.1546	24.5083	26.3188	27.4724	26.5249	F8W7C6;A0A087V	F8W7C6;A0A087WV22		RPL10
31.5373	30.5253	31.2445	32.0039	31.2426	31.5647	30.057	31.1855	31.1773	30.9179	31.3895	30.9777	P27635;X1WI28;C	P27635;X1WI28	60S ribosomal prc	RPL10
31.6561	29.9672	30.6325	31.7473	30.2109	30.7841	29.9148	30.0851	28.8463	30.3746	30.1701	29.1816	P62906	P62906	60S ribosomal prc	RPL10A
31.1621	30.6195	30.6715	31.2539	31.3588	30.8616	29.8474	31.1747	30.2729	30.9475	31.3411	30.6623	P62913;Q5VVC8;(	P62913;Q5VVC8;(	60S ribosomal prc	RPL11
24.6475	25.037	25.7467	26.0215	26.2013	25.9209	24.1472	25.0463	NaN	25.7175	26.3145	NaN	P62913-2	P62913-2	60S ribosomal prc	RPL11
29.2432	28.9308	28.9874	29.3109	29.7038	29.1594	28.2157	29.0741	28.5332	28.9133	29.2906	28.7849	P30050;P30050-2	P30050;P30050-2	60S ribosomal prc	RPL12
31.1712	30.5784	30.9801	31.5826	31.2379	31.2239	30.1024	31.3952	30.671	30.6145	31.563	31.3187	P26373;P26373-2	P26373;P26373-2	60S ribosomal prc	RPL13
31.4861	30.5716	31.2587	31.8326	31.1317	31.3803	30.5905	31.4509	30.2704	31.4868	31.6123	30.9947	P40429;M0QYS1;(	P40429;M0QYS1;(	60S ribosomal prc	RPL13A;RPL13a
30.8672	30.0359	30.2192	31.1845	30.6905	30.4994	29.7887	30.7451	29.9307	30.63	30.8504	30.644	P50914;E7EPB3	P50914;E7EPB3	60S ribosomal prc	RPL14
31.9199	30.8359	31.4731	32.2762	31.7167	31.6908	30.8837	32.0989	30.8214	31.6315	31.922	31.019	P61313;E7EQV9;E	P61313;E7EQV9;E	60S ribosomal prc	RPL15
30.5854	29.7263	30.3398	31.0326	30.147	30.5957	29.1832	30.2101	29.3359	30.109	30.2192	29.7838	P18621;A0A087W	P18621;A0A087W	60S ribosomal prc	RPL17;RPL17-C18c
32.2254	30.6498	31.3123	32.2031	30.8686	31.2383	30.9374	31.4166	30.4266	31.2801	31.2279	30.8002	Q07020;J3QQ67;(	Q07020;J3QQ67;(	60S ribosomal prc	RPL18
30.328	30.2661	30.9684	31.5001	31.0394	31.1984	29.6853	31.2163	30.6675	30.8728	31.3012	30.5429	Q02543;M0R117;	Q02543;M0R117;	60S ribosomal prc	RPL18A
30.5522	29.9086	30.4641	30.7832	30.6006	30.7037	30.1803	31.4256	30.2219	30.7634	31.334	30.5795	P84098;J3QR09;J3	P84098;J3QR09;J3	60S ribosomal prc	RPL19

30.8831	30.2041	30.693	31.0543	30.8027	30.8277	29.7708	31.2293	30.5402	30.4613	31.115	30.7417	P46778;M0R181;(	P46778;M0R181	60S ribosomal prc	RPL21
30.1633	29.2896	30.2526	30.8345	30.12	30.5211	29.2784	30.4559	29.7624	30.296	30.7668	30.1177	P35268;K7ERI7;K7	P35268;K7ERI7;K7	60S ribosomal prc	RPL22
NaN	NaN	23.0611	NaN	NaN	NaN	NaN	NaN	NaN	NaN	NaN	NaN	Q6P5R6;C9JYQ9;H	Q6P5R6;C9JYQ9;H	60S ribosomal prc	RPL22L1
30.0058	29.299	29.3485	30.1855	29.567	29.5004	29.0987	29.8101	29.7385	30.0026	30.158	29.5924	P62829;C9JD32;J3	P62829;C9JD32;J3	60S ribosomal prc	RPL23
30.1712	29.1404	29.5704	29.8893	29.0906	29.3929	29.609	30.2497	28.2215	29.754	30.1251	29.1548	P62750;H7BY10;K	P62750;H7BY10;K	60S ribosomal prc	RPL23A
29.7662	29.1218	29.6227	30.0656	29.7085	29.6277	27.5906	29.6339	28.9557	28.7678	29.6192	29.2912	P83731;C9JXB8;C9	P83731;C9JXB8;C9	60S ribosomal prc	RPL24
31.0539	30.545	30.7046	31.2371	30.9673	31.0142	29.8589	31.447	30.7351	30.8802	31.3778	31.0836	P61254;J3QRI7;J3	P61254;J3QRI7;J3	60S ribosomal prc	RPL26;RPL26L1
31.4081	29.6058	30.2842	31.1255	30.0034	30.3246	29.7793	30.3965	29.0758	30.5782	30.6952	29.9389	P61353;K7ELC7;K7	P61353;K7ELC7;K7	60S ribosomal prc	RPL27
30.3373	29.3953	29.983	30.4741	30.0377	30.0441	28.4074	30.4783	29.6475	29.6651	30.3727	30.036	P46776;E9PJD9;E9	P46776;E9PJD9;E9	60S ribosomal prc	RPL27A
30.9876	30.5676	30.8429	31.2432	31.3603	30.8024	30.3514	31.8702	31.006	31.0659	31.5499	30.6033	P46779;H0YKD8;P	P46779;H0YKD8;P	60S ribosomal prc	RPL28
28.8212	28.8892	29.1648	29.2125	29.2947	29.383	28.2658	30.3532	28.8945	28.6283	29.9548	29.8957	P47914;F8WF43;(	P47914	60S ribosomal prc	RPL29
32.7834	31.8426	32.2534	32.8564	32.4551	32.3094	31.3947	32.7286	31.8335	32.0089	32.2484	32.2571	P39023;G5E9G0;E	P39023;G5E9G0;E	60S ribosomal prc	RPL3
30.4618	29.8386	29.7533	30.2685	30.501	29.81	28.8939	30.4309	29.2323	29.2437	30.2058	29.9886	P62888;E5RI99;E5	P62888;E5RI99;E5	60S ribosomal prc	RPL30
29.7742	28.9676	29.4451	29.9933	29.3712	29.4601	28.9514	30.2535	29.1551	29.7586	30.1833	29.4387	P62899;P62899-3	P62899;P62899-3	60S ribosomal prc	RPL31
30.857	29.9714	30.3545	30.948	30.5877	30.623	29.3737	30.832	29.9147	30.745	31.0091	30.0292	P62910;D3YTB1;F	P62910;D3YTB1;F	60S ribosomal prc	RPL32
30.2641	29.1205	29.8302	30.0214	29.6653	30.0923	28.9433	30.0271	29.1337	29.2589	30.0967	29.7559	P49207	P49207	60S ribosomal prc	RPL34
29.5981	28.6897	29.4434	29.8955	29.4671	29.78	28.3973	29.972	29.1191	29.068	29.7689	29.5712	P42766;F2Z388	P42766;F2Z388	60S ribosomal prc	RPL35
29.907	29.0992	29.6284	30.4018	29.6407	29.6029	29.1013	30.1873	29.1067	30.2335	30.126	29.5213	P18077;C9K025;F	P18077;C9K025;F	60S ribosomal prc	RPL35A
29.4636	28.882	29.4046	29.6097	28.9678	29.3682	28.8376	29.7155	28.9556	29.2194	29.8712	29.3075	Q9Y3U8;J3QSB5;J	Q9Y3U8;J3QSB5	60S ribosomal prc	RPL36
NaN	NaN	NaN	NaN	NaN	NaN	NaN	24.2588	NaN	NaN	NaN	NaN	P83881;J3KQN4;H	P83881;J3KQN4;H	60S ribosomal prc	RPL36A;RPL36A-H
27.1125	26.7925	26.357	26.8249	27.0757	25.9189	26.9813	28.7954	27.3147	25.3795	27.037	27.2477	Q969Q0;R4GN19	Q969Q0	60S ribosomal prc	RPL36AL
23.6664	NaN	24.5965	NaN	NaN	25.8658	26.7888	28.7636	27.8859	NaN	25.8912	20.6062	P61927;D6RG19	P61927	60S ribosomal prc	RPL37
27.6669	26.9499	26.7539	28.0027	27.2214	26.5946	27.9435	28.2038	NaN	26.3534	26.838	26.0604	P61513;C9J4Z3;Q	P61513;C9J4Z3;Q	60S ribosomal prc	RPL37A
27.774	27.9832	28.1046	28.7131	28.2416	28.3166	26.9753	27.7606	27.279	27.493	28.0926	27.1604	P63173;J3KT73;J3	P63173;J3KT73;J3	60S ribosomal prc	RPL38
24.1007	24.571	25.566	24.0622	NaN	NaN	NaN	NaN	23.8832	NaN	26.3271	25.6288	P62891;Q59GN2	P62891;Q59GN2	60S ribosomal prc	RPL39;RPL39P5
NaN	24.6871	19.9439	NaN	25.6597	19.7485	22.2423	NaN	NaN	NaN	NaN	NaN	Q92901	Q92901	60S ribosomal prc	RPL3L
32.2937	31.2966	31.8908	32.4061	31.6328	32.0214	30.9995	31.9631	30.865	31.8868	32.0174	31.4641	P36578;H3BM89;I	P36578;H3BM89	60S ribosomal prc	RPL4
30.8121	30.1456	30.9354	30.8449	30.3238	30.7211	30.5829	30.8689	29.9096	31.3886	31.3969	30.7658	P46777;Q5T7N0;F	P46777	60S ribosomal prc	RPL5
31.9778	30.9433	31.5087	32.1131	31.3258	31.6148	30.6912	31.6168	30.7658	31.6127	31.998	31.4652	Q02878;F8VZ45;U	Q02878	60S ribosomal prc	RPL6
32.3731	31.0958	31.7819	32.6186	31.5138	32.0625	31.1138	31.6314	30.8411	31.7842	31.6088	31.386	P18124;A8MUD9;	P18124;A8MUD9	60S ribosomal prc	RPL7
32.369	31.2159	31.7048	32.4765	31.6729	31.873	31.1853	31.8266	30.7965	31.5653	31.8615	31.5663	P62424;Q5T8U3;(	P62424;Q5T8U3	60S ribosomal prc	RPL7A
24.8936	22.664	24.1266	25.3423	23.4447	23.834	23.4754	NaN	NaN	23.5403	23.0866	NaN	Q6DKI1;R4GMU7;	Q6DKI1;R4GMU7;	60S ribosomal prc	RPL7L1
31.7072	30.9032	31.3513	31.6635	31.2771	31.535	30.6834	31.786	30.792	31.1311	31.6513	31.1793	P62917;E9PKZ0;E	P62917;E9PKZ0;E	60S ribosomal prc	RPL8
29.9593	29.2699	29.81	30.3509	29.8276	29.7051	28.7281	29.8628	29.5025	29.3263	29.5937	29.2671	P32969;D6RAN4;E	P32969;D6RAN4;E	60S ribosomal prc	RPL9
31.2004	29.8953	30.0811	31.06	30.4458	30.3237	29.7443	29.9614	29.6588	30.0559	30.0976	29.7801	P05388;F8VWS0;F	P05388;F8VWS0;F	60S acidic ribosom	RPLP0;RPLP0P6
30.8092	30.0677	28.7146	31.2903	29.7306	NaN	NaN	NaN	NaN	NaN	NaN	NaN	P05386	P05386	60S acidic ribosom	RPLP1
31.0292	30.434	30.1861	30.9801	30.2468	30.2186	29.7661	30.2319	29.3413	30.2683	30.0198	29.3037	P05387;H0YDD8;F	P05387	60S acidic ribosom	RPLP2
28.5532	28.4663	28.6427	29.1696	28.6131	28.9531	28.9269	29.0909	29.759	28.8246	28.4925	28.7671	P04843;B7Z4L4;F	P04843;B7Z4L4	Dolichyl-diphosph	RPN1
27.4977	27.3639	27.3709	27.9388	27.2935	27.5236	27.7304	27.8931	28.6697	27.2442	27.1668	27.4898	P04844;P04844-2	P04844;P04844-2	Dolichyl-diphosph	RPN2
20.7635	NaN	NaN	NaN	NaN	NaN	NaN	NaN	NaN	NaN	NaN	NaN	O95059	O95059	Ribonuclease P pr	RPP14
23.0317	22.7517	NaN	23.0305	23.025	NaN	NaN	NaN	NaN	22.7393	24.1518	NaN	Q9BUL9	Q9BUL9	Ribonuclease P pr	RPP25
25.0213	23.0283	23.2689	24.5478	23.4493	23.7581	22.3803	NaN	NaN	24.579	22.9911	NaN	P78346;P78346-2	P78346;P78346-2	Ribonuclease P pr	RPP30
23.8185	NaN	NaN	24.0099	NaN	NaN	NaN	NaN	NaN	23.3038	23.8151	NaN	O75818;O75818-2	O75818;O75818-2	Ribonuclease P pr	RPP40
25.0844	25.9544	25.6918	25.4353	26.3843	25.9796	25.292	25.2254	25.6366	26.1685	26.4247	26.7514	Q9NQG5;E9PIQ9;	Q9NQG5	Regulation of nucl	RPRD1B
NaN	NaN	NaN	22.1231	NaN	NaN	NaN	NaN	NaN	NaN	NaN	NaN	Q5VT52;Q5VT52-5	Q5VT52;Q5VT52-5	Regulation of nucl	RPRD2
29.7475	29.529	29.4149	29.6103	29.687	29.4278	28.7861	29.302	28.4947	29.4085	29.232	28.7168	P46783;F6U211;C	P46783;F6U211	40S ribosomal prc	RPS10
NaN	NaN	23.1742	NaN	22.8072	23.0987	NaN	NaN	NaN	23.0867	23.3028	23.1349	S4R435;O95989	S4R435		RPS10-NUDT3
31.4195	30.4585	30.8807	31.3425	30.91	30.9443	29.9779	31.4939	30.9175	30.9273	31.445	31.1765	P62280;M0QZC5;I	P62280;M0QZC5	40S ribosomal prc	RPS11
29.4904	28.5454	28.4182	29.1521	28.2765	28.3231	28.6387	28.2454	27.916	28.4743	28.7804	28.2851	P25398	P25398	40S ribosomal prc	RPS12
30.9417	29.6189	30.2353	30.8283	29.9717	30.2547	29.2595	30.1689	29.5722	30.0706	30.2971	29.9411	P62277;J3KMX5;E	P62277;J3KMX5	40S ribosomal prc	RPS13
31.0884	30.5024	30.4387	31.0473	30.8658	30.5258	29.5032	30.886	29.8047	30.3157	30.8395	30.5052	P62263;H0YB22;E	P62263;H0YB22;E	40S ribosomal prc	RPS14
28.3047	28.7232	28.8705	29.2094	28.4934	28.4475	28.9236	29.5691	27.9973	28.8135	29.3793	28.421	P62841;K7ELC2;K	P62841;K7ELC2;K	40S ribosomal prc	RPS15
30.4445	29.6247	30.1122	30.7159	30.0911	30.2539	28.5885	29.3324	29.7331	29.7571	30.2569	29.6839	P62244;I3L3P7;I3	P62244;I3L3P7;I3	40S ribosomal prc	RPS15A
31.5576	30.8761	31.1737	31.9196	31.426	31.4713	30.0823	31.0517	30.7486	31.3829	31.3449	30.8711	P62249;M0R210;A	P62249;M0R210;A	40S ribosomal prc	RPS16;ZNF90
30.5229	30.1266	30.0752	30.123	30.7913	29.8944	29.792	31.0375	30.5878	29.639	30.2317	30.1081	P08708;H0YN88;A	P08708;H0YN88;A	40S ribosomal prc	RPS17
31.3005	30.3806	30.8415	31.3681	30.9373	30.9245	30.2766	31.628	30.8166	31.1899	31.1421	30.6331	P62269;J3JS69;A0	P62269	40S ribosomal prc	RPS18
29.9822	29.9248	29.8435	29.8782	29.8687	29.7338	29.4335	30.8502	29.2888	30.2281	30.5984	29.7981	P39019;A0A075B(	P39019;A0A075B(	40S ribosomal prc	RPS19
32.0682	30.8542	31.5114	32.1517	31.314	31.6413	30.4753	31.2892	30.5096	31.3073	31.3	31.003	P15880;H0YEN5;E	P15880;H0YEN5;E	40S ribosomal prc	RPS2
29.7045	29.477	29.3483	29.8824	29.6202	29.4175	28.6921	29.1129	29.1526	29.2099	29.8472	29.2532	P60866;P60866-2	P60866;P60866-2	40S ribosomal prc	RPS20
27.8666	28.8062	28.2909	27.411	28.7923	27.9986	27.9797	29.7728	28.7359	27.5754	29.006	29.0619	P63220;Q8WVC2;	P63220;Q8WVC2;	40S ribosomal prc	RPS21

30.6734	29.8624	29.9974	30.5607	30.6888	30.1719	29.1981	30.8879	29.7841	29.5781	30.3174	30.0715	P62266;D6RD47;D	P62266;D6RD47;D	40S ribosomal prc	RPS23
30.0919	29.714	30.0784	30.2984	30.2283	30.3144	29.061	30.4804	29.719	29.7894	30.4493	30.0358	P62847;E7ETK0;A	P62847;E7ETK0;A	40S ribosomal prc	RPS24
29.9788	28.8089	29.5262	30.1422	29.212	29.5384	28.4482	29.8802	29.2085	29.6993	29.8868	29.7095	P62851	P62851	40S ribosomal prc	RPS25
30.952	29.4887	29.9035	30.5392	29.4263	29.4831	28.7305	29.3661	28.703	29.3888	28.9967	29.0384	P62854;Q5JNZ5	P62854	40S ribosomal prc	RPS26
30.1597	28.9336	28.9846	29.9027	29.0466	28.9803	27.5819	28.7727	27.5535	29.0024	29.3405	28.0206	P42677;Q5T4L4;C	P42677;Q5T4L4	40S ribosomal prc	RPS27
30.4148	30.1801	30.6735	30.5305	29.9127	30.5057	30.6422	30.689	30.455	30.2554	30.1655	29.9276	P62979;P62987;J3	P62979;P62987;J3	Ubiquitin-40S ribc	RPS27A;UBA52
25.7975	26.0628	25.8037	25.7975	26.2971	25.7974	25.6116	NaN	NaN	25.9413	26.6759	NaN	Q71UM5;H0YMV8	Q71UM5;H0YMV8	40S ribosomal prc	RPS27L
26.5925	27.3269	26.9886	26.5464	27.0756	26.7459	27.5064	27.7149	26.7822	26.4633	27.7665	27.1204	P62857	P62857	40S ribosomal prc	RPS28
26.2678	26.6971	26.7966	25.7941	26.6875	26.3474	26.4205	27.8394	NaN	26.7844	27.6337	NaN	P62273;P62273-2	P62273;P62273-2,	40S ribosomal prc	RPS29
NaN	NaN	NaN	NaN	NaN	NaN	NaN	NaN	NaN	NaN	20.7199	NaN	E9PJN9	E9PJN9		RPS3
31.9119	31.0647	31.54	32.185	31.6824	31.7373	30.5317	31.0249	31.1227	31.6595	31.7546	30.904	P23396;P23396-2,	P23396;P23396-2,	40S ribosomal prc	RPS3
32.3506	31.4018	31.7242	32.492	31.5316	31.8055	30.9746	31.6896	30.911	31.4716	31.5178	31.1782	P61247;D6RAT0;D	P61247;D6RAT0;D	40S ribosomal prc	RPS3A
32.1183	31.2286	31.9016	32.5742	31.8062	32.0944	30.9657	31.9247	31.5691	32.0347	32.2442	31.7926	P62701;Q8TD47	P62701	40S ribosomal prc	RPS4X
31.0625	30.419	30.5606	31.0392	30.8893	30.4306	29.7134	30.3554	29.8114	30.1135	30.5814	30.0816	P46782;M0R0F0;N	P46782;M0R0F0;N	40S ribosomal prc	RPS5
31.5524	30.7984	31.2177	31.7885	31.174	31.4615	30.3215	31.4168	30.7516	31.1823	31.4072	30.8486	P62753;A2A3R5;A	P62753;A2A3R5	40S ribosomal prc	RPS6
25.9804	25.5766	25.6155	25.8965	25.6804	25.8894	25.8217	25.5718	26.3033	25.8982	25.6323	25.8699	Q15418;Q15418-2	Q15418;Q15418-2	Ribosomal proteir	RPS6KA1
27.9083	27.0702	27.5617	27.6394	27.1019	27.5598	27.0547	26.9716	26.7486	27.6152	27.3532	27.098	P51812;B1AXG1;C	P51812	Ribosomal proteir	RPS6KA3
23.3753	NaN	23.5754	23.4453	NaN	23.268	NaN	NaN	NaN	23.6066	23.8394	NaN	O75676;A0A0A6Y	O75676;A0A0A6Y	Ribosomal proteir	RPS6KA4
24.0027	23.0152	23.8748	23.7632	23.3217	NaN	NaN	NaN	NaN	NaN	NaN	NaN	P23443;P23443-2,	P23443;P23443-2,	Ribosomal proteir	RPS6KB1
31.5389	30.7991	31.2816	31.4281	31.1825	31.3277	30.0894	31.2063	30.7436	30.6411	31.3328	30.9539	P62081;B5MCP9	P62081;B5MCP9	40S ribosomal prc	RPS7
32.1262	31.0865	31.6356	32.2581	31.3026	31.6403	30.6284	31.7733	30.7044	31.5073	31.6007	31.0596	P62241;Q5JR95	P62241;Q5JR95	40S ribosomal prc	RPS8
32.6294	31.1194	31.9576	32.7808	31.729	32.1518	30.8562	31.3659	31.0876	31.6603	31.8036	31.4443	P46781;A0A024R4	P46781;A0A024R4	40S ribosomal prc	RPS9
31.3277	30.7018	30.8556	31.3372	30.7428	30.8766	30.2793	30.6821	30.474	31.8183	32.1025	30.7345	P08865;C9J9K3;A	P08865;C9J9K3;A	40S ribosomal prc	RPSA
26.4786	25.3121	25.3713	26.1285	25.1785	26.3507	25.4916	25.5405	NaN	25.6881	25.5504	26.1179	Q8N122;Q8N122-	Q8N122;Q8N122-	Regulatory-associ	RPTOR
21.3475	NaN	21.803	21.2161	21.4295	NaN	NaN	NaN	NaN	21.9456	22.2981	22.5546	Q8IZ73;Q8IZ73-2;	Q8IZ73;Q8IZ73-2	RNA pseudouridyl	RPUSD2
24.8191	24.4649	24.2857	24.4551	NaN	24.2407	NaN	NaN	NaN	24.1056	NaN	NaN	Q92600;Q92600-2	Q92600;Q92600-2	Cell differentiatior	RQCD1
NaN	NaN	NaN	NaN	NaN	NaN	NaN	NaN	22.8588	NaN	NaN	NaN	P55042;J3KRG9;J3	P55042;J3KRG9;J3	GTP-binding prote	RRAD
25.321	24.8633	24.5888	25.2706	24.8169	NaN	NaN	NaN	NaN	24.8767	24.8877	NaN	Q7L523;Q5VZM2-	Q7L523;Q5VZM2-	Ras-related GTP-b	RRAGA;RRAGB
24.2912	23.8399	23.7709	23.5242	23.6192	23.4237	23.6711	NaN	23.7588	23.8431	23.5877	23.6682	Q9HB90;Q9NQL2;	Q9HB90;Q9NQL2	Ras-related GTP-b	RRAGC;RRAGD
27.2254	26.9201	27.6147	27.1107	26.8553	27.6676	26.7137	26.3621	27.1326	27.0074	26.5792	26.9539	P10301	P10301	Ras-related protei	RRAS
29.422	29.3779	30.2134	29.5832	29.6153	30.4904	29.0875	28.7852	29.2561	29.8433	29.6393	29.7292	P62070;P62070-4,	P62070;P62070-4,	Ras-related protei	RRAS2
28.2839	27.791	27.3688	28.196	27.5837	26.3326	26.9537	27.9737	26.7144	24.4762	24.1323	25.199	Q9P2E9;A0A0A0N	Q9P2E9;A0A0A0N	Ribosome-binding	RRBP1
28.654	27.9206	28.5873	27.4139	27.4832	27.4905	27.386	26.2693	27.0345	26.8782	26.6392	25.1234	P23921;E9PL69;E9	P23921;E9PL69	Ribonucleoside-di	RRM1
26.2781	26.2845	26.5588	24.5369	25.2563	25.0949	25.7999	26.7069	26.0559	24.8229	24.9704	25.7585	P31350;P31350-2,	P31350;P31350-2,	Ribonucleoside-di	RRM2
22.0463	NaN	NaN	NaN	NaN	NaN	NaN	NaN	NaN	NaN	NaN	NaN	Q7LG56;Q7LG56-(	Q7LG56;Q7LG56-(	Ribonucleoside-di	RRM2B
23.1043	NaN	22.219	22.6228	22.4115	NaN	NaN	NaN	NaN	NaN	NaN	NaN	P56182;D6RE82	P56182	Ribosomal RNA pr	RRP1
27.8455	25.2504	26.2476	27.3336	25.535	26.2399	24.2497	NaN	NaN	26.1621	25.3503	23.5171	Q5JTH9;Q5JTH9-2	Q5JTH9;Q5JTH9-2	RRP12-like proteir	RRP12
24.2942	NaN	23.46	24.3663	23.0716	23.383	NaN	NaN	NaN	NaN	23.5291	NaN	Q9Y3B9	Q9Y3B9	RRP15-like proteir	RRP15
23.8164	NaN	23.0604	23.5352	NaN	NaN	NaN	NaN	NaN	NaN	NaN	NaN	Q14684;Q14684-2	Q14684;Q14684-2	Ribosomal RNA pr	RRP1B
23.1361	NaN	23.3137	23.7876	23.1515	NaN	NaN	NaN	NaN	NaN	NaN	NaN	O43159;E9PPP6;E	O43159;E9PPP6;E	Ribosomal RNA-pı	RRP8
25.6221	23.8113	24.4633	25.754	24.9845	23.3484	NaN	NaN	NaN	25.8549	25.6474	25.0501	O43818	O43818	U3 small nucleola	RRP9
25.3833	24.0186	24.2047	25.3201	24.9508	23.7905	NaN	NaN	NaN	23.7149	23.7428	NaN	Q15050	Q15050	Ribosome biogen	RRS1
23.1354	NaN	22.2314	23.521	22.2435	22.5305	NaN	NaN	NaN	NaN	NaN	NaN	Q5VWQ0;A0A0C4	Q5VWQ0;A0A0C4	Round spermatid	RSBN1
NaN	NaN	NaN	NaN	NaN	NaN	NaN	NaN	NaN	NaN	21.8529	NaN	Q96T23;H7C306;F	Q96T23;H7C306;F	Remodeling and s	RSF1
28.8673	27.3474	28.0146	29.2966	28.2011	28.3636	26.3562	25.0226	26.0594	28.1239	28.1683	27.6838	O76021;J3QSV6;J	O76021;J3QSV6;J	Ribosomal L1 dom	RSL1D1
NaN	NaN	NaN	21.806	NaN	NaN	NaN	NaN	NaN	NaN	NaN	NaN	Q9UHA3;H3BMQ2	Q9UHA3;H3BMQ2	Probable ribosom	RSL24D1
23.4696	23.554	24.3705	23.7153	23.4606	24.5949	24.4237	NaN	NaN	24.9806	24.478	23.8086	Q96DX4	Q96DX4	RING finger and S	RSPRY1
23.9345	NaN	NaN	23.655	NaN	NaN	NaN	NaN	NaN	NaN	NaN	NaN	Q7L4I2;Q7L4I2-2;	Q7L4I2;Q7L4I2-2	Arginine/serine-ri	RSRC2
25.3503	25.9502	25.605	25.3322	25.5327	25.4422	25.9153	25.5092	25.169	25.7751	25.1858	25.8503	Q15404;Q15404-2	Q15404;Q15404-2	Ras suppressor pr	RSU1
25.658	24.9442	25.1704	25.7397	24.9306	25.089	24.8756	23.9482	24.0897	25.6895	25.3783	24.8627	O00442;O00442-2	O00442;O00442-2	RNA 3-terminal pl	RTCA
29.2117	28.6248	28.339	28.5794	28.8347	28.4901	28.2314	28.495	28.4153	29.4815	29.6738	28.7453	Q9Y3I0	Q9Y3I0	tRNA-splicing liga:	RTCB
22.8138	23.1369	23.196	23.1207	22.831	23.0392	22.3343	NaN	NaN	22.8041	NaN	NaN	Q9BY42;A2A2L5;A	Q9BY42;A2A2L5;A	Protein RTF2 hom	RTFDC1
NaN	NaN	NaN	NaN	23.7574	NaN	NaN	NaN	NaN	NaN	NaN	NaN	Q9BST9;Q9BST9-2	Q9BST9;Q9BST9-2	Rhotekin	RTKN
25.7313	24.7628	26.1506	25.5141	25.0652	26.468	25.8332	26.8507	26.7958	26.364	24.7267	26.5015	O95197;O95197-3	O95197;O95197-3	Reticulon-3	RTN3
NaN	NaN	24.0394	NaN	NaN	NaN	NaN	NaN	NaN	23.9488	21.7901	NaN	Q9NQC3-2;F8W91	Q9NQC3-2;F8W91	Reticulon-4;Reticu	RTN4
28.1064	27.3562	27.9334	28.1208	27.1287	27.7683	28.1175	27.5047	27.5659	27.8664	27.0878	27.1029	Q9NQC3-5	Q9NQC3-5	Reticulon-4	RTN4
NaN	NaN	22.2892	NaN	NaN	NaN	NaN	NaN	NaN	NaN	22.4244	NaN	Q8WWV3;G3V1R7	Q8WWV3;G3V1R7	Reticulon-4-intera	RTN4IP1
26.7146	26.5209	26.5682	26.3245	26.014	26.3619	26.2934	26.6721	26.26	25.7487	25.6885	25.8539	Q96T51;Q96T51-2	Q96T51;Q96T51-2	RUN and FYVE dor	RUFY1
22.6578	NaN	NaN	22.5991	NaN	NaN	NaN	NaN	NaN	NaN	NaN	NaN	Q8WXA3-2;Q8WX	Q8WXA3-2;Q8WX	RUN and FYVE dor	RUFY2
30.5258	30.3856	30.2126	30.8281	30.7226	30.443	29.7068	30.4042	29.9883	30.3207	30.4212	30.1891	Q9Y265;Q9Y265-2	Q9Y265;Q9Y265-2	RuvB-like 1	RUVBL1

30.7142	30.6147	30.2242	30.807	30.747	30.4582	29.7875	30.515	29.8885	30.5315	30.6933	30.3179	Q9Y230;Q9Y230-2	Q9Y230;Q9Y230-2	RuvB-like 2	RUVBL2
NaN	NaN	NaN	NaN	NaN	NaN	NaN	NaN	NaN	NaN	NaN	21.8493	Q6NW29;D6R9C7	Q6NW29;D6R9C7	RWD domain-con	RWDD4
20.659	NaN	NaN	21.2788	NaN	NaN	NaN	NaN	NaN	NaN	20.7897	NaN	P28702;A0A0G2JI	P28702;A0A0G2JI	Retinoic acid rece	RXRB
31.9732	30.5706	31.1366	31.5073	30.1111	31.0315	31.4615	30.0469	29.5783	31.5875	30.3568	29.9422	P60903	P60903	Protein S100-A10	S100A10
30.3591	29.2695	29.3132	29.7432	28.7583	28.7973	29.9263	27.9241	NaN	29.964	28.7601	27.8078	P31949	P31949	Protein S100-A11;	S100A11
24.5471	27.047	25.507	25.5318	26.059	25.3907	26.6987	26.3853	26.0935	25.4487	25.6839	27.278	Q99584	Q99584	Protein S100-A13	S100A13
27.135	27.9094	28.0148	27.1557	27.4255	28.0551	28.2488	27.2135	26.9348	28.832	28.184	27.5417	Q96FQ6	Q96FQ6	Protein S100-A16	S100A16
NaN	21.7029	23.8521	NaN	NaN	22.9915	NaN	NaN	NaN	21.844	NaN	NaN	P26447	P26447	Protein S100-A4	S100A4
26.5139	27.3138	27.0655	27.0065	27.0132	27.2798	28.8241	28.1472	27.6498	28.3829	27.8296	28.0523	P06703;R4GN98	P06703;R4GN98	Protein S100-A6;F	S100A6
NaN	NaN	NaN	NaN	NaN	NaN	NaN	NaN	NaN	NaN	NaN	19.8075	P31151;Q86SG5	P31151;Q86SG5	Protein S100-A7;F	S100A7;S100A7A
NaN	NaN	NaN	NaN	NaN	NaN	NaN	NaN	23.1626	NaN	NaN	NaN	P06702	P06702	Protein S100-A9	S100A9
NaN	NaN	NaN	NaN	NaN	NaN	NaN	NaN	NaN	NaN	21.5382	NaN	P25815	P25815	Protein S100-P	S100P
NaN	24.29	23.6262	NaN	23.24	23.4299	NaN	NaN	NaN	NaN	NaN	NaN	P0DJI8;E9PQD6;P(	P0DJI8;E9PQD6;P(	Serum amyloid A-	SAA1;SAA2;SAA2-!
23.3817	NaN	22.1825	23.0885	NaN	NaN	NaN	NaN	NaN	23.0301	22.7491	23.5465	Q96ER3;J3KND1;E	Q96ER3;J3KND1;E	Protein SAAL1	SAAL1
NaN	NaN	NaN	23.2995	NaN	NaN	NaN	NaN	NaN	NaN	NaN	NaN	A6NKF1;F8WC89;	A6NKF1;F8WC89;	SAC3 domain-con	SAC3D1
26.2269	25.7362	25.9453	26.4302	25.9407	25.9387	26.7959	26.7949	26.6972	26.9464	26.5358	26.4733	Q9NTJ5;E9PGZ4;C	Q9NTJ5;E9PGZ4;C	Phosphatidylinosi	SACM1L
24.8804	24.3058	23.764	25.4247	24.3533	24.553	24.8281	NaN	NaN	NaN	23.1906	23.9174	Q9NZJ4;Q9NZJ4-2	Q9NZJ4;Q9NZJ4-2	Sacsin	SACS
25.978	26.3115	26.1965	25.9822	26.7452	26.1511	25.5034	25.835	25.1751	26.1754	26.6753	26.2738	Q9UBE0;Q9UBE0-	Q9UBE0;Q9UBE0-	SUMO-activating	SAE1
25.4178	25.9177	25.8555	25.3755	26.2228	25.6768	24.8009	26.2998	25.6492	24.9445	26.5391	26.9421	Q15424;Q15424-3	Q15424;Q15424-3	Scaffold attachme	SAFB
NaN	NaN	NaN	NaN	NaN	NaN	NaN	NaN	NaN	NaN	NaN	23.3689	Q14151;Q14151-2	Q14151	Scaffold attachme	SAFB2
23.0782	NaN	21.4353	NaN	NaN	NaN	NaN	NaN	NaN	NaN	NaN	NaN	Q9UPU9;Q9UPU9	Q9UPU9;Q9UPU9	Protein Smaug ho	SAMD4A
26.8769	25.8298	26.3233	27.0338	26.8457	26.7582	24.6116	NaN	25.0501	27.2477	27.3726	26.5948	Q9Y3Z3;Q9Y3Z3-4	Q9Y3Z3;Q9Y3Z3-4	Deoxynucleoside	SAMHD1
23.0536	23.3148	23.3317	23.2164	23.3508	23.0682	NaN	NaN	24.0654	NaN	NaN	22.8212	Q9Y512	Q9Y512	Sorting and assen	SAMM50
NaN	NaN	NaN	NaN	26.4027	NaN	NaN	NaN	NaN	NaN	NaN	NaN	Q9H0E3;H7BXF5;(	Q9H0E3;H7BXF5;(	Histone deacetyla	SAP130
27.5148	26.5481	26.6905	27.6209	26.8457	26.5774	25.4961	26.0395	NaN	27.3002	26.923	26.179	X6RAL5;O00422;H	X6RAL5;O00422;H	Histone deacetyla	SAP18
NaN	NaN	NaN	NaN	NaN	NaN	20.8044	NaN	NaN	NaN	NaN	NaN	O75446	O75446	Histone deacetyla	SAP30
28.3067	27.2145	27.6516	27.8226	27.218	27.4597	28.4951	27.6048	27.0852	28.1217	27.762	26.9388	Q9NR31;Q9NR31-	Q9NR31;Q9NR31-	GTP-binding prote	SAR1A
NaN	NaN	NaN	NaN	NaN	NaN	NaN	NaN	20.3852	NaN	NaN	NaN	Q9Y6B6;D6RD69;(	Q9Y6B6;D6RD69	GTP-binding prote	SAR1B
24.3034	25.8945	24.9101	24.1255	25.179	24.9144	24.7964	26.9209	24.6199	24.2887	25.3189	24.9672	P82979;F8VZQ9;H	P82979;F8VZQ9;H	SAP domain-conta	SARNP
29.6023	28.3079	28.4664	28.8488	27.9598	28.0488	28.4223	27.7641	26.5822	29.1184	28.6325	27.8268	P49591;Q5T5C7	P49591;Q5T5C7	Serine--tRNA ligas	SARS
23.8937	23.351	23.2034	23.8815	23.1552	23.0023	NaN	NaN	NaN	23.7753	23.5774	NaN	Q9NP81;M0QWZ	Q9NP81;M0QWZ	Serine--tRNA ligas	SARS2
24.0957	23.6823	24.0076	24.0164	23.8715	23.9527	NaN	NaN	NaN	23.2595	23.6411	NaN	O43290;E9PQI8	O43290	U4/U6.U5 tri-snRI	SART1
26.9147	25.5459	26.3956	26.9696	26.3003	26.1762	23.9046	24.781	23.9914	26.2783	26.3059	26.5407	Q15020;Q15020-4	Q15020;Q15020-4	Squamous cell car	SART3
21.8459	NaN	NaN	NaN	NaN	NaN	NaN	NaN	NaN	NaN	NaN	NaN	Q6UVJ0	Q6UVJ0	Spindle assembly	SASS6
23.1765	NaN	23.2194	23.1259	22.8945	NaN	NaN	NaN	NaN	23.0721	NaN	NaN	Q9H4B6;H0YJH0;I	Q9H4B6;H0YJH0	Protein salvador I	SAV1
26.1998	26.3666	26.1338	26.4255	26.3732	25.8756	27.1253	26.955	27.3014	27.2559	27.1319	27.8011	Q9Y3A5;A0A087X	Q9Y3A5;A0A087X	Ribosome matura	SBDS
24.1243	23.9872	25.3067	24.674	24.2488	24.7465	23.0499	23.3386	23.2067	25.0312	24.905	24.6587	O95248;O95248-4	O95248;O95248-4	Myotubularin-rela	SBF1
22.9841	NaN	22.5239	22.591	NaN	NaN	NaN	NaN	NaN	23.152	22.8213	NaN	A3KN83;A3KN83-:	A3KN83;A3KN83-:	Protein strawberr	SBNO1
NaN	NaN	NaN	NaN	NaN	NaN	NaN	NaN	NaN	NaN	21.8833	NaN	Q9H7N4	Q9H7N4	Splicing factor, arg	SCAF1
NaN	NaN	NaN	NaN	NaN	NaN	NaN	NaN	NaN	NaN	20.7073	NaN	Q99590;A0A0A0M	Q99590;A0A0A0M	Protein SCAF11	SCAF11
23.5603	NaN	22.499	22.954	NaN	NaN	NaN	NaN	NaN	NaN	NaN	NaN	O95104;O95104-2	O95104;O95104-2	Splicing factor, arg	SCAF4
NaN	NaN	NaN	21.6101	NaN	NaN	NaN	NaN	NaN	NaN	NaN	NaN	Q9UPN6;A0A0A0I	Q9UPN6;A0A0A0I	Protein SCAF8	SCAF8
27.6905	26.9999	27.4597	27.6202	27.1479	27.4119	27.2706	26.7946	26.3618	26.2831	26.1481	26.3803	O15126;A0A087W	O15126;A0A087W	Secretory carrier-a	SCAMP1
27.7364	27.3753	27.9553	28.1526	27.5391	28.2259	26.6534	26.7552	27.1966	26.4543	26.6499	27.0746	O15127;H3BV04;I	O15127;H3BV04;I	Secretory carrier-a	SCAMP2
27.6195	27.101	27.2918	27.7329	27.2078	27.595	26.2797	26.7314	27.4356	26.3213	26.3624	27.0964	O14828;O14828-2	O14828;O14828-2	Secretory carrier-a	SCAMP3
26.9944	25.9004	26.3003	26.2032	26.3367	26.7366	25.807	26.1364	27.294	26.5861	26.5504	26.572	Q969E2;Q969E2-2	Q969E2;Q969E2-2	Secretory carrier-a	SCAMP4
26.4467	25.5236	25.912	26.087	25.677	25.8483	26.082	25.5844	26.1938	27.0165	25.8037	26.0202	Q8WTV0-2;B7ZKC	Q8WTV0-2;B7ZKC	Scavenger recepto	SCARB1
28.1841	27.6814	27.8612	27.4725	27.3619	27.8301	27.0705	26.9979	27.8813	26.9798	26.645	27.4989	Q14108;Q14108-2	Q14108	Lysosome membr	SCARB2
25.7973	26.7381	26.4459	25.9749	26.7897	26.4084	25.9714	25.8719	27.2339	26.7372	26.6589	27.2047	Q14162;A0A0A0M	Q14162;A0A0A0M	Scavenger recepto	SCARF1
24.1587	NaN	24.0733	24.6893	24.3228	NaN	24.0799	24.7694	24.6847	24.3038	24.0175	NaN	Q8NBX0	Q8NBX0	Saccharopine deh	SCCPDH
26.6636	27.1707	26.5401	26.1475	26.9654	26.5195	26.4899	26.8948	26.8105	25.9621	26.4119	26.5779	Q8WVM8;Q8WVM	Q8WVM8;Q8WVM	Sec1 family doma	SCFD1
NaN	NaN	NaN	NaN	NaN	NaN	23.0689	NaN	NaN	NaN	NaN	NaN	P13521	P13521	Secretogranin-2;S	SCG2
23.0163	23.1429	23.0248	23.0411	23.9013	NaN	NaN	22.7778	22.7843	23.0444	NaN	23.2713	Q96I15;H7C3V9;H	Q96I15;H7C3V9;H	Selenocysteine lya	SCLY
NaN	NaN	NaN	20.9429	NaN	NaN	NaN	NaN	NaN	NaN	NaN	NaN	Q9UQR0	Q9UQR0	Sex comb on midl	SCML2
25.2721	26.1756	25.7701	25.3326	25.8943	26.0031	25.4589	25.323	26.2391	25.4548	25.6398	25.4935	P22307;P22307-7	P22307;P22307-7	Non-specific lipid-	SCP2
24.6743	23.5765	24.1489	23.033	22.9859	23.0155	25.0337	23.6645	24.2662	25.6532	25.2595	NaN	Q9HB40;I3L4Z9;Q	Q9HB40	Retinoid-inducible	SCPEP1
28.016	28.1387	28.9293	28.463	28.3457	29.1106	28.4991	27.7641	28.0144	29.0489	28.8089	29.1111	Q14160;A0A0G2JI	Q14160;A0A0G2JI	Protein scribble h	SCRIB
26.7217	27.1706	26.9417	26.8826	27.131	27.0967	27.2543	27.3411	26.9721	26.9121	26.9041	27.2825	Q12765;Q12765-2	Q12765;Q12765-2	Secernin-1	SCRN1
23.0946	23.6388	23.0473	22.3593	23.8318	NaN	NaN	NaN	NaN	23.413	23.2945	NaN	Q96FV2;J3QL71;Q	Q96FV2;J3QL71;Q	Secernin-2	SCRN2
26.7612	26.2135	26.6435	26.5045	26.3433	26.561	27.0551	26.724	26.6271	26.9495	26.8858	26.8235	Q96KG9;Q96KG9-	Q96KG9;Q96KG9-	N-terminal kinase	SCYL1

23.2547	NaN	NaN	22.6056	22.3827	NaN	NaN	NaN	NaN	23.1386	22.7158	NaN	Q6P3W7;F8VSC5;	Q6P3W7;F8VSC5;	SCY1-like protein	SCYL2
24.4462	NaN	22.957	24.5687	23.1669	23.0807	NaN	NaN	NaN	NaN	23.4044	NaN	Q9NVU7;E7EW05	Q9NVU7;E7EW05	Protein SDA1 hom	SDAD1
NaN	NaN	NaN	NaN	NaN	NaN	26.7253	NaN	NaN	NaN	NaN	NaN	P18827;E9PHH3;I	P18827	Syndecan-1	SDC1
NaN	NaN	NaN	NaN	NaN	NaN	24.4084	NaN	NaN	NaN	NaN	NaN	P34741;E5RJB8;E5	P34741;E5RJB8;E5	Syndecan-2;Synde	SDC2
26.9863	27.0601	27.2376	26.7768	26.4519	26.6756	29.8203	29.0876	27.1831	27.7507	27.4421	27.9209	P31431;P31431-2	P31431;P31431-2	Syndecan-4	SDC4
30.9274	31.0419	30.122	30.0975	29.8288	29.8036	31.851	30.6964	31.3013	30.4786	29.7025	30.6719	O00560;O00560-3	O00560;O00560-3	Syntenin-1	SDCBP
25.1698	24.6246	23.9944	24.1755	NaN	23.3242	25.6213	NaN	NaN	24.0603	22.5732	23.5153	O00560-2;G5EA09	O00560-2;G5EA09	Syntenin-1	SDCBP
22.3342	NaN	NaN	NaN	NaN	NaN	NaN	NaN	NaN	NaN	NaN	NaN	Q96C92;Q96C92-4	Q96C92;Q96C92-4	Serologically defin	SDCCAG3
24.9693	25.052	24.0332	NaN	24.4735	24.204	23.8508	NaN	24.5597	23.688	NaN	23.6484	Q99470;K7ELI9	Q99470	Stromal cell-deriv	SDF2
24.7311	25.1923	24.4853	24.8779	25.2842	24.8956	NaN	25.869	25.4301	23.7362	24.1846	24.4566	Q9HCN8	Q9HCN8	Stromal cell-deriv	SDF2L1
24.8152	25.7987	25.2219	24.4703	26.1147	24.4271	27.3592	26.4625	26.5315	25.2099	25.7429	25.8823	Q9BRK5;Q9BRK5-	Q9BRK5;Q9BRK5-	45 kDa calcium-bi	SDF4
27.1207	26.8776	26.463	27.0301	26.8879	26.4752	26.0243	27.5978	27.7407	26.8383	27.0044	26.6231	P31040;P31040-2	P31040;P31040-2	Succinate dehydro	SDHA
23.8019	24.7251	24.6311	24.6438	24.9136	24.9035	NaN	25.258	26.4839	23.4822	24.9292	24.719	P21912;A0A087W	P21912;A0A087W	Succinate dehydro	SDHB
26.1509	25.8863	25.9509	26.292	25.4798	25.5835	26.2817	26.1411	NaN	25.6153	24.9502	24.6427	O95810	O95810	Serum deprivation	SDPR
23.7646	24.1268	23.566	23.2304	23.4529	23.551	23.5896	NaN	NaN	NaN	24.0402	NaN	Q96GA7;F8VYZ3;I	Q96GA7;F8VYZ3	Serine dehydratas	SDSL
24.2674	24.7041	24.7347	25.0147	24.6429	24.8578	25.0281	24.9037	25.5919	24.9649	24.4712	24.8385	P67812;H0YNG3;I	P67812;H0YNG3;I	Signal peptidase c	SEC11A
26.4277	26.72	26.0108	26.4293	26.3689	26.4356	26.2188	26.5703	26.1909	26.7393	26.5316	26.8419	P55735;A8MXL6;A	P55735;A8MXL6;A	Protein SEC13 hor	SEC13
22.9088	NaN	NaN	NaN	NaN	NaN	NaN	NaN	NaN	NaN	NaN	NaN	Q92503;K7EJ08;Q	Q92503;K7EJ08;Q	SEC14-like proteir	SEC14L1
24.0488	24.7147	24.556	23.611	24.6094	24.7561	24.1249	NaN	24.6323	23.8206	24.0202	24.0612	O76054;B3KRD8;(	O76054;B3KRD8;(	SEC14-like proteir	SEC14L2
25.4942	25.2507	24.8278	24.864	25.2177	24.5835	24.0441	25.0152	25.1105	24.0966	24.1834	25.2317	J3KNL6;O15027;O	J3KNL6;O15027;O	Protein transport	SEC16A
27.8621	27.8547	27.5125	27.4807	27.4746	27.1157	27.3829	26.4179	27.2888	26.5105	26.3971	26.3823	O75396;A0A087X	O75396	Vesicle-trafficking	SEC22B
28.987	28.9557	29.0763	28.7099	28.6787	28.9622	28.8563	28.8462	28.317	28.9119	28.5568	28.4363	Q15436;F5H365;C	Q15436;F5H365;C	Protein transport	SEC23A
26.7343	26.5082	26.5152	26.6125	26.3743	26.2738	26.3243	26.3332	26.0191	25.9783	25.7207	25.6569	Q15437;Q5QPE1;(	Q15437	Protein transport	SEC23B
27.741	27.4629	27.6453	27.8645	27.7701	27.6994	27.0086	27.6671	27.176	27.3699	27.3723	27.3684	Q9Y6Y8;Q9Y6Y8-2	Q9Y6Y8;Q9Y6Y8-2	SEC23-interacting	SEC23IP
25.1546	24.7643	25.3221	25.4303	25.1417	24.9064	24.9217	NaN	24.8278	24.826	24.6296	24.9447	O95486;O95486-2	O95486	Protein transport	SEC24A
24.8136	24.4134	24.4793	24.7843	24.7064	24.8293	24.3899	24.7607	24.6327	24.6959	24.5493	24.6467	O95487;O95487-3	O95487;O95487-3	Protein transport	SEC24B
27.2338	26.9923	27.0816	26.7628	26.6709	26.9054	27.0091	27.0804	26.398	26.7361	26.6363	26.6914	P53992;G5EA31;P	P53992;G5EA31	Protein transport	SEC24C
26.0116	24.7934	25.0079	24.7927	24.7818	24.7965	NaN	24.5426	25.1711	24.8226	24.7149	23.7269	O94855;O94855-2	O94855;O94855-2	Protein transport	SEC24D
28.3281	27.9361	28.2148	28.35	28.0952	28.3754	27.6591	27.903	27.6421	28.2785	28.2683	27.8998	O94979;D6REX3;(	O94979;D6REX3;(	Protein transport	SEC31A
27.2193	25.6647	26.0256	26.6653	25.8934	26.3008	26.6898	26.26	26.6484	26.6648	26.2635	25.9634	P61619;B4DR61;C	P61619;B4DR61;C	Protein transport	SEC61A1;SEC61A2
26.5729	25.8656	26.3304	26.6014	25.8008	26.0487	26.6309	26.6608	26.091	26.2454	26.3877	25.8573	P60468;S4R3B5	P60468	Protein transport	SEC61B
NaN	NaN	NaN	25.3623	NaN	NaN	NaN	NaN	NaN	NaN	NaN	NaN	P60059	P60059	Protein transport	SEC61G
26.851	26.1867	26.6288	26.8259	26.3146	26.4745	25.7144	26.9151	25.7131	26.4476	26.1936	26.114	Q99442;F8WF48;I	Q99442;F8WF48;I	Translocation pro	SEC62
24.4276	23.7894	24.0749	24.6608	23.7538	24.0304	24.3238	24.8288	24.7566	24.2889	24.3894	24.6409	Q9UGP8;A6PVC9	Q9UGP8	Translocation pro	SEC63
27.0789	26.417	26.5392	26.6641	26.3841	26.5	26.1613	26.6417	26.1476	26.7948	27.0906	26.5715	Q96EE3-1;Q96EE3	Q96EE3-1;Q96EE3	Nucleoporin SEH1	SEH1L
23.4262	23.9843	24.1537	23.7009	24.4527	24.2048	23.1032	NaN	NaN	NaN	NaN	NaN	Q9UBV2	Q9UBV2	Protein sel-1 hom	SEL1L
23.5686	NaN	23.1444	NaN	NaN	NaN	25.2308	24.4425	NaN	25.0633	23.7207	23.1726	Q14563;C9J9C4	Q14563	Semaphorin-3A	SEMA3A
22.6154	22.7476	22.6471	22.2444	22.5055	NaN	26.0232	23.5229	24.615	25.6327	24.6128	23.463	Q99985;Q99985-2	Q99985	Semaphorin-3C	SEMA3C
24.1338	25.0047	22.9076	24.0687	24.018	21.2761	27.4827	27.9552	25.9765	24.7462	24.2651	NaN	O75326;O75326-2	O75326;O75326-2	Semaphorin-7A	SEMA7A
NaN	NaN	NaN	NaN	NaN	NaN	NaN	NaN	NaN	NaN	NaN	23.1394	P04279;P04279-2	P04279;P04279-2	Semenogelin-1;Al	SEMG1;SEMG2
23.9186	23.4791	24.4107	24.8482	24.1787	24.1721	NaN	NaN	NaN	23.6452	24.2256	NaN	Q9H4L4;A0A087X	Q9H4L4;A0A087X	Sentrin-specific pr	SENP3;SENP3-EIF4
22.6509	23.7309	23.5055	23.055	24.0737	23.2545	NaN	NaN	NaN	23.5657	23.562	23.377	P49903;P49903-2	P49903;P49903-2	Selenide, water di	SEPHS1
25.5913	26.1139	26.2264	25.8096	26.2343	26.3173	26.5017	26.8123	25.7908	26.7017	26.6546	25.8143	Q9P0V9;E7EW69;	Q9P0V9;E7EW69;	Septin-10	SEPT10
28.7394	29.6781	29.6324	28.955	30.0305	29.8846	30.1545	30.3599	30.062	29.4332	29.902	30.0572	Q9NVA2;D6RGI3;I	Q9NVA2;D6RGI3;I	Septin-11	SEPT11
29.2141	30.1288	30.1831	29.4704	30.2243	30.4757	30.5531	30.7614	30.1444	30.111	30.1834	30.3161	Q15019;Q15019-2	Q15019;Q15019-2	Septin-2	SEPT2
23.2162	23.5397	23.7648	23.2874	23.7938	23.7131	24.1004	24.3028	24.5324	23.7459	23.5429	NaN	Q14141;B1AMS2;	Q14141;B1AMS2;	Septin-6	SEPT6
29.2454	30.1475	30.3321	29.5305	30.4188	30.5802	30.7663	30.6127	30.2285	30.3989	30.4878	30.2885	Q16181;E7ES33;E	Q16181;E7ES33;E	Septin-7	SEPT7
25.5901	26.1264	26.2435	25.5314	26.4203	26.2791	26.4437	26.5886	26.5061	26.3337	25.9711	24.9511	Q92599;Q92599-4	Q92599;Q92599-4	Septin-8	SEPT8
28.8824	29.394	29.4429	28.8629	29.5713	29.6944	29.3584	29.8323	29.4783	29.2967	29.2939	29.2387	Q9UHD8;Q9UHD8	Q9UHD8;Q9UHD8	Septin-9	SEPT9
28.8084	29.7209	29.2775	28.2956	29.3036	28.7301	29.079	30.5189	29.3894	27.7987	29.0279	29.064	Q8NC51;Q8NC51-	Q8NC51;Q8NC51-	Plasminogen activ	SERBP1
NaN	NaN	23.6819	NaN	NaN	NaN	NaN	NaN	NaN	NaN	NaN	NaN	P84101;F8WBN3;I	P84101;F8WBN3;I	Small EDRK-rich fa	SERF2
26.8709	NaN	26.4944	26.8387	25.5942	26.7551	NaN	NaN	NaN	26.9917	26.3186	NaN	Q9NRX5	Q9NRX5	Serine incorporato	SERINC1
NaN	NaN	NaN	22.4765	NaN	NaN	NaN	NaN	NaN	NaN	NaN	NaN	Q13530;Q13530-2	Q13530;Q13530-2	Serine incorporato	SERINC3
26.3325	27.4324	26.9473	26.1496	27.1288	26.6911	26.2976	25.8181	27.0857	26.4299	26.6859	26.9733	P30740;P30740-2	P30740;P30740-2	Leukocyte elastas	SERPINB1
NaN	24.5659	22.7887	NaN	24.6735	22.9654	22.777	NaN	24.9689	22.3428	NaN	23.9355	P05120	P05120	Plasminogen activ	SERPINB2
26.5015	26.8566	26.7648	25.9713	26.6524	26.8309	26.891	26.5161	26.496	26.7577	26.7154	26.6695	P35237;A0A024Q	P35237;A0A024Q	Serpin B6	SERPINB6
24.2107	24.492	24.1532	23.4892	24.0349	NaN	23.9307	NaN	24.2664	24.062	NaN	24.1538	P50452;P50452-2	P50452;P50452-2	Serpin B8	SERPINB8
22.8206	24.4054	NaN	NaN	23.8342	NaN	NaN	NaN	NaN	NaN	NaN	NaN	P50453	P50453	Serpin B9	SERPINB9
31.068	30.2592	30.6854	29.9751	30.1309	30.0837	30.7503	29.9624	29.6904	28.8352	28.4606	28.4587	P05121;P05121-2	P05121;P05121-2	Plasminogen activ	SERPINE1
24.8421	23.149	23.689	23.5828	23.2689	23.4091	27.4136	24.9882	25.7108	24.7709	24.5627	24.2142	P07093-2;P07093	P07093-2;P07093	Glia-derived nexir	SERPINE2

27.4171	29.0037	28.3657	27.9686	28.923	28.4609	29.5881	30.0116	30.5406	29.168	29.2464	29.6723	P50454;E9PKH2;E	P50454;E9PKH2;E	Serpin H1	SERPINH1
27.6048	27.3933	27.7147	27.6352	27.598	28.1145	28.2845	28.0397	26.9067	28.617	28.4714	28.7794	Q01105;Q01105-2	Q01105;Q01105-2	Protein SET;Protei	SET;SETSIP
23.1261	NaN	NaN	NaN	NaN	NaN	NaN	NaN	NaN	23.2206	NaN	NaN	Q86TU7;Q6NXR6;	Q86TU7;Q6NXR6;	Histone-lysine N-r	SETD3
22.0442	NaN	22.1121	21.8382	NaN	NaN	21.9368	NaN	NaN	21.9034	22.1328	NaN	Q8WTS6;D6RJA0	Q8WTS6;D6RJA0	Histone-lysine N-r	SETD7
NaN	NaN	21.7281	NaN	22.7151	NaN	NaN	NaN	NaN	NaN	NaN	NaN	Q6UXD5;A0A087\	Q6UXD5;A0A087\	Seizure 6-like prot	SEZ6L2
25.9787	26.0459	26.4461	26.1227	26.4851	26.4653	25.6614	25.8647	25.0233	26.3243	26.4782	26.8733	Q15637-6;Q1563	Q15637-6;Q1563	Splicing factor 1	SF1
26.68	25.2158	26.2014	26.7626	25.5553	26.4367	24.8775	NaN	NaN	26.3121	26.3792	24.8746	Q15459;Q15459-2	Q15459;Q15459-2	Splicing factor 3A	SF3A1
25.7998	25.9692	26.169	26.668	26.4271	26.5995	24.9956	26.7584	24.9917	26.8171	27.245	26.5221	Q15428;K7EMT0	Q15428;K7EMT0	Splicing factor 3A	SF3A2
27.2874	25.7344	26.5715	26.9892	26.1458	26.6659	25.7181	26.0359	25.506	27.0578	26.9977	26.8651	Q12874	Q12874	Splicing factor 3A	SF3A3
29.1299	27.5379	28.5067	29.3443	28.311	28.8671	26.5266	26.5839	26.2725	28.2841	28.4402	27.9895	O75533;H7C341;F	O75533	Splicing factor 3B	SF3B1
27.5101	27.4253	27.5267	27.3139	27.6523	27.4938	26.3802	27.8333	26.6315	27.4572	27.8986	28.2834	Q13435;A0A087W	Q13435;A0A087W	Splicing factor 3B	SF3B2
29.8531	28.8788	29.6466	29.9809	29.4961	29.745	28.4472	28.8719	28.1749	29.4587	29.6004	29.25	Q15393;Q15393-3	Q15393	Splicing factor 3B	SF3B3
24.2811	24.3435	23.6371	24.2376	NaN	23.6532	NaN	NaN	NaN	24.1938	24.2595	NaN	Q15427;Q5SZ64	Q15427;Q5SZ64	Splicing factor 3B	SF3B4
26.6077	27.16	26.7972	27.4527	27.5975	27.316	25.5164	26.5276	25.8469	26.8454	27.5604	27.234	Q9BWJ5	Q9BWJ5	Splicing factor 3B	SF3B5
24.9553	24.615	24.0392	24.6128	23.5015	24.0277	24.3485	NaN	23.9495	24.334	24.4713	24.9177	Q9Y3B4	Q9Y3B4	Splicing factor 3B	SF3B6
30.5989	31.2873	31.1529	30.1519	30.9287	31.0037	31.7019	31.5945	31.0335	31.1937	31.3468	31.1687	P31947;P31947-2	P31947;P31947-2	14-3-3 protein sig	SFN
30.2864	30.1426	30.1328	30.4006	30.4972	30.5058	29.1019	29.6573	29.2817	30.4423	30.8525	30.6285	P23246;P23246-2,	P23246;P23246-2	Splicing factor, pr	SFPQ
NaN	NaN	NaN	25.3239	NaN	NaN	NaN	NaN	NaN	NaN	NaN	NaN	O95562;Q5TIH2	O95562;Q5TIH2	Vesicle transport	SFT2D2
NaN	NaN	21.618	NaN	21.5576	NaN	NaN	NaN	NaN	NaN	NaN	NaN	Q587I9	Q587I9	Vesicle transport	SFT2D3
27.2071	27.5541	27.3761	27.6379	27.7482	27.3958	26.0692	27.5905	28.5673	24.7793	26.4366	26.9828	Q9H9B4;D6RFI0;D	Q9H9B4;D6RFI0;D	Sideroflexin-1	SFXN1
25.5449	25.3408	25.5935	26.0172	25.943	25.8109	24.6538	26.0527	26.9151	NaN	24.7078	25.1517	Q9BWM7;A0A0A(	Q9BWM7;A0A0A(	Sideroflexin-3;Sid	SFXN3
NaN	22.3114	NaN	21.7813	NaN	NaN	NaN	NaN	NaN	NaN	NaN	NaN	O43556;E9PEH6;C	O43556;E9PEH6;C	Epsilon-sarcoglyca	SGCE
24.2894	23.3087	23.6492	24.4062	23.5626	23.6802	24.3355	NaN	NaN	23.7715	23.1111	NaN	O95470;H0Y3V8;H	O95470	Sphingosine-1-ph	SGPL1
23.2587	23.2167	23.2809	23.2283	23.1777	NaN	NaN	NaN	NaN	23.3896	23.6492	23.3681	Q9BX95	Q9BX95	Sphingosine-1-ph	SGPP1
NaN	NaN	NaN	NaN	NaN	NaN	23.5244	23.9135	NaN	NaN	NaN	NaN	P51688;I3NI22;I3I	P51688	N-sulphoglucosan	SGSH
26.6017	26.0703	25.7042	25.7761	25.5121	25.5474	NaN	26.1349	25.8126	26.2656	26.0212	26.3015	O43765;K7EMD6;	O43765	Small glutamine-r	SGTA
22.1477	NaN	NaN	NaN	NaN	NaN	NaN	NaN	NaN	NaN	NaN	NaN	Q96EQ0	Q96EQ0	Small glutamine-r	SGTB
22.3439	NaN	NaN	NaN	21.4954	NaN	NaN	NaN	NaN	NaN	NaN	NaN	Q9UQQ2;R4GN84	Q9UQQ2;R4GN84	SH2B adapter pro	SH2B3
23.2992	22.8717	22.7521	22.7091	22.8999	NaN	NaN	NaN	NaN	NaN	22.9517	NaN	Q9H788;Q9H788-	Q9H788;Q9H788-	SH2 domain-conta	SH2D4A
NaN	NaN	NaN	20.3105	NaN	NaN	NaN	NaN	NaN	NaN	NaN	NaN	Q6ZV89;E5RGV2;E	Q6ZV89;E5RGV2;E	SH2 domain-conta	SH2D5
22.4873	24.2556	24.6008	22.8685	23.842	24.8869	24.2386	NaN	24.2467	24.7184	24.7464	25.0728	O75368	O75368	SH3 domain-bindi	SH3BGRL
27.6219	29.5817	28.9389	28.1852	29.4397	29.2021	29.7739	29.8976	29.6904	29.26	29.6918	29.4919	Q9H299;Q5T123;/	Q9H299;Q5T123;/	SH3 domain-bindi	SH3BGRL3
23.7996	23.6001	23.8641	23.768	24.0157	NaN	NaN	NaN	NaN	NaN	24.0931	NaN	Q9Y3L3;Q9Y3L3-2	Q9Y3L3;Q9Y3L3-2	SH3 domain-bindi	SH3BP1
25.9233	25.6022	26.3306	25.9911	25.4528	26.433	25.4111	NaN	NaN	26.2801	25.5203	25.2214	Q9P0V3;Q9P0V3-:	Q9P0V3;Q9P0V3-:	SH3 domain-bindi	SH3BP4
20.3406	NaN	NaN	NaN	NaN	NaN	NaN	NaN	NaN	NaN	NaN	NaN	Q7L8J4;Q7L8J4-2	Q7L8J4;Q7L8J4-2	SH3 domain-bindi	SH3BP5L
26.6335	27.3425	26.7341	26.4063	27.0392	26.9707	27.9911	28.0984	27.8278	26.7883	26.6337	27.808	Q99961;Q99961-3	Q99961;Q99961-3	Endophilin-A2	SH3GL1
26.8305	27.1093	26.8791	26.7352	26.8401	27.0658	26.8923	27.3069	27.0241	26.5626	26.4514	26.7586	Q9Y371;A0A087W	Q9Y371;A0A087W	Endophilin-B1	SH3GLB1
25.7359	26.2035	25.7794	25.7268	25.9569	25.9784	25.9047	26.2011	25.6898	25.7929	25.8814	25.7543	Q9NR46;B7ZC38;E	Q9NR46;B7ZC38;E	Endophilin-B2	SH3GLB2
25.8488	26.847	26.5476	25.457	26.6013	26.1946	27.0959	27.4809	27.0758	26.1571	26.009	26.6079	Q96B97;Q96B97-2	Q96B97;Q96B97-2	SH3 domain-conta	SH3KBP1
23.2945	23.4485	23.4488	23.1452	23.3392	23.6039	23.1648	NaN	NaN	NaN	NaN	NaN	Q5TCZ1;H0Y507;C	Q5TCZ1;H0Y507;C	SH3 and PX doma	SH3PXD2A
22.5045	NaN	NaN	NaN	NaN	NaN	NaN	NaN	NaN	NaN	NaN	NaN	Q9H0F6;Q9H0F6-:	Q9H0F6;Q9H0F6-:	Sharpin	SHARPIN
25.1133	24.9814	24.3141	24.1689	24.8698	24.5559	24.6866	23.9661	25.1114	24.3807	24.316	25.2746	P29353;P29353-2,	P29353;P29353-2,	SHC-transforming	SHC1
25.0769	24.3964	25.5834	25.7148	25.2257	25.8774	27.005	26.7666	26.531	26.6382	26.6047	26.7125	Q8NEM2;I3L3Z1	Q8NEM2	SHC SH2 domain-l	SHCBP1
23.8146	NaN	24.4272	24.1938	NaN	24.6382	NaN	25.8282	NaN	NaN	26.3165	NaN	P60896	P60896	26S proteasome c	SHFM1
NaN	NaN	NaN	NaN	NaN	NaN	21.8586	NaN	NaN	NaN	NaN	NaN	Q6UWI4	Q6UWI4	Protein shisa-2 ho	SHISA2
22.8529	22.6467	23.2446	NaN	22.6696	NaN	NaN	NaN	NaN	NaN	NaN	NaN	Q8TBC3;M0R2P6;	Q8TBC3;M0R2P6;	SH3KBP1-binding	SHKBP1;KCTD3
26.8636	26.2367	26.3229	26.2331	26.113	25.967	26.5216	26.2039	26.1739	27.0165	27.0922	26.2138	P34896;P34896-2,	P34896;P34896-2,	Serine hydroxyme	SHMT1
30.0109	29.893	29.5228	30.114	30.0351	29.5836	28.9315	29.879	30.1946	29.9653	30.5282	29.5956	P34897;P34897-3,	P34897;P34897-3,	Serine hydroxyme	SHMT2
23.5135	23.5753	23.9686	23.9152	24.1444	24.583	NaN	NaN	NaN	25.3414	25.2257	23.0095	Q9UQ13;Q9UQ13	Q9UQ13;Q9UQ13	Leucine-rich repea	SHOC2
23.3819	NaN	NaN	NaN	22.8411	NaN	24.2526	NaN	NaN	23.6784	24.0149	NaN	Q9UHJ6;A0A0B4J:	Q9UHJ6;A0A0B4J:	Sedoheptulokinas	SHPK;TRPV1
NaN	NaN	NaN	NaN	NaN	NaN	23.6068	NaN	NaN	NaN	NaN	NaN	Q9HAT2;Q9HAT2-	Q9HAT2;Q9HAT2-	Sialate O-acetyles	SIAE
NaN	NaN	23.1465	22.5477	22.872	22.8069	NaN	NaN	24.1693	22.7463	22.8852	23.6826	Q99720;Q99720-2	Q99720;Q99720-2	Sigma non-opioid	SIGMAR1
NaN	NaN	NaN	NaN	NaN	NaN	25.1547	NaN	NaN	NaN	NaN	NaN	Q9H173;D6REA1;I	Q9H173;D6REA1	Nucleotide exchar	SIL1
25.3425	24.2963	24.5619	24.8242	24.5607	23.8356	NaN	NaN	NaN	24.4559	24.5902	NaN	Q96ST3	Q96ST3	Paired amphipath	SIN3A
NaN	NaN	NaN	NaN	NaN	NaN	NaN	NaN	NaN	NaN	NaN	NaN	O75182-2;O7518:	O75182-2;O7518:	Paired amphipath	SIN3B
24.4774	23.8819	23.8424	23.6086	23.9007	23.9985	NaN	NaN	NaN	23.6986	23.079	23.2431	Q96FS4;F6RY50;E	Q96FS4;F6RY50	Signal-induced pr	SIPA1
25.7255	25.5928	26.0503	25.9247	25.2707	26.044	24.2809	25.5466	25.687	24.8969	24.9375	24.7148	O43166;O43166-2	O43166;O43166-2	Signal-induced pr	SIPA1L1
22.8936	23.0262	23.4884	22.8058	22.8991	23.2835	NaN	NaN	NaN	22.7423	NaN	NaN	O60292	O60292	Signal-induced pr	SIPA1L3
NaN	NaN	NaN	19.5633	NaN	NaN	NaN	NaN	NaN	NaN	NaN	NaN	Q96EB6;B0QZ35;E	Q96EB6;B0QZ35;E	NAD-dependent p	SIRT1
NaN	NaN	NaN	21.1517	NaN	NaN	NaN	NaN	NaN	NaN	NaN	NaN	Q9NXA8;Q9NXA8	Q9NXA8;Q9NXA8	NAD-dependent p	SIRT5

NaN	NaN	NaN	NaN	NaN	NaN	NaN	NaN	NaN	22.7213	23.5355	NaN	Q8N6T7;Q8N6T7-	Q8N6T7;Q8N6T7-	NAD-dependent p	SIRT6
NaN	NaN	NaN	23.5631	NaN	NaN	NaN	NaN	NaN	NaN	NaN	NaN	Q8WVK7;J3QL03;.	Q8WVK7;J3QL03;.	Spindle and kinet	SKA2
25.9466	NaN	NaN	27.0024	23.8738	NaN	NaN	NaN	NaN	NaN	NaN	NaN	Q8IX90;Q8IX90-3	Q8IX90;Q8IX90-3	Spindle and kinet	SKA3
25.1484	24.7573	25.0649	24.8059	25.4981	24.97	24.3871	23.9494	24.6451	24.9813	25.0071	24.949	P42285;H0Y8U3;H	P42285	Superkiller viralici	SKIV2L2
27.0505	27.2152	26.3929	25.9862	26.8017	NaN	26.7533	26.9453	NaN	26.3973	26.7979	26.3276	P63208;E5RJR5;E7	P63208;E5RJR5;E7	S-phase kinase-as	SKP1
22.5906	23.0232	22.4199	21.2807	22.3544	NaN	NaN	NaN	NaN	NaN	NaN	NaN	Q9P270	Q9P270	SLAIN motif-conta	SLAIN2
27.1122	27.0201	27.9228	27.1548	27.5563	27.8908	27.1943	25.9192	27.5753	28.0876	27.7017	28.1815	P55011;P55011-3	P55011;P55011-3	Solute carrier fam	SLC12A2
26.6918	26.5265	26.8729	26.8178	26.562	26.9299	27.4534	27.3684	27.4804	27.3845	27.389	27.1301	Q9UP95;Q9UP95-	Q9UP95;Q9UP95-	Solute carrier fam	SLC12A4
NaN	22.4981	22.7191	NaN	NaN	NaN	NaN	NaN	NaN	NaN	NaN	NaN	Q9UHW9;Q9UHW	Q9UHW9;Q9UHW	Solute carrier fam	SLC12A6
22.8663	21.9937	22.9071	22.6863	22.2608	22.7499	22.8426	NaN	NaN	23.4868	22.7978	NaN	Q9Y666;A0A0U1R	Q9Y666;A0A0U1R	Solute carrier fam	SLC12A7
21.1885	22.3378	21.8737	22.0811	21.7982	NaN	NaN	NaN	NaN	21.2815	NaN	NaN	Q9BXP2;Q9BXP2-:	Q9BXP2	Solute carrier fam	SLC12A9
23.3814	NaN	NaN	23.3317	NaN	NaN	NaN	NaN	NaN	NaN	NaN	NaN	Q8N697	Q8N697	Solute carrier fam	SLC15A4
29.7686	28.453	29.3921	29.7261	28.4925	29.7492	28.9177	27.8535	28.5825	29.7188	29.0381	29.1597	O15427;J3QQV2;J	O15427	Monocarboxylate	SLC16A3
23.7372	NaN	23.4815	23.1444	NaN	23.5982	NaN	NaN	NaN	22.883	NaN	NaN	O60669;F8VS95;F	O60669	Monocarboxylate	SLC16A7
23.0871	NaN	NaN	NaN	NaN	NaN	NaN	NaN	NaN	NaN	NaN	NaN	Q9NRA2;Q9NRA2	Q9NRA2;Q9NRA2	Sialin	SLC17A5
23.2947	NaN	23.5629	23.6433	NaN	23.5463	NaN	NaN	NaN	NaN	NaN	NaN	O60779;O60779-2	O60779	Thiamine transpo	SLC19A2
24.5142	23.5619	25.4431	24.744	24.0454	25.8131	23.5093	23.9693	NaN	25.2478	24.4196	24.3431	P43007;P43007-2	P43007;P43007-2	Neutral amino aci	SLC1A4
30.7509	30.0362	30.7842	30.7764	30.0559	30.9606	30.3236	29.7961	30.2136	31.4253	30.7543	31.2175	Q15758;M0QXM4	Q15758;M0QXM4	Neutral amino aci	SLC1A5
24.5361	22.9789	24.2888	24.271	23.5359	NaN	24.1013	NaN	NaN	25.0275	23.9899	23.4378	Q8WUM9;H7BZK4	Q8WUM9	Sodium-depender	SLC20A1
24.4851	24.5333	24.6418	24.4226	24.7224	25.1684	24.1166	NaN	NaN	24.3398	24.1618	24.8731	Q08357	Q08357	Sodium-depender	SLC20A2
NaN	NaN	22.7996	NaN	NaN	NaN	NaN	NaN	23.2136	23.4735	23.2613	23.6687	Q9H015	Q9H015	Solute carrier fam	SLC22A4
NaN	NaN	NaN	22.4018	NaN	NaN	NaN	NaN	NaN	NaN	NaN	NaN	O76082;O76082-3	O76082;O76082-3	Solute carrier fam	SLC22A5
23.3813	22.9699	23.5564	23.4626	23.2451	23.6272	23.7439	NaN	NaN	24.6739	23.6718	NaN	Q9UGH3;Q9UGH3	Q9UGH3;Q9UGH3	Solute carrier fam	SLC23A2
24.5427	24.2696	24.5287	24.9485	24.1133	24.617	24.2654	25.0184	26.2364	22.5441	NaN	24.3168	P53007;B4DP62	P53007;B4DP62	Tricarboxylate tra	SLC25A1
24.884	24.1842	24.1143	24.9726	23.7177	23.9997	NaN	NaN	26.1758	NaN	NaN	NaN	Q02978;I3L1P8;Q	Q02978;I3L1P8;Q	Mitochondrial 2-o	SLC25A11
23.5356	23.6543	23.4429	23.8803	24.1136	23.842	NaN	NaN	24.0438	NaN	NaN	NaN	Q9UJS0;Q9UJS0-2	Q9UJS0;Q9UJS0-2	Calcium-binding n	SLC25A13
23.432	NaN	23.1577	23.8057	23.2475	NaN	NaN	NaN	24.2476	NaN	NaN	NaN	Q9H936;E9PJH7;E	Q9H936;E9PJH7;E	Mitochondrial glu	SLC25A22
26.8149	27.222	26.8335	27.2905	27.5795	27.0058	26.1321	27.2354	28.2764	25.2969	26.3616	27.0801	Q6NUK1;Q6NUK1	Q6NUK1;Q6NUK1	Calcium-binding n	SLC25A24
29.6427	29.5593	29.8955	30.3008	29.7645	30.0811	29.1074	29.5158	30.7666	28.5873	28.6784	29.6204	Q00325;Q00325-2	Q00325;Q00325-2	Phosphate carrier	SLC25A3
29.7889	29.1013	29.4514	30.0033	29.39	29.6291	29.1977	29.6254	30.6018	28.0774	28.5435	29.2691	P05141;Q9H0C2	P05141	ADP/ATP transloc	SLC25A5
26.4765	26.2804	26.2012	26.6792	26.1306	25.825	25.6683	25.6145	27.3039	24.2967	24.8729	25.7573	P12236	P12236	ADP/ATP transloc	SLC25A6
24.7651	24.9761	25.7497	25.1618	25.3938	25.8551	NaN	NaN	NaN	26.4975	26.3954	NaN	P50443;C9JAN6	P50443	Sulfate transporte	SLC26A2
23.9082	23.9998	24.8584	24.459	24.6098	25.0845	24.2138	NaN	24.1328	25.6794	24.8714	25.2486	Q9BXS9-3;G3XAC	Q9BXS9-3;G3XAC	Solute carrier fam	SLC26A6
NaN	NaN	NaN	21.8809	NaN	NaN	NaN	NaN	NaN	NaN	NaN	NaN	Q9BXS9;Q9BXS9-7	Q9BXS9;Q9BXS9-7	Solute carrier fam	SLC26A6
20.929	NaN	NaN	NaN	NaN	NaN	NaN	NaN	NaN	21.466	21.2216	21.1976	Q6P1M0;Q6P1M0	Q6P1M0;Q6P1M0	Long-chain fatty a	SLC27A4
28.1186	27.3862	28.631	28.559	27.3118	28.8342	28.4455	27.2108	26.9327	29.9954	28.799	28.1467	Q99808;Q99808-2	Q99808;Q99808-2	Equilibrative nucle	SLC29A1
23.1726	NaN	23.1495	23.0338	NaN	23.0139	NaN	NaN	NaN	23.9912	NaN	NaN	Q14542;Q14542-3	Q14542;Q14542-3	Equilibrative nucle	SLC29A2
30.6026	30.2845	31.1086	31.115	30.5694	31.6286	30.2437	30.0456	30.733	31.6861	31.2817	31.156	P11166;A6NL68;A	P11166	Solute carrier fam	SLC2A1
27.2485	27.2284	27.7508	27.7169	27.6118	27.9705	27.9407	27.1262	28.2671	28.4969	27.9979	28.2277	P11169;Q8TDB8;C	P11169;Q8TDB8;C	Solute carrier fam	SLC2A3;SLC2A14
21.4793	21.7411	21.5227	NaN	NaN	NaN	NaN	NaN	22.9248	21.8416	NaN	23.2425	Q9UGQ3;Q9UGQ3	Q9UGQ3;Q9UGQ3	Solute carrier fam	SLC2A6
22.4285	22.5913	22.9841	23.27	23.2173	NaN	23.2864	NaN	NaN	23.801	24.1684	23.929	Q9Y6M5	Q9Y6M5	Zinc transporter 1	SLC30A1
25.2557	24.9604	25.7123	25.7764	24.437	25.752	24.8185	NaN	NaN	NaN	NaN	25.0468	Q8NEW0;H0Y362	Q8NEW0;H0Y362	Zinc transporter 7	SLC30A7
NaN	NaN	NaN	NaN	NaN	NaN	NaN	NaN	NaN	25.8662	25.2435	NaN	O15431	O15431	High affinity copp	SLC31A1
NaN	NaN	21.5119	21.653	22.1211	NaN	NaN	NaN	NaN	NaN	NaN	NaN	L0R6Q1	L0R6Q1		SLC35A4
25.0236	24.6875	25.1122	24.9954	24.2075	24.8433	24.6014	NaN	NaN	24.9947	23.7283	NaN	Q8TB61;Q8TB61-3	Q8TB61;Q8TB61-3	Adenosine 3-phos	SLC35B2
24.304	23.5683	24.1295	24.378	23.6154	23.6992	NaN	NaN	NaN	23.4114	NaN	NaN	Q96K37;H7C1I0;C	Q96K37;H7C1I0	Solute carrier fam	SLC35E1
21.634	NaN	NaN	21.925	NaN	NaN	NaN	NaN	NaN	NaN	NaN	NaN	Q8IXU6;E9PJD1;Q	Q8IXU6;E9PJD1;Q	Solute carrier fam	SLC35F2
26.6951	25.1273	25.1843	25.6885	24.6235	25.2328	NaN	NaN	NaN	24.9173	24.8917	NaN	Q8N357;F8WCT7	Q8N357	Solute carrier fam	SLC35F6
23.2726	22.6923	23.3848	23.0823	22.4008	NaN	NaN	22.7751	NaN	23.5135	22.9224	NaN	O43826;U3KPU7;I	O43826;U3KPU7;I	Glucose-6-phosph	SLC37A4
27.3957	26.877	27.2725	27.0885	27.9118	27.1641	27.5568	28.1958	27.7006	27.3021	27.3109	28.3317	Q9H2H9;F8VX04;I	Q9H2H9;F8VX04;I	Sodium-coupled n	SLC38A1
27.5273	27.2695	27.3527	27.2881	26.6564	26.9348	27.3121	26.9129	27.8127	27.1775	26.0634	27.616	Q96QD8;F8VUY8;	Q96QD8;F8VUY8	Sodium-coupled n	SLC38A2
26.8702	27.61	27.4884	26.5223	27.2167	27.2685	29.3876	27.9295	27.3322	27.1433	26.0095	25.9145	Q9ULF5;E7ENT5;E	Q9ULF5	Zinc transporter Z	SLC39A10
26.2899	25.1539	25.4929	25.983	24.7741	25.1552	26.9277	23.2406	26.0615	27.6291	27.2354	NaN	Q15043;Q15043-2	Q15043;Q15043-2	Zinc transporter Z	SLC39A14
24.568	25.2528	24.9378	24.0434	24.4145	24.6998	25.1023	24.6149	NaN	24.958	23.8597	NaN	Q13433;K7EQ91;Q	Q13433	Zinc transporter Z	SLC39A6
26.7986	23.5735	25.3166	26.3033	24.0056	24.2458	NaN	NaN	NaN	25.5678	24.0828	NaN	Q92504;A0A087W	Q92504;A0A087W	Zinc transporter S	SLC39A7
32.798	32.1526	32.8981	32.9188	32.4025	33.1655	32.5153	31.6142	32.1801	33.2629	32.7939	32.7525	P08195-2;P08195	P08195-2;P08195	4F2 cell-surface ai	SLC3A2
NaN	NaN	NaN	22.7042	22.273	NaN	NaN	NaN	NaN	NaN	NaN	NaN	Q8IVJ1	Q8IVJ1	Solute carrier fam	SLC41A1
22.3646	NaN	22.8602	22.618	NaN	23.1829	NaN	NaN	NaN	23.2509	22.7899	22.7947	O75387;E9PJ68;O	O75387;E9PJ68;O	Large neutral ami	SLC43A1
28.5526	28.2786	29.3923	28.917	28.1923	29.7084	28.6174	27.47	28.7681	29.7727	29.4495	29.0279	Q8NBI5;Q8NBI5-2	Q8NBI5;Q8NBI5-2	Solute carrier fam	SLC43A3
25.3282	25.0696	25.9258	25.4043	24.9019	26.0533	25.6793	24.8667	25.7154	25.9438	25.2019	26.0432	Q8WWI5;Q8WWI	Q8WWI5;Q8WWI	Choline transporte	SLC44A1

26.427	25.9707	27.2338	27.316	26.1417	27.4143	25.4464	NaN	NaN	26.8502	26.0053	25.1833	Q8IWA5;Q8IWA5-	Q8IWA5;Q8IWA5-	Choline transport	SLC44A2
23.9788	23.4397	23.8121	23.7975	23.4732	24.057	NaN	NaN	23.85	24.1608	NaN	24.1733	Q96NT5;Q96NT5-	Q96NT5;Q96NT5-	Proton-coupled fc	SLC46A1
22.5484	NaN	21.9676	22.7701	22.983	22.4724	NaN	NaN	NaN	22.1974	22.84	23.2731	Q9BWU0;A0A087	Q9BWU0;A0A087	Kanadaptin	SLC4A1AP
22.4816	23.5489	23.4762	23.719	23.2213	NaN	NaN	NaN	25.5777	24.9595	24.8184	25.7857	P04920;J3KNG9;P	P04920;J3KNG9;P	Anion exchange p	SLC4A2
26.9016	26.8442	27.459	27.3929	26.9328	27.5595	27.3577	26.1037	27.0892	27.9602	27.2547	27.4862	Q9Y6M7-6;Q9Y6N	Q9Y6M7-6;Q9Y6N	Sodium bicarbona	SLC4A7
24.5778	NaN	23.964	24.4093	NaN	25.2704	25.772	NaN	NaN	25.8184	25.3472	25.3479	P53794	P53794	Sodium/myo-inos	SLC5A3
25.9658	24.9656	25.7961	26.0644	25.182	26.0648	25.8083	NaN	25.5117	26.7316	26.0219	25.9655	Q9Y289	Q9Y289	Sodium-depender	SLC5A6
24.2476	24.1386	24.8775	24.3087	24.3897	24.7005	25.1254	NaN	25.7644	25.9051	25.3104	26.0126	P31641;A0A087W	P31641;A0A087W	Sodium- and chlor	SLC6A6
27.2056	26.6058	27.5203	27.3815	26.102	27.2965	26.8423	26.2725	26.3922	27.0697	26.1928	26.2101	P30825;Q5JR49	P30825	High affinity catio	SLC7A1
27.5239	27.9556	28.2559	28.4679	28.0954	28.3642	27.4584	27.3235	27.8176	28.482	28.3454	28.2025	Q9UPY5;H0Y9F9	Q9UPY5	Cystine/glutamat	SLC7A11
26.4385	25.3367	26.0653	25.4312	25.4154	25.2835	25.5493	NaN	26.3164	23.8224	NaN	NaN	P52569;P52569-3	P52569;P52569-3	Cationic amino ac	SLC7A2
30.2359	29.3732	30.8704	30.5666	30.0287	31.0327	29.7977	29.1176	29.7245	30.9717	30.6645	30.4317	Q01650;A0A0C4D	Q01650	Large neutral ami	SLC7A5
24.2005	23.7261	24.7417	24.6604	24.3458	24.8096	24.1027	NaN	24.4147	25.1962	24.6984	NaN	Q92536;H3BSB6;H	Q92536	Y+L amino acid tra	SLC7A6
NaN	NaN	NaN	NaN	NaN	NaN	NaN	NaN	NaN	NaN	NaN	22.7792	Q96CW6;I3L4S1;A	Q96CW6;I3L4S1;A	Probable RNA pol	SLC7A6OS
23.2106	23.225	23.999	23.2953	24.0464	23.7588	22.9846	NaN	NaN	23.8852	23.7304	24.1316	P19634	P19634	Sodium/hydrogen	SLC9A1
28.4326	29.3903	29.7742	28.9066	29.4741	30.1383	28.5648	27.8842	27.5935	30.3366	29.4706	28.8881	O14745;J3QRP6;C	O14745;J3QRP6	Na(+)/H(+) exchar	SLC9A3R1
28.7916	29.8874	29.414	28.699	29.5952	29.6013	29.1117	28.8673	28.8787	29.8899	29.6946	29.4714	Q15599;H3BQS0;I	Q15599;H3BQS0	Na(+)/H(+) exchar	SLC9A3R2
23.9195	24.9209	24.2468	23.7859	24.6568	24.4669	23.6855	NaN	NaN	24.0807	24.3803	NaN	Q15599-2	Q15599-2	Na(+)/H(+) exchar	SLC9A3R2
25.7816	27.5868	26.9662	26.2116	27.2362	27.0724	26.9533	27.0217	26.3628	27.0407	27.3259	27.2662	Q15599-3;H3BN5	Q15599-3;H3BN5	Na(+)/H(+) exchar	SLC9A3R2
23.7352	NaN	23.9201	23.9308	NaN	24.2195	NaN	NaN	NaN	NaN	23.1984	NaN	Q92581-2;Q9258	Q92581-2;Q9258	Sodium/hydrogen	SLC9A6
23.05	NaN	23.1613	23.3438	NaN	NaN	NaN	NaN	NaN	22.7912	NaN	NaN	Q96T83;A0A087W	Q96T83;A0A087W	Sodium/hydrogen	SLC9A7
26.75	26.8663	26.0383	26.8898	26.8018	26.2219	25.3561	25.6255	25.4122	25.3505	25.4014	25.0002	Q9GZT3;Q9GZT3-	Q9GZT3;Q9GZT3-	SRA stem-loop-int	SLIRP
27.7813	28.0794	28.0295	27.9421	28.1488	28.1936	28.4301	28.5482	28.5534	28.5044	28.5256	28.5344	Q9H2G2;Q9H2G2	Q9H2G2;Q9H2G2	STE20-like serine/	SLK
23.4429	23.5377	23.2765	23.3791	23.7346	23.67	NaN	23.9496	NaN	23.0646	23.56	23.5544	Q9NWH9;H7C3F4	Q9NWH9;H7C3F4	SAFB-like transcri	SLTM
NaN	NaN	21.909	NaN	NaN	NaN	NaN	NaN	NaN	NaN	NaN	NaN	O95391	O95391	Pre-mRNA-splicin	SLU7
24.8195	23.6031	24.1319	24.3473	23.5686	24.1398	23.3545	NaN	NaN	23.8331	23.6784	NaN	P84022;P84022-3	P84022;P84022-3	Mothers against c	SMAD3
23.6781	23.4605	24.1301	23.155	23.5121	23.388	NaN	NaN	NaN	NaN	NaN	NaN	Q99717;Q15797;I	Q99717;Q15797;I	Mothers against c	SMAD5;SMAD1
24.1029	24.6867	24.5567	23.8488	24.4686	24.2734	NaN	NaN	NaN	23.7242	24.1049	24.3843	O00193;E9PM92;I	O00193;E9PM92;I	Small acidic prote	SMAP;C11orf58
22.942	NaN	22.6939	22.6065	NaN	22.6014	NaN	NaN	NaN	NaN	NaN	NaN	Q8IYB5;Q8IYB5-3;	Q8IYB5;Q8IYB5-3;	Stromal membrar	SMAP1
25.2973	24.281	24.9081	25.4242	24.4704	25.1531	NaN	NaN	NaN	24.4749	25.1434	24.3094	P51532;Q9HBD4;	P51532;Q9HBD4;	Transcription acti	SMARCA4
27.0594	25.8954	26.2027	27.2376	26.3881	26.3324	25.583	26.5975	25.4109	26.7709	26.8263	26.1463	O60264	O60264	SWI/SNF-related r	SMARCA5
23.2215	22.0468	22.321	23.8886	NaN	22.6611	NaN	NaN	NaN	23.4956	NaN	NaN	Q9H4L7;Q9H4L7-2	Q9H4L7;Q9H4L7-2	SWI/SNF-related r	SMARCAD1
22.3511	NaN	NaN	NaN	NaN	NaN	NaN	NaN	NaN	NaN	NaN	NaN	Q92922;F8WE13	Q92922	SWI/SNF complex	SMARCC1
24.9917	23.936	24.7177	24.9558	24.975	23.8386	25.9892	26.6792	NaN	25.2216	25.0855	25.1911	Q8TAQ2;Q8TAQ2-	Q8TAQ2;Q8TAQ2-	SWI/SNF complex	SMARCC2
24.7648	24.3363	24.6688	25.0419	24.8122	24.6477	NaN	NaN	24.0096	24.5599	24.8809	24.2277	Q92925;J3KMX2;E	Q92925;J3KMX2;E	SWI/SNF-related r	SMARCD2
NaN	NaN	NaN	NaN	NaN	NaN	NaN	NaN	NaN	NaN	NaN	20.5312	Q6STE5;Q6STE5-2	Q6STE5;Q6STE5-2	SWI/SNF-related r	SMARCD3
24.1773	NaN	23.8004	24.5881	23.9196	24.2148	NaN	NaN	NaN	23.7988	24.0639	23.9284	Q969G3;J3QKS7;E	Q969G3;J3QKS7;E	SWI/SNF-related r	SMARCE1
28.713	27.9002	28.1931	28.6863	28.5373	28.6197	27.1951	27.5655	26.1201	28.5573	28.6592	28.0236	Q14683;G8JLG1;H	Q14683;G8JLG1	Structural mainte	SMC1A
29.551	28.6839	28.8445	29.4769	28.9168	28.9322	28.385	28.4226	27.8709	29.3493	28.9511	28.386	O95347;O95347-2	O95347;O95347-2	Structural mainte	SMC2
30.4693	30.3145	30.4424	30.3563	30.3644	30.4476	29.3844	30.0492	29.2385	30.0181	29.9442	29.866	Q9UQE7	Q9UQE7	Structural mainte	SMC3
29.7943	28.768	28.918	29.4657	28.9649	29.057	28.7818	29.4064	28.546	29.2678	29.1832	28.5999	Q9NTJ3;E9PD53;C	Q9NTJ3;E9PD53;C	Structural mainte	SMC4
23.1656	NaN	22.7827	23.5608	NaN	22.8385	NaN	NaN	NaN	NaN	NaN	NaN	Q8IY18	Q8IY18	Structural mainte	SMC5
22.7847	NaN	NaN	22.8282	NaN	NaN	NaN	NaN	NaN	NaN	NaN	NaN	Q96SB8;Q96SB8-2	Q96SB8;Q96SB8-2	Structural mainte	SMC6
28.5798	27.1948	27.5091	28.4345	27.9205	27.6608	24.9651	24.096	25.4999	27.5368	27.5583	25.3672	A6NHR9;A6NHR9-	A6NHR9;A6NHR9-	Structural mainte	SMCHD1
19.5419	NaN	NaN	NaN	NaN	NaN	NaN	NaN	NaN	NaN	NaN	NaN	Q8TEV9;Q8TEV9-2	Q8TEV9;Q8TEV9-2	Smith-Magenis sy	SMCR8
25.1619	24.6778	24.5588	25.1904	24.9801	24.9687	NaN	NaN	NaN	24.3008	25.7804	27.3729	Q6IN85;G3V5Z3;C	Q6IN85;G3V5Z3;C	Serine/threonine-	SMEK1
27.5126	25.9293	26.5496	26.7469	25.9472	25.819	25.416	25.5788	NaN	24.2306	25.2842	NaN	J3KRA9;Q96Q15;C	J3KRA9;Q96Q15;C	Serine/threonine-	SMG1
22.5074	NaN	NaN	NaN	NaN	NaN	NaN	NaN	NaN	NaN	NaN	NaN	Q86US8;Q86US8-	Q86US8;Q86US8-	Telomerase-bindi	SMG6
24.9585	22.591	23.6629	24.5412	23.687	22.9486	NaN	NaN	NaN	22.94	23.6638	NaN	Q8ND04;Q8ND04	Q8ND04;Q8ND04	Protein SMG8	SMG8
24.9228	24.0455	24.8445	24.5733	24.2743	24.4603	23.8393	NaN	23.9822	24.0765	23.982	NaN	Q9H0W8;Q9H0W	Q9H0W8;Q9H0W	Protein SMG9	SMG9
20.9661	NaN	NaN	NaN	NaN	NaN	NaN	NaN	NaN	NaN	NaN	NaN	B2RUZ4;H3BS66	B2RUZ4;H3BS66	Small integral mer	SMIM1
27.1419	25.601	26.1716	27.206	25.4263	25.9312	24.4479	NaN	NaN	25.1692	25.449	23.3546	Q16637;E7EQZ4;C	Q16637;E7EQZ4;C	Survival motor ne	SMN1;SMN2
28.9455	29.29	29.1166	28.5206	29.2456	28.8395	29.3449	29.3162	29.3368	28.8823	29.2535	29.3551	P52788;H7C2R7;P	P52788;H7C2R7;P	Spermine synthas	SMS
25.792	26.0527	26.5474	26.4845	26.9154	26.5866	25.766	25.8948	25.7383	26.503	26.7317	27.1953	P53814-5;P53814	P53814-5;P53814	Smoothelin	SMTN
25.239	24.8572	25.0151	25.5046	25.0871	25.6074	24.333	24.3157	24.1218	25.7021	25.5499	25.4569	Q2TAY7;Q2TAY7-2	Q2TAY7;Q2TAY7-2	WD40 repeat-con	SMU1
25.1266	24.1076	24.6251	24.7721	NaN	24.6101	NaN	NaN	NaN	24.518	24.399	NaN	Q9HAU4;J3QQM4	Q9HAU4;J3QQM4	E3 ubiquitin-prote	SMURF2
22.694	NaN	NaN	23.0279	NaN	NaN	NaN	NaN	NaN	23.1765	23.1531	NaN	Q9NRG4;B0R0U3;	Q9NRG4	N-lysine methyltra	SMYD2
24.8873	24.5745	24.3252	25.1608	24.6131	24.567	NaN	NaN	23.3329	24.2966	24.6502	NaN	Q9H7B4;Q9H7B4-	Q9H7B4;Q9H7B4-	Histone-lysine N-r	SMYD3
NaN	NaN	NaN	22.8502	NaN	NaN	NaN	NaN	NaN	NaN	NaN	NaN	Q6GMV2;E2QRN5	Q6GMV2	SET and MYND do	SMYD5
27.7102	27.7087	27.9079	27.3371	27.4299	27.9692	26.9993	27.0309	27.7376	27.6061	27.1504	27.8978	O00161;O00161-2	O00161;O00161-2	Synaptosomal-ass	SNAP23

25.0198	26.0311	25.5283	24.1892	25.5592	25.3774	25.4677	24.6564	25.3997	24.8839	24.5584	25.513	O95721;C9JAF7	O95721;C9JAF7	Synaptosomal-ass	SNAP29
NaN	NaN	NaN	21.4119	NaN	NaN	NaN	NaN	NaN	NaN	21.9892	NaN	Q16533	Q16533	snRNA-activating	SNAPC1
23.0274	23.3141	22.7209	22.7453	22.9363	22.3893	NaN	NaN	NaN	22.2086	22.7496	NaN	O95295	O95295	SNARE-associated	SNAPIN
30.1967	29.9087	29.9167	30.137	29.9488	29.9805	29.4156	29.7882	29.646	29.7447	29.8473	29.9435	Q7KZF4;H7C597	Q7KZF4	Staphylococcal nu	SND1
23.9645	24.1765	24.1016	23.6321	24.0161	NaN	24.2086	NaN	NaN	NaN	24.5446	24.6645	Q96H20;Q96H20-	Q96H20;Q96H20-	Vacuolar-sorting p	SNF8
29.0839	27.8476	28.7066	29.2232	28.3555	28.7499	27.9396	27.6407	26.7571	28.7164	28.5662	28.3013	O75643;B4E0P5;C	O75643	U5 small nuclear r	SNRNP200
22.1144	NaN	NaN	22.387	NaN	NaN	NaN	NaN	NaN	NaN	NaN	NaN	Q8WVK2;B8ZZ98	Q8WVK2;B8ZZ98	U4/U6.U5 small n	SNRNP27
27.4051	26.0221	25.9659	26.5757	25.9324	25.7824	25.2638	NaN	24.1385	26.9618	26.669	25.0868	Q96DI7;Q96DI7-2	Q96DI7;Q96DI7-2	U5 small nuclear r	SNRNP40;DKFZp43
28.7915	28.2453	28.5691	28.8606	28.813	28.8249	28.1792	29.4529	27.5748	29.1772	29.5793	29.2993	P08621;P08621-2,	P08621;P08621-2,	U1 small nuclear r	SNRNP70
25.8439	26.5745	25.5657	25.9167	26.6326	25.8685	NaN	25.8964	24.9389	25.445	26.7876	26.3731	P09012;M0QZG7;	P09012;M0QZG7;	U1 small nuclear r	SNRPA
27.5043	26.7007	26.8407	27.9259	27.0784	26.9584	25.5227	24.8239	24.6777	27.2396	27.7054	26.1791	P09661;H0YMA0;I	P09661;H0YMA0;I	U2 small nuclear r	SNRPA1
28.4465	27.9863	28.0756	28.7163	28.3499	28.5194	27.6413	28.0899	27.7684	28.2915	28.735	28.612	P14678;P14678-2,	P14678;P14678-2,	Small nuclear ribo	SNRPB;SNRPN
26.895	25.1897	25.2741	27.2293	25.3805	25.8166	23.8671	24.5929	NaN	26.9033	25.4308	24.6619	P08579	P08579	U2 small nuclear r	SNRPB2
22.8582	23.1363	NaN	22.6239	22.3443	22.5589	NaN	NaN	NaN	NaN	NaN	NaN	P09234;A0A0A0M	P09234;A0A0A0M	U1 small nuclear r	SNRPC
29.3893	28.0638	28.7443	29.4177	28.4197	29.0603	27.8701	27.775	27.5169	29.4382	29.244	28.7874	P62314;J3QLI9;J3(	P62314;J3QLI9	Small nuclear ribo	SNRPD1
29.5236	27.949	28.2876	28.9036	28.1183	28.2274	27.532	28.0847	27.8613	28.2603	29.009	28.1971	P62316;P62316-2,	P62316;P62316-2	Small nuclear ribo	SNRPD2
27.2943	27.7455	27.4529	27.9932	27.9098	27.6136	26.5872	28.2388	27.8713	26.7438	28.2731	29.0211	P62318;P62318-2,	P62318;P62318-2,	Small nuclear ribo	SNRPD3
27.6889	27.3814	27.379	27.7421	27.6395	27.6803	25.9602	27.2332	27.1779	27.0357	27.7838	27.8264	P62304;A6NHK2	P62304	Small nuclear ribo	SNRPE
28.1852	27.8056	27.9506	28.4475	28.0829	28.168	26.7155	27.8321	27.0647	28.3192	28.4957	27.7352	P62306;A0A0B4J2	P62306;A0A0B4J2	Small nuclear ribo	SNRPF
27.2023	27.8974	27.1016	26.8066	28.3489	27.0331	27.2411	28.4321	27.5777	27.1679	28.0784	28.6064	P62308;A8MWD9	P62308;A8MWD9	Small nuclear ribo	SNRPG;SNRPGP15
23.5606	24.6951	24.9903	23.8617	24.9256	25.3304	24.0591	NaN	NaN	25.2824	24.945	25.3161	Q13884;Q13884-2	Q13884;Q13884-2	Beta-1-syntrophir	SNTB1
26.9611	26.9387	27.5978	27.3018	27.3196	27.7979	27.7683	27.4256	27.189	29.0292	28.4921	28.2238	Q13425;Q13425-2	Q13425;Q13425-2	Beta-2-syntrophir	SNTB2
23.8726	23.3872	23.6476	23.6524	24.0047	NaN	NaN	NaN	NaN	24.1021	24.868	NaN	O95149;H3BUB4;I	O95149;H3BUB4;I	Snurportin-1	SNUPN
24.7408	25.2812	25.0345	24.8907	25.468	25.4316	24.2693	24.7659	NaN	24.6239	24.9229	NaN	Q13573;G3V4X8;(	Q13573;G3V4X8;(	SNW domain-cont	SNW1
25.759	26.2113	25.8527	25.6131	26.1363	25.9194	25.8495	26.3416	26.3042	25.7105	25.9899	26.2628	Q13596;Q13596-2	Q13596;Q13596-2	Sorting nexin-1	SNX1
20.3137	NaN	NaN	NaN	NaN	NaN	NaN	NaN	NaN	NaN	NaN	NaN	Q9Y5W9;J3QRB9;	Q9Y5W9;J3QRB9;	Sorting nexin-11	SNX11
24.1268	25.2256	24.2666	24.4486	24.3915	23.9559	24.8406	24.0149	24.8763	24.4692	24.3984	24.9969	Q9UMY4;A0A087.	Q9UMY4;A0A087.	Sorting nexin-12	SNX12
22.7312	23.4303	22.7355	22.5392	22.8796	22.808	NaN	NaN	NaN	NaN	22.3879	NaN	Q9NRS6;E9PK26;E	Q9NRS6;E9PK26;E	Sorting nexin-15	SNX15
25.7221	25.9067	25.1069	25.3959	24.992	25.0201	24.4874	NaN	25.7165	24.5541	24.7738	NaN	Q15036;Q15036-2	Q15036;Q15036-2	Sorting nexin-17	SNX17
23.5479	23.7966	23.943	23.4114	23.6607	23.6457	23.6433	NaN	NaN	23.3574	23.4616	23.994	Q96RF0-2;Q96RF(	Q96RF0-2;Q96RF(	Sorting nexin-18	SNX18
27.6497	28.2328	27.8791	27.4832	28.1967	27.7682	27.7193	28.0411	28.0241	27.5101	27.6749	28.1654	O60749;D6RC15	O60749	Sorting nexin-2	SNX2
23.9359	23.9805	23.7999	23.7428	23.4356	23.7454	NaN	NaN	NaN	23.4423	23.2966	NaN	Q96L92;Q96L92-3	Q96L92;Q96L92-3	Sorting nexin-27	SNX27
28.1745	28.9838	28.2082	27.8715	28.1794	27.7071	28.0169	28.18	28.723	27.4564	27.5624	28.1385	O60493;O60493-2	O60493;O60493-2	Sorting nexin-3	SNX3
25.67	25.1151	25.4681	25.3396	24.8179	25.2531	25.3816	25.5102	25.4692	24.7233	24.8658	25.1534	Q8WV41;H3BPR3	Q8WV41;H3BPR3	Sorting nexin-33	SNX33
22.3478	22.0899	22.2251	22.5471	22.4231	22.1663	NaN	NaN	NaN	21.9462	21.8175	NaN	O95219;F8W9T3;(	O95219;F8W9T3	Sorting nexin-4	SNX4
24.9528	25.0776	24.9697	24.8962	25.2186	24.9259	26.1838	25.8721	25.7197	25.5227	25.8061	26.1329	Q9Y5X3;Q5QPE5;,	Q9Y5X3;Q5QPE5	Sorting nexin-5	SNX5
27.4888	28.4462	27.8519	27.4101	28.188	27.8629	27.828	27.7441	27.6106	27.8073	27.7803	27.8146	Q9UNH7;A0A0A0I	Q9UNH7;A0A0A0I	Sorting nexin-6;Sc	SNX6
23.8046	24.0245	23.3709	23.4005	23.5264	23.58	NaN	23.4496	24.0235	22.8947	NaN	24.1301	Q9Y5X2;C9J271;C	Q9Y5X2;C9J271	Sorting nexin-8	SNX8
26.7096	27.0425	26.8768	26.6486	26.9192	26.9552	26.8858	26.9277	27.1827	26.6576	26.5093	26.8802	Q9Y5X1;A0A087W	Q9Y5X1;A0A087W	Sorting nexin-9;Sc	SNX9
24.0089	22.7679	22.4787	23.5948	21.6529	NaN	NaN	NaN	NaN	22.8256	21.7841	NaN	P35610;B1APM4;I	P35610;B1APM4;I	Sterol O-acyltrans	SOAT1
NaN	NaN	NaN	NaN	NaN	NaN	NaN	NaN	NaN	NaN	19.0483	NaN	O14508;F8VS53;F	O14508;F8VS53;F	Suppressor of cyto	SOCS2
28.1067	29.3224	28.9005	28.1898	28.854	28.8405	29.0371	28.333	28.2009	28.9667	28.6498	28.2254	P00441;H7BYH4	P00441;H7BYH4	Superoxide dismu	SOD1
26.5226	28.8605	27.3245	26.6043	28.2346	27.1176	27.4214	28.8655	29.8502	26.6555	27.4417	28.5923	P04179;A0A0C4D	P04179;A0A0C4D	Superoxide dismu	SOD2
NaN	NaN	NaN	NaN	NaN	NaN	25.2221	25.5383	23.7407	NaN	NaN	23.083	P08294;M0R1V4	P08294	Extracellular supe	SOD3
NaN	21.9636	21.5133	NaN	NaN	NaN	NaN	NaN	NaN	NaN	22.2842	NaN	O94964;O94964-2	O94964;O94964-2	Protein SOGA1;N-	SOGA1
26.7636	26.2896	25.9542	26.7397	26.2191	25.9175	24.4274	26.4543	25.3996	25.9189	26.2557	26.1429	P18583-5;P18583	P18583-5;P18583	Protein SON	SON
23.0875	NaN	23.0569	22.5206	NaN	NaN	NaN	NaN	NaN	NaN	NaN	NaN	O60504;O60504-2	O60504;O60504-2	Vinexin	SORBS3
27.0782	26.2978	26.1514	26.6483	26.1748	26.034	27.238	26.3353	26.3759	27.2589	26.9326	26.4425	Q00796;H0YLA4;H	Q00796;H0YLA4	Sorbitol dehydrog	SORD
NaN	23.5465	23.6239	23.914	23.3084	23.115	24.0939	NaN	NaN	23.8013	23.4875	NaN	Q99523;Q99523-2	Q99523;Q99523-2	Sortilin	SORT1
24.117	22.9717	23.1968	23.5723	23.5217	23.4584	NaN	NaN	NaN	22.852	NaN	NaN	P23497;P23497-4,	P23497	Nuclear autoantig	SP100
22.5455	NaN	22.8789	23.1885	23.642	22.9742	NaN	NaN	23.3343	NaN	NaN	24.6367	Q02447;Q02447-5	Q02447;Q02447-5	Transcription facto	SP3
25.298	25.6173	25.5424	25.143	25.2094	25.4756	25.0469	NaN	24.4731	25.2231	25.0299	NaN	Q07617	Q07617	Sperm-associated	SPAG1
26.0029	24.8115	25.4789	25.9531	24.96	24.9895	24.0327	NaN	NaN	24.2447	24.9911	24.4419	Q96R06;J3KTQ0;K	Q96R06	Sperm-associated	SPAG5
NaN	NaN	NaN	NaN	NaN	NaN	NaN	NaN	NaN	NaN	22.4785	NaN	O75391;I3L0Q5;I3	O75391;I3L0Q5;I3	Sperm-associated	SPAG7
26.8895	26.6457	26.5182	26.283	26.6745	26.3955	25.8778	26.7817	26.673	25.2627	25.1644	25.9001	O60271-4;O60271	O60271-4;O60271	C-Jun-amino-term	SPAG9
NaN	NaN	NaN	NaN	NaN	NaN	21.2968	NaN	NaN	NaN	NaN	NaN	P09486;E5RK62;F!	P09486;E5RK62;F!	SPARC	SPARC
23.1083	NaN	24.0512	23.0604	NaN	23.3711	NaN	NaN	NaN	NaN	NaN	NaN	Q9UBP0;Q9UBP0-	Q9UBP0;Q9UBP0-	Spastin	SPAST
26.9404	25.4988	25.7574	26.7689	25.8941	25.7321	24.9032	25.6323	24.3128	25.5976	25.2715	25.3458	Q8NB90;Q8NB90-	Q8NB90;Q8NB90-	Spermatogenesis-	SPATA5
25.3877	24.2004	24.6703	25.5347	24.6244	24.4336	23.4835	23.8888	24.1687	24.3657	24.5685	23.1558	Q9BVQ7;Q9BVQ7	Q9BVQ7;Q9BVQ7	Spermatogenesis-	SPATA5L1
28.1764	26.3061	26.281	26.288	25.6893	25.3338	24.0093	25.1794	NaN	24.5733	24.4311	NaN	Q9NUQ6;A0A0A0	Q9NUQ6;A0A0A0	SPATS2-like prote	SPATS2L

24.3758	24.2544	23.7393	24.1483	23.4814	24.0019	23.8582	NaN	NaN	24.3769	23.8616	NaN	K7EJH0;K7EMX1;K	K7EJH0;K7EMX1;K7EJV2;K7ESQ2		SPC24
NaN	NaN	NaN	24.5603	NaN	NaN	NaN	NaN	NaN	NaN	NaN	NaN	Q8NBT2;Q8NBT2-	Q8NBT2;Q8NBT2-	Kinetochore prote	SPC24
21.7004	NaN	NaN	NaN	NaN	NaN	NaN	NaN	NaN	NaN	NaN	NaN	Q9HBM1	Q9HBM1	Kinetochore prote	SPC25
25.063	23.0879	23.9646	24.5292	23.921	23.7492	NaN	NaN	24.8573	24.399	23.8243	23.37	Q9Y6A9;Q9JBL1;X	Q9Y6A9	Signal peptidase c	SPCS1
26.7591	26.3517	26.7521	26.9302	26.4838	26.8794	26.882	26.9786	27.3635	26.915	26.7042	26.8831	Q15005;E9PL01;A	Q15005;E9PL01;A	Signal peptidase c	SPCS2
25.6303	24.5649	24.926	25.1942	24.41	24.7215	NaN	25.0026	25.7042	24.0926	24.567	24.4593	P61009	P61009	Signal peptidase c	SPCS3
21.7161	NaN	NaN	NaN	22.2526	NaN	NaN	NaN	NaN	NaN	NaN	NaN	Q96EA4;Q96EA4-:	Q96EA4;Q96EA4-:	Protein Spindly	SPDL1
22.2147	22.87	23.779	NaN	22.7921	23.2871	NaN	NaN	NaN	NaN	22.5702	NaN	Q5M775;Q5M775	Q5M775;Q5M775	Cytospin-B	SPECC1
23.3239	23.8368	23.6548	23.3838	23.8382	23.4157	NaN	NaN	22.6149	22.8205	23.017	22.6992	Q69YQ0;Q69YQ0-	Q69YQ0;Q69YQ0-	Cytospin-A	SPECC1L;SPECC1L-
22.3696	NaN	22.3737	22.3762	NaN	NaN	NaN	NaN	NaN	NaN	NaN	NaN	Q96JI7;C4B7M2;C	Q96JI7;C4B7M2;C	Spatacsin	SPG11
26.5876	25.8979	26.2043	25.4089	25.2924	25.8128	26.2093	26.3316	25.215	25.5415	25.5571	25.2798	Q8N0X7	Q8N0X7	Spartin	SPG20
NaN	NaN	NaN	22.1187	NaN	NaN	NaN	NaN	NaN	NaN	NaN	NaN	Q9NZD8;H0YML6;	Q9NZD8;H0YML6;	Maspardin	SPG21
25.0783	24.8715	24.8226	24.6932	24.4265	24.7213	24.8243	25.1089	25.2037	24.6846	24.5525	24.6072	O43291;K7EM91;(	O43291;K7EM91;(	Kunitz-type prote	SPINT2
24.0807	23.9924	23.8642	23.8421	23.4193	23.204	23.6859	23.6526	NaN	23.2814	23.2189	NaN	Q08AE8;Q08AE8-:	Q08AE8;Q08AE8-:	Protein spire hom	SPIRE1
22.1026	NaN	21.4679	22.0877	NaN	NaN	NaN	NaN	NaN	NaN	NaN	NaN	Q9H2V7;Q9H2V7-	Q9H2V7;Q9H2V7-	Protein spinster h	SPNS1
NaN	NaN	NaN	NaN	NaN	NaN	NaN	NaN	NaN	20.2679	NaN	NaN	Q8IVW8	Q8IVW8	Protein spinster h	SPNS2
NaN	NaN	25.9573	24.1018	NaN	25.7843	NaN	NaN	NaN	NaN	24.5221	NaN	Q8TCT8;H0YNA7;I	Q8TCT8;H0YNA7	Signal peptide pe	SPPL2A
25.2093	25.3115	24.9331	25.2182	25.1568	25.1123	25.096	24.8884	25.6048	24.9739	25.2062	25.7126	P35270	P35270	Sepiapterin reduc	SPR
NaN	NaN	22.7686	NaN	23.212	22.9773	23.1473	NaN	NaN	23.1559	22.6913	NaN	Q7Z699	Q7Z699	Sprouty-related, E	SPRED1
24.2223	24.6941	25.4605	24.8126	25.1777	25.7019	25.356	NaN	25.1125	26.351	25.9089	25.8555	Q7Z698;C9JG63;C	Q7Z698;C9JG63;C	Sprouty-related, E	SPRED2
22.0124	NaN	21.2346	21.388	NaN	NaN	NaN	NaN	NaN	NaN	NaN	NaN	O43597;A0A0A0N	O43597;A0A0A0N	Protein sprouty h	SPRY2
25.0951	25.1839	25.2711	25.0524	24.983	25.4331	24.6558	NaN	24.9529	24.438	24.5238	25.2248	Q9C004;A0A0C4D	Q9C004;A0A0C4D	Protein sprouty h	SPRY4
25.1502	24.4964	24.9807	25.234	24.4071	25.0957	24.6405	24.7292	24.4455	24.6855	24.2455	24.1736	Q5W111;Q5W111	Q5W111	SPRY domain-cont	SPRYD7
NaN	NaN	22.14	NaN	NaN	NaN	NaN	NaN	NaN	NaN	23.9099	NaN	P02549;A0A087W	P02549;A0A087W	Spectrin alpha cha	SPTA1
29.9627	29.9371	29.9008	29.9958	29.5685	30.0276	30.1085	29.8324	29.4099	30.04	29.7947	29.5619	Q13813;Q13813-2	Q13813;Q13813-2	Spectrin alpha cha	SPTAN1
30.636	30.3597	30.6692	30.623	30.2357	30.7464	30.6603	30.4842	30.3314	30.6445	30.4195	30.4264	Q01082;A0A087W	Q01082;A0A087W	Spectrin beta chai	SPTBN1
23.7192	23.6413	23.9089	23.5051	NaN	NaN	NaN	NaN	NaN	NaN	NaN	NaN	Q01082-3	Q01082-3	Spectrin beta chai	SPTBN1
NaN	NaN	23.5601	NaN	NaN	NaN	NaN	NaN	NaN	NaN	NaN	NaN	O15020;O15020-2	O15020;O15020-2	Spectrin beta chai	SPTBN2
25.9739	25.5179	25.7623	26.2092	25.6414	25.6206	26.0159	26.1418	26.7533	26.2435	25.9281	26.0979	O15269;O15269-2	O15269	Serine palmitoyltr	SPTLC1
24.5022	23.8389	23.8933	24.07	23.4863	23.6826	23.9647	24.0236	23.504	23.2365	NaN	NaN	O15270;H0YJV2	O15270;H0YJV2	Serine palmitoyltr	SPTLC2
26.6473	26.8441	26.702	27.0963	27.1173	26.956	25.4378	26.9897	28.1904	25.2871	26.2298	26.8013	Q9Y6N5;H3BNX3;	Q9Y6N5;H3BNX3	Sulfide:quinone o:	SQRDL
31.6385	30.6355	31.0815	30.4338	29.6692	30.313	31.0033	30.1562	30.1813	29.6663	29.1909	29.4557	Q13501;Q13501-2	Q13501;Q13501-2	Sequestosome-1	SQSTM1
22.8847	NaN	21.96	21.7389	NaN	21.996	NaN	NaN	NaN	NaN	NaN	NaN	Q9HD15;R4GMW	Q9HD15	Steroid receptor F	SRA1
24.4534	22.6781	24.1028	25.3521	23.684	23.7362	NaN	NaN	NaN	23.702	22.804	NaN	Q8N5C6;Q8N5C6-	Q8N5C6;Q8N5C6-	S1 RNA-binding do	SRBD1
26.3371	26.4659	26.8642	26.1264	26.1004	26.821	26.7512	25.4875	26.282	26.6671	26.4093	26.3761	P12931;P12931-2	P12931;P12931-2	Proto-oncogene t	SRC
24.7691	NaN	23.5623	24.3848	23.4896	NaN	NaN	NaN	NaN	23.6213	24.0141	NaN	Q8WXA9-2;Q8WX	Q8WXA9-2;Q8WX	Splicing regulator	SREK1
NaN	NaN	NaN	NaN	NaN	NaN	NaN	NaN	NaN	NaN	23.8368	NaN	Q8N9Q2	Q8N9Q2	Protein SREK1IP1	SREK1IP1
24.9319	25.0267	25.3901	24.5889	24.68	24.6247	24.0555	NaN	24.6875	24.7559	25.0107	NaN	Q7Z6B7;G5EA48;(	Q7Z6B7;G5EA48;(	SLIT-ROBO Rho G	SRGAP1
26.3542	26.7633	27.1683	25.8151	26.0418	26.2966	26.2927	24.0915	25.5156	26.4945	26.2851	26.0047	O75044;A2RUF3;A	O75044;A2RUF3;A	SLIT-ROBO Rho G	SRGAP2;SRGAP2B
25.7142	27.2814	26.3762	24.8071	26.5769	25.4881	29.9796	30.2425	28.1719	25.4538	25.8667	26.9394	P10124	P10124	Serglycin	SRGN
27.5764	28.0464	27.8529	27.4725	27.6444	27.8899	28.2771	27.086	26.8028	28.6446	28.1226	28.1951	P30626;C9J0K6;P3	P30626;C9J0K6;P3	Sorcin	SRI
28.1554	28.1097	27.7446	27.7428	28.0461	27.8216	27.3702	27.7671	27.862	27.8241	28.0708	28.0367	P19623;K7EKM4;I	P19623	Spermidine synth	SRM
27.2009	27.1384	26.8622	27.2157	26.9692	26.9311	26.9459	27.4544	26.5836	27.2639	26.7967	27.3774	P37108;H0YLA2;H	P37108;H0YLA2	Signal recognition	SRP14
26.2071	25.6441	26.3126	25.9074	26.0941	25.4695	25.3265	NaN	NaN	26.1357	26.7455	24.4036	P09132;A0A087W	P09132;A0A087W	Signal recognition	SRP19
28.4211	27.6931	27.7685	28.0206	27.6301	27.4698	26.6584	26.388	25.4002	26.1385	26.4195	26.3759	P61011;P61011-2	P61011;P61011-2	Signal recognition	SRP54
30.0828	28.3662	28.5171	29.9064	28.4029	28.6271	27.9761	27.5728	26.8229	28.2325	27.9063	27.4309	Q9UHB9;Q9UHB9	Q9UHB9;Q9UHB9	Signal recognition	SRP68
29.7364	28.4107	28.499	29.7019	28.5137	28.4597	27.798	27.9903	27.7532	28.5204	28.5034	27.9607	O76094;O76094-2	O76094;O76094-2	Signal recognition	SRP72
27.5665	27.5901	27.0095	26.8321	27.8994	27.2684	26.4401	28.0967	27.6438	27.2867	27.8473	27.3629	P49458;E9PE20;P	P49458	Signal recognition	SRP9
28.2172	26.8912	27.3502	28.1117	27.2213	27.5731	26.2296	27.5229	25.6986	27.0268	27.5868	26.8747	Q96SB4;Q96SB4-3	Q96SB4;Q96SB4-3	SRSF protein kina	SRPK1
22.2556	NaN	23.4471	22.7233	NaN	NaN	NaN	NaN	NaN	NaN	NaN	NaN	P78362;P78362-2	P78362;P78362-2	SRSF protein kina	SRPK2
27.914	27.1485	27.3807	27.7799	27.123	27.1569	27.2654	27.0795	26.8113	27.0569	26.9847	26.9862	P08240;P08240-2	P08240;P08240-2	Signal recognition	SRPR
27.218	26.4981	26.9829	27.3529	26.576	27.1223	27.1575	27.5388	26.1768	27.183	26.6136	26.2343	Q9Y5M8;H7C4H2;	Q9Y5M8;H7C4H2	Signal recognition	SRPRB
23.9774	NaN	23.3372	23.4551	22.9036	NaN	25.7992	24.0657	23.3667	24.3135	22.9703	NaN	P78539;P78539-3	P78539;P78539-3	Sushi repeat-cont	SRPX
22.8155	23.0138	NaN	NaN	23.0168	NaN	NaN	NaN	NaN	NaN	NaN	NaN	Q9GZT4;V9GYE8;I	Q9GZT4;V9GYE8	Serine racemase	SRR
25.2275	NaN	24.4918	25.0037	NaN	24.7779	NaN	NaN	NaN	25.7174	NaN	NaN	Q8IYB3;A9Z1X7;E!	Q8IYB3;A9Z1X7;E!	Serine/arginine re	SRRM1
27.626	27.0144	26.6258	27.3011	26.5247	27.1813	24.8346	25.4934	26.8927	26.0745	26.5635	26.9515	Q9UQ35;Q9UQ35	Q9UQ35	Serine/arginine re	SRRM2
28.055	26.53	27.2568	28.0733	27.1822	27.0079	25.5117	25.2101	25.1707	27.2723	27.4954	27.2937	Q9BXP5;Q9BXP5-.	Q9BXP5;Q9BXP5-.	Serrate RNA effec	SRRT
30.1886	28.9801	29.2074	30.1202	28.7733	29.4213	28.1936	28.8678	27.6397	29.4628	29.74	28.7079	Q07955;J3KTL2;Q	Q07955;J3KTL2;Q	Serine/arginine-ri	SRSF1
27.453	27.082	27.5592	27.9003	27.2832	27.8081	26.1855	27.5464	26.2328	27.7528	28.2781	27.737	O75494;O75494-3	O75494;O75494-3	Serine/arginine-ri	SRSF10
26.1342	24.9292	25.4911	26.1003	25.6381	25.7722	24.4854	NaN	23.2415	24.8775	25.7011	25.5313	Q05519;Q05519-2	Q05519;Q05519-2	Serine/arginine-ri	SRSF11

27.703	26.8998	27.3873	27.6115	27.3509	27.6152	27.4222	28.2849	27.3125	28.0036	28.7163	28.0184	Q01130;J3KP15;J3	Q01130;J3KP15;J3	Serine/arginine-ri	SRSF2
29.401	29.157	29.5938	29.9355	29.5161	29.7512	28.7321	29.4377	28.5734	29.9349	30.2125	29.7444	P84103;P84103-2	P84103;P84103-2	Serine/arginine-ri	SRSF3
25.5648	25.2085	25.3386	25.1645	25.5319	25.6721	NaN	NaN	NaN	25.368	25.7751	25.2099	Q08170;A0A0D9S	Q08170;A0A0D9S	Serine/arginine-ri	SRSF4
26.1975	25.8355	26.0712	26.7505	26.3901	26.4257	25.2513	26.5376	27.8697	25.8273	26.3986	26.7902	Q13243;Q13243-3	Q13243;Q13243-3	Serine/arginine-ri	SRSF5
29.6427	28.7729	29.3473	29.8317	29.2266	29.531	28.1292	29.1004	27.9741	29.7311	29.9203	29.2705	Q13247;Q13247-3	Q13247;Q13247-3	Serine/arginine-ri	SRSF6
28.8748	27.8825	28.4529	29.0671	28.3321	29.0323	27.7651	28.1973	27.6677	29.0285	29.1714	29.0625	Q16629;Q16629-4	Q16629;Q16629-4	Serine/arginine-ri	SRSF7
20.0922	NaN	NaN	NaN	NaN	NaN	NaN	NaN	NaN	NaN	NaN	NaN	Q9BRL6;Q9BRL6-2	Q9BRL6;Q9BRL6-2	Serine/arginine-ri	SRSF8
27.79	27.5899	27.0926	27.7794	27.5546	26.9728	26.2739	27.898	26.056	26.6744	27.3745	27.0394	Q13242;S4R3G0;H	Q13242;S4R3G0;H	Serine/arginine-ri	SRSF9
24.5681	24.8876	24.7405	24.1804	24.8106	24.1271	24.9949	NaN	25.1197	NaN	24.8947	25.323	Q9BYN0	Q9BYN0	Sulfiredoxin-1	SRXN1
28.3505	27.7319	28.3327	28.7133	28.2626	28.3024	27.5961	23.6118	26.2478	28.0667	27.9324	27.6905	P05455;E7ERC4;E9	P05455	Lupus La protein	SSB
27.4524	28.7161	27.6058	27.6861	28.3707	27.6873	28.396	28.2123	28.5693	28.1396	27.1945	27.6323	Q04837;A0A0G2JI	Q04837;A0A0G2JI	Single-stranded D	SSBP1
22.7898	NaN	22.7708	22.8299	NaN	22.6781	NaN	NaN	NaN	NaN	NaN	NaN	P28290;E9PHV5;P	P28290;E9PHV5;P	Sperm-specific an	SSFA2
25.9333	24.466	24.8943	25.3667	24.4887	24.532	NaN	NaN	25.2931	25.505	24.6838	NaN	P43307;C9J3L8;C9	P43307;C9J3L8;C9	Translocon-associ	SSR1
24.4998	23.5018	23.9163	24.4228	23.6208	24.0379	24.3475	24.0592	NaN	24.1553	NaN	NaN	Q9UNL2;C9JA28;C	Q9UNL2;C9JA28;C	Translocon-associ	SSR3
27.4174	27.8098	27.7928	28.0388	28.2805	27.9952	27.4105	28.0737	28.4843	27.7054	27.6813	27.7539	P51571;A6NLM8	P51571	Translocon-associ	SSR4
28.0144	26.7229	27.3301	28.1906	27.1223	27.5805	26.5557	26.4357	25.7879	27.7246	27.233	27.3651	Q08945;E9PMD4;	Q08945	FACT complex sub	SSRP1
21.4519	NaN	NaN	NaN	NaN	NaN	NaN	NaN	NaN	NaN	NaN	NaN	Q9Y2D8;S4R403;C	Q9Y2D8;S4R403;C	Afadin- and alpha	SSX2IP
27.3891	27.5516	27.7895	26.9286	27.2044	27.8784	28.3944	27.794	27.7064	28.1753	28.0259	28.2164	P50502;Q3KNR6;C	P50502;Q3KNR6;C	Hsc70-interacting	ST13;ST13P5
21.9163	NaN	21.7951	21.8528	NaN	NaN	24.6593	NaN	NaN	24.3043	22.2245	NaN	Q11201;E5RHV6;E	Q11201;E5RHV6	CMP-N-acetylneu	ST3GAL1
22.3225	NaN	NaN	22.7632	NaN	NaN	NaN	NaN	NaN	NaN	NaN	NaN	Q8WVM7;Q8WVM	Q8WVM7;Q8WVM	Cohesin subunit S	STAG1;DKFZp781D
23.3867	22.9921	23.139	23.3286	22.1403	23.0049	NaN	NaN	NaN	23.0896	23.1629	NaN	Q8N3U4;Q8N3U4	Q8N3U4;Q8N3U4	Cohesin subunit S	STAG2
26.2548	26.7394	25.7561	26.0835	25.2009	24.928	26.3897	24.6356	26.4213	25.9802	24.6046	25.8127	Q92783;Q92783-2	Q92783;Q92783-2	Signal transducing	STAM
23.7029	23.1373	23.0295	22.6121	22.1506	22.2578	NaN	NaN	NaN	22.1597	NaN	NaN	O75886;O75886-2	O75886;O75886-2	Signal transducing	STAM2
26.57	27.19	26.6305	26.2326	26.5075	26.5689	27.0474	27.1239	27.3279	26.3703	26.3604	26.9863	O95630;C9JK83;O	O95630;C9JK83;O	STAM-binding pro	STAMBP
24.294	24.0784	24.0436	23.7287	23.7553	24.0438	24.4194	NaN	NaN	23.7963	24.032	NaN	Q96FJ0;Q96FJ0-2;	Q96FJ0;Q96FJ0-2;	AMSH-like protea	STAMBPL1
20.853	NaN	NaN	NaN	NaN	NaN	NaN	NaN	NaN	NaN	NaN	NaN	Q96DR4;Q96DR4-	Q96DR4;Q96DR4-	StAR-related lipid	STARD4
27.4234	26.4763	26.895	27.0562	26.4894	27.1883	26.9534	26.621	26.6031	26.9366	26.9902	26.272	P42224;P42224-2	P42224;P42224-2	Signal transducer	STAT1
23.9426	23.1954	23.3668	23.1923	23.0777	23.3606	NaN	NaN	NaN	NaN	NaN	NaN	P52630;P52630-4	P52630;P52630-4	Signal transducer	STAT2
26.8975	26.2263	26.5895	26.7836	26.3176	26.5632	26.2604	26.1637	25.9602	25.8065	25.3914	25.9955	P40763;P40763-2	P40763;P40763-2	Signal transducer	STAT3
22.798	22.5343	22.6435	22.5328	23.0106	NaN	NaN	NaN	NaN	22.9236	NaN	24.1161	P51692;P42229;K	P51692;P42229;K	Signal transducer	STAT5B;STAT5A
24.4914	23.7081	24.3573	24.3237	23.9444	24.3115	23.6927	23.6888	24.2406	23.8596	23.9082	23.8654	P42226;P42226-3	P42226;P42226-3	Signal transducer	STAT6
27.5784	26.8311	26.5586	27.2383	27.0828	26.6207	26.1629	27.5405	26	26.5357	26.6923	26.6847	O95793;O95793-2	O95793;O95793-2	Double-stranded	STAU1
24.1383	NaN	22.7067	23.0221	22.9262	NaN	NaN	NaN	NaN	NaN	NaN	NaN	Q9NUL3;E5RJN7;A	Q9NUL3;E5RJN7;A	Double-stranded	STAU2
25.7119	26.5683	26.0026	25.4108	26.7459	25.426	31.3953	31.0123	29.9078	29.126	28.1363	28.701	P52823;P52823-2	P52823;P52823-2	Stanniocalcin-1	STC1
NaN	NaN	NaN	NaN	NaN	NaN	NaN	NaN	NaN	NaN	NaN	24.1618	Q9UHE8;Q6NZ63;	Q9UHE8	Metalloreductase	STEAP1
24.4762	24.3418	25.1432	24.6164	24.4946	25.8199	24.7346	NaN	24.7129	26.1027	24.6099	25.1563	Q658P3;Q658P3-3	Q658P3;Q658P3-3	Metalloreductase	STEAP3
22.3899	22.6606	22.5736	22.2163	22.5302	NaN	NaN	NaN	NaN	NaN	NaN	NaN	Q13586;G0XQ39;I	Q13586;G0XQ39;I	Stromal interactic	STIM1
30.6074	30.8979	30.707	30.2432	30.6358	30.6565	31.4226	31.2925	30.4334	30.9698	31.0722	31.0012	P31948;P31948-2	P31948;P31948-2	Stress-induced-ph	STIP1
29.0361	29.6042	29.4958	28.9745	29.6141	29.5851	28.6187	27.6264	28.0364	28.8407	28.9202	28.7945	O94804;H0YB71	O94804	Serine/threonine-	STK10
21.4517	NaN	21.685	NaN	NaN	NaN	NaN	NaN	NaN	NaN	NaN	NaN	Q15831;Q15831-2	Q15831;Q15831-2	Serine/threonine-	STK11
NaN	22.7297	22.3856	22.4956	NaN	22.8447	22.3091	NaN	NaN	NaN	NaN	NaN	O75716;B8ZZI5;B8	O75716;B8ZZI5;B8	Serine/threonine-	STK16
26.8964	26.2837	26.9597	26.4792	26.4941	26.8216	27.0791	25.8014	26.001	27.0605	26.7694	26.9005	Q9Y6E0-2;B4DR8	Q9Y6E0-2;B4DR8	Serine/threonine-	STK24
24.6347	24.5902	25.1445	24.8802	24.376	25.0519	NaN	NaN	NaN	24.3907	24.6937	NaN	O00506;O00506-3	O00506;O00506-3	Serine/threonine-	STK25
24.0481	22.7357	23.7901	23.5657	23.1296	23.809	NaN	NaN	NaN	23.9256	NaN	NaN	Q9P289;Q8NBY1;I	Q9P289;Q8NBY1;I	Serine/threonine-	STK26
21.7409	NaN	22.4702	21.6525	NaN	22.6085	NaN	NaN	NaN	NaN	NaN	NaN	Q13188;Q13188-2	Q13188;Q13188-2	Serine/threonine-	STK3
23.05	NaN	23.0526	23.1103	22.8904	23.7857	NaN	NaN	NaN	23.4526	23.5615	NaN	Q86UX6;Q86UX6-	Q86UX6;Q86UX6-	Serine/threonine-	STK32C
26.3458	26.0278	26.6232	25.7396	25.9024	26.1017	26.0724	26.0193	24.8954	26.2422	26.1104	25.7913	Q15208;Q9Y2H1;I	Q15208	Serine/threonine-	STK38
25.1362	24.479	24.9788	25.4211	24.6289	24.9374	24.37	25.1412	NaN	24.3363	24.785	24.9277	Q9UEW8;Q9UEW	Q9UEW8;Q9UEW	STE20/SPS1-relate	STK39
26.4721	25.8604	26.1643	26.4283	25.7076	26.3526	26.3614	26.0632	26.0054	26.4298	25.6128	25.3632	Q13043;Q13043-2	Q13043;Q13043-2	Serine/threonine-	STK4
29.3076	30.3296	29.9452	28.4873	29.5716	29.0292	29.1898	30.0585	29.2388	28.2102	28.684	28.9452	P16949;P16949-2	P16949;P16949-2	Stathmin	STMN1
NaN	NaN	NaN	27.0183	NaN	NaN	NaN	NaN	NaN	NaN	NaN	NaN	Q93045;E5RGX5;C	Q93045;E5RGX5;C	Stathmin-2;Stathr	STMN2
25.852	26.649	27.3802	26.4544	26.7923	27.635	27.8869	27.1994	28.8513	29.5152	28.401	29.1852	P27105;P27105-2	P27105	Erythrocyte band	STOM
25.547	27.3498	26.4445	26.2837	27.2345	26.5774	25.7935	27.501	28.3286	24.9137	25.6558	26.9892	Q9UJZ1;Q9UJZ1-2	Q9UJZ1;Q9UJZ1-2	Stomatin-like prot	STOML2
NaN	NaN	NaN	NaN	NaN	NaN	NaN	NaN	NaN	NaN	NaN	26.3529	Q8WXE9;Q8WXE9	Q8WXE9;Q8WXE9	Stonin-2	STON2
NaN	NaN	NaN	NaN	NaN	NaN	NaN	NaN	NaN	NaN	NaN	19.1259	Q7RTN6;J3QRC1;J	Q7RTN6;J3QRC1;J	STE20-related kin	STRADA
29.1249	28.6129	28.4346	28.469	28.4383	28.4571	28.3729	28.4736	27.8624	28.9897	29.043	28.3086	Q9Y3F4;Q9Y3F4-2	Q9Y3F4;Q9Y3F4-2	Serine-threonine	STRAP
24.1166	23.4353	23.8512	24.1137	23.4749	23.6804	23.7065	NaN	NaN	23.7433	23.6354	NaN	Q5VSL9;Q5VSL9-2	Q5VSL9;Q5VSL9-2	Striatin-interactin	STRIP1;STRIP2
24.6404	24.0571	23.9469	24.1077	24.4142	23.834	24.1049	24.5583	NaN	24.2823	24.6824	24.0048	O43815;O43815-2	O43815;O43815-2	Striatin	STRN
26.8165	25.7181	26.0083	26.0448	25.9399	25.9867	26.3444	25.8756	25.7528	26.1778	26.068	25.4765	Q13033;Q13033-2	Q13033;Q13033-2	Striatin-3	STRN3
26.8631	26.2154	26.1182	25.7112	25.9985	NaN	25.4537	NaN	26.3503	25.9913	26.7869	NaN	Q9NRL3;Q9NRL3-	Q9NRL3;Q9NRL3-	Striatin-4	STRN4

NaN	NaN	NaN	NaN	21.5116	NaN	NaN	NaN	23.9264	NaN	NaN	21.9329	P08842	P08842	Steryl-sulfatase	STS
26.4937	25.3529	25.8119	26.2542	25.0878	25.8471	25.3705	NaN	25.4384	25.8853	24.728	24.468	P46977;P46977-2,	P46977;P46977-2	Dolichyl-diphosph	STT3A
26.0796	25.0675	25.4585	25.9813	25.1948	25.4282	26.2121	25.3984	26.5399	25.5911	25.3029	26.093	Q8TCJ2	Q8TCJ2	Dolichyl-diphosph	STT3B
26.5103	27.2292	26.6628	26.2983	26.9628	26.5208	26.5106	26.7888	27.2426	26.9015	26.875	27.3122	Q9UNE7;Q9UNE7	Q9UNE7;Q9UNE7	E3 ubiquitin-prote	STUB1
NaN	NaN	NaN	NaN	NaN	NaN	NaN	NaN	22.3258	NaN	NaN	NaN	O60499;K7EQ84;K	O60499;K7EQ84;K	Syntaxin-10	STX10
24.4556	NaN	23.414	23.5065	NaN	NaN	NaN	NaN	NaN	NaN	NaN	NaN	Q86Y82;B1AJQ6	Q86Y82;B1AJQ6	Syntaxin-12	STX12
23.6973	24.0533	23.8927	24.0176	23.5961	23.9503	23.7509	23.9447	NaN	23.46	NaN	NaN	O14662;O14662-5	O14662;O14662-5	Syntaxin-16	STX16;STX16-NPEI
24.5578	23.9252	24.6534	24.4169	NaN	25.0044	24.4576	NaN	24.2582	25.3056	24.7818	25.0905	P32856;P32856-3,	P32856;P32856-3,	Syntaxin-2	STX2
25.383	24.7627	24.7313	25.3339	24.3195	NaN	24.7703	NaN	NaN	25.5235	25.1451	NaN	Q13277;A0A0J9Y\	Q13277;A0A0J9Y\	Syntaxin-3	STX3
28.0862	28.1637	28.4662	28.4133	28.3604	28.8058	28.3124	28.2619	28.449	28.891	28.5846	29.0236	Q12846;Q12846-2	Q12846;Q12846-2	Syntaxin-4	STX4
24.6759	24.7586	24.6715	24.7133	24.796	24.7756	24.1143	NaN	25.3893	24.6774	24.8083	25.1958	Q13190;Q13190-2	Q13190;Q13190-2	Syntaxin-5	STX5
25.5123	24.9631	25.1673	25.3534	24.9249	24.437	NaN	NaN	NaN	24.7816	24.9168	NaN	O43752;O43752-2	O43752	Syntaxin-6	STX6
26.6375	27.1358	26.7881	26.3733	26.5659	26.7897	27.0494	26.9932	27.3381	26.6579	26.6741	27.0255	O15400;O15400-2	O15400;O15400-2	Syntaxin-7	STX7
22.6587	22.1104	22.9225	22.5911	22.2473	22.7379	NaN	NaN	22.5963	NaN	21.7769	22.3845	Q9UNK0;I3L305;K	Q9UNK0;I3L305	Syntaxin-8	STX8
25.8611	26.6387	26.8006	25.9178	26.6357	27.0572	27.0345	25.9201	26.9954	27.4233	27.1418	27.4195	P61764;P61764-2,	P61764;P61764-2	Syntaxin-binding p	STXBP1
25.6333	26.2633	26.5164	25.999	26.5022	26.7502	23.5744	22.6053	24.1648	24.3469	24.4977	24.7083	Q15833;Q15833-2	Q15833;Q15833-2	Syntaxin-binding p	STXBP2
27.8351	27.9223	28.0907	27.7833	28.0086	28.3435	27.6262	26.8207	27.7091	28.3901	28.0252	28.0602	O00186;REV__H7	O00186	Syntaxin-binding p	STXBP3
21.828	NaN	NaN	21.8535	22.1941	NaN	21.9262	NaN	NaN	21.6598	21.9416	22.0528	Q5T5C0;Q5T5C0-2	Q5T5C0;Q5T5C0-2	Syntaxin-binding p	STXBP5
28.2562	28.3389	27.849	27.6223	28.2256	27.852	28.5707	28.8967	27.7341	28.0612	28.3041	28.7958	P53999;D6RC37;D	P53999	Activated RNA pol	SUB1
25.497	24.137	24.1876	25.6426	24.7368	24.7193	23.4555	NaN	23.8025	24.182	24.0566	23.4291	Q9P2R7;Q9P2R7-:	Q9P2R7;Q9P2R7-:	Succinyl-CoA ligas	SUCLA2
26.8478	26.8655	26.4409	26.9407	26.7456	26.3227	25.9047	27.0328	28.016	26.4311	26.4977	26.1155	P53597;H7C233	P53597	Succinyl-CoA ligas	SUCLG1
27.075	26.4198	26.3171	26.9248	26.441	26.6192	25.4089	25.9577	26.9216	26.6583	26.3394	25.7797	Q96I99;Q96I99-2;	Q96I99;Q96I99-2;	Succinyl-CoA ligas	SUCLG2
NaN	NaN	NaN	NaN	NaN	NaN	NaN	NaN	NaN	NaN	20.5941	NaN	Q8IWZ8	Q8IWZ8	SURP and G-patch	SUGP1
21.9981	NaN	22.8167	23.3453	NaN	NaN	NaN	NaN	NaN	NaN	NaN	NaN	Q8IX01;Q8IX01-3;	Q8IX01;Q8IX01-3;	SURP and G-patch	SUGP2
25.926	25.8298	25.2895	25.3148	25.6684	25.7105	25.4764	25.564	25.8252	25.1143	25.7307	26.1408	Q9Y2Z0;Q9Y2Z0-2	Q9Y2Z0;Q9Y2Z0-2	Suppressor of G2	SUGT1
NaN	NaN	NaN	NaN	NaN	NaN	22.4483	23.0367	NaN	NaN	22.7369	NaN	Q8NBK3;E9PF05;(	Q8NBK3;E9PF05;(	Sulfatase-modifyi	SUMF1
25.2511	26.1853	25.723	25.164	26.2867	25.8416	26.403	27.5031	26.5577	26.2458	26.5595	26.3649	Q8NBJ7;J3KQJ1;C	Q8NBJ7;J3KQJ1;C	Sulfatase-modifyi	SUMF2
25.1199	26.3965	25.8662	25.203	25.9441	25.8348	25.9398	26.3559	25.8689	26.0059	26.3356	26.6377	P63165;B8ZZN6;B	P63165;B8ZZN6;B	Small ubiquitin-re	SUMO1
28.3059	27.8002	27.423	27.2334	27.4065	27.5884	28.1507	NaN	NaN	28.281	27.5478	NaN	P61956;P61956-2,	P61956;P61956-2	Small ubiquitin-re	SUMO2
29.0987	27.8022	28.4521	29.2091	28.2124	28.7706	27.9474	27.6025	27.2131	28.9716	28.787	28.6673	Q9Y5B9;G3V5A4;(	Q9Y5B9	FACT complex sub	SUPT16H
24.5049	24.4429	23.8851	24.0576	24.8086	NaN	NaN	NaN	NaN	NaN	24.2577	NaN	P63272;D3DTZ5;J	P63272;D3DTZ5;J	Transcription elon	SUPT4H1
27.0734	26.3285	26.481	27.0182	26.5535	26.6017	25.1009	23.7308	23.8535	26.445	26.6607	26.2124	O00267;O00267-2	O00267;O00267-2	Transcription elon	SUPT5H
26.738	25.2193	26.1484	26.7322	26.729	26.343	23.5876	24.234	24.0226	25.9908	25.9839	26.0616	Q7KZ85;Q7KZ85-3	Q7KZ85	Transcription elon	SUPT6H
22.3623	NaN	NaN	22.3538	NaN	NaN	NaN	NaN	NaN	NaN	NaN	NaN	Q8IYB8;B1AR60	Q8IYB8	ATP-dependent R	SUPV3L1
27.638	26.8442	27.3476	27.3746	26.7762	27.4461	26.7961	NaN	27.148	27.0926	26.93	24.4038	O15260;Q5T8U5;(	O15260;Q5T8U5;(	Surfeit locus prote	SURF4
24.893	24.4459	25.11	24.9909	24.8135	NaN	25.7819	NaN	24.62	25.7533	25.7287	NaN	O60279;H7C2K7	O60279	Sushi domain-con	SUSD5
22.7359	22.0189	22.2562	23.605	22.3503	22.256	NaN	NaN	21.8347	22.5162	22.6302	NaN	Q15022;J3QQW9	Q15022;J3QQW9	Polycomb protein	SUZ12
24.2426	24.7478	25.0442	24.3135	24.9337	25.2357	24.8641	NaN	24.7753	24.4096	24.4031	24.9352	O95425-2;O9542	O95425-2;O9542	Supervillin	SVIL
23.7765	24.9103	25.3035	24.231	24.7779	25.6023	NaN	NaN	24.2638	NaN	24.2836	25.3103	Q8NHG7	Q8NHG7	Small VCP/p97-int	SVIP
27.2136	26.6857	27.3415	26.5293	27.0865	27.0593	26.1999	23.1966	25.244	26.6791	26.3203	25.8422	Q9UH65;E7EMB1;	Q9UH65;E7EMB1	Switch-associated	SWAP70
25.6694	24.9574	25.2414	24.7189	24.4193	24.7604	25.3738	25.0914	24.8824	24.8306	24.7207	24.8905	Q96A49	Q96A49	Synapse-associate	SYAP1
NaN	NaN	NaN	NaN	NaN	NaN	NaN	NaN	NaN	20.1463	NaN	NaN	Q15431;Q5VXJ5;A	Q15431;Q5VXJ5;A	Synaptonemal co	SYCP1
22.3398	NaN	22.08	22.3717	NaN	22.0783	NaN	NaN	NaN	NaN	NaN	NaN	Q6ZW31;Q6ZW31	Q6ZW31;Q6ZW31	Rho GTPase-activ	SYDE1
24.6295	NaN	23.9258	24.8752	23.9407	23.7726	NaN	NaN	NaN	24.3033	24.122	NaN	Q92797;A0A087W	Q92797;A0A087W	Symplekin	SYMPK
29.7756	29.6559	29.3203	29.7772	29.5386	29.4899	29.0733	28.9747	29.1694	29.2818	29.6063	29.7229	O60506-3;O6050	O60506-3;O6050	Heterogeneous n	SYNCRIP
28.0377	28.2741	28.0768	27.9462	27.841	28.3516	28.7364	NaN	28.0953	29.4403	28.565	NaN	Q8NF91;E7ENN3;(	Q8NF91;E7ENN3;(	Nesprin-1	SYNE1
25.0952	27.7236	27.06	25.0029	26.7836	26.5263	NaN	28.7428	28.4081	27.4681	28.6935	29.5123	Q8WXH0;G3V5X4	Q8WXH0;G3V5X4	Nesprin-2	SYNE2
23.486	23.0583	NaN	24.0566	23.249	NaN	23.4761	NaN	NaN	23.3598	NaN	NaN	O43759;O43759-2	O43759;O43759-2	Synaptogyrin-1	SYNGR1
27.5563	26.9495	27.6139	27.9608	27.5083	27.7964	27.0555	27.0224	27.1755	27.0639	26.5816	26.862	O43760;O43760-2	O43760;O43760-2	Synaptogyrin-2	SYNGR2
23.6281	23.8068	23.9174	23.881	23.9939	NaN	NaN	NaN	NaN	NaN	23.7465	NaN	P57105;A0A087X	P57105;A0A087X	Synaptojanin-2-bi	SYNJ2BP;SYNJ2BP-
23.6992	22.8523	23.565	23.3998	23.4538	NaN	NaN	NaN	NaN	NaN	NaN	NaN	O15061;A0A075B	O15061;A0A075B	Synemin	SYNM
26.4475	NaN	25.4612	26.148	24.4926	25.5559	NaN	NaN	NaN	26.2929	25.545	NaN	Q16563-2;Q1656	Q16563-2;Q1656	Synaptophysin-lik	SYPL1
25.3248	NaN	24.3736	NaN	NaN	NaN	NaN	NaN	NaN	25.1271	NaN	NaN	Q96C24;Q96C24-2	Q96C24;Q96C24-2	Synaptotagmin-lik	SYTL4
24.3851	25.4571	24.9612	23.8222	24.6086	24.2903	24.2525	NaN	24.5571	NaN	24.1982	NaN	Q7Z422-5;Q7Z422	Q7Z422-5;Q7Z422	SUZ domain-conta	SZRD1
23.6782	23.5571	23.5823	23.5891	23.5795	23.455	23.8191	NaN	NaN	23.7091	23.6044	NaN	Q15750;Q15750-2	Q15750;Q15750-2	TGF-beta-activate	TAB1
22.0156	NaN	NaN	NaN	NaN	NaN	NaN	NaN	NaN	NaN	NaN	NaN	Q9NYJ8;Q9NYJ8-2	Q9NYJ8;Q9NYJ8-2	TGF-beta-activate	TAB2
26.4487	25.8658	26.6861	24.4866	24.3185	24.0408	25.0138	25.4409	NaN	22.356	NaN	NaN	O75410;O75410-2	O75410;O75410-2	Transforming acid	TACC1
23.377	NaN	NaN	22.5564	NaN	NaN	NaN	NaN	NaN	NaN	NaN	NaN	O95359;O95359-2	O95359;O95359-2	Transforming acid	TACC2
23.2172	23.5217	23.0131	22.6769	23.1319	NaN	NaN	NaN	NaN	NaN	NaN	NaN	Q9Y6A5;H0Y8F2	Q9Y6A5	Transforming acid	TACC3
24.2859	24.028	23.5066	24.7206	24.1312	23.3844	NaN	NaN	NaN	NaN	NaN	NaN	Q9BSH4	Q9BSH4	Translational activ	TACO1

27.1556	26.7568	27.7717	27.3456	27.0852	27.969	27.0388	26.5101	26.7152	27.4597	26.7069	27.0905	P09758	P09758	Tumor-associated	TACSTD2
26.2104	26.5131	26.3247	26.3797	26.6773	26.3326	25.5979	26.8609	26.1371	26.4025	26.4532	26.8232	Q92804;Q92804-	Q92804;Q92804-	TATA-binding prot	TAF15
23.0832	NaN	23.7846	23.1716	NaN	NaN	NaN	NaN	NaN	NaN	NaN	NaN	Q15542;Q15542-	Q15542;Q15542-	Transcription initi	TAF5
20.3093	NaN	NaN	NaN	NaN	NaN	NaN	NaN	NaN	NaN	NaN	NaN	P49848;P49848-3	P49848;P49848-3	Transcription initi	TAF6
NaN	21.586	NaN	NaN	NaN	NaN	NaN	NaN	NaN	NaN	NaN	NaN	Q7Z7C8;A0A0A0N	Q7Z7C8;A0A0A0N	Transcription initi	TAF8
23.839	25.657	23.9933	23.7302	25.3529	NaN	NaN	NaN	NaN	23.9147	NaN	NaN	Q01995;H0YCU9;	Q01995;H0YCU9	Transgelin	TAGLN
28.5919	28.6827	28.5541	28.3287	28.3997	28.8056	29.5136	28.3995	28.167	28.2856	28.3077	28.5591	P37802;X6RJP6;P	P37802;X6RJP6;P	Transgelin-2	TAGLN2
23.5738	24.4836	24.3705	23.8867	24.895	24.2981	24.1364	24.7063	NaN	24.6016	24.1616	NaN	Q9UI15;C9J5W6;	Q9UI15;C9J5W6;	Transgelin-3;Trans	TAGLN3
29.7765	31.2207	30.5778	29.7478	31.2041	30.9604	29.91	30.1316	30.0495	30.3741	30.8122	31.0804	P37837;F2Z393;E	P37837;F2Z393	Transaldolase	TALDO1
22.3435	NaN	NaN	21.9197	NaN	NaN	NaN	NaN	NaN	NaN	21.4041	NaN	Q9C0D5;Q9C0D5-	Q9C0D5;Q9C0D5-	Protein TANC1	TANC1
NaN	NaN	21.7923	NaN	NaN	NaN	NaN	NaN	NaN	NaN	NaN	NaN	Q9HCD6;A0A087	Q9HCD6;A0A087	Protein TANC2	TANC2
26.6108	26.8677	27.3328	26.3551	26.3503	26.7129	26.3876	25.1511	25.6265	27.0051	26.2426	26.1115	Q7L7X3;Q7L7X3-3	Q7L7X3;Q7L7X3-3	Serine/threonine-	TAOK1
NaN	NaN	22.222	NaN	NaN	NaN	NaN	NaN	NaN	22.1114	NaN	NaN	Q9UL54-2;Q9UL5	Q9UL54-2;Q9UL5	Serine/threonine-	TAOK2
27.8529	27.7523	27.8058	27.0919	27.0459	27.2802	27.1768	26.6451	27.1546	27.1717	26.958	26.9044	Q9H2K8;G3V1Q8;	Q9H2K8;G3V1Q8	Serine/threonine-	TAOK3
24.203	24.6071	24.4045	24.4062	24.577	24.6292	24.4582	24.9862	24.9179	24.0093	23.9142	24.0576	Q03518	Q03518	Antigen peptide t	TAP1
24.0261	24.7597	25.0731	24.7549	24.7047	24.7165	24.5935	NaN	NaN	24.3316	23.9538	NaN	Q03519;A0A0G2J	Q03519;A0A0G2J	Antigen peptide t	TAP2
25.5869	26.7016	26.2395	25.9469	26.4339	26.3914	25.7704	25.0022	26.5305	26.0534	25.8018	26.2746	O15533;A0A0G2J	O15533;A0A0G2J	Tapasin	TAPBP
27.4794	26.7551	26.9945	27.559	27.0135	27.1761	25.9523	26.7979	26.5932	27.1054	27.1257	26.6633	Q13148;A0A087W	Q13148;A0A087W	TAR DNA-binding	TARDBP
30.1658	29.8486	29.9784	29.8605	29.9463	29.8916	30.2383	30.4246	30.1799	30.2523	30.2888	30.3118	P26639;P26639-2	P26639;P26639-2	Threonine--tRNA	TARS
21.1191	NaN	20.8843	22.0408	20.7899	NaN	NaN	NaN	NaN	NaN	NaN	NaN	Q9BW92;U3KQG	Q9BW92;U3KQG	Threonine--tRNA	TARS2
NaN	23.4087	NaN	NaN	23.2222	NaN	NaN	NaN	NaN	NaN	22.7241	NaN	Q6P1N9;H0YBX2;	Q6P1N9;H0YBX2;	Putative deoxyrib	TATDN1
29.2272	27.9666	29.291	27.629	25.9414	27.6734	29.3868	27.5624	25.9912	27.0818	26.0064	23.2791	Q86VP1-2;Q86VP	Q86VP1-2;Q86VP	Tax1-binding prot	TAX1BP1
25.1647	24.6114	24.6289	24.8182	24.8442	24.6147	25.6529	25.1131	25.0217	25.2895	25.2668	25.4844	O14907;A0A087X	O14907;A0A087X	Tax1-binding prot	TAX1BP3
25.8728	26.1028	27.0997	25.9624	26.6171	27.0441	25.3767	NaN	NaN	27.071	26.523	26.2425	Q9BXI6;Q9BXI6-2;	Q9BXI6;Q9BXI6-2;	TBC1 domain fam	TBC1D10A
26.0851	26.2276	26.8578	26.1186	26.6339	26.9302	25.9588	25.8145	25.772	27.2564	27.3434	27.3734	Q4KMP7;Q4KMP	Q4KMP7	TBC1 domain fam	TBC1D10B
23.0897	22.9142	22.9894	22.841	23.1882	NaN	NaN	23.2946	23.8151	23.4629	23.4439	23.4413	Q9NVG8;Q9NVG	Q9NVG8;Q9NVG	TBC1 domain fam	TBC1D13
26.5054	25.6077	26.1415	26.0502	25.5433	26.0253	25.8375	26.5796	25.6657	25.7135	25.8847	NaN	Q8TC07;Q8TC07-	Q8TC07;Q8TC07-	TBC1 domain fam	TBC1D15
23.987	23.1407	23.0268	23.3572	23.032	23.1532	23.3044	NaN	NaN	23.3876	23.1974	NaN	Q9HA65;Q9HA65-	Q9HA65;Q9HA65-	TBC1 domain fam	TBC1D17
23.5939	22.2387	23.0691	23.7063	22.7572	23.3198	22.4839	23.2787	NaN	22.8571	22.8671	NaN	Q9BYX2;Q9BYX2-	Q9BYX2;Q9BYX2-	TBC1 domain fam	TBC1D2
23.7873	24.0319	23.8601	23.5612	23.7576	23.5567	24.2082	NaN	NaN	24.1353	23.921	NaN	Q9NUY8;E9PGE5;	Q9NUY8;E9PGE5;	TBC1 domain fam	TBC1D23
23.1164	23.4898	23.4105	23.3894	23.216	23.0734	23.3991	NaN	NaN	23.0877	NaN	NaN	Q9ULP9;Q9ULP9-	Q9ULP9;Q9ULP9-	TBC1 domain fam	TBC1D24
23.0468	NaN	22.5209	23.5522	22.6577	NaN	NaN	NaN	NaN	22.8266	NaN	NaN	Q9UPU7;Q9UPU7	Q9UPU7;Q9UPU7	TBC1 domain fam	TBC1D2B
28.5475	27.0058	27.8209	27.9243	26.8897	27.0978	26.8324	27.1726	25.1356	26.2874	25.7435	25.0829	O60343;O60343-	O60343;O60343-	TBC1 domain fam	TBC1D4
22.8972	NaN	23.2922	22.7413	23.3481	NaN	25.0549	25.3677	NaN	NaN	NaN	NaN	Q92609;Q92609-	Q92609;Q92609-	TBC1 domain fam	TBC1D5
24.7571	23.9736	24.5086	24.4383	24.0045	24.1864	24.4383	23.976	24.3221	24.4643	24.3304	24.1822	Q66K14;Q66K14-	Q66K14;Q66K14-	TBC1 domain fam	TBC1D9B
28.9126	30.0844	29.5339	28.6072	29.5192	29.3446	29.6791	29.1329	29.6436	29.465	29.6106	29.7772	O75347;E5RJD8;E	O75347;E5RJD8;E	Tubulin-specific ch	TBCA
26.5777	26.754	26.9864	26.4985	26.0777	26.2889	26.4883	25.8397	26.4792	25.8875	26.4077	26.8283	Q99426;K7EP07;C	Q99426;K7EP07;C	Tubulin-folding co	TBCB
23.2981	23.8751	23.9774	23.1414	24.0038	23.6872	NaN	NaN	24.4447	NaN	NaN	NaN	Q15814	Q15814	Tubulin-specific ch	TBCC
28.4323	26.6374	27.4888	27.8894	26.8702	27.4181	26.8687	26.3244	26.8497	27.3085	27.0491	26.5919	Q9BTW9;J3KR97;	Q9BTW9;J3KR97;	Tubulin-specific ch	TBCD
26.0284	25.6217	25.6552	25.8171	25.708	25.4752	25.5328	NaN	25.2857	25.4053	25.9733	NaN	Q15813;Q15813-	Q15813;Q15813-	Tubulin-specific ch	TBCE
26.4874	25.467	26.0522	25.8281	25.185	25.5597	26.2685	25.2747	26.0767	25.4014	25.3425	25.422	Q9UHD2;F5H206;	Q9UHD2	Serine/threonine-	TBK1
22.3008	NaN	NaN	21.9968	NaN	NaN	NaN	NaN	NaN	NaN	NaN	22.2424	Q9BZK7;A0A0D9S	Q9BZK7;A0A0D9S	F-box-like/WD rep	TBL1XR1;TBL1X;TB
23.8639	23.8518	23.9608	23.7675	23.7254	NaN	NaN	24.3169	24.2049	24.6795	24.0999	NaN	Q9Y4P3;E9PF19;C	Q9Y4P3;E9PF19;C	Transducin beta-li	TBL2
26.2588	25.0505	25.6532	26.9863	25.4757	25.6085	NaN	23.5619	NaN	25.78	25.7527	23.9578	Q12788;J3KNP2;A	Q12788;J3KNP2	Transducin beta-li	TBL3
22.3204	NaN	NaN	22.8569	NaN	NaN	NaN	NaN	NaN	22.1086	22.159	NaN	P62380;Q7Z6U1;	P62380;Q7Z6U1;	TATA box-binding	TBPL1
26.6889	24.8633	25.2358	26.3661	24.3748	25.5172	NaN	NaN	NaN	24.7412	24.5427	NaN	Q969Z0;C9IZN7;Q	Q969Z0;C9IZN7;Q	Protein TBRG4	TBRG4
28.7788	27.338	28.093	28.2778	27.2063	27.6812	27.9296	NaN	NaN	27.6215	28.173	NaN	Q9Y4C2;Q9Y4C2-	Q9Y4C2;Q9Y4C2-	TRPM8 channel-a	TCAF1
22.6111	NaN	22.2123	22.6977	21.9198	22.1551	NaN	NaN	NaN	NaN	NaN	NaN	A6NFQ2;A6NFQ2-	A6NFQ2;A6NFQ2-	TRPM8 channel-a	TCAF2
24.3377	24.768	24.4197	24.3977	25.1847	24.8608	24.3151	24.284	24.1552	24.8666	25.1632	25.3494	P23193;P23193-2	P23193;P23193-2	Transcription elon	TCEA1
22.5915	23.1122	23.9802	22.7282	23.824	24.2423	NaN	NaN	NaN	NaN	23.2812	NaN	Q969E4;Q5H9L2;	Q969E4;Q5H9L2;	Transcription elon	TCEAL3;TCEAL5;TC
27.931	28.3264	27.8904	27.9674	28.0829	28.0644	28.2952	28.4379	27.695	28.6192	28.3212	28.4543	Q15369;E5RHG8;	Q15369;E5RHG8;	Transcription elon	TCEB1
26.251	27.1221	26.7058	26.4771	26.7272	26.5922	27.339	27.3122	27.175	27.2578	27.4419	26.5199	Q15370;Q15370-	Q15370;Q15370-	Transcription elon	TCEB2
NaN	NaN	22.6485	NaN	23.1569	NaN	NaN	NaN	NaN	23.0875	NaN	NaN	Q14241;A0A024R	Q14241;A0A024R	Transcription elon	TCEB3
25.7945	25.2983	26.0771	26.1446	25.9777	26.0969	24.5947	NaN	24.6844	26.4294	26.7905	26.5348	O14776;O14776-	O14776;O14776-	Transcription elon	TCERG1
23.2356	NaN	NaN	23.7535	NaN	NaN	NaN	24.1757	NaN	24.1194	23.6813	NaN	Q9BQ70;H3BQ53;	Q9BQ70;H3BQ53;	Transcription fact	TCF25
23.1503	23.008	23.4315	23.8072	23.012	23.9024	NaN	NaN	23.1343	23.1753	23.1378	NaN	Q13488;E9PM12;	Q13488;E9PM12;	V-type proton AT	TCIRG1
25.0438	25.0106	25.1928	25.4483	25.2922	25.1035	24.9715	NaN	24.4176	24.1284	25.3567	25.3394	Q13428;Q13428-	Q13428;Q13428-	Treacle protein	TCOF1
31.9428	31.6664	31.6202	32.0185	31.9066	31.7745	31.5787	31.9847	31.5346	32.0041	32.0231	31.7245	P17987;E7EQR6;E	P17987;E7EQR6;E	T-complex protei	TCP1
NaN	NaN	NaN	19.7679	NaN	NaN	NaN	NaN	NaN	NaN	NaN	NaN	Q9NUW8;G3V2F4	Q9NUW8;G3V2F4	Tyrosyl-DNA phos	TDP1
21.9022	NaN	NaN	NaN	NaN	NaN	NaN	NaN	NaN	21.0055	NaN	NaN	O95551;X6R5A3;	O95551;X6R5A3;	Tyrosyl-DNA phos	TDP2

27.0889	26.3064	26.9166	27.19	26.2386	27.1701	27.1865	27.2241	26.7484	27.5742	26.8347	26.8251	Q9NZ01;M0R3C3;	Q9NZ01	Very-long-chain e	TECR
25.1221	23.5347	24.0479	24.9554	23.8417	24.2648	23.6828	NaN	23.7828	24.9317	23.9843	NaN	Q9Y4R8;H3BRS3	Q9Y4R8;H3BRS3	Telomere length r	TELO2
25.3858	25.4183	26.3474	25.4743	25.2806	25.3054	25.9381	22.9682	NaN	25.7225	NaN	NaN	Q9P273;A0A0A0N	Q9P273;A0A0A0N	Teneurin-3	TENM3
22.1209	NaN	21.8018	21.8981	NaN	21.4586	NaN	NaN	NaN	NaN	NaN	NaN	Q15554;U3KQ35;I	Q15554;U3KQ35;I	Telomeric repeat-	TERF2
22.8556	NaN	NaN	NaN	NaN	NaN	NaN	NaN	NaN	NaN	NaN	NaN	Q9NYB0	Q9NYB0	Telomeric repeat-	TERF2IP
26.6161	26.8901	26.8163	26.3467	26.6339	26.8121	26.6172	26.6653	26.4814	26.5826	26.6508	26.597	Q9UGI8;Q9UGI8-:	Q9UGI8;Q9UGI8-:	Testin	TES
24.7366	23.4615	24.3619	25.2978	24.1697	24.4981	NaN	NaN	NaN	24.0548	24.04	NaN	Q9NXF1;Q9NXF1-	Q9NXF1;Q9NXF1-	Testis-expressed s	TEX10
24.9121	23.5226	23.9205	23.9883	NaN	23.9627	25.1489	NaN	24.0587	24.6046	23.9139	NaN	Q9Y6I9;C9JXQ7;C	Q9Y6I9;C9JXQ7;C	Testis-expressed s	TEX264
23.5908	NaN	NaN	23.0329	NaN	NaN	NaN	NaN	NaN	22.1923	NaN	NaN	Q5JUR7;A0A0C4D	Q5JUR7;A0A0C4D	Testis-expressed s	TEX30
25.9986	25.8819	25.3874	26.193	25.9446	25.503	24.5562	26.0999	26.8881	24.7067	25.0708	25.0784	Q00059;Q00059-2	Q00059;Q00059-2	Transcription fact	TFAM
22.6672	NaN	21.7473	22.0802	21.7904	NaN	NaN	NaN	NaN	NaN	22.3129	NaN	Q9H5Q4	Q9H5Q4	Dimethyladenosir	TFB2M
24.5816	24.1585	24.1328	24.7595	24.1713	24.0554	23.8925	23.9773	23.8996	23.8739	23.7196	23.842	Q12800;Q12800-2	Q12800;Q12800-2	Alpha-globin trans	TFCP2
27.8476	27.825	27.5557	27.1296	26.9465	27.2038	27.7418	27.2329	27.3035	27.5642	27.3043	27.3405	Q92734;Q92734-2	Q92734;Q92734-2	Protein TFG	TFG
23.9193	22.4049	23.2689	24.7227	23.3468	23.4029	NaN	NaN	NaN	23.1988	NaN	NaN	Q9UBB9;Q9UBB9	Q9UBB9	Tuftelin-interactin	TFIP11
21.8965	22.3984	23.6995	21.826	NaN	22.3884	27.8878	30.4118	28.7308	NaN	26.0084	25.3028	P10646;C9JBB3;C	P10646;C9JBB3	Tissue factor path	TFPI
32.5854	32.2118	32.6563	33.0001	32.4001	32.8732	31.5759	31.3338	31.1612	32.2478	31.5223	31.3434	P02786;G3V0E5;H	P02786;G3V0E5	Transferrin recept	TFRC
NaN	NaN	NaN	NaN	NaN	NaN	23.7283	NaN	NaN	NaN	NaN	NaN	P01137;M0R2S0	P01137	Transforming grov	TGFB1
22.8763	NaN	NaN	NaN	NaN	NaN	25.3535	NaN	NaN	NaN	NaN	NaN	P61812;P61812-2	P61812;P61812-2	Transforming grov	TGFB2
23.5401	26.6807	25.9416	23.7712	25.6905	24.3626	30.1216	30.3596	28.4882	26.0006	27.4056	27.1884	Q15582;H0Y8L3;S	Q15582;H0Y8L3	Transforming grov	TGFBI
NaN	NaN	NaN	NaN	NaN	NaN	NaN	NaN	NaN	22.4775	NaN	NaN	P36897;F8W1R9;I	P36897;F8W1R9;I	TGF-beta recepto	TGFBR1;ACVR1B;A
25.4358	25.4237	24.9425	24.9301	24.9388	25.1339	25.6215	26.8733	26.9104	25.8459	25.143	26.1105	P37173;P37173-2	P37173;P37173-2	TGF-beta recepto	TGFBR2
23.3949	23.1364	23.1209	23.5829	NaN	22.858	NaN	NaN	NaN	23.1608	22.8883	NaN	Q8WUH2	Q8WUH2	Transforming grov	TGFBRAP1
31.6549	30.862	31.4068	31.318	30.9389	31.3017	30.1994	30.2103	30.1685	29.9203	29.9141	29.8357	P21980;P21980-2	P21980;P21980-2	Protein-glutamine	TGM2
NaN	NaN	NaN	NaN	NaN	NaN	NaN	20.7897	NaN	NaN	NaN	NaN	Q08188	Q08188	Protein-glutamine	TGM3
26.2241	25.8498	25.5521	25.8574	25.3555	25.707	27.4613	25.4764	28.3553	26.1157	25.5466	24.2554	O43493;O43493-2	O43493;O43493-2	Trans-Golgi netwo	TGOLN2
24.1216	23.1627	23.6223	24.1118	23.1868	NaN	NaN	NaN	NaN	23.4184	NaN	NaN	Q6YHU6;Q6YHU6	Q6YHU6;Q6YHU6	Thyroid adenoma	THADA
25.5447	24.2228	24.4361	NaN	NaN	NaN	24.7795	NaN	NaN	25.1621	NaN	NaN	P07204	P07204	Thrombomodulin	THBD
28.3092	27.9827	28.8623	27.3967	27.876	28.2451	29.9427	29.1231	28.116	28.5704	28.4427	27.8481	P07996;P07996-2	P07996;P07996-2	Thrombospondin-	THBS1
NaN	NaN	NaN	NaN	NaN	NaN	23.7775	NaN	NaN	NaN	NaN	NaN	P35443;E7ES19	P35443;E7ES19	Thrombospondin-	THBS4
25.9704	24.7987	25.5485	26.2207	25.026	25.572	25.5129	26.0578	26.8247	25.7586	25.551	26.0153	Q8WUY1;H0YAR9	Q8WUY1	Protein THEM6	THEM6
21.5569	NaN	NaN	NaN	NaN	NaN	NaN	NaN	NaN	NaN	NaN	NaN	Q5TEJ8;H7C124;Q	Q5TEJ8;H7C124;Q	Protein THEMIS2	THEMIS2
23.207	NaN	NaN	22.9916	NaN	NaN	22.5182	NaN	NaN	22.817	NaN	NaN	Q9NWX6;E5RIQ8	Q9NWX6	Probable tRNA(Hi	THG1L
24.1026	NaN	23.4835	24.4212	23.3735	23.9003	NaN	NaN	23.3217	23.7309	23.5452	NaN	Q96FV9;A0A087W	Q96FV9;A0A087W	THO complex sub	THOC1
25.4619	NaN	23.9571	25.368	NaN	24.0138	NaN	NaN	NaN	24.7436	23.7689	23.4986	Q8NI27;A0A0C4D	Q8NI27;A0A0C4D	THO complex sub	THOC2
24.4341	24.2764	24.3213	24.2507	24.7184	23.9604	24.3153	24.5487	24.2938	24.5986	24.6605	24.3903	Q96J01;Q96J01-2	Q96J01;Q96J01-2	THO complex sub	THOC3
23.4849	NaN	23.0017	23.5643	22.5951	22.7045	NaN	NaN	NaN	NaN	NaN	NaN	Q13769;C9JXG5;F	Q13769;C9JXG5;F	THO complex sub	THOC5
25.4969	23.8833	23.8178	24.8042	24.4296	NaN	23.3119	NaN	23.714	24.4236	24.3489	23.9139	Q86W42;Q86W42	Q86W42;Q86W42	THO complex sub	THOC6
24.4759	24.3665	23.9415	24.4833	24.9095	24.2484	NaN	NaN	NaN	23.9623	24.3566	25.1108	Q6I9Y2;H7C5E3;A	Q6I9Y2	THO complex sub	THOC7
27.6061	27.686	27.301	26.9395	27.4792	27.3271	27.8781	27.6796	28.0352	27.9811	27.8779	28.206	P52888;K7EP46;K	P52888;K7EP46	Thimet oligopepti	THOP1
27.1528	27.2394	27.2625	27.3371	27.3879	27.529	26.1296	26.906	26.4674	27.156	27.6455	27.1628	Q9Y2W1	Q9Y2W1	Thyroid hormone	THRAP3
22.4763	23.813	23.1203	22.6068	23.0829	22.8222	27.1562	26.3571	26.8173	25.6132	23.6977	25.8763	Q6ZMP0;Q6ZMP0	Q6ZMP0;Q6ZMP0	Thrombospondin	THSD4
23.6486	24.1353	24.076	24.1534	24.672	24.5074	22.7477	NaN	NaN	23.7238	24.2811	24.4591	Q9NXG2;H3BNW	Q9NXG2;H3BNW	THUMP domain-c	THUMPD1
25.4399	24.5475	25.0025	25.4306	24.9234	25.0588	25.1718	NaN	25.374	25.7449	25.9105	25.3766	Q9BV44;H7C3J3;H	Q9BV44;H7C3J3	THUMP domain-c	THUMPD3
25.5064	24.5499	24.0091	24.7505	24.5818	24.3015	24.8099	NaN	NaN	24.5371	24.4176	NaN	Q01085-2;Q0108	Q01085-2;Q0108	Nucleolysin TIAR	TIAL1
NaN	NaN	NaN	NaN	NaN	NaN	20.0192	NaN	NaN	NaN	NaN	NaN	P35590	P35590	Tyrosine-protein k	TIE1
23.37	24.2965	23.463	22.9155	23.3721	22.9281	23.7479	NaN	23.5321	23.539	23.444	23.5926	Q9NQ88;A0A0U1I	Q9NQ88	Fructose-2,6-bispl	TIGAR
23.4806	25.1998	24.0648	23.9395	25.1044	23.8547	NaN	25.5076	26.2098	24.0021	24.9822	25.0618	P62072	P62072	Mitochondrial imp	TIMM10
23.9315	23.8138	NaN	23.637	NaN	NaN	NaN	NaN	NaN	NaN	NaN	NaN	Q9Y5L4;K7EIT2	Q9Y5L4;K7EIT2	Mitochondrial imp	TIMM13
26.1794	24.5814	24.9656	25.7487	24.9465	24.8927	23.6799	NaN	25.8563	24.3942	24.0019	NaN	O43615;M0QXU7	O43615;M0QXU7	Mitochondrial imp	TIMM44
NaN	23.3957	22.9039	23.3059	23.3679	NaN	NaN	23.9491	NaN	NaN	23.8385	NaN	Q3ZCQ8;Q3ZCQ8-	Q3ZCQ8;Q3ZCQ8-	Mitochondrial imp	TIMM50
NaN	23.8801	23.0353	22.3937	24.1632	NaN	NaN	24.6202	24.0615	NaN	NaN	24.4469	O60220	O60220	Mitochondrial imp	TIMM8A
NaN	22.1252	NaN	NaN	NaN	NaN	NaN	23.4961	NaN	NaN	22.2219	NaN	Q9Y5J9;E9PIR3;G	Q9Y5J9;E9PIR3;G	Mitochondrial imp	TIMM8B
23.8177	25.0796	24.4879	24.0239	24.8806	23.7707	24.6124	24.6568	25.2913	NaN	24.8413	25.2656	Q9Y5J7;G3V2F3;G	Q9Y5J7;G3V2F3;G	Mitochondrial imp	TIMM9
24.0789	26.3122	24.3268	23.6707	25.1491	23.7385	27.5227	27.4084	27.0881	25.3052	NaN	25.6372	P01033;Q5H9A7;	P01033;Q5H9A7;	Metalloproteinase	TIMP1
25.5311	26.9732	26.4089	25.3457	26.2444	26.2667	28.8268	28.9023	28.5232	25.8015	26.4514	27.0337	P16035;B4DFW2;	P16035;B4DFW2	Metalloproteinase	TIMP2
24.5694	23.6126	24.8141	24.9444	24.1417	24.4715	27.6747	26.9909	27.4394	27.7788	26.9436	26.5241	P35625	P35625	Metalloproteinase	TIMP3
23.7808	25.0903	25.6486	22.403	23.5609	24.0404	27.5137	26.6907	25.8378	24.3803	24.3116	24.5728	Q9GZM7;Q9GZM	Q9GZM7;Q9GZM	Tubulointerstitial	TINAGL1
25.4506	25.5933	25.5499	25.2932	25.2232	25.9945	25.7772	NaN	25.3388	25.7696	25.7443	26.1144	O75663;O75663-2	O75663;O75663-2	TIP41-like protein	TIPRL
24.587	23.9969	23.8984	24.334	23.8877	23.5289	24.0722	NaN	NaN		23.5323	NaN	Q5JTD0;Q5JTD0-2	Q5JTD0;Q5JTD0-2	Tight junction-ass	TJAP1
27.3376	27.3975	27.3512	27.0349	27.3576	27.4956	27.3913	27.0999	27.3057	27.3993	27.3125	27.393	Q07157-2;A0A087	Q07157-2;A0A087	Tight junction pro	TJP1

28.5444	28.8739	28.7903	28.3802	28.6549	28.7423	29.1297	28.9833	28.795	28.6847	28.5951	28.7924	Q9UDY2;Q9UDY2-	Q9UDY2;Q9UDY2-	Tight junction pro	TJP2
24.9119	24.7302	25.2098	25.2101	25.417	25.5907	24.5013	NaN	25.2457	24.8023	25.3851	25.5235	P04183;K7ERV3;K	P04183;K7ERV3;K	Thymidine kinase,	TK1
30.5465	31.7264	31.0415	30.6473	31.9482	31.2831	31.7139	31.8824	31.922	32.0233	32.2741	32.5892	P29401;P29401-2	P29401;P29401-2	Transketolase	TKT
27.1633	27.3635	27.6216	26.6742	27.1726	27.5207	28.2402	28.3016	28.6821	27.9238	28.0586	28.4211	Q6P9B6;H3BM75;	Q6P9B6;H3BM75;	TLD domain-conta	TLDC1
22.197	22.9278	22.3158	22.222	22.9342	NaN	NaN	NaN	NaN	22.3436	22.9841	NaN	Q04726;Q04726-3	Q04726;Q04726-3	Transducin-like er	TLE3;TLE4;TLE1
NaN	NaN	NaN	NaN	NaN	NaN	NaN	NaN	NaN	20.7759	NaN	NaN	Q9UKI8-2;Q9UKI8	Q9UKI8-2;Q9UKI8	Serine/threonine-	TLK1
NaN	NaN	22.5936	NaN	22.8846	NaN	NaN	NaN	NaN	NaN	NaN	NaN	Q86UE8;Q86UE8-	Q86UE8;Q86UE8-	Serine/threonine-	TLK2
31.0097	31.6102	31.5225	30.6547	31.3925	31.269	31.749	31.9156	31.579	31.2392	31.2514	31.5763	Q9Y490	Q9Y490	Talin-1	TLN1
23.0492	NaN	22.7918	23.152	22.6975	NaN	NaN	23.0557	NaN	23.1255	22.6473	NaN	Q9Y4G6;H0YMT1;	Q9Y4G6;H0YMT1	Talin-2	TLN2
NaN	NaN	NaN	NaN	NaN	20.8294	NaN	NaN	NaN	NaN	NaN	NaN	O60603	O60603	Toll-like receptor	TLR2
25.8853	NaN	25.3522	24.6367	NaN	24.5181	27.485	NaN	NaN	26.1519	24.9052	NaN	F8WF27;F8WBG6;	F8WF27;F8WBG6;	Transmembrane 4	TM4SF1
26.697	26.403	26.4874	26.223	25.9948	26.136	26.8301	25.0365	26.1697	26.3587	25.5678	26.8866	Q99805	Q99805	Transmembrane 9	TM9SF2
28.7793	28.044	28.6453	28.7774	27.8345	28.5819	27.963	27.4014	27.9508	28.1377	27.8385	26.9793	Q9HD45;Q5TB53	Q9HD45;Q5TB53	Transmembrane 9	TM9SF3
26.9739	25.4602	25.9289	26.5427	25.2139	25.915	26.2838	NaN	24.0241	25.8885	24.7659	25.0413	Q92544;A0A0C4D	Q92544;A0A0C4D	Transmembrane 9	TM9SF4
23.539	NaN	NaN	23.7046	NaN	NaN	NaN	NaN	NaN	NaN	NaN	NaN	Q96EY4;H0Y9X1;D	Q96EY4;H0Y9X1;D	Translation machi	TMA16
25.6606	27.6855	26.0826	25.6442	27.2645	25.8239	27.0217	27.9542	NaN	26.4632	27.172	26.6637	Q9Y2S6	Q9Y2S6	Translation machi	TMA7
22.9736	22.5074	22.8533	22.6517	23.2394	22.6933	22.7654	NaN	NaN	23.4161	NaN	NaN	Q969X1;C9JEN3;C	Q969X1;C9JEN3;C	Protein lifeguard	TMBIM1
24.1303	23.5651	24.8182	24.3278	22.6465	24.8698	NaN	NaN	23.9396	23.9749	NaN	23.3262	Q7Z403;K7ERH0;C	Q7Z403;K7ERH0;C	Transmembrane c	TMC6
22.2236	22.2259	22.788	22.7874	NaN	22.7626	NaN	NaN	NaN	NaN	NaN	NaN	Q8IU68;K7EJ72;Q	Q8IU68;K7EJ72	Transmembrane c	TMC8
25.0496	24.4017	24.517	25.0597	24.4459	24.6559	25.4576	25.2613	25.2347	25.4764	24.7466	NaN	Q9UM00;J9JIE6;Q	Q9UM00;J9JIE6;Q	Transmembrane a	TMCO1
23.506	NaN	NaN	NaN	NaN	NaN	NaN	NaN	NaN	22.7369	NaN	NaN	Q13445;K7EIN4;K	Q13445;K7EIN4;K	Transmembrane e	TMED1
30.1269	30.0335	30.1087	30.1958	29.9004	30.3011	29.2713	29.3722	30.0038	29.0964	29.2725	29.4503	P49755;G3V2K7	P49755	Transmembrane e	TMED10
27.1537	25.3617	25.9199	26.5386	25.117	24.9195	25.5381	NaN	24.1857	25.2571	24.5326	24.0558	Q15363;E7EQ72;F	Q15363;E7EQ72;F	Transmembrane e	TMED2
24.2907	23.5505	22.8139	24.605	23.3675	NaN	NaN	NaN	NaN	NaN	NaN	NaN	Q9Y3Q3;G3V1J9;F	Q9Y3Q3;G3V1J9;F	Transmembrane e	TMED3
26.9308	27.1074	26.6002	26.6946	27.0879	26.8684	25.4227	25.9426	26.5492	25.6822	25.7185	25.5383	Q7Z7H5;Q7Z7H5-	Q7Z7H5;Q7Z7H5-	Transmembrane e	TMED4
26.0742	25.7872	25.8875	25.9844	25.9252	26.0108	26.0828	26.5521	26.5908	26.3016	25.7562	25.8201	Q9Y3A6;M0R072;	Q9Y3A6	Transmembrane e	TMED5
28.2385	27.8748	27.7747	28.0016	27.3892	27.8927	26.9068	26.4189	26.5776	26.8064	26.6447	26.6768	Q9Y3B3	Q9Y3B3	Transmembrane e	TMED7
28.1943	28.0985	28.1141	28.3299	27.683	28.3117	27.7361	27.0412	28.1381	27.4672	26.9638	27.7641	Q9BVK6	Q9BVK6	Transmembrane e	TMED9
26.0216	25.6183	26.1047	25.7899	25.4031	26.0795	25.1943	25.1757	26.576	25.1449	25.2522	26.2102	Q9NUM4;C9JZ87;	Q9NUM4;C9JZ87;	Transmembrane p	TMEM106B
26.6071	26.0968	26.5889	26.8339	26.557	26.6579	26.9786	27.5041	28.1545	26.9675	27.0127	26.8224	Q9BVC6	Q9BVC6	Transmembrane p	TMEM109
NaN	NaN	NaN	22.7651	NaN	NaN	NaN	NaN	NaN	NaN	NaN	NaN	Q12893	Q12893	Transmembrane p	TMEM115
24.5579	25.5092	25.1733	24.6014	24.8757	25.2488	25.5083	24.8398	NaN	NaN	NaN	NaN	Q8N131;E9PKT4;C	Q8N131;E9PKT4;C	Porimin	TMEM123
NaN	27.1558	25.808	NaN	NaN	NaN	NaN	NaN	NaN	NaN	NaN	23.2701	Q9NPI0;J3QSZ6	Q9NPI0;J3QSZ6	Transmembrane p	TMEM138
NaN	NaN	NaN	22.0039	NaN	NaN	NaN	NaN	NaN	NaN	NaN	NaN	Q96B96;I3NI21;I3	Q96B96;I3NI21;I3	Promethin	TMEM159
NaN	NaN	NaN	NaN	NaN	NaN	NaN	NaN	NaN	NaN	NaN	20.5629	Q9NX61;K7EQE7;I	Q9NX61;K7EQE7;I	Transmembrane p	TMEM161A
23.7788	NaN	23.7435	23.7961	NaN	23.819	23.6315	NaN	NaN	24.0028	23.2574	NaN	Q9HC07;V9GY93;	Q9HC07;V9GY93;	Transmembrane p	TMEM165
22.1161	NaN	21.3166	NaN	NaN	NaN	NaN	NaN	NaN	NaN	NaN	NaN	Q7Z7N9;G3V185	Q7Z7N9;G3V185	Transmembrane p	TMEM179B
25.009	23.4903	23.834	24.2847	23.1756	NaN	NaN	NaN	NaN	NaN	NaN	NaN	Q9P2C4	Q9P2C4	Transmembrane p	TMEM181
NaN	NaN	NaN	NaN	NaN	NaN	NaN	NaN	NaN	NaN	NaN	21.1975	Q96HH6;F8VRE8;I	Q96HH6;F8VRE8;I	Transmembrane p	TMEM19
25.8059	25.4609	26.3808	26.3713	25.7131	26.6256	25.9188	NaN	25.7483	26.3297	25.9335	26.1481	Q9UHN6;Q9UHN6	Q9UHN6;Q9UHN6	Transmembrane p	TMEM2
24.5976	24.1106	24.8201	24.8029	24.6164	24.6407	24.7597	NaN	NaN	24.4312	NaN	NaN	Q6UW68;K7EM09	Q6UW68;K7EM09	Transmembrane p	TMEM205
23.6757	23.3471	23.9869	24.3943	NaN	23.8073	NaN	NaN	NaN	23.6731	NaN	NaN	Q9H813;Q9H813-	Q9H813;Q9H813-	Transmembrane p	TMEM206
23.0813	NaN	23.3741	22.8131	NaN	NaN	NaN	NaN	NaN	22.4948	NaN	NaN	Q9H0R3;H0Y870;I	Q9H0R3;H0Y870;I	Transmembrane p	TMEM222
24.1399	NaN	NaN	NaN	NaN	NaN	NaN	NaN	NaN	23.4519	NaN	NaN	Q96A57;Q96A57-	Q96A57;Q96A57-	Transmembrane p	TMEM230
24.6139	25.6258	25.3219	24.6794	25.149	25.3288	25.2985	NaN	25.6877	25.7575	25.6803	25.2742	Q96Q45;Q96Q45-	Q96Q45;Q96Q45-	Transmembrane p	TMEM237
28.6478	28.5125	28.6274	29.0332	NaN	NaN	28.4854	NaN	NaN	29.4693	NaN	NaN	Q9H330;Q9H330-	Q9H330;Q9H330-	Transmembrane p	TMEM245
25.8713	25.8197	25.9782	25.2972	25.1309	25.0861	24.8255	24.3846	NaN	24.6519	24.2394	24.5547	Q8WUH6	Q8WUH6	Transmembrane p	TMEM263
26.9214	27.0052	27.5508	27.3297	27.3349	27.7373	27.0566	26.9698	27.3194	27.4457	27.2194	27.4088	Q9NV96;Q9NV96-	Q9NV96;Q9NV96-	Cell cycle control	TMEM30A
26.6098	25.4884	26.0581	26.4546	25.2716	25.9699	26.0561	25.4252	26.1174	26.5879	25.8565	25.4707	P57088;D6RAA6;H	P57088;D6RAA6;H	Transmembrane p	TMEM33
24.3458	24.3429	25.0543	24.3613	24.1039	24.7872	23.6482	NaN	NaN	23.5724	23.8217	NaN	Q5BJD5;E9PJ42;Q	Q5BJD5;E9PJ42;Q	Transmembrane p	TMEM41B
25.6788	26.0841	26.3709	26.3007	26.3291	26.5394	27.5816	27.608	28.5733	27.718	27.4376	28.0022	Q9BTV4;V9GY05	Q9BTV4	Transmembrane p	TMEM43
NaN	NaN	22.4782	NaN	NaN	22.0786	NaN	NaN	NaN	NaN	22.3402	22.9972	Q9NW97	Q9NW97	Transmembrane p	TMEM51
24.9317	24.8703	24.9941	24.7119	24.6957	24.2352	24.6565	NaN	26.299	24.3488	23.8725	24.9712	Q9BXS4;Q5T6Z8;C	Q9BXS4;Q5T6Z8;C	Transmembrane p	TMEM59
22.3328	22.3899	23.1088	NaN	22.7538	NaN	NaN	NaN	NaN	NaN	NaN	NaN	O94886;Q2HIZ8;X	O94886	CSC1-like protein	TMEM63A
23.8306	23.7154	24.0738	24.0191	24.121	24.6451	NaN	NaN	NaN	NaN	NaN	NaN	Q5T3F8;H0YCP6;H	Q5T3F8;H0YCP6;H	CSC1-like protein	TMEM63B
25.4901	25.0811	25.5775	25.7608	25.2216	25.7185	25.737	NaN	24.9159	24.7806	24.7596	24.3419	Q8NBN3;Q8NBN3	Q8NBN3;Q8NBN3	Transmembrane p	TMEM87A
NaN	NaN	22.0354	NaN	NaN	NaN	NaN	NaN	NaN	NaN	NaN	NaN	Q9HCN3;K4DI83	Q9HCN3	Transmembrane p	TMEM8A
NaN	23.4891	24.361	23.6241	23.7624	23.919	NaN	NaN	NaN	NaN	NaN	NaN	Q9P0T7;B1ALM5	Q9P0T7;B1ALM5	Transmembrane p	TMEM9
24.1338	NaN	23.719	24.6269	22.5693	23.5443	NaN	NaN	NaN	25.0191	24.0189	NaN	Q5BJF2;J3KT68;Q	Q5BJF2;J3KT68	Transmembrane p	TMEM97
22.8894	NaN	23.9021	23.5023	23.269	23.8848	NaN	NaN	NaN	NaN	NaN	NaN	Q9NQ34;Q9NQ34	Q9NQ34;Q9NQ34	Transmembrane p	TMEM9B

23.0489	NaN	23.1916	22.6813	NaN	NaN	NaN	NaN	NaN	NaN	NaN	NaN	P82094;A0A0A0M	P82094;A0A0A0M	TATA element mo	TMF1
27.4661	27.9018	27.4634	27.3506	27.9247	27.5676	26.5429	27.5368	27.5029	26.2025	27.0208	27.223	Q9NYL9;H0YKU1;I	Q9NYL9;H0YKU1;I	Tropomodulin-3	TMOD3
28.023	27.5186	27.144	27.6258	27.1	27.2471	27.0225	27.4301	26.0957	27.6324	27.7447	27.3568	P42166;P42167-3	P42166	Lamina-associatec	TMPO
25.997	25.1011	25.6354	25.9754	25.4842	25.7911	24.7208	NaN	25.01	25.3611	24.0802	24.869	P42167;G5E972;P	P42167;G5E972;P	Lamina-associatec	TMPO
NaN	NaN	NaN	20.7216	NaN	NaN	NaN	NaN	NaN	NaN	NaN	NaN	Q6ZT21;Q6ZT21-2	Q6ZT21;Q6ZT21-2	Transmembrane p	TMPPE
29.7122	29.5379	29.3512	29.0092	28.7381	29.2112	29.8502	28.997	28.192	29.6993	29.5174	29.1937	P63313	P63313	Thymosin beta-10	TMSB10
30.6189	30.2417	30.3168	29.8517	29.4644	30.3012	31.6287	29.6966	29.2127	31.6262	30.4889	29.8368	P62328	P62328	Thymosin beta-4;I	TMSB4X
NaN	NaN	21.7939	NaN	NaN	NaN	NaN	NaN	NaN	NaN	NaN	NaN	Q9BVT8;C9JE12;C	Q9BVT8;C9JE12;C	Transmembrane a	TMUB1
27.3927	26.5195	26.7725	27.6204	26.4138	26.7496	27.7254	27.0739	27.1135	27.5927	27.029	26.6885	Q9H3N1;G3V448	Q9H3N1	Thioredoxin-relate	TMX1
23.5842	22.9305	23.4927	23.1463	23.27	NaN	24.293	24.9793	23.4925	25.0129	24.0709	23.5156	Q9Y320;Q9Y320-2	Q9Y320;Q9Y320-2	Thioredoxin-relate	TMX2
NaN	23.0394	23.0029	22.7505	23.0793	22.9539	23.2111	NaN	NaN	22.3473	NaN	23.277	Q96JJ7;Q96JJ7-2;I	Q96JJ7;Q96JJ7-2	Protein disulfide-i	TMX3
24.1223	24.0526	23.3951	NaN	NaN	NaN	30.7174	30.6189	29.1581	24.9469	26.3318	27.0392	P24821;E9PC84;P	P24821;E9PC84;P	Tenascin	TNC
24.9697	25.0005	25.6397	24.8743	25.1695	25.4048	NaN	NaN	25.0346	25.5429	24.8213	24.5075	Q03169;H0YLC0;H	Q03169	Tumor necrosis fa	TNFAIP2
22.8201	NaN	22.133	NaN	NaN	NaN	NaN	NaN	NaN	NaN	NaN	NaN	P21580;A0A087W	P21580;A0A087W	Tumor necrosis fa	TNFAIP3
22.1435	NaN	NaN	NaN	NaN	NaN	NaN	NaN	NaN	NaN	NaN	NaN	O95379-3;O95379	O95379-3;O95379	Tumor necrosis fa	TNFAIP8
25.8642	25.3167	26.0672	25.6325	25.2443	25.9235	24.868	NaN	24.7864	25.5172	25.3096	25.0539	O00220;F8U8C0	O00220;F8U8C0	Tumor necrosis fa	TNFRSF10A
26.2898	26.5714	26.8002	26.0116	26.073	26.856	26.1625	NaN	25.5008	26.4976	26.2973	26.6494	O14763;O14763-2	O14763;O14763-2	Tumor necrosis fa	TNFRSF10B
25.8429	25.2235	24.8147	24.8533	24.5557	NaN	NaN	NaN	NaN	NaN	NaN	NaN	Q9UBN6	Q9UBN6	Tumor necrosis fa	TNFRSF10D
NaN	NaN	NaN	NaN	NaN	NaN	21.1311	NaN	NaN	NaN	NaN	NaN	P19438;F5H061;P	P19438;F5H061;P	Tumor necrosis fa	TNFRSF1A
29.6734	29.2578	29.9636	28.387	27.8102	28.6977	28.9293	28.8912	28.2611	27.5339	26.9007	27.4884	Q15025;Q15025-2	Q15025;Q15025-2	TNFAIP3-interacti	TNIP1
24.6462	25.6396	25.437	24.2952	25.208	25.4197	25.51	25.0134	25.3794	24.9516	25.1656	25.8234	Q9C0C2;Q9C0C2-	Q9C0C2;Q9C0C2-	182 kDa tankyrase	TNKS1BP1
28.8555	27.8713	28.4793	28.972	28.1549	28.5488	28.1261	28.1674	28.5285	28.4757	28.6097	28.1439	Q92973;Q92973-2	Q92973;Q92973-2	Transportin-1	TNPO1
24.7523	23.1168	23.6383	25.2226	23.4317	23.8678	NaN	NaN	NaN	24.3317	NaN	22.8422	O14787-2;O14787	O14787-2;O14787	Transportin-2	TNPO2
23.655	23.535	23.8534	23.8985	23.8091	23.5362	23.8912	NaN	23.7558	24.2352	24.1263	24.1111	Q9Y5L0;C9J7E5;Q	Q9Y5L0;C9J7E5;Q	Transportin-3	TNPO3
25.5068	25.5388	25.9525	25.0988	25.3062	25.6006	25.9919	25.7347	25.9063	25.456	25.2479	25.5955	Q68CZ2;Q68CZ2-2	Q68CZ2;Q68CZ2-2	Tensin-3	TNS3
25.0684	23.6534	24.0172	24.938	23.938	24.103	NaN	23.4609	NaN	24.025	24.7629	NaN	Q96GM8;Q96GM	Q96GM8;Q96GM	Target of EGR1 pr	TOE1
26.9785	26.8774	27.194	26.4571	26.3106	26.6337	26.9574	NaN	26.6421	26.5287	25.8485	26.0388	Q9H0E2;F2Z2Y8;E	Q9H0E2;F2Z2Y8;E	Toll-interacting pr	TOLLIP
24.2215	24.3433	24.244	22.9286	23.5885	23.0935	23.9867	NaN	23.5132	21.8902	NaN	NaN	O60784;O60784-4	O60784;O60784-4	Target of Myb pro	TOM1
24.5827	25.0433	24.8921	23.8973	24.5249	24.5512	24.8577	NaN	NaN	NaN	NaN	NaN	O75674;J3KQU4;I	O75674;J3KQU4;I	TOM1-like proteir	TOM1L1
23.7984	24.0929	24.0383	24.1232	24.0086	23.4611	24.2936	23.9863	NaN	23.6616	NaN	NaN	Q6ZVM7;Q6ZVM7	Q6ZVM7;Q6ZVM7	TOM1-like proteir	TOM1L2
22.6095	22.1969	22.837	23.3088	21.5893	22.1523	22.0008	NaN	NaN	NaN	NaN	NaN	Q9NS69	Q9NS69	Mitochondrial imp	TOMM22
29.196	29.3225	28.854	28.9977	29.1134	28.6892	28.8171	28.7492	28.8652	28.6801	29.0642	29.1225	Q15785	Q15785	Mitochondrial imp	TOMM34
24.0896	24.6514	24.5006	24.6711	24.6451	24.4326	NaN	25.0215	24.9698	24.6769	23.9629	24.4107	O96008;O96008-2	O96008;O96008-2	Mitochondrial imp	TOMM40
23.9543	24.3587	23.8481	24.2045	24.5517	24.2773	23.7809	NaN	NaN	NaN	NaN	NaN	Q8N4H5;F8W8Z9;	Q8N4H5;F8W8Z9;	Mitochondrial imp	TOMM5
24.5761	26.2477	25.6655	25.2786	26.4569	25.6382	25.0921	27.1615	NaN	24.5012	25.2098	NaN	Q96B49	Q96B49	Mitochondrial imp	TOMM6
26.3862	25.921	25.9205	26.3654	26.0412	25.9372	26.2061	26.2407	25.7337	26.7158	26.236	25.1188	O94826	O94826	Mitochondrial imp	TOMM70A
NaN	20.8207	22.5448	22.6176	NaN	NaN	NaN	NaN	NaN	NaN	22.1617	NaN	Q96HA7;Q96HA7-	Q96HA7;Q96HA7-	Tonsoku-like prot	TONSL
28.5172	27.0269	27.0457	28.7964	27.6139	26.9223	28.7257	25.539	26.3767	28.0597	27.4037	27.2178	P11387;Q969P6;E	P11387	DNA topoisomera	TOP1
29.6668	28.6087	29.6427	30.4776	29.8737	29.8818	28.2153	27.0165	27.1982	29.7702	29.6945	28.6889	P11388;P11388-2,	P11388;P11388-2,	DNA topoisomera	TOP2A
25.4039	22.9498	24.5164	25.8854	24.0537	24.5871	NaN	NaN	NaN	24.3393	24.3187	NaN	Q02880;Q02880-2	Q02880;Q02880-2	DNA topoisomera	TOP2B
22.4457	NaN	21.9196	22.9822	23.0457	NaN	NaN	NaN	NaN	22.312	22.2199	21.8402	Q13472;Q13472-3	Q13472;Q13472-3	DNA topoisomera	TOP3A
NaN	NaN	NaN	22.4582	NaN	NaN	NaN	NaN	NaN	NaN	NaN	NaN	O95985;O95985-3	O95985;O95985-3	DNA topoisomera	TOP3B
23.6613	22.9546	23.6091	23.2163	23.5291	23.4413	NaN	NaN	NaN	23.8541	23.4801	23.2785	O14656;O14656-2	O14656	Torsin-1A	TOR1A
25.1692	25.2843	25.035	25.2127	25.5505	25.2535	24.2063	25.5877	25.0574	24.7346	24.9352	24.4458	Q5JTV8;A0A0A0M	Q5JTV8;A0A0A0M	Torsin-1A-interact	TOR1AIP1
NaN	24.2059	NaN	23.0712	NaN	NaN	23.6553	24.2356	NaN	23.1539	23.1186	23.6347	Q8NFQ8	Q8NFQ8	Torsin-1A-interact	TOR1AIP2
23.6182	22.0747	22.8179	22.9516	22.3077	22.4692	24.306	NaN	NaN	24.3193	NaN	NaN	O14657;H0Y7C8	O14657	Torsin-1B	TOR1B
NaN	NaN	NaN	NaN	20.2425	NaN	NaN	NaN	NaN	NaN	NaN	NaN	O94842;O94842-3	O94842;O94842-3	TOX high mobility	TOX4
27.4675	27.796	27.6047	28.2313	28.0415	27.8818	26.0737	25.8295	26.5424	27.434	27.901	27.2275	P04637;P04637-4	P04637;P04637-4,	Cellular tumor ant	TP53
23.1793	NaN	21.785	22.2151	NaN	22.0359	NaN	NaN	NaN	NaN	NaN	NaN	Q12888;A6NNK5;	Q12888;A6NNK5;	Tumor suppressor	TP53BP1
NaN	NaN	23.4624	21.3371	NaN	NaN	NaN	NaN	NaN	22.8187	NaN	NaN	Q13625;Q13625-2	Q13625;Q13625-2	Apoptosis-stimula	TP53BP2
22.9213	NaN	23.104	NaN	23.9404	NaN	NaN	NaN	NaN	23.775	NaN	NaN	Q53FA7;H7BZH6;(	Q53FA7;H7BZH6;(	Quinone oxidored	TP53I3
25.9515	25.8372	25.8408	26.064	25.9433	25.7925	24.696	24.2579	25.0006	26.557	26.9616	26.0636	Q96S44;Q5JZ02	Q96S44;Q5JZ02	TP53-regulating ki	TP53RK
27.5521	27.534	28.3495	27.8833	27.8478	28.4989	27.8262	27.8948	28.3991	28.2449	28.0076	28.87	Q13641	Q13641	Trophoblast glyco	TPBG
23.7493	23.9006	24.6173	24.6585	24.4024	24.7744	23.7512	23.6678	24.3645	NaN	23.2963	NaN	Q9ULQ1;Q9ULQ1	Q9ULQ1;Q9ULQ1	Two pore calcium	TPCN1
26.1441	27.404	26.6771	25.7397	27.112	26.4777	26.2095	26.6544	26.5828	25.3737	25.674	26.8288	P55327-2;P55327	P55327-2;P55327	Tumor protein D5	TPD52
28.6332	29.6955	29.0588	28.4539	29.5947	28.9623	29.2991	29.7682	29.5116	28.3009	28.9128	29.3806	O43399;O43399-5	O43399;O43399-5	Tumor protein D5	TPD52L2
23.8075	24.2606	23.7417	23.5948	24.097	NaN	23.8292	NaN	NaN	NaN	23.7775	NaN	O43399-2;O43399	O43399-2;O43399	Tumor protein D5	TPD52L2
32.4741	33.3696	32.7913	32.068	32.8114	32.726	32.7433	32.8754	32.8881	32.8102	32.872	33.1411	P60174;P60174-1	P60174;P60174-1	Triosephosphate i	TPI1
27.537	28.8063	28.4359	27.9005	28.9074	28.799	28.3648	28.7028	28.5037	28.0957	28.6015	28.851	H7BYY1;F5H7S3;B	H7BYY1;F5H7S3;B	Tropomyosin alph	TPM1
31.5309	32.397	32.1386	31.7505	32.4047	32.4081	32.2057	32.1786	31.8858	32.2042	32.2455	32.5446	P06753-2;P06753	P06753-2;P06753	Tropomyosin alph	TPM3;DKFZp686J1

29.9653	30.6149	30.5637	30.1695	30.4204	30.5386	30.552	30.7038	29.9513	30.7963	30.8395	30.5916	P67936;K7EP68;K	P67936	Tropomyosin alph	TPM4
24.6836	NaN	25.1525	NaN	NaN	24.9871	25.9036	25.1007	NaN	NaN	NaN	NaN	P67936-2;K7ENT6	P67936-2;K7ENT6	Tropomyosin alph	TPM4
26.0351	26.9333	26.3756	25.5926	25.8656	26.0594	27.3481	26.8621	26.218	25.4256	24.9981	25.5907	O14773;A0A0C4D	O14773;A0A0C4D	Tripeptidyl-peptid	TPP1
28.0637	28.6368	28.0047	28.02	28.4417	27.855	28.4715	28.9801	28.4235	28.3413	28.2054	28.1248	P29144;Q5VZU9	P29144;Q5VZU9	Tripeptidyl-peptid	TPP2
27.8134	27.437	27.9456	28.3223	28.2341	28.1558	25.7136	26.6326	25.7346	28.1023	28.206	27.8491	P12270;P12270-2,	P12270	Nucleoprotein TPI	TPR
24.3868	NaN	23.012	23.0725	NaN	NaN	24.8557	NaN	NaN	23.8843	23.0756	NaN	Q5T0D9;Q5T0D9-	Q5T0D9;Q5T0D9-	Tumor protein p6	TPRG1L
23.208	22.9527	22.7131	22.8152	22.9739	23.0519	22.9783	NaN	NaN	23.0211	23.0866	23.5232	Q9Y3C4;Q9Y3C4-3	Q9Y3C4;Q9Y3C4-3	EKC/KEOPS compl	TPRKB
22.0351	22.4696	22.5454	22.193	22.5061	23.5082	NaN	NaN	NaN	NaN	NaN	NaN	Q4KMQ1;H3BLU1	Q4KMQ1	Taperin	TPRN
NaN	NaN	22.2195	NaN	NaN	NaN	22.9321	NaN	NaN	NaN	NaN	NaN	O60507;C9J3I4	O60507	Protein-tyrosine s	TPST1
29.1017	30.0528	29.609	28.9212	29.82	29.5972	30.3939	30.3117	30.023	29.6331	29.631	30.1454	P13693;Q5W0H4;	P13693;Q5W0H4;	Translationally-co	TPT1
NaN	NaN	NaN	NaN	22.9898	NaN	NaN	NaN	NaN	NaN	NaN	NaN	Q9ULW0;Q9ULW(	Q9ULW0;Q9ULW(	Targeting protein	TPX2
27.9771	27.3994	28.1248	28.5112	28.0167	28.282	27.092	28.2483	26.8489	28.7254	28.9391	28.1835	Q13595;Q13595-3	Q13595;Q13595-3	Transformer-2 prc	TRA2A
28.2234	27.4427	28.0363	28.4717	27.4084	28.3092	27.0416	27.4129	26.6446	28.7189	28.5483	27.7088	P62995;P62995-3,	P62995;P62995-3,	Transformer-2 prc	TRA2B
NaN	NaN	NaN	21.8752	NaN	NaN	NaN	NaN	NaN	NaN	NaN	NaN	Q15628;Q15628-2	Q15628;Q15628-2	Tumor necrosis fa	TRADD
22.4071	NaN	NaN	22.6102	NaN	NaN	NaN	NaN	NaN	NaN	NaN	NaN	Q12933;Q12933-4	Q12933;Q12933-4	TNF receptor-assc	TRAF2
22.1276	21.5866	21.6747	22.1103	21.7366	NaN	NaN	NaN	NaN	21.578	21.5086	NaN	Q9Y4K3	Q9Y4K3	TNF receptor-assc	TRAF6
25.0822	NaN	24.6205	24.4348	24.4782	24.4339	NaN	NaN	NaN	NaN	NaN	NaN	O14545;F8VNX8	O14545;F8VNX8	TRAF-type zinc fin	TRAFD1
22.5121	NaN	22.1978	22.3225	NaN	NaN	NaN	NaN	NaN	22.3114	21.9875	NaN	Q15629;G3XAN4;	Q15629;G3XAN4;	Translocating chai	TRAM1
26.4785	27.2317	26.4329	26.5229	26.9915	26.3532	25.9334	26.5044	27.4105	26.3602	26.5952	26.6424	Q12931;Q12931-2	Q12931;Q12931-2	Heat shock protei	TRAP1
23.4238	23.3404	23.5995	23.6227	23.6941	23.6431	22.9115	NaN	22.9724	22.8344	23.5649	23.4925	Q9Y5R8;I3L3S0;I3	Q9Y5R8;I3L3S0	Trafficking proteir	TRAPPC1
21.3342	NaN	21.4595	22.3352	NaN	21.8383	NaN	NaN	NaN	21.0068	NaN	NaN	P48553	P48553	Trafficking proteir	TRAPPC10
24.4368	23.2943	24.2896	24.5476	23.792	24.4742	24.1847	NaN	NaN	24.1182	NaN	NaN	Q7Z392;Q7Z392-4	Q7Z392;Q7Z392-4	Trafficking proteir	TRAPPC11
24.319	NaN	NaN	24.0487	NaN	24.2095	NaN	NaN	NaN	NaN	NaN	NaN	Q8WVT3;A0A0J9Y	Q8WVT3;A0A0J9Y	Trafficking proteir	TRAPPC12;CGI-87
NaN	23.9876	23.7853	23.5775	24.1029	NaN	NaN	NaN	NaN	NaN	23.9621	NaN	A5PLN9;A5PLN9-2	A5PLN9;A5PLN9-2	Trafficking proteir	TRAPPC13
NaN	NaN	21.397	21.6466	NaN	NaN	NaN	NaN	NaN	NaN	NaN	NaN	P0DI81;P0DI82;PC	P0DI81;P0DI82;PC	Trafficking proteir	TRAPPC2;TRAPPC2
NaN	NaN	23.005	23.421	NaN	NaN	NaN	NaN	23.1896	23.1263	23.0239	23.168	Q9UL33;A0A0B4J	Q9UL33;A0A0B4J	Trafficking proteir	TRAPPC2L
25.4841	25.2889	24.8312	25.1761	25.2859	25.2528	24.4687	24.6	24.7608	24.4326	24.8417	25.0258	O43617;O43617-2	O43617;O43617-2	Trafficking proteir	TRAPPC3
23.6279	22.7806	23.2843	23.6087	22.629	23.048	NaN	NaN	NaN	22.5731	22.344	NaN	Q9Y296;G3V1A0;(	Q9Y296;G3V1A0;(	Trafficking proteir	TRAPPC4
23.7477	NaN	23.1484	23.8227	22.4623	23.2224	NaN	NaN	NaN	22.8238	NaN	NaN	Q8IUR0;M0QZQ8	Q8IUR0	Trafficking proteir	TRAPPC5
23.677	NaN	NaN	24.0425	NaN	NaN	NaN	NaN	NaN	NaN	NaN	NaN	Q86SZ2;Q86SZ2-2	Q86SZ2;Q86SZ2-2	Trafficking proteir	TRAPPC6B
23.5666	23.3393	23.9739	24.1702	23.8021	23.5128	23.0222	NaN	NaN	23.1239	NaN	23.2797	Q9Y2L5;J3QQJ5;Q	Q9Y2L5;J3QQJ5;Q	Trafficking proteir	TRAPPC8
NaN	NaN	NaN	23.03	22.6781	NaN	NaN	NaN	NaN	NaN	NaN	NaN	Q96Q05;H0YBR0;(	Q96Q05;H0YBR0;(	Trafficking proteir	TRAPPC9
NaN	NaN	NaN	NaN	NaN	NaN	NaN	NaN	NaN	NaN	NaN	24.9865	O14717;B7Z8H2;C	O14717;B7Z8H2;C	tRNA (cytosine(38	TRDMT1
21.6678	21.426	NaN	21.977	NaN	NaN	NaN	NaN	NaN	NaN	21.0814	NaN	Q14142;Q14142-3	Q14142;Q14142-3	Tripartite motif-cc	TRIM14
24.4443	23.7691	24.1768	24.2027	24.2136	NaN	NaN	NaN	NaN	23.6445	23.9506	NaN	O95361;B3KP96;H	O95361;B3KP96;H	Tripartite motif-cc	TRIM16;TRIM16L
22.6982	NaN	22.6312	NaN	NaN	23.2646	NaN	NaN	NaN	NaN	NaN	NaN	P19474;P19474-2	P19474;P19474-2	E3 ubiquitin-prote	TRIM21
NaN	NaN	NaN	NaN	NaN	NaN	24.4109	NaN	NaN	NaN	NaN	NaN	P36406;P36406-3,	P36406;P36406-3,	E3 ubiquitin-prote	TRIM23
22.1788	NaN	21.7919	22.3962	NaN	NaN	NaN	NaN	NaN	22.4213	22.2064	NaN	O15164;A0A087X	O15164;A0A087X	Transcription inte	TRIM24
29.3371	28.4425	28.9089	29.4387	28.6375	28.8592	28.0185	27.8945	27.8651	28.8821	28.5233	27.9942	Q14258	Q14258	E3 ubiquitin/ISG1!	TRIM25
23.9369	22.8827	23.2046	24.5171	23.2458	23.6443	22.8935	NaN	NaN	23.3384	22.9517	NaN	Q12899;A2AE48;A	Q12899	Tripartite motif-cc	TRIM26
28.3746	28.3614	28.5129	29.0488	29.0644	28.8416	27.514	27.4796	27.693	29.2596	29.3891	29.0073	Q13263;Q13263-2	Q13263;Q13263-2	Transcription inte	TRIM28
22.0108	NaN	NaN	21.7653	NaN	NaN	NaN	NaN	NaN	21.7796	NaN	NaN	Q13049;Q5JVY0	Q13049	E3 ubiquitin-prote	TRIM32
24.2405	23.2805	23.7542	24.6382	24.1684	24.1301	NaN	NaN	NaN	24.2517	24.4427	24.2828	Q9UPN9;Q9UPN9	Q9UPN9;Q9UPN9	E3 ubiquitin-prote	TRIM33
23.48	NaN	23.908	23.7032	NaN	NaN	NaN	25.1282	NaN	24.1046	24.2207	NaN	O00635	O00635	E3 ubiquitin-prote	TRIM38
24.9751	24.8826	24.9905	25.6882	25.7179	24.7073	23.1611	NaN	NaN	25.1045	25.2549	NaN	Q9BRZ2;C9JI91;Q	Q9BRZ2	E3 ubiquitin-prote	TRIM56
NaN	NaN	NaN	NaN	NaN	NaN	NaN	NaN	NaN	NaN	NaN	25.1058	Q5EBN2	Q5EBN2	Putative tripartite	TRIM61
26.3218	24.033	25.358	25.3121	23.9927	24.8614	24.7838	NaN	22.6339	25.2758	24.4878	23.9564	O75962;O75962-4	O75962;O75962-4	Triple functional d	TRIO
24.4269	25.2918	24.9521	24.4042	24.9954	25.377	24.6256	25.657	NaN	24.6422	24.7049	NaN	Q9H2D6;Q9H2D6	Q9H2D6;Q9H2D6	TRIO and F-actin-l	TRIOBP
25.3608	25.8391	25.674	25.0161	25.5442	25.5402	26.6591	26.321	25.829	25.8902	25.8456	26.139	Q15642;Q15642-2	Q15642;Q15642-2	Cdc42-interacting	TRIP10
22.2586	NaN	NaN	22.0885	NaN	NaN	NaN	NaN	NaN	NaN	NaN	NaN	Q15643;H0YJ97	Q15643;H0YJ97	Thyroid receptor-i	TRIP11
24.4925	23.624	23.9173	24.9142	23.5039	23.9646	23.4502	23.8411	NaN	23.9572	23.8378	22.6654	Q14669;Q14669-3	Q14669;Q14669-3	E3 ubiquitin-prote	TRIP12
26.8755	24.8128	25.8354	27.0092	24.7955	25.8532	24.1077	23.303	24.7708	25.6326	25.5103	24.6341	Q15645;H0YAL2;C	Q15645	Pachytene checkp	TRIP13
23.6742	23.0485	23.0246	NaN	23.467	NaN	NaN	NaN	NaN	22.1396	24.7072	NaN	Q15654;A0A0D9S	Q15654;A0A0D9S	Thyroid receptor-i	TRIP6
24.553	23.9395	23.8179	24.3158	24.3993	24.1547	NaN	24.6185	NaN	24.556	24.7073	24.7842	Q9NXH9;Q9NXH9	Q9NXH9;Q9NXH9	tRNA (guanine(26	TRMT1
28.1205	27.2536	26.9668	28.828	27.3219	27.1943	NaN	25.6255	NaN	26.4627	26.5228	26.9811	Q7L0Y3;C9JVB6	Q7L0Y3;C9JVB6	Mitochondrial ribc	TRMT10C
25.2173	25.1597	24.3938	25.0136	24.9135	24.2362	25.8504	NaN	NaN	25.8516	24.8497	NaN	Q7Z4G4;Q7Z4G4-	Q7Z4G4;Q7Z4G4-	tRNA (guanine(10	TRMT11
26.4282	25.9349	25.7905	25.7608	25.8435	25.7633	25.9186	25.1501	25.4934	25.9414	25.8828	26.1776	Q9UI30;F5GX77;C	Q9UI30;F5GX77;C	Multifunctional m	TRMT112
23.078	NaN	NaN	23.0371	NaN	NaN	NaN	NaN	NaN	NaN	NaN	NaN	Q7Z2T5;X6RK96;C	Q7Z2T5;X6RK96;C	TRMT1-like protei	TRMT1L
25.3005	24.7775	24.5463	25.0275	25.0139	24.776	NaN	NaN	24.15	25.5411	26.5773	24.7524	Q32P41;G3V5X1;(	Q32P41	tRNA (guanine(37	TRMT5
25.8476	24.7894	24.8009	25.738	25.1995	25.0334	23.6831	24.7014	24.5035	25.3148	25.8195	25.2683	Q9UJA5;Q9UJA5-4	Q9UJA5;Q9UJA5-4	tRNA (adenine(58	TRMT6

24.2927	23.8857	23.7124	24.9785	24.2509	NaN	NaN	NaN	23.2651	24.4201	24.9856	23.7882	Q96FX7;H0Y2Q1	Q96FX7;H0Y2Q1	tRNA (adenine(58	TRMT61A
24.3932	24.2585	23.7288	23.7601	23.9465	23.6321	NaN	NaN	24.5878	24.6367	24.9981	24.5804	Q96Q11;Q96Q11-	Q96Q11;Q96Q11-	CCA tRNA nucleot	TRNT1
26.9516	26.2066	26.6518	26.4214	26.3273	26.6956	26.4101	26.4605	25.9393	26.7007	26.6078	26.1081	P10155;P10155-5	P10155;P10155-5	60 kDa SS-A/Ro ril	TROVE2
23.6457	22.472	23.4516	23.8096	23.5921	23.4774	NaN	NaN	NaN	23.3581	23.0304	NaN	Q9Y4A5;F2Z2U4;C	Q9Y4A5;F2Z2U4;C	Transformation/tr	TRRAP
NaN	NaN	NaN	21.1856	NaN	NaN	NaN	NaN	NaN	NaN	NaN	NaN	Q8WWH5	Q8WWH5	Probable tRNA ps	TRUB1
NaN	NaN	NaN	20.812	NaN	NaN	NaN	NaN	NaN	NaN	NaN	NaN	P49815;P49815-3	P49815;P49815-3	Tuberin	TSC2
NaN	NaN	NaN	NaN	NaN	NaN	23.6634	NaN	NaN	NaN	NaN	NaN	Q15714;Q15714-2	Q15714;Q15714-2	TSC22 domain fan	TSC22D1
NaN	NaN	NaN	NaN	NaN	NaN	20.9004	NaN	NaN	NaN	NaN	NaN	Q9Y3Q8;H7BZ77;(	Q9Y3Q8;H7BZ77;(	TSC22 domain fan	TSC22D4
NaN	NaN	NaN	22.2002	NaN	NaN	NaN	NaN	NaN	NaN	NaN	NaN	Q8WW01;F2Z3M(	Q8WW01;F2Z3M(	tRNA-splicing end	TSEN15
24.2888	24.087	23.7652	24.557	23.8007	23.854	NaN	NaN	NaN	NaN	23.8169	NaN	P43897;P43897-2	P43897;P43897-2	Elongation factor	TSFM
27.107	26.6845	26.6444	26.3655	26.2416	26.2758	26.803	26.0947	27.0684	26.2145	25.7126	26.2261	Q99816;F5H442;C	Q99816;F5H442;C	Tumor susceptibil	TSG101
22.2277	22.15	22.3439	NaN	22.5534	22.651	24.1341	NaN	NaN	23.8752	NaN	NaN	Q8WUA8;E9PLG7	Q8WUA8	Tsukushin	TSKU
27.0916	27.0362	27.2934	27.0245	27.08	27.3261	27.3371	26.2887	26.8563	27.3301	27.3731	27.117	Q15631;E9PGT1;H	Q15631;E9PGT1;H	Translin	TSN
25.7883	26.5652	26.2411	25.5412	26.5043	25.9763	26.0576	26.9924	26.385	26.0714	26.4829	26.9247	Q99598;C4P0D6;(	Q99598;C4P0D6;(	Translin-associate	TSNAX;DISC1
25.449	24.832	25.3369	25.3113	24.3876	25.1598	24.5391	NaN	25.4657	25.5414	24.6525	25.2567	Q8NG11;H7BXY6;	Q8NG11;H7BXY6;	Tetraspanin-14;Te	TSPAN14
NaN	NaN	NaN	NaN	22.1474	NaN	NaN	NaN	NaN	NaN	NaN	NaN	O95858;H7C285	O95858;H7C285	Tetraspanin-15	TSPAN15
26.859	NaN	NaN	NaN	NaN	NaN	NaN	NaN	28.4809	NaN	NaN	27.6649	O60637;H0YKJ9;O	O60637;H0YKJ9;O	Tetraspanin-3	TSPAN3
NaN	26.5575	27.2125	26.127	26.3254	26.9242	27.142	26.5257	27.5572	26.2484	25.4957	26.8289	E9PSH3;E9PN41;E	E9PSH3;E9PN41;E	Tetraspanin;Tetra	TSPAN4
23.963	24.5787	25.1961	24.1925	24.0739	NaN	23.9279	NaN	24.9397	24.8619	24.3673	24.4647	B5MD23;O75954	B5MD23;O75954	Tetraspanin;Tetra	TSPAN9
26.6359	25.2089	26.0543	26.945	25.7993	26.0044	24.7882	25.8945	24.031	25.5572	25.5104	25.6792	Q2NL82;I3L1Q5;I3	Q2NL82;I3L1Q5	Pre-rRNA-process	TSR1
22.1845	NaN	NaN	NaN	NaN	NaN	NaN	NaN	NaN	NaN	NaN	NaN	Q9UJK0	Q9UJK0	Ribosome biogene	TSR3
25.4122	25.2425	25.0884	25.135	25.2712	25.0642	25.2889	25.6169	25.1955	25.4843	25.7143	25.3849	Q53HC9;A8MUM1	Q53HC9;A8MUM1	Protein TSSC1	TSSC1
25.8974	25.8176	26.0008	25.507	25.8386	25.6173	24.1229	24.966	NaN	25.7481	26.0718	25.5779	Q16762;B1AH48	Q16762	Thiosulfate sulfurt	TST
27.4496	27.1678	27.0533	27.0811	27.1499	27.1253	26.3226	25.2279	26.7579	27.2036	28.0044	26.9691	Q13630;E9PKL9;E	Q13630;E9PKL9;E	GDP-L-fucose synt	TSTA3
25.7639	26.0059	25.2627	24.977	25.6796	25.1771	NaN	26.9114	25.6617	26.1692	25.7574	NaN	Q99614;H0YB37	Q99614	Tetratricopeptide	TTC1
26.0286	24.993	25.386	25.9144	24.9228	25.3455	24.7284	25.3069	24.928	25.6623	25.5043	25.2504	Q6P3X3;F8WCH1;	Q6P3X3	Tetratricopeptide	TTC27
22.7129	NaN	NaN	NaN	NaN	NaN	NaN	NaN	NaN	NaN	NaN	NaN	Q6PGP7;D6RCE2	Q6PGP7;D6RCE2	Tetratricopeptide	TTC37
NaN	22.7779	22.1267	NaN	22.3539	NaN	22.3038	NaN	NaN	22.7241	22.892	NaN	Q5R3I4;A9UJP8;H	Q5R3I4	Tetratricopeptide	TTC38
23.8956	22.9054	23.1403	23.3419	23.5657	NaN	23.0188	NaN	NaN	23.2172	NaN	23.4666	O95801	O95801	Tetratricopeptide	TTC4
NaN	NaN	NaN	NaN	23.7644	NaN	NaN	NaN	NaN	NaN	NaN	NaN	Q9ULT0;G5E9G4;(	Q9ULT0;G5E9G4;(	Tetratricopeptide	TTC7A
25.1842	25.5928	26.3466	25.591	25.8179	26.1314	25.3425	24.512	24.9802	26.4911	25.8916	25.2924	Q86TV6;Q86TV6-2	Q86TV6;Q86TV6-2	Tetratricopeptide	TTC7B
25.1275	26.2093	25.5887	24.9745	25.9895	25.8594	26.2007	26.2859	25.9568	25.7218	26.264	26.1318	Q8N5M4;H0YDR3	Q8N5M4;H0YDR3	Tetratricopeptide	TTC9C
24.0004	23.9782	23.7158	23.5663	23.5605	23.697	23.0506	NaN	23.681	23.3941	23.856	NaN	Q9UNY4;Q9UNY4	Q9UNY4	Transcription tern	TTF2
25.4108	23.6695	24.2464	25.1304	23.9288	24.3468	23.7241	NaN	23.8256	24.2075	24.4072	NaN	O43156	O43156	TELO2-interacting	TTI1
25.346	NaN	23.9554	25.0014	23.4735	23.5721	NaN	NaN	NaN	24.4392	23.8294	NaN	Q6NXR4;E5RIH5	Q6NXR4;E5RIH5	TELO2-interacting	TTI2
23.564	23.4772	23.6764	23.8011	23.3926	23.9048	NaN	NaN	NaN	23.2161	NaN	NaN	P33981;P33981-2	P33981;P33981-2	Dual specificity pr	TTK
27.4193	25.96	26.3139	26.7174	26.0576	26.3161	26.5282	26.6337	26.7278	27.0584	27.0073	27.0197	Q14166;V9GY16	Q14166	Tubulin--tyrosine	TTLL12
NaN	NaN	NaN	25.0489	NaN	NaN	NaN	NaN	NaN	NaN	NaN	NaN	Q8WZ42;A0A0A0I	Q8WZ42;A0A0A0I	Titin	TTN
24.8259	25.0565	24.8978	24.1555	24.7268	24.9449	24.8957	NaN	25.999	24.912	NaN	25.3628	Q9C0H2;Q9C0H2-	Q9C0H2;Q9C0H2-	Protein tweety hc	TTYH3
27.1072	25.665	24.3292	27.5382	24.6397	NaN	NaN	NaN	NaN	NaN	NaN	NaN	Q71U36;Q71U36-	Q71U36;Q71U36-	Tubulin alpha-1A (	TUBA1A;TUBA3C
34.8198	33.9449	34.4603	34.7201	34.0661	34.4421	34.3414	34.3853	34.1993	34.8649	34.7605	33.6689	P68363;P68363-2	P68363;P68363-2	Tubulin alpha-1B (	TUBA1B
27.405	25.8811	23.902	27.1303	25.5005	23.7824	NaN	NaN	NaN	NaN	NaN	NaN	Q9BQE3;F5H5D3;	Q9BQE3;F5H5D3	Tubulin alpha-1C (	TUBA1C
27.5325	27.0041	27.1642	27.4726	27.1331	27.2369	27.0778	27.0196	26.644	27.5062	26.985	26.2067	P68366;P68366-2	P68366;P68366-2	Tubulin alpha-4A (	TUBA4A
32.8028	32.1311	32.7137	32.7638	32.4057	32.7414	32.0003	32.4912	32.0948	32.6515	32.7001	31.9371	P07437;Q5JP53;Q	P07437;Q5JP53;Q	Tubulin beta chair	TUBB
27.1878	27.5648	27.8792	28.111	27.7615	28.0368	27.7907	27.9456	28.1421	28.7715	28.3836	27.336	Q13885;Q9BVA1	Q13885;Q9BVA1	Tubulin beta-2A cl	TUBB2A;TUBB2B
NaN	NaN	NaN	19.6848	NaN	NaN	NaN	NaN	NaN	NaN	NaN	NaN	G3V542	G3V542		TUBB3
27.6404	27.7247	28.0276	28.0365	28.1828	28.072	28.8682	29.0887	29.2889	29.3977	29.6822	28.404	Q13509;A0A0B4J2	Q13509;A0A0B4J2	Tubulin beta-3 cha	TUBB3
34.3421	33.6267	34.1025	34.4083	33.8139	34.0955	33.8596	33.9913	33.8316	34.6348	34.5693	33.5693	P68371;M0R2D3;I	P68371	Tubulin beta-4B cl	TUBB4B
31.6475	30.5926	31.2517	31.8882	30.94	31.3406	30.2162	30.6299	30.6554	31.071	30.9175	30.1745	Q9BUF5;K7ESM5;	Q9BUF5;K7ESM5	Tubulin beta-6 cha	TUBB6
27.5777	26.2915	NaN	26.3664	24.3813	NaN	NaN	NaN	NaN	NaN	NaN	NaN	Q3ZCM7;A0A075E	Q3ZCM7;A0A075E	Tubulin beta-8 cha	TUBB8
25.6608	NaN	NaN	NaN	NaN	NaN	NaN	NaN	NaN	NaN	NaN	NaN	Q9UJT0;S4R3K3	Q9UJT0;S4R3K3	Tubulin epsilon ch	TUBE1
27.9533	27.1821	27.5489	27.8207	27.5447	27.7239	26.7502	27.7419	27.4145	27.4122	27.6722	27.3989	P23258;Q9NRH3;I	P23258;Q9NRH3	Tubulin gamma-1	TUBG1;TUBG2
26.3269	25.0039	25.5539	26.1516	24.9136	25.5165	25.2275	23.8618	25.2142	26.0028	25.6358	24.9544	Q9BSJ2;Q9BSJ2-4;	Q9BSJ2;Q9BSJ2-4;	Gamma-tubulin co	TUBGCP2
25.5157	24.2253	24.7607	25.5097	24.4043	24.8291	23.854	24.6613	24.256	25.1208	24.1755	23.1682	Q96CW5;Q96CW5	Q96CW5;Q96CW5	Gamma-tubulin co	TUBGCP3
23.6983	NaN	22.6517	23.125	22.5152	NaN	22.7377	NaN	NaN	22.7729	22.4526	NaN	Q9UGJ1;Q9UGJ1-	Q9UGJ1;Q9UGJ1-	Gamma-tubulin co	TUBGCP4
21.2199	NaN	NaN	NaN	NaN	NaN	NaN	NaN	NaN	NaN	NaN	NaN	Q96RT7;E7EQL8;C	Q96RT7;E7EQL8;C	Gamma-tubulin co	TUBGCP6
30.5503	29.9684	29.6878	30.5905	30.0718	30.0589	28.5262	29.1064	29.6176	29.5959	29.5588	29.2997	P49411;H3BNU3	P49411	Elongation factor	TUFM
NaN	NaN	22.4166	22.7208	NaN	NaN	NaN	NaN	25.0015	NaN	22.4803	NaN	Q13454;D6RDV0;I	Q13454;D6RDV0;I	Tumor suppressor	TUSC3
23.2568	NaN	22.7969	22.7563	23.058	22.8422	NaN	23.3748	NaN	NaN	NaN	23.4209	Q9NYZ1;C9JYS5;J3	Q9NYZ1;C9JYS5;J3	Golgi apparatus m	TVP23B;TVP23C;T\
25.8438	26.4486	26.0878	25.4747	26.1833	25.9834	26.5227	26.3105	27.2816	26.3373	26.0918	26.6862	Q12792;Q12792-4	Q12792;Q12792-4	Twinfilin-1	TWF1

26.1529	27.6347	27.0512	26.6751	27.2862	27.12	27.7843	27.6751	27.3724	27.3995	27.2431	27.7788	Q6IBS0;D6RG15;H	Q6IBS0;D6RG15;H	Twinfilin-2	TWF2
NaN	NaN	NaN	NaN	NaN	NaN	26.1328	25.9411	25.7337	NaN	NaN	NaN	Q9GZX9;J3QS03	Q9GZX9;J3QS03	Twisted gastrulati	TWSG1
27.6403	27.6012	27.6014	27.6441	27.3448	27.5173	27.1012	28.1213	27.2707	27.2391	27.5324	27.0416	P40222;Q8N3L3	P40222	Alpha-taxilin	TXLNA
25.7468	25.9625	25.7252	25.9296	25.9036	25.3327	25.9247	26.2603	NaN	25.508	25.714	25.7081	Q9NUQ3;Q9NUQ3	Q9NUQ3;Q9NUQ3	Gamma-taxilin	TXLNG
29.0865	28.4397	28.5159	29.0886	28.2344	28.4371	28.2222	26.9509	26.8827	27.9425	27.5848	27.2591	P10599;P10599-2	P10599;P10599-2	Thioredoxin	TXN
24.696	26.2657	25.7442	25.3703	26.2846	26.0191	26.0263	26.7788	26.5979	25.5378	25.8871	26.3233	O95881;V9GY50;\	O95881	Thioredoxin doma	TXNDC12
22.6237	22.7953	22.35	21.8945	22.019	NaN	23.7966	NaN	23.8299	21.7064	NaN	NaN	Q96J42;H0Y997;Q	Q96J42;H0Y997;Q	Thioredoxin doma	TXNDC15
25.4702	26.7934	26.3717	25.5909	26.49	26.262	26.5308	26.5644	27.0203	25.7819	26.5416	27.0298	Q9BRA2;I3L0K2;I3	Q9BRA2;I3L0K2;I3	Thioredoxin doma	TXNDC17
27.8297	28.9179	28.4045	28.1518	28.7465	28.3482	28.211	29.8992	29.4928	28.5982	28.7229	28.5799	Q8NBS9;Q8NBS9-	Q8NBS9;Q8NBS9-	Thioredoxin doma	TXNDC5
24.4322	24.0067	24.178	23.7556	23.5924	23.948	23.7263	24.0422	NaN	24.4252	23.9822	23.9752	O14530;O14530-2	O14530;O14530-2	Thioredoxin doma	TXNDC9
25.8122	24.8122	25.6269	24.9481	23.9635	24.5908	24.2544	23.0678	NaN	24.3771	NaN	NaN	Q9H3M7;Q9H3M	Q9H3M7;Q9H3M	Thioredoxin-intera	TXNIP
25.538	25.9496	25.6695	25.5058	25.7559	25.6706	25.1842	25.5633	25.6954	25.853	25.9651	25.9491	O43396;K7ER96;K	O43396;K7ER96	Thioredoxin-like p	TXNL1
27.955	28.9919	28.292	27.869	28.8784	28.2702	27.9988	27.8714	28.5658	28.1457	28.0635	28.8045	Q16881;Q16881-3	Q16881;Q16881-3	Thioredoxin reduc	TXNRD1
23.3237	23.7957	24.9583	23.3164	24.0321	25.145	23.0198	NaN	NaN	24.5486	24.5221	NaN	P29597;E9PM19;E	P29597;E9PM19;E	Non-receptor tyro	TYK2
27.624	26.9548	27.6945	27.2556	27.1258	27.3697	27.2482	27.6834	27.2316	27.9504	27.9087	27.7801	P04818;P04818-2	P04818;P04818-2	Thymidylate synth	TYMS
NaN	NaN	NaN	NaN	NaN	NaN	NaN	NaN	NaN	20.5357	NaN	NaN	Q2T9J0;Q2T9J0-2	Q2T9J0;Q2T9J0-2	Peroxisomal leade	TYSND1
27.8046	26.0257	26.2162	28.3423	26.1325	26.3409	NaN	NaN	24.0526	26.6425	26.0963	NaN	Q01081;P0DN76;(	Q01081;P0DN76;(	Splicing factor U2	U2AF1
25.8399	24.9127	24.4689	25.4539	25.6852	25.1575	25.6211	26.5483	NaN	24.7196	25.9705	26.7203	P26368;P26368-2	P26368;P26368-2	Splicing factor U2	U2AF2
26.0604	25.5165	25.9167	26.3969	25.8134	26.345	NaN	NaN	23.8001	25.3351	25.084	24.9894	O15042;O15042-2	O15042;O15042-2	U2 snRNP-associa	U2SURP
25.8721	25.5542	25.3811	25.7117	25.4013	25.4601	25.5964	25.7826	25.6307	25.4285	25.5718	25.7594	Q9BZF9;H0YNH8;(	Q9BZF9;H0YNH8;(	Uveal autoantiger	UACA
27.9378	28.0616	27.6371	27.7629	27.8582	27.7214	28.0167	27.7394	27.6115	28.1516	28.1818	28.2729	Q16222;Q16222-2	Q16222;Q16222-2	UDP-N-acetylhexc	UAP1
24.4558	23.6282	23.5109	23.8471	23.3831	NaN	24.0804	NaN	23.3477	25.5167	25.5121	NaN	Q3KQV9;Q3KQV9	Q3KQV9;Q3KQV9	UDP-N-acetylhexc	UAP1L1
29.8382	29.7168	29.5652	29.5138	29.7559	29.6532	29.7953	29.7834	30.0977	29.7794	29.9227	30.1075	P22314-2;P22314	P22314-2;P22314	Ubiquitin-like mo	UBA1
26.9787	26.1764	26.9915	27.2596	27.0045	27.0681	25.7977	25.5951	24.5191	27.1351	27.0469	26.6292	Q9UBT2;Q9UBT2-	Q9UBT2;Q9UBT2-	SUMO-activating	UBA2
24.5818	24.7521	24.4226	24.6494	24.9107	24.7946	25.1413	24.2615	24.8906	25.523	25.6421	25.9413	Q8TBC4;Q8TBC4-2	Q8TBC4;Q8TBC4-2	NEDD8-activating	UBA3
24.5101	24.358	24.4289	24.3421	24.5901	24.1479	24.6376	24.1334	24.6036	24.4834	24.7322	24.6976	Q9GZZ9;E7EWE1;I	Q9GZZ9;E7EWE1;I	Ubiquitin-like mo	UBA5
28.6623	27.5646	27.9742	28.5049	27.9163	28.0464	27.4992	27.5971	27.4731	28.1388	27.8777	27.5506	A0AVT1;A0AVT1-2	A0AVT1;A0AVT1-2	Ubiquitin-like mo	UBA6
21.9595	NaN	NaN	NaN	NaN	NaN	NaN	NaN	NaN	NaN	NaN	NaN	P41226	P41226	Ubiquitin-like mo	UBA7
20.8063	NaN	NaN	NaN	NaN	NaN	NaN	NaN	NaN	20.9075	21.0065	NaN	Q9BSL1	Q9BSL1	Ubiquitin-associat	UBAC1
22.5559	22.768	22.5702	22.0201	22.6849	22.5464	NaN	NaN	NaN	NaN	NaN	NaN	Q5T6F2;F5H2U4;(	Q5T6F2;F5H2U4;(	Ubiquitin-associat	UBAP2
26.2916	26.9898	26.2908	25.9591	26.4746	25.9682	25.6372	26.6655	25.4126	25.2681	25.8371	25.3081	Q14157;Q14157-5	Q14157;Q14157-5	Ubiquitin-associat	UBAP2L
26.481	25.3178	25.6399	25.7669	24.6538	25.5443	25.4632	25.0095	23.8139	24.2738	24.5024	23.6201	Q8TF42	Q8TF42	Ubiquitin-associat	UBASH3B
25.3495	25.5998	25.4515	24.6453	24.7918	24.7319	25.6435	25.8202	25.538	24.8154	24.7702	25.088	O00762;O00762-2	O00762;O00762-2	Ubiquitin-conjuga	UBE2C
NaN	NaN	NaN	NaN	NaN	NaN	NaN	NaN	NaN	NaN	NaN	22.5676	P62837	P62837	Ubiquitin-conjuga	UBE2D2
26.0649	26.028	26.2476	25.6756	26.0351	26.1638	26.1485	NaN	26.4892	26.449	26.5976	25.8388	P61077;P61077-3	P61077;P61077-3	Ubiquitin-conjuga	UBE2D3
22.069	22.5294	22.2538	NaN	NaN	NaN	22.2819	NaN	NaN	22.2162	NaN	NaN	P62253;I3L0Q0;I3	P62253;I3L0Q0;I3	Ubiquitin-conjuga	UBE2G1
24.6556	25.1657	24.802	24.4359	25.1234	24.6482	24.9009	NaN	24.3746	24.9773	24.7425	NaN	P60604;P60604-2	P60604;P60604-2	Ubiquitin-conjuga	UBE2G2
NaN	NaN	NaN	21.3049	NaN	NaN	NaN	NaN	NaN	NaN	NaN	NaN	P62256;H7C4M9;(	P62256;H7C4M9;(	Ubiquitin-conjuga	UBE2H
25.2502	26.5101	25.9108	25.6288	26.6565	26.1196	25.5585	NaN	26.5068	26.172	26.6301	26.6802	P63279;H3BQQ9;I	P63279;H3BQQ9;I	SUMO-conjugatin	UBE2I
25.9109	25.8751	25.9187	25.2656	25.6221	25.6369	25.7268	25.2942	25.6213	26.3414	25.9673	25.5582	P61086;D6RDM7;	P61086;D6RDM7;	Ubiquitin-conjuga	UBE2K
24.957	26.5581	26.0008	25.2156	26.1584	25.6678	26.4248	25.0819	26.0121	25.864	25.7102	25.9142	P68036;P68036-2	P68036;P68036-2	Ubiquitin-conjuga	UBE2L3
NaN	NaN	NaN	NaN	NaN	NaN	NaN	NaN	NaN	NaN	22.733	NaN	O14933;E9PQT7;(	O14933;E9PQT7;(	Ubiquitin/ISG15-c	UBE2L6
26.3531	27.1672	27.0565	26.6479	27.0346	27.2933	26.9135	25.2083	27.4193	27.3325	27.5261	27.7138	P61081;M0QX69;	P61081	NEDD8-conjugatir	UBE2M
28.4959	28.3063	28.5033	28.0479	28.1088	28.5884	28.6537	27.3075	28.0865	29.083	28.7221	28.7516	P61088;F8VQQ8;F	P61088;F8VQQ8;F	Ubiquitin-conjuga	UBE2N
25.9246	24.9046	25.5875	25.9126	25.1406	25.2689	25.0997	25.5617	25.7662	25.1455	25.3877	25.8208	Q9C0C9;K7ES11;K	Q9C0C9;K7ES11	E2/E3 hybrid ubiq	UBE2O
22.5422	22.1996	22.5837	22.9551	22.57	22.2739	22.2756	NaN	NaN	NaN	22.2198	22.3004	Q7Z7E8;Q7Z7E8-2	Q7Z7E8	Ubiquitin-conjuga	UBE2Q1
25.4252	25.4195	25.9732	25.8679	26.1104	26.0808	24.8495	25.1302	25.3866	26.0244	26.6232	26.6009	Q16763;K7EPJ1;K	Q16763;K7EPJ1	Ubiquitin-conjuga	UBE2S
23.3313	23.0472	23.3762	22.7889	NaN	23.2982	NaN	NaN	NaN	23.7686	23.8066	23.2623	Q9NPD8	Q9NPD8	Ubiquitin-conjuga	UBE2T
26.9767	26.6828	26.6328	26.7455	26.5237	26.6024	26.8606	27.0546	26.0583	27.1923	26.6749	27.221	Q13404;I3L0A0;Q	Q13404;I3L0A0;Q	Ubiquitin-conjuga	UBE2V1;TMEM18
27.6778	28.0417	27.8995	27.4439	27.6108	27.9679	27.7854	26.8982	27.2535	27.8401	28.039	27.8851	Q15819;G3V113;H	Q15819;G3V113;H	Ubiquitin-conjuga	UBE2V2
24.5219	25.5873	25.2456	24.7093	25.0907	25.2551	25.23	NaN	NaN	25.2575	25.8399	25.1916	Q9H832;Q9H832-	Q9H832;Q9H832-	Ubiquitin-conjuga	UBE2Z
24.5318	23.5277	24.0945	24.2855	23.4722	23.9544	23.6871	24.3762	NaN	24.6762	24.3305	NaN	Q05086;Q05086-2	Q05086;Q05086-2	Ubiquitin-protein	UBE3A
26.8742	25.6909	26.2349	26.7715	26.0809	25.9436	25.0842	25.7453	25.3591	26.1866	26.2134	24.1613	Q15386;Q15386-2	Q15386	Ubiquitin-protein	UBE3C
23.7056	22.7375	23.6484	24.2492	23.1494	22.695	NaN	NaN	23.4022	23.242	NaN	NaN	Q14139;Q14139-2	Q14139;Q14139-2	Ubiquitin conjuga	UBE4A
23.0948	NaN	22.171	22.6537	22.6328	22.5772	NaN	NaN	NaN	NaN	NaN	NaN	O95155;O95155-3	O95155;O95155-3	Ubiquitin conjuga	UBE4B
22.9965	NaN	NaN	23.2154	NaN	NaN	NaN	NaN	NaN	NaN	22.8298	NaN	Q9Y5Z9;Q9Y5Z9-2	Q9Y5Z9;Q9Y5Z9-2	UbiA prenyltransf	UBIAD1
22.8338	23.5722	23.4185	22.4896	23.2996	23.0048	NaN	NaN	NaN	NaN	22.9236	NaN	P11441;Q5HY81	P11441;Q5HY81	Ubiquitin-like prot	UBL4A
23.4917	23.2509	23.1428	23.6641	22.9614	23.0277	NaN	NaN	NaN	NaN	22.8263	NaN	Q8WVY7	Q8WVY7	Ubiquitin-like don	UBLCP1
21.8241	NaN	21.419	21.9408	NaN	NaN	NaN	NaN	21.9335	NaN	NaN	NaN	Q9NZI7;Q9NZI7-4	Q9NZI7;Q9NZI7-4	Upstream-binding	UBP1
27.1927	27.2539	27.2418	26.4686	26.8521	26.7839	26.833	27.1353	26.1035	26.799	26.8188	26.7637	Q9UMX0;Q9UMX	Q9UMX0	Ubiquilin-1	UBQLN1

NaN	NaN	NaN	NaN	NaN	NaN	21.9991	NaN	NaN	NaN	NaN	NaN	Q9UMX0-2	Q9UMX0-2	Ubiquilin-1	UBQLN1
23.3363	22.4738	NaN	23.4221	NaN	NaN	NaN	NaN	NaN	NaN	NaN	NaN	Q9UHD9	Q9UHD9	Ubiquilin-2	UBQLN2
21.4297	NaN	NaN	NaN	NaN	NaN	NaN	NaN	NaN	NaN	NaN	NaN	Q9NRR5;H0YDS0	Q9NRR5;H0YDS0	Ubiquilin-4	UBQLN4;UBQLN1
22.7369	NaN	22.1725	22.2582	21.7723	NaN	NaN	NaN	NaN	22.5313	NaN	NaN	Q8IWV7;A0A087V	Q8IWV7;A0A087V	E3 ubiquitin-prote	UBR1
23.2883	22.6058	22.8684	23.3683	NaN	22.9818	NaN	NaN	NaN	23.216	NaN	NaN	Q8IWV8;Q8IWV8-	Q8IWV8;Q8IWV8-	E3 ubiquitin-prote	UBR2
29.6054	28.6617	29.1759	29.6048	29.0357	29.2666	28.8942	29.1194	29.04	29.1862	28.9928	28.8728	Q5T4S7;Q5T4S7-3	Q5T4S7;Q5T4S7-3	E3 ubiquitin-prote	UBR4
23.3265	23.8468	23.7695	24.2136	23.6642	23.4301	NaN	NaN	NaN	23.6404	24.1058	23.4854	O95071;E7EMW7	O95071;E7EMW7	E3 ubiquitin-prote	UBR5
NaN	NaN	NaN	NaN	22.3825	NaN	NaN	NaN	NaN	NaN	NaN	NaN	Q8N806;E9PCJ7;H	Q8N806;E9PCJ7;H	Putative E3 ubiqu	UBR7
25.8797	26.0268	26.2207	25.6489	25.9232	26.395	25.7727	NaN	25.527	27.0167	26.405	25.9462	Q9HAC8	Q9HAC8	Ubiquitin domain-	UBTD1
23.6374	23.9309	24.3667	23.8362	NaN	24.3788	NaN	NaN	NaN	24.227	23.8275	NaN	Q8WUN7	Q8WUN7	Ubiquitin domain-	UBTD2
NaN	NaN	21.9686	22.3922	NaN	NaN	NaN	NaN	NaN	NaN	NaN	22.9077	P17480;P17480-2	P17480;P17480-2	Nucleolar transcri	UBTF
25.6229	26.3568	26.1137	25.275	26.9688	25.801	25.9313	26.1339	26.1815	24.9513	26.0766	26.6428	Q04323;E9PJ81;E	Q04323;E9PJ81;E	UBX domain-cont	UBXN1
22.6831	NaN	22.0474	22.4542	NaN	NaN	NaN	NaN	NaN	NaN	NaN	NaN	Q92575;F8WB86;	Q92575	UBX domain-cont	UBXN4
22.6489	23.0921	22.901	NaN	22.5817	NaN	NaN	NaN	NaN	22.9192	NaN	NaN	O94888;F8WB69;	O94888;F8WB69;	UBX domain-cont	UBXN7
28.1782	28.4471	28.197	27.7081	27.9703	28.0205	28.5152	27.7	27.7638	28.4513	27.9194	28.2245	P15374;A0A087W	P15374;A0A087W	Ubiquitin carboxy	UCHL3
25.0998	24.9473	24.6474	24.8068	24.2354	24.5005	24.6016	NaN	23.8345	24.5847	24.3272	24.6377	Q5LJA5;Q9Y5K5-2	Q5LJA5;Q9Y5K5-2	Ubiquitin carboxy	UCHL5
25.9409	25.3546	25.3357	25.9015	25.4793	25.2415	24.8339	25.3466	25.0882	25.375	25.7142	25.754	Q9BZX2;Q9BZX2-2	Q9BZX2	Uridine-cytidine k	UCK2
24.9962	NaN	23.5645	24.5301	23.1986	23.4681	NaN	NaN	NaN	23.6505	24.0966	NaN	Q9NWZ5;Q9NWZ	Q9NWZ5;Q9NWZ	Uridine-cytidine k	UCKL1
25.2965	24.5747	25.2123	24.5671	24.1611	25.0182	26.1095	NaN	24.2445	24.6795	NaN	NaN	Q8IX04;Q8IX04-6;	Q8IX04;Q8IX04-6;	Ubiquitin-conjuga	UEVLD
24.1571	24.3594	24.2906	23.6159	24.2489	24.3879	23.989	NaN	NaN	23.8785	23.8134	NaN	Q9Y3C8	Q9Y3C8	Ubiquitin-fold mo	UFC1
24.6956	24.8399	24.6747	24.1991	23.9245	23.9846	24.3093	25.1079	24.1248	23.5121	24.1089	24.2313	Q92890;Q92890-1	Q92890;Q92890-1	Ubiquitin fusion d	UFD1L
26.3051	25.7396	26.1215	26.3405	26.0187	26.0077	25.6958	25.4762	25.842	26.0258	25.9506	25.679	O94874;O94874-3	O94874;O94874-3	E3 UFM1-protein	UFL1
22.9824	24.9733	24.1376	23.4229	23.2078	24.0027	24.0704	NaN	NaN	23.3884	23.4957	NaN	P61960;H0Y614	P61960;H0Y614	Ubiquitin-fold mo	UFM1
23.735	23.2468	23.1483	NaN	NaN	NaN	NaN	NaN	NaN	NaN	NaN	NaN	Q9NUQ7;H0YA18	Q9NUQ7;H0YA18	Ufm1-specific pro	UFSP2
25.1295	25.038	25.0164	24.6623	24.314	24.2546	NaN	NaN	25.0777	24.4192	NaN	24.7782	Q16739	Q16739	Ceramide glucosy	UGCG
28.3475	27.9512	27.6761	28.009	28.1941	27.6387	27.1762	27.2504	27.6989	27.8608	28.0935	27.9055	O60701;O60701-2	O60701;O60701-2	UDP-glucose 6-de	UGDH
26.4358	26.7905	26.7414	26.4851	26.8559	26.7782	26.0888	26.963	27.2535	25.7435	25.9585	26.4998	Q9NYU2;Q9NYU2	Q9NYU2;Q9NYU2	UDP-glucose:glyc	UGGT1
28.2532	29.1264	28.7546	28.0624	28.929	28.6347	28.6536	28.9653	28.8175	28.6304	28.7487	28.9666	Q16851-2;C9JNZ1	Q16851-2;C9JNZ1	UTP--glucose-1-pl	UGP2
NaN	22.4062	22.8949	23.3637	22.6517	22.7397	22.3004	NaN	NaN	23.0457	23.1763	22.9035	Q96T88;Q96T88-2	Q96T88;Q96T88-2	E3 ubiquitin-prote	UHRF1
22.9011	NaN	NaN	22.7228	NaN	NaN	NaN	NaN	NaN	NaN	NaN	NaN	Q6BDS2;H7C1J4	Q6BDS2;H7C1J4	UHRF1-binding pr	UHRF1BP1
23.6074	24.2568	23.92	23.9226	24.3012	NaN	NaN	NaN	NaN	25.1301	24.77	NaN	Q96PU4;B1AL33;	Q96PU4;B1AL33;	E3 ubiquitin-prote	UHRF2
NaN	NaN	NaN	NaN	NaN	NaN	24.9213	NaN	NaN	22.9994	NaN	NaN	Q9BZM5;Q6H3X3	Q9BZM5	NKG2D ligand 2	ULBP2
23.993	23.866	24.5194	24.6454	24.3327	24.4728	25.0749	NaN	24.7317	25.7953	25.3889	25.2874	Q9BZM4;Q9BZM6	Q9BZM4	NKG2D ligand 3	ULBP3
28.5462	27.6114	27.8373	28.0899	27.7735	27.8419	27.3339	27.2747	27.3444	28.2604	28.4311	27.6144	P11172;P11172-3	P11172;P11172-3	Uridine 5-monoph	UMPS
NaN	NaN	22.3466	NaN	NaN	NaN	NaN	NaN	NaN	NaN	NaN	NaN	A6NIH7;K7EJU3;K	A6NIH7;K7EJU3;K	Protein unc-119 h	UNC119B;UNC119
27.7325	27.1451	27.5319	27.4038	26.7918	27.194	27.2332	27.2762	27.4563	27.1828	26.6744	26.7941	Q70J99;Q70J99-3	Q70J99;Q70J99-3	Protein unc-13 ho	UNC13D
28.0419	27.3733	27.5915	27.8471	27.5001	27.6668	27.5741	27.6158	27.3572	27.6483	27.5966	27.5428	Q9H3U1-2;Q9H3U	Q9H3U1-2;Q9H3U	Protein unc-45 ho	UNC45A
29.634	28.7382	29.1716	29.5698	28.9728	29.263	28.9563	29.3329	28.9265	29.5093	29.4898	29.1635	Q92900-2;Q9290	Q92900-2;Q9290	Regulator of nons	UPF1
24.0283	22.9013	23.383	23.8037	22.9742	23.3073	23.2428	NaN	NaN	23.2523	NaN	NaN	Q9HAU5;Q9HAU5	Q9HAU5	Regulator of nons	UPF2
24.4287	23.8161	23.6516	24.0723	24.5865	NaN	NaN	NaN	23.6381	24.2259	24.0748	NaN	Q16831;F8WD51;	Q16831;F8WD51;	Uridine phosphor	UPP1
24.2678	NaN	22.4283	22.6867	22.3456	NaN	NaN	NaN	NaN	NaN	NaN	NaN	Q96BW1;E9PSD7;	Q96BW1;E9PSD7;	Uracil phosphorib	UPRT
23.6685	23.6662	24.0547	24.1974	23.849	23.654	NaN	NaN	NaN	23.3574	23.4433	NaN	Q9BRT2;Q5TAQ0;	Q9BRT2;Q5TAQ0;	Ubiquinol-cytochr	UQCC2
23.8089	25.6605	25.0925	24.8472	25.8279	25.3444	23.8897	26.2428	26.816	23.3585	24.5978	25.4307	P14927;B7Z2R2;P	P14927;B7Z2R2;P	Cytochrome b-c1	UQCRB
25.9621	26.8241	26.466	26.4482	26.867	26.4734	25.6705	27.4062	28.5401	25.421	25.9502	26.9038	P31930	P31930	Cytochrome b-c1	UQCRC1
26.3048	27.5322	26.9115	27.1421	27.9977	27.165	25.9071	27.7657	28.6546	23.5342	26.5722	27.3724	P22695;H3BRG4;H	P22695;H3BRG4;H	Cytochrome b-c1	UQCRC2
24.6298	25.653	24.9055	24.8679	26.0751	25.0609	23.8313	25.8025	26.9204	NaN	25.0499	25.4097	P47985;P0C7P4	P47985;P0C7P4	Cytochrome b-c1	UQCRFS1;UQCRFS
24.5532	24.8121	24.6998	25.0088	24.6649	25.17	24.2376	NaN	NaN	NaN	NaN	NaN	O14949	O14949	Cytochrome b-c1	UQCRQ
21.1264	NaN	20.9433	21.7762	NaN	NaN	NaN	NaN	NaN	NaN	NaN	NaN	O60287	O60287	Nucleolar pre-ribo	URB1
22.4599	NaN	NaN	22.6728	NaN	NaN	NaN	NaN	NaN	21.2756	NaN	NaN	Q14146;Q5VYD0	Q14146	Unhealthy ribosor	URB2
23.0251	21.5091	21.8914	22.9404	21.6552	22.0886	NaN	NaN	NaN	21.7677	NaN	NaN	Q8TCY9;Q8TCY9-2	Q8TCY9;Q8TCY9-2	Up-regulator of ce	URGCP
25.6818	26.4398	26.0055	25.5539	26.378	26.0541	26.3136	26.2347	26.5073	26.251	26.2509	26.8654	P06132;H0Y5R6;C	P06132;H0Y5R6	Uroporphyrinoger	UROD
NaN	NaN	NaN	22.1599	NaN	NaN	NaN	NaN	NaN	NaN	NaN	NaN	Q15853;Q15853-3	Q15853;Q15853-3	Upstream stimula	USF2
24.225	24.7197	24.8765	24.7954	24.9396	24.7072	NaN	NaN	25.6636	23.3872	24.0489	NaN	Q96IX5	Q96IX5	Up-regulated duri	USMG5
27.5592	26.9808	27.2539	27.2179	26.9298	27.2412	27.1667	27.0628	26.8371	26.7351	26.7593	26.9155	O60763;O60763-2	O60763;O60763-2	General vesicular	USO1
26.2057	25.2777	25.6273	25.9725	25.5936	25.4902	25.5843	25.6286	25.9906	26.3962	26.8368	26.3149	Q14694;Q14694-3	Q14694;Q14694-3	Ubiquitin carboxy	USP10
23.1666	22.5715	23.1871	23.4082	22.5029	22.5201	NaN	NaN	NaN	22.0886	NaN	NaN	P51784;G5E9A6;C	P51784;G5E9A6	Ubiquitin carboxy	USP11
24.9518	23.8436	24.33	24.4929	23.9427	24.2375	23.8079	NaN	NaN	23.4961	24.2304	NaN	Q92995;Q92995-2	Q92995;Q92995-2	Ubiquitin carboxy	USP13
28.2216	27.9027	28.3716	27.9406	28.0488	28.4221	28.0396	28.3273	28.2562	28.2762	28.4619	28.4426	P54578;P54578-2	P54578;P54578-2	Ubiquitin carboxy	USP14
25.1696	24.7487	25.2779	25.3209	24.9659	25.0319	25.1517	25.7776	25.5212	24.729	25.0678	25.7823	Q9Y4E8;Q9Y4E8-2	Q9Y4E8;Q9Y4E8-2	Ubiquitin carboxy	USP15
23.7426	NaN	23.5381	23.7722	24.0731	23.5574	NaN	NaN	NaN	23.8855	NaN	NaN	Q9Y5T5;Q9Y5T5-3	Q9Y5T5;Q9Y5T5-3	Ubiquitin carboxy	USP16

24.6902	22.6292	23.6831	24.2543	23.5763	23.4964	NaN	NaN	NaN	24.0415	24.3959	NaN	O94966-7;O9496(	O94966-7;O9496(	Ubiquitin carboxy	USP19
23.0426	22.4737	23.2826	23.2246	22.9969	NaN	NaN	NaN	NaN	23.4571	23.3935	22.871	Q9UPT9;Q9UPT9-	Q9UPT9;Q9UPT9-	Ubiquitin carboxy	USP22
24.5085	23.5898	23.942	24.5662	24.0173	24.0439	NaN	24.7117	24.1784	24.3932	24.11	23.4481	Q9UPU5;A0A0U1I	Q9UPU5	Ubiquitin carboxy	USP24
22.5914	NaN	NaN	22.2054	NaN	NaN	NaN	NaN	NaN	NaN	NaN	NaN	Q9UHP3;Q9UHP3	Q9UHP3;Q9UHP3	Ubiquitin carboxy	USP25
21.1397	NaN	NaN	NaN	NaN	NaN	NaN	NaN	NaN	NaN	NaN	NaN	Q9Y6I4;H0YLB7;H	Q9Y6I4;H0YLB7;H	Ubiquitin carboxy	USP3
23.0892	22.6918	22.5719	NaN	NaN	NaN	NaN	NaN	NaN	NaN	NaN	NaN	Q8NFA0;K7EKZ1;K	Q8NFA0;K7EKZ1;K	Ubiquitin carboxy	USP32
22.6749	NaN	NaN	NaN	NaN	NaN	NaN	NaN	NaN	NaN	22.8551	NaN	Q8TEY7;Q8TEY7-3	Q8TEY7;Q8TEY7-3	Ubiquitin carboxy	USP33
22.2902	NaN	NaN	22.1239	NaN	NaN	NaN	NaN	NaN	NaN	NaN	NaN	Q9P275;E9PEW0;	Q9P275;E9PEW0;	Ubiquitin carboxy	USP36
25.1206	NaN	23.2704	24.8551	NaN	23.102	NaN	NaN	NaN	23.3708	23.3401	NaN	Q53GS9;B9A018;	Q53GS9;B9A018;	U4/U6.U5 tri-snRI	USP39
25.8094	NaN	24.834	25.928	24.5019	25.0242	NaN	NaN	NaN	25.3931	25.6265	25.3805	Q13107;Q13107-2	Q13107;Q13107-2	Ubiquitin carboxy	USP4
22.4875	NaN	22.0213	23.1622	NaN	NaN	NaN	NaN	NaN	22.1052	22.2495	NaN	Q9NVE5;Q9NVE5-	Q9NVE5;Q9NVE5-	Ubiquitin carboxy	USP40
NaN	NaN	NaN	22.3411	NaN	NaN	NaN	NaN	NaN	NaN	NaN	NaN	H7BZK6;P62068-2	H7BZK6;P62068-2	Ubiquitin carboxy	USP46;USP12
25.774	24.2966	24.8993	25.2276	24.688	24.7396	23.812	24.383	23.9522	24.5099	24.0293	23.396	Q96K76-4;Q96K7(	Q96K76-4;Q96K7(	Ubiquitin carboxy	USP47
NaN	NaN	NaN	21.4283	NaN	NaN	NaN	NaN	NaN	21.9922	NaN	NaN	Q86UV5;Q86UV5-	Q86UV5;Q86UV5-	Ubiquitin carboxy	USP48
28.7997	28.7129	28.7883	28.4861	28.646	28.5234	28.5989	28.5952	28.386	28.636	28.5871	28.5771	P45974;P45974-2	P45974;P45974-2	Ubiquitin carboxy	USP5
21.8096	22.4441	23.4505	23.0075	22.7074	24.01	NaN	22.4283	NaN	23.8614	23.1314	NaN	Q92738;X6RAB3;(	Q92738;X6RAB3;(	USP6 N-terminal-l	USP6NL
27.0351	25.7442	26.2937	27.1975	26.3083	26.4511	25.7066	25.7031	24.9353	27.0357	26.9379	26.0145	Q93009;Q93009-3	Q93009;Q93009-3	Ubiquitin carboxy	USP7
NaN	NaN	22.6957	NaN	22.6919	NaN	NaN	NaN	NaN	NaN	NaN	NaN	P40818;P40818-2	P40818;P40818-2	Ubiquitin carboxy	USP8
28.3689	26.9652	27.5897	28.1301	27.18	27.4676	27.1894	27.1114	27.4629	27.5978	27.3673	27.2079	Q93008;Q93008-1	Q93008;Q93008-1	Probable ubiquitir	USP9X
NaN	NaN	NaN	22.3973	NaN	NaN	NaN	NaN	NaN	NaN	NaN	NaN	Q9BVJ6;Q9BVJ6-2	Q9BVJ6;Q9BVJ6-2	U3 small nucleola	UTP14A;UTP14C
24.4662	24.2812	24.1628	24.9378	24.7536	24.73	NaN	NaN	NaN	24.5594	25.2282	NaN	Q8TED0;Q8TED0-	Q8TED0;Q8TED0-	U3 small nucleola	UTP15
25.9065	24.1438	25.0338	25.6256	24.7726	24.7629	23.0854	NaN	NaN	24.7966	25.5106	24.1057	Q9Y5J1;J3KSR7;J3	Q9Y5J1	U3 small nucleola	UTP18
26.332	23.6271	24.7711	27.5039	24.6391	24.6706	22.8862	NaN	24.7483	23.542	23.0695	NaN	O75691	O75691	Small subunit pro	UTP20
NaN	26.5037	26.864	NaN	26.5005	NaN	NaN	NaN	NaN	NaN	NaN	NaN	Q9BRU9;E5RH13;I	Q9BRU9;E5RH13;I	rRNA-processing p	UTP23
22.4277	NaN	NaN	22.037	NaN	NaN	NaN	NaN	NaN	NaN	NaN	NaN	Q9NYH9	Q9NYH9	U3 small nucleola	UTP6
NaN	NaN	NaN	NaN	NaN	NaN	NaN	NaN	NaN	NaN	21.5219	NaN	H0Y337;Q5JT44	H0Y337		UTRN
27.9501	28.0056	28.9774	28.3731	28.4578	29.288	28.5219	27.6867	27.9895	29.8981	29.4637	29.4567	P46939;P46939-2	P46939;P46939-2	Utrophin	UTRN
22.3988	NaN	21.9268	22.3395	NaN	NaN	21.9834	NaN	NaN	22.0009	22.0834	NaN	Q9P2Y5;E9PR71;E	Q9P2Y5;E9PR71;E	UV radiation resis	UVRAG
25.0972	25.0613	24.8037	24.5607	25.2143	23.2959	27.1043	25.9307	25.9284	26.8679	25.9618	NaN	Q8NBZ7;Q8NBZ7-	Q8NBZ7;Q8NBZ7-	UDP-glucuronic ac	UXS1
26.067	25.4401	25.7375	26.1055	25.5919	25.6995	25.9639	26.3375	26.0502	26.2562	26.1128	25.5369	Q08AM6;H3BUU8	Q08AM6	Protein VAC14 ho	VAC14
24.1771	23.8746	23.9176	23.7709	23.5237	23.708	23.1616	23.8605	24.4504	NaN	NaN	NaN	P63027;J3QRU4;F	P63027;J3QRU4;F	Vesicle-associatec	VAMP2
28.2824	27.8746	28.2879	28.3937	27.6877	28.4808	27.5303	27.418	27.8218	27.2671	27.501	27.4384	Q15836;K7ENK9;K	Q15836;K7ENK9;K	Vesicle-associatec	VAMP3;VAMP2
24.6189	23.7513	24.6049	24.1377	23.3843	24.1631	24.397	NaN	NaN	23.4022	NaN	NaN	P51809;P51809-3	P51809;P51809-3	Vesicle-associatec	VAMP7
28.6589	28.6204	28.8963	28.3385	28.6125	28.9498	27.8357	26.6922	27.4185	27.9449	27.5076	27.828	Q9BV40;B8ZZT4;C	Q9BV40;B8ZZT4	Vesicle-associatec	VAMP8
25.4002	25.5357	26.4384	25.7468	25.3396	26.7309	25.6561	NaN	25.1368	26.8374	26.1777	26.1059	Q8TAA9;Q8TAA9-	Q8TAA9;Q8TAA9-	Vang-like protein	VANGL1
28.7118	27.8425	28.3227	28.165	27.2132	27.5826	28.7057	26.899	26.4402	28.6249	27.8312	26.8617	Q9P0L0;Q9P0L0-2	Q9P0L0;Q9P0L0-2	Vesicle-associatec	VAPA
26.2121	26.0389	25.8342	25.872	25.5382	25.5946	25.8539	25.2304	25.1061	24.9929	25.3172	24.9099	O95292;E5RK64;C	O95292	Vesicle-associatec	VAPB
31.0456	29.7681	30.3274	30.9578	30.4168	30.5435	29.8102	29.7868	29.6295	30.3825	30.6831	29.7598	P26640;P26640-2	P26640	Valine--tRNA ligas	VARS
28.3541	28.802	28.7112	28.4455	28.8408	28.7295	29.5988	29.2657	29.5508	29.3359	29.4122	29.4831	P50552;K7EM16;K	P50552	Vasodilator-stimu	VASP
29.1258	28.9782	29.1851	28.8061	28.961	29.0455	30.0301	29.0283	29.6473	29.5765	29.2351	29.5331	Q99536;Q99536-3	Q99536;Q99536-3	Synaptic vesicle m	VAT1
22.0786	NaN	NaN	NaN	NaN	NaN	NaN	NaN	NaN	22.6576	NaN	NaN	P52735;P52735-3	P52735;P52735-3	Guanine nucleotic	VAV2
25.6074	26.4921	26.0977	25.2877	26.7661	26.2858	25.955	27.053	26.5741	25.8099	26.5561	26.6371	P61758;B4DWR3	P61758;B4DWR3	Prefoldin subunit	VBP1
30.3315	30.2292	30.4762	30.3032	29.8969	30.3126	30.9154	30.1022	30.1019	30.7002	30.1733	30.3724	P18206;P18206-2	P18206;P18206-2	Vinculin	VCL
30.966	31.5105	31.0933	30.6066	31.1855	31.0264	31.4407	31.7656	31.1989	31.2438	31.3329	31.271	P55072;C9JUP7;C	P55072	Transitional endo	VCP
23.2651	NaN	22.8472	23.1044	NaN	23.1074	NaN	NaN	NaN	23.2194	23.103	NaN	Q96JH7	Q96JH7	Deubiquitinating p	VCPIP1
29.1012	29.4547	29.0706	29.3388	29.3098	29.3055	29.0035	29.5047	30.3403	27.8836	28.4436	29.3867	P21796;C9JI87	P21796;C9JI87	Voltage-depender	VDAC1
28.1583	28.5358	28.4348	28.6351	28.8084	28.7234	28.0097	28.9803	30.2548	27.3744	27.6563	29.1235	P45880;P45880-2	P45880;P45880-2	Voltage-depender	VDAC2
26.2229	26.6349	26.6259	26.7178	26.9075	26.9493	25.7839	27.2766	28.209	25.763	26.3	27.3644	Q9Y277;Q9Y277-2	Q9Y277;Q9Y277-2	Voltage-depender	VDAC3
23.5242	26.1442	23.4812	22.684	24.2045	23.5082	29.7673	29.785	NaN	26.2125	23.9114	NaN	H0YBI8;P15692-1	H0YBI8;P15692-1	Vascular endothel	VEGFA
NaN	23.5788	NaN	NaN	NaN	NaN	24.3139	25.3354	NaN	NaN	NaN	NaN	P49767	P49767	Vascular endothel	VEGFC
33.6893	34.5651	33.8512	33.4092	34.1737	33.5938	33.9779	34.5618	33.6593	33.8944	33.8266	33.6814	P08670;B0YJC4;B(	P08670;B0YJC4	Vimentin	VIM
23.4461	23.3939	NaN	NaN	23.4347	NaN	23.7785	NaN	NaN	NaN	NaN	NaN	Q9H9C1;G3V4K3;	Q9H9C1;G3V4K3;	Spermatogenesis-	VIPAS39
NaN	25.8107	NaN	NaN	NaN	NaN	NaN	NaN	NaN	NaN	NaN	NaN	O95497	O95497	Pantetheinase	VNN1
22.4861	NaN	NaN	21.777	NaN	NaN	NaN	NaN	NaN	NaN	NaN	NaN	Q9Y4B6;Q9Y4B6-3	Q9Y4B6;Q9Y4B6-3	Protein VPRBP	VPRBP
26.077	25.1832	25.8863	26.2959	26.0712	25.6682	24.2489	24.7733	23.1068	25.0676	25.1297	23.2458	A0A087WXL6;Q9I	A0A087WXL6;Q9I	Vacuolar protein s	VPS11
23.6823	NaN	NaN	23.383	NaN	NaN	NaN	NaN	NaN	NaN	NaN	NaN	Q96RL7;Q96RL7-2	Q96RL7;Q96RL7-2	Vacuolar protein s	VPS13A
23.3983	22.8953	23.4538	22.6282	22.9583	22.8417	NaN	NaN	NaN	22.628	NaN	NaN	Q709C8;Q709C8-4	Q709C8;Q709C8-4	Vacuolar protein s	VPS13C
25.1588	24.2967	24.5605	24.8012	25.0075	24.5638	24.2889	25.4032	24.1951	24.2917	24.5551	24.1857	Q9H269;Q9H269-	Q9H269;Q9H269-	Vacuolar protein s	VPS16
25.9338	24.0221	25.2464	25.63	24.8835	24.8552	24.2779	NaN	NaN	24.7198	24.0723	NaN	Q9P253;H0YMC9	Q9P253	Vacuolar protein s	VPS18
NaN	22.5977	22.6401	22.5267	22.1344	22.8327	NaN	NaN	NaN	22.6327	22.9009	NaN	Q9BRG1;K7ENE3;I	Q9BRG1;K7ENE3;I	Vacuolar protein-:	VPS25

27.2715	26.8482	26.9024	26.7347	26.5131	26.7016	27.8209	27.051	27.031	27.2857	26.9682	26.7132	O75436;S4R3Q6;(	O75436;S4R3Q6;(	Vacuolar protein s	VPS26A
21.8125	NaN	21.9418	21.9276	NaN	NaN	23.1476	NaN	NaN	21.5633	NaN	NaN	Q4G0F5;H0YCI7	Q4G0F5	Vacuolar protein s	VPS26B
25.0699	25.9597	25.6978	24.8994	25.6388	25.5198	25.5889	26.0726	26.6623	24.6313	24.9394	25.306	Q9UK41;Q9UK41-	Q9UK41;Q9UK41-	Vacuolar protein s	VPS28
27.0236	26.9123	26.6757	26.3085	26.5073	26.6375	27.8637	27.3139	27.1847	27.2168	27.1745	26.7904	Q9UBQ0;F8VXU5;	Q9UBQ0;F8VXU5;	Vacuolar protein s	VPS29
24.3642	24.3617	24.2321	24.3857	24.6246	24.0719	23.855	NaN	23.952	24.067	23.748	23.6729	Q96AX1;H3BMM5	Q96AX1;H3BMM5	Vacuolar protein s	VPS33A
26.5812	26.0082	26.2857	26.2119	26.1074	26.1132	25.8958	25.6903	26.2494	26.2315	26.1041	25.9103	Q9H267;F5H008;(	Q9H267;F5H008;(	Vacuolar protein s	VPS33B
28.8075	28.1207	28.3088	28.4174	28.0232	28.2626	28.8222	28.7828	28.8432	28.788	28.5308	28.4884	Q96QK1;I3L4S0;I3	Q96QK1	Vacuolar protein s	VPS35
25.0095	25.0121	24.7442	24.7489	25.2249	24.8626	24.9616	25.2713	25.4007	25.0532	25.2953	25.2494	Q86VN1;Q86VN1-	Q86VN1;Q86VN1-	Vacuolar protein-s	VPS36
24.9388	25.4533	24.8424	24.4637	24.9672	24.5336	25.4536	25.281	25.3837	24.5158	NaN	24.8814	Q9H9H4;F5H1F6;I	Q9H9H4;F5H1F6;I	Vacuolar protein s	VPS37B
23.9771	24.7572	24.5423	23.9244	24.4005	24.1928	24.3169	24.6833	NaN	NaN	NaN	23.9349	A5D8V6;F5H4Q5	A5D8V6;F5H4Q5	Vacuolar protein s	VPS37C
25.2189	25.3805	25.2864	25.1247	25.2952	25.2331	25.4417	NaN	24.9094	24.8114	24.7715	24.859	Q9NRW7;A0A087	Q9NRW7;A0A087	Vacuolar protein s	VPS45
26.4893	26.9421	26.7601	26.5833	26.5552	26.7552	27.971	27.5447	28.7889	27.3895	27.4943	27.8894	Q9UN37	Q9UN37	Vacuolar protein s	VPS4A
25.6885	25.6559	25.6011	25.2104	25.1781	25.6249	25.9455	25.794	26.4821	25.3944	25.4839	25.7804	O75351;K7EL71;K	O75351	Vacuolar protein s	VPS4B
24.8811	24.1885	24.6991	25.0112	24.6439	24.8154	24.2735	24.0667	24.3817	24.5634	24.7196	NaN	Q9UID3;Q9UID3-2	Q9UID3;Q9UID3-2	Vacuolar protein s	VPS51
26.1914	25.2945	25.7523	25.7768	25.371	25.7731	25.8401	23.5949	25.1934	26.0173	25.6572	25.337	Q8N1B4;Q8N1B4-	Q8N1B4;Q8N1B4-	Vacuolar protein s	VPS52
24.7198	23.6243	24.2838	24.6849	24.0538	24.4398	23.8231	24.6608	NaN	23.9485	23.8798	23.7587	Q5VIR6-4;Q5VIR6	Q5VIR6-4;Q5VIR6	Vacuolar protein s	VPS53
22.8125	22.536	22.2004	22.8317	22.0297	22.2435	NaN	NaN	NaN	NaN	NaN	NaN	Q8N3P4;C9JJN9;C	Q8N3P4;C9JJN9;C	Vacuolar protein s	VPS8
25.6261	24.01	24.65	25.8254	24.0397	24.2199	NaN	NaN	NaN	24.9727	24.8156	NaN	Q99986;H0YJ50;H	Q99986;H0YJ50	Serine/threonine-	VRK1
27.3618	27.355	27.3343	26.9999	26.7882	27.2052	27.8212	26.7553	27.8856	27.1067	26.7376	27.6473	Q9NP79;A0A087V	Q9NP79;A0A087V	Vacuolar protein s	VTA1
24.6643	24.6752	25.2454	25.3081	24.6592	25.1721	24.921	24.8256	25.8651	24.6771	24.6794	24.479	Q96AJ9;Q96AJ9-1	Q96AJ9;Q96AJ9-1	Vesicle transport	VTI1A
25.0251	24.768	25.02	25.0746	24.7532	25.2647	24.4034	24.3514	25.5148	24.3169	24.6595	25.3027	Q9UEU0;Q9UEU0	Q9UEU0;Q9UEU0	Vesicle transport	VTI1B
22.3493	22.3583	22.4116	22.553	22.7475	22.4918	NaN	NaN	NaN	NaN	NaN	NaN	Q96SY0;H3BRY6;E	Q96SY0;H3BRY6;E	von Willebrand fa	VWA9
25.2201	NaN	24.9001	25.3168	25.5865	25.3579	NaN	NaN	NaN	25.4292	25.4271	25.1352	Q7Z5K2;Q7Z5K2-2	Q7Z5K2;Q7Z5K2-2	Wings apart-like p	WAPAL
28.5787	28.7101	28.2542	28.3463	28.5273	28.1965	28.506	28.5826	28.5615	28.21	28.1771	28.5797	P23381;P23381-2	P23381;P23381-2	Tryptophan--tRNA	WARS
25.705	25.9115	25.7646	25.1978	25.8436	25.5551	25.3356	25.5742	25.4737	25.6635	26.13	25.7126	Q9Y6W5;Q9Y6W5	Q9Y6W5;Q9Y6W5	Wiskott-Aldrich sy	WASF2
25.0726	NaN	NaN	NaN	26.8234	NaN	NaN	NaN	NaN	NaN	NaN	NaN	A8K0Z3;A0A096LF	A8K0Z3;A0A096LF	WAS protein fami	WASH1;WASH3P;\
23.5799	23.7874	23.5346	23.5	23.638	23.9713	23.7817	23.5236	NaN	23.3623	23.2748	24.5423	O00401	O00401	Neural Wiskott-Al	WASL
24.2926	23.6629	23.6201	23.5699	23.85	23.7027	23.0606	NaN	23.5643	24.1592	24.3688	24.4442	Q9Y2W2;F5H5G4;	Q9Y2W2;F5H5G4	WW domain-bind	WBP11
23.2928	23.6389	23.1277	22.6413	NaN	22.7456	NaN	NaN	NaN	NaN	NaN	NaN	Q969T9;A6NG10;I	Q969T9;A6NG10;I	WW domain-bind	WBP2
23.3814	22.8868	NaN	23.1554	23.0153	23.2289	NaN	NaN	NaN	22.9893	23.1499	NaN	Q96I51;Q96I51-2;	Q96I51;Q96I51-2;	Williams-Beuren s	WBSCR16
24.1459	22.889	23.0892	23.5252	22.4968	22.7198	NaN	NaN	NaN	23.3504	23.2372	NaN	O43709;H7C170;(	O43709;H7C170;(	Probable 18S rRN	WBSCR22
25.4204	24.839	24.9442	25.0421	24.4818	25.2253	25.2927	NaN	NaN	25.5347	24.8878	NaN	Q8IWB7;C9JJ54	Q8IWB7;C9JJ54	WD repeat and FY	WDFY1
24.9832	23.41	23.7684	24.4162	23.931	23.5686	24.1241	NaN	23.7863	23.8618	23.4451	NaN	Q8IZQ1;Q8IZQ1-2	Q8IZQ1;Q8IZQ1-2	WD repeat and FY	WDFY3
26.2162	26.1952	26.3613	26.5425	26.5326	26.1782	23.7259	25.5059	24.7882	26.1822	26.0225	26.5019	O75717;O75717-2	O75717;O75717-2	WD repeat and HI	WDHD1
30.7505	31.3676	31.3335	30.6551	31.3809	31.4219	31.3546	31.2322	31.0951	31.8079	31.7057	31.2921	O75083;D6RD66;(	O75083;D6RD66;(	WD repeat-contai	WDR1
26.7589	26.0141	26.2491	26.8359	26.358	26.2312	25.7736	26.1963	26.3819	26.2987	26.3079	25.887	Q9BZH6;S4R3Z0;S	Q9BZH6	WD repeat-contai	WDR11
25.0328	25.3484	25.4173	25.8474	26.1316	25.3947	24.5975	24.1243	NaN	25.9909	26.0238	25.1087	Q9GZL7	Q9GZL7	Ribosome biogen	WDR12
25.021	25.0512	25.0309	25.4486	25.5787	25.0568	NaN	NaN	NaN	24.7788	25.3001	NaN	Q9BV38;U3KQC1;	Q9BV38;U3KQC1;	WD repeat-contai	WDR18
25.0654	25.4887	25.9622	25.2322	25.7167	25.8904	25.1704	25.0547	26.3204	26.2329	25.7397	26.5092	A0A088AWN2;Q8	A0A088AWN2;Q8	WD repeat-contai	WDR20
24.6718	24.8022	25.081	25.4064	25.4554	25.0252	23.8671	24.6221	24.9863	24.6543	24.7464	24.9746	Q9H7D7-2;Q9H7D	Q9H7D7-2;Q9H7D	WD repeat-contai	WDR26
25.1013	24.1206	24.6782	25.6724	24.7251	25.2762	24.3063	NaN	23.7792	24.6338	25.7203	NaN	Q9UNX4	Q9UNX4	WD repeat-contai	WDR3
24.9571	23.7468	23.0964	23.9149	23.8989	23.1416	23.508	NaN	NaN	24.0639	23.356	NaN	Q9C0J8;Q9C0J8-2	Q9C0J8	pre-mRNA 3 end p	WDR33
27.1855	25.0796	25.8274	27.2055	25.6151	26.0457	24.0766	NaN	24.6951	26.4812	26.6664	23.4914	Q8NI36;A0A0A0M	Q8NI36;A0A0A0M	WD repeat-contai	WDR36
24.4025	24.3007	24.4707	24.1151	24.5578	24.211	24.1227	NaN	NaN	24.3336	24.3634	NaN	Q9Y2I8;Q9Y2I8-2;	Q9Y2I8;Q9Y2I8-2	WD repeat-contai	WDR37
22.1128	NaN	21.8758	NaN	21.8613	NaN	NaN	NaN	NaN	NaN	NaN	NaN	Q9HAD4;H0Y9Z3;	Q9HAD4;H0Y9Z3;	WD repeat-contai	WDR41
24.541	24.2957	24.4111	25.2214	24.7441	24.546	24.5345	NaN	NaN	24.5187	24.7189	23.9646	Q15061;C9IZK7;C	Q15061	WD repeat-contai	WDR43
25.4632	25.183	25.2472	24.8514	25.2425	25.2941	25.2662	NaN	25.2912	26.0326	25.936	25.8764	Q5JSH3;H7BY83;C	Q5JSH3;H7BY83;C	WD repeat-contai	WDR44
24.4287	23.6523	23.9555	23.9568	23.5841	23.7283	23.4429	NaN	NaN	24.1336	23.9113	23.673	Q9Y484;C9J471;A	Q9Y484;C9J471;A	WD repeat domai	WDR45
NaN	NaN	23.5833	NaN	NaN	NaN	NaN	NaN	NaN	NaN	NaN	NaN	Q5MNZ6;I3L3A5;I	Q5MNZ6	WD repeat domai	WDR45B
24.6316	23.1908	23.4767	24.7331	22.66	23.3281	NaN	NaN	NaN	23.0639	23.4193	NaN	O15213;H0Y6G3;/	O15213;H0Y6G3;/	WD repeat-contai	WDR46
23.8149	NaN	NaN	23.1842	23.3461	NaN	NaN	NaN	NaN	23.5808	23.9307	NaN	O94967;A0A0A0N	O94967;A0A0A0N	WD repeat-contai	WDR47
24.9151	25.8064	25.6036	25.3849	25.6927	25.7949	25.5292	25.4299	25.5264	25.9755	25.8717	25.957	Q8TAF3;Q8TAF3-	Q8TAF3;Q8TAF3-	WD repeat-contai	WDR48
26.4996	26.2726	25.6782	26.9569	26.4768	26.2255	24.8966	NaN	NaN	26.1732	26.3048	25.835	P61964;V9GZ59;V	P61964;V9GZ59	WD repeat-contai	WDR5
22.0689	NaN	22.1402	22.2471	NaN	NaN	NaN	NaN	NaN	NaN	NaN	NaN	Q9H977;C9J016;B	Q9H977;C9J016;B	WD repeat-contai	WDR54
23.9711	NaN	NaN	23.5306	NaN	NaN	NaN	NaN	NaN	23.3834	23.7695	NaN	Q9H6Y2;G3V1J0;(	Q9H6Y2	WD repeat-contai	WDR55
27.264	25.797	26.3266	26.992	25.9206	26.1869	25.6765	25.1315	25.9112	26.2817	26.5233	25.9535	Q9NNW5;E9PDU5	Q9NNW5;E9PDU5	WD repeat-contai	WDR6
25.5039	24.1764	24.4357	24.8249	24.6536	24.9747	23.5504	23.5229	NaN	25.7283	26.0189	NaN	Q9GZS3;H0YN81;I	Q9GZS3;H0YN81;I	WD repeat-contai	WDR61
23.0676	22.4782	22.5523	22.5112	NaN	NaN	NaN	NaN	22.862	22.9993	22.5159	NaN	O43379-4;O43379	O43379-4;O43379	WD repeat-contai	WDR62
27.3269	27.1778	26.6029	26.9474	26.9828	26.7261	27.2172	NaN	NaN	27.6242	NaN	NaN	B1ANS9;B1ANS9-	B1ANS9;B1ANS9-	WD repeat-contai	WDR64
23.3415	NaN	NaN	23.5543	NaN	NaN	NaN	NaN	NaN	23.5321	23.7934	NaN	Q9NW82;D6RIW8	Q9NW82	WD repeat-contai	WDR70

23.4442	23.6806	23.7195	24.169	23.9429	23.893	NaN	NaN	NaN	23.3917	24.3229	23.3395	Q6RFH5;Q6RFH5-	Q6RFH5;Q6RFH5-	WD repeat-contai	WDR74
24.6039	23.8512	24.7722	25.4447	25.0308	24.7581	NaN	NaN	24.4583	24.4772	24.8697	24.6969	Q8IWA0	Q8IWA0	WD repeat-contai	WDR75
27.6933	27.4816	27.1548	27.858	27.427	27.4262	27.3745	27.1438	26.7148	27.2582	27.0738	26.902	Q9BQA1;Q9BQA1	Q9BQA1;Q9BQA1	Methylosome pro	WDR77
27.3862	25.9162	25.9444	26.2773	25.8642	25.8742	26.0679	24.5121	25.7577	27.5842	27.2359	26.2999	Q6UXN9;C9JBU3	Q6UXN9	WD repeat-contai	WDR82
NaN	NaN	NaN	22.2814	NaN	NaN	NaN	NaN	NaN	NaN	NaN	NaN	Q96FK6;G3V4B8	Q96FK6;G3V4B8	WD repeat-contai	WDR89
25.0307	24.8298	24.2725	24.2627	24.3621	24.0863	24.4992	24.3746	NaN	25.1127	25.5183	24.6747	Q96MX6;Q96MX6	Q96MX6;Q96MX6	WD repeat-contai	WDR92;DKFZp434
23.7669	23.8952	23.927	23.5109	23.935	23.9744	24.6163	23.8178	24.3229	24.2098	23.6699	24.2581	O76024;H0Y9G5	O76024	Wolframin	WFS1
22.8651	21.6346	21.7761	21.6784	NaN	NaN	NaN	NaN	NaN	NaN	NaN	NaN	Q8TF30;Q1A5X7;(	Q8TF30	WASP homolog-a:	WHAMM
NaN	22.9929	22.8893	NaN	23.0499	NaN	NaN	NaN	NaN	NaN	NaN	NaN	Q9BRP8;Q9BRP8-	Q9BRP8;Q9BRP8-	Partner of Y14 an	WIBG
23.9344	24.5398	24.6372	23.0386	NaN	23.0164	23.1106	24.3292	NaN	NaN	23.0192	NaN	Q5MNZ9;Q5MNZ!	Q5MNZ9;Q5MNZ!	WD repeat domai	WIPI1
24.9751	23.9809	24.2238	23.9051	23.9725	23.621	24.2881	NaN	NaN	24.6671	24.8636	NaN	Q9Y4P8;Q9Y4P8-5	Q9Y4P8;Q9Y4P8-5	WD repeat domai	WIPI2
NaN	NaN	NaN	NaN	NaN	NaN	NaN	NaN	NaN	NaN	NaN	20.7737	Q5T9L3;Q5T9L3-2	Q5T9L3;Q5T9L3-2	Protein wntless h	WLS
22.5704	22.3572	22.8523	23.2272	22.4268	NaN	NaN	NaN	NaN	NaN	NaN	NaN	Q9H4A3;Q9H4A3-	Q9H4A3;Q9H4A3-	Serine/threonine-	WNK1
22.6913	NaN	22.39	22.6411	22.4271	NaN	NaN	NaN	NaN	22.9217	23.2382	NaN	Q9BUR4;E9PMG4	Q9BUR4;E9PMG4	Telomerase Cajal	WRAP53
24.728	24.3659	25.049	25.6922	25.0009	25.2763	NaN	NaN	NaN	25.0385	25.199	25.3386	Q96S55;Q96S55-2	Q96S55;Q96S55-2	ATPase WRNIP1	WRNIP1
30.4913	30.5676	30.6237	30.9101	31.3585	30.562	NaN	NaN	NaN	NaN	NaN	30.973	Q15007;Q15007-2	Q15007	Pre-mRNA-splicin	WTAP
23.1156	23.5297	23.3028	23.0256	NaN	23.4227	NaN	NaN	24.4585	22.9493	23.1085	24.0236	O00308;O00308-2	O00308;O00308-2	NEDD4-like E3 ubi	WWP2
26.4873	24.7497	25.6094	26.6968	25.5675	25.9278	NaN	NaN	23.0303	25.3612	26.0543	25.4387	Q9HCS7	Q9HCS7	Pre-mRNA-splicin	XAB2
27.2803	26.9409	27.0073	26.7335	26.8401	27.0648	27.2496	26.7946	26.9007	26.9915	26.6867	27.1002	Q9NQW7;Q9NQW	Q9NQW7;Q9NQW	Xaa-Pro aminopep	XPNPEP1
27.7967	27.3896	27.795	27.8367	27.515	27.7806	27.7833	27.7979	28.031	27.9931	27.8392	28.2353	O14980;C9JKM9;(	O14980	Exportin-1	XPO1
23.2147	22.4506	22.8832	22.9151	22.8484	NaN	NaN	NaN	NaN	22.9245	23.7221	NaN	Q9C0E2;F2Z2X4	Q9C0E2;F2Z2X4	Exportin-4	XPO4
28.389	26.7236	27.5861	28.325	27.1912	27.7292	26.7739	27.1086	26.8053	27.8614	27.5013	27.3272	Q9HAV4;E2QRM3	Q9HAV4	Exportin-5	XPO5
27.5913	26.0874	26.7048	27.3069	26.5633	27.0128	26.5237	26.363	26.2398	27.3857	27.0767	26.4295	Q9UIA9;E7ESC6;H	Q9UIA9;E7ESC6	Exportin-7	XPO7
25.7733	24.2773	24.997	25.1969	25.332	24.4251	24.6968	24.985	24.6618	24.9518	25.1026	NaN	O43592;F8WDU6;	O43592	Exportin-T	XPOT
22.5234	NaN	22.1479	22.5543	21.9323	NaN	NaN	NaN	NaN	23.3176	NaN	NaN	Q9UBH6;Q9UBH6	Q9UBH6;Q9UBH6	Xenotropic and pc	XPR1
NaN	NaN	21.351	21.6096	NaN	NaN	NaN	NaN	NaN	20.9998	20.9171	NaN	Q13426;Q13426-3	Q13426;Q13426-3	DNA repair protei	XRCC4
29.7224	29.436	29.3548	29.7975	29.902	29.6943	28.6885	28.5321	28.7197	29.7946	30.0335	29.3044	P13010;C9JZ81;H	P13010	X-ray repair cross-	XRCC5
29.3679	29.2961	29.3786	29.8288	29.4793	29.6961	29.1722	29.1014	28.455	29.6547	29.8852	29.5207	P12956;B1AHC9;F	P12956;B1AHC9;F	X-ray repair cross-	XRCC6
25.6109	NaN	25.2543	25.7849	25.8928	25.851	NaN	NaN	NaN	25.421	24.9088	NaN	Q8IZH2;Q8IZH2-2	Q8IZH2;Q8IZH2-2	5-3 exoribonuclea	XRN1
26.8086	26.0981	26.768	27.1406	26.6009	26.7352	25.7365	25.6252	26.4699	27.125	27.2554	27.3241	Q9H0D6;Q9H0D6	Q9H0D6;Q9H0D6	5-3 exoribonuclea	XRN2
NaN	NaN	NaN	NaN	NaN	NaN	NaN	NaN	NaN	24.9172	23.972	NaN	O75191;O75191-2	O75191;O75191-2	Xylulose kinase	XYLB
26.072	26.9517	26.3049	25.4797	26.3921	25.8819	25.3562	25.944	26.116	24.2398	25.2563	25.8565	P46937;P46937-2	P46937;P46937-2	Transcriptional co	YAP1
29.1351	28.3985	28.3289	28.7866	28.2597	28.3454	27.8966	27.5711	27.3097	27.916	27.6623	27.3558	P54577;A0A0C4D	P54577;A0A0C4D	Tyrosine--tRNA lig	YARS
24.9642	24.0003	24.073	25.011	24.014	24.0429	NaN	NaN	NaN	23.8968	24.0008	NaN	Q9Y2Z4;H0YHS6	Q9Y2Z4;H0YHS6	Tyrosine--tRNA lig	YARS2
30.0316	29.1182	29.216	29.5115	28.6687	28.7455	28.9406	30.0704	28.7201	28.5695	28.6975	28.6772	P67809;A0A087X1	P67809;A0A087X1	Nuclease-sensitiv	YBX1
23.7606	NaN	NaN	23.5507	NaN	NaN	NaN	NaN	NaN	NaN	NaN	NaN	P16989;P16989-2	P16989;P16989-2	Y-box-binding pro	YBX3
29.5284	29.2743	29.9144	29.5544	29.2055	30.018	29.6744	29.2569	29.4927	30.5948	30.07	29.9646	P07947;J3QRU1;P	P07947;J3QRU1	Tyrosine-protein k	YES1
NaN	NaN	23.0021	22.7605	NaN	NaN	NaN	NaN	NaN	22.5513	NaN	22.6768	O95070;A6NGW1	O95070;A6NGW1	Protein YIF1A	YIF1A
NaN	NaN	NaN	22.7145	NaN	NaN	NaN	NaN	NaN	NaN	NaN	NaN	Q5BJH7;Q5BJH7-6	Q5BJH7;Q5BJH7-6	Protein YIF1B	YIF1B
24.8147	24.4011	24.4807	24.5527	23.5601	24.173	24.0318	23.7285	NaN	23.7396	23.3449	23.8503	Q9GZM5;D6RFI3;I	Q9GZM5;D6RFI3;I	Protein YIPF3;Pro	YIPF3
23.9668	22.9862	23.0256	23.5193	23.1714	NaN	NaN	NaN	NaN	NaN	22.8581	NaN	Q9BSR8;H7C0D5	Q9BSR8;H7C0D5	Protein YIPF4	YIPF4
23.5816	23.072	23.734	23.4736	22.8747	NaN	NaN	NaN	NaN	NaN	NaN	NaN	Q969M3;Q969M3	Q969M3;Q969M3	Protein YIPF5;Pro	YIPF5
24.8333	24.3582	25.0325	25.1341	24.3149	25.1172	24.5301	NaN	25.0795	24.5661	24.3153	24.6799	Q96EC8;Q96EC8-2	Q96EC8;Q96EC8-2	Protein YIPF6;Pro	YIPF6
24.7789	24.5275	25.0611	24.6038	24.1335	24.9121	25.107	NaN	24.3612	25.2265	23.8391	23.7455	O15498;O15498-2	O15498;O15498-2	Synaptobrevin ho	YKT6
24.2189	24.528	24.0826	24.1629	24.2293	24.2433	NaN	NaN	NaN	24.0397	24.5715	25.9641	P49750-4;P49750	P49750-4;P49750	YLP motif-contain	YLPM1
NaN	NaN	NaN	21.1941	NaN	NaN	NaN	NaN	NaN	NaN	NaN	NaN	Q96TA2;Q96I63;C	Q96TA2;Q96I63;C	ATP-dependent zi	YME1L1
23.534	23.0506	23.0465	23.4733	23.4969	23.3631	23.1219	NaN	NaN	23.5088	NaN	NaN	P62699	P62699	Protein yippee-lik	YPEL5
23.638	NaN	23.1026	23.3195	NaN	NaN	NaN	NaN	NaN	22.9426	23.2075	NaN	Q86U90	Q86U90	YrdC domain-cont	YRDC
25.7724	24.7668	25.3191	25.6187	25.3489	25.5587	NaN	NaN	NaN	24.5397	25.4875	25.1021	Q96MU7;Q96MU	Q96MU7;Q96MU	YTH domain-conta	YTHDC1
24.1474	22.8243	23.3211	24.3442	23.7228	NaN	NaN	NaN	NaN	23.1619	23.5906	NaN	Q9H6S0;D6RA70;I	Q9H6S0	Probable ATP-dep	YTHDC2
26.6891	25.8822	26.2144	26.5582	25.9361	26.1467	25.5016	25.4016	NaN	26.1107	26.0564	NaN	Q9BYJ9;Q9BYJ9-2	Q9BYJ9	YTH domain-conta	YTHDF1
NaN	NaN	NaN	NaN	NaN	NaN	NaN	NaN	NaN	NaN	NaN	21.6729	Q7Z739;A0A087X	Q7Z739;A0A087X	YTH domain-conta	YTHDF3
30.8011	32.191	31.8919	30.9439	31.8533	31.754	32.0116	31.835	31.6862	32.0483	31.8716	31.9116	P31946;Q4VY19;A	P31946;Q4VY19;A	14-3-3 protein bet	YWHAB
NaN	27.2186	26.6227	NaN	28.9183	26.8247	NaN	30.1109	30.3735	NaN	27.818	30.5449	P31946-2	P31946-2	14-3-3 protein bet	YWHAB
31.6353	31.8085	31.7956	31.371	31.1887	31.6556	32.3846	30.941	31.1542	32.1178	31.639	31.6779	P62258;P62258-2	P62258;P62258-2	14-3-3 protein eps	YWHAE
29.4053	30.3214	30.1634	29.2033	29.9954	30.1382	30.3495	30.2059	30.4232	30.0223	30.2418	30.7375	P61981	P61981	14-3-3 protein gar	YWHAG
29.1888	29.7347	29.3911	28.6261	29.1531	29.4553	29.7885	29.2829	29.3844	29.3853	29.3236	29.2403	Q04917;A2IDB2;F	Q04917;A2IDB2	14-3-3 protein eta	YWHAH
31.093	31.0341	31.1578	30.7377	30.4195	31.0927	31.6028	31.0924	30.6507	31.5263	30.9259	30.6364	P27348;E9PG15	P27348;E9PG15	14-3-3 protein the	YWHAQ
32.8875	33.7121	33.5018	32.7135	33.2562	33.3932	34.4579	33.6792	33.4146	33.6642	33.4912	33.465	P63104;E7EX29;E	P63104;E7EX29;E	14-3-3 protein zet	YWHAZ
22.4606	22.6445	22.8481	23.0372	22.8702	NaN	NaN	NaN	NaN	NaN	NaN	NaN	P25490;H0YJV7;O	P25490;H0YJV7	Transcriptional re	YY1

26.5231	25.4653	25.6668	25.9084	24.9243	25.2116	24.7143	24.7923	24.4722	26.1448	25.669	24.726	Q9NYL2	Q9NYL2	Mitogen-activatec	ZAK
23.7261	NaN	22.3937	22.9013	NaN	22.9775	NaN	NaN	NaN	22.3675	NaN	NaN	Q9NYL2-2;D4Q8H	Q9NYL2-2;D4Q8H	Mitogen-activatec	ZAK;pk
22.638	NaN	22.9436	23.023	NaN	NaN	NaN	NaN	NaN	NaN	22.9924	NaN	O75152;E9PBY7;E	O75152;E9PBY7;E	Zinc finger CCCH c	ZC3H11A
23.7942	24.2902	24.5811	24.5923	24.1416	24.4469	NaN	NaN	NaN	23.9808	24.6877	24.1591	Q8WU90;Q8WU9	Q8WU90;Q8WU9	Zinc finger CCCH c	ZC3H15
24.3878	24.6633	24.1723	24.5865	24.7232	24.1194	NaN	NaN	NaN	24.1639	24.7707	24.5194	Q86VM9;E7ERS3;	Q86VM9;E7ERS3;	Zinc finger CCCH c	ZC3H18
NaN	NaN	21.0732	21.443	NaN	NaN	NaN	NaN	NaN	NaN	NaN	NaN	Q9UGR2;Q9UGR2	Q9UGR2;Q9UGR2	Zinc finger CCCH c	ZC3H7B
27.4584	27.1662	27.0175	27.456	27.395	27.3713	27.0158	26.6732	27.294	27.2963	27.0446	27.0361	Q7Z2W4;C9J6P4;(	Q7Z2W4;C9J6P4;(	Zinc finger CCCH-t	ZC3HAV1
23.8848	23.9009	23.735	24.4191	24.0971	24.0378	24.1726	NaN	23.3005	24.9889	NaN	24.0755	Q96H79;Q96H79-	Q96H79	Zinc finger CCCH-t	ZC3HAV1L
25.9728	22.7636	23.3899	24.7676	22.3272	23.1596	NaN	23.9185	NaN	22.4249	22.8466	NaN	Q5VYS8;Q5VYS8-6	Q5VYS8;Q5VYS8-6	Terminal uridylylt	ZCCHC6
23.5314	NaN	22.694	22.7426	22.2367	NaN	NaN	NaN	NaN	NaN	22.5223	NaN	Q6NZY4;Q6NZY4-	Q6NZY4;Q6NZY4-	Zinc finger CCHC c	ZCCHC8
22.863	22.5504	22.4242	22.3919	22.2507	NaN	22.3534	22.4255	NaN	NaN	NaN	NaN	Q8IUH5;H0YIK0;Q	Q8IUH5;H0YIK0;Q	Palmitoyltransfera	ZDHHC17
22.8343	22.968	23.0487	22.8749	NaN	NaN	NaN	23.1309	NaN	23.2004	23.3738	22.7612	Q9NUE0;H0YEN1;	Q9NUE0	Palmitoyltransfera	ZDHHC18
26.278	26.3325	26.6524	26.0951	25.9102	26.5423	25.4031	24.8177	26.2311	26.5818	25.9036	26.3028	Q5W0Z9;B4DRN8	Q5W0Z9;B4DRN8	Probable palmitoy	ZDHHC20
27.1523	27.914	27.9151	26.5505	27.1632	27.2736	27.4936	27.1023	27.7885	27.147	26.6497	27.5714	Q9C0B5;Q9C0B5-	Q9C0B5;Q9C0B5-	Palmitoyltransfera	ZDHHC5
NaN	NaN	NaN	NaN	NaN	NaN	NaN	NaN	NaN	22.2646	NaN	NaN	Q9ULC8;Q9ULC8-	Q9ULC8;Q9ULC8-	Probable palmitoy	ZDHHC8
22.1983	NaN	NaN	NaN	NaN	NaN	NaN	NaN	NaN	NaN	NaN	NaN	Q07352;G3V2D5;(	Q07352;G3V2D5;(	Zinc finger protein	ZFP36L1
22.9204	NaN	NaN	23.8196	NaN	NaN	NaN	NaN	22.974	23.4681	NaN	NaN	O95159;E9PQA5;E	O95159;E9PQA5;E	Zinc finger protein	ZFPL1
24.2282	23.9684	23.8371	24.4059	24.1558	23.7554	NaN	NaN	NaN	23.9723	24.4461	NaN	Q96KR1;H0Y8W1;	Q96KR1	Zinc finger RNA-bi	ZFR
24.7523	24.2413	23.8681	23.7515	23.7388	23.788	24.1794	NaN	NaN	23.6038	23.9958	NaN	Q9HBF4;G3V5N8;	Q9HBF4;G3V5N8;	Zinc finger FYVE d	ZFYVE1
22.6531	NaN	NaN	NaN	NaN	NaN	NaN	NaN	NaN	NaN	NaN	NaN	Q7Z3T8;Q7Z3T8-3	Q7Z3T8;Q7Z3T8-3	Zinc finger FYVE d	ZFYVE16
23.54	23.8925	24.3155	23.2894	23.7012	24.4164	23.7841	24.5406	24.5709	23.2305	23.9354	NaN	Q96K21;H3BRF9;(	Q96K21;H3BRF9;(	Abscission/NoCut	ZFYVE19
20.0577	NaN	NaN	NaN	NaN	NaN	NaN	NaN	NaN	NaN	NaN	NaN	Q9BQ24;Q9BQ24-	Q9BQ24;Q9BQ24-	Zinc finger FYVE d	ZFYVE21
20.7723	NaN	NaN	NaN	NaN	NaN	NaN	NaN	NaN	NaN	NaN	NaN	Q68DK2;Q68DK2-	Q68DK2;Q68DK2-	Zinc finger FYVE d	ZFYVE26
24.024	NaN	NaN	NaN	23.0725	NaN	NaN	NaN	NaN	NaN	NaN	NaN	Q8N5A5;V9GY48;	Q8N5A5;V9GY48;	Zinc finger CCCH-t	ZGPAT
24.8259	24.4023	25.0104	25.2712	24.5883	24.993	25.7312	25.6901	26.4323	25.5795	25.2716	25.8728	O75844	O75844	CAAX prenyl prote	ZMPSTE24
NaN	NaN	NaN	NaN	NaN	NaN	NaN	NaN	NaN	22.2213	NaN	NaN	Q9ULU4;Q9ULU4-	Q9ULU4;Q9ULU4-	Protein kinase C-b	ZMYND8
NaN	23.3449	23.2522	NaN	23.4889	23.4952	NaN	NaN	NaN	NaN	22.958	NaN	O15231;O15231-4	O15231;O15231-4	Zinc finger protein	ZNF185
NaN	NaN	24.69	NaN	NaN	NaN	NaN	NaN	NaN	NaN	NaN	NaN	O15231-2	O15231-2	Zinc finger protein	ZNF185
27.6473	28.6126	28.7586	27.6442	28.4443	28.8635	27.4898	27.3754	27.3179	27.9937	28.0861	28.2987	O15231-3;H0Y704	O15231-3;H0Y704	Zinc finger protein	ZNF185
24.5938	24.5913	23.9668	24.473	24.3447	24.3623	23.6309	NaN	NaN	NaN	NaN	NaN	O43670;O43670-2	O43670;O43670-2	BUB3-interacting	ZNF207
23.9724	23.91	24.0429	24.2372	24.0772	24.0935	NaN	NaN	NaN	24.0148	24.1877	24.1453	Q5BKZ1;A0A0A0N	Q5BKZ1	DBIRD complex su	ZNF326
24.0977	24.0241	24.3422	24.5698	24.3487	23.9575	NaN	NaN	NaN	24.308	24.444	NaN	Q8NB15;Q8NB15-	Q8NB15;Q8NB15-	Zinc finger protein	ZNF511
25.1984	24.2654	24.5942	25.2089	24.0943	24.3697	24.1961	24.0999	NaN	24.4367	24.2401	24.4554	Q969S3	Q969S3	Zinc finger protein	ZNF622
27.6486	23.0521	24.7809	28.8018	28.5506	NaN	NaN	29.7657	32.2774	NaN	30.2147	31.7101	Q14966;Q14966-4	Q14966;Q14966-4	Zinc finger protein	ZNF638
NaN	NaN	NaN	25.0576	NaN	NaN	NaN	NaN	NaN	NaN	NaN	NaN	Q0VGE8	Q0VGE8	Zinc finger protein	ZNF816
22.1995	22.1509	21.9584	22.9495	NaN	22.0311	NaN	NaN	NaN	22.3574	NaN	NaN	Q9P2E3;Q5JXR6;C	Q9P2E3;Q5JXR6	NFX1-type zinc fin	ZNFX1
22.0819	NaN	NaN	NaN	NaN	NaN	NaN	NaN	NaN	NaN	NaN	NaN	Q8NHG8	Q8NHG8	E3 ubiquitin-prote	ZNRF2
26.3744	26.9965	26.4891	26.2712	26.8458	26.0986	26.7343	26.2151	26.6016	26.3458	26.3432	26.9599	O75312;H7C0E5;	O75312;H7C0E5;	Zinc finger protein	ZPR1
22.0198	NaN	21.4679	21.621	NaN	21.7631	NaN	NaN	NaN	NaN	NaN	NaN	A7E2V4;H7BZ52;S	A7E2V4;H7BZ52;S	Zinc finger SWIM	ZSWIM8
25.7899	25.345	25.8186	25.9916	25.7789	25.6877	25.5834	26.1878	25.1558	25.8567	25.7875	25.9267	O43264;O43264-2	O43264;O43264-2	Centromere/kinet	ZW10
23.9242	23.2558	23.4722	23.5185	23.6506	NaN	23.921	24.2318	NaN	23.6539	23.7259	23.7591	Q9H900;Q9H900-	Q9H900;Q9H900-	Protein zwilch hor	ZWILCH
21.9667	22.268	22.3281	22.0492	21.8981	NaN	NaN	NaN	NaN	NaN	NaN	NaN	O95229;O95229-2	O95229;O95229-2	ZW10 interactor	ZWINT
NaN	NaN	NaN	21.8068	NaN	NaN	NaN	NaN	NaN	NaN	NaN	NaN	Q9C0D3;A8DPD7	Q9C0D3;A8DPD7	Protein zyg-11 hor	ZYG11B
26.5263	27.2822	26.9121	26.1847	27.334	26.8748	26.7535	27.2245	26.9024	26.3877	26.6334	27.3546	Q15942;H0Y2Y8;C	Q15942;H0Y2Y8;C	Zyxin	ZYX
NaN	NaN	NaN	NaN	NaN	NaN	NaN	NaN	NaN	19.814	NaN	NaN	O43149	O43149	Zinc finger ZZ-type	ZZEF1
27.7958	27.4612	27.9247	27.3566	27.6848	26.8965	27.9494	27.4398	27.5699	28.0121	NaN	27.8338	A0A0U1RQN6	A0A0U1RQN6		
NaN	NaN	NaN	NaN	NaN	NaN	NaN	NaN	NaN	NaN	NaN	28.2352	A4GXA9-2	A4GXA9-2		
27.1928	27.9985	27.2395	27.4371	28.1899	27.6156	26.0963	NaN	27.5096	26.621	27.1219	26.9463	B4DLN1;F6QW41	B4DLN1		
25.7932	25.7403	27.0376	26.552	26.5982	27.5303	26.6958	26.9113	26.5823	27.8702	27.147	26.7475	H3BN98;Q15041;	H3BN98		
NaN	23.4549	23.0162	NaN	24.338	NaN	NaN	NaN	25.1427	NaN	22.9143	NaN	M0QYT0;M0R076	M0QYT0		
NaN	NaN	NaN	24.6428	NaN	NaN	NaN	NaN	NaN	NaN	NaN	NaN	P05112-2	P05112-2		
